# Multivariate approach of metal-chiral covalent organic frameworks for leaching-resistant asymmetric transfer hydrogenation

**DOI:** 10.1038/s41467-026-78066-y

**Published:** 2026-09-25

**Authors:** Zhu Yin, Jun Lin, Xinru Gong, Yingqi Wang, Beili Lu, Xiong Chen, Jiajia Cheng

**Affiliations:** 1 State Key Laboratory of Chemistry for NBC Hazards Protection, College of Chemistry, Fuzhou University, Fuzhou, China; 2 State Key Laboratory of Photocatalysis on Energy and Environment, College of Chemistry, Fuzhou University, Fuzhou, China; 3 College of Material Engineering, Fujian Agriculture and Forestry University, Fuzhou, China

**Keywords:** Polymers, Polymers, Metal-organic frameworks

## Abstract

Achieving precise integration of chiral microenvironments and catalytic metals in covalent organic frameworks (COFs) remains a formidable challenge, often resulting in amorphous materials with poor stability and metal leaching. Here, we introduce a modular multivariate approach strategy coupled with post-synthetic metalation to engineer metal-chiral COFs (MCCOFs) with tunable crystallinity, porosity, and stereocontrol. Co-condensation of chiral Boc-protected diamines with variable achiral diamine ratios and tritopic aldehydes produced three crystalline frameworks (CCOFX-Boc, X = 0–2). Ruthenium metalation afforded CCOF2-Ru, which catalyzes asymmetric transfer hydrogenation of ketones with >99% yield, 97% ee, and a TOF of 1104 h^−1^. The catalyst exhibits broad substrate tolerance, including pharmacologically relevant chroman-4-ols, and maintains ≥95% ee over 5 gram-scale cycles with minimal Ru leaching (0.1 wt%). This approach upgrades unstable binary COFs into robust MCCOFs, enhancing activity, enantioselectivity, and durability via optimized pore architecture. It establishes a versatile platform for leaching-resistant chiral catalysts, advancing sustainable enantiopure pharmaceutical synthesis.

## Abstract (web summary)

Achieving precise integration of chiral microenvironments and catalytic metals in covalent organic frameworks (COFs) remains challenging. Here the authors introduce a modular multivariate approach strategy coupled with post-synthetic metallization to engineer metal-chiral COFs with tunable crystallinity, porosity, and stereocontrol.

## Introduction

Covalent organic frameworks (COFs) represent a transformative class of crystalline porous polymers, assembled through robust covalent linkages between organic building blocks, offering exceptional tunability for applications in gas separation, energy storage, sensing, and catalysis^1–17^. Among these, chiral COFs (CCOFs) stand out for their potential in asymmetric transformations^18–26^, leveraging ordered nanochannels and precisely positioned stereogenic centers to achieve high enantioselectivity^27–29^. Recent advances have demonstrated CCOFs’ efficacy in enantiomeric separations and circularly polarized luminescence, yet their deployment in heterogeneous asymmetric catalysis remains underexplored, limited by challenges in incorporating catalytic metals without compromising framework integrity^30–39^.

The design of metal-chiral COFs (MCCOFs) promises to merge the stereocontrol of molecular catalysts with the recyclability of heterogeneous systems, but persistent hurdles impede progress. Direct one-pot synthesis or subcomponent self-assembly often yields low-crystallinity materials due to steric clashes and incompatibility between bulky chiral units and metal precursors, which disrupt π-π stacking and result in amorphous or structurally unstable products^40–42^. For instance, recently developed MCCOFs synthesized via one-pot methods achieve high enantioselectivity (ee) but exhibit limited recyclability ( > 6% ee loss over five cycles) due to structural degradation. Post-synthetic metalation offers greater flexibility by coordinating metals to pre-formed chiral scaffolds^33,43–46^. However, deprotection processes often destabilize pre-formed chiral frameworks (as in Boc/Me-excised binary COFs)^47^, while metal leaching during catalysis undermines long-term stability^48,49^. Existing MCCOFs, though promising, suffer from these issues, resulting in suboptimal activity, enantioselectivity, and recyclability—critical barriers for industrial applications like chiral pharmaceutical synthesis. These limitations—sluggish kinetics (>24 h reaction time), metal leaching (>0.1 wt%/cycle), and attenuated enantioselectivity (>6% ee loss per cycle)—compromise scalability and industrial viability, particularly for reactions like asymmetric synthesis (expanded in Supplementary Table 13).

**Fig. 1:**
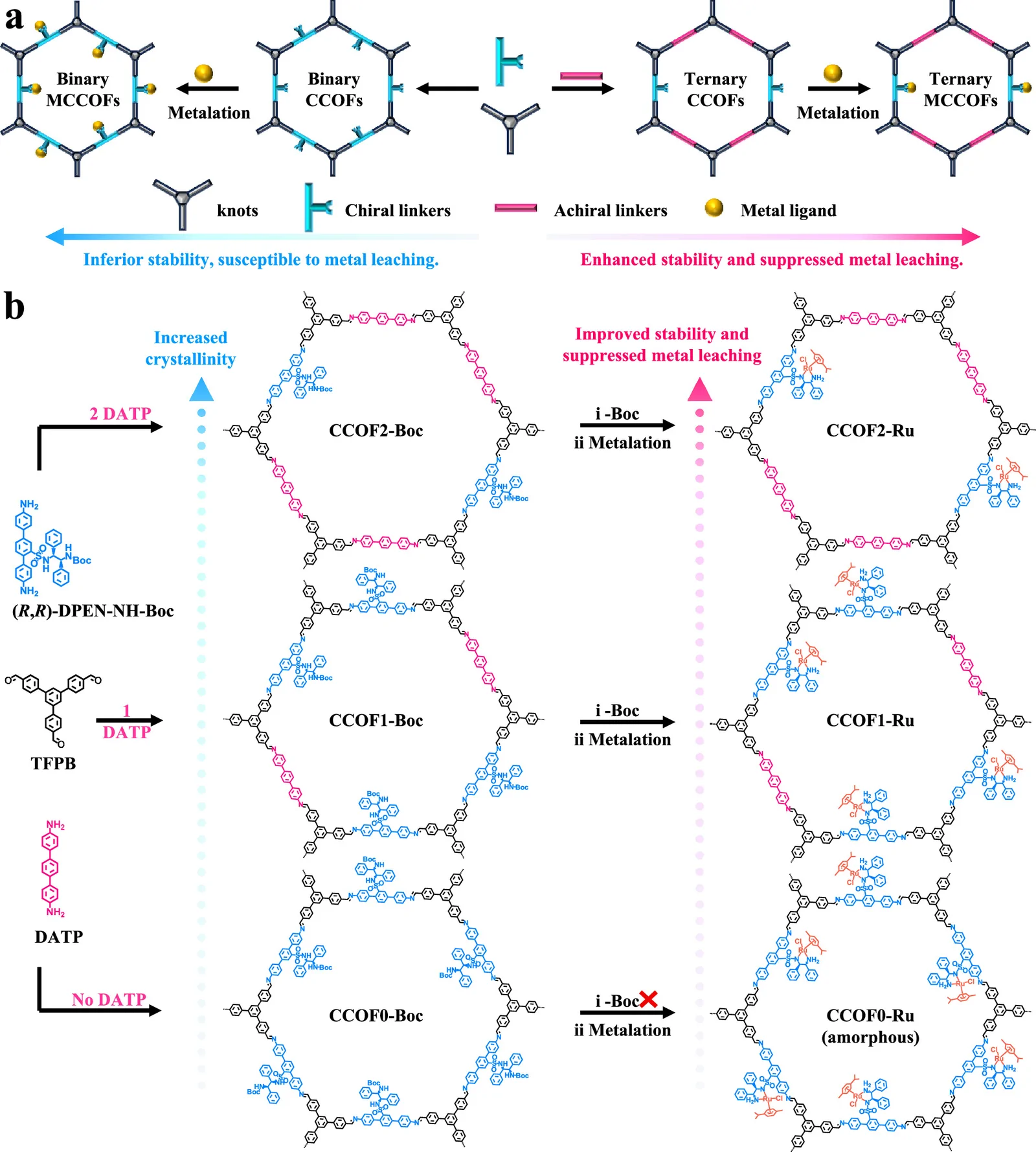
Construction of MCCOFs via post-synthetic metalation. **a** A comparison of binary and ternary MCCOFs**. b** Synthesis of binary and ternary MCCOFs, highlighting DATP incorporation for tunable chirality, enhanced stability, and suppressed metal leaching. -Boc denotes the deprotection step that removes the Boc protecting group.

To overcome these limitations, we report a modular strategy that integrates a multivariate approach with post-synthetic metalation, enabling the construction of MCCOFs with precisely controlled crystallinity, porosity, and chiral density (Fig. 1a). By co-condensing chiral Boc-protected diamines with variable ratios of achiral diamines and tritopic aldehydes, we engineered ternary frameworks (CCOFX-Boc, X = 0–2) that “upgrade” disordered binary precursors into ordered, stable structures resistant to amorphization during deprotection. This multivariate approach enhances π-π interactions, enlarges pores (from ~2 nm to ~3 nm), and bolsters chemical stability, transforming disordered polymers into robust crystalline materials^47,50–57^. Asymmetric transfer hydrogenation (ATH) of ketones is a classic transformation. However, the development of robust heterogeneous systems that can approach the microenvironmental precision of homogeneous catalysts—while offering superior recyclability and site isolation—remains a formidable yet highly active research frontier^58,59^. Ruthenium incorporation yields CCOF2-Ru, which excels in asymmetric transfer hydrogenation (ATH) of ketones, delivering quantitative yields, up to 97% ee, and a turnover frequency of 1104 h^−1^ across diverse substrates, including sterically demanding and heterocyclic ketones. Remarkably, the catalyst sustains performance over 5 gram-scale cycles with negligible metal leaching (0.1 wt%), attributed to the stabilized framework and high-density chiral sites that recapture dissociated metals. This result surpasses binary limitations and recent benchmarks: for example, our optimized CCOF2-Ru achieves a TOF of 1104 h^−1^ while maintaining ≥95% ee over 5 cycles with minimal leaching (0.1 wt%), attributed to the third component’s stabilizing role. This work establishes a versatile paradigm for MCCOF design, addressing key pain points in heterogeneous asymmetric catalysis. By upgrading binary systems to stable ternary architectures, our strategy not only boosts catalytic metrics but also paves the way for scalable, sustainable production of enantiopure compounds, with broad implications for pharmaceutical and fine chemical industries.

## Results

### Synthesis and structural characterization

**Fig. 2:**
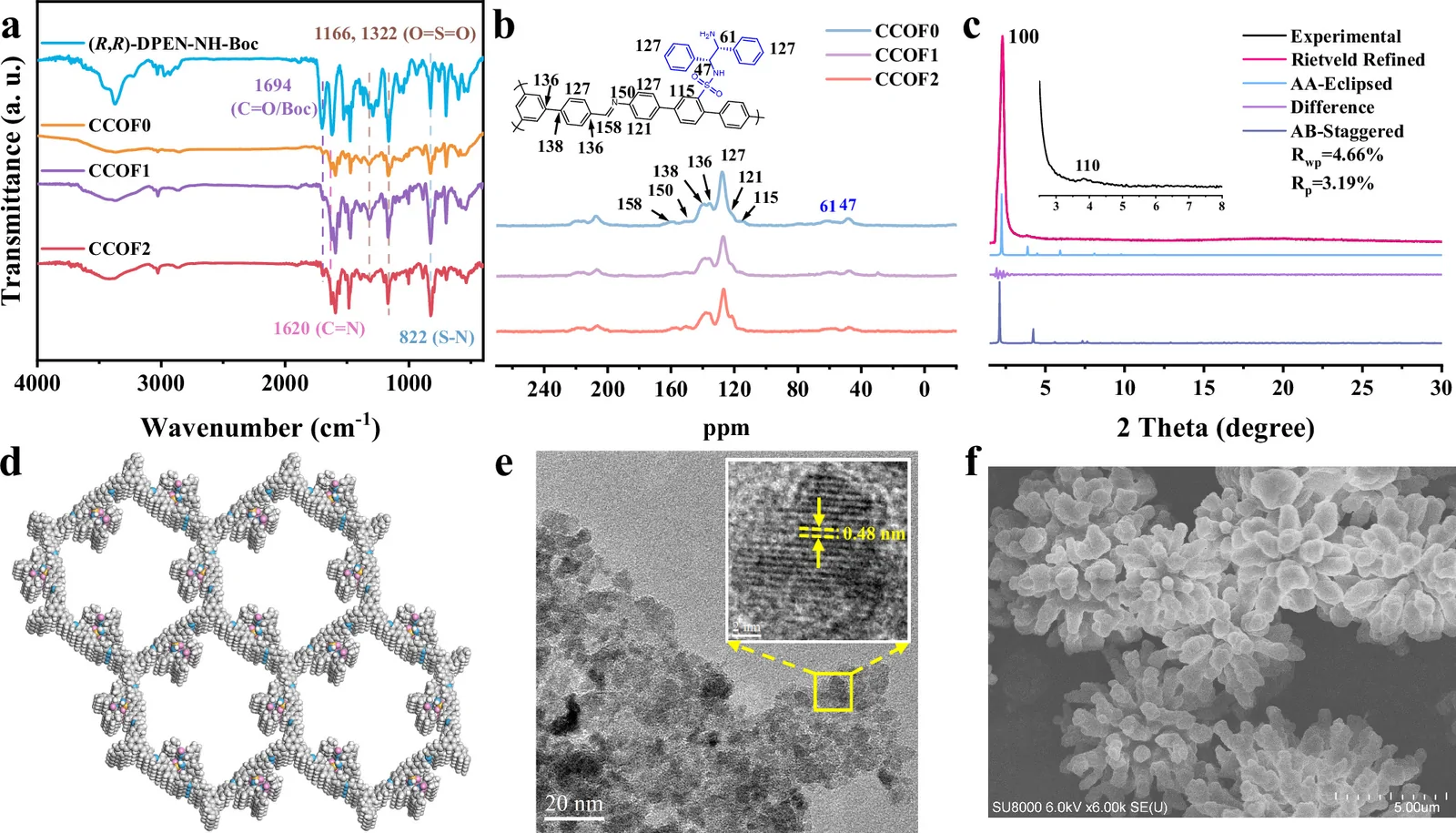
Structural characterization of CCOFs. **a** FTIR spectra and **b** solid-state ^13^C CP/MAS NMR spectra of CCOFX. **c** Experimental and model-based PXRD profiles of CCOF0-Boc, **d** simulated AA stacking crystal structure of top view (gray, C; blue, N; red, O; yellow, S; white, H), **e** HRTEM image and SEM image **f** of CCOF0-Boc.

To accommodate bulky Ru complexes while preserving substrate diffusion pathways, we designed CCOFs with enlarged pores through a multivariate approach strategy. As shown in Fig. 1b, one binary and two ternary CCOFs were prepared using 1,3,5-Tris(4-formylphenyl)benzene (TFPB) as knots and tert-butyl ((1*R*, 2*R*)−2-((4,4”-diamino-[1,1’:4’,1”-terphenyl])−2’-sulfonamido)−1,2-diphenylethyl) carbamate ((*R,R*)-DPEN-NH-Boc) as linkers. Binary CCOF0-Boc was synthesized via solvothermal condensation of TFPB (0.12 mmol) and (*R, R*)-DPEN-NH-Boc (0.18 mmol) in mesitylene/1,4-dioxane (4:1 v/v) with 3 M acetic acid at 100 °C for 72 h. However, Boc deprotection with TMSOTf yielded amorphous CCOF0, likely due to supramolecular interactions between deprotected amines and imine groups that disrupt interlayer π–π stacking^47,60^. Direct synthesis using unprotected chiral diamines also failed, producing amorphous powders. In contrast, co-condensation of TFPB (0.12 mmol) with varying 4,4”-Diamino-p-terphenyl (DATP) and (*R, R*)-DPEN-NH-Boc ratios afforded crystalline ternary CCOF1-Boc and CCOF2-Boc (Supplementary Fig. 1), which retained high crystallinity post-deprotection (yielding CCOF1 and CCOF2). This multivariate approach, by incorporating an achiral spacer, strengthens π-π interactions and stabilizes frameworks against amorphization—a key innovation enabling robust MCCOFs for catalysis^47^. The molecular-level structures of the synthesized CCOFs were characterized using Fourier-transform infrared spectroscopy (FTIR) and solid-state NMR spectroscopy. FTIR spectroscopy confirmed imine formation (1620 cm^−1^ stretch) and chiral group incorporation (O = S = O at 1166/1322 cm^−1^, S-N at 822 cm^−1^; Supplementary Fig. 2), with Boc removal evidenced by loss of the 1694 cm^−1^ C = O peak (Fig. 2a). Solid-state ^13^C cross-polarization magic-angle spinning (CP/MAS) NMR corroborated imine carbons (~158 ppm) and signals associated with the incorporated DPEN-derived unit near approximately 62 and 49 ppm (Supplementary Fig. 3). The Boc-protected samples additionally exhibit resonances near approximately 156, 80, and 29 ppm, assigned to the carbamate carbonyl, quaternary carbon, and tert-butyl methyl carbons, respectively. These Boc-associated signals decrease markedly or disappear following deprotection, whereas the DPEN-derived carbon signals remain, supporting Boc removal without loss of the chiral linker (Fig. 2b). Elemental analysis validated achiral linker integration, with N/S ratios of 5.55 (CCOF1-Boc) and 7.71 (CCOF2-Boc) matching theoretical values (5 and 8; Supplementary Table 1). This supports an incorporation level near the intended bulk composition, which is essential for uniform chiral environments and efficient metal coordination in catalysis.

**Fig. 3:**
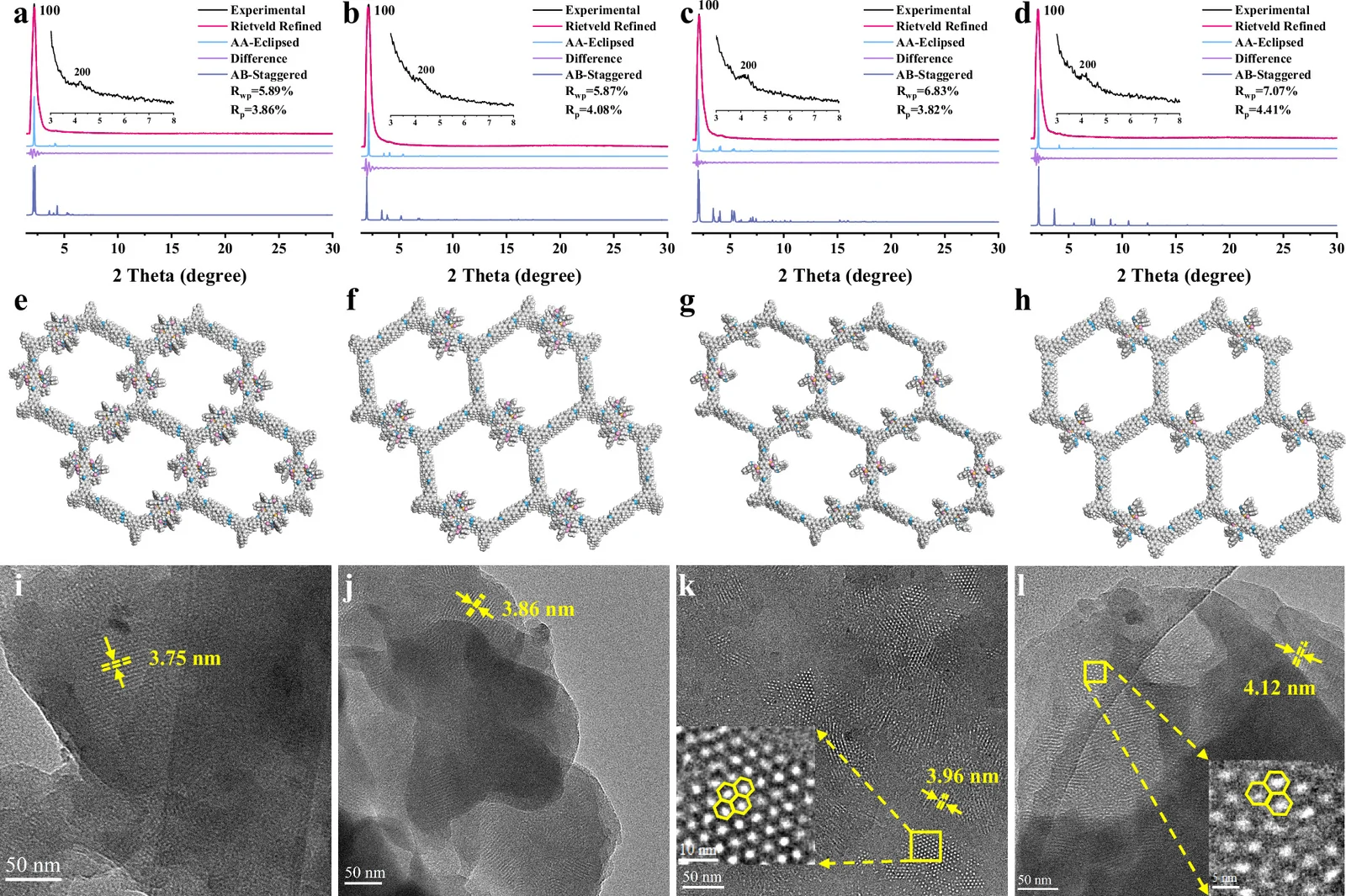
PXRD analysis, structural models and HR-TEM images of ternary CCOFs. Experimental PXRD patterns of **a** CCOF1-Boc, **b** CCOF2-Boc, **c** CCOF1, and **d** CCOF2. Simulated AA stacking crystal structure of top views of **e** CCOF1-Boc, **f** CCOF2-Boc, **g** CCOF1, and **h** CCOF2 (gray, C; blue, N; red, O; yellow, S; white, H). TEM images: **i** CCOF1-Boc, **j** CCOF2-Boc, **k** CCOF1, and **l** CCOF2.

Powder X-ray diffraction (PXRD) analysis supports periodic structural order of the CCOF series. The Boc-protected variants (CCOF0-Boc, CCOF1-Boc, and CCOF2-Boc) displayed intense low-angle diffraction peaks at 2.26°, 2.20°, and 2.12°, respectively, corresponding to the (100) reflection, together with weak higher-order reflections for selected samples (Supplementary Fig. 4). The long-range order of these frameworks is evidenced by the narrow full width at half maximum (FWHM) values of the (100) plane (0.44°–0.51°). Rietveld refinements using AA-eclipsed structural models provide satisfactory agreement with the experimental patterns, as reflected by the residual factors summarized in Supplementary Table 3. For the binary CCOF0-Boc, comparison of experimental profiles with simulated patterns (Fig. 2c) supports its assignment to a hexagonal 2D network with an eclipsed AA stacking arrangement (space group *P*3, Fig. 2d). A weak diffraction peak near 3.82° in the inset of Fig. 2c is consistent with the calculated position of the (110) reflection of the proposed model. The refinement yielded unit-cell parameters of a = b = 45.4766 Å, c = 4.8584 Å, α = β = 90°, and γ = 120°, in good agreement with the theoretical model, with low residual factors (Rp = 3.19% and Rwp = 4.66%). High-resolution transmission electron microscopy (HRTEM) provided corroborating local structural insight (Fig. 2e and Supplementary Fig. 5); distinct lattice fringes (Fig. 2e) revealed an interplanar spacing of 0.48 nm (inset), consistent with the d_001_ spacing expected for AA stacking. Morphologically, field-emission scanning electron microscopy (FE-SEM) revealed a hierarchical, sea urchin-like texture for CCOF0-Boc (Fig. 2f). Similarly, the ternary analogues (CCOF1-Boc through CCOF2) are most consistent with the evaluated AA-eclipsed stacking models within hexagonal 2D networks (space group P2_1_), as supported by Rietveld refinements (Fig. 3a–h, Supplementary Fig. 6 and Supplementary Tables 3–8). The weak diffraction peaks at 4.20°, 4.18°, 4.18°, and 4.16° in the insets of Fig. 3a–d can be assigned to the (200) reflections of CCOF1-Boc, CCOF2-Boc, CCOF1, and CCOF2, respectively. The structural integrity and stacking modes of CCOF1 and CCOF2 were largely preserved following Boc deprotection. HRTEM imaging (Fig. 3i–l) further highlighted the honeycomb-like porosity and layered architecture of the ternary systems, offering complementary local evidence consistent with the proposed ordered porous structures. The honeycomb-like channels observed in the HRTEM images of CCOF1 and CCOF2 (Fig. 3k, l) are consistent with the proposed AA-eclipsed hexagonal arrangement and are less compatible with a fully AB-staggered model, which would be expected to substantially reduce or distort the open hexagonal channels in projection.

Nitrogen adsorption-desorption isotherms at 77 K yielded Brunauer-Emmett-Teller (BET) surface areas of 21, 70, and 518 m^2^ g^−1^ for CCOF0-Boc, CCOF1-Boc, and CCOF2-Boc, respectively (Supplementary Fig. 7). As shown in Supplementary Table 2, the experimentally measured BET surface areas and pore volumes are lower than those calculated using Zeo++ from the idealized structural models. This discrepancy is most pronounced for CCOF0-Boc and CCOF1-Boc and is mainly attributed to the difference between ideal, defect-free theoretical models and real powder samples containing bulky Boc-protected chiral side chains, reduced long-range order, local defects, and partially inaccessible channels. The bulky chiral units occupy part of the internal pore volume and may weaken interlayer π–π interactions, thereby reducing structural order and channel accessibility^61,62^. Importantly, the reduced accessible porosity does not necessarily imply a global AB-staggered stacking arrangement. Comparison of optimized AA-eclipsed and AB-staggered models shows that the experimentally observed pore-size distributions are more consistent with the AA-eclipsed models, particularly for the more crystalline CCOF2-Boc and CCOF2. The significantly higher BET surface area of CCOF2-Boc is therefore consistent with its enhanced crystallinity and supports the role of the multivariate strategy in improving framework order and pore accessibility. This steric-driven limitation is further corroborated by our control experiment with CCOF-DACH-Boc (Supplementary Fig. 24), which demonstrates that reducing the steric bulk of the chiral groups significantly restores crystallinity under identical synthetic conditions. Pore size distributions centered at ~2 nm for CCOF0-Boc and CCOF1-Boc, broadening to ~3 nm for CCOF2-Boc, reflecting the enhanced pore accessibility of the multivariate framework. Post-deprotection, BET areas decreased slightly to 14, 40, and 473 m2 g-1 for CCOF0, CCOF1, and CCOF2, with CCOF0 showing reduced porosity (2–3 nm) due to loss of crystallinity^63,64^. FE-SEM revealed sea urchin-like morphologies for CCOF0-Boc and CCOF0 (Fig. 2f and Supplementary Fig. 8a, b), contrasting with the irregular blocks of ternary CCOF1-Boc/CCOF1 and CCOF2-Boc/CCOF2 (Supplementary Fig. 8c–f). Thermogravimetric analysis (TGA) was also performed to evaluate the thermal stability of the CCOFs. As shown in Supplementary Fig. 9, the Boc-protected CCOF samples exhibit an obvious mass loss starting at approximately 210 °C that is markedly reduced in the corresponding deprotected samples, attributed to thermal deprotection of the Boc groups. A further mass-loss region near approximately 275 °C is observed for the chiral CCOF samples, but is less evident for the TFPB-DATP control, which lacks the chiral sulfonamide-containing component. This feature is therefore tentatively associated with the decomposition of the pendant chiral sulfonamide-containing groups^65,66^. At temperatures above approximately 480 °C, all frameworks, including the CCOFs and the TFPB-DATP control, undergo substantial mass loss, which is consistent with major framework decomposition and carbonization under nitrogen. Taken together, the PXRD, HRTEM, and gas-sorption results support the formation of porous 2D frameworks with periodic in-plane order. The combined experimental data are most consistent with the evaluated AA-eclipsed stacking model, although small layer offsets, stacking faults, and local disorder cannot be excluded. A more definitive determination of the local stacking arrangement will require future higher-resolution structural characterization, such as synchrotron powder diffraction, total-scattering/pair-distribution-function analysis, or three-dimensional electron diffraction.

A pivotal innovation of our approach is the incorporation of an achiral third component (DATP), which circumvents amorphization—a ubiquitous limitation in binary chiral COFs—while enabling unprecedented structural control and catalytic enhancement^67,68^. Conventional binary systems, such as CCOF0, succumb to crystallinity loss upon Boc deprotection due to disruptive supramolecular interactions between exposed amines and imine linkages, weakening π-π stacking (FWHM broadening from 0.509° to disordered). In contrast, DATP acts as a spacer in ternary CCOF1-Boc and CCOF2-Boc, diluting chiral site density. DATP incorporation correlates with sharper low-angle diffraction features (FWHM reduced to 0.440° in CCOF2-Boc; Supplementary Fig. 4), increased accessible porosity, and improved structural retention after deprotection. This multivariate strategy not only enhances crystalline quality—evidenced by sharper PXRD peaks (Supplementary Fig. 4)—but also imparts superior chemical stability: CCOF2 retained >95% mass and crystallinity after exposure to 0.1 M HCl, 1 M KOH, or 1 M HCOONa in H_2_O/^*i*^PrOH (60 °C; Supplementary Figs. 10 and 11), far outperforming CCOF0 (>20% mass loss) and CCOF1 (partial hydrolysis with aldehyde peak intensification at 1694 cm^−1^; Supplementary Fig. 12)

### Metalation of CCOFs and catalytic performance

**Fig. 4:**
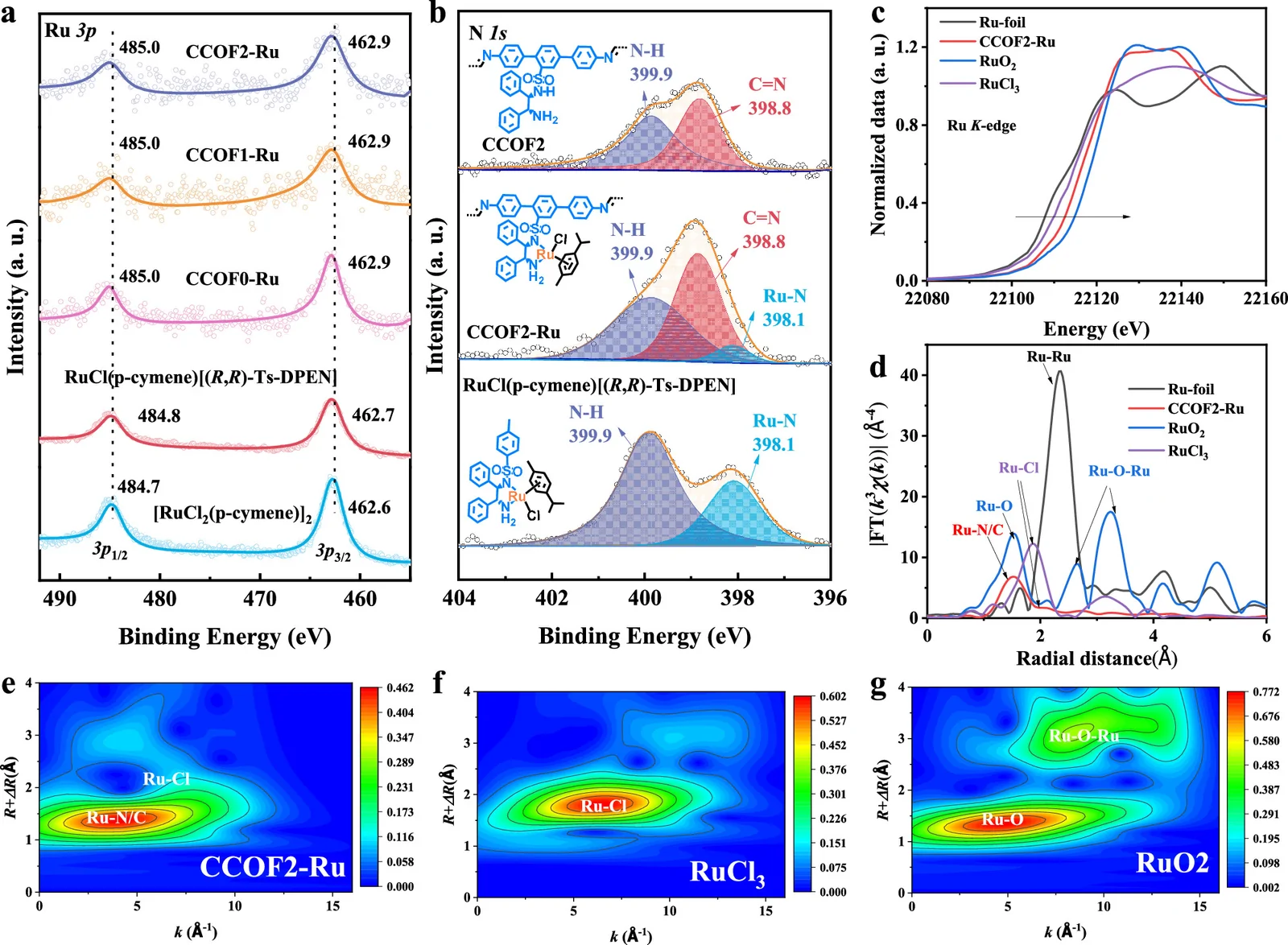
Structural investigation of CCOF2-Ru. XPS spectra of **a** Ru 3p and **b** N 1s. **c** XANES spectra, **d** EXAFS of CCOF2-Ru. Wavelet transform of **e** CCOF2-Ru, **f** RuCl_3_ and **g** RuO_2_.

Bridging the gap between the atomic precision of homogeneous Noyori-type catalysts and the robustness of heterogeneous supports remains a formidable challenge. Specifically, embedding chiral Ru–N active sites without compromising the framework’s structural integrity or the catalyst’s intrinsic activity is non-trivial^69,70^. To address this, we employed a ternary multivariate strategy to construct periodic MCCOFs that stabilize Ru–diamine centers within a chiral sulfonyldiamine microenvironment. Post-synthetic metalation with [Ru(p-cymene)Cl_2_]_2_ yielded the MCCOF series (CCOF0-Ru, CCOF1-Ru, CCOF2-Ru). Crucially, the multivariate architecture dictates the structural outcome: while the binary CCOF0 analog suffers significant crystallinity loss upon deprotection and metalation, the ternary CCOF2-Ru retains high crystallinity and porosity (Supplementary Figs. 13 and 14). This stark contrast validates our hypothesis that introducing a third component regulates the density of coordination sites, preventing the structural collapse often observed in binary systems. Quantitative analysis via ICP-OES (Supplementary Table 9) confirmed tunable Ru loadings (0.89–2.12 wt%). HRTEM demonstrated that the ordered pore architectures of CCOF1-Ru and CCOF2-Ru were well-maintained after Ru coordination. Furthermore, high-angle annular dark-field scanning transmission electron microscopy (HAADF-STEM) coupled with corresponding energy-dispersive X-ray spectroscopy (EDS) mapping revealed a highly homogeneous distribution of Ru, C, N, and O elements throughout the COF matrix, with no observable metal nanoparticle aggregation (Supplementary Fig. 15). This confirms the uniform integration of the catalytic Ru sites within the framework. The Raman spectra (Supplementary Fig. 17b) confirm that CCOF2-Ru retains the same framework structure as CCOF2 after Ru coordination. Furthermore, the characteristic Ru-N peak was not observed. This is consistent with recent literature^71^ and is attributed to the ultra-low Ru loading (0.89 wt%) combined with the inherently low Raman scattering cross-section of Ru-N bonds. To rigorously confirm the coordination mode, we utilized X-ray photoelectron spectroscopy (XPS). The Ru *3p* binding energies in the MCCOFs shifted to 462.9 and 485.0 eV (distinct from the 462.6 and 484.7 eV of the precursor), precluding the presence of physisorbed species (Fig. 4a). The high-resolution N *1s* spectrum of CCOF2-Ru reveals that the C = N signal at 398.8 eV remains unchanged compared to that of CCOF2 (Fig. 4b). Upon Ru coordination, a new signal emerges at 398.1 eV, corresponding to the Ru–N species, alongside a feature at approximately 399.9 eV attributed to N–H. To ensure the accuracy of this assignment, we measured a commercially available RuCl(p-cymene)[(*R, R*)-Ts-DPEN] complex as a standard reference, which exhibited identical binding energies for the Ru-N and N-H species. To further investigate the chemical state and coordination environment of the CCOF2 Ru catalyst, X-ray absorption fine structure (XAFS) analysis was conducted. As shown in Fig. 4c, by comparing the Ru K-edge X-ray absorption near-edge structure (XANES) spectra of CCOF2 Ru with those of RuCl_3_ and RuO_2_, the Ru K-edge position of CCOF2-Ru lies between those of the selected RuCl_3_ and RuO_2_ references. As presented in Fig. 4d, analysis of the extended X-ray absorption fine structure (EXAFS) data reveals two distinct peaks in the Fourier transform (FT) EXAFS spectrum of CCOF2 Ru, attributed to Ru–N/C (1.53 Å) and Ru–Cl (2.02 Å) bonds. No Ru–Ru scattering path or Ru nanoparticles were detected within the sensitivity of EXAFS and TEM. Furthermore, wavelet transform analysis of the EXAFS spectra further confirms the presence of Ru–N/C and Ru–Cl coordination structures (Fig. 4e–g). Using a coordination shell composed of chloride, p-cymene, and a DPEN ligand around Ru as the initial structural model for EXAFS curve fitting of the CCOF2 Ru samples, with atomic coordinates taken from the crystal structure previously reported by Dub et al^72^. (COD ID: 4128180), the FT *R*-space EXAFS spectra (Supplementary Fig. 19) together with the fitting results (Supplementary Table 10) indicate the presence of a first coordination shell comprising Ru–N/C (bond length: 2.159 Å, coordination number (CN) ≈ 7.7) and Ru–Cl (bond length: 2.436 Å, CN ≈ 0.9) in CCOF2-Ru. The total coordination number is consistent with the expected structure of CCOF2 Ru (Fig. 1b), namely CN = 1 for Ru–Cl, CN = 2 for Ru–N, and CN = 6 for Ru–C. These spectroscopic signatures confirm that the Ru centers are anchored via the chiral N–H motifs, successfully replicating the active site of homogeneous catalysts within a robust, versatile, and scalable heterogeneous scaffold. This strategy could also be readily extended to various metal centers (e.g., Rh and Ir; Supplementary Fig. 20), thus showcasing its versatility.

To understand the specific role of the chiral diamine pockets, we investigated the coordination behavior of the control framework, TFPB-DATP (Supplementary information Section 2.9; Supplementary Figs. 21 and 22). ICP-OES revealed a remarkably low Ru loading of only 0.17 wt% in TFPB-DATP-Ru, indicating that the pristine imine backbone lacks strong coordination sites for the metal precursor. The combined EXAFS and XPS results (consistent with an arene–Ru–Cl coordination environment containing Ru–N interactions) and the low Ru uptake of TFPB-DATP support preferential coordination at the deprotected diamine-containing sites. High-resolution N 1s XPS analysis of TFPB-DATP-Ru (Supplementary Fig. 22) displayed a binding energy shift for the imine C = N moiety, suggesting that the trace Ru species merely coordinates to the backbone imine nitrogens. This weak and non-specific coordination sharply contrasts with the robust metalation achieved in CCOF2, thereby confirming the necessity of the engineered chiral microenvironment for efficient catalyst loading.

**Fig. 5:**
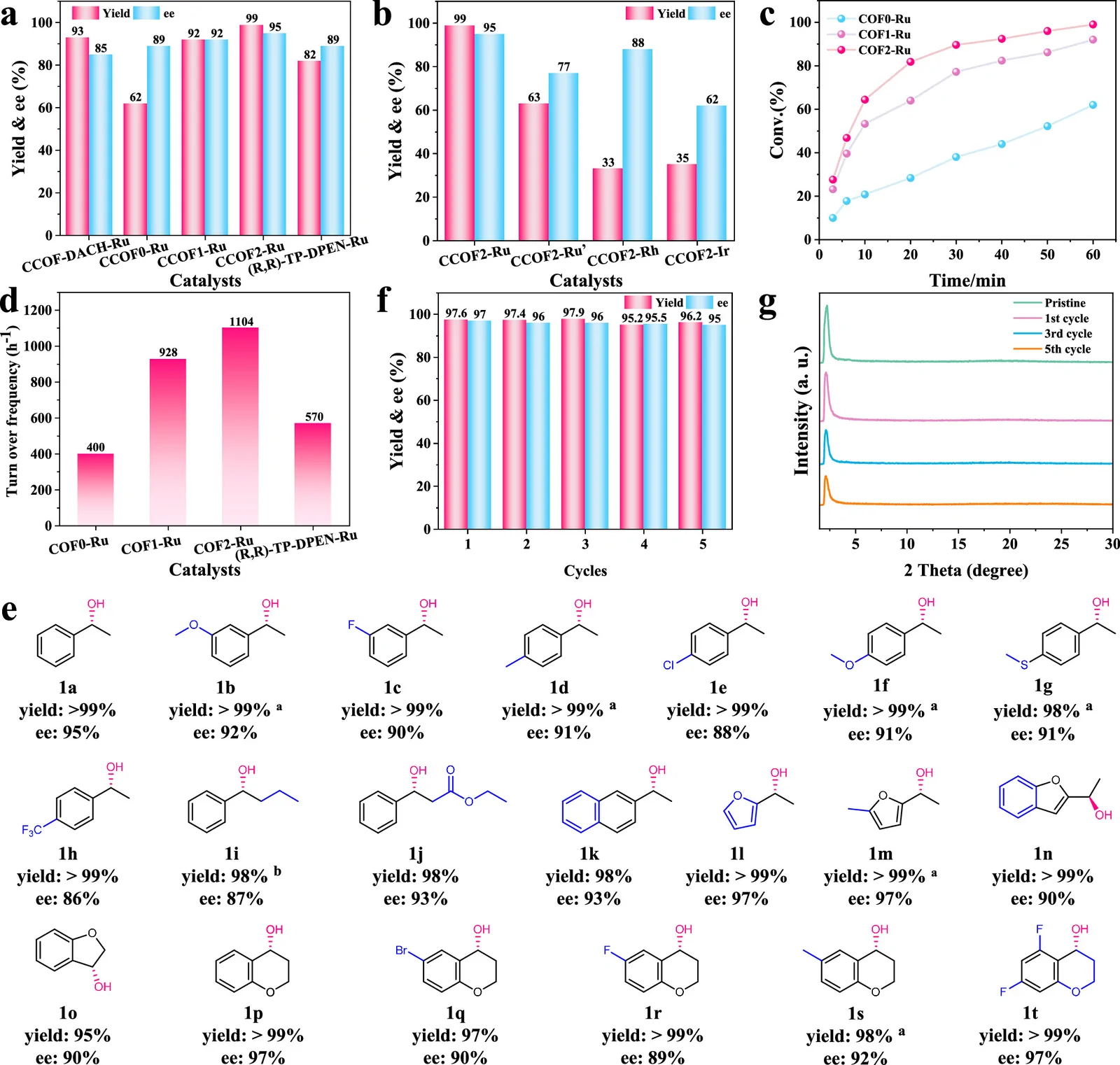
Catalytic performance evaluation of MCCOFs in ATH reaction. **a** Comparison of yield and ee across different catalysts. **b** Yield and ee for CCOF2-supported Ru, Rh, and Ir variants. **c** Kinetic profiles for ATH of acetophenone. **d** TOFs for selected catalysts (TOF was calculated at conversions below 30% using data collected after 3 min of reaction). **e** Substrate scope for CCOF2-Ru-catalyzed ATH reaction of ketones (Reaction conditions: ketone (0.2 mmol), HCOONa (1 mmol), CCOF2-Ru (0.5 mol% of Ru based on ICP analysis), N_2_ atmosphere, 1 mL ^*i*^PrOH/ H_2_O (1:1 v/v), 60 °C, 1 h unless noted. ^a^ 7 h, ^b^ 3 h). **f** Gram-scale synthesis of 1t over 5 reaction cycles. **g** PXRD patterns of pristine CCOF2-Ru and the recovered catalyst after 5 cycles.

The catalytic performance of solid catalyst CCOFX-Ru was evaluated in the ATH reaction of acetophenone, which is a benchmark reaction for producing enantiopure alcohols^73–76^. In acetophenone ATH, CCOF2-Ru delivered >99% yield and 95% ee within 1 h (TOF 1104 h^−1^), surpassing its homogeneous Ru analogue (570 h^−1^) and the binary, partially disordered CCOF0-Ru (400 h^−1^; Fig. 5a, d). Kinetic profiling establishes a clear structure–function relationship: ordered, ternary frameworks (CCOF1/2-Ru) are decisively faster than the binary control, with CCOF2-Ru driving near-quantitative conversion in 1 h (Fig. 5c). To evaluate the generality of CCOF2 as a versatile chiral platform and to systematically investigate the influence of different metal centers on the catalytic performance, we extended our post-synthetic metalation strategy to include other classic Noyori-type metal precursors. Specifically, we attempted the coordination of [Ru(η^6^-C_6_H_6_)Cl_2_]_2_, [CpRhCl_2_]_2_, and [CpIrCl_2_]_2_ onto the CCOF2 framework. The results reveal that this multivariate assembly exhibits excellent modularity: CCOF2 can uniformly stabilize Ru, Rh, and Ir metal centers via an identical protocol while fully retaining its crystalline architecture (PXRD, Supplementary Fig. 20). Among these isostructural metallo-COFs, CCOF2-Ru displays the highest catalytic activity and enantioselectivity for asymmetric transfer hydrogenation (Fig. 5b), highlighting the critical role of metal selection in optimizing the confined chiral microenvironment. Controls underscore the necessity of both site geometry and chiral environment: replacing diphenylethylenediamine with cyclohexanediamine (CCOF-DACH-Ru, Supplementary Figs. 20a and 23–26) depresses ee (85% vs 89% for CCOF0-Ru, Fig. 5a), and a deliberately disordered analogue (CCOF2-amp-Ru, Supplementary Table 10, entry 5) affords only 87% ee. Notably, the distinct catalytic performances of CCOF-DACH-Ru (ordered structure, low ee), CCOF-DPEN-Ru (disordered structure, moderate ee), and CCOF2-Ru (ordered structure, high ee) fully demonstrate the necessity of the multivariate approach in the design and synthesis of MCCOFs, as it elegantly balances the requirement for high enantioselectivity in asymmetric catalysis and the adverse impact of sterically hindered chiral functionalities on the structural ordering during MCCOF fabrication. Stringent leaching tests (Supplementary Table 11) confirm that CCOF2-Ru is a robust, genuinely heterogeneous catalyst: filtrates after pre-stirring show only trace activity, in contrast to minor residual activity from CCOF0/1-Ru, consistent with their lower stability. Benchmarking against state-of-the-art systems confirms the superiority of CCOF2-Ru: it achieves complete substrate conversion within 1 h with a TOF of 1104 h^−1^ while maintaining ≥95% ee and high activity over multiple cycles (expanded Supplementary Table 13). This performance surpasses most reported catalysts—including CCOFs, CMOFs, and polymer-based systems—for ATH or AH^19,48,58,77–84^. This enhanced activity stems from DATP-mediated pore expansion (~3 nm), which optimizes mass transport and active-site accessibility via ordered chiral pockets and enlarged mesopores that synergistically maximize site utilization efficiency.

Mechanistic probes support a Noyori-type inner-sphere cycle localized within the framework (Supplementary Fig. 29). Unmetalated CCOF2 is inactive (Supplementary Table 12, entry 6), and an achiral analogue lacking the sulfonyldiamine pocket (TFPB-DATP-Ru, Supplementary Figs. 21 and 22) shows no conversion (Supplementary Table 12, entry 7), indicating that both Ru–N coordination and the chiral pocket are indispensable. To further elucidate the mechanism of the asymmetric transfer hydrogenation of ketones catalyzed by CCOF2-Ru, we performed additional control and deuterium-labeling experiments. Reducing the catalyst loading leads to a decrease in yield (Supplementary Table 12, entry 8), and the yield progressively declines with decreasing amounts of sodium formate (Supplementary Table 12, entries 9–10), suggesting that sodium formate serves as the hydrogen source. When isopropanol was used as the solvent, both the yield and ee decreased significantly (Supplementary Table 12, entry 11, 41% yield, 86% ee). Using the polar aprotic solvent acetonitrile resulted in a marked reduction in yield (Supplementary Table 12, entry 12, 18% yield, 95% ee). These results indicate that water actively participates in the catalytic cycle.

The deuterium-labeling experiments further confirm the role of sodium formate. As shown in Supplementary Fig. 27, these experiments demonstrate that the hydrogen atoms originate predominantly from sodium formate, rather than from water or isopropanol. Furthermore, a kinetic isotope effect of *k*_*H*_*/k*_*D*_ = 3.0 was determined using a 1:1 molar mixture of DCO_2_Na and HCO_2_Na (conversion <10%, product deuteration level of 25%; Supplementary Figs. 27 and 28), which is in excellent agreement with the value reported for the homogeneous Noyori-type catalyst (*k*_*H*_/*k*_*D*_ = 3.2)^85^. These findings suggest that cleavage of the formate C–H/D bond contributes to the kinetically significant sequence. Furthermore, GC analysis of the reaction tube confirmed the presence of H_2_ and CO_2_. The formation of CO_2_ can be attributed to the β-hydride elimination of the CCOF2-Ru formate complex, which simultaneously generates the active Ru–H intermediate. Meanwhile, the evolution of H_2_ is likely associated with the Ru–H intermediate releasing H_2_ upon interaction with H^+^ from water.

Based on our experimental results and the reported mechanisms of Noyori-type catalysts, we propose a plausible reaction mechanism (Supplementary Fig. 29)^85–89^. Upon treatment with HCOONa, CCOF2-Ru undergoes ion exchange to generate the Ru–formate complex (CCOF2-Ru I). Subsequent β-hydride elimination affords the catalytically active Ru–H intermediate (CCOF2-Ru II). Stereoselective hydride transfer to the prochiral ketone substrate takes place within the confined chiral microenvironment of CCOF2-Ru, producing the Ru–diamine species (CCOF2-Ru III). In the presence of sodium formate and H_2_O, CCOF2-Ru III is converted back to CCOF2-Ru I, thereby entering the next catalytic cycle. The periodic mesoporosity ensures efficient substrate/product diffusion, enabling high site utilization and fast turnover.

### Substrate scope and cyclic reactions

The substrate scope of the ATH reaction was systematically investigated using a diverse library of ketone substrates (Fig. 5e). Under optimized conditions (HCOONa; ^*i*^PrOH/H_2_O = 1:1 v/v), the catalyst delivers broad substrate generality with consistently high stereocontrol (up to 97% ee) across a diverse ketone library. Under optimized conditions (HCOONa; ^*i*^PrOH/H_2_O = 1:1 v/v), the catalyst delivers broad substrate generality with consistently high stereocontrol (86–97% ee) across a diverse ketone library. Para-substituents modulate reaction kinetics in line with electronic effects: electron-deficient aryl ketones (-F, –Cl, –CF_3_, –COOEt) reach full conversion within 1 h, whereas electron-rich analogues (–OCH_3_, –SCH_3_, –CH_3_, –CH_2_CH_2_CH_3_) require 3–7 h for quantitative conversion (1b-1j). The rigid, open framework confers remarkable steric tolerance—bulky 2-ethylnaphthyl ketone affords 1k in 98% yield and 93% ee within 1 h. Moreover, excellent stereocontrol was achieved for furan derivatives (90–97% ee), including methyl- and phenyl-substituted substrates (1l–1o). The asymmetric transfer hydrogenation of 4-chromanone derivatives, which are widely found in nature and exhibit diverse biological activities, also proceeded with outstanding stereocontrol (89–97% ee), covering halogen (F, Br) and methyl substitutions (1p–1t). Showcasing pharmaceutical relevance, (*R*)−5,7-difluorochroman-4-ol (1t), a key Tegoprazan intermediate, is obtained in >99% yield and 97% ee within 1 h; gram-scale synthesis proceeds in 60 min with 97.6% isolated yield and 97% ee.

The catalyst combines stereo-retention with practical robustness (Fig. 5f). The gram-scale production of 1t was successfully achieved using CCOF2-Ru as a heterogeneous catalyst, delivering a 98% isolated yield with 97% ee within 60 min. Across five recycles, enantioselectivity remains ≥95% ee; only a modest activity decay appears after the third use, which is fully compensated by a 10–20 min extension in reaction time. To further verify the structural robustness of the catalyst under reaction conditions, the recovered CCOF2-Ru catalyst was thoroughly characterized after five consecutive catalytic cycles. The PXRD pattern of the recycled catalyst (Fig. 5g) demonstrated that the long-range crystalline order was fully retained. Additionally, FTIR spectroscopy (Supplementary Fig. 30a) confirmed that the chemical structure of the framework remained intact, with no observable degradation of the functional groups, highlighting the exceptional stability of the multivariate assembled MCCOFs. This durability stems from suppressed metal leaching, with the measured Ru content of the recovered catalyst decreased from 0.89 to 0.79 wt% over five cycles—superior to CCOF0-Ru (1.02 wt% after three cycles) and CCOF1-Ru (0.35 wt% after three cycles; Supplementary Table 15). In contrast to conventional polymer-supported catalysts, which often suffer >0.1 wt% leaching per cycle and require >200% time extensions for sustained yields due to active site decomposition (Supplementary Table 14), our multivariate framework minimizes degradation. Solvent resistance tests further validate that DATP incorporation bolsters chemical stability under harsh conditions (Supplementary Fig. 10–12 and Supplementary Table 11). Based on our ICP-OES analysis, a massive excess ( ~ 85%) of uncoordinated, structurally identical chelating sites is densely distributed along the 1D channels of the COF. The synthesis of CCOF2-Ru is straightforward (stirring at 40 °C without additional base), XPS (Fig. 4a, b) and EXAFS (Fig. 4d, e) alongside TEM-EDS mapping (Supplementary Fig. 15f) confirm the facile and uniform coordination of Ru to these sites. Because the initial coordination is thermodynamically favorable and kinetically rapid, any Ru species that transiently dissociates during the catalytic cycle will immediately encounter a highly concentrated local environment of these free bidentate chelating sites within the confined nanopores, driving rapid re-coordination^58,80^. These features—enabled by the ordered ternary architecture—establish a leaching-resistant paradigm, enhancing recyclability for industrial asymmetric synthesis without compromising performance.

## Discussion

In summary, we have developed a modular strategy that integrates a multivariate approach with post-synthetic metalation to construct MCCOFs with tunable crystallinity, porosity, and stereocontrol. By incorporating an achiral diamine spacer into binary chiral COFs, we overcame amorphization during deprotection, yielding stable ternary frameworks with enhanced π-π stacking and enlarged pores (~3 nm). Ruthenium integration afforded CCOF2-Ru, which excelled in ATH of ketones, delivering quantitative yields, up to 97% ee, and a TOF of 1104 h^−1^ across diverse substrates—including pharmacologically relevant chroman-4-ols. Notably, the catalyst sustained ≥95% ee over five gram-scale cycles with minimal Ru leaching (0.1 wt%), outperforming binary analogues and state-of-the-art benchmarks. This approach advances MCCOF design by transforming unstable binary systems into robust, leaching-resistant catalysts, addressing key limitations in heterogeneous asymmetric catalysis such as structural degradation and metal loss. The multivariate modulation not only boosts activity and enantioselectivity through optimized chiral microenvironments but also enhances scalability, as evidenced by extensions to Rh and Ir variants. These findings establish a versatile platform for sustainable chiral synthesis, with profound implications for industrial production of enantiopure pharmaceuticals and potential applications in other enantioselective transformations.

## Methods

### Preparation of CCOF2-Boc

Typically, 46.8 mg of TFPB (0.12 mmol), 31.2 mg DATP (0.12 mmol) and 38 mg (*R,R*)-DPEN-NH-Boc (0.06 mmol) were added to a 15 mL Pyrex tube. A mixture of 3 mL xylene and 1,4-dioxane (v/v ratio 4:1) was introduced as the solvent, followed by ultrasonic treatment for 5 min to ensure uniform dispersion of the components. Subsequently, 250 μL of 3 M acetic acid was added as a catalyst, and the mixture underwent additional ultrasonic treatment for 10 min to facilitate thorough mixing. The system was then degassed through three freeze-pump-thaw cycles to remove dissolved gases. The tube was sealed, and the mixture was heated at 120 °C for 3 days to allow the reaction to proceed to completion. After cooling, the resulting yellow-green powder was collected via filtration and washed sequentially with ethanol and dichloromethane. The powder was subsequently subjected to Soxhlet extraction using tetrahydrofuran (THF) and acetone for 24 h to achieve further purification. The final product was obtained by overnight vacuum drying at 60 °C. Yield: 92 mg (84%).

The synthetic procedures for the chiral Boc-protected diamine, as well as for CCOF0-Boc, CCOF1-Boc, and other comparative COFs, are described in the Supplementary Information.

### Preparation of CCOFX

CCOFX-Boc (300 mg) was charged into an oven-dried 25 mL round-bottom flask equipped with a magnetic stir bar. Under an ice-water bath (0 °C), anhydrous dichloromethane (6 mL) was introduced via syringe, followed by dropwise addition of trimethylsilyl trifluoromethanesulfonate (TMSOTf, 0.1 mL). The reaction mixture was maintained at 0 °C with vigorous stirring for 6 h. Upon completion, the suspension was filtered through a Büchner funnel. The collected solid was sequentially washed with cold dichloromethane, saturated aqueous NaHCO_3_ solution, deionized water and ethanol. The crude product was subsequently subjected to purification by Soxhlet extraction using acetone as eluent (24 h continuous extraction). Finally, the purified sample was dried under high vacuum at 80 °C for 12 h to afford CCOFX.

### Preparation of CCOFX-Ru

A mixture of CCOFX (200 mg), dichloromethane (DCM, 5 mL), and a stoichiometric amount of [Ru(p-cymene)Cl_2_]_2_ (chiral diamine ligand-to-Ru molar ratio = 2:1) was charged into a 15 mL Schlenk tube under argon atmosphere. The reaction was vigorously stirred at 40 °C for 4 h. Upon completion, the resultant solid was collected by filtration and purified via Soxhlet extraction with DCM (24 h) to remove unreacted species. Subsequently, the product was dried under reduced pressure at 40 °C for 12 h to afford the target complex CCOFX-Ru. For comparative studies, analogous complexes CCOF2-Ru’, CCOF2-Rh, and CCOF2-Ir were synthesized by replacing [Ru(p-cymene)Cl_2_]_2_ with [Ru(η^6^-C_6_H_6_)Cl_2_]_2_, [Cp*RhCl_2_]_2_, and [Cp*IrCl_2_]_2_, respectively, following identical synthetic protocols.

### Experimental procedure for asymmetric catalysis

A representative catalytic asymmetric transfer hydrogenation was conducted as follows. A 10 mL Schlenk tube equipped with a magnetic stir bar was charged with: CCOF2-Ru catalyst (12.1 mg, 1.0 μmol Ru, 0.5 mol% based on ICP-OES analysis), Sodium formate (HCOONa, 68.0 mg, 1.0 mmol), Acetophenone derivative (0.20 mmol) and H₂O/i-PrOH co-solvent system (1:1 v/v, total 1.0 mL). The reaction system was subjected to three consecutive freeze-pump-thaw cycles under a nitrogen atmosphere to eliminate dissolved oxygen. The mixture was stirred in a thermostatically controlled oil bath at 60 °C for 1 h. After completion, the crude mixture was filtered through a 0.22 μm PTFE membrane. The yield and ee were determined by HPLC analysis.

### Gram-scale synthesis and cycling experiments

A 100 mL Schlenk tube equipped with a magnetic stir bar was charged with: CCOF2-Ru catalyst (0.5 mol% of Ru active site based on ICP analysis), Sodium formate (HCOONa, 27.1 mmol), 5,7-difluorochroman-4-one (1 g, 5.43 mmol) and H₂O/i-PrOH co-solvent system (1:1 v/v, total 27 mL). The reaction system was subjected to three consecutive freeze-pump-thaw cycles under a nitrogen atmosphere to eliminate dissolved oxygen. The mixture was stirred in a thermostatically controlled oil bath at 60 °C for 1 ~ 1.5 h until the substrate was completely converted (monitored by TLC). Upon completion of the reaction, the crude mixture was filtered, and the product was purified by silica gel column chromatography using a mixture of petroleum ether and ethyl acetate (PE/EA = 10:1) as the eluent. The ee was determined by HPLC analysis. The catalyst was recovered by washing thoroughly with water and methanol, followed by drying at room temperature, and was subsequently reused directly in further catalytic cycles.

## Supplementary information

Supplementary Information

Transparent Peer Review file

## Source data

Source Data

## Data Availability

All data generated or analyzed during this study are included in this published article and its supplementary information files. Source data are provided with this paper. All data are available from the corresponding author upon request. Source data are provided with this paper.
