# Supplementary material for: Multivariate approach of metal-chiral covalent organic frameworks for leaching-resistant asymmetric transfer hydrogenation
Source: Nat Commun. 2026 Sep 25;17:11147. doi: 10.1038/s41467-026-78066-y (PMC13710755; doi:10.1038/s41467-026-78066-y)
Supplement: Supplementary file 1 [file 41467_2026_78066_MOESM1_ESM.pdf]

Supplementary Information

Multivariate Approach of Metal-Chiral Covalent Organic Frameworks for Leaching-Resistant Asymmetric Transfer Hydrogenation

Zhu Yin,[1,2] Jun Lin,[1,2] Xinru Gong,[1,2] Yingqi Wang,[1,2] Beili Lu,[3] Xiong Chen,[1,2] Jiajia Cheng[1,2,*]

[1] State Key Laboratory of Chemistry for NBC Hazards Protection, College of Chemistry, Fuzhou University, Fuzhou 350116, China. [2] State Key Laboratory of Photocatalysis on Energy and Environment, College of Chemistry, Fuzhou University, Fuzhou 350116, China. [3] College of Material Engineering, Fujian Agriculture and Forestry University, Fuzhou 350108, China.

*Corresponding author E-mail: jjcheng@fzu.edu.cn

Table of Contents

Section S1 General methods **S3**

Section S2 Synthetic procedures **S5**

Section S3 Supplementary Figures **S34**

Section S4 Supplementary Tables **S55**

Section S5 HPLC of catalytic products **S87**

REFERENCES **S120**

Section 1. General methods

Chemicals

1,3,5-Tris(4-formylphenyl)benzene (TFPB), 4,4''-diamino-*p*-terphenyl (DATP), 2,5-dibromobenzenesulfonyl chloride, (1*R*,2*R*)-1,2-diphenylethane-1,2-diamine, (±)-1,2-diphenylethane-1,2-diamine, (1*R*,2*R*)-cyclohexane-1,2-diamine, (4,4,5,5-tetramethyl-1,3,2-dioxaborolan-2-yl)benzene, trifluoroacetic acid (TFA), 4-(4,4,5,5-tetramethyl-1,3,2-dioxaborolan-2-yl)aniline, di-tert-butyl dicarbonate (Boc_2O), trimethylsilyl trifluoromethanesulfonate (TMSOTf), $[Ru(p\text{-cymene})Cl_2]_2$, $[Ru(\eta^6\text{-}C_6H_6)Cl_2]_2$, $[Cp^*RhCl_2]_2$, $[Cp^*IrCl_2]_2$, RuCl(p-cymene)[(*R,R*)-Ts-DPEN], tetrakis(triphenylphosphine)palladium(0) $[Pd(PPh_3)_4]$, *n*-butanol, ethyl acetate, petroleum ether, dichloromethane, sodium formate, ammonium formate, acetophenone, and other ketone derivatives were purchased from Energy Chemical and used as received. 1,4-Dioxane, potassium bromide, anhydrous ethanol, tetrahydrofuran (THF), acetic acid, potassium carbonate (K_2CO_3), triethylamine, isopropanol, sodium hydroxide, potassium hydroxide, and anhydrous sodium sulfate were obtained from Sinopharm Chemical Reagent Co., Ltd. All other commercial reagents were used as received unless otherwise noted.

Characterization

The IR (KBr pellet) spectra were recorded on a Nicolet Magna 750 FT-IR spectrometer. Thermogravimetric analyses (TGA) were carried out in an N_2 atmosphere with a heating rate of 10 °C/min on a STA449C integration thermal analyzer. Scanning electron microscopy (SEM) images were obtained on a Hitachi SU8000 and a Thermo Scientific Verios G4 scanning electron microscope. Powder X-ray diffraction (PXRD) patterns were collected on a Bruker D8 Advance diffractometer using Cu Kα radiation in a low-angle configuration over a 2θ range of 1.5–30°, at

a scan rate of 10° min^{-1}, with the slit width set to 0.1 mm. Transmission Electron Microscopy (TEM) images were obtained on a JEM-2100 instrument. Raman spectra were measured using an InVia Reflex 1900099S spectrometer. Elemental analysis of the COFs was performed on an Elementar Vario EL Cube analyzer. The molecular masses of the compounds were determined by LC-MS (AB Sciex ZenoTOF 7600). XPS spectra were obtained using an X-ray photoelectron spectrometer (Thermo Scientific ESCALAB QXi). Liquid ^{1}H NMR, ^{13}C NMR, DEPT NMR and ^{19}F NMR spectra were conducted on Bruker Advance III 400 MHz NMR spectrometer. Solid-state ^{13}C NMR experiments were carried out on AVANCE III HD 500 NMR spectrometers operating at resonance frequencies of 500 MHz. Analytical high performance liquid chromatography (HPLC) was performed on a Waters 1525 (Waters) with UV detection. N_2 adsorption isotherms of COFs were measured using a Micrometritics ASAP 2020 system at 77 K with liquid N_2 bath. The structural models of the COF were constructed using the Materials Studio software package. The unit cell parameters were optimized using the Forcite module with molecular mechanics calculations employing the Universal force field. Rietveld refinements were performed with the Reflex module implemented in Materials Studio. The theoretical BET surface areas and pore volumes were calculated directly from the simulated structures using Zeo++.[1] Ru K-edge analysis was performed with Si(311) crystal monochromators at the BL13ssw beamlines at the Shanghai Synchrotron Radiation Facility (SSRF) (Shanghai, China). Before the analysis at the beamline, samples were pressed into thin sheets with 1 cm in diameter and sealed using Kapton tape film. The XAFS spectra were recorded at room temperature using a 4-channel Silicon Drift Detector (SDD) Bruker 5040. Ru K-edge extended X-ray absorption fine structure (EXAFS) spectra were recorded in transmission mode. Negligible changes in the line-shape and peak position of Ru *K*-edge XANES spectra were observed between two scans taken for a specific sample. The XAFS

spectra of these standard samples (Ru foil, $RuCl_3$, and RuO_2) were recorded in transmission mode. The XAFS data were processed according to the standard procedures using the Athena module implemented in the IFEFFIT software packages. The EXAFS spectra were obtained by subtracting the post-edge background from the overall absorption and then normalizing with respect to the edge-jump step. Subsequently, the $\chi(k)$ data of were Fourier transformed to real (R) space using a hanning windows (dk = 1.0 $Å^{-1}$) to separate the EXAFS contributions from different coordination shells. To obtain the quantitative structural parameters around central atoms, least-squares curve parameter fitting was performed using the ARTEMIS module of IFEFFIT software packages.[2, 3] For Wavelet Transform analysis, the $\chi(k)$ exported from Athena was imported into the Hama Fortran code.[4] The parameters were listed as follow: R-range, 0.0-4.0 Å, k-range, 0-16.0 $Å^{-1}$ for sample and Standards; k weight, 2; and Morlet function with κ=8, σ=1 was used as the mother wavelet to provide the overall distribution.

Section 2. Synthetic procedures

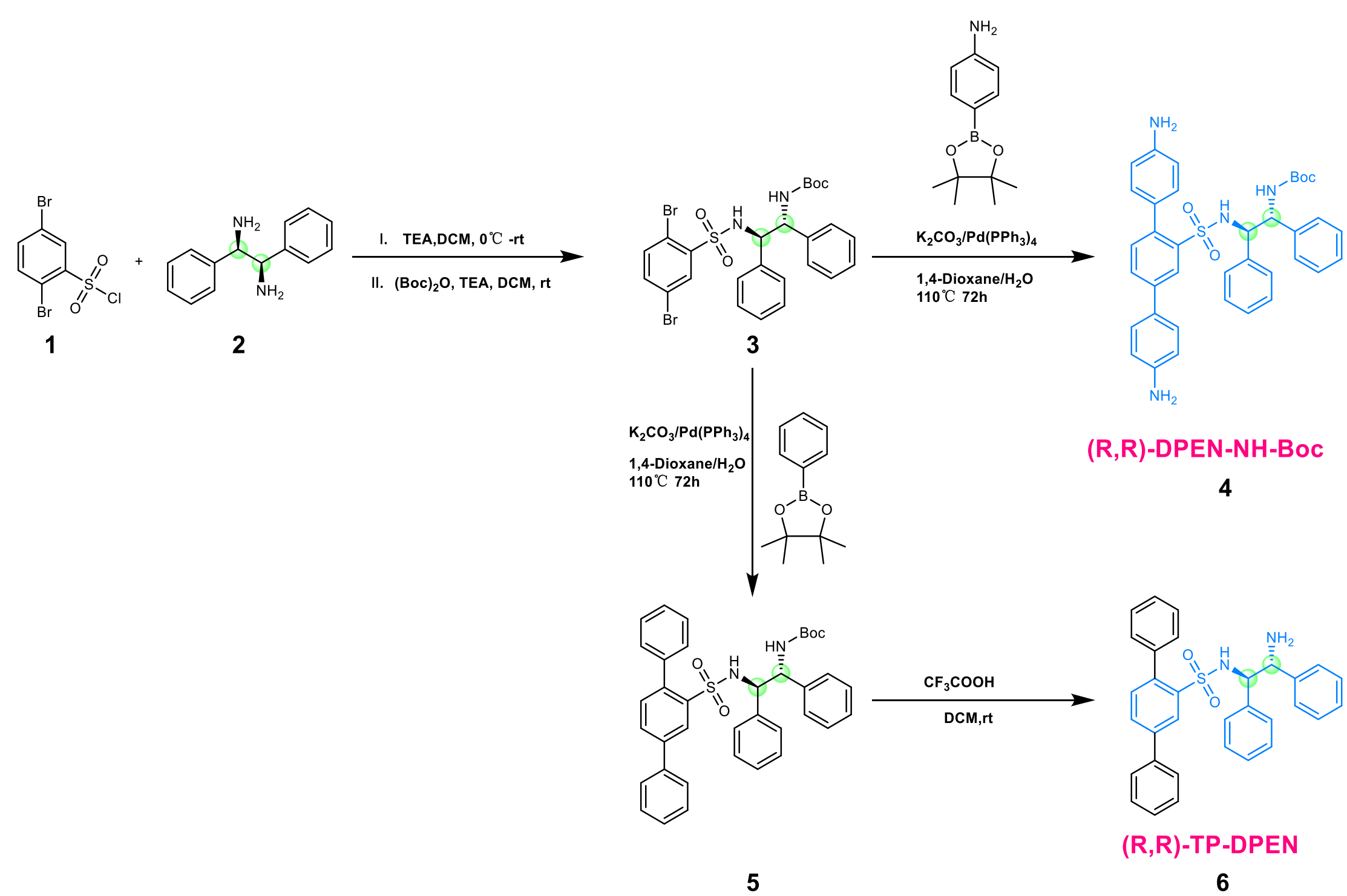

2.1. Synthesis of compound 3 (tert-butyl ((1R,2R)-2-((2,5-dibromophenyl) sulfonamido)-1,2-diphenylethyl) carbamate):

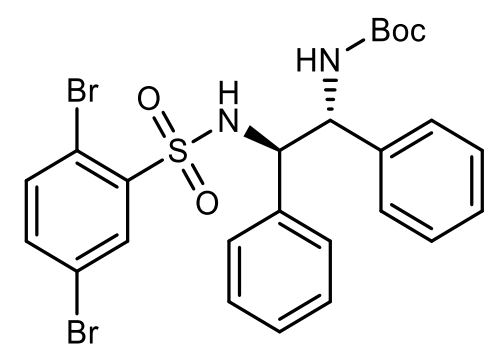

(1R,2R)-1,2-diphenylethane-1,2-diamine (**2**, 5.0 g, 14.9 mmol) was dissolved in 60 mL of dichloromethane (DCM), and triethylamine (4.0 mL, 28.7 mmol) was added dropwise with stirring at 0 °C. A solution of 2,5-dibromobenzenesulfonyl chloride (1, 5.0 g, 14.9 mmol) in 20 mL of DCM was added slowly to the mixture at 0 °C. After stirring at 0 °C for 30 min, the reaction mixture was warmed to room temperature and stirred for 4 h. Then, the mixture was cooled back to 0 °C, and an additional 4.0 mL of triethylamine was added, followed by slow addition of di--butyl dicarbonate (Boc_2O, 4.0 mL, 17.3 mmol). The reaction was then warmed to room temperature and stirred overnight. The organic solvent was removed under reduced pressure, and the residue was acidified to pH = 2 with 1 M aqueous potassium bisulfate ($KHSO_4$) and extracted with DCM. The combined organic layers were washed with saturated aqueous brine, dried over anhydrous Na_2SO_4, and concentrated under vacuum.

Purification was performed via column chromatography on silica gel (200–300 mesh) using petroleum ether / ethyl acetate (PE/EA, 4:1, v/v) as the eluent. The product was dried under vacuum at 60 °C for 12 h to afford 7.8 g of white solid (**3**) in 86% yield. ^{1}H NMR (600 MHz, DMSO-D_6) δ 8.58 – 8.46 (d, J = 8.7 Hz, 1H), 7.67 – 7.64 (d, J = 2.4 Hz, 1H), 7.49 – 7.39 (m, 3H), 7.25 – 7.19 (dd, J = 7.4, 5.0 Hz, 4H), 7.14 – 7.08 (t, J = 7.5 Hz, 4H), 7.07 – 7.02 (q, J = 7.0 Hz, 2H), 5.02 – 4.85 (dd, J = 9.7, 6.3 Hz, 1H), 4.82 – 4.67 (t, J = 7.3 Hz, 1H), 1.33 – 1.24 (s, 9H); ^{13}C NMR (151 MHz, DMSO-D_6) δ 28.7, 59.0, 63.0, 78.6, 118.6, 120.9, 127.3, 127.4, 127.5, 128.1, 128.2, 132.9, 136.3, 137.3, 139.4, 140.5, 141.8, 155.6; DEPT 135° NMR (151 MHz, DMSO-D_6)

δ 28.7, 59.0, 63.0, 127.3, 127.4, 127.5, 128.1, 128.2, 132.9, 136.3, 137.3. ESI-HRMS: Found: m/z 630.9889. Calcd for $C_{25}H_{26}Br_2N_2O_4S$: $(M+Na)^+$ 630.9872.

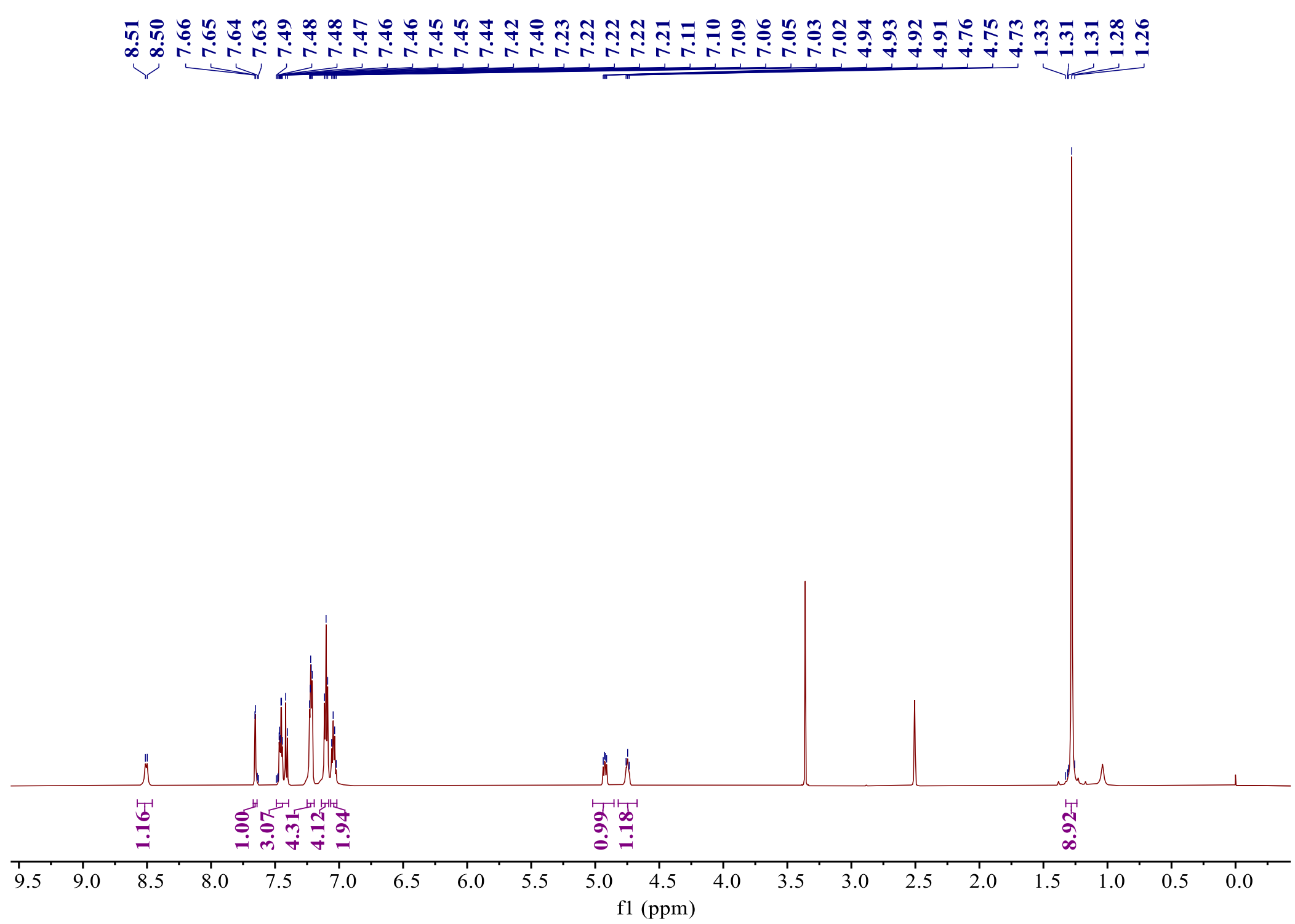

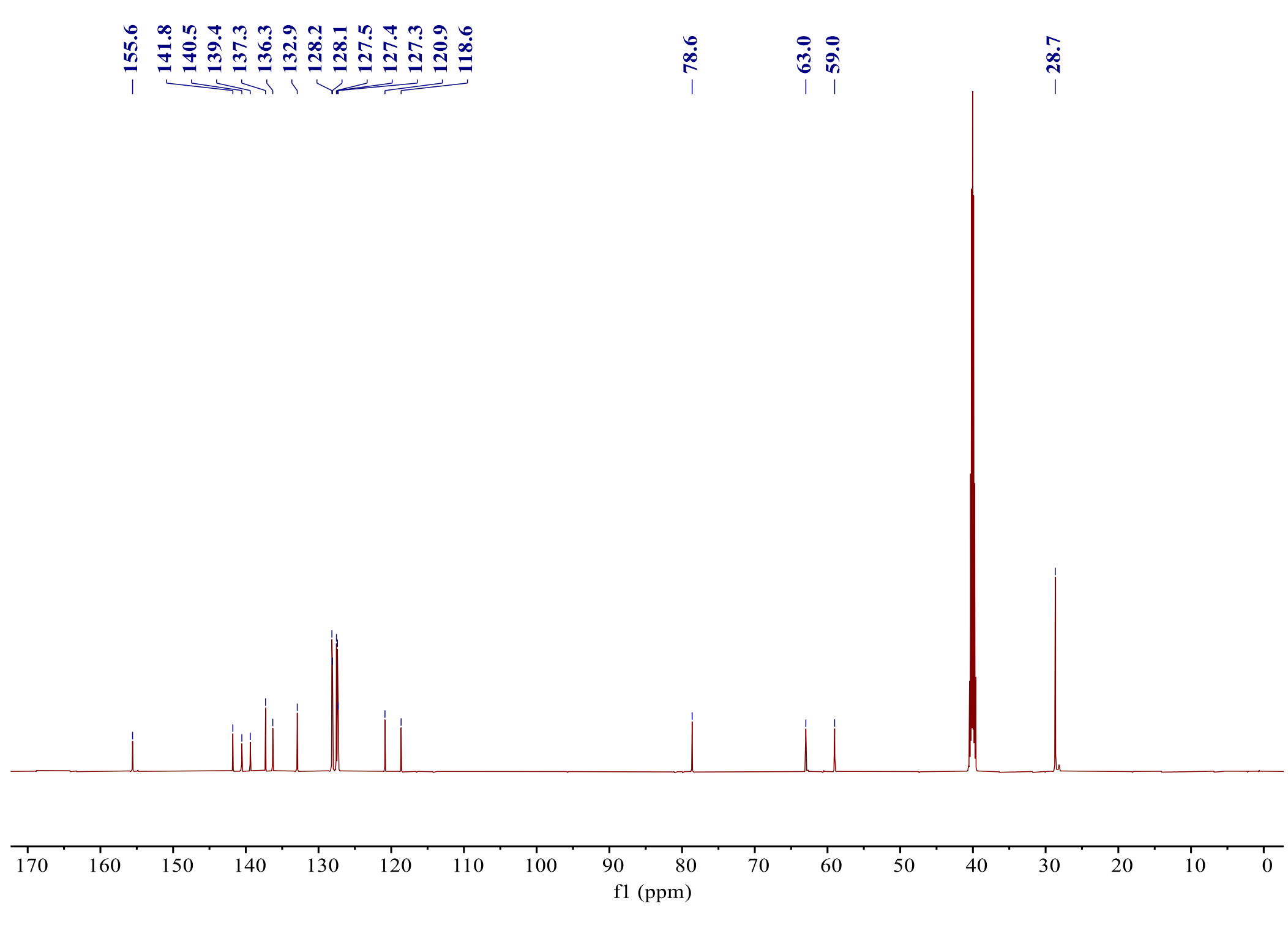

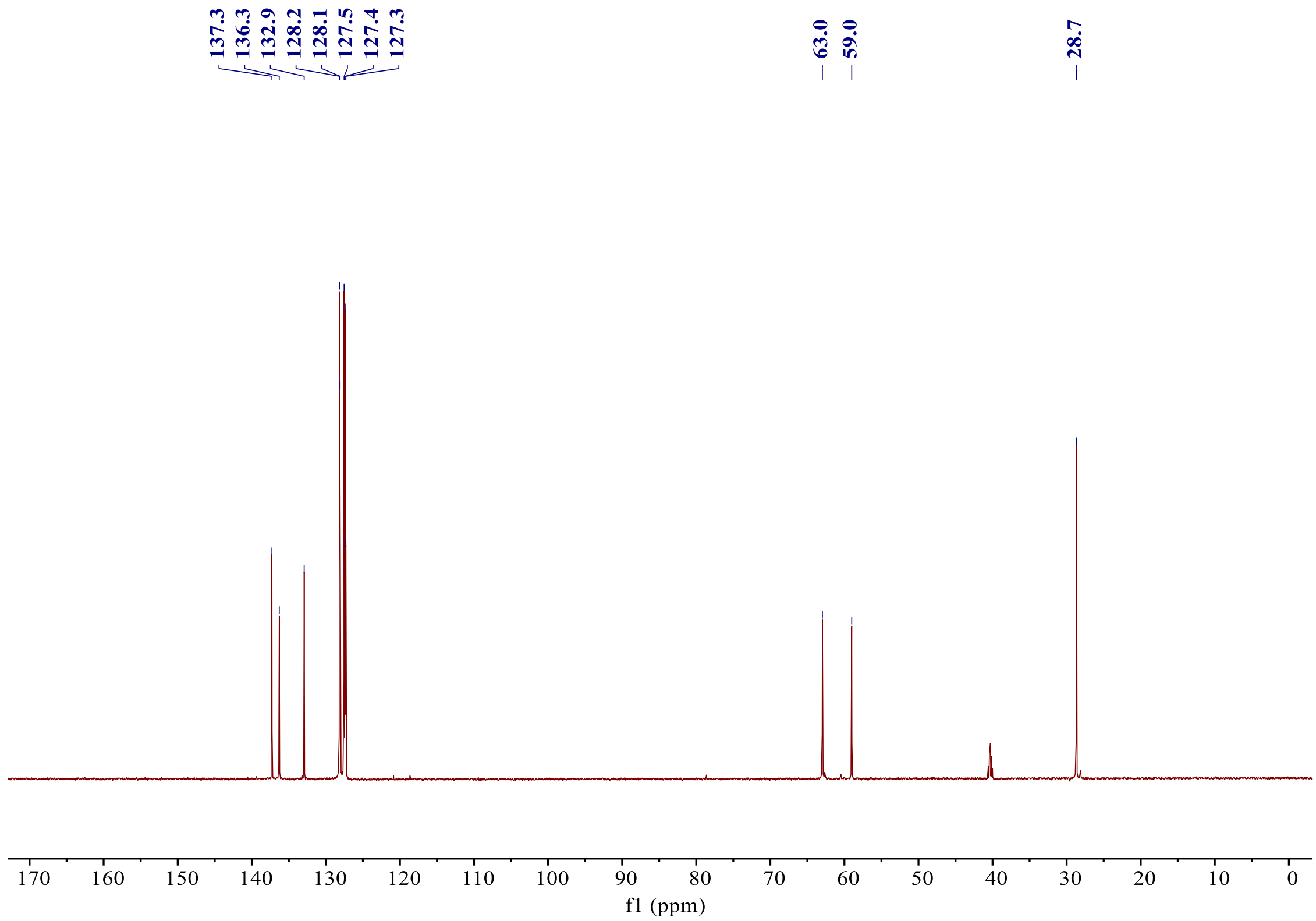

The ee-value of compound **3** was determined by HPLC analysis with a 25 cm × 4.6 mm Daicel Chiralcel IC column (hexane/i-PrOH = 90/10, 1.0 mL/min). t_{minor} = 11.1 min, t_{major} = 7.5 min; ee = 99.2 %. The racemic sample used in the HPLC analysis was synthesized by replacing (1*R*,2*R*)-1,2-diphenylethane-1,2-diamine with (±)-1,2-diphenylethane-1,2-diamine under the identical synthetic procedure.

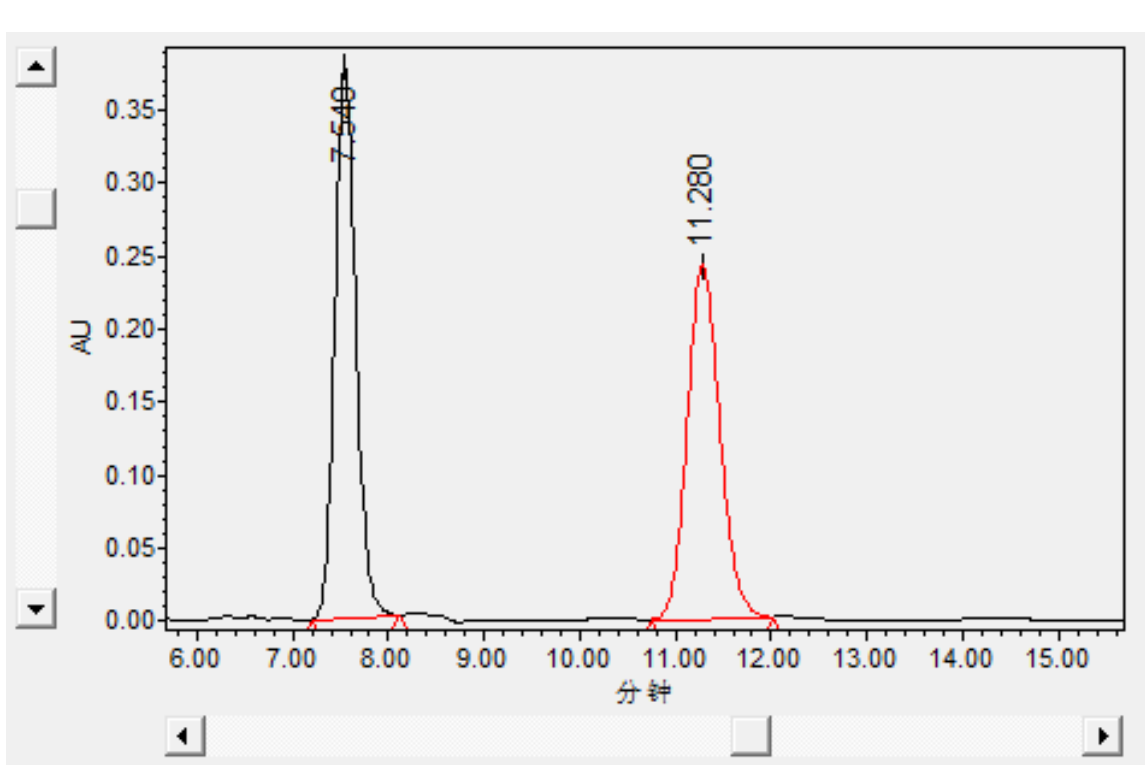

	名称	保留时间 (分钟)	面积 (微伏*秒)	% 面积	高度 (微伏)	积分类型	含量	单位	峰类型	峰代码
1		7.540	5788048	50.34	377355	bb			未知	
2		11.280	5709955	49.66	241982	bb			未知	

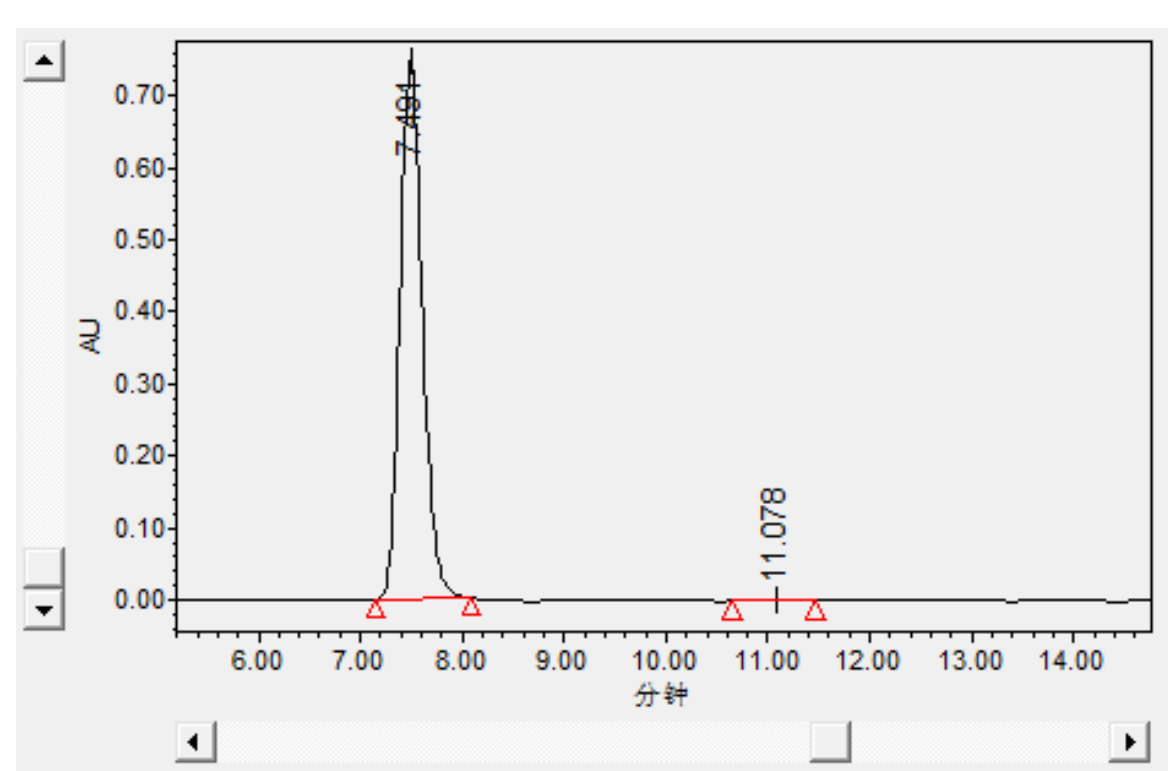

	名称	保留时间 (分钟)	面积 (微伏*秒)	% 面积	高度 (微伏)	积分类型	含量	单位	峰类型	峰代码
1		7.491	11528807	99.63	749239	bb			未知	
2		11.078	42485	0.37	1941	bb			未知	

Peaks number	Retention time (min)	Area	Area percentage (%)	Height of peaks
1	7.491	11528807	99.63	749239
2	11.078	42485	0.37	1941

2.2. Synthesis of (*R,R*)-DPEN-NH-Boc (4) (tert-butyl ((1R,2R)-2-((4,4''-diamino-[1,1':4',1''-terphenyl])-2'-sulfonamido)-1,2-diphenylethyl) carbamate):

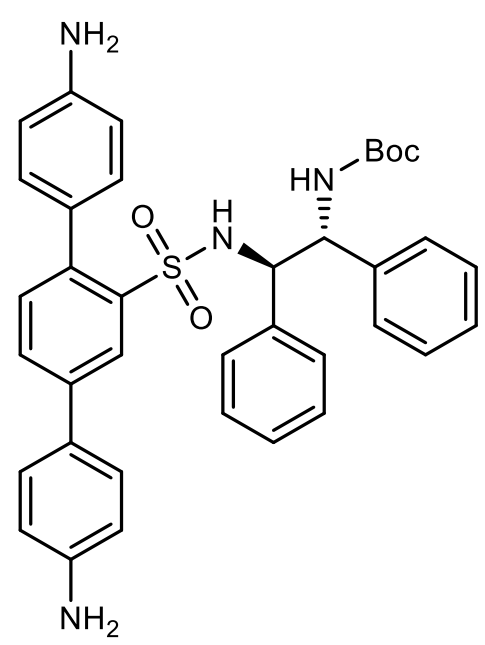

Compound **3** (5.0 g, 8.2 mmol), 4-(4,4,5,5-tetramethyl-1,3,2-dioxaborolan-2-yl)aniline (5.4 g, 24.6 mmol), potassium carbonate (K_2CO_3, 4.53g, 32.8 mmol), and tetrakis(triphenylphosphine)palladium(0) ($Pd(PPh_3)_4$, 0.47 g, 0.41 mmol) were charged into a 250 mL three-necked flask. The reaction system was degassed and backfilled with nitrogen three times to maintain an inert atmosphere, followed by addition of a pre-degassed mixed solvent of 1,4-dioxane/water (4:1, v/v, 170 mL). The mixture was heated to 110 °C and stirred at this temperature for 72 h. After cooling to room temperature, insoluble residues were removed by filtration, and the organic solvent was evaporated under reduced pressure.

The crude residue was extracted with dichloromethane (DCM), and the combined organic phase was washed with saturated aqueous brine, dried over anhydrous sodium sulfate (Na_2SO_4), and concentrated under vacuum. Purification was carried out by column chromatography on silica gel with DCM/methanol (25:1, v/v) as the eluent. The resulting product was dried under vacuum at

60 °C for 24 h to afford 4.63 g of pale yellow powder, identified as (*R,R*)-DPEN-NH-Boc (**4**), in 89% yield with 99.1% ee. ^{1}H NMR (600 MHz, DMSO-D_6) δ 8.02 – 7.91 (d, *J* = 9.6 Hz, 1H), 7.72 – 7.65 (d, *J* = 2.0 Hz, 1H), 7.55 – 7.42 (m, 1H), 7.20 – 7.14 (d, *J* = 9.1 Hz, 1H), 7.13 – 7.10 (dd, *J* = 7.2, 4.9 Hz, 4H), 7.10 – 7.03 (dt, *J* = 14.6, 6.8 Hz, 7H), 7.03 – 7.01 (d, *J* = 7.0 Hz, 1H), 7.01 – 6.99 (d, *J* = 7.8 Hz, 1H), 6.93 – 6.87 (d, *J* = 8.1 Hz, 2H), 6.63 – 6.59 (d, *J* = 8.0 Hz, 2H), 6.57 – 6.53 (d, *J* = 8.3 Hz, 2H), 5.39 – 5.10 (d, 4H), 4.85 – 4.79 (t, *J* = 8.3 Hz, 1H), 4.71 – 4.53 (t, *J* = 8.5 Hz, 1H), 1.34 – 1.26 (s, 9H); ^{13}C NMR (151 MHz, DMSO-D_6) δ 28.7, 60.0, 62.5, 78.7, 113.4, 114.7, 125.1, 126.1, 127.2, 127.4, 127.5, 127.6, 127.7, 127.9, 128.2, 128.2, 128.3, 130.6, 133.7, 138.4, 139.2, 139.6, 140.4, 140.8, 148.3, 149.3, 155.5; DEPT 135° NMR (151 MHz, DMSO-D_6) δ 28.7, 60.0, 62.5, 113.4, 114.7, 125.1, 127.2, 127.4, 127.6, 127.7, 127.9, 128.2, 128.2, 128.3, 130.6, 133.7. ESI-HRMS: Found: m/z 657.2537. Calcd for $C_{25}H_{26}Br_2N_2O_4S$: $(M+Na)^+$ 657.2506.

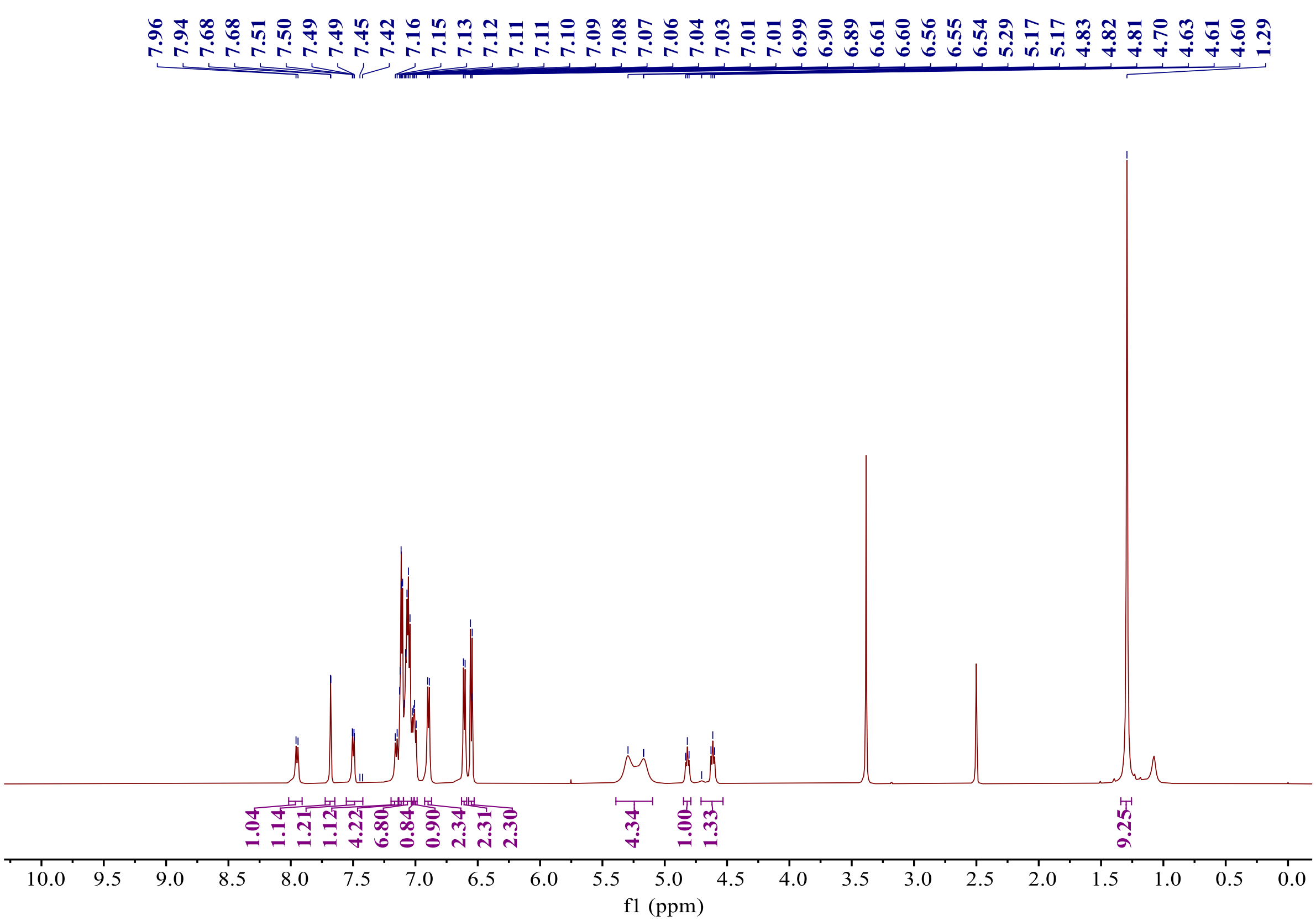

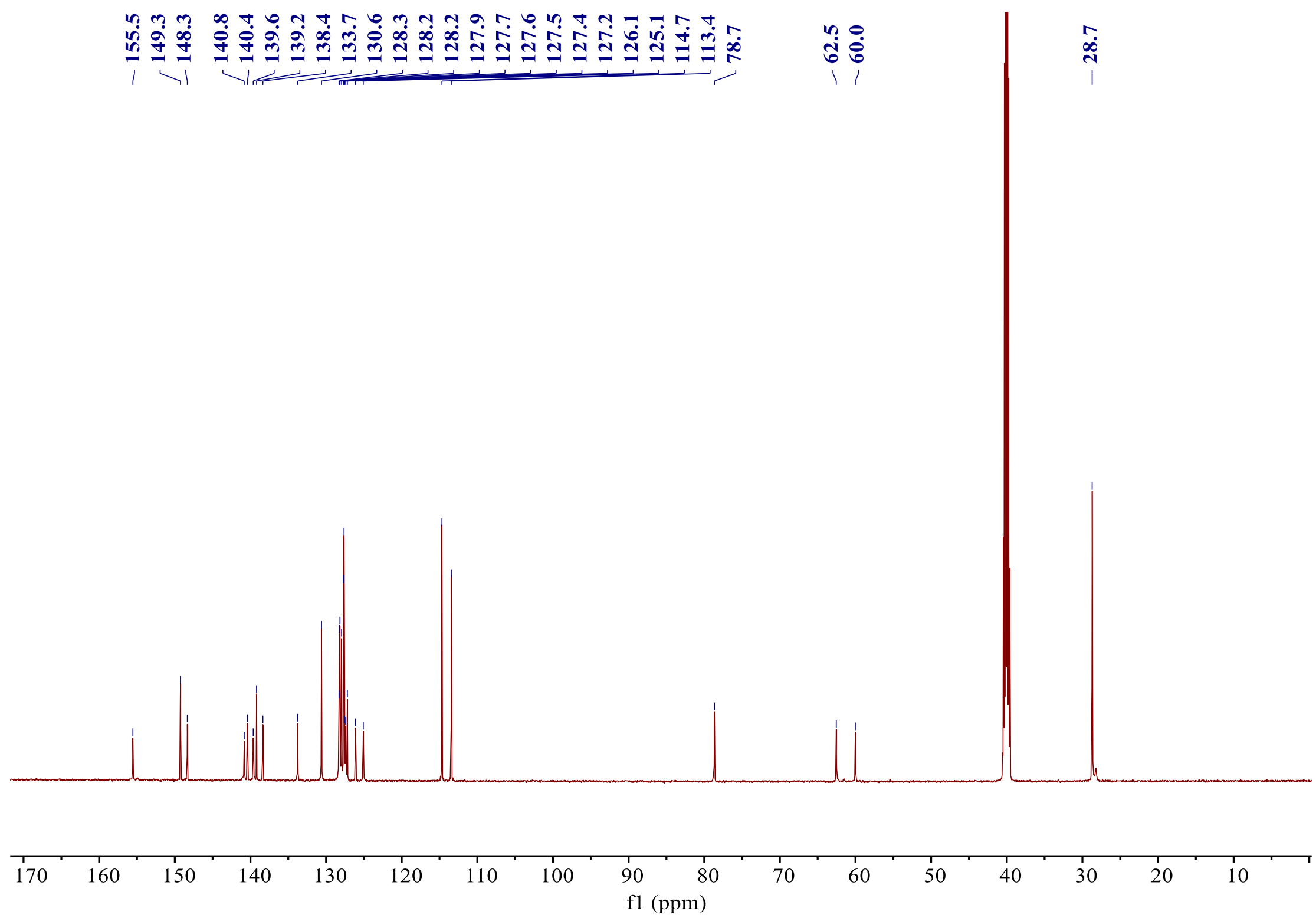
155.5
149.3
148.3
140.8
140.4
139.6
139.2
138.4
133.7
130.6
128.3
128.2
128.2
127.9
127.7
127.6
127.5
127.4
127.2
126.1
125.1
114.7
113.4
78.7
62.5
60.0
28.7
f1 (ppm)

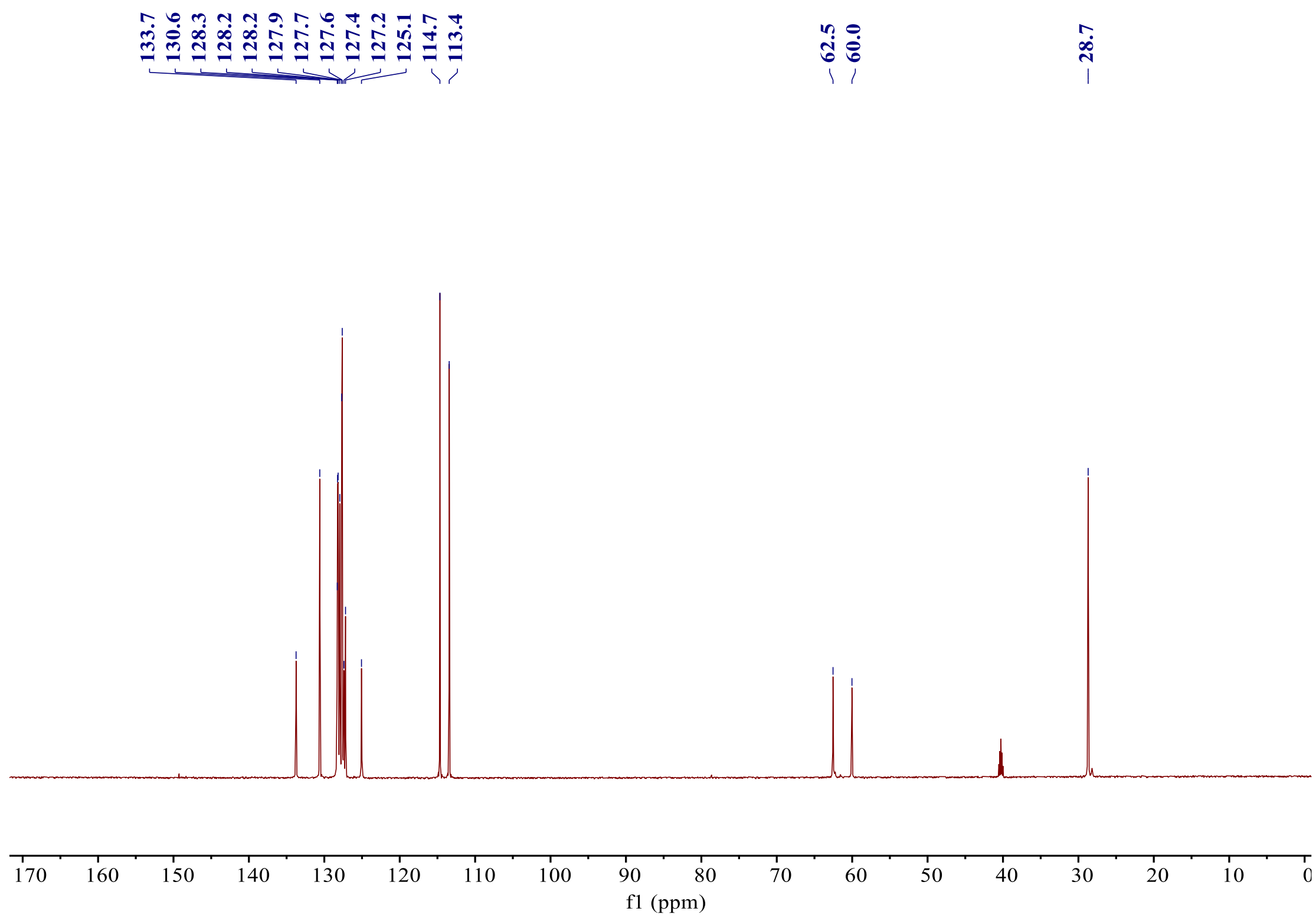
133.7
130.6
128.3
128.2
128.2
127.9
127.7
127.6
127.4
127.2
125.1
114.7
113.4
62.5
60.0
28.7
f1 (ppm)

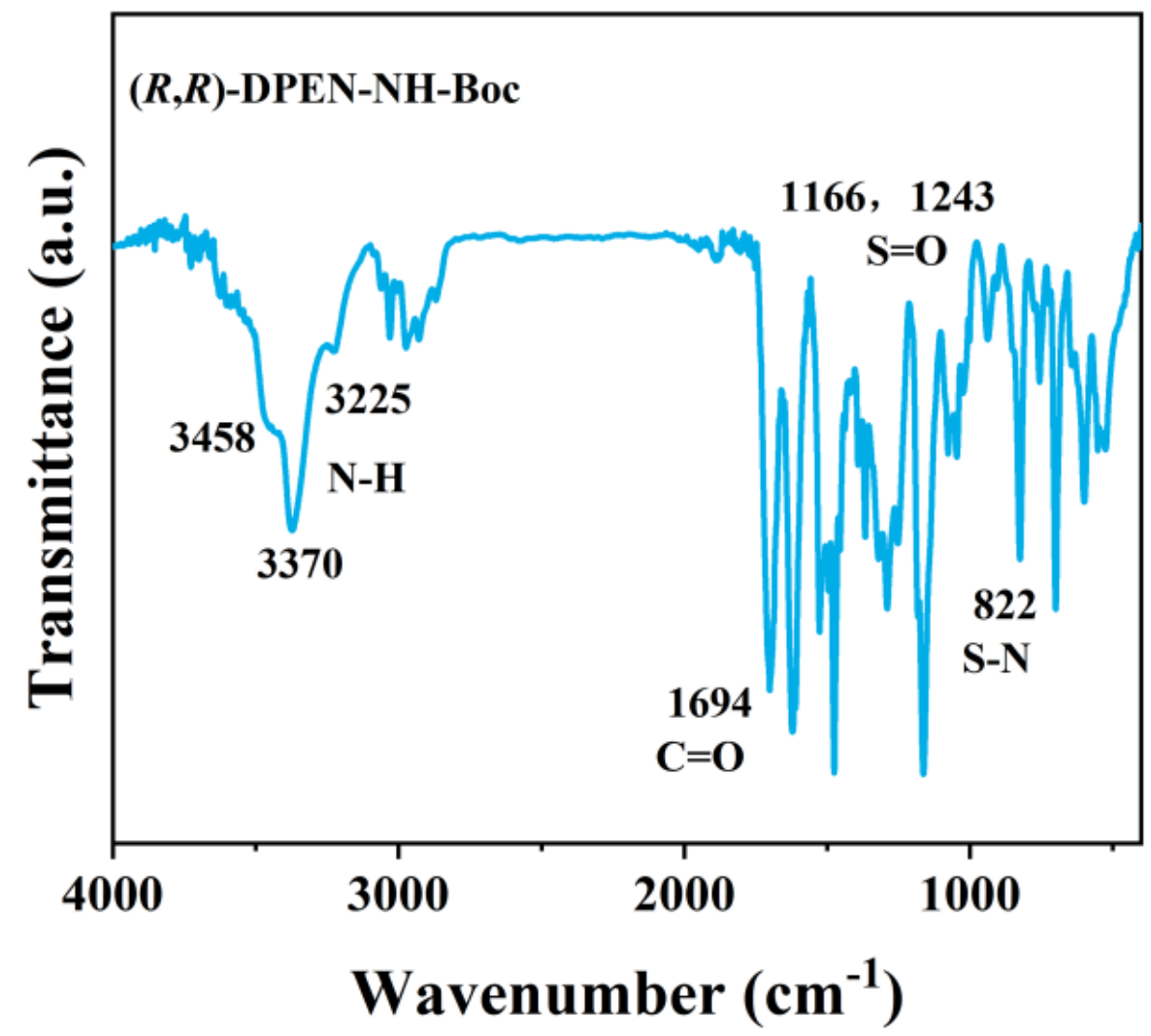

The ee-value of **(*R*,*R*)-DPEN-NH-Boc** was determined by HPLC analysis with a 25 cm × 4.6 mm Daicel Chiralcel IE column (hexane/i-PrOH = 60/40, 1.0 mL/min). t_{minor} = 33.7 min, t_{major} = 25.3 min; ee = 99.1%. The racemic reference was prepared following the same synthetic procedure, starting from racemic compound 3, which was derived from (±)-1,2-diphenylethane-1,2-diamine instead of the (1R,2R) enantiomer.

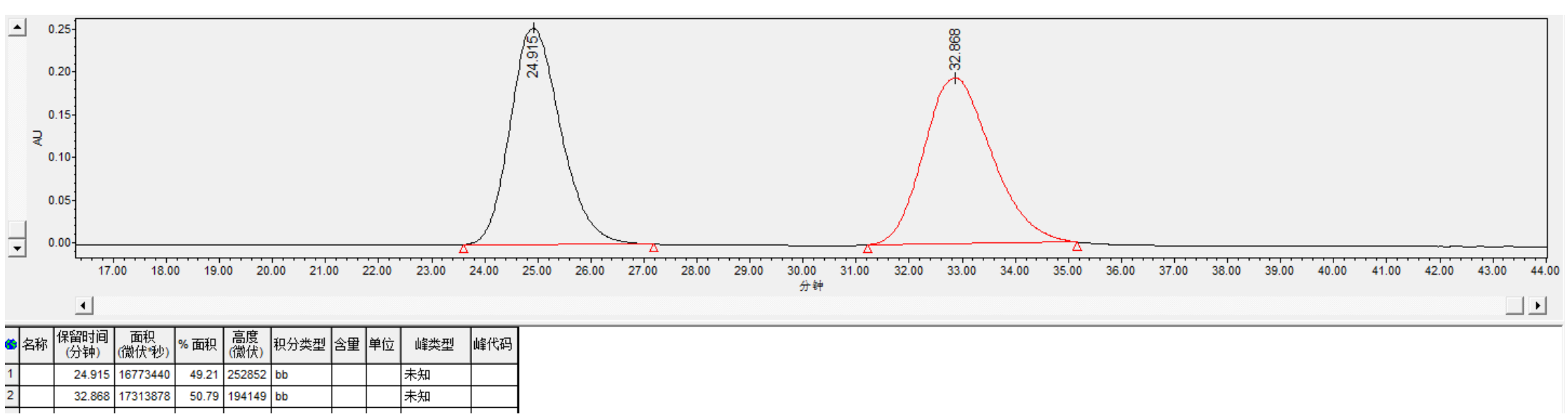

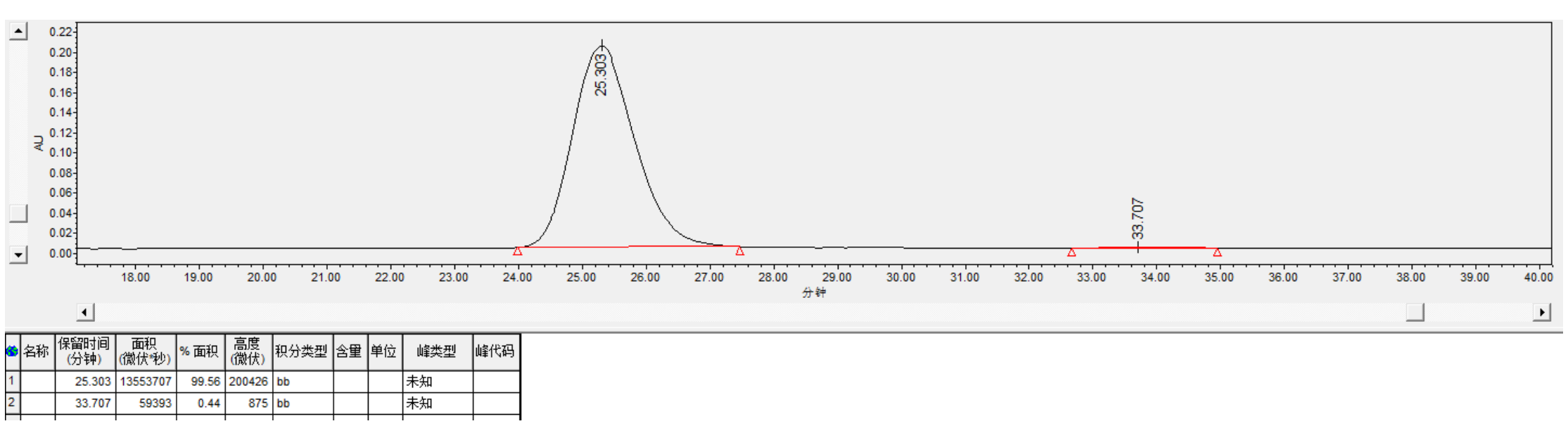

Peaks number	Retention time (min)	Area	Area percentage (%)	Height of peaks

1	25.303	13553707	99.56	200426
2	33.707	59393	0.44	875

2.3. Synthesis of compound 5 (tert-butyl((1R,2R)-2-([1,1':4',1''-terphenyl]-2'-sulfonamido) -1,2-diphenylethyl) carbamate):

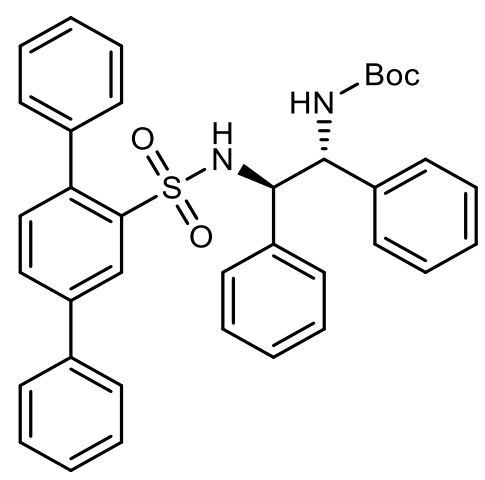

Compound **3** (1.26 g, 2.06 mmol), (4,4,5,5-tetramethyl-1,3,2-dioxaborolan-2-yl)benzene (6.18 mmol, 1.17 g), potassium carbonate (K_2CO_3, 8.24 mmol, 1.14 g), and tetrakis(triphenylphosphine)palladium(0) ($Pd(PPh_3)_4$, 0.1 mmol, 0.116 g) were charged into a 100 mL three-necked flask. The reaction system was degassed and backfilled with nitrogen three times to establish an inert atmosphere, followed by the addition of 50 mL of a pre-degassed 1,4-dioxane/water mixture (4:1, v/v,). The mixture was heated to 110 °C and stirred at this temperature for 72 h. After cooling to ambient temperature, insoluble residues were removed by filtration, and the organic solvent was evaporated under reduced pressure.

The crude product was extracted with dichloromethane (DCM), and the combined organic layer was washed with saturated aqueous brine, dried over anhydrous Na_2SO_4, and concentrated in vacuo. Purification was performed via column chromatography on silica gel using petroleum ether / ethyl acetate (PE / EA, 4:1, v/v) as the eluent. The target product was dried under vacuum at 60 °C for 24 h to afford 1.06 g of white solid (compound **5**) in 85% yield. ^{1}H NMR (600 MHz, CHLOROFORM-*D*) δ 8.17 – 8.10 (s, 1H), 7.72 – 7.63 (m, 1H), 7.55 – 7.50 (d, *J* = 7.6 Hz, 2H), 7.49 – 7.44 (t, *J* = 7.5 Hz, 2H), 7.43 – 7.37 (m, 2H), 7.36 – 7.29 (t, *J* = 7.7 Hz, 4H), 7.21 – 7.17 (d,

J = 7.8 Hz, 1H), 7.13 – 7.05 (m, 4H), 7.04 – 6.98 (t, J = 7.5 Hz, 2H), 6.95 – 6.89 (m, 2H), 5.50 – 5.42 (d, J = 8.1 Hz, 1H), 5.01 – 4.94 (d, J = 7.6 Hz, 1H), 4.85 – 4.77 (t, J = 9.1 Hz, 1H), 4.43 – 4.35 (t, J = 8.9 Hz, 1H), 1.46 – 1.35 (s, 9H); ^{13}C NMR (151 MHz, CHLOROFORM-*D*) δ 28.4, 59.4, 63.7, 80.3, 127.2, 127.3, 127.5, 127.7, 127.9, 128.1, 128.1, 128.2, 128.4, 128.5, 129.0, 129.8, 130.1, 133.1, 137.4, 138.4, 138.7, 139.0, 139.4, 139.7, 140.3, 156.4; DEPT 135° NMR (151 MHz, CHLOROFORM-*D*) δ 28.4, 59.4, 63.7, 127.2, 127.3, 127.5, 127.7, 127.9, 128.1, 128.2, 128.2, 128.4, 128.5, 129.1, 129.8, 130.2, 133.1; ESI-HRMS: Found: m/z 627.2310. Calcd for $C_{37}H_{36}N_2O_4S$: $(M+Na)^+$ 627.2288.

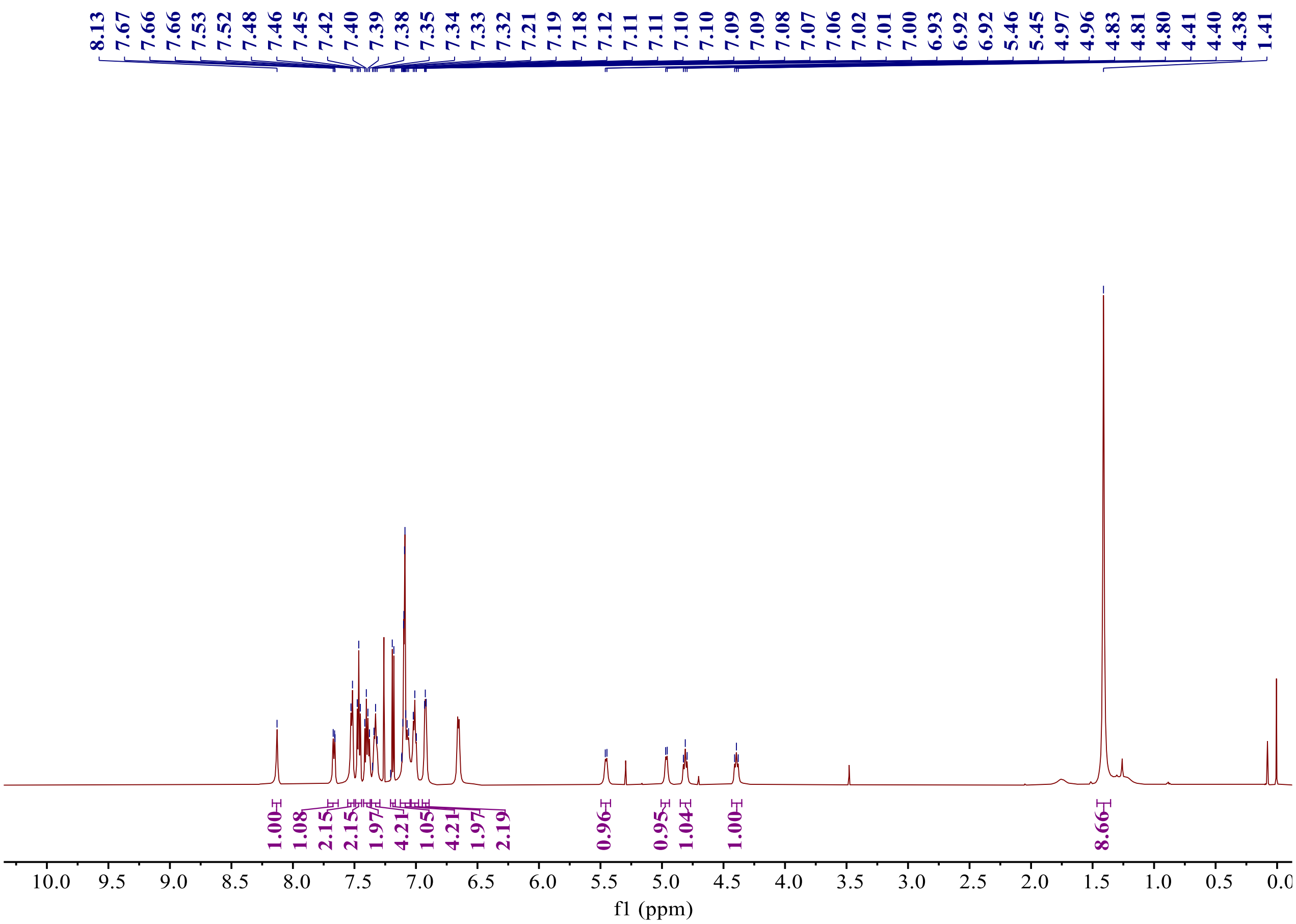

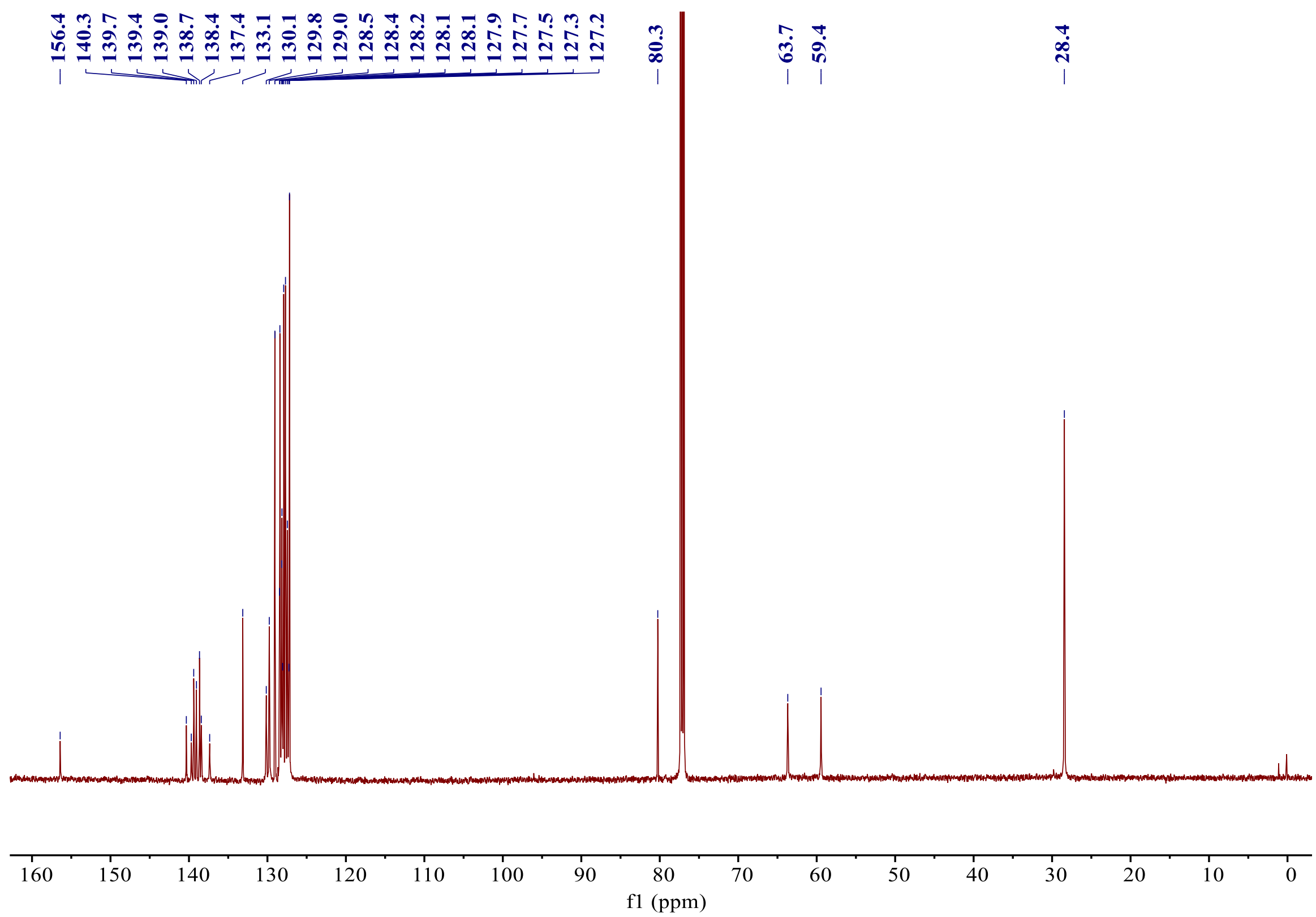
156.4
140.3
139.7
139.4
139.0
138.7
138.4
137.4
133.1
130.1
129.8
129.0
128.5
128.4
128.2
128.1
128.1
127.9
127.7
127.5
127.3
127.2
80.3
63.7
59.4
28.4
160 150 140 130 120 110 100 90 80 70 60 50 40 30 20 10 0
f1 (ppm)

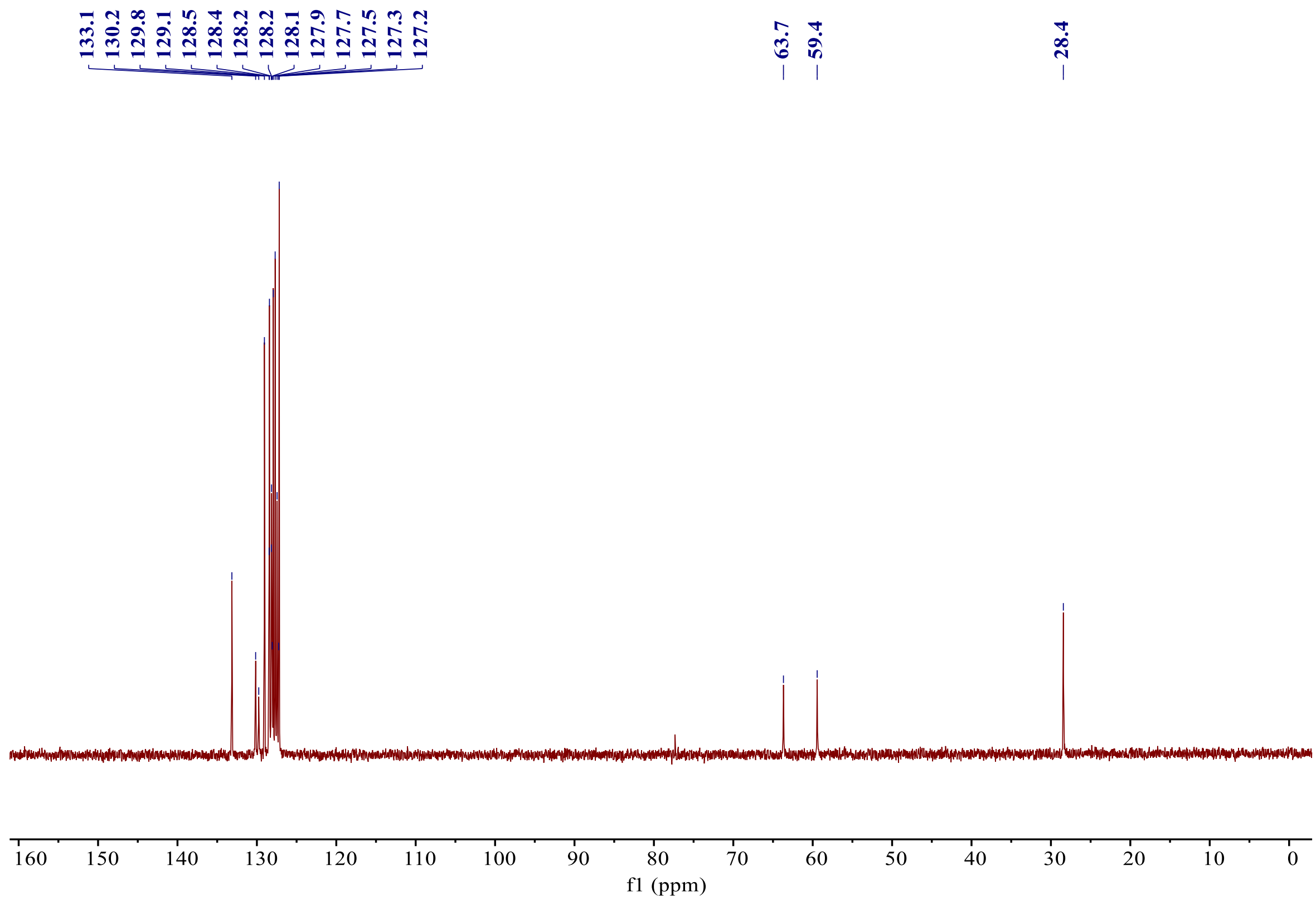
133.1
130.2
129.8
129.1
128.5
128.4
128.2
128.2
128.1
127.9
127.7
127.5
127.3
127.2
63.7
59.4
28.4
160 150 140 130 120 110 100 90 80 70 60 50 40 30 20 10 0
f1 (ppm)

The ee-value of **compound 5** was determined by HPLC analysis with a 25 cm × 4.6 mm Daicel Chiralcel IA column (hexane/i-PrOH = 90/10, 1.0 mL/min). t_{minor} = 15.3 min, t_{major} = 8.2 min; ee = 99.4%. The racemic reference was prepared following the same synthetic procedure, starting from racemic compound 3, which was derived from (±)-1,2-diphenylethane-1,2-diamine instead of the (1R,2R) enantiomer.

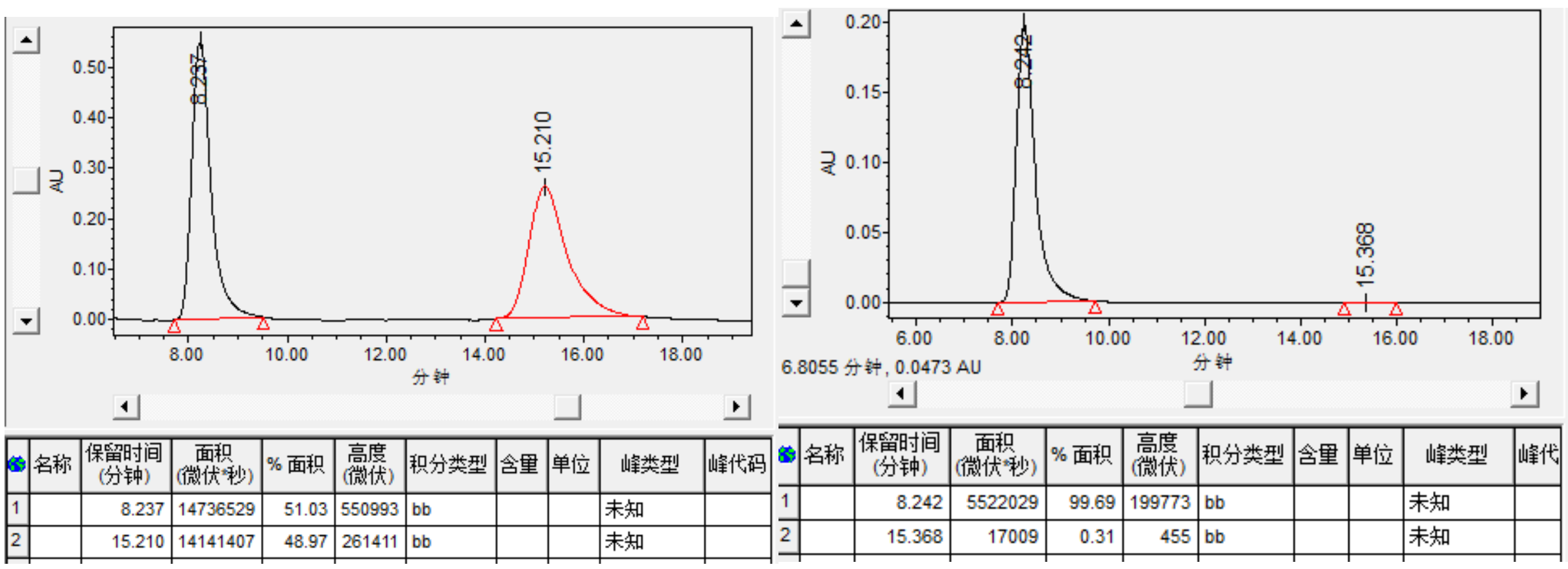

Peaks number	Retention time (min)	Area	Area percentage (%)	Height of peaks
1	8.242	5522029	99.69	199773
2	15.368	17009	0.31	455

2.4. Synthesis of (*R*,*R*)-TP-DPEN (6) (N-((1R,2R)-2-amino-1,2-diphenylethyl)-[1,1':4',1''-terphenyl]-2'-sulfonamide):

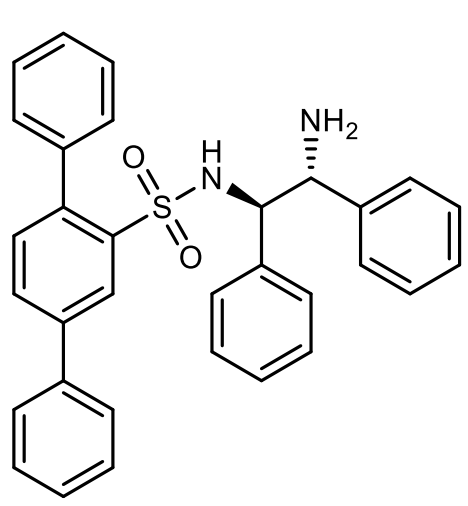

Compound **5** (500 mg) was dissolved in 3 mL of anhydrous dichloromethane (DCM). Trifluoroacetic acid (TFA, 1 mL) was added to the solution, and the mixture was stirred at room

temperature for 2 h. Excess TFA was neutralized with saturated aqueous sodium bicarbonate solution. The mixture was extracted with DCM, and the combined organic phase was washed with saturated aqueous brine and dried over anhydrous Na_2SO_4. After removal of the solvent under vacuum, purification was performed via column chromatography on silica gel using DCM/methanol (10:1, v/v) as the eluent. The residue was further dried under vacuum at 60 °C for 24 h to afford 392 mg of white powder (*R,R*)-TP-DPEN (6) in 94% yield. ^{1}H NMR (600 MHz, DMSO-*D*$_6$) δ 7.91 – 7.88 (d, *J* = 2.0 Hz, 1H), 7.74 – 7.71 (dd, *J* = 7.9, 2.0 Hz, 1H), 7.47 – 7.43 (ddd, *J* = 8.8, 6.4, 0.9 Hz, 2H), 7.42 – 7.36 (m, 6H), 7.34 – 7.30 (m, 2H), 7.22 – 7.20 (d, *J* = 7.9 Hz, 1H), 7.08 – 6.96 (m, 10H), 4.39 – 4.31 (d, *J* = 8.2 Hz, 1H), 4.03 – 3.96 (d, *J* = 8.2 Hz, 1H). ^{13}C NMR (151 MHz, DMSO-*D*$_6$) δ 61.3, 65.4, 126.5, 127.2, 127.3, 127.8, 128.0, 128.1, 128.5, 129.5, 129.8, 129.8, 133.7, 138.7, 139.5, 139.7, 140.0, 140.1, 141.0, 143.1; DEPT 135° NMR (151 MHz, DMSO-*D*$_6$) δ 61.3, 65.4, 126.5, 127.2, 127.3, 127.8, 128.0, 128.1, 128.5, 129.5, 129.8, 129.8, 133.7; ESI-HRMS: Found: m/z 527.1783. Calcd for $C_{32}H_{28}N_2O_2S$: $(M+Na)^+$ 527.1764.

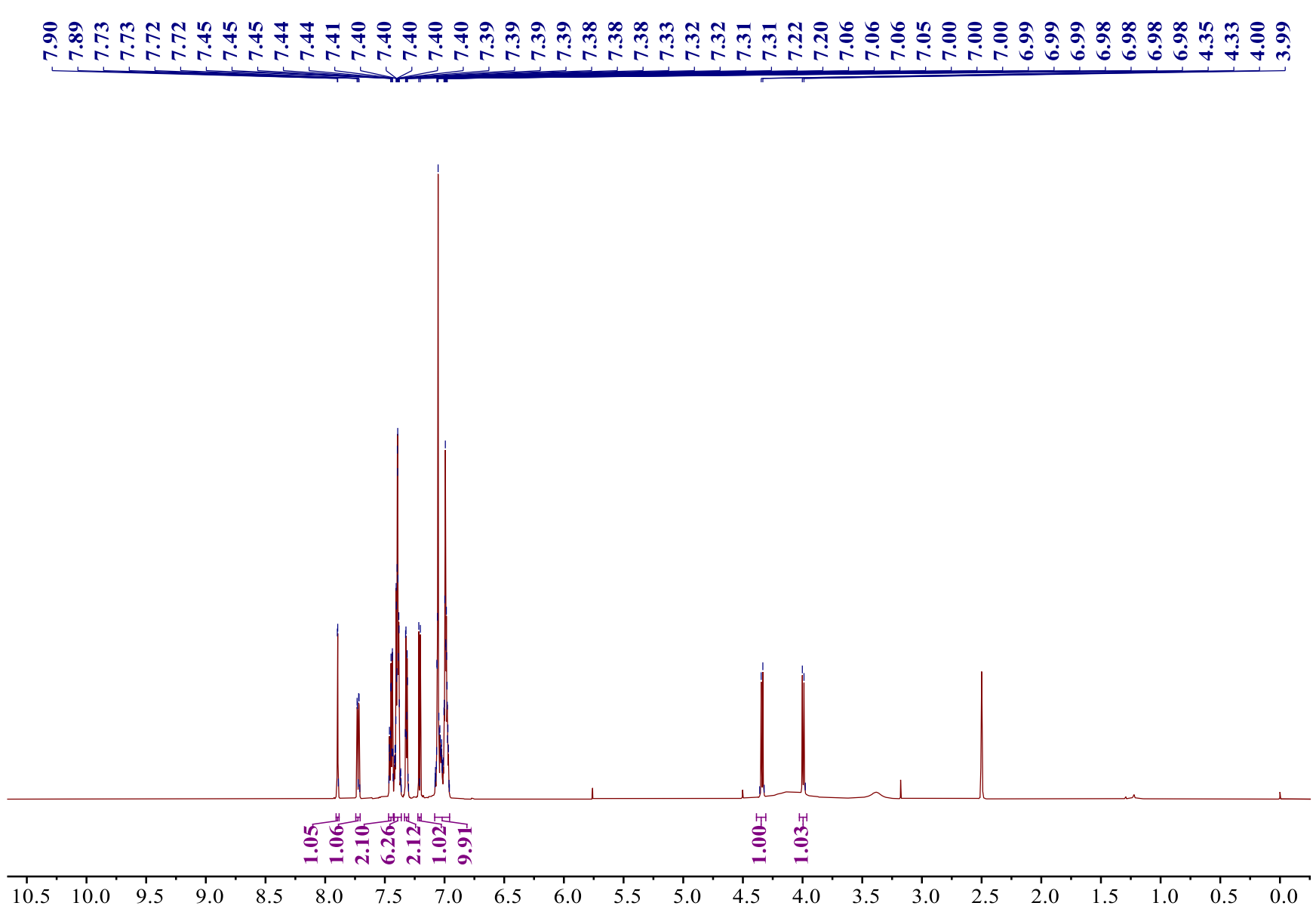

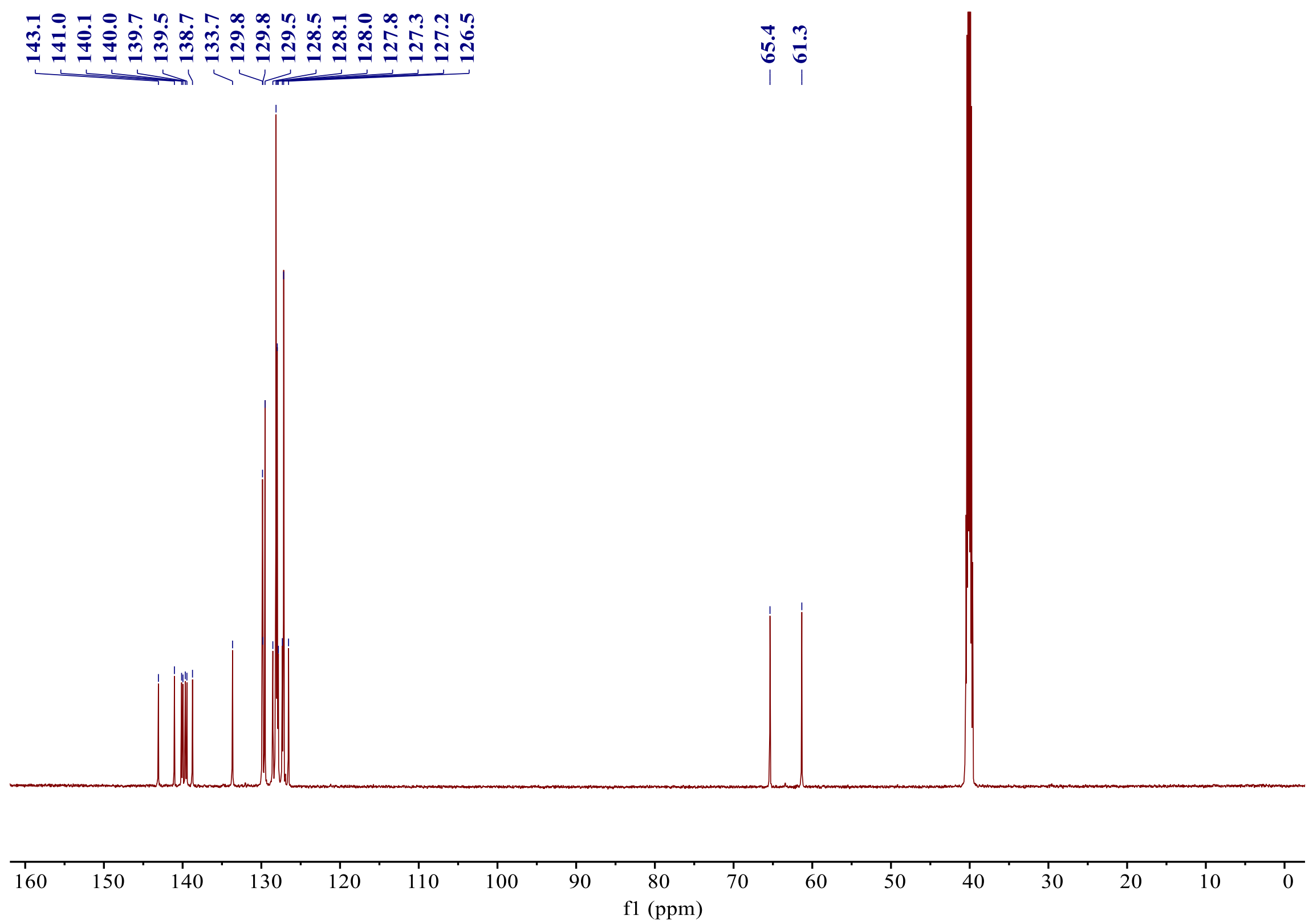
143.1
141.0
140.1
140.0
139.7
139.5
138.7
133.7
129.8
129.8
129.5
128.5
128.1
128.0
127.8
127.3
127.2
126.5
65.4
61.3
160 150 140 130 120 110 100 90 80 70 60 50 40 30 20 10 0
f1 (ppm)

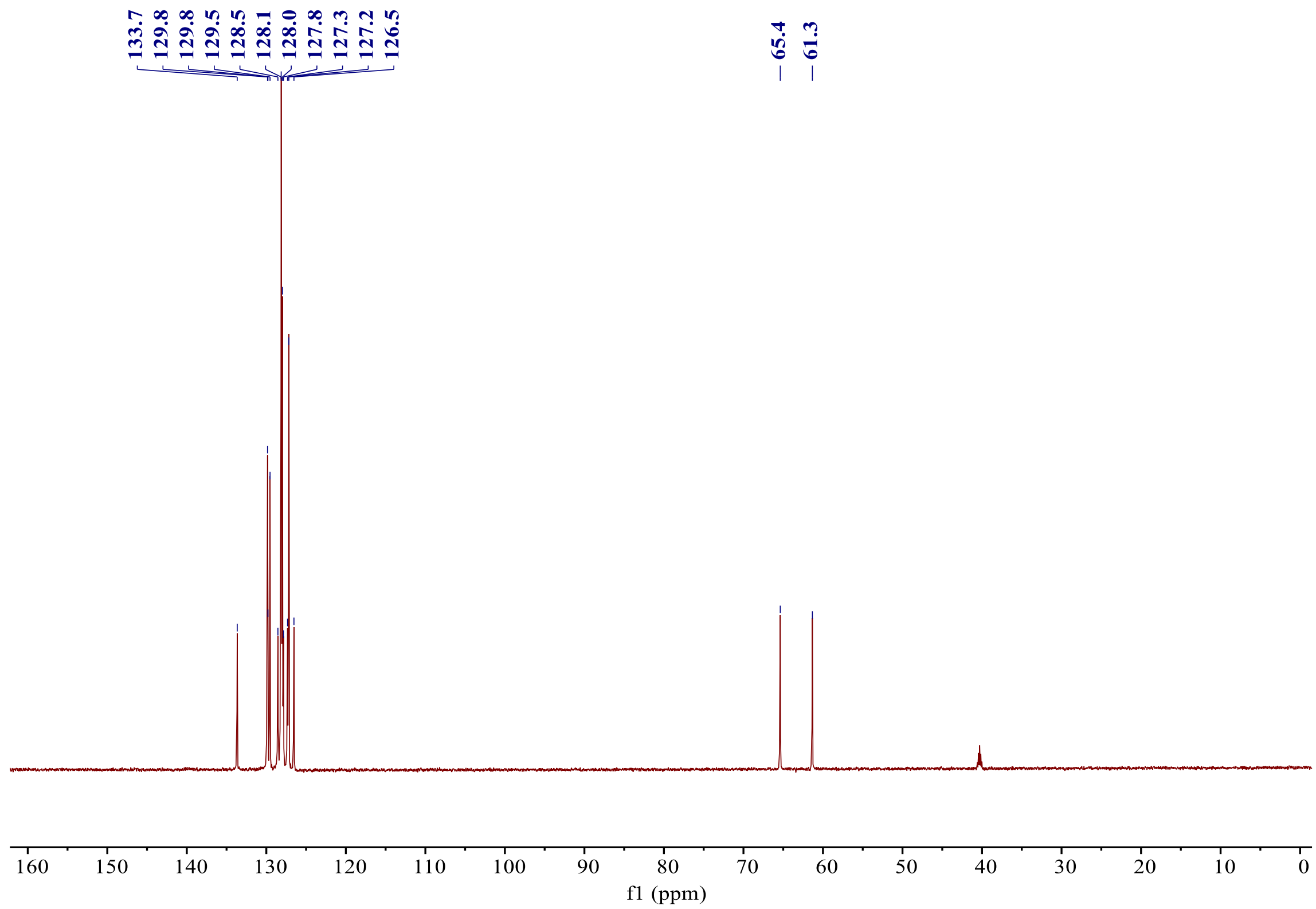
133.7
129.8
129.8
129.5
128.5
128.1
128.0
127.8
127.3
127.2
126.5
65.4
61.3
160 150 140 130 120 110 100 90 80 70 60 50 40 30 20 10 0
f1 (ppm)

The ee-value of **(*R*,*R*)-TP-DPEN (6)** was determined by HPLC analysis with a 25 cm × 4.6 mm Daicel Chiralcel IE column (hexane/i-PrOH = 70/30, 1.0 mL/min). t_{minor} = 15.3 min, t_{major} = 8.2 min; ee = 99.9%. The racemic reference was prepared following the same synthetic procedure, starting from racemic compound 5, which was derived from (±)-1,2-diphenylethane-1,2-diamine instead of the (1R,2R) enantiomer.

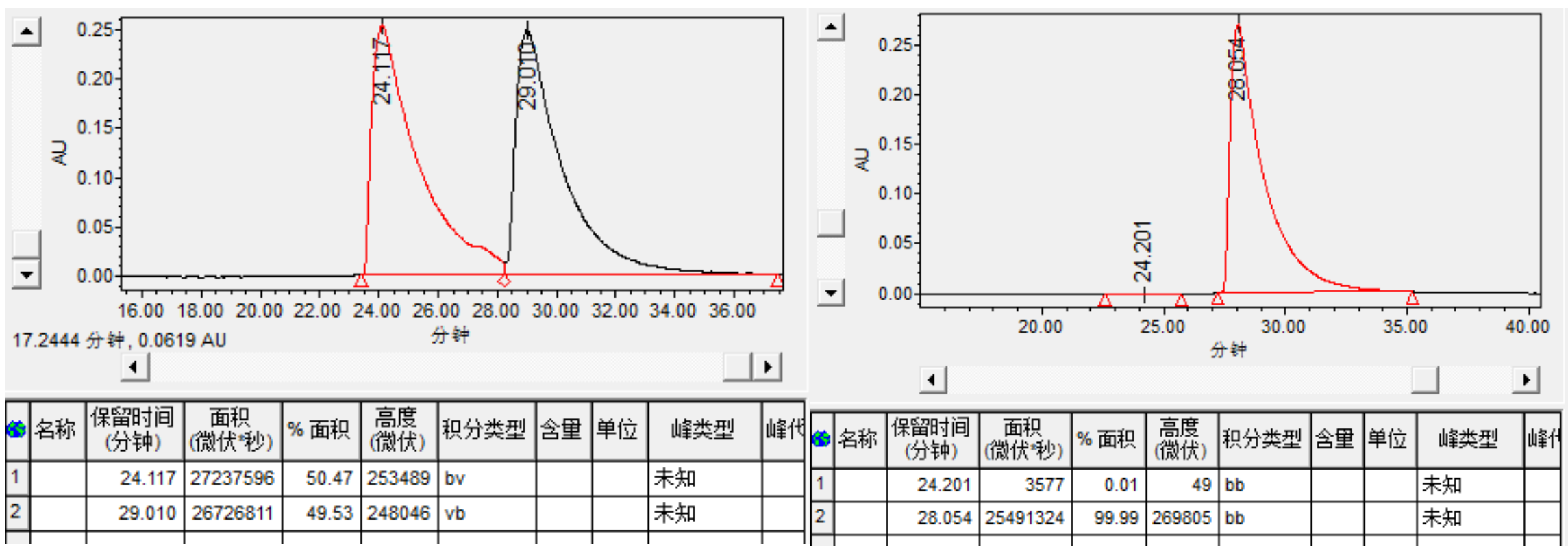

Peaks number	Retention time (min)	Area	Area percentage (%)	Height of peaks
1	24.201	3577	0.01	49
2	28.054	25491327	99.99	259805

2.5. Synthesis of (*R*,*R*)-TP-DPEN-Ru:

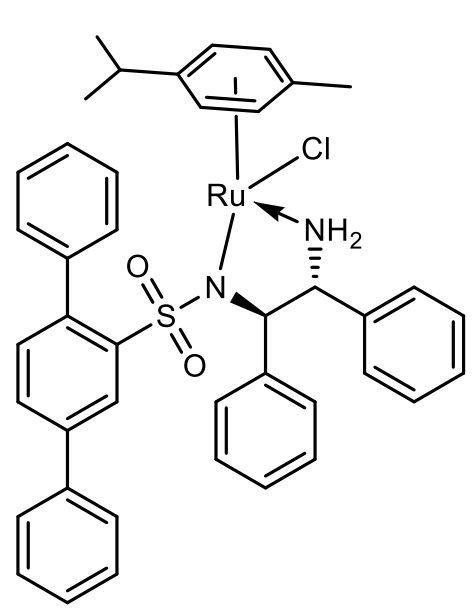

A mixture of [Ru(p-cymene)Cl_2]$_2$ (153 mg, 0.25 mmol), (*R*,*R*)-TP-DPEN (504 mg, 1 mmol), and NEt_3 (0.14 mL, 1 mmol) in CH_2Cl_2 (10 mL) was stirred at room temperature for 1 h under a N_2 atmosphere. The mixture was then washed with water and dried over anhydrous Na_2SO_4.

Removal of the solvent under reduced pressure afforded the crude product. Purification by flash column chromatography on silica gel using dichloromethane/methanol (100:1, v/v) as the eluent gave the pure product as an orange-yellow powder (264 mg, 85% yield). ^{1}H NMR (600 MHz, ACETONITRILE-*D*$_3$) δ 8.04 – 7.94 (s, 1H), 7.66 – 7.45 (m, 6H), 7.44 – 7.31 (m, 6H), 7.10 – 7.04 (m, 3H), 6.90 – 6.84 (d, *J* = 7.3 Hz, 3H), 6.67 – 6.57 (s, 5H), 6.01 – 5.92 (s, 1H), 5.57 – 5.52 (s, 1H), 5.17 – 5.09 (s, 1H), 5.02 – 4.95 (s, 1H), 3.90 – 3.81 (d, *J* = 11.2 Hz, 1H), 3.75 – 3.68 (t, *J* = 12.6 Hz, 1H), 3.45 – 3.34 (t, *J* = 11.8 Hz, 1H), 3.01 – 2.93 (s, 1H), 2.27 – 2.23 (s, 3H), 1.34 – 1.27 (dd, *J* = 20.0, 7.0 Hz, 6H). ESI-HRMS: Found: m/z 739.1912. Calcd for $C_{42}H_{41}N_2O_2RuS$: $(M-Cl)^+$ 739.1927.

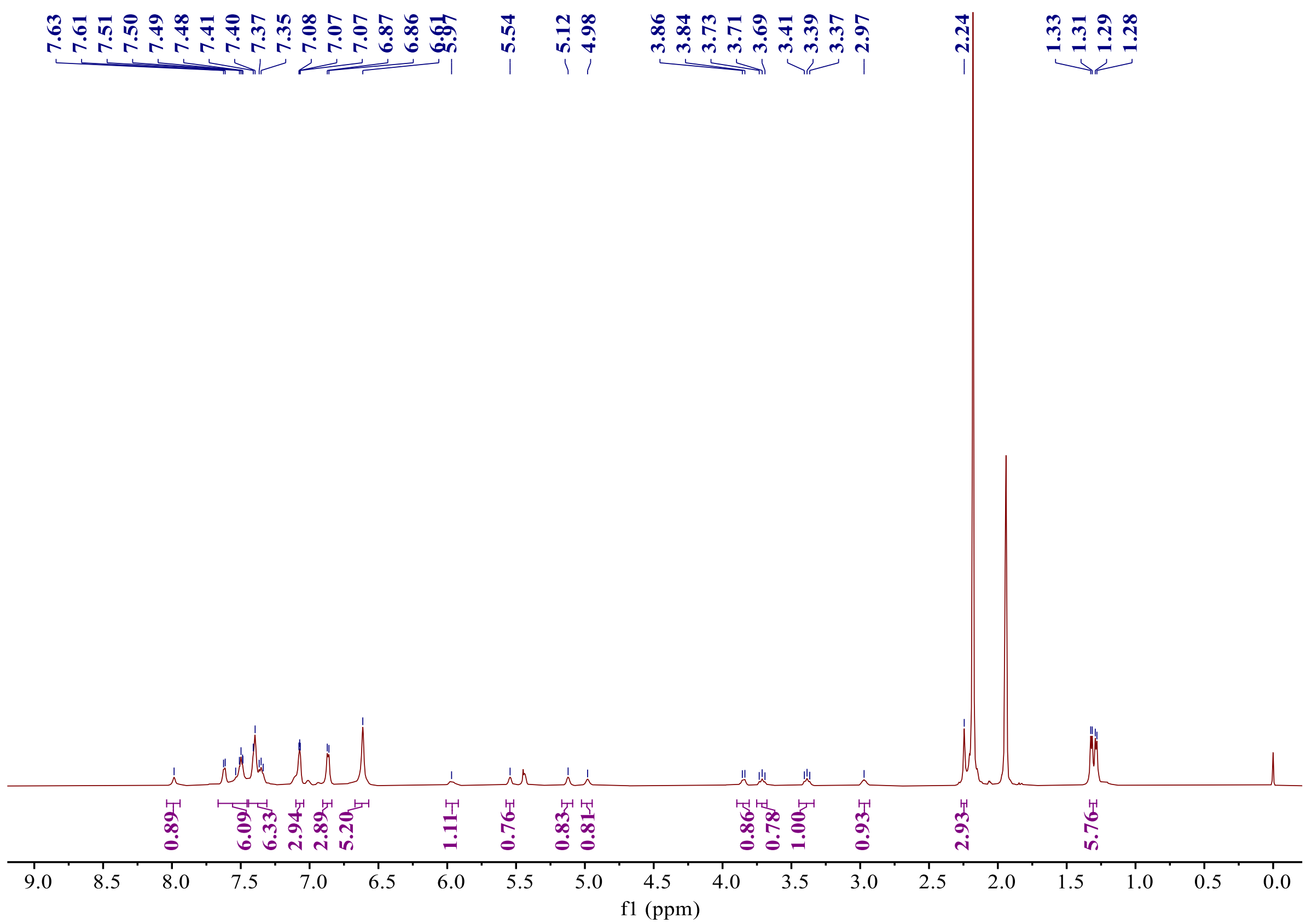

2.6. Synthesis of (*R,R*)-DACH-NH-Boc (9) (tert-butyl ((1R,2R)-2-((4,4''-diamino-[1,1':4',1''-terphenyl])-2'-sulfonamido)cyclohexyl)carbamate):

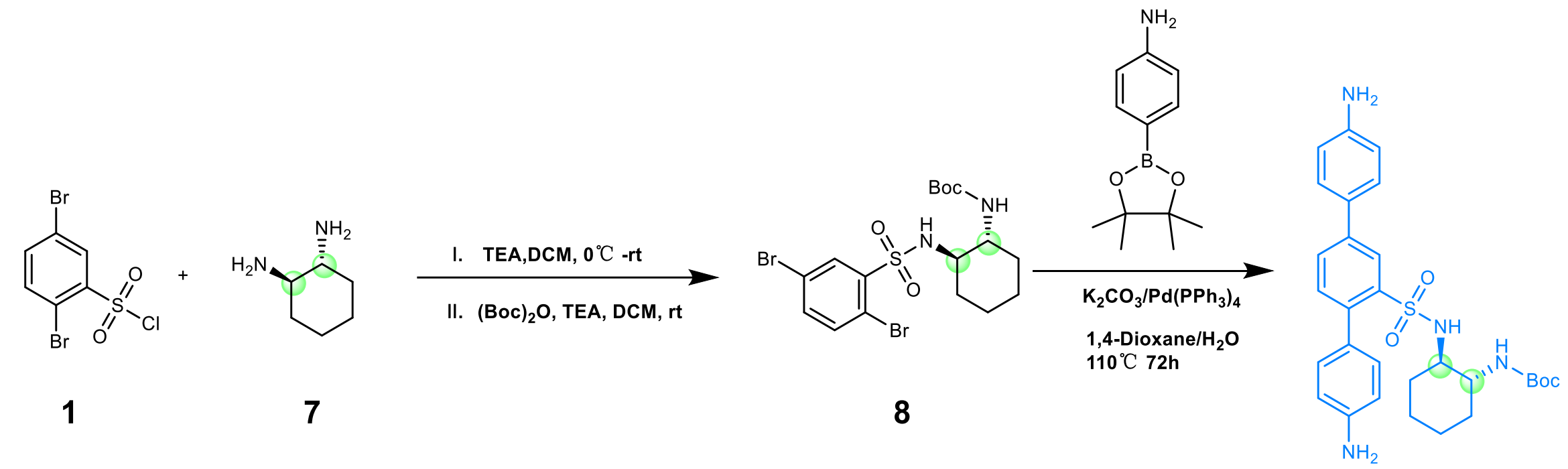

(R,R)-DACH-NH-Boc

9

The synthetic procedure for (*R,R*)-DACH-NH-Boc (**9**) is analogous to that of (*R,R*)-DPEN-NH-Boc, requiring only the substitution of (1R,2R)-1,2-diphenylethane-1,2-diamine (**2**) with (1R,2R)-cyclohexane-1,2-diamine (**7**).

(1R,2R)-cyclohexane-1,2-diamine (**7**, 1.7 g, 14.9 mmol) was dissolved in 60 mL of dichloromethane (DCM), and triethylamine (4.0 mL, 28.7 mmol) was added dropwise with stirring at 0 °C. A solution of 2,5-dibromobenzenesulfonyl chloride (**1**, 5.0 g, 14.9 mmol) in 20 mL of DCM was added slowly to the mixture at 0 °C. After stirring at 0 °C for 30 min, the reaction mixture was warmed to room temperature and stirred for 4 h. Then, the mixture was cooled back to 0 °C, and an additional 4.0 mL of triethylamine was added, followed by slow addition of di--butyl dicarbonate (Boc_2O, 4.0 mL, 17.3 mmol). The reaction was then warmed to room temperature and stirred overnight. The organic solvent was removed under reduced pressure, and the residue was acidified to pH = 2 with 1 M aqueous potassium bisulfate ($KHSO_4$) and extracted with DCM. The combined organic layers were washed with saturated aqueous brine, dried over anhydrous Na_2SO_4, and concentrated under vacuum. Purification was performed via column chromatography on silica gel (200–300 mesh) using petroleum ether / ethyl acetate (PE/EA, 4:1, v/v) as the eluent. The product was dried under vacuum at 60 °C for 12 h to afford 6.3 g of white solid (**8**) in 83% yield.

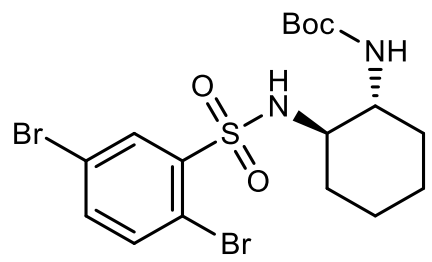

^{1}H NMR (600 MHz, DMSO-D_6) δ 8.05 – 8.03 (d, J = 2.4 Hz, 1H), 7.86 – 7.83 (d, J = 8.8 Hz, 1H), 7.71 – 7.65 (m, 2H), 6.10 – 6.03 (d, J = 8.9 Hz, 1H), 3.21 – 3.13 (qd, J = 10.3, 4.0 Hz, 1H), 2.99 – 2.90 (dtd, J = 14.5, 9.6, 4.2 Hz, 1H), 1.70 – 1.65 (m, 1H), 1.61 – 1.56 (m, 1H), 1.52 – 1.46 (m, 2H), 1.33 – 1.30 (s, 9H), 1.30 – 1.24 (dd, J = 12.5, 2.9 Hz, 1H), 1.17 – 0.94 (m, 2H). ^{13}C NMR (151 MHz, DMSO-D_6) δ 24.6, 24.8, 28.8, 32.6, 33.4, 53.4, 56.8, 78.0, 119.1, 121.1, 133.2, 136.6, 137.5, 143.1, 155.8; DEPT 135° NMR (151 MHz, DMSO-D_6) δ 24.6, 24.8, 28.8, 32.6, 33.4, 53.4, 56.8, 133.2, 136.5, 137.5. ESI-HRMS: Found: m/z 532.9726. Calcd for $C_{17}H_{24}Br_2N_2O_4S$: $(M+Na)^+$ 532.9713.

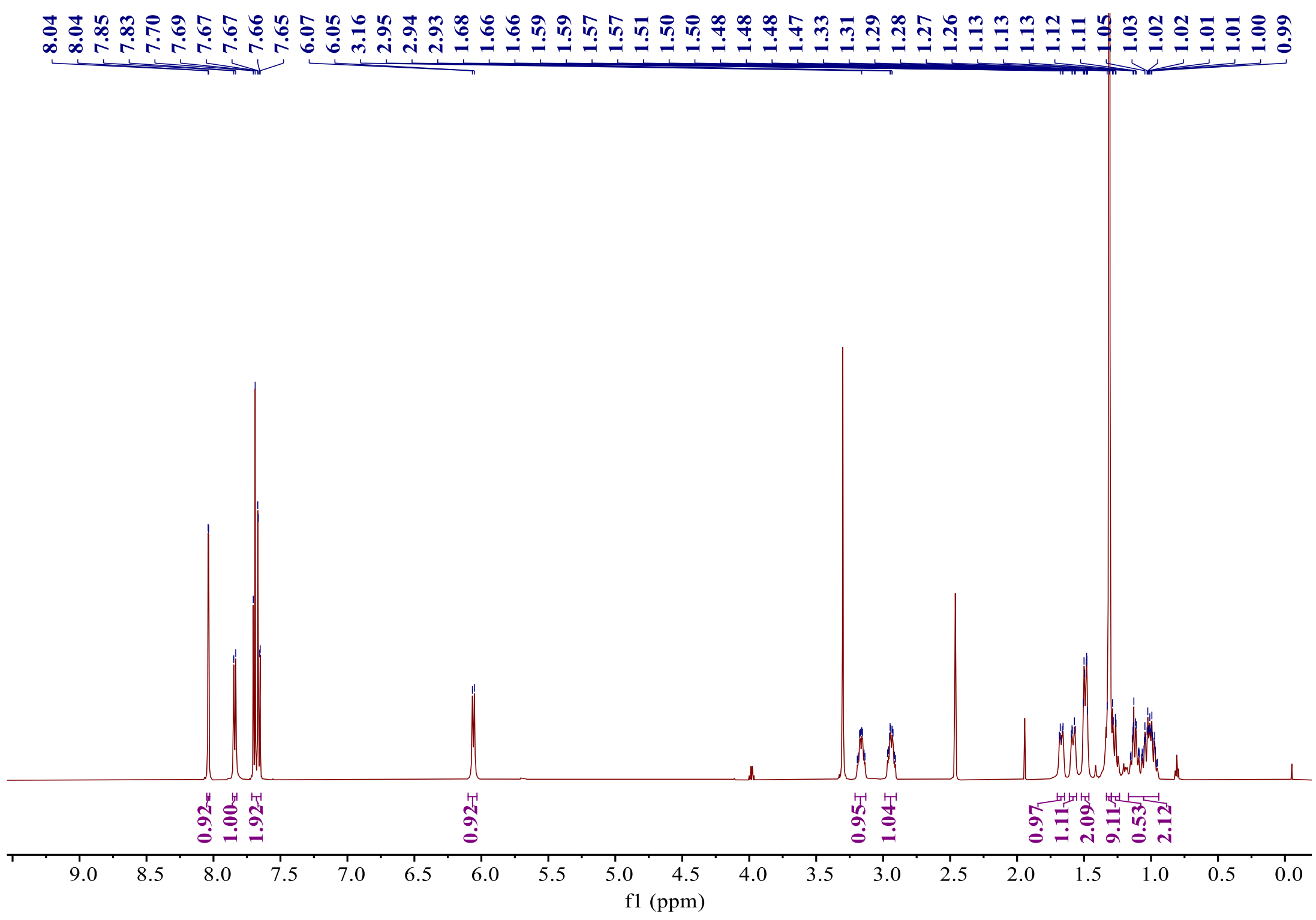

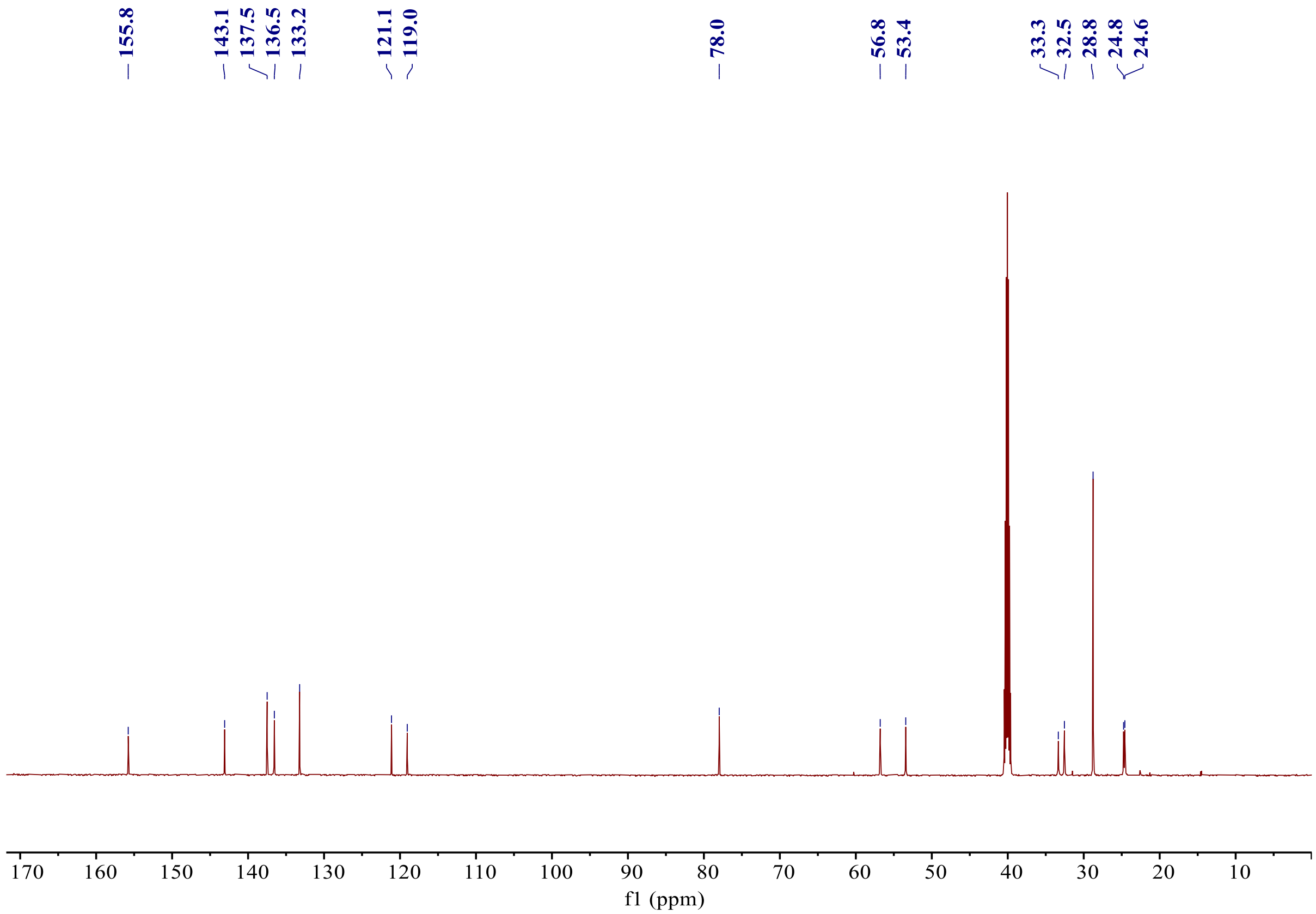
155.8
143.1
137.5
136.5
133.2
121.1
119.0
78.0
56.8
53.4
33.3
32.5
28.8
24.8
24.6
170 160 150 140 130 120 110 100 90 80 70 60 50 40 30 20 10
f1 (ppm)

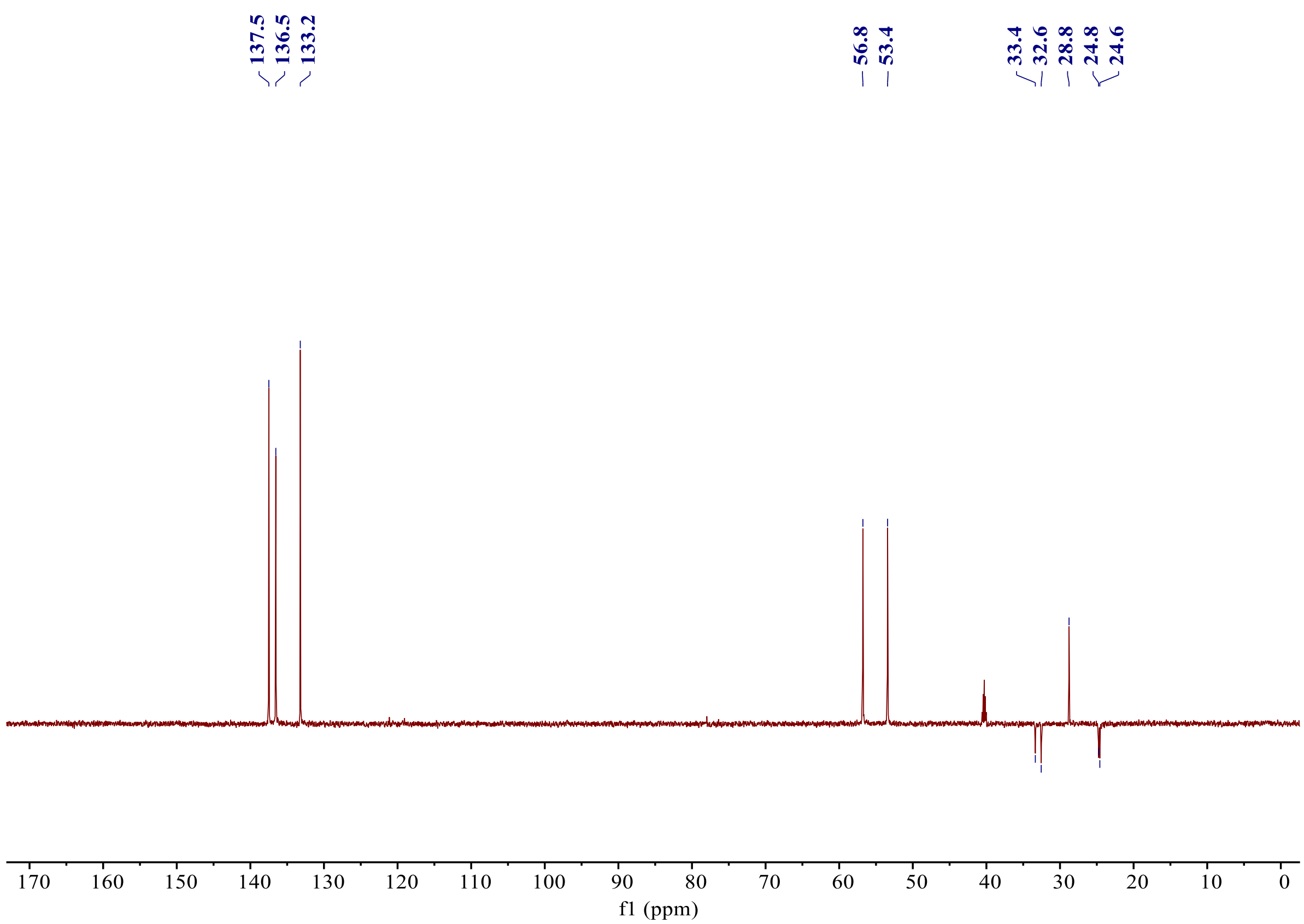
137.5
136.5
133.2
56.8
53.4
33.4
32.6
28.8
24.8
24.6
170 160 150 140 130 120 110 100 90 80 70 60 50 40 30 20 10 0
f1 (ppm)

The ee-value of compound **8** was determined by HPLC analysis with a 25 cm × 4.6 mm Daicel Chiralcel IC column (hexane/i-PrOH = 90/10, 1.0 mL/min). t_{minor} = 11.2 min, t_{major} = 9.7 min; ee = 99.8 %. The racemic sample used in the HPLC analysis was synthesized by replacing (1R,2R)-cyclohexane-1,2-diamine with (±)-trans-1,2-Diaminocyclohexane under the identical synthetic procedure.

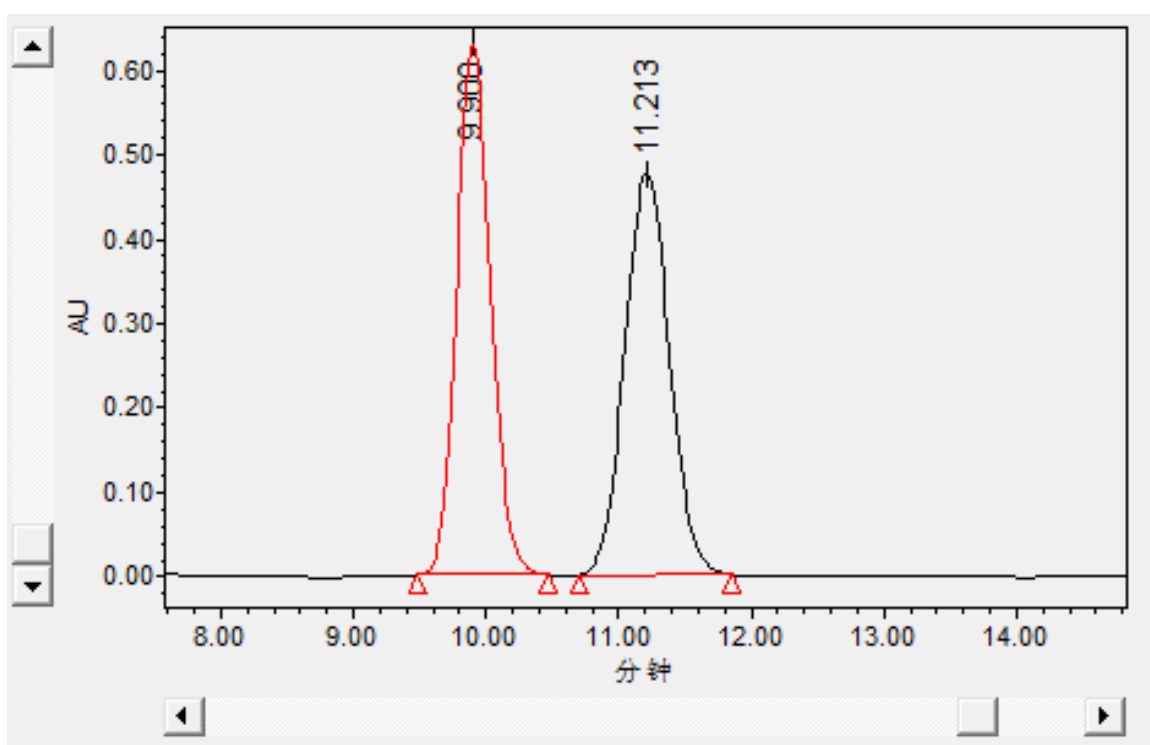

	名称	保留时间 (分钟)	面积 (微伏*秒)	% 面积	高度 (微伏)	积分类型	含量	单位	峰类型	峰代码
1		9.900	11151184	49.93	628404	bb			未知	
2		11.213	11182430	50.07	475941	bb			未知	

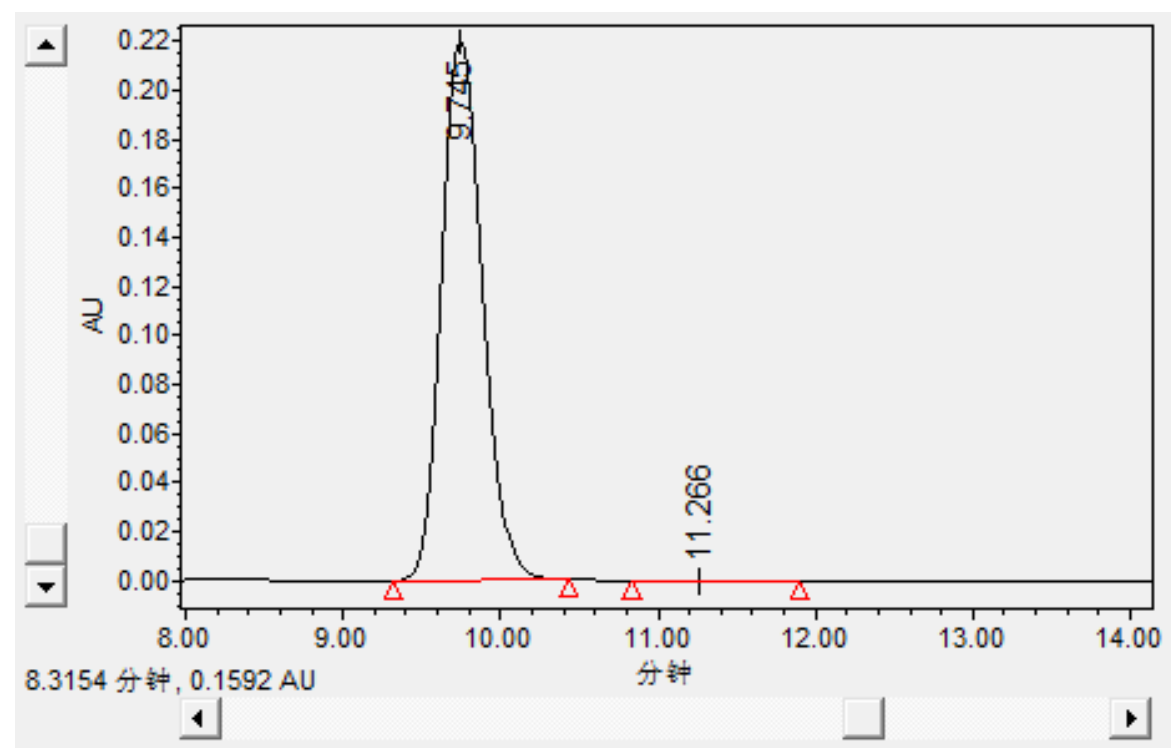

	名称	保留时间 (分钟)	面积 (微伏*秒)	% 面积	高度 (微伏)	积分类型	含量	单位	峰类型	峰代码
1		9.745	3956162	99.93	219443	bb			未知	
2		11.266	2701	0.07	130	bb			未知	

Peaks number	Retention time (min)	Area	Area percentage (%)	Height of peaks
1	9.745	3956162	99.93	219443
2	11.266	2701	0.07	130

Compound **8** (3.0 g, 5.86 mmol), 4-(4,4,5,5-tetramethyl-1,3,2-dioxaborolan-2-yl)aniline (3.86 g, mmol), potassium carbonate (K_2CO_3, 3.23 g, mmol), and tetrakis(triphenylphosphine)palladium(0) ($Pd(PPh_3)_4$, 0.41 mmol) were charged into a 250 mL three-necked flask. The reaction system was degassed and backfilled with nitrogen three times to maintain an inert atmosphere, followed by addition of a pre-degassed mixed solvent of 1,4-dioxane/water (4:1, v/v, 170 mL). The mixture was heated to 110 °C and stirred at this temperature for 72 h. After cooling to room temperature,

insoluble residues were removed by filtration, and the organic solvent was evaporated under reduced pressure.

The crude residue was extracted with dichloromethane (DCM), and the combined organic phase was washed with saturated aqueous brine, dried over anhydrous sodium sulfate (Na_2SO_4), and concentrated under vacuum. Purification was carried out by column chromatography on silica gel with DCM/methanol (25:1, v/v) as the eluent. The resulting product was dried under vacuum at 60 °C for 24 h to afford 1.75 g of pale yellow powder, identified as (*R,R*)-DACH-NH-Boc (**9**), in 56% yield with 99.4% enantiomeric excess (e.e.).

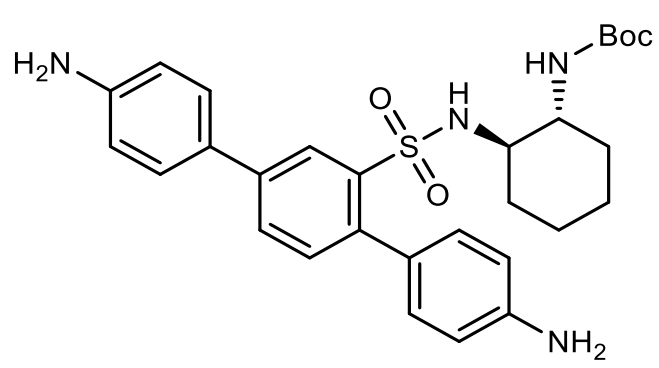

^{1}H NMR (600 MHz, DMSO-D_6) δ 8.14 – 8.13 (d, *J* = 2.0 Hz, 1H), 7.75 – 7.70 (dd, *J* = 8.0, 2.0 Hz, 1H), 7.65 – 7.54 (m, 2H), 7.46 – 7.43 (m, 2H), 7.27 – 7.20 (d, *J* = 8.0 Hz, 1H), 7.12 – 7.06 (d, *J* = 8.3 Hz, 2H), 6.91 – 6.84 (d, *J* = 8.3 Hz, 1H), 6.72 – 6.65 (m, 2H), 6.61 – 6.54 (m, 2H), 6.27 – 6.20 (d, *J* = 8.1 Hz, 1H), 5.41 – 5.33 (m, 2H), 5.25 – 5.15 (s, 2H), 3.15 – 3.09 (d, *J* = 9.9 Hz, 1H), 2.89 – 2.80 (dd, *J* = 8.4, 4.9 Hz, 1H), 1.83 – 1.67 (d, *J* = 11.9 Hz, 1H), 1.52 – 1.43 (q, *J* = 13.4 Hz, 3H), 1.35 – 1.31 (s, 9H), 1.18 – 1.03 (m, 2H); ^{13}C NMR (151 MHz, DMSO-*D*6) δ 24.4, 24.6, 28.8, 32.2, 53.7, 56.9, 78.2, 113.6, 114.8, 125.1, 126.1, 127.4, 127.7, 128.5, 129.3, 129.3, 130.6, 132.0, 132.1, 132.6, 132.9, 133.6, 134.0, 138.6, 139.4, 141.6, 148.5, 149.5, 156.0; DEPT 135° NMR (151 MHz, DMSO-D_6) δ 24.4, 24.6, 28.8, 32.2, 53.7, 56.9, 113.6, 114.8, 125.1, 127.7, 128.5, 129.3, 129.3, 130.6, 132.0, 132.1, 132.6, 132.6, 134.0; ESI-HRMS: Found: m/z 559.2374. Calcd for $C_{29}H_{36}N_4O_4S$: $(M+Na)^+$ 559.2349.

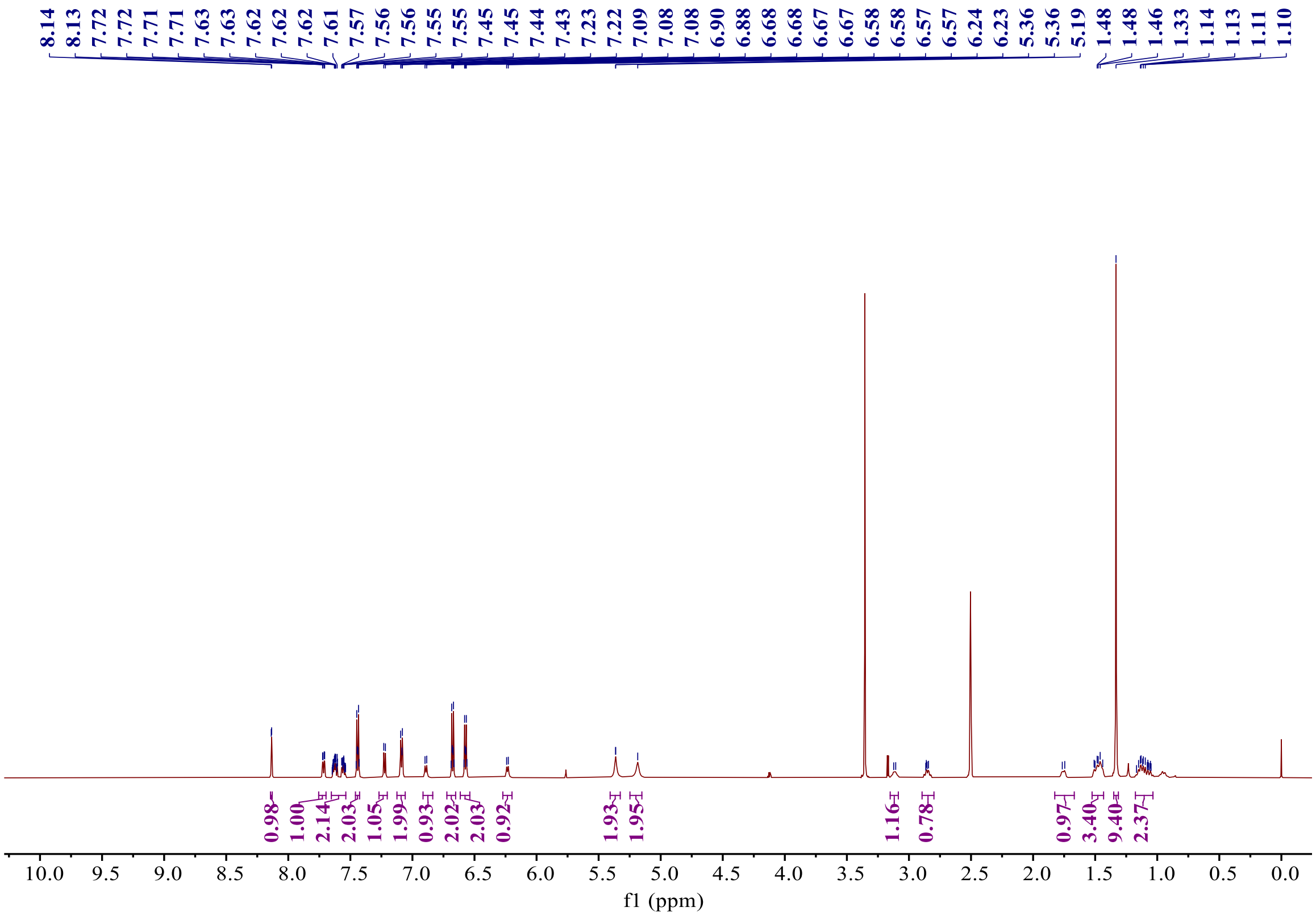
8.14
8.13
7.72
7.72
7.71
7.71
7.63
7.63
7.62
7.62
7.61
7.57
7.56
7.56
7.55
7.55
7.45
7.45
7.44
7.43
7.23
7.22
7.09
7.08
7.08
6.90
6.88
6.68
6.68
6.67
6.67
6.58
6.58
6.57
6.57
6.24
6.23
5.36
5.36
5.19
1.48
1.48
1.46
1.33
1.14
1.13
1.11
1.10
0.98
1.00
2.14
2.03
1.05
1.99
0.93
2.02
2.03
0.92
1.93
1.95
1.16
0.78
0.97
3.40
9.40
2.37
f1 (ppm)

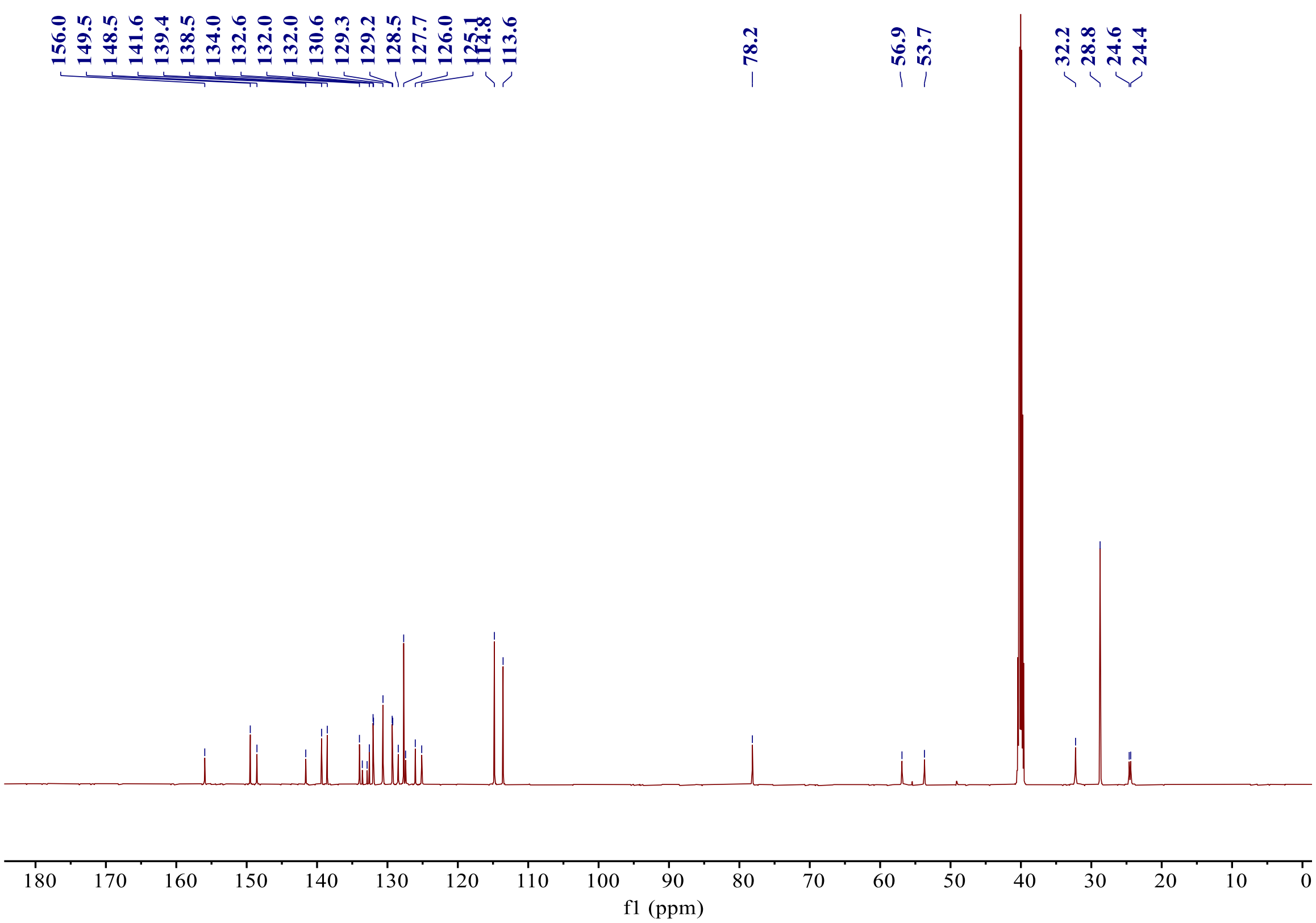
156.0
149.5
148.5
141.6
139.4
138.5
134.0
132.6
132.0
132.0
130.6
129.3
129.2
128.5
127.7
126.0
125.1
114.8
113.6
78.2
56.9
53.7
32.2
28.8
24.6
24.4
f1 (ppm)

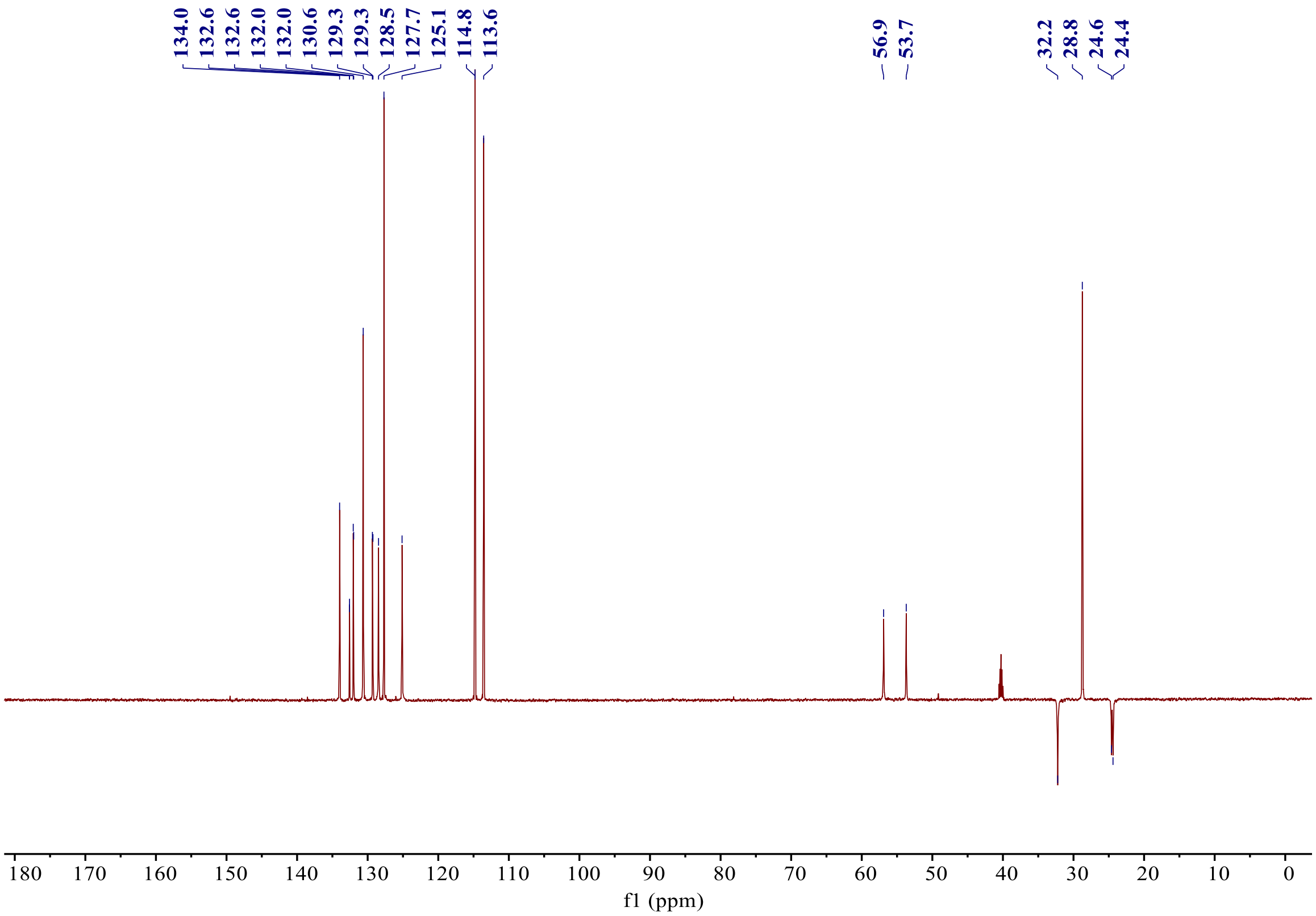

The ee-value of compound **9** was determined by HPLC analysis with a 25 cm × 4.6 mm Daicel Chiralcel IE column (hexane/i-PrOH = 60/40, 1.0 mL/min). t_{minor} = 46.0 min, t_{major} = 117.2 min; ee = 99.4 %. The racemic reference was prepared following the same synthetic procedure, starting from racemic **compound 8**, which was derived from (±)-trans-1,2-diaminocyclohexane instead of the (1R,2R) enantiomer.

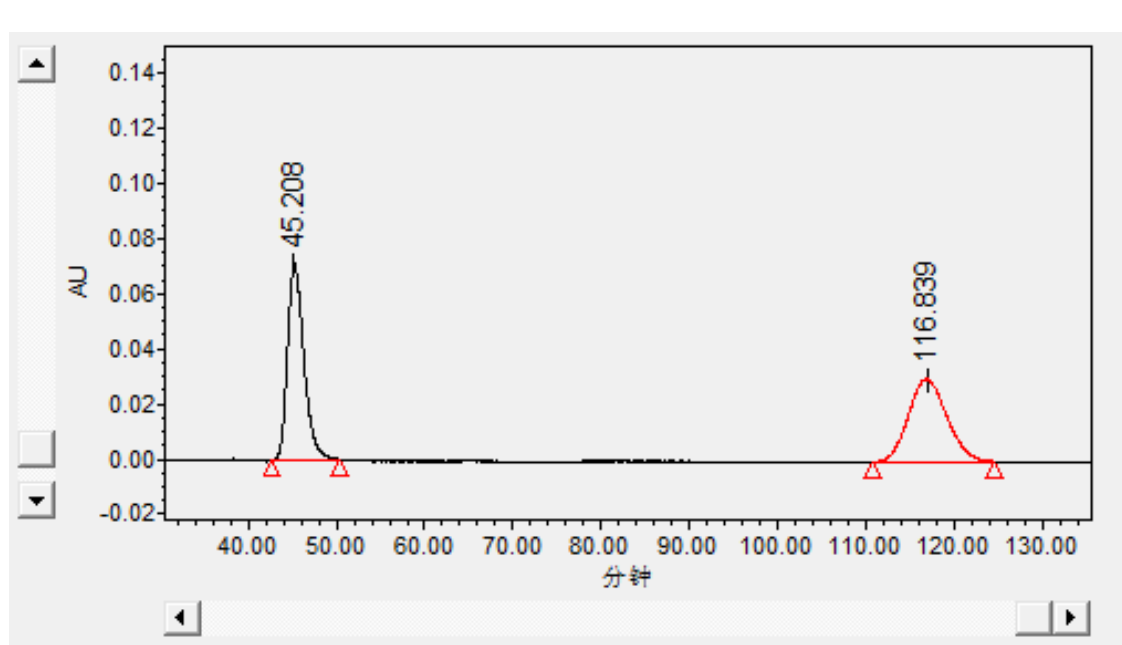

	名称	保留时间 (分钟)	面积 (微伏*秒)	% 面积	高度 (微伏)	积分类型	含量	单位	峰类型	峰代码
1		45.208	9215384	50.08	71259	bb			未知	
2		116.839	9185513	49.92	30278	bb			未知	

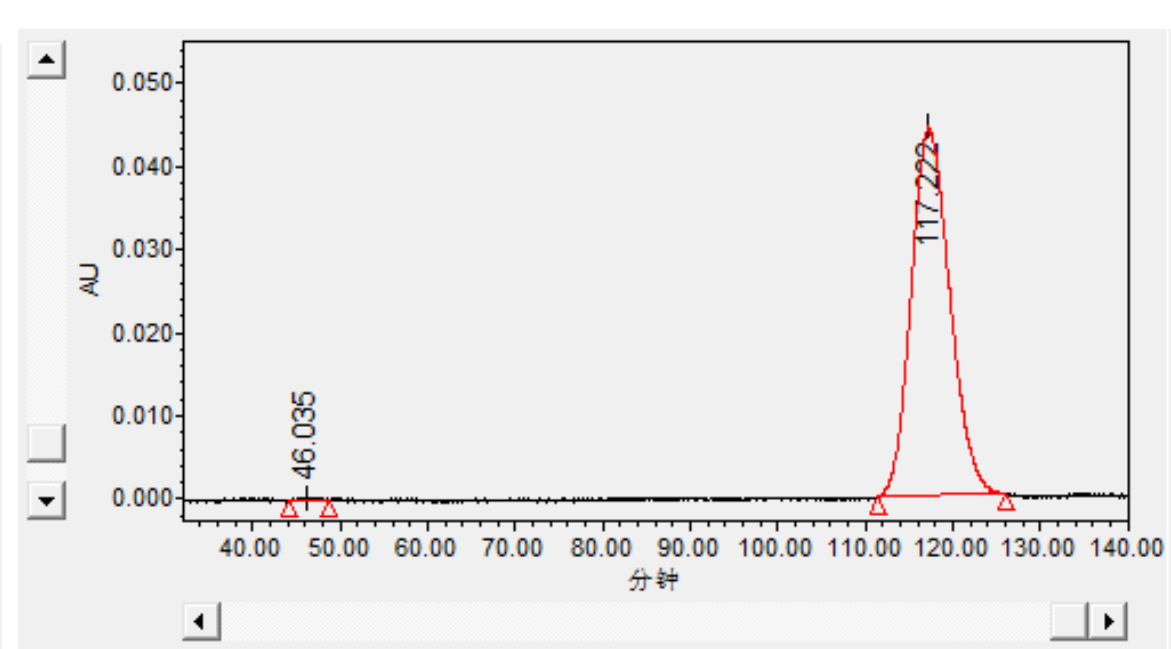

	名称	保留时间 (分钟)	面积 (微伏*秒)	% 面积	高度 (微伏)	积分类型	含量	单位	峰类型	峰代码
1		46.035	41310	0.30	355	bb			未知	
2		117.222	13539583	99.70	44407	bb			未知	

Peaks number	Retention time (min)	Area	Area percentage (%)	Height of peaks
1	46.035	41310	0.3	355
2	117.222	13539583	99.7	44407

2.7. Synthesis of CCOFX-Boc:

Synthesis of CCOF0-Boc:

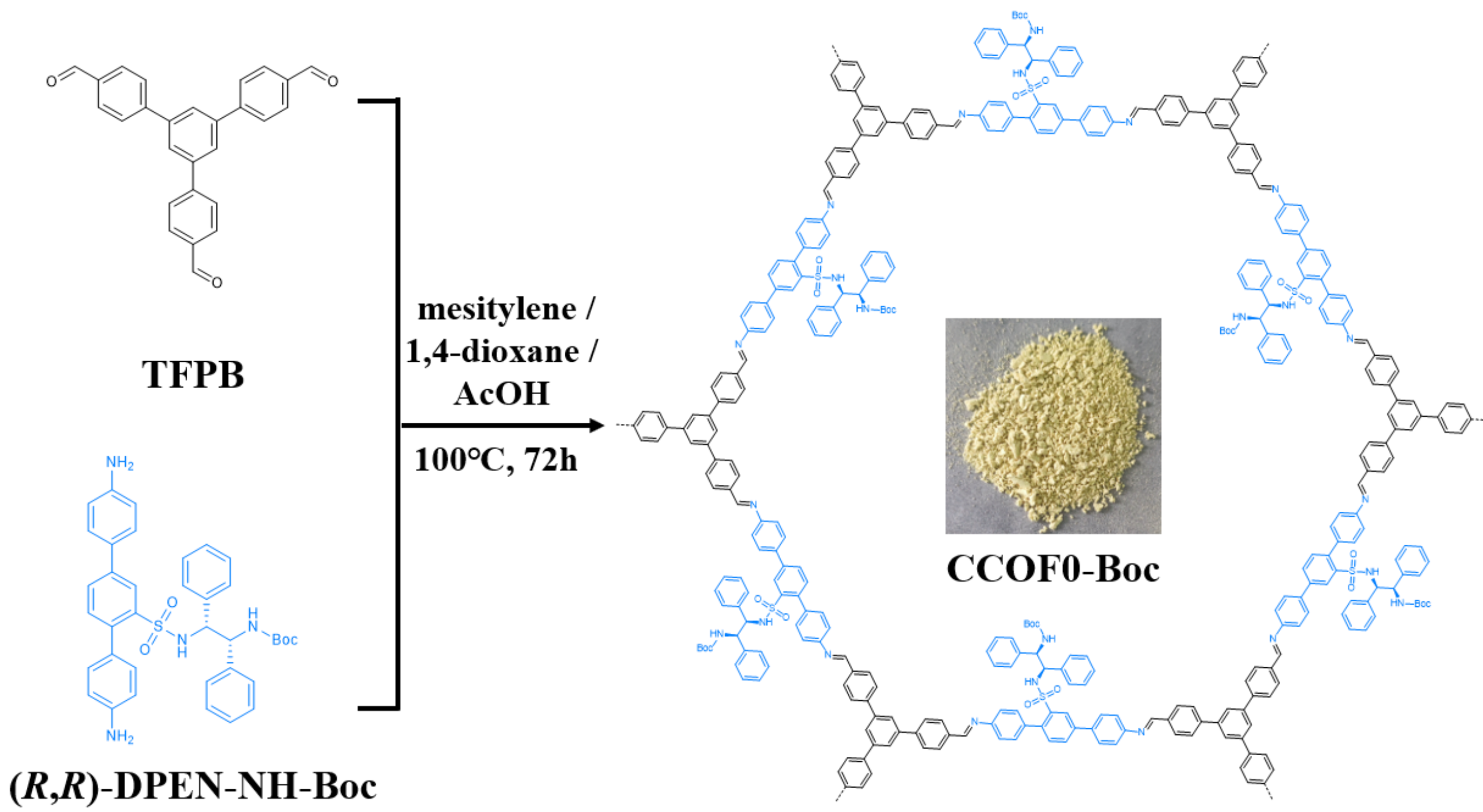

CCOF0-Boc. Add 46.8 mg of TFPB (0.12 mmol) and 114 mg of (*R,R*)-DPEN-NH-Boc (0.18 mmol) to a 15 mL Pyrex tube. A mixture of 3 mL mesitylene and 1,4-dioxane (v/v ratio 4:1) was introduced as the solvent, followed by ultrasonic treatment for 5 minutes to ensure uniform dispersion of the components. Subsequently, 50 μL of 3 M acetic acid was added as a catalyst, and the mixture underwent additional ultrasonic treatment for 10 minutes to facilitate thorough mixing. The system was then degassed through three freeze-pump-thaw cycles to remove dissolved gases. The tube was sealed, and the mixture was heated at 100°C for 3 days to allow the reaction to

proceed to completion. After cooling, the resulting yellow-green powder was collected via filtration and washed sequentially with ethanol and dichloromethane. The powder was subsequently subjected to Soxhlet extraction using tetrahydrofuran (THF) and acetone for 24 hours to achieve further purification. The final product was obtained by overnight vacuum drying at 60°C. Yield: 98 mg (64 %).

Synthesis of CCOF1-Boc:

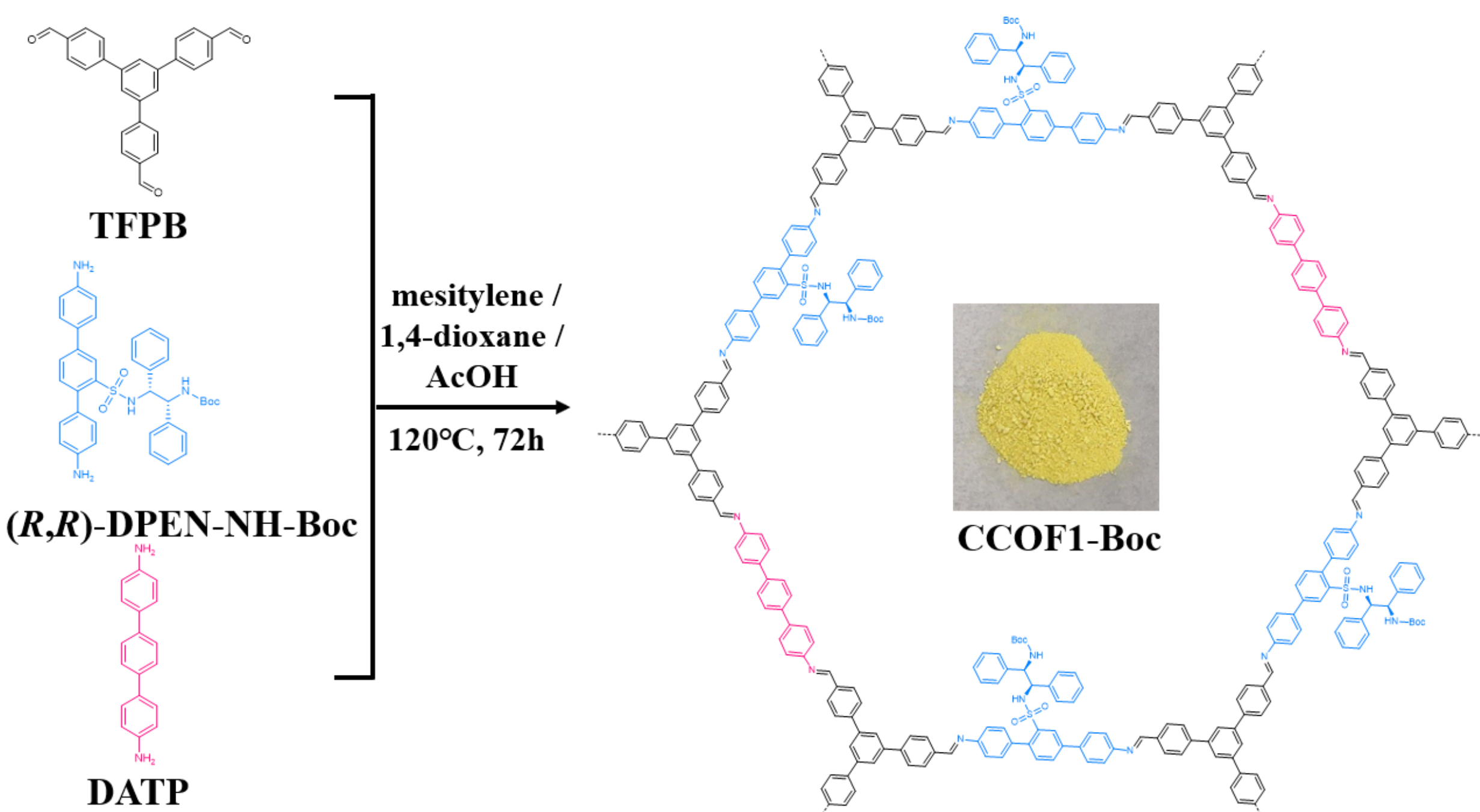

CCOF1-Boc. Add 46.8 mg of TFPB (0.12 mmol), 15.6 mg DATP (0.06 mmol) and 76 mg (*R,R*)-DPEN-NH-Boc (0.12 mmol) to a 15 mL Pyrex tube. A mixture of 3 mL mesitylene and 1,4-dioxane (v/v ratio 4:1) was introduced as the solvent, followed by ultrasonic treatment for 5 minutes to ensure uniform dispersion of the components. Subsequently, 150 μL of 3 M acetic acid was added as a catalyst, and the mixture underwent additional ultrasonic treatment for 10 minutes to facilitate thorough mixing. The system was then degassed through three freeze-pump-thaw cycles to remove dissolved gases. The tube was sealed, and the mixture was heated at 120°C for 3

days to allow the reaction to proceed to completion. After cooling, the resulting yellow-green powder was collected via filtration and washed sequentially with ethanol and dichloromethane. The powder was subsequently subjected to Soxhlet extraction using tetrahydrofuran (THF) and acetone for 24 hours to achieve further purification. The final product was obtained by overnight vacuum drying at 60°C. Yield: 105 mg (80 %).

Synthesis of CCOF2-Boc:

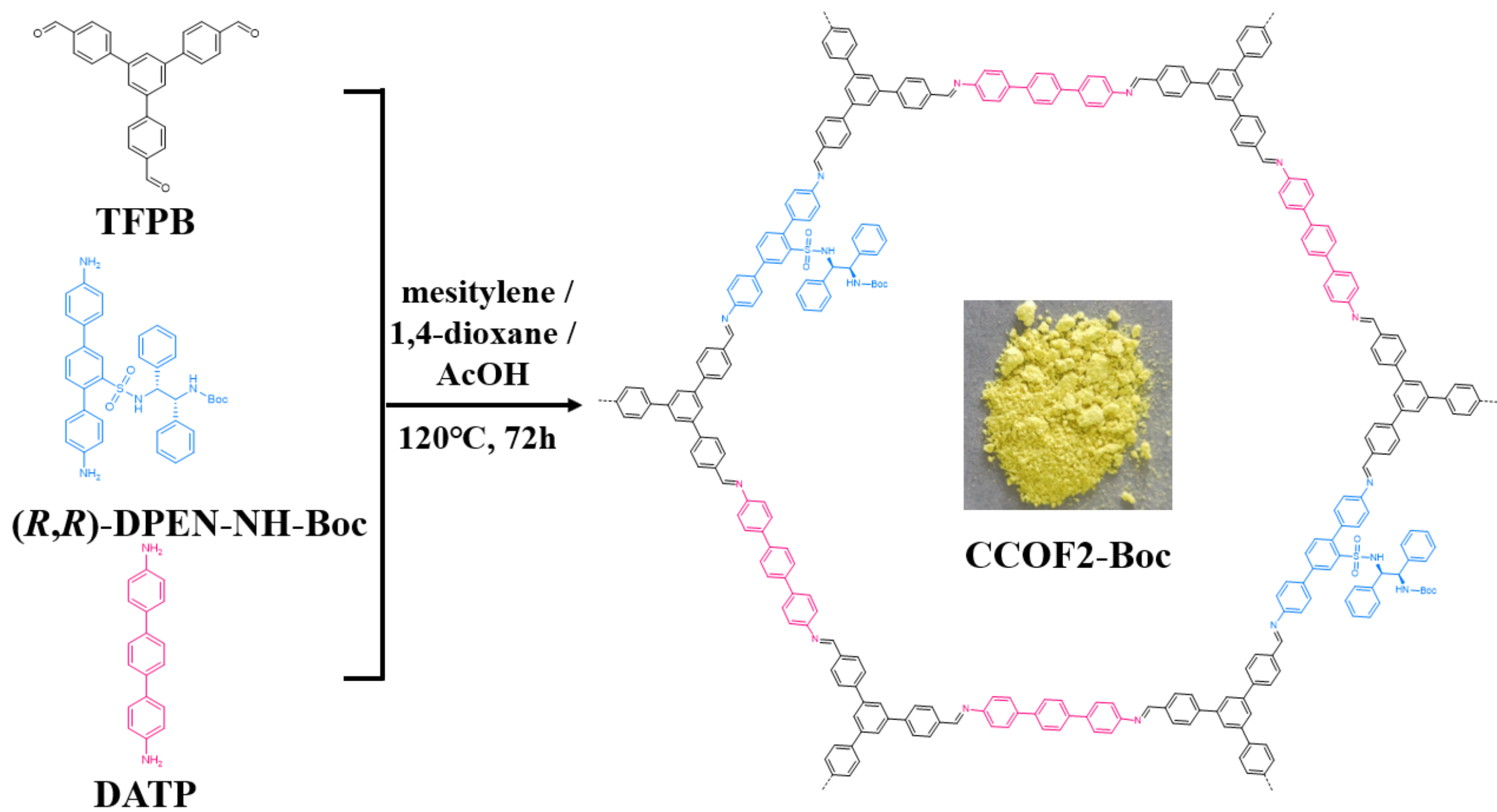

CCOF2-Boc. Add 46.8 mg of TFPB (0.12 mmol), 31.2 mg DATP (0.12 mmol) and 38 mg (*R,R*)-DPEN-NH-Boc (0.06 mmol) to a 15 mL Pyrex tube. A mixture of 3 mL mesitylene and 1,4-dioxane (v/v ratio 4:1) was introduced as the solvent, followed by ultrasonic treatment for 5 minutes to ensure uniform dispersion of the components. Subsequently, 250 μL of 3 M acetic acid was added as a catalyst, and the mixture underwent additional ultrasonic treatment for 10 minutes to facilitate thorough mixing. The system was then degassed through three freeze-pump-thaw cycles to remove dissolved gases. The tube was sealed, and the mixture was heated at 120°C for 3

days to allow the reaction to proceed to completion. After cooling, the resulting yellow-green powder was collected via filtration and washed sequentially with ethanol and dichloromethane. The powder was subsequently subjected to Soxhlet extraction using tetrahydrofuran (THF) and acetone for 24 hours to achieve further purification. The final product was obtained by overnight vacuum drying at 60°C. Yield: 92 mg (84 %).

2.8. Synthesis of CCOF-DACH-Ru:

Synthesis of CCOF-DACH-Boc

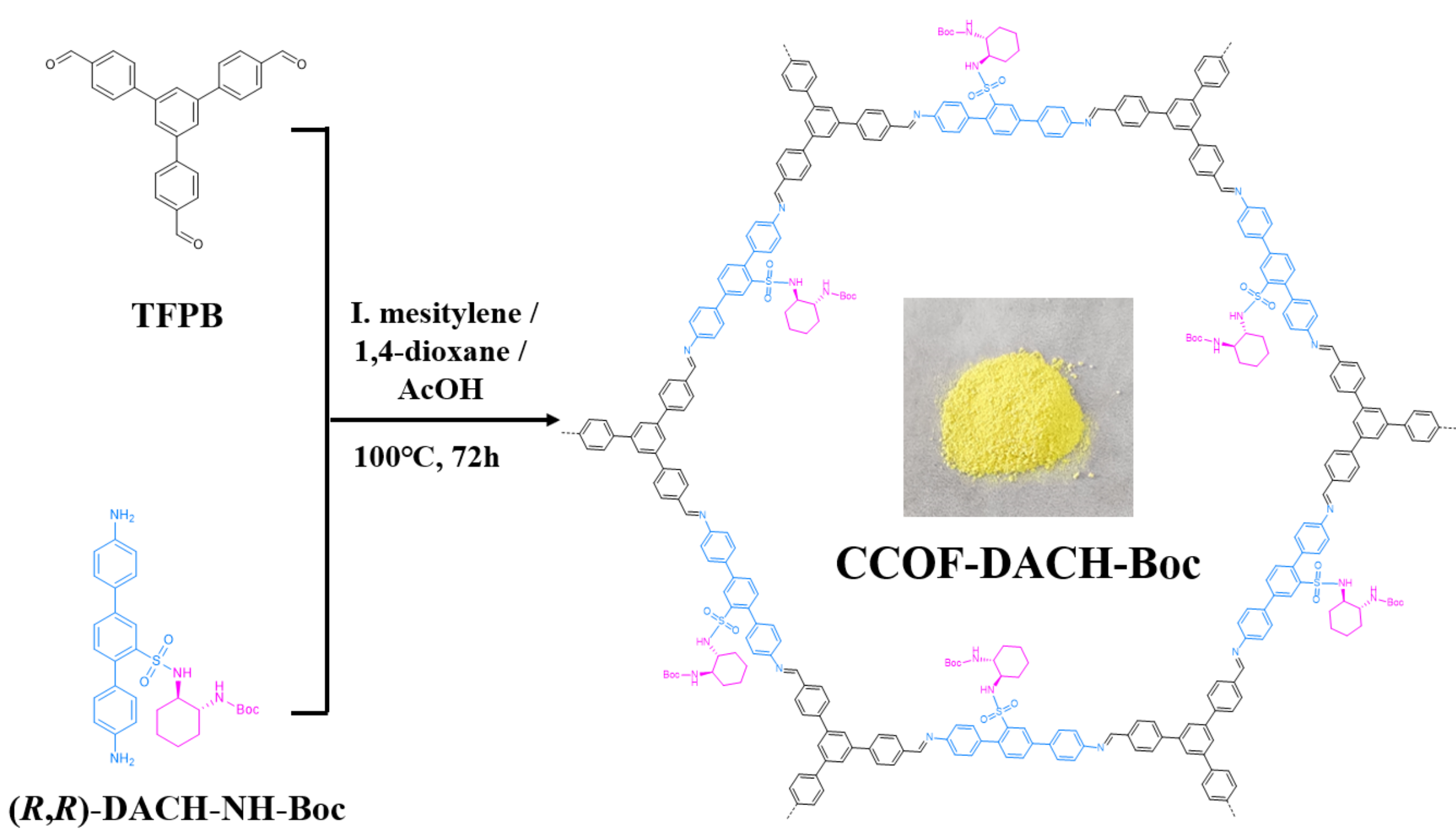

Add 46.8 mg of TFPB (0.12 mmol) and 96.5 mg of (*R,R*)-DACH-NH-Boc (**9**) (0.18 mmol) to a 15 mL Pyrex tube. A mixture of 3 mL mesitylene and 1,4-dioxane (v/v ratio 4:1) was introduced as the solvent, followed by ultrasonic treatment for 5 minutes to ensure uniform dispersion of the components. Subsequently, 100 μL of 3 M acetic acid was added as a catalyst, and the mixture underwent additional ultrasonic treatment for 10 minutes to facilitate thorough mixing. The system was then degassed through three freeze-pump-thaw cycles to remove dissolved gases. The tube

was sealed, and the mixture was heated at 100°C for 3 days to allow the reaction to proceed to completion. After cooling, the resulting yellow powder was collected via filtration and washed sequentially with ethanol and dichloromethane. The powder was subsequently subjected to Soxhlet extraction using tetrahydrofuran (THF) and acetone for 24 hours to achieve further purification. The CCOF-DACH-Boc was obtained by overnight vacuum drying at 60°C. Yield: 75 mg (55 %).

Synthesis of CCOF-DACH

CCOF-DACH-Boc (300 mg) was charged into an oven-dried 25 mL round-bottom flask equipped with a magnetic stir bar. Under an ice-water bath (0 °C), anhydrous dichloromethane (6 mL) was introduced via syringe followed by dropwise addition of trimethylsilyl trifluoromethanesulfonate (TMSOTf, 0.1 mL). The reaction mixture was maintained at 0 °C with vigorous stirring for 6 h. Upon completion, the suspension was filtered through a Büchner funnel. The collected solid was sequentially washed with cold dichloromethane, saturated aqueous $NaHCO_3$ solution, deionized water and ethanol. The crude product was subsequently subjected to purification by Soxhlet extraction using acetone as eluent (24 h continuous extraction). Finally, the purified sample was dried under high vacuum at 80 °C for 12 h to afford CCOF-DACH.

Synthesis of CCOF-DACH-Ru

A mixture of CCOF-DACH (40 mg), dichloromethane (DCM, 5 mL), and $[Ru(p\text{-cymene})Cl_2]_2$ (8.2 mg, chiral diamine ligand-to-Ru molar ratio = 2:1) was charged into a 15 mL Schlenk tube under argon atmosphere. The reaction was vigorously stirred at 40 °C for 4 h. Upon completion, the resultant solid was collected by filtration and purified via Soxhlet extraction with DCM (24 h) to remove unreacted species. Subsequently, the product was dried under reduced pressure at 40 °C for 12 h to afford the target complex CCOF-DACH-Ru.

2.9. Synthesis of TFPB-DATP-Ru:

Synthesis of TFPB-DATP

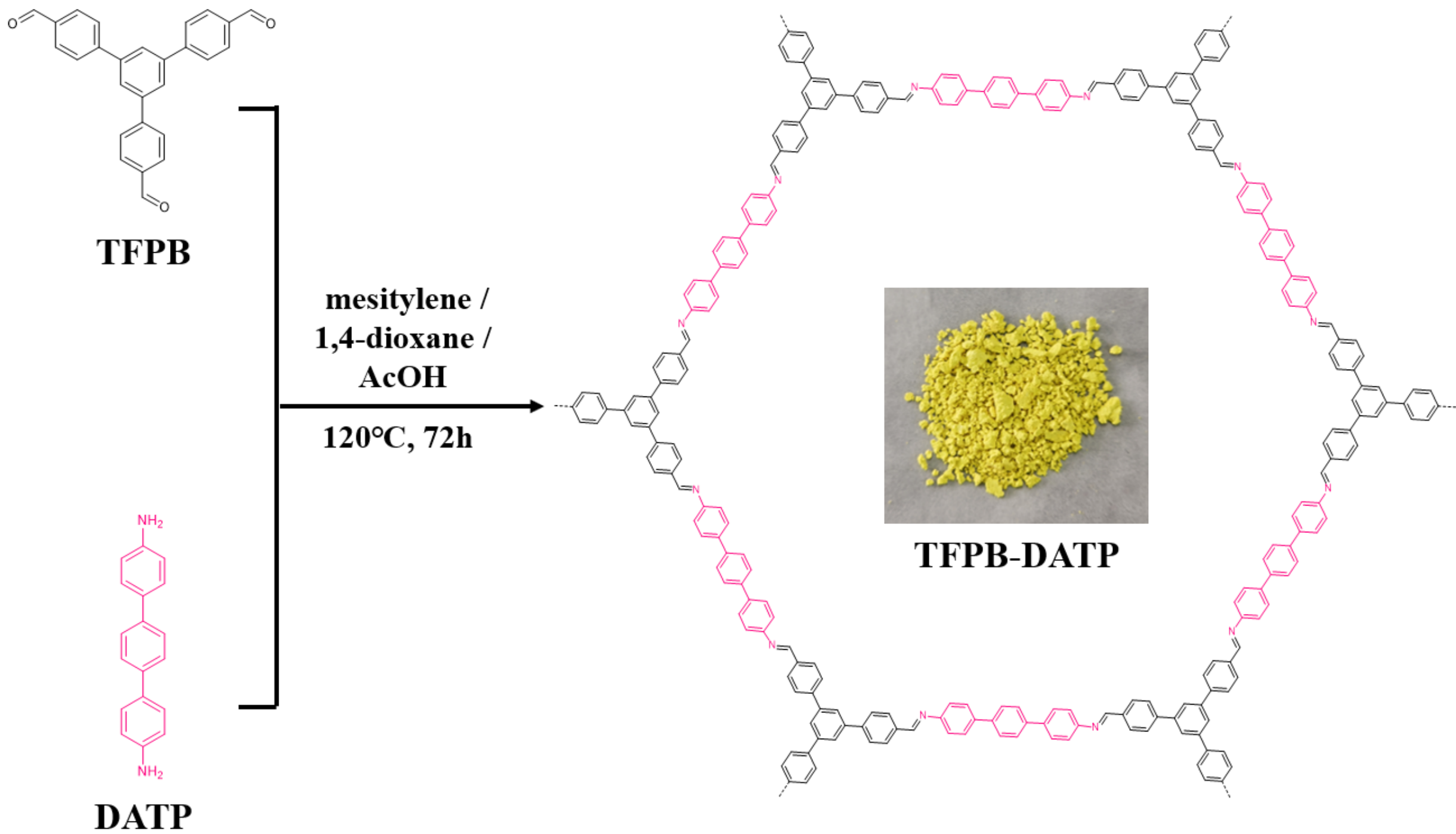

Initially, TFPB-DATP was prepared with a modest modification to the synthetic protocol reported in previous literature.[5, 6] Add 46.8 mg of TFPB (0.12 mmol) and 46.8 mg of DATP (0.18 mmol) to a 15 mL Pyrex tube. A mixture of 3 mL mesitylene and 1,4-dioxane (v/v ratio 4:1) was introduced as the solvent, followed by ultrasonic treatment for 5 minutes to ensure uniform dispersion of the components. Subsequently, 200 μL of 3 M acetic acid was added as a catalyst, and the mixture underwent additional ultrasonic treatment for 10 minutes to facilitate thorough mixing. The system was then degassed through three freeze-pump-thaw cycles to remove dissolved gases. The tube was sealed, and the mixture was heated at 120°C for 3 days to allow the reaction to proceed to completion. After cooling, the resulting yellow powder was collected via filtration and washed sequentially with ethanol and dichloromethane. The powder was subsequently subjected to Soxhlet extraction using tetrahydrofuran (THF) and acetone for 24 hours

to achieve further purification. The TFPB-DATP was obtained by overnight vacuum drying at 60°C.

Synthesis of TFPB-DATP-Ru

A mixture of TFPB-DATP (200 mg), dichloromethane (DCM, 5 mL), and $[Ru(p\text{-cymene})Cl_2]_2$ (17.7mg, the same loading as used for the synthesis of CCOF2-Ru) was charged into a 15 mL Schlenk tube under argon atmosphere. The reaction was vigorously stirred at 40 °C for 4 h. Upon completion, the resulting solid was collected by filtration and further purified by Soxhlet extraction with dichloromethane (DCM) for 24 h to remove unreacted substrates. The product was then dried under vacuum at 40 °C for 12 h, yielding TFPB-DATP-Ru. The ruthenium loading was determined to be 0.17 wt% by inductively coupled plasma optical emission spectrometry (ICP-OES).

Section 3. Supplementary Figures.

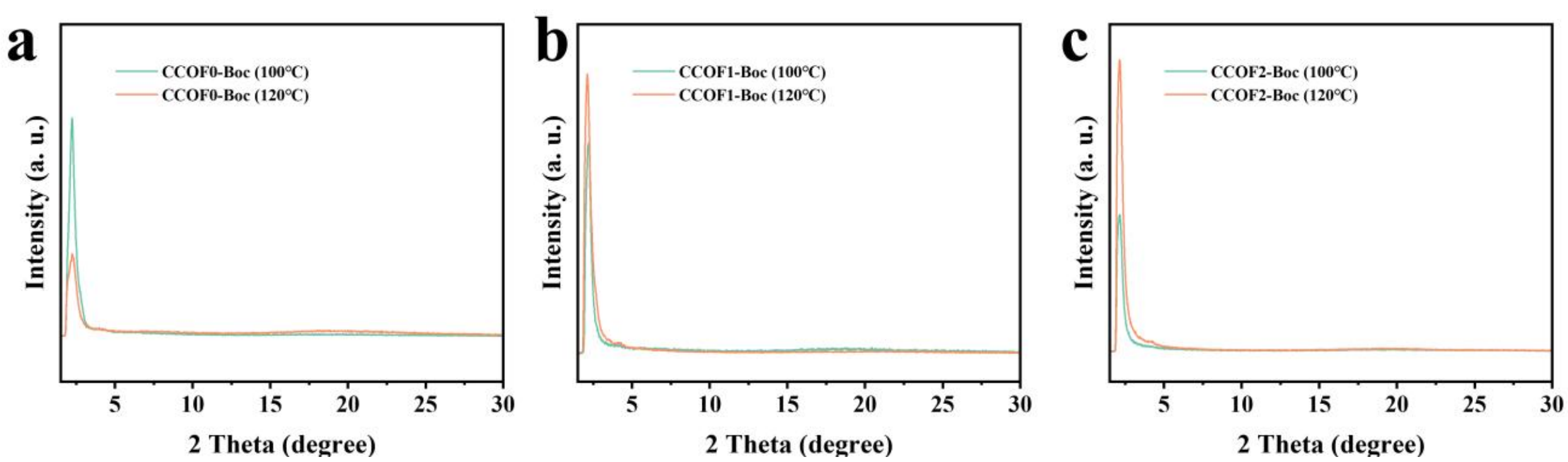

Supplementary Fig. 1 PXRD patterns of **a** CCOF0-Boc, **b** CCOF1-Boc and **c** CCOF2-Boc synthesized at 100 °C and 120 °C. CCOF0- Boc: higher crystallinity at 100 °C; CCOF1- Boc and CCOF2- Boc: higher crystallinity at 120 °C. The retention of a substantial amount of Boc groups in CCOF1-BOC and CCOF2-BOC was confirmed by FTIR (Supplementary Fig. 2, the C=O peak of Boc at 1694 cm^{-1}), solid-state NMR (Supplementary Fig. 3, δ = 80, 27 ppm) and TGA curves (Supplementary Fig. 9, an obvious mass loss starting at approximately 210 °C). These results

verify that the employed solvothermal conditions do not significantly affect the synthesis of the CCOFs.

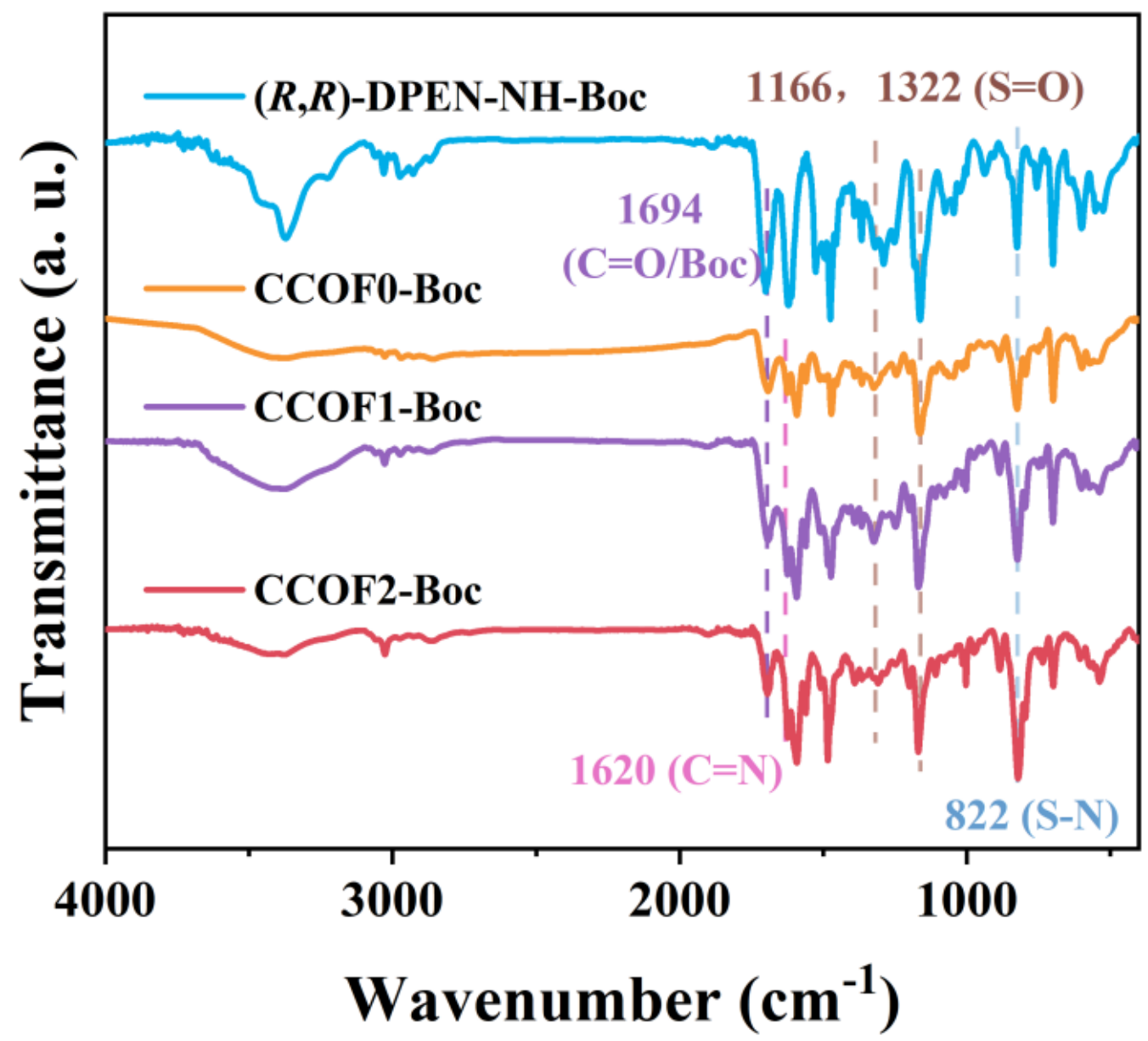

Supplementary Fig. 2 FTIR spectra of Boc-protected CCOFX-Boc.

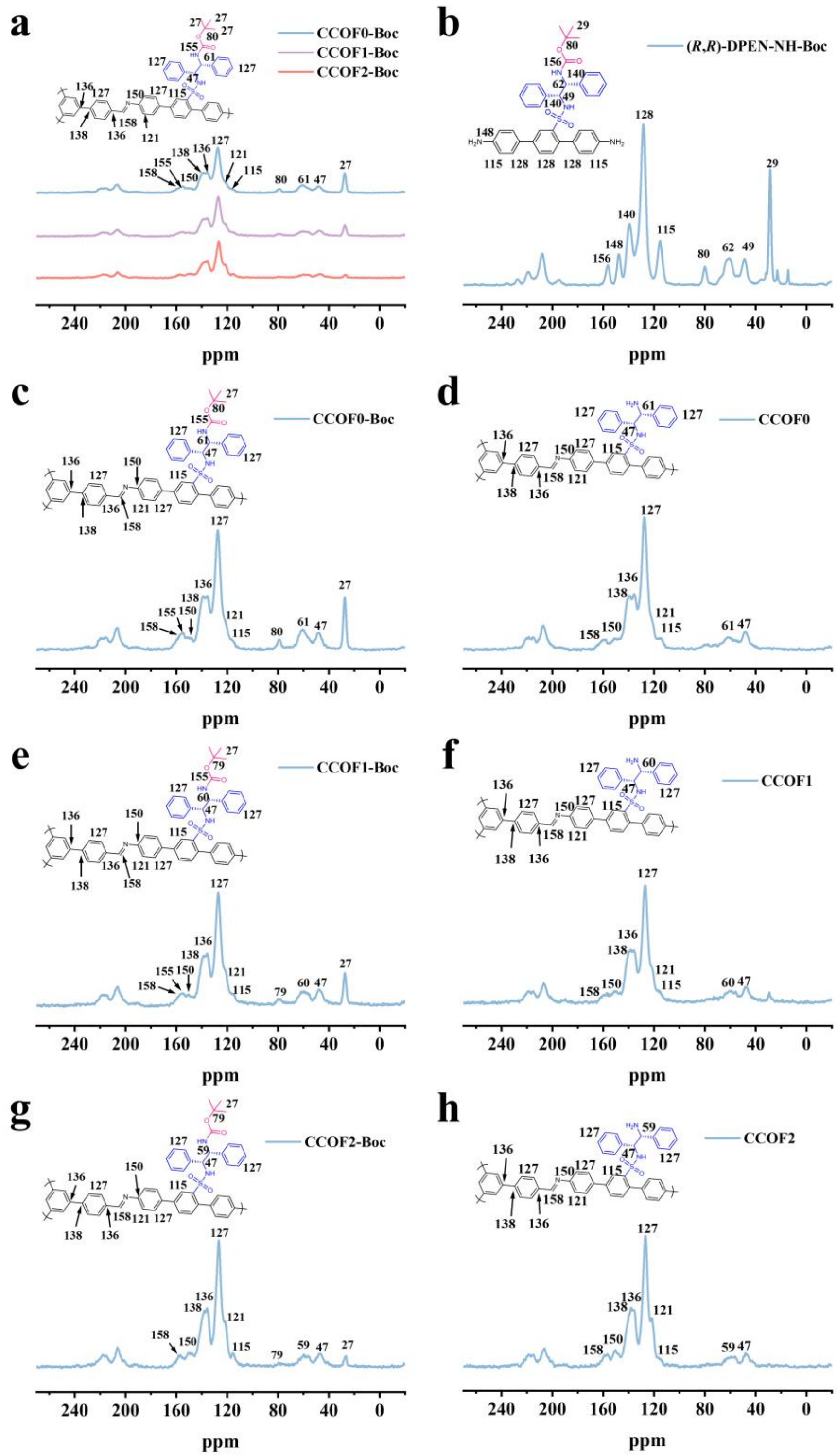

Supplementary Fig. 3 solid-state ^{13}C CP/MAS NMR spectra of the chiral monomer and COF samples. **a** Stacked comparison of the Boc-protected CCOFs, **b** (*R*,*R*)-DPEN-NH-Boc, **c** CCOF0-Boc, **d** CCOF0, **e** CCOF1-Boc, **f** CCOF1, **g** CCOF2-Boc and **h** CCOF2. The resonances at

approximately 156, 80, and 29 ppm are assigned to the Boc carbamate carbonyl, quaternary carbon, and tert-butyl methyl carbons, respectively, whereas those at approximately 62 and 49 ppm are associated primarily with the stereogenic carbons of the DPEN-derived unit. Following deprotection, the Boc-associated resonances are substantially attenuated or no longer clearly resolved, whereas the DPEN-derived signals remain, with some broadening and small chemical-shift changes. In CCOF2-Boc, the Boc carbamate-carbonyl resonance is not resolved as a separate signal, likely because of overlap with nearby framework resonances.

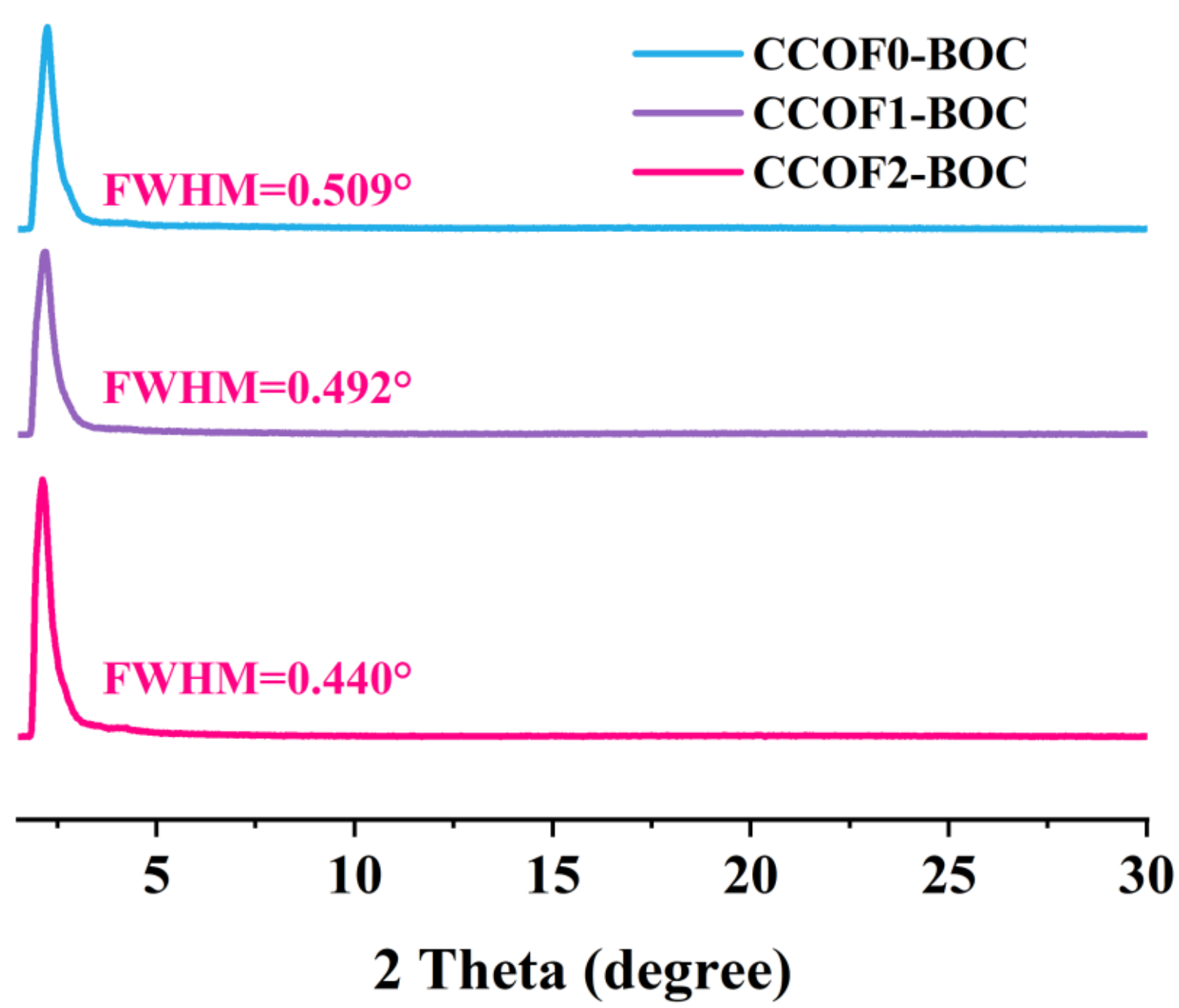

Supplementary Fig. 4 PXRD patterns of CCOF0-Boc, CCOF1-Boc and CCOF2-Boc. FWHM, full width at half maximum.

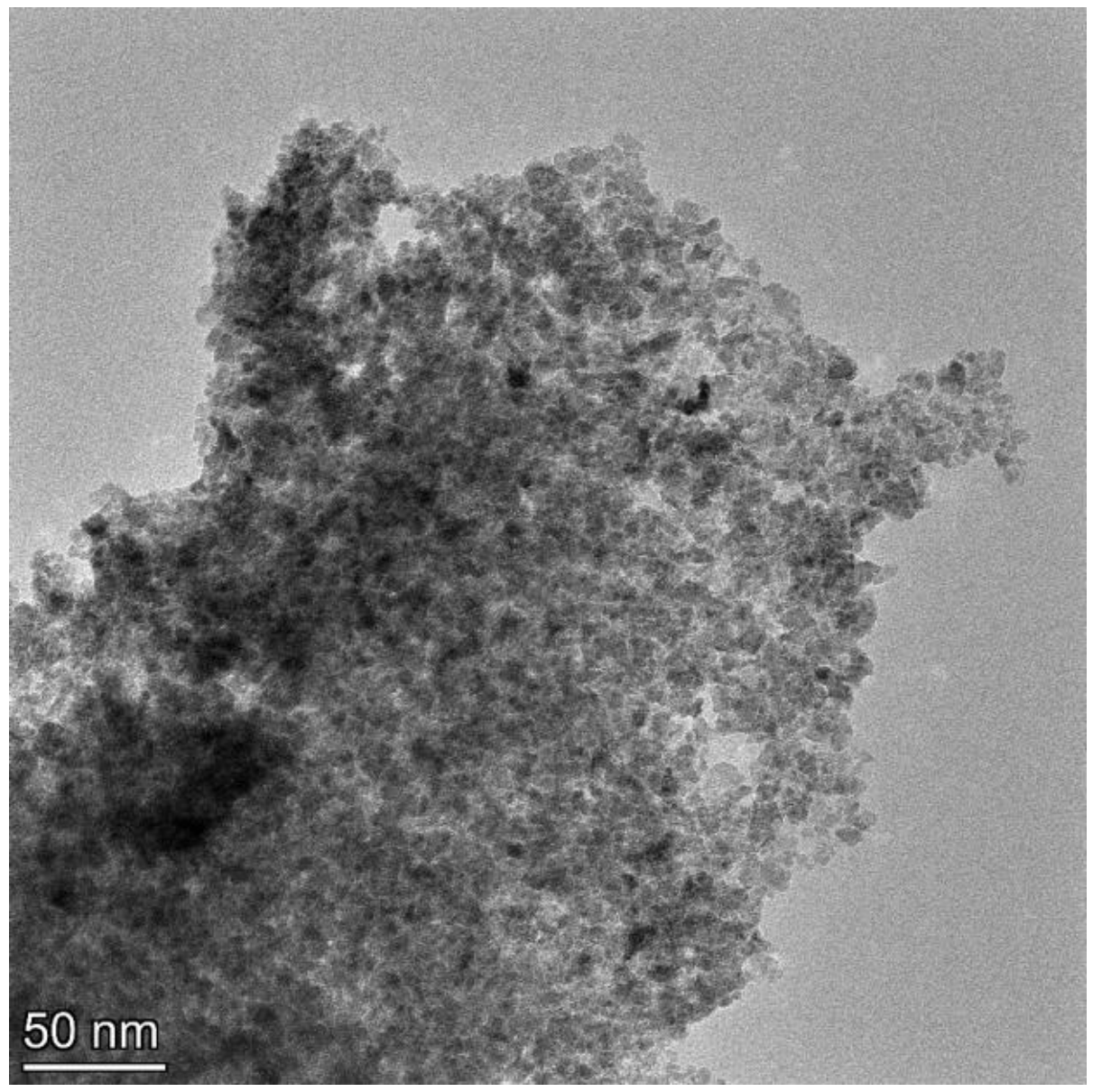

Supplementary Fig. 5 TEM images of CCOF0- Boc. TEM images further revealed that CCOF0- Boc composed of smaller anisotropic structures. When these particles are deposited onto the XRD holder or TEM grid, they tend to lie flat. Consequently, the (100) planes are oriented parallel to the substrate, resulting in a strongly enhanced (100) reflection in XRD (preferred orientation/texturing). However, because these same (100) planes are perpendicular to the vertical electron beam in TEM, their lattice fringes are geometrically difficult to capture unless the particle is specifically tilted. Instead, the (001) planes (which are orthogonal to the substrate) align perfectly with the electron beam, making the (001) fringes (0.48 nm) readily visible in HRTEM (Fig. 2e). CCOF0-Boc has relatively lower crystallinity compared to CCOF1/2-Boc. In COF structures, the (100) planes represent the porous and fragile in-plane channel structures, whereas the (001) planes represent the dense, strongly interacting p-p stacking layers. Under high-energy electron beam irradiation, the porous (100) channels of the less crystalline CCOF0-Boc are susceptible to rapid structural degradation (knock-on damage). While we did sporadically observe faint, larger-spacing (100) fringes locally, they amorphized too quickly to capture high-quality images. The dense (001) stacking, however, is significantly more robust against beam damage,

allowing for clear observation. To ensure absolute data reliability, we only annotated the unambiguous (001) fringes.

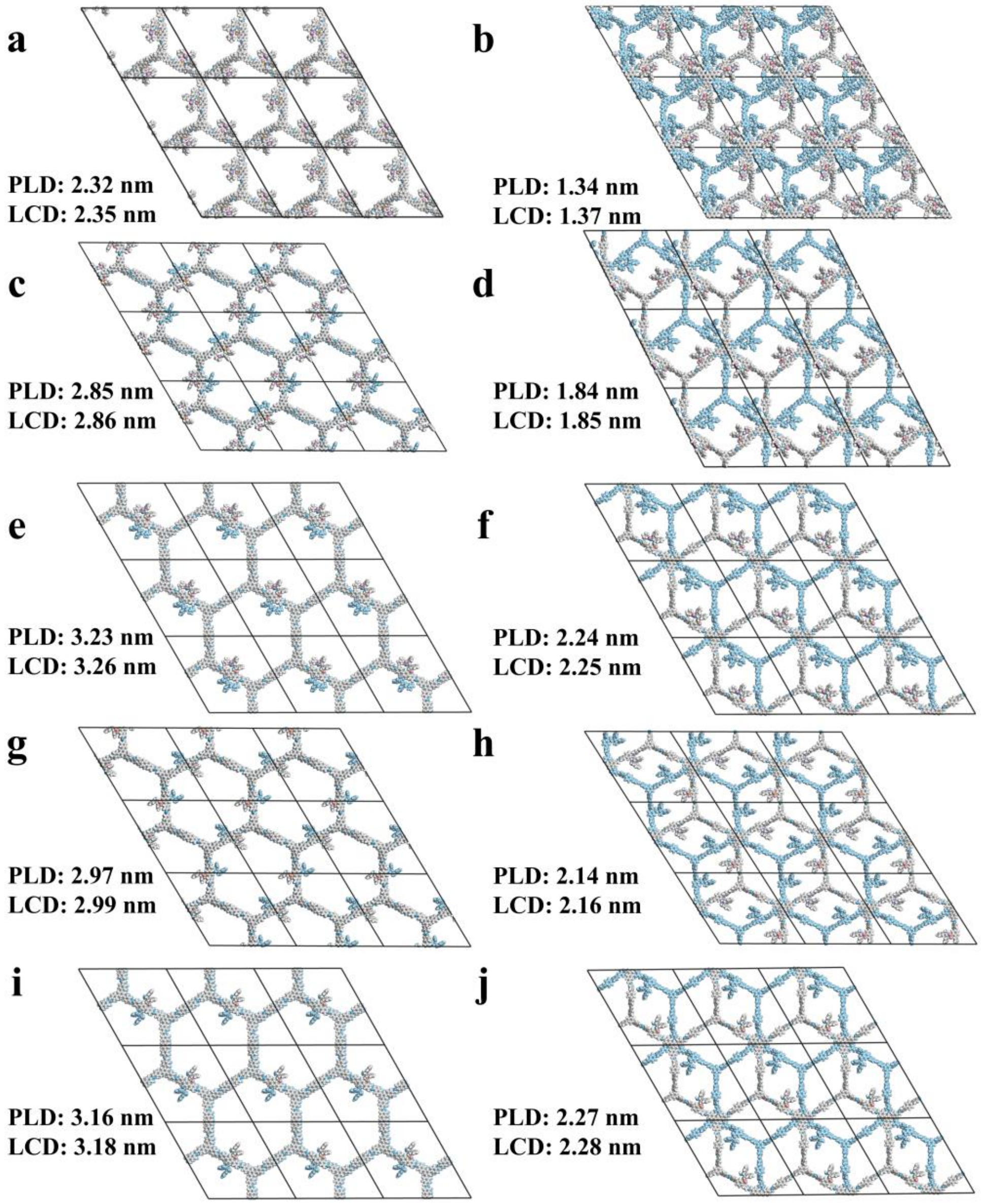

Supplementary Fig. 6 Optimized AA-eclipsed structural models of **a** CCOF0-Boc, **c** CCOF1-Boc, **e** CCOF2-Boc, **g** CCOF1, and **i** CCOF2, and AB-staggered structural models of **b** CCOF0-Boc, **d** CCOF1-Boc, **f** CCOF2-Boc, **h** CCOF1, and **j** CCOF2. The pore limiting diameter (PLD) and the largest cavity diameter (LCD) were calculated using ZEO++. For all CCOFs, the PLD and LCD values of the AA-eclipsed models match the experimentally derived pore-size distributions

from N_2 sorption (Supplementary Fig. 7) better than those of the AB-staggered models, suggesting that these CCOFs are more likely to adopt AA-eclipsed stacking. Color code: gray, C; blue, N; red, O; yellow, S; white, H.

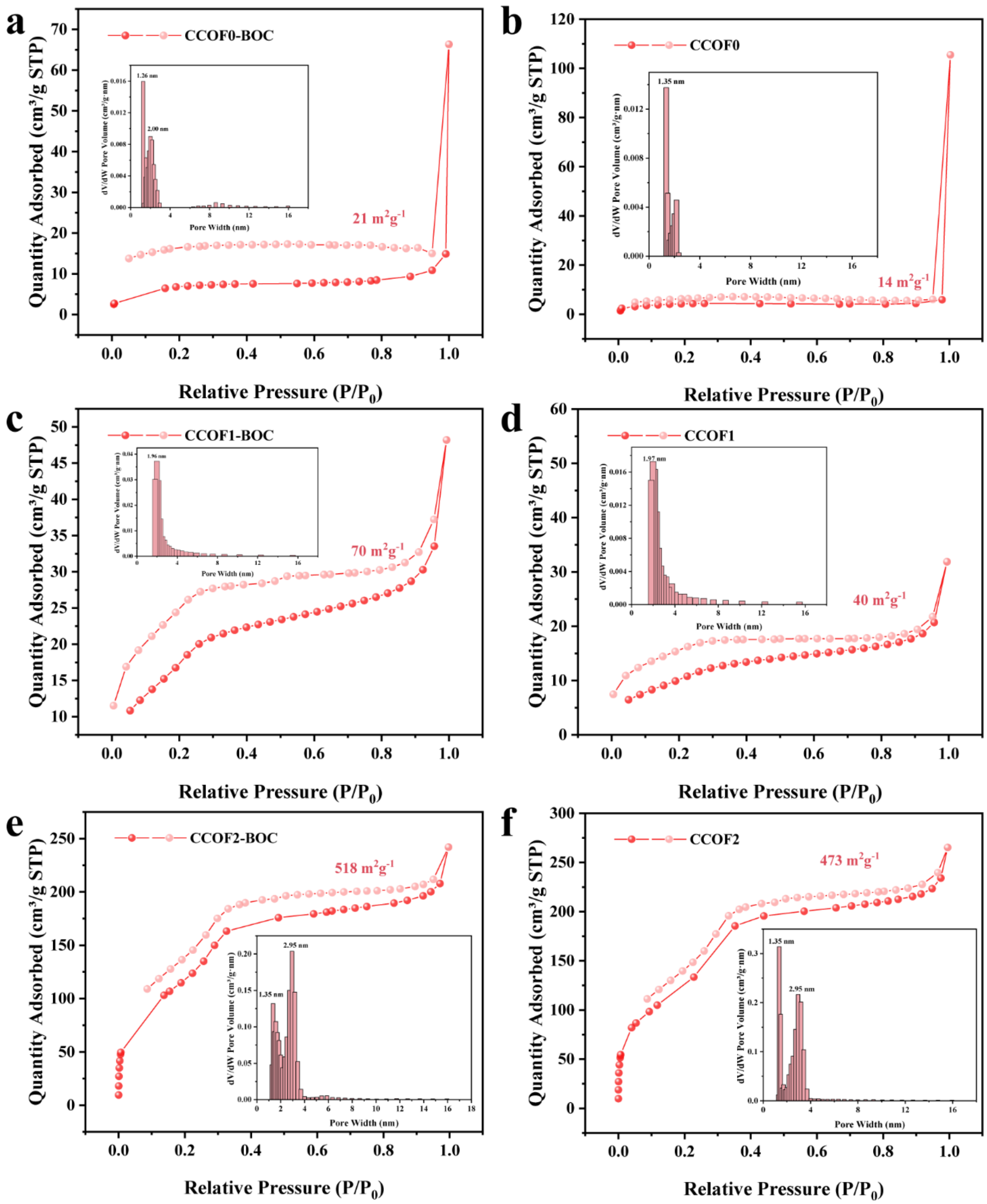

Supplementary Fig. 7 Nitrogen adsorption/desorption isotherms of **a** CCOF0-Boc, **b** CCOF0, **c** CCOF1-Boc, **d** CCOF1, **e** CCOF2-Boc, and **f** CCOF2 measured at 77 K. Corresponding pore size distributions are shown in the inset.

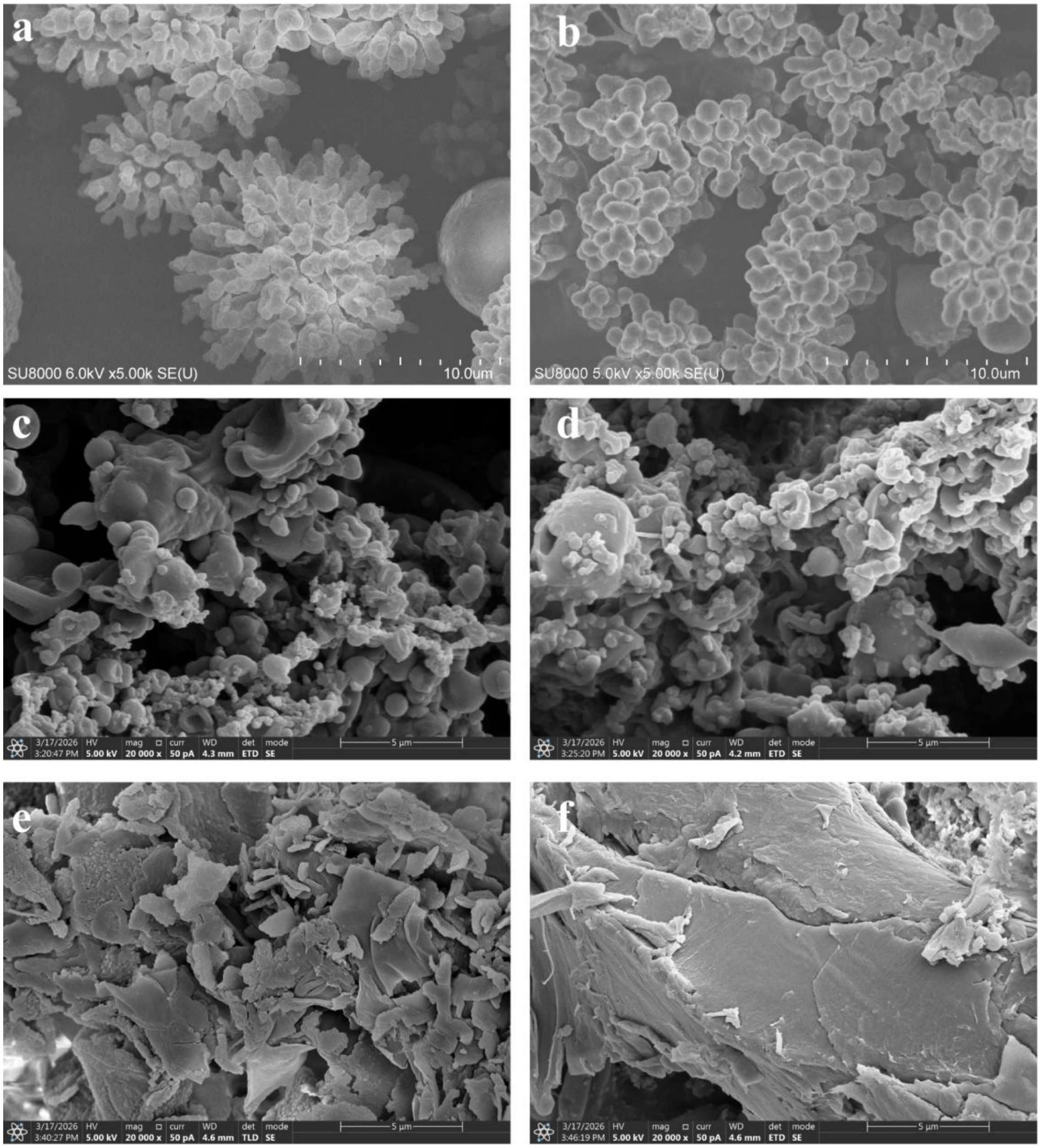

Supplementary Fig. 8 SEM images of **a** CCOF0-Boc, **b** CCOF0, **c** CCOF1-Boc, **d** CCOF1, **e** CCOF2-Boc and **f** CCOF2.

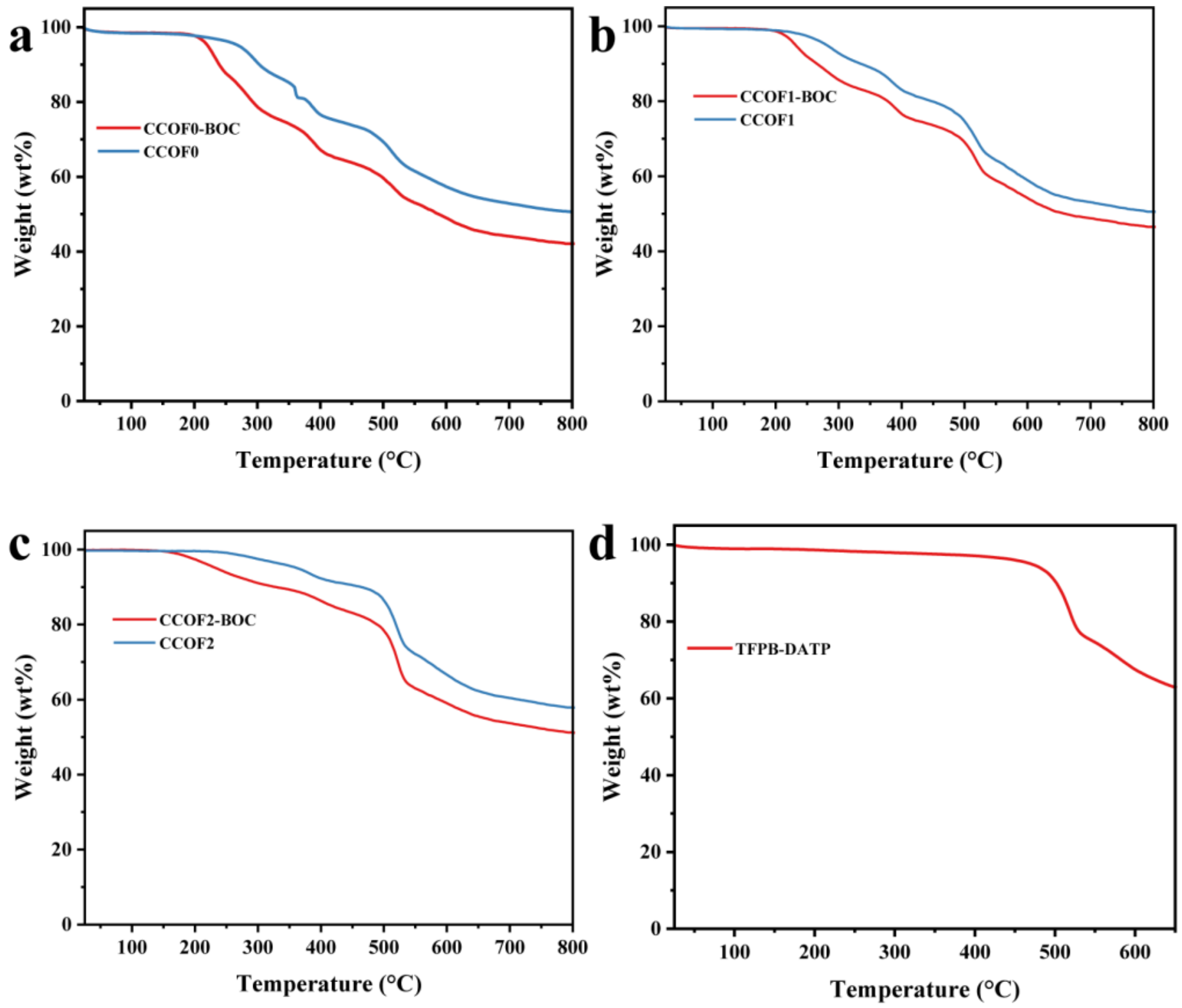

Supplementary Fig. 9 TGA curves of **a** CCOF0-Boc and CCOF0, **b** CCOF1-Boc and CCOF1, **c** CCOF2-Boc and CCOF2, and **d** TFPB-DATP. The additional mass-loss feature beginning near approximately 210 °C in the Boc-protected samples is assigned to decomposition of the Boc-containing groups based on comparison with the corresponding deprotected samples. The mass-loss region near approximately 275 °C is tentatively associated with decomposition of the chiral sulfonamide-containing component. The substantial higher-temperature mass loss is consistent with framework decomposition and carbonization. These assignments are tentative because evolved-gas analysis was not performed.

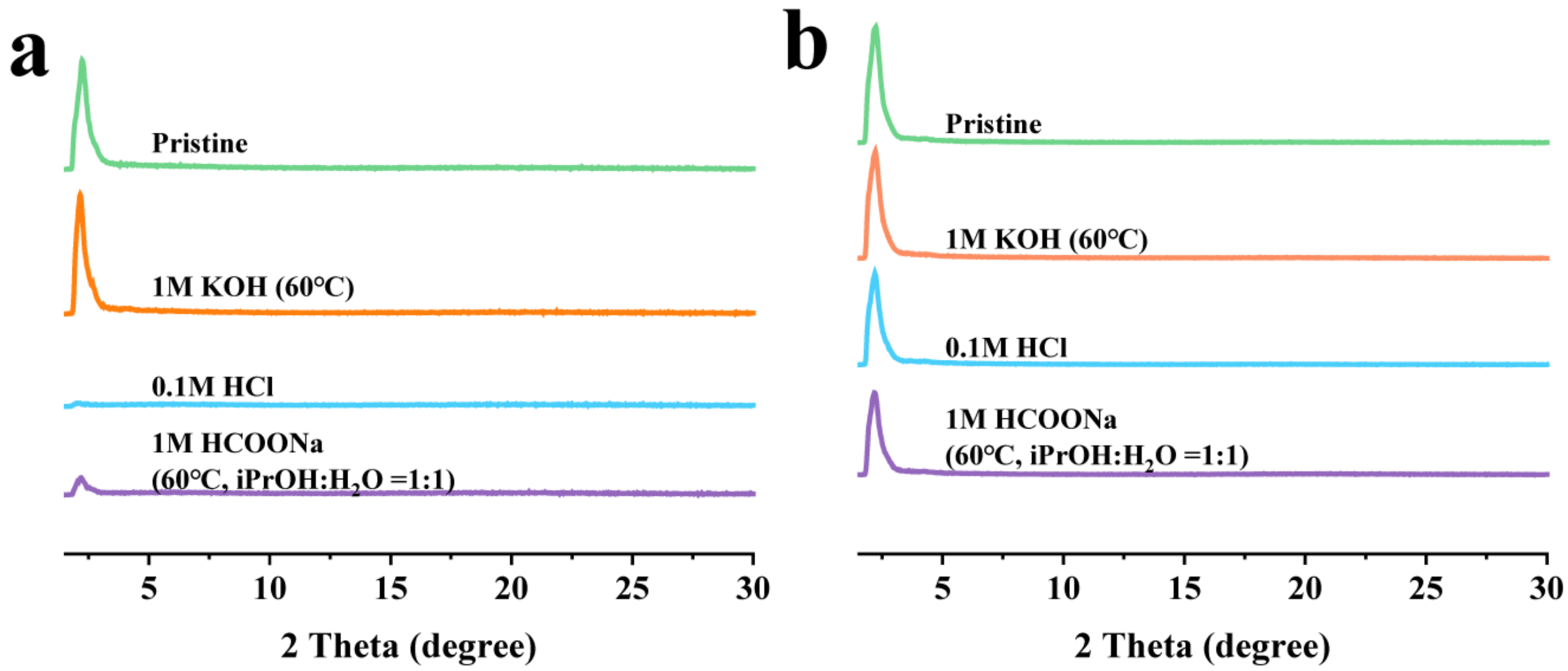

Supplementary Fig. 10 PXRD patterns of **a** CCOF1 and **b** CCOF2 after immersion in different solvents for 48 h. (After solvent treatment, the sample was sequentially washed with deionized water and ethanol, purified via acetone Soxhlet extraction for 12 h, and finally dried under vacuum at 80 °C for 12 h.)

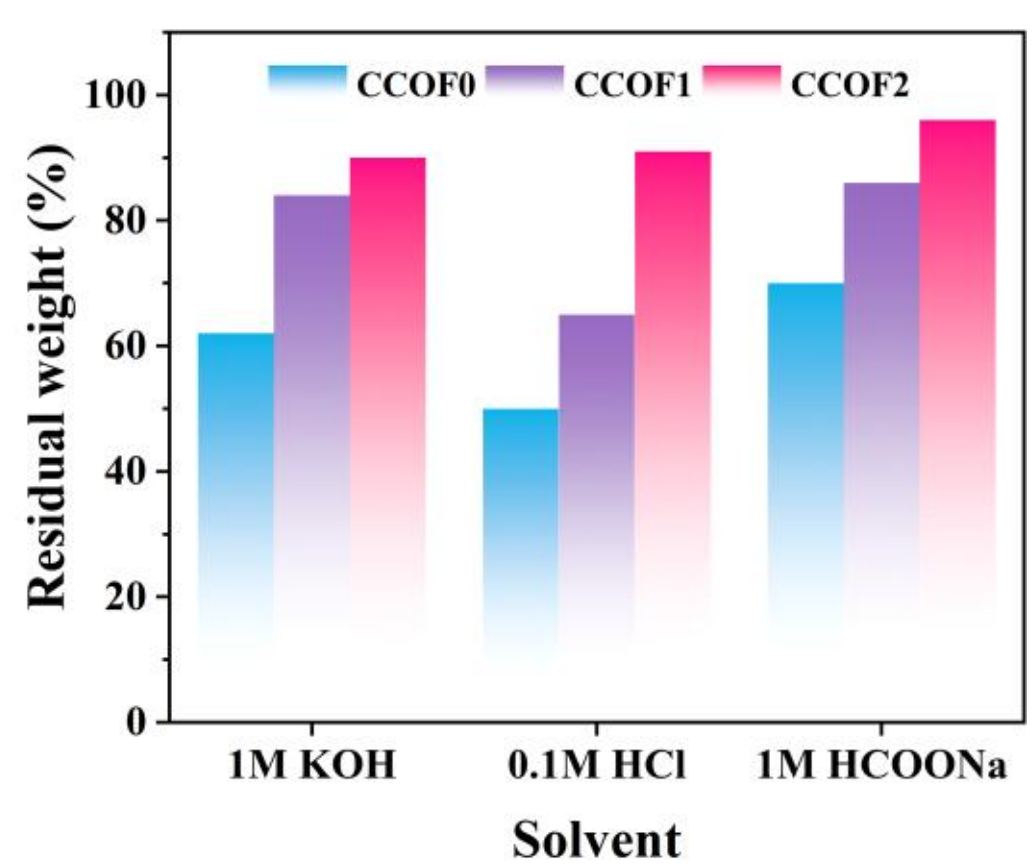

Supplementary Fig. 11 Residual weights of CCOFX after immersion in different solvents for 48 h.

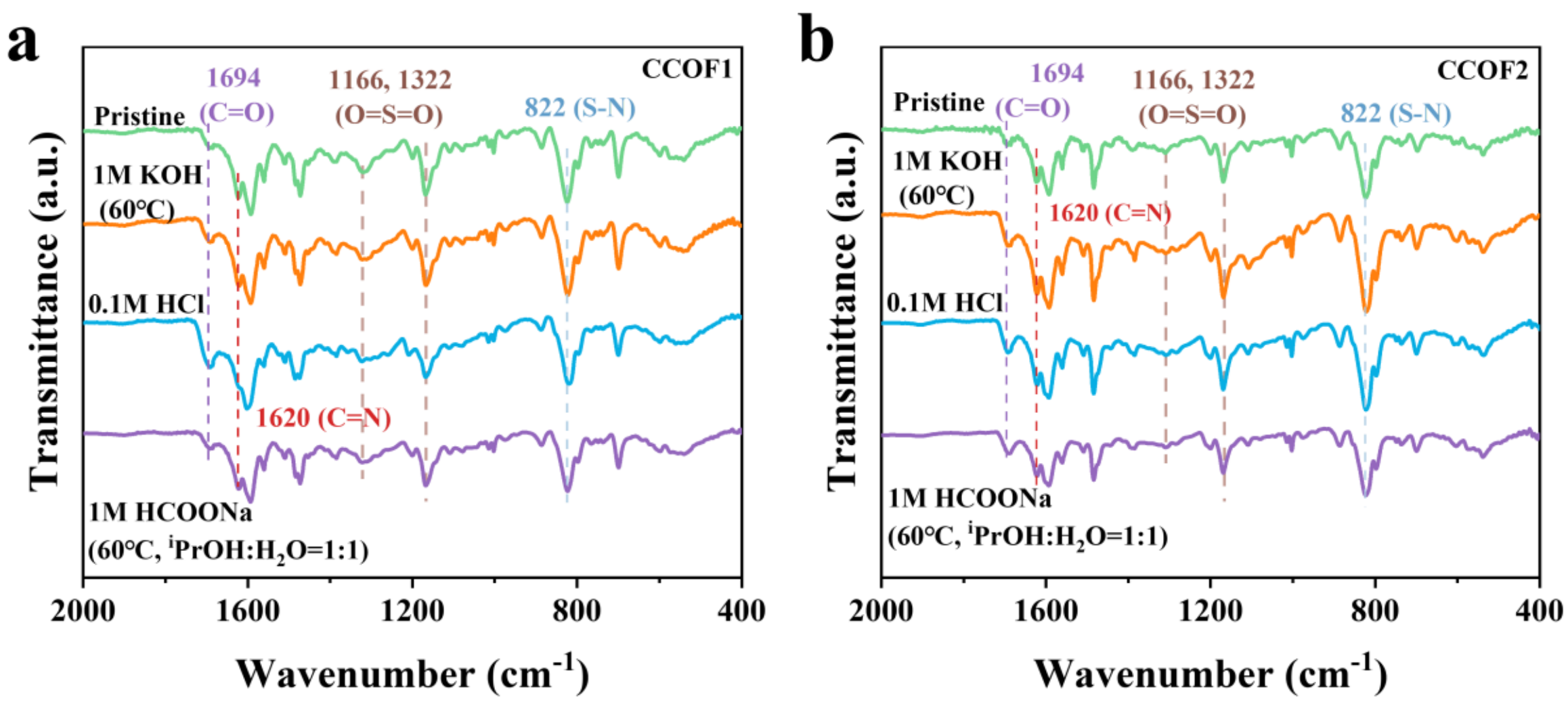

Supplementary Fig. 12 FTIR spectra of **a** CCOF1 and **b** CCOF2 after immersion in different solvents for 48 h. (After solvent treatment, the sample was sequentially washed with deionized water and ethanol, purified via acetone Soxhlet extraction for 12 h, and finally dried under vacuum at 80 °C for 12 h.)

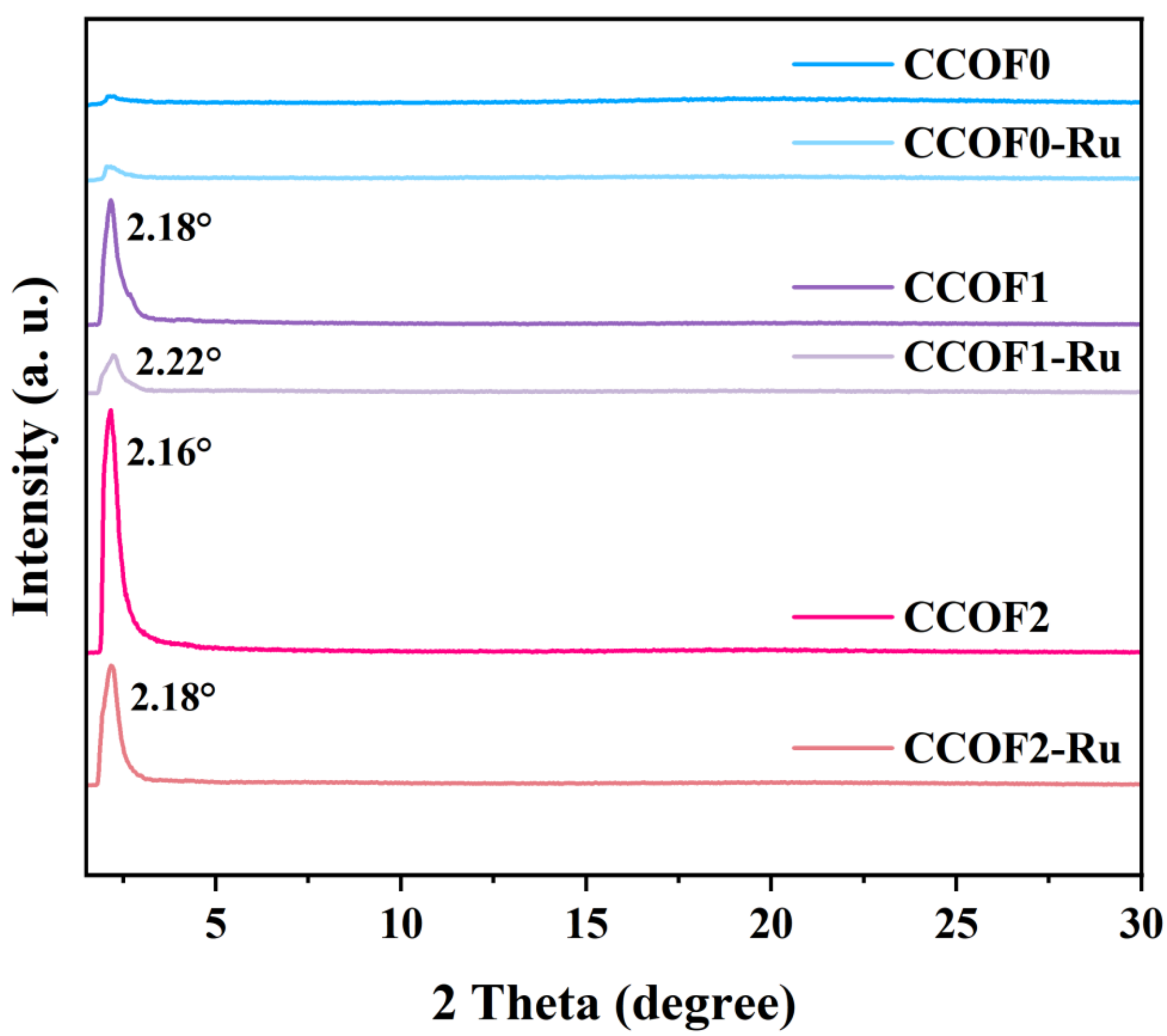

Supplementary Fig. 13 PXRD patterns of the CCOFX and CCOFX-Ru.

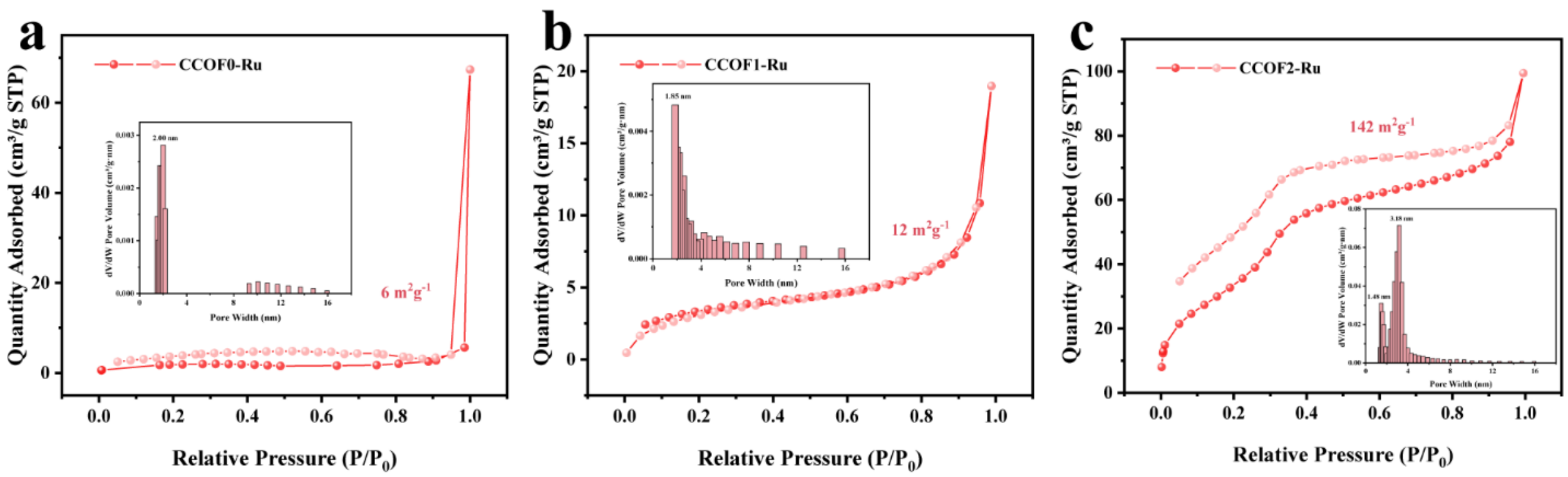

Supplementary Fig. 14 Nitrogen adsorption/desorption isotherms of **a** CCOF0-Ru, **b** CCOF1-Ru, and **c** CCOF2- Ru measured at 77 K. Corresponding pore size distributions are shown in the inset.

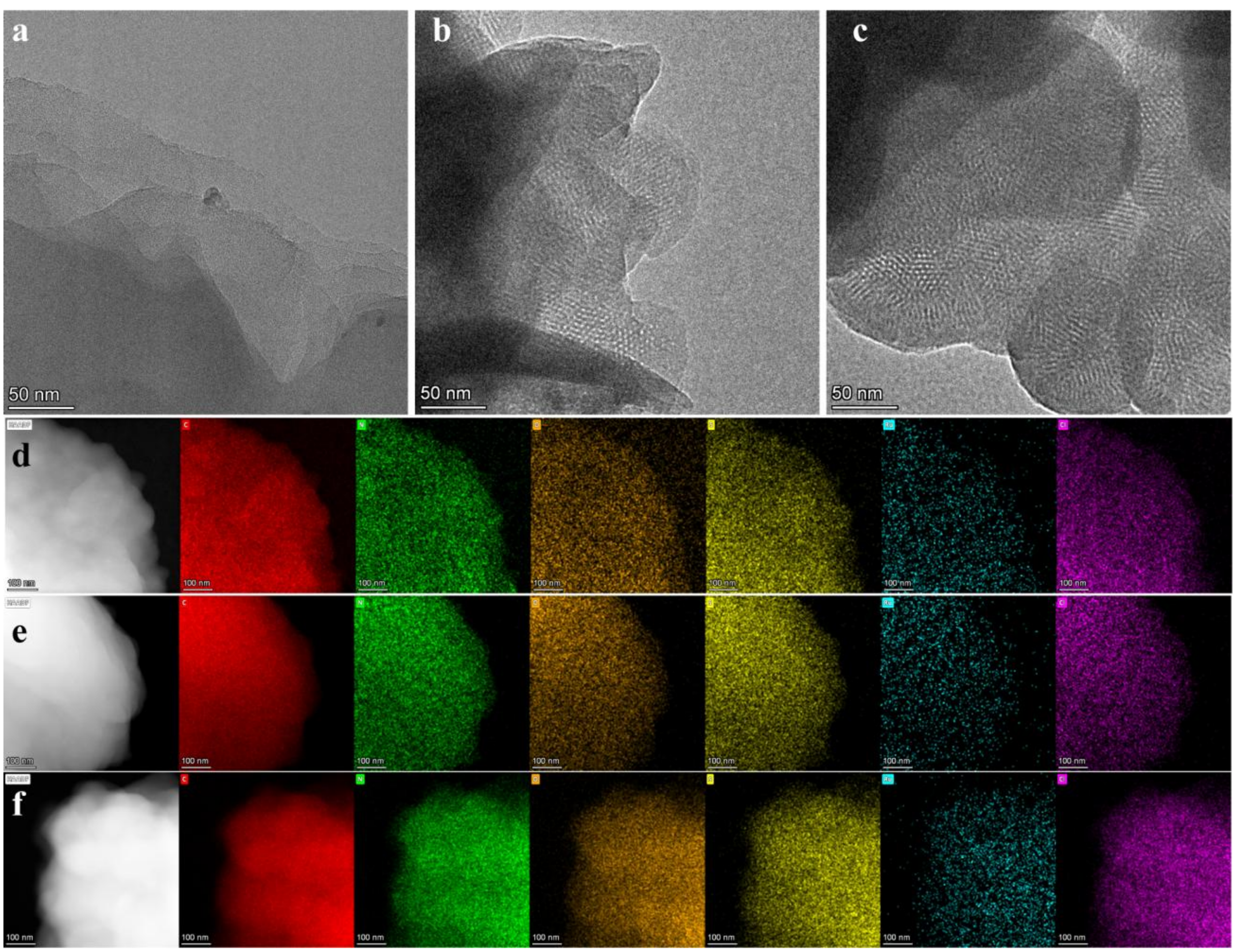

Supplementary Fig. 15 TEM image of **a** CCOF0-Ru, **b** CCOF1-Ru and **c** CCOF2-Ru. HAADF image and TEM-EDS mapping of **d** CCOF0-Ru**, e** CCOF1-Ru and **f** CCOF2-Ru.

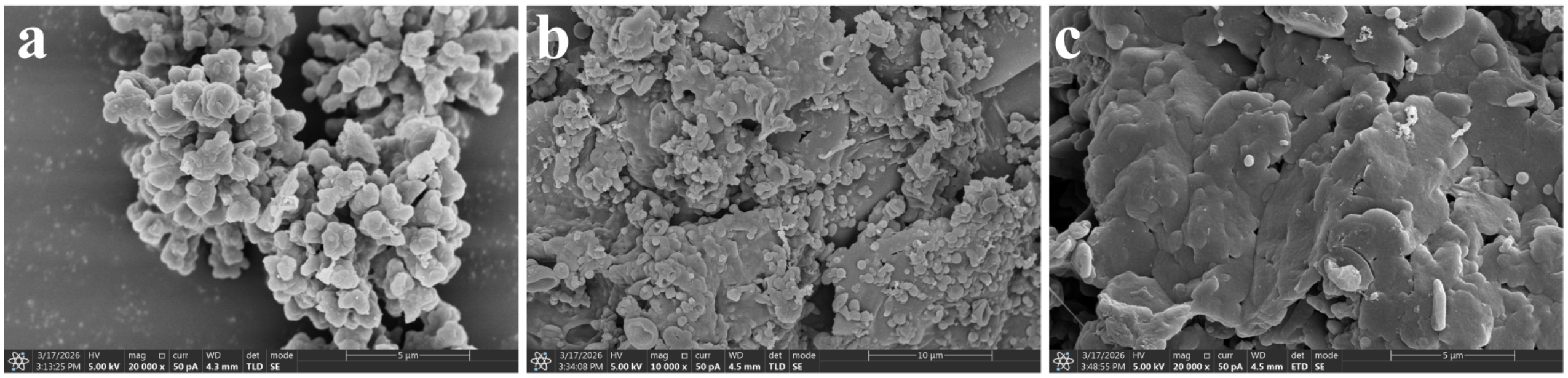

Supplementary Fig. 16 SEM image of **a** CCOF0-Ru, **b** CCOF1-Ru and **c** CCOF2-Ru.

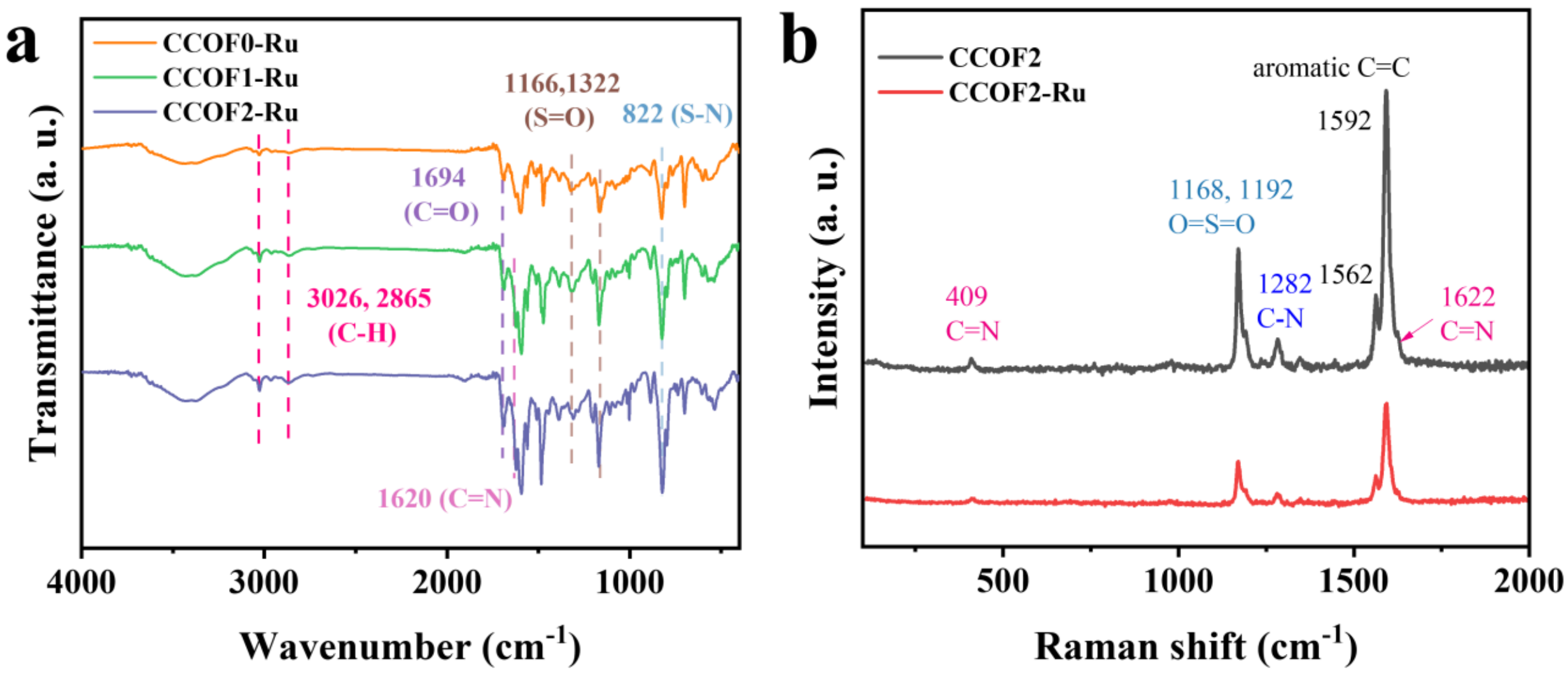

Supplementary Fig. 17 a FTIR spectra of CCOFX-Ru, **b** Raman spectra of CCOF2 and CCOF2-Ru. The peaks at 1694 cm^{-1} in the FTIR spectra is attributed to terminal aldehydes.

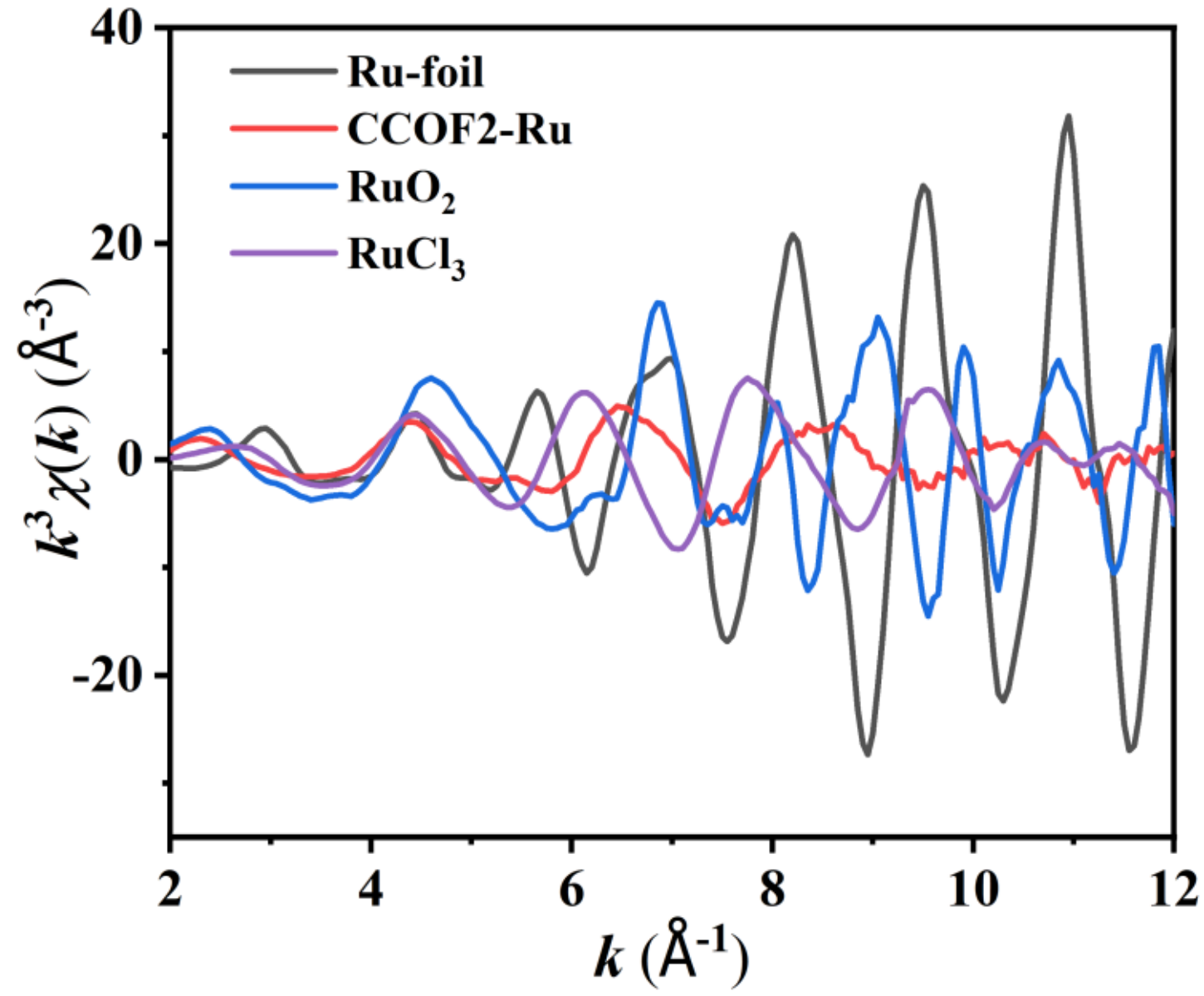

Supplementary Fig. 18 CCOF2-Ru and standard referenced $k^3\chi(k)$ oscillation curve.

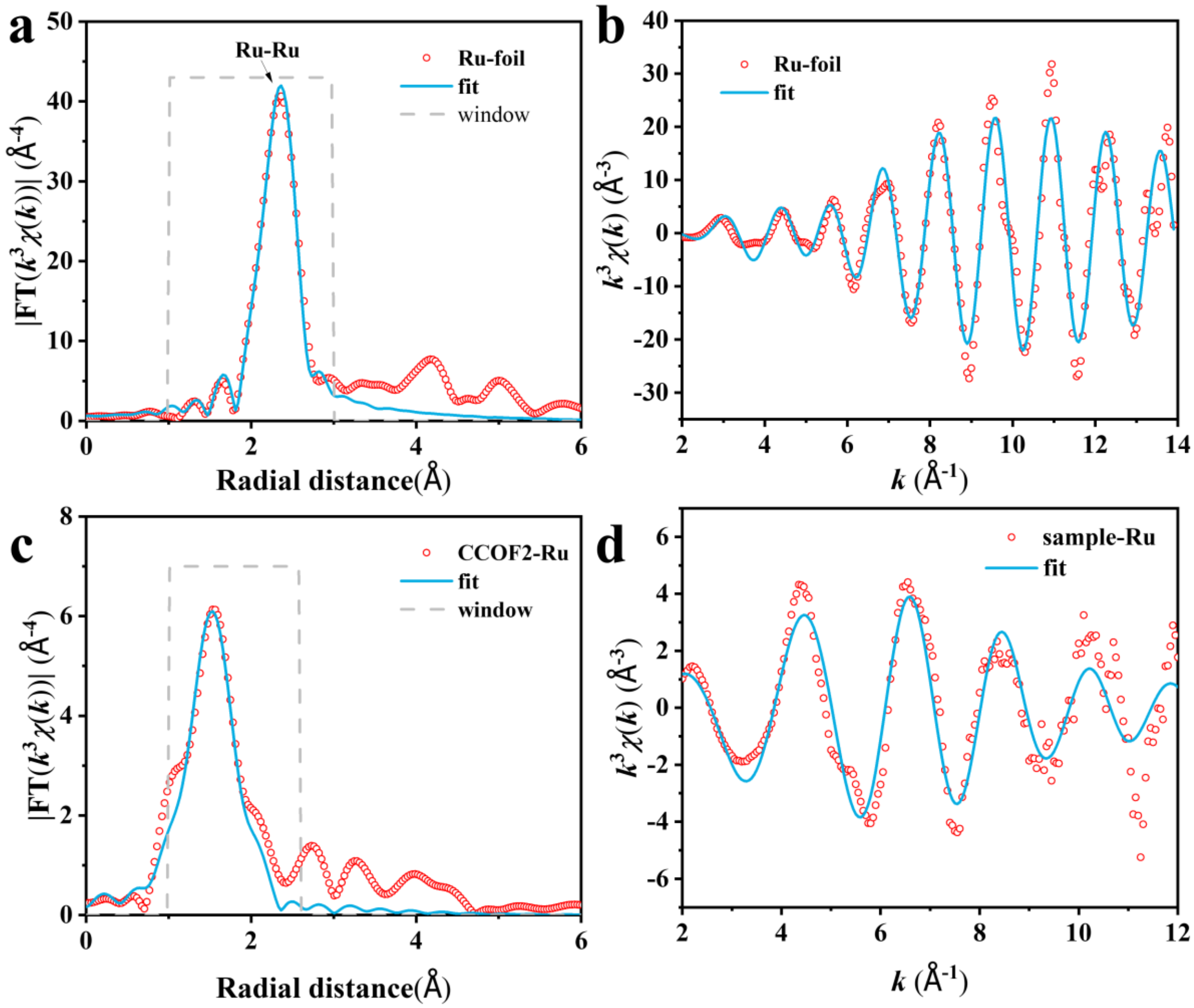

Supplementary Fig. 19 The fitted Fourier transform R-spaced extended X-ray absorption fine spectra (FT-EXAFS) of **a** Ru foil and **c** CCOF2-Ru. The fitted $k^3\chi(k)$ oscillation curve of **b** Ru foil and **d** CCOF2-Ru.

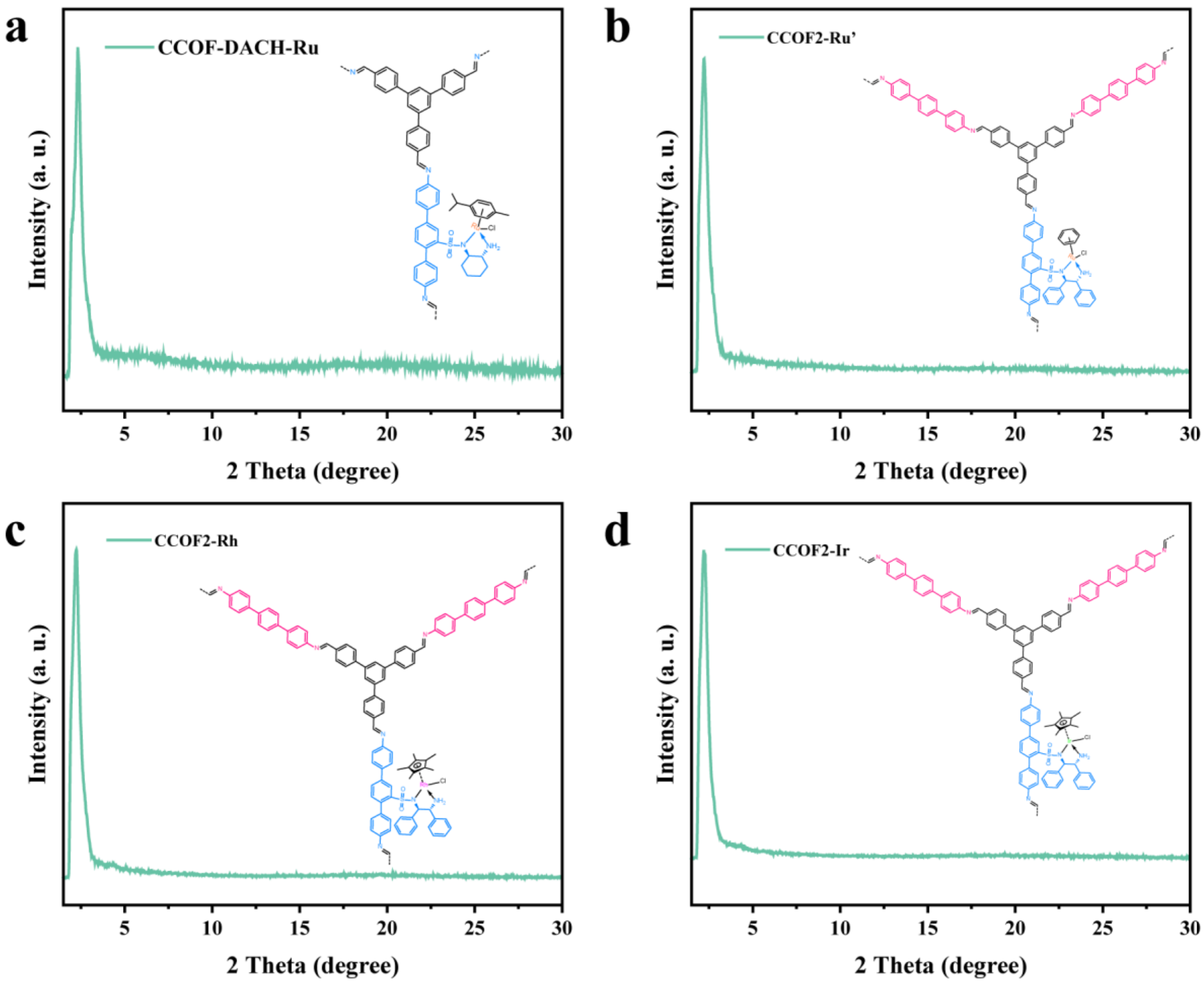

Supplementary Fig. 20 PXRD patterns of **a** CCOF-DACH-Ru, **b** CCOF2-Ru', **c** CCOF2-Rh and **d** CCOF2-Ir.

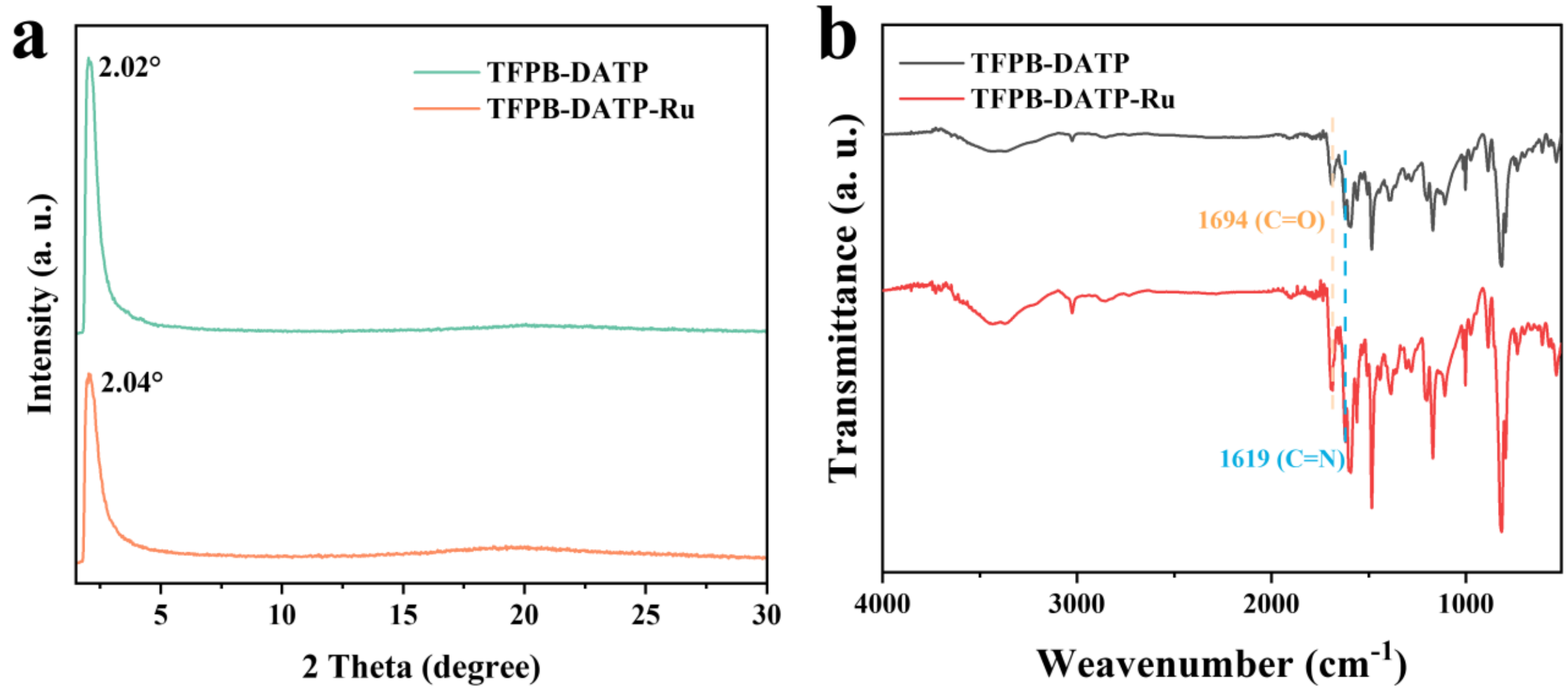

Supplementary Fig. 21 a PXRD patterns and **b** FTIR spectra of TFPB-DATP and TFPB-DATP-Ru. Supplementary Fig. 21 shows the PXRD patterns and FTIR spectra of TFPB-DATP before and after Ru coordination. The absence of Ru cluster or oxide peaks in PXRD confirms the high dispersion of the trace Ru species and the preservation of framework crystallinity.

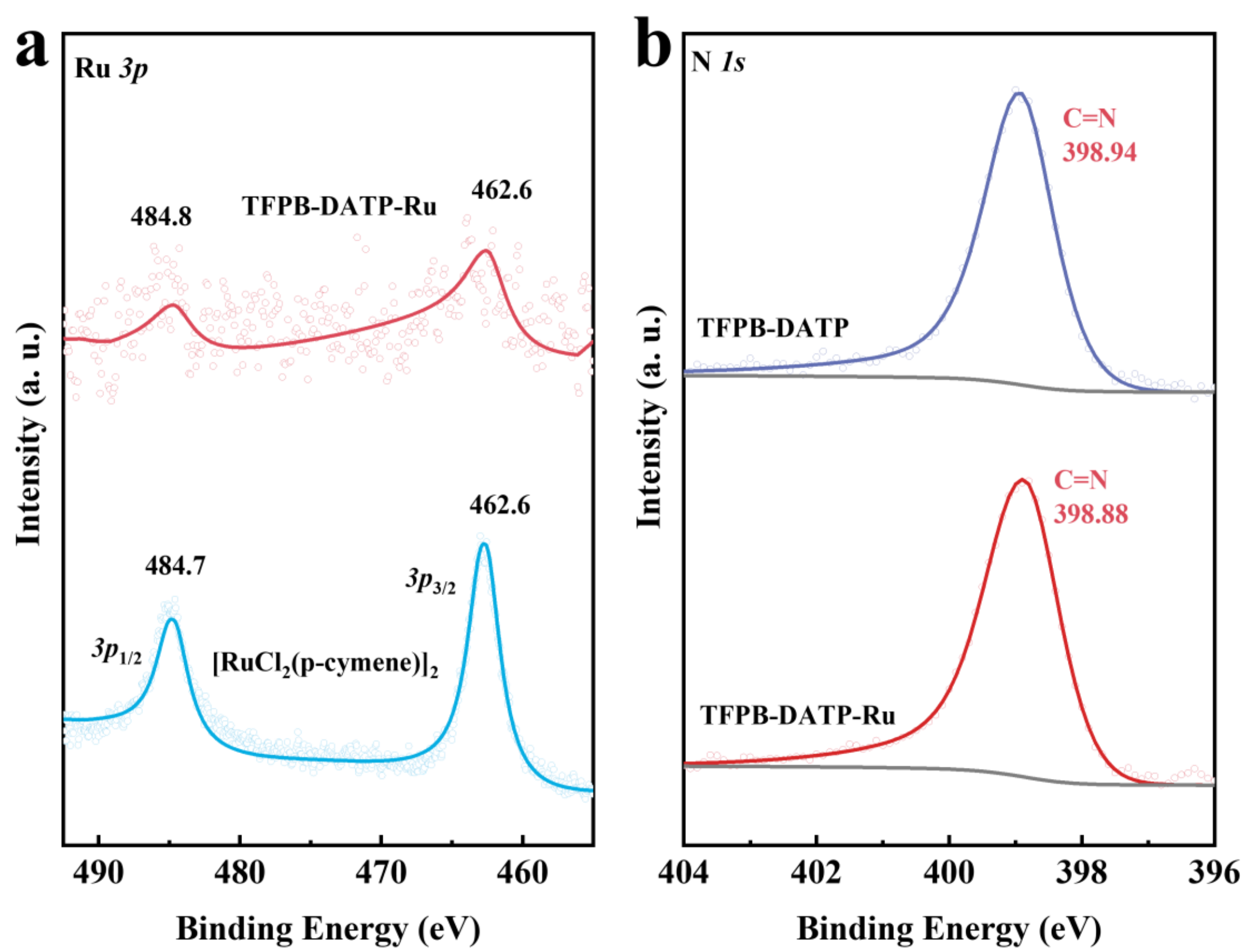

Supplementary Fig. 22 XPS spectra: **a** Ru 3p, **b** N 1s for TFPB-DATP-Ru. A slight shift of the imine C=N peak is observed in the N 1s spectrum of TPB-DATP-Ru, indicating the possible coordination of Ru to C=N.

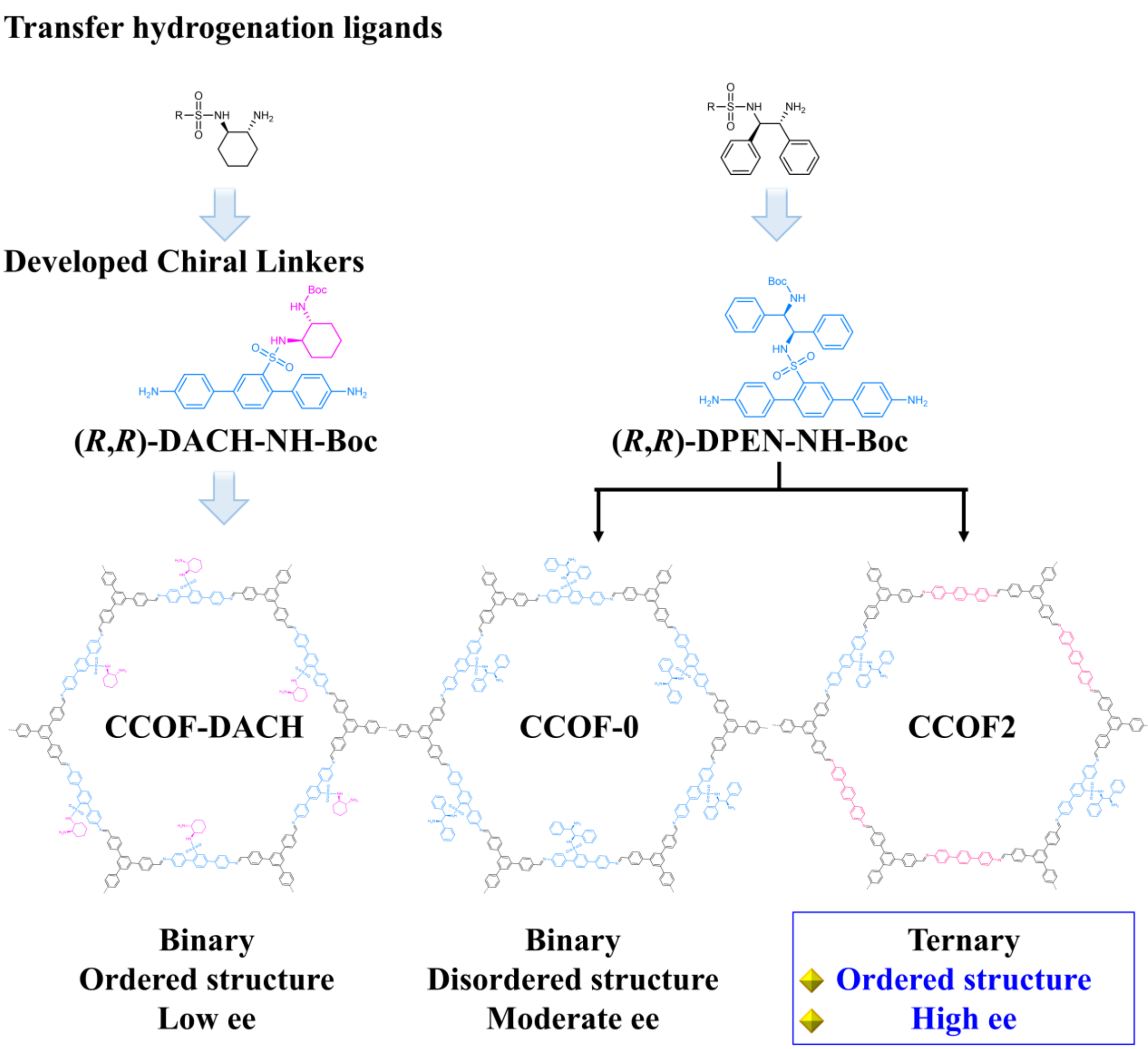

Supplementary Fig. 23 Design of Noyori-Inspired CCOFs.

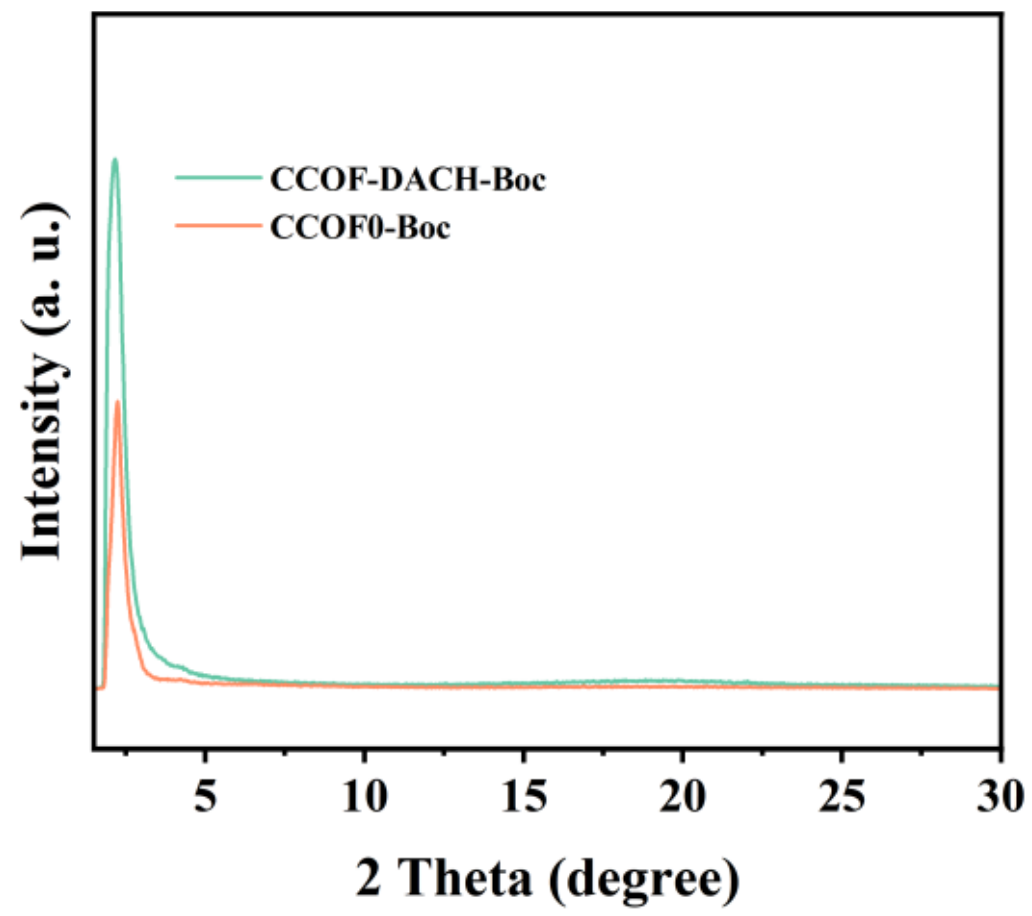

Supplementary Fig. 24 PXRD patterns of Boc-protected CCOF0-Boc and CCOF-DACH-Boc synthesized at 100 °C. The patterns reveal that CCOF-DACH-Boc exhibits significantly higher crystallinity than CCOF0-Boc. Both frameworks share the same topology and were prepared under identical conditions, including the same Boc group concentration and a reaction temperature of 100 °C. Hence, the lower crystallinity of CCOF0-Boc is more likely attributable to greater steric hindrance from its substituents, which impairs COF crystallinity.

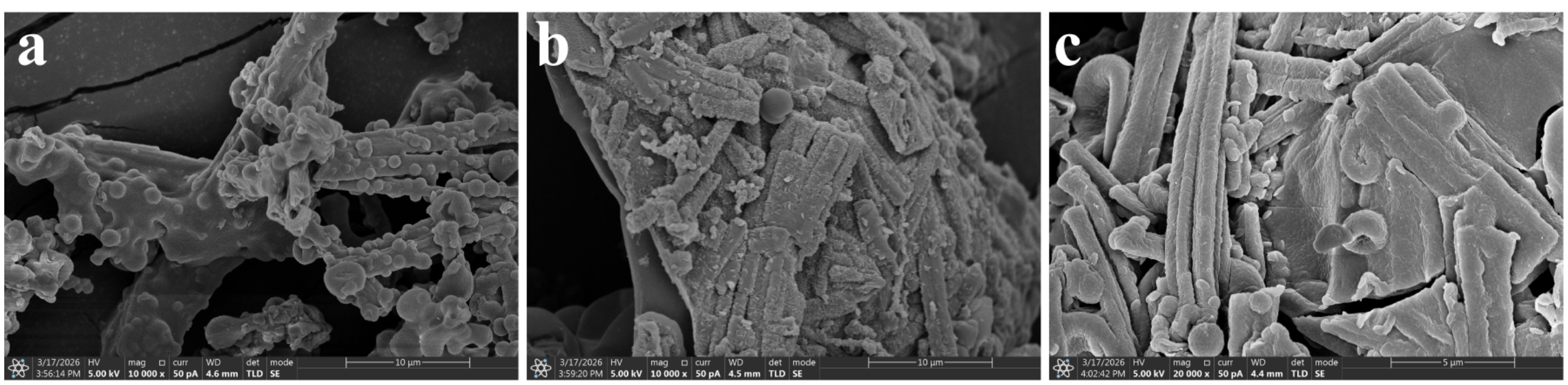

Supplementary Fig. 25 SEM image of **a** CCOF-DACH-Boc, **b** CCOF-DACH and **c** CCOF-DACH-Ru.

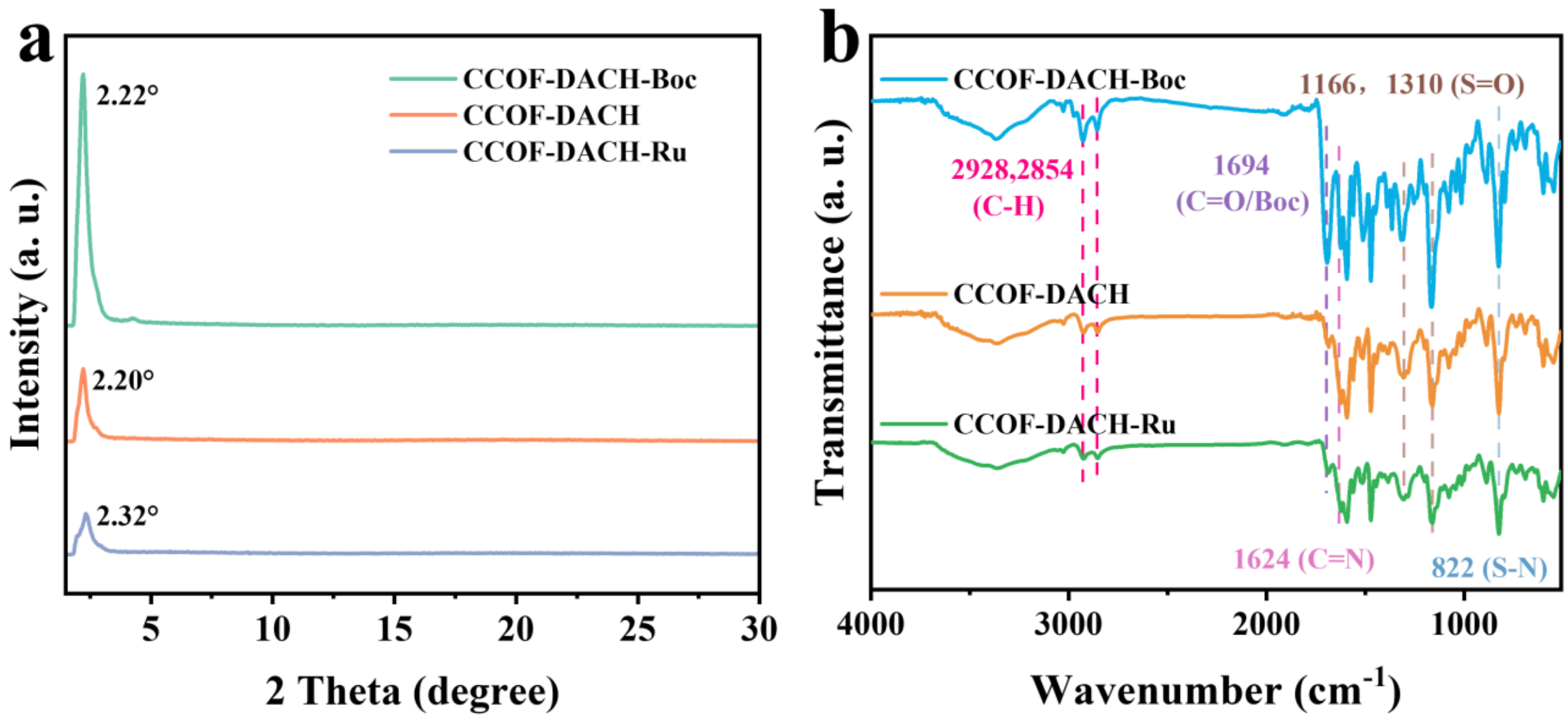

Supplementary Fig. 26 a PXRD patterns and **b** FTIR spectra of CCOF-DACH-Boc, CCOF-DACH,and CCOF-DACH-Ru.

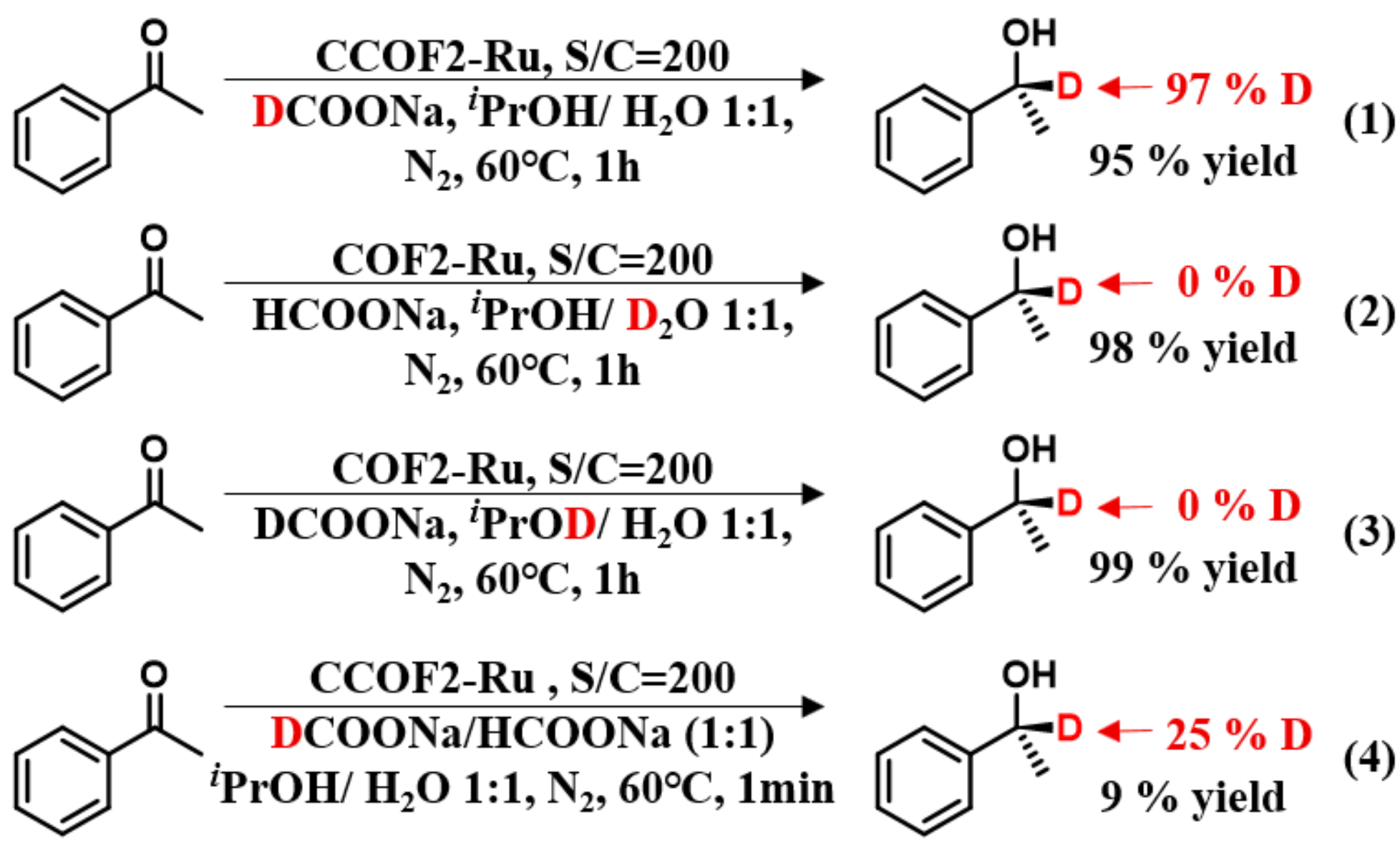

Supplementary Fig. 27 Deuterium-Labeling Studies.

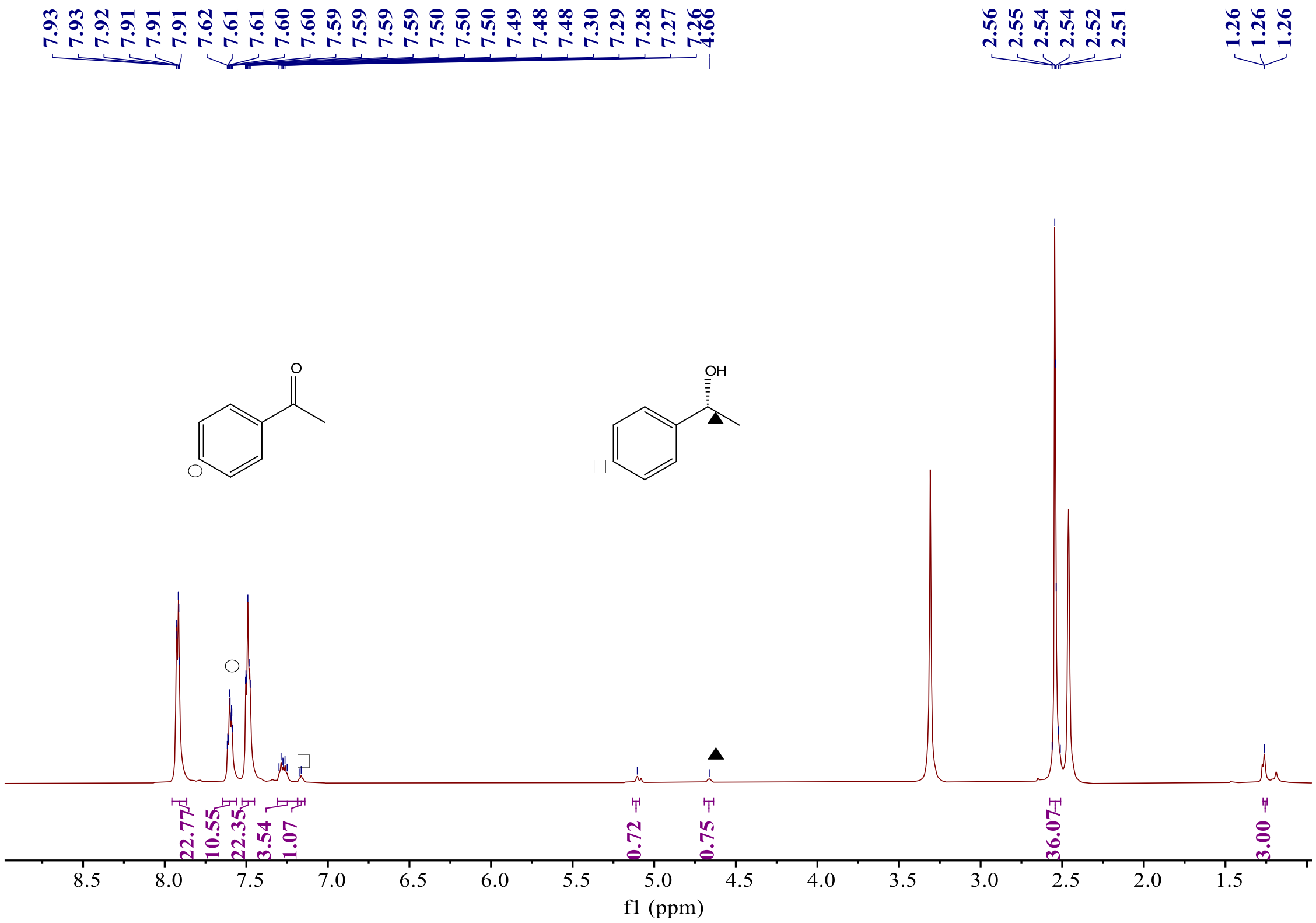

Supplementary Fig. 28 ^{1}H NMR (600 MHz, DMSO-D_6) spectra of deuterium-labeling KIE studies. At a conversion of <10%, the deuterium incorporation in the product was determined to be 25%, corresponding to a kinetic isotope effect of $k_H/k_D \approx 3.0$.

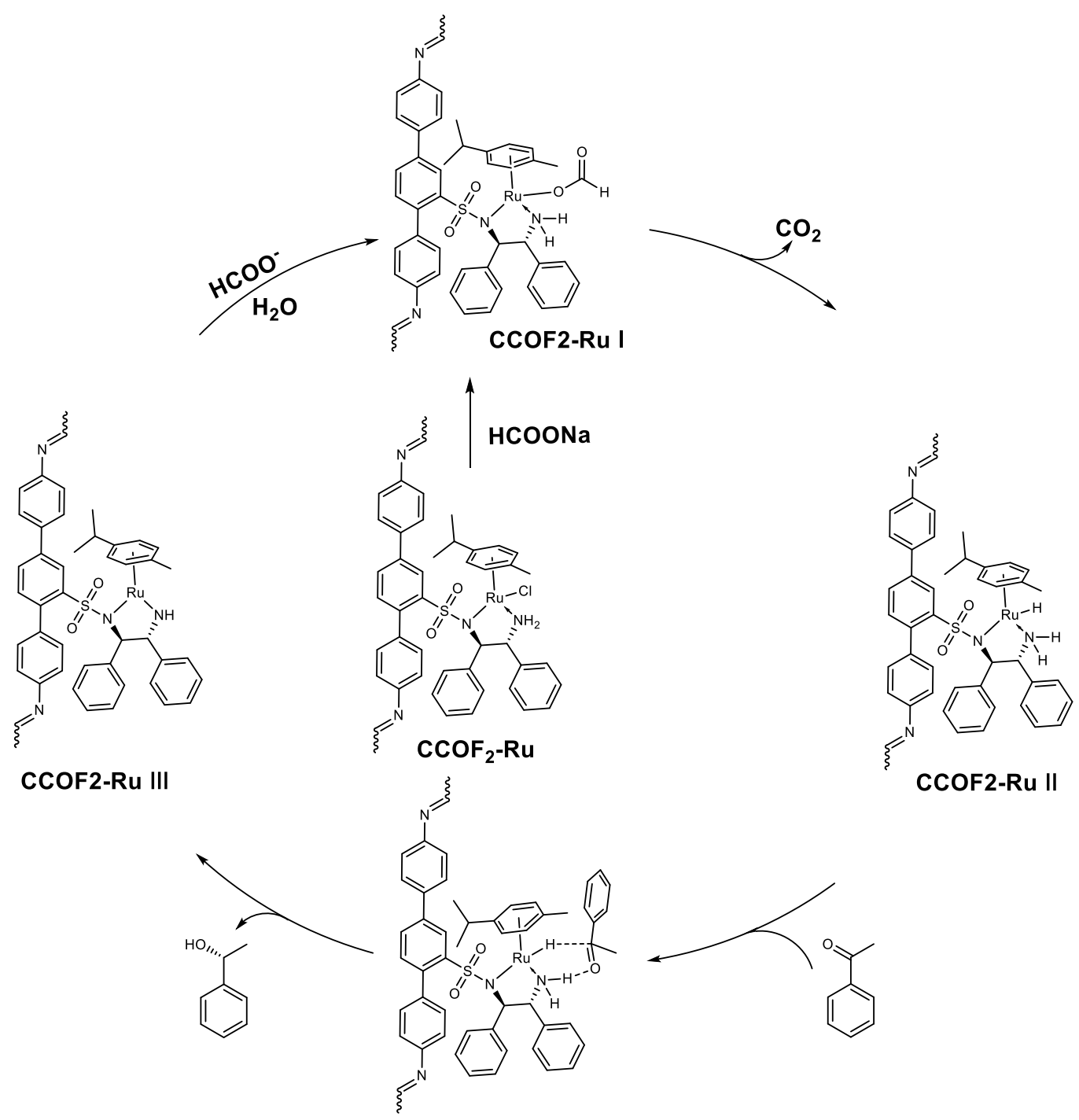

Supplementary Fig. 29 Plausible reaction mechanism for COF2-Ru catalyzed asymmetric transfer hydrogenation of acetophenone.

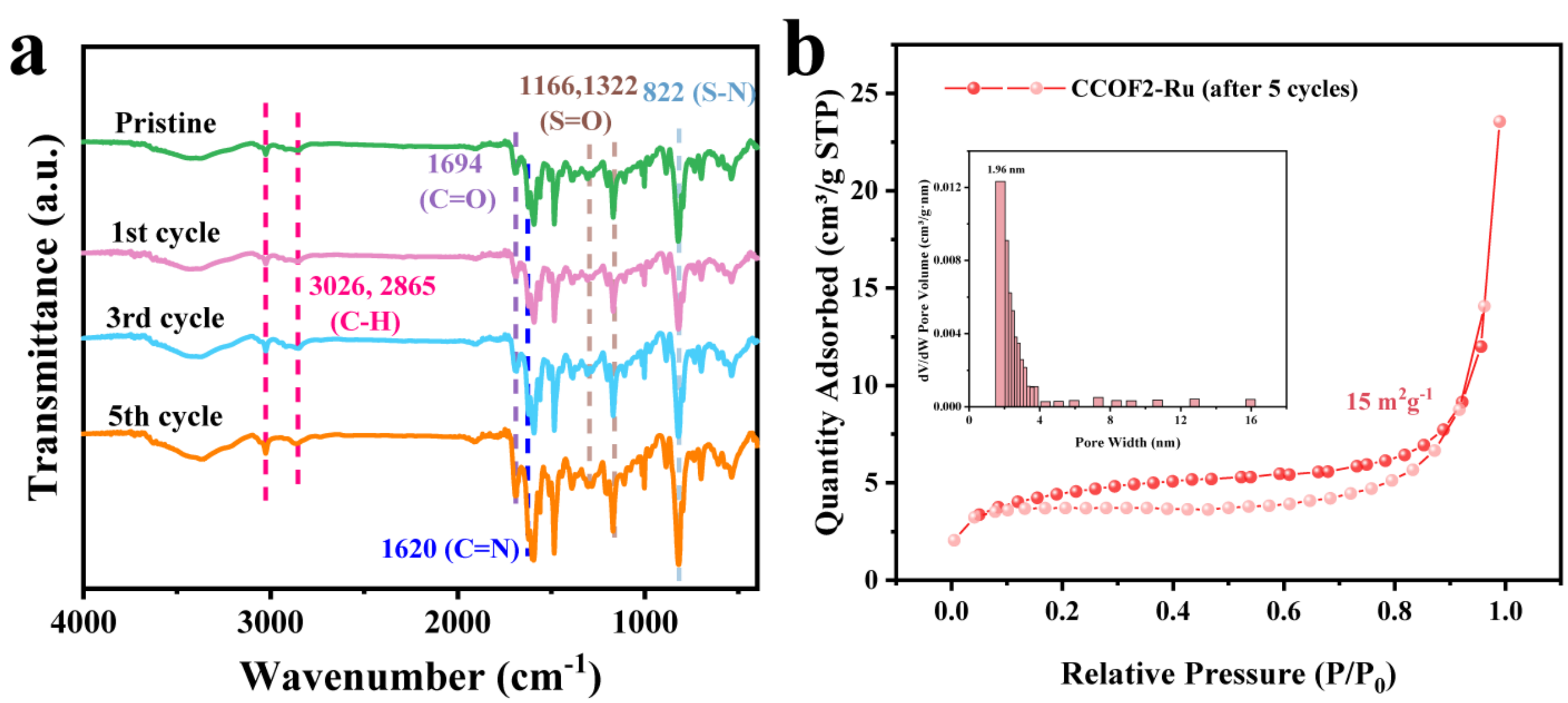

Supplementary Fig. 30 a FTIR spectra of pristine CCOF2-Ru and the recovered catalyst after 5 cycles; **b** nitrogen adsorption/desorption isotherms (measured at 77 K, corresponding pore size distributions are shown in the inset.) of CCOF2-Ru after 5 cycles.

Section 4. Supplementary Tables

Supplementary Table 1. Element analysis data.[a]

COFs	C (wt%)	H (wt%)	N (wt%)	S (wt%)	$n_{(N)}/ n_{(S)}$
CCOF1-Boc	76.33	5.22	6.05	2.49	5.55/1
Theoretical Value	79.61	5.31	6.36	2.91	5/1
CCOF2-Boc	79.75	5.18	5.67	1.68	7.71/1
Theoretical Value	83.42	5.18	6.13	1.75	8/1

[a]The elemental analysis data are reported as the average of two independent measurements.

During the synthesis of CCOF2-Boc, 0.12 mmol of TFPB, 0.12 mmol of DATP, and 0.06 mmol of (R)-NH_2-Boc were used. Each mole of DATP contains 2 moles of nitrogen (N) atoms, while each mole of (R)-NH_2-Boc contains 4 moles of N atoms and 1 mole of sulfur (S) atom.

Therefore, the theoretical molar ratio of nitrogen (N) to sulfur (S) atoms in CCOF2-Boc is calculated as follows:

N atoms from DATP = 0.12 mmol × 2 = 0.24 mmol

N atoms from (R)-NH_2-Boc = 0.06 mmol × 4 = 0.24 mmol

Total N atoms = 0.24 mmol + 0.24 mmol = 0.48 mmol

S atoms from (R)-NH_2-Boc = 0.06 mmol × 1 = 0.06 mmol

Thus, the theoretical molar ratio of N to S atoms in CCOF2-Boc is:

N:S ratio = 0.48 mmol / 0.06 mmol = 8:1

In summary, the theoretical molar ratio of N to S atoms in CCOF2-Boc is 8:1. Similarly, the theoretical molar ratio of N to S atoms in CCOF1-Boc is 5:1.

Supplementary Table 2. Theoretical BET surface area and pore volumes of CCOFs.

COFs	BET surface areas (m^2/g)	Theoretical BET surface area (m^2/g)	Pore volumes (cm^3/g)	Theoretical pore volumes (cm^3/g)
CCOF0-Boc	21	2009	0.02	1.30
CCOF1-Boc	70	2446	0.03	1.69
CCOF2-Boc	518	2304	0.33	2.03
CCOF1	40	2663	0.02	2.05
CCOF2	473	2451	0.36	2.19

Supplementary Table 3. Rietveld refined crystal unit cell parameters of CCOFs.

COFs	stacking mode	unit-cell parameters	Rwp, Rp
CCOF0-Boc	AA	P3 a = b = 45.4766 Å, c = 4.8584 Å, α =β = 90.00°, γ = 120.00°	Rwp = 4.66%<br>Rp = 3.19%
CCOF1-Boc	AA	$P2_1$ a = 52.8905 Å, b = 51.8086 Å, c = 7.6248 Å α = β = 90°, γ = 120.7785°	Rwp = 5.89%<br>Rp = 3.86%
CCOF2-Boc	AA	$P2_1$ a = 51.8041 Å, b = 52.2932 Å, c = 7.1590Å α =β = 90°, γ = 119.8850°	Rwp = 5.87%<br>Rp = 4.08%
CCOF1	AA	$P2_1$ a = 52.9766 Å, b = 51.9749 Å, c = 7.9221 Å α =β = 90°, γ = 121.2603°	Rwp = 6.93%<br>Rp = 3.82%
CCOF2	AA	$P2_1$ a = 51.7202 Å, b = 51.9502 Å, c = 7.1166 Å α =β = 90°, γ = 119.3502°	Rwp = 7.07%<br>Rp = 4.41%

Supplementary Table 4. Atomic coordinates for the AA-stacking mode of CCOF0-Boc.

Atom	Element	x	y	z
C1	C	0.32174	0.17302	-0.67884
C2	C	0.34299	0.15778	-0.67657
C3	C	0.37215	0.17289	-0.52267

C4	C	0.3824	0.20235	-0.36058
C5	C	0.36229	0.21826	-0.38053
C6	C	0.33357	0.20464	-0.53714
C7	C	0.41078	0.21465	-0.17144
C8	C	0.28816	0.15581	-0.78116
C9	C	0.42652	0.24799	-0.05732
C10	C	0.45269	0.25885	0.12891
C11	C	0.46537	0.23769	0.21322
C12	C	0.45015	0.20484	0.10236
C13	C	0.4238	0.19353	-0.08185
C14	C	0.27649	0.17032	-0.98684
C15	C	0.2437	0.15274	-1.08283
C16	C	0.22004	0.12062	-0.97913
C17	C	0.23048	0.10688	-0.76657
C18	C	0.26324	0.12345	-0.67021
N19	N	0.49266	0.24825	0.39798
N20	N	0.18718	0.10351	-1.09321
C21	C	0.16234	0.07362	-1.02977
C22	C	0.1314	0.05882	-1.19151
C23	C	0.50428	0.27373	0.56683
C24	C	0.5348	0.28285	0.72409
C25	C	0.10568	0.02526	-1.1381
C26	C	0.07583	0.01001	-1.28394
C27	C	0.06917	0.02801	-1.49433
C28	C	0.09557	0.06194	-1.54856
C29	C	0.12536	0.0765	-1.40179
C30	C	0.54683	0.30961	0.91584
C31	C	0.57579	0.31937	1.06696
C32	C	0.59563	0.30329	1.03276
C33	C	0.58288	0.27571	0.84094
C34	C	0.55378	0.26631	0.6929
C35	C	0.62908	0.31725	1.1531
C36	C	0.65031	0.35282	1.17676
C37	C	0.03594	0.0144	-1.61554
C38	C	0.01415	-0.02092	-1.63882
S39	S	0.33358	0.12136	-0.87849
O40	O	0.31227	0.11761	-1.11003
O41	O	0.36355	0.11861	-0.91126
C42	C	0.30843	0.03435	-0.61103
C43	C	0.2936	0.05362	-0.76868
N44	N	0.34565	0.05209	-0.62919
N45	N	0.3083	0.08849	-0.66153
C46	C	0.29621	-0.00243	-0.70009
C47	C	0.25493	0.03472	-0.78763
H48	H	0.30227	0.03443	-0.39167

H49	H	0.30322	0.05647	-0.98182
H50	H	0.35512	0.0585	-0.82199
H51	H	0.32157	0.09165	-0.48762
C52	C	0.36688	0.05577	-0.42112
O53	O	0.39933	0.07113	-0.52294
C54	C	0.42565	0.0816	-0.32522
O55	O	0.35865	0.04662	-0.18495
C56	C	0.45915	0.09874	-0.49144
C57	C	0.42367	0.05075	-0.17075
C58	C	0.42563	0.10921	-0.13879
H59	H	0.46137	0.12075	-0.60335
H60	H	0.459	0.08079	-0.64318
H61	H	0.48155	0.10744	-0.3584
H62	H	0.41752	0.03044	-0.32097
H63	H	0.40387	0.04068	-0.01074
H64	H	0.44791	0.05742	-0.07115
H65	H	0.45029	0.12537	-0.03989
H66	H	0.4061	0.09858	0.0231
H67	H	0.41955	0.12501	-0.27093
H68	H	0.38746	0.16039	-0.52016
H69	H	0.36869	0.24106	-0.25876
H70	H	0.31755	0.21647	-0.53539
H71	H	0.41809	0.26588	-0.1176
H72	H	0.46385	0.28491	0.20746
H73	H	0.45922	0.1875	0.16407
H74	H	0.41212	0.16717	-0.15372
H75	H	0.29435	0.19541	-1.07228
H76	H	0.23604	0.16412	-1.2474
H77	H	0.21283	0.08219	-0.67428
H78	H	0.27089	0.11198	-0.50597
H79	H	0.16366	0.05793	-0.86316
H80	H	0.4925	0.28957	0.59475
H81	H	0.10897	0.01044	-0.97429
H82	H	0.05602	-0.01573	-1.22461
H83	H	0.09181	0.07706	-1.7069
H84	H	0.14475	0.10277	-1.45191
H85	H	0.53296	0.32334	0.94951
H86	H	0.58421	0.34075	1.21109
H87	H	0.59745	0.26296	0.79563
H88	H	0.54566	0.24548	0.54354
H89	H	0.63877	0.36876	1.14138
H90	H	0.02522	-0.03725	-1.6012
C91	C	0.95338	1.19342	-0.00568
C92	C	0.96923	1.17424	-0.03913
C93	C	0.99528	1.17895	0.13919

C94	C	1.0048	1.20283	0.35023
C95	C	0.98852	1.22169	0.38111
C96	C	1.01998	1.3258	0.44268
C97	C	1.0537	1.34862	0.37724
C98	C	1.06895	1.34214	0.15443
C99	C	1.04969	1.31294	-0.00202
C100	C	1.01578	1.29087	0.06302
H101	H	0.93265	1.18924	-0.14615
H102	H	0.96118	1.1555	-0.20655
H103	H	1.00801	1.16398	0.11467
H104	H	1.0251	1.2067	0.49412
H105	H	0.99653	1.24021	0.54925
H106	H	1.00879	1.33115	0.62302
H107	H	1.06836	1.37145	0.50394
H108	H	1.09564	1.35977	0.10232
H109	H	1.06118	1.30742	-0.17946
H110	H	1.00142	1.2687	-0.06939

Supplementary Table 5. Atomic coordinates for the AA-stacking mode of CCOF1-Boc.

Atom	Element	x	y	z
C1	C	0.67892	0.36423	0.20984
C2	C	0.64825	0.35136	0.20199
C3	C	0.63057	0.32074	0.15815
C4	C	0.64321	0.30312	0.12287
C5	C	0.67396	0.31683	0.1249
C6	C	0.69195	0.34715	0.17116
C7	C	0.62448	0.27009	0.09513
C8	C	0.72426	0.36058	0.18539
C9	C	0.63467	0.36984	0.23972
C10	C	0.63312	0.25069	0.16544
C11	C	0.61527	0.2196	0.14718
C12	C	0.5883	0.20711	0.0582
C13	C	0.57962	0.22633	-0.01322
C14	C	0.59752	0.25757	0.005
C15	C	0.74365	0.38962	0.12185
C16	C	0.77408	0.40192	0.1337
C17	C	0.7856	0.38556	0.21185
C18	C	0.76625	0.35674	0.27738
C19	C	0.73589	0.3443	0.26334
C20	C	0.64836	0.39982	0.18254
C21	C	0.63471	0.4166	0.20632
C22	C	0.60713	0.4038	0.28787

C23	C	0.59392	0.3743	0.35154
C24	C	0.60753	0.35745	0.32769
C25	C	0.56933	0.17422	0.03877
C26	C	0.81759	0.39816	0.2272
C27	C	0.59228	0.42128	0.30008
C28	C	0.53122	0.01044	0.11069
C29	C	0.51642	0.0269	0.11486
C30	C	0.48537	0.0106	0.11373
C31	C	0.53188	0.0605	0.11652
C32	C	0.52081	0.07529	0.0163
C33	C	0.53442	0.10666	0.01854
C34	C	0.55995	0.12403	0.1187
C35	C	0.57099	0.10944	0.21857
C36	C	0.55671	0.07808	0.22166
N37	N	0.57546	0.1563	0.12385
C38	C	0.9814	0.51599	0.10633
C39	C	0.96279	0.4842	0.11633
C40	C	0.97631	0.4667	0.12178
C41	C	0.92983	0.46834	0.12512
C42	C	0.91407	0.44223	0.22814
C43	C	0.88327	0.42651	0.23358
C44	C	0.86745	0.43701	0.13983
C45	C	0.88289	0.46281	0.03822
C46	C	0.91356	0.47773	0.02632
N47	N	0.83589	0.42217	0.1404
C48	C	0.49857	0.46729	0.46207
C49	C	0.51166	0.49693	0.52286
C50	C	0.46894	0.4525	0.40945
C51	C	0.51647	0.45275	0.44341
C52	C	0.50434	0.42195	0.47668
C53	C	0.5214	0.40856	0.4554
C54	C	0.5507	0.42551	0.4005
C55	C	0.56301	0.45642	0.3698
C56	C	0.546	0.46978	0.3895
N57	N	0.56716	0.41061	0.38088
H58	H	0.69267	0.38731	0.25202
H59	H	0.60692	0.31046	0.15482
H60	H	0.68406	0.30389	0.09051
H61	H	0.65315	0.25945	0.24134
H62	H	0.6223	0.20518	0.2048
H63	H	0.55915	0.21721	-0.085
H64	H	0.59058	0.27193	-0.05447
H65	H	0.7352	0.4024	0.05889
H66	H	0.78869	0.42416	0.08157
H67	H	0.77466	0.34393	0.34046

H68	H	0.72137	0.32213	0.31676
H69	H	0.66891	0.40979	0.11097
H70	H	0.6452	0.43928	0.15541
H71	H	0.57289	0.36427	0.4179
H72	H	0.59684	0.33477	0.37834
H73	H	0.55066	0.16568	-0.0485
H74	H	0.82516	0.38678	0.3116
H75	H	0.60239	0.44274	0.23416
H76	H	0.47309	0.02212	0.11875
H77	H	0.50205	0.06261	-0.06881
H78	H	0.52536	0.11697	-0.06235
H79	H	0.5905	0.12263	0.29723
H80	H	0.56512	0.06773	0.30773
H81	H	0.9632	0.44243	0.12549
H82	H	0.92555	0.4343	0.30872
H83	H	0.87223	0.40682	0.31509
H84	H	0.87085	0.47067	-0.03753
H85	H	0.92422	0.49612	-0.06454
H86	H	0.53418	0.50867	0.56874
H87	H	0.4583	0.42974	0.35991
H88	H	0.48183	0.40831	0.52135
H89	H	0.51191	0.38487	0.48152
H90	H	0.58571	0.47052	0.33153
H91	H	0.5559	0.4934	0.3591
C92	C	0.30811	0.62398	0.19578
C93	C	0.33869	0.63605	0.18882
C94	C	0.35719	0.66709	0.1615
C95	C	0.34556	0.6859	0.13684
C96	C	0.31486	0.67309	0.13876
C97	C	0.29598	0.64229	0.1704
C98	C	0.3654	0.71891	0.11178
C99	C	0.26361	0.62936	0.17878
C100	C	0.35128	0.61624	0.20936
C101	C	0.35763	0.7392	0.17904
C102	C	0.37628	0.77014	0.15774
C103	C	0.40317	0.78152	0.06901
C104	C	0.41113	0.76147	0.00236
C105	C	0.39246	0.73048	0.02352
C106	C	0.24415	0.60118	0.10426
C107	C	0.2137	0.58902	0.11406
C108	C	0.20219	0.60478	0.19939
C109	C	0.22156	0.63292	0.27319
C110	C	0.25196	0.64513	0.26278
C111	C	0.33651	0.58693	0.14063
C112	C	0.34799	0.5681	0.16201

C113	C	0.37467	0.5783	0.25077
C114	C	0.3899	0.60781	0.31549
C115	C	0.37824	0.62652	0.29628
C116	C	0.42295	0.81413	0.04448
C117	C	0.17019	0.59232	0.21414
C118	C	0.3861	0.55788	0.27619
C119	C	0.46951	0.9789	0.10691
C120	C	0.48401	0.96252	0.1082
C121	C	0.51493	0.97861	0.11133
C122	C	0.46704	0.92889	0.10938
C123	C	0.4793	0.91237	0.04142
C124	C	0.46364	0.88093	0.04775
C125	C	0.435	0.86524	0.11708
C126	C	0.42282	0.88145	0.1852
C127	C	0.43856	0.91288	0.18235
N128	N	0.41787	0.83305	0.1264
C129	C	0.00684	0.47926	0.1256
C130	C	0.02506	0.51053	0.11496
C131	C	0.01183	0.52796	0.10057
C132	C	0.05775	0.52531	0.12179
C133	C	0.07487	0.55411	0.19599
C134	C	0.10552	0.56844	0.19922
C135	C	0.11998	0.55428	0.12982
C136	C	0.10307	0.52564	0.05655
C137	C	0.07231	0.5112	0.05287
N138	N	0.15151	0.56821	0.12864
C139	C	0.4671	0.49806	0.45975
C140	C	0.45338	0.46773	0.40783
C141	C	0.49612	0.51204	0.52289
C142	C	0.45213	0.51573	0.43823
C143	C	0.4687	0.54671	0.40087
C144	C	0.45491	0.5633	0.37759
C145	C	0.42437	0.54933	0.38333
C146	C	0.40753	0.51855	0.42211
C147	C	0.42131	0.50193	0.45079
N148	N	0.41126	0.56721	0.3534
H149	H	0.29368	0.60043	0.22558
H150	H	0.38072	0.67661	0.15908
H151	H	0.3055	0.68699	0.11244
H152	H	0.33756	0.73124	0.25334
H153	H	0.36989	0.78529	0.21207
H154	H	0.43157	0.76978	-0.06861
H155	H	0.39892	0.7156	-0.03327
H156	H	0.25253	0.58885	0.03517
H157	H	0.19907	0.56744	0.05407

H158	H	0.21315	0.64528	0.3414
H159	H	0.26641	0.66649	0.32541
H160	H	0.31632	0.57872	0.06726
H161	H	0.33623	0.54565	0.10794
H162	H	0.41046	0.61606	0.3853
H163	H	0.3899	0.64867	0.35467
H164	H	0.44129	0.82174	-0.04489
H165	H	0.16279	0.60367	0.30016
H166	H	0.37374	0.53528	0.22412
H167	H	0.52698	0.96697	0.11728
H168	H	0.50072	0.92344	-0.02044
H169	H	0.47406	0.86918	-0.00094
H170	H	0.40116	0.86946	0.24425
H171	H	0.42871	0.92442	0.2443
H172	H	0.01621	0.46507	0.14045
H173	H	0.02524	0.55203	0.08578
H174	H	0.06467	0.56543	0.25663
H175	H	0.11743	0.5903	0.25845
H176	H	0.11399	0.51479	-0.0004
H177	H	0.06004	0.48934	-0.00836
H178	H	0.43123	0.45622	0.35617
H179	H	0.50715	0.53494	0.57011
H180	H	0.4923	0.55811	0.3846
H181	H	0.46789	0.58704	0.34837
H182	H	0.38388	0.50762	0.43309
H183	H	0.40784	0.47842	0.48437
S184	S	0.42901	-0.0265	-0.39522
O185	O	0.4155	-0.03386	-0.20882
O186	O	0.41234	-0.0574	-0.488
C187	C	0.38395	0.01752	-0.48815
C188	C	0.39153	-0.0046	-0.39966
N189	N	0.41074	0.04706	-0.5151
N190	N	0.41489	-0.00559	-0.49518
C191	C	0.36184	0.02127	-0.38076
C192	C	0.36838	0.03262	-0.20853
C193	C	0.33424	0.01317	-0.45239
C194	C	0.34766	0.03535	-0.10937
C195	C	0.3136	0.01613	-0.35317
C196	C	0.32029	0.02714	-0.18159
C197	C	0.36423	-0.03578	-0.38503
C198	C	0.35223	-0.04772	-0.21921
C199	C	0.35039	-0.05305	-0.53556
C200	C	0.32717	-0.07634	-0.2045
C201	C	0.32527	-0.08161	-0.52012
C202	C	0.31371	-0.09326	-0.35478

H203	H	0.37383	0.00767	-0.61785
H204	H	0.40012	0.00399	-0.26598
H205	H	0.42488	0.05805	-0.41071
H206	H	0.41119	-0.01	-0.62766
H207	H	0.3894	0.03912	-0.15023
H208	H	0.32866	0.00453	-0.58499
H209	H	0.35287	0.04381	0.02385
H210	H	0.29245	0.00992	-0.40911
H211	H	0.30425	0.02928	-0.1046
H212	H	0.3625	-0.03497	-0.10141
H213	H	0.35926	-0.04461	-0.66479
H214	H	0.3183	-0.08544	-0.07612
H215	H	0.31497	-0.09483	-0.6366
H216	H	0.29441	-0.11551	-0.34293
C217	C	0.41874	0.06132	-0.67315
O218	O	0.44532	0.08884	-0.68403
C219	C	0.45492	0.10407	-0.84794
O220	O	0.40298	0.04994	-0.80057
C221	C	0.48487	0.13175	-0.81292
C222	C	0.43414	0.11426	-0.91337
C223	C	0.45874	0.08474	-0.9844
H224	H	0.49412	0.1456	-0.9329
H225	H	0.50024	0.12465	-0.76623
H226	H	0.48288	0.1455	-0.70848
H227	H	0.4125	0.09485	-0.9506
H228	H	0.44349	0.12885	-1.03037
H229	H	0.43064	0.12681	-0.8081
H230	H	0.47216	0.09841	-1.09715
H231	H	0.43731	0.06709	-1.03632
H232	H	0.46932	0.07351	-0.92041
S233	S	0.03105	-0.54217	-0.39347
O234	O	0.05954	-0.53332	-0.29148
O235	O	0.03916	-0.54647	-0.58167
C236	C	-0.01914	-0.62486	-0.15378
C237	C	0.00643	-0.5917	-0.16705
N238	N	-0.04784	-0.62661	-0.14772
N239	N	0.00479	-0.57963	-0.33668
C240	C	-0.01623	-0.64178	-0.00021
C241	C	-0.01715	-0.63331	0.17347
C242	C	-0.01194	-0.66614	-0.03106
C243	C	-0.01408	-0.64904	0.3126
C244	C	-0.00866	-0.68159	0.10897
C245	C	-0.00973	-0.67306	0.28091
C246	C	0.03597	-0.5896	-0.14141
C247	C	0.05215	-0.57696	0.0133

C248	C	0.0472	-0.60075	-0.26867
C249	C	0.07893	-0.57562	0.04022
C250	C	0.07399	-0.59939	-0.2411
C251	C	0.08983	-0.58685	-0.08673
H252	H	-0.01807	-0.63636	-0.27366
H253	H	0.0033	-0.57852	-0.06334
H254	H	-0.05552	-0.62272	-0.0307
H255	H	-0.00103	-0.59384	-0.44316
H256	H	-0.02024	-0.61461	0.20116
H257	H	-0.01096	-0.6731	-0.16345
H258	H	-0.01528	-0.64276	0.44499
H259	H	-0.00535	-0.70023	0.0841
H260	H	-0.00735	-0.68507	0.3898
H261	H	0.04399	-0.56828	0.11381
H262	H	0.03539	-0.61004	-0.38995
H263	H	0.09128	-0.56581	0.15962
H264	H	0.08257	-0.60784	-0.34048
H265	H	0.11066	-0.58564	-0.06624
C266	C	-0.06692	-0.63588	-0.28521
O267	O	-0.09559	-0.64193	-0.25556
C268	C	-0.11771	-0.65707	-0.38616
O269	O	-0.0588	-0.63868	-0.42979
C270	C	-0.14635	-0.66253	-0.3054
C271	C	-0.12153	-0.68758	-0.43243
C272	C	-0.11111	-0.63748	-0.54864
H273	H	-0.15108	-0.6762	-0.18459
H274	H	-0.16507	-0.67436	-0.39786
H275	H	-0.14426	-0.64094	-0.26749
H276	H	-0.14147	-0.70094	-0.51401
H277	H	-0.12351	-0.70009	-0.31029
H278	H	-0.10243	-0.68495	-0.50737
H279	H	-0.09276	-0.63631	-0.62494
H280	H	-0.10459	-0.61447	-0.50673
H281	H	-0.13056	-0.64653	-0.63623
H282	H	0.44574	0.96765	0.09929

Supplementary Table 6. Atomic coordinates for the AA-stacking mode of CCOF2-Boc.

Atom	Element	x	y	z
C1	C	0.68832	0.36191	0.10539
C2	C	0.65752	0.35053	0.08323
C3	C	0.63915	0.31973	0.09418
C4	C	0.65032	0.30049	0.12648

C5	C	0.68136	0.31325	0.14623
C6	C	0.701	0.34387	0.13475
C7	C	0.63073	0.26907	0.13873
C8	C	0.73278	0.35621	0.14823
C9	C	0.64522	0.36958	0.05133
C10	C	0.64065	0.24866	0.09683
C11	C	0.62183	0.21877	0.111
C12	C	0.59196	0.20669	0.16789
C13	C	0.58195	0.22664	0.20868
C14	C	0.60032	0.2566	0.1946
C15	C	0.75209	0.38599	0.20544
C16	C	0.78242	0.39771	0.21156
C17	C	0.79603	0.38112	0.16232
C18	C	0.77717	0.35171	0.10678
C19	C	0.7468	0.33951	0.09996
C20	C	0.66273	0.39878	-0.02068
C21	C	0.65107	0.41712	-0.04267
C22	C	0.62136	0.40813	0.00382
C23	C	0.60379	0.37923	0.0727
C24	C	0.61496	0.36051	0.0952
C25	C	0.57165	0.17522	0.18568
C26	C	0.82799	0.39287	0.16603
C27	C	0.61005	0.42832	-0.01964
C28	C	0.53441	0.01517	0.16532
C29	C	0.51862	0.03104	0.1627
C30	C	0.48692	0.01385	0.16428
C31	C	0.53401	0.06293	0.15757
C32	C	0.51994	0.07922	0.21334
C33	C	0.53484	0.10953	0.20942
C34	C	0.56466	0.12586	0.15008
C35	C	0.57871	0.11008	0.09417
C36	C	0.56417	0.0798	0.09695
N37	N	0.58082	0.1569	0.14442
C38	C	0.98826	0.51639	0.25382
C39	C	0.97241	0.48493	0.25351
C40	C	0.98993	0.47083	0.25579
C41	C	0.94025	0.46815	0.24707
C42	C	0.92473	0.43831	0.18395
C43	C	0.89419	0.42259	0.17475
C44	C	0.8768	0.4352	0.22757
C45	C	0.8918	0.46452	0.29065
C46	C	0.92233	0.48059	0.3008
N47	N	0.84545	0.41986	0.21919
C48	C	0.51867	0.48219	0.03693
C49	C	0.53254	0.51346	0.0402

C50	C	0.48724	0.4665	0.03662
C51	C	0.53546	0.46691	0.03701
C52	C	0.52249	0.43697	0.097
C53	C	0.53833	0.42236	0.09176
C54	C	0.56795	0.43615	0.03007
C55	C	0.58118	0.46576	-0.02765
C56	C	0.56567	0.48076	-0.02399
N57	N	0.58299	0.41999	0.03144
H58	H	0.70322	0.38587	0.09658
H59	H	0.6151	0.31013	0.0721
H60	H	0.6906	0.29875	0.17492
H61	H	0.66361	0.25667	0.04954
H62	H	0.63071	0.20415	0.07592
H63	H	0.55888	0.21848	0.25436
H64	H	0.59129	0.271	0.23195
H65	H	0.74286	0.40003	0.24733
H66	H	0.79614	0.42075	0.25699
H67	H	0.78657	0.33776	0.06572
H68	H	0.73337	0.31665	0.05075
H69	H	0.68605	0.40715	-0.06114
H70	H	0.66574	0.43934	-0.09868
H71	H	0.58044	0.37109	0.11183
H72	H	0.60022	0.33856	0.15466
H73	H	0.54891	0.16822	0.23373
H74	H	0.83609	0.37801	0.12063
H75	H	0.62533	0.45026	-0.07758
H76	H	0.4734	0.02469	0.16013
H77	H	0.49688	0.06773	0.26474
H78	H	0.52274	0.12065	0.25586
H79	H	0.60191	0.12207	0.04568
H80	H	0.5763	0.06879	0.04845
H81	H	0.93708	0.42744	0.13685
H82	H	0.8838	0.39983	0.12315
H83	H	0.87893	0.47502	0.33422
H84	H	0.9327	0.5033	0.35393
H85	H	0.55695	0.52701	0.04243
H86	H	0.47563	0.44231	0.0306
H87	H	0.49962	0.42501	0.15154
H88	H	0.52742	0.39936	0.1394
H89	H	0.60426	0.47764	-0.07807
H90	H	0.57691	0.50371	-0.07376
C91	C	0.31983	0.63756	0.09806
C92	C	0.35067	0.64862	0.07959
C93	C	0.36932	0.67937	0.09389
C94	C	0.3584	0.69877	0.1285

C95	C	0.32737	0.68627	0.14624
C96	C	0.30746	0.65578	0.13075
C97	C	0.378	0.73013	0.14602
C98	C	0.27578	0.64394	0.14604
C99	C	0.36267	0.62922	0.05066
C100	C	0.36811	0.75064	0.10602
C101	C	0.38689	0.78048	0.12249
C102	C	0.4167	0.79245	0.18016
C103	C	0.42662	0.77241	0.22057
C104	C	0.40827	0.74246	0.20435
C105	C	0.25611	0.61421	0.20263
C106	C	0.22587	0.60302	0.21025
C107	C	0.21271	0.62011	0.16277
C108	C	0.23195	0.6495	0.10863
C109	C	0.26224	0.66121	0.10093
C110	C	0.345	0.60006	-0.0215
C111	C	0.3563	0.58139	-0.04146
C112	C	0.38584	0.58996	0.00694
C113	C	0.40357	0.61878	0.07664
C114	C	0.39272	0.63781	0.09764
C115	C	0.43687	0.82394	0.19856
C116	C	0.18084	0.6089	0.16701
C117	C	0.39664	0.56931	-0.01594
C118	C	0.47246	0.98378	0.1679
C119	C	0.48825	0.96791	0.16914
C120	C	0.51996	0.9851	0.16761
C121	C	0.47294	0.93602	0.17003
C122	C	0.48731	0.92011	0.22772
C123	C	0.47287	0.88982	0.22359
C124	C	0.44315	0.87312	0.16308
C125	C	0.42866	0.8885	0.10734
C126	C	0.44276	0.91877	0.11034
N127	N	0.42758	0.84209	0.15556
C128	C	0.02027	0.48659	0.25543
C129	C	0.03611	0.51805	0.25393
C130	C	0.01861	0.53216	0.25423
C131	C	0.06829	0.53472	0.24879
C132	C	0.08406	0.5645	0.18483
C133	C	0.11462	0.57991	0.17503
C134	C	0.13174	0.56701	0.22799
C135	C	0.11648	0.53778	0.2925
C136	C	0.08596	0.52206	0.30373
N137	N	0.16306	0.58189	0.21842
C138	C	0.48422	0.51119	0.0448
C139	C	0.46995	0.47947	0.04427

C140	C	0.51604	0.52721	0.04382
C141	C	0.46867	0.52746	0.03737
C142	C	0.48104	0.55616	0.11883
C143	C	0.46597	0.57156	0.11546
C144	C	0.43785	0.56011	0.03234
C145	C	0.42561	0.53222	-0.05249
C146	C	0.44065	0.51675	-0.05432
N147	N	0.42344	0.57689	0.03733
H148	H	0.30465	0.61368	0.0869
H149	H	0.39341	0.68879	0.07497
H150	H	0.31834	0.70085	0.17821
H151	H	0.34525	0.74277	0.05718
H152	H	0.37802	0.79513	0.08824
H153	H	0.44964	0.78051	0.26721
H154	H	0.41721	0.72794	0.24061
H155	H	0.265	0.59979	0.24259
H156	H	0.21183	0.57999	0.25502
H157	H	0.22292	0.66385	0.06954
H158	H	0.27595	0.68411	0.05304
H159	H	0.32182	0.59198	-0.06422
H160	H	0.34147	0.55923	-0.09804
H161	H	0.42678	0.62656	0.11766
H162	H	0.40756	0.65967	0.15772
H163	H	0.45953	0.83109	0.24905
H164	H	0.17309	0.62414	0.12402
H165	H	0.38113	0.54762	-0.07621
H166	H	0.53348	0.97427	0.16602
H167	H	0.51039	0.93193	0.27868
H168	H	0.48527	0.87901	0.27009
H169	H	0.40551	0.87616	0.05812
H170	H	0.43039	0.92947	0.06067
H171	H	0.0719	0.57551	0.13692
H172	H	0.12524	0.60262	0.12223
H173	H	0.12914	0.52707	0.33647
H174	H	0.07541	0.49939	0.35777
H175	H	0.52808	0.55149	0.042
H176	H	0.50258	0.56605	0.19167
H177	H	0.47606	0.59321	0.18372
H178	H	0.40382	0.52221	-0.12166
H179	H	0.43027	0.49528	-0.12586
S180	S	-0.43095	-0.45467	-0.44117
O181	O	-0.42065	-0.44049	-0.61913
O182	O	-0.41542	-0.46658	-0.33935
C183	C	-0.40049	-0.37498	-0.19271
C184	C	-0.39911	-0.40136	-0.27399

N185	N	-0.43001	-0.37708	-0.18045
N186	N	-0.42861	-0.42753	-0.29592
C187	C	-0.3804	-0.34782	-0.30852
C188	C	-0.38757	-0.34432	-0.49121
C189	C	-0.35356	-0.32573	-0.23465
C190	C	-0.36874	-0.31958	-0.59675
C191	C	-0.33446	-0.30086	-0.33822
C192	C	-0.34196	-0.29765	-0.52066
C193	C	-0.3762	-0.40495	-0.16218
C194	C	-0.34781	-0.39589	-0.23235
C195	C	-0.38283	-0.41686	0.0171
C196	C	-0.32682	-0.39893	-0.12774
C197	C	-0.36218	-0.42007	0.12409
C198	C	-0.3339	-0.4111	0.05147
H199	H	-0.39244	-0.37109	-0.04682
H200	H	-0.39115	-0.39676	-0.41965
H201	H	-0.44313	-0.38607	-0.29278
H202	H	-0.43659	-0.43715	-0.1723
H203	H	-0.40852	-0.36153	-0.55329
H204	H	-0.3474	-0.32803	-0.09118
H205	H	-0.37503	-0.31737	-0.74018
H206	H	-0.31347	-0.28377	-0.27642
H207	H	-0.32697	-0.27809	-0.60339
H208	H	-0.34203	-0.38641	-0.37349
H209	H	-0.40502	-0.42379	0.07438
H210	H	-0.30462	-0.39172	-0.18645
H211	H	-0.3684	-0.42961	0.26535
H212	H	-0.31737	-0.4135	0.13421
C213	C	-0.43413	-0.35762	-0.07591
O214	O	-0.46113	-0.36074	-0.12325
C215	C	-0.46863	-0.34062	-0.0407
O216	O	-0.41696	-0.34071	0.04015
C217	C	-0.50016	-0.34997	-0.11486
C218	C	-0.44728	-0.30875	-0.10989
C219	C	-0.47034	-0.34302	0.17606
H220	H	-0.51565	-0.37288	-0.07368
H221	H	-0.49969	-0.34908	-0.26829
H222	H	-0.50865	-0.33584	-0.06208
H223	H	-0.44611	-0.30892	-0.26319
H224	H	-0.42456	-0.30011	-0.05561
H225	H	-0.4552	-0.29366	-0.06701
H226	H	-0.44824	-0.3331	0.24179
H227	H	-0.48248	-0.36635	0.21623
H228	H	-0.48189	-0.33194	0.23374
H229	H	0.55888	0.02708	0.16645

H230	H	0.448	0.97187	0.17094
H231	H	0.97609	0.52861	0.24995
H232	H	0.97912	0.44664	0.25783
H233	H	0.03239	0.47433	0.25302
H234	H	0.02944	0.55635	0.255

Supplementary Table 7. Atomic coordinates for the AA-stacking mode of CCOF1.

Atom	Element	x	y	z
C1	C	0.67926	0.36595	0.24929
C2	C	0.64862	0.35432	0.23382
C3	C	0.63026	0.32311	0.1951
C4	C	0.64186	0.30423	0.1657
C5	C	0.67282	0.31857	0.1485
C6	C	0.69214	0.34878	0.20348
C7	C	0.62333	0.27207	0.15136
C8	C	0.72311	0.36119	0.21241
C9	C	0.6368	0.37336	0.25789
C10	C	0.63113	0.2523	0.23218
C11	C	0.61279	0.22138	0.22497
C12	C	0.58578	0.20763	0.13696
C13	C	0.5782	0.22679	0.05385
C14	C	0.59617	0.25783	0.0583
C15	C	0.74381	0.38978	0.13597
C16	C	0.77365	0.40113	0.14473
C17	C	0.78526	0.3855	0.2288
C18	C	0.7652	0.3575	0.30438
C19	C	0.73526	0.34549	0.2975
C20	C	0.65069	0.40306	0.184
C21	C	0.63818	0.42055	0.1966
C22	C	0.61162	0.41049	0.28464
C23	C	0.59843	0.38193	0.36413
C24	C	0.6105	0.36397	0.35425
C25	C	0.56621	0.17494	0.12981
C26	C	0.81665	0.39699	0.24046
C27	C	0.59925	0.42981	0.28954
C28	C	0.53097	0.01332	0.1936
C29	C	0.5148	0.02888	0.21175
C30	C	0.48319	0.01062	0.2144
C31	C	0.52791	0.06086	0.21871
C32	C	0.51701	0.07616	0.11844
C33	C	0.53055	0.10724	0.121
C34	C	0.55575	0.12532	0.22066

C35	C	0.56566	0.11048	0.32571
C36	C	0.55084	0.07939	0.3367
N37	N	0.57148	0.15731	0.2209
C38	C	0.97879	0.51651	0.21055
C39	C	0.96085	0.48393	0.18621
C40	C	0.97571	0.46729	0.1808
C41	C	0.92958	0.46732	0.17425
C42	C	0.91188	0.44036	0.27199
C43	C	0.88156	0.42499	0.26475
C44	C	0.86661	0.43507	0.16239
C45	C	0.88372	0.46055	0.05995
C46	C	0.91398	0.47477	0.05445
N47	N	0.83559	0.42096	0.15696
C48	C	0.51093	0.48236	0.39821
C49	C	0.52468	0.51466	0.40332
C50	C	0.47935	0.46483	0.38984
C51	C	0.52806	0.46812	0.39841
C52	C	0.51853	0.44025	0.4857
C53	C	0.53423	0.42587	0.47421
C54	C	0.56075	0.43835	0.38292
C55	C	0.57128	0.46671	0.30495
C56	C	0.55557	0.48111	0.31044
N57	N	0.57542	0.42206	0.37491
H58	H	0.69385	0.38967	0.2888
H59	H	0.60618	0.31304	0.19497
H60	H	0.68239	0.30474	0.11491
H61	H	0.6517	0.26207	0.30577
H62	H	0.61962	0.20731	0.29169
H63	H	0.55749	0.21709	-0.01817
H64	H	0.58942	0.27195	-0.00859
H65	H	0.73558	0.40251	0.06779
H66	H	0.7886	0.42299	0.0831
H67	H	0.77328	0.34458	0.37192
H68	H	0.72033	0.32356	0.35847
H69	H	0.67096	0.41137	0.10917
H70	H	0.64935	0.44273	0.13306
H71	H	0.57792	0.3735	0.43669
H72	H	0.59932	0.34169	0.41674
H73	H	0.54686	0.16538	0.04598
H74	H	0.82404	0.38487	0.32139
H75	H	0.61047	0.45116	0.21774
H76	H	0.47024	0.02178	0.21943
H77	H	0.49835	0.06297	0.03204
H78	H	0.52159	0.11753	0.03757
H79	H	0.58493	0.12389	0.40743

H80	H	0.55925	0.06858	0.42084
H81	H	0.96265	0.44238	0.17079
H82	H	0.92268	0.43263	0.36016
H83	H	0.86942	0.40507	0.34673
H84	H	0.87276	0.46869	-0.02384
H85	H	0.92663	0.49468	-0.02744
H86	H	0.54889	0.52894	0.4102
H87	H	0.46794	0.43993	0.38645
H88	H	0.49791	0.4296	0.55899
H89	H	0.52577	0.40411	0.53973
H90	H	0.59197	0.47783	0.23286
H91	H	0.56399	0.50259	0.24171
C92	C	0.30695	0.62521	0.22937
C93	C	0.33761	0.63677	0.21725
C94	C	0.35639	0.66833	0.19217
C95	C	0.34543	0.68795	0.17323
C96	C	0.3145	0.67451	0.16122
C97	C	0.29468	0.64351	0.19917
C98	C	0.36483	0.72005	0.1628
C99	C	0.2637	0.6314	0.20701
C100	C	0.34942	0.61754	0.23275
C101	C	0.35814	0.74038	0.24692
C102	C	0.37705	0.77122	0.2383
C103	C	0.40377	0.78425	0.1477
C104	C	0.4107	0.7645	0.06593
C105	C	0.39209	0.73357	0.07119
C106	C	0.24296	0.60394	0.12088
C107	C	0.21312	0.59258	0.13122
C108	C	0.20157	0.60729	0.225
C109	C	0.22169	0.63441	0.30822
C110	C	0.2516	0.64625	0.30118
C111	C	0.336	0.58861	0.15014
C112	C	0.34829	0.57087	0.1604
C113	C	0.37448	0.58024	0.2512
C114	C	0.3878	0.60856	0.33331
C115	C	0.37578	0.62656	0.32738
C116	C	0.42391	0.81678	0.13658
C117	C	0.17021	0.59581	0.23963
C118	C	0.38687	0.56094	0.25611
C119	C	0.46885	0.97982	0.1947
C120	C	0.48442	0.96422	0.2116
C121	C	0.51569	0.9823	0.21527
C122	C	0.46895	0.93174	0.21922
C123	C	0.48045	0.91482	0.14349
C124	C	0.4647	0.88364	0.14599

C125	C	0.43661	0.86716	0.22186
C126	C	0.42571	0.88359	0.30275
C127	C	0.44123	0.91473	0.30451
N128	N	0.41918	0.83523	0.22316
C129	C	0.00766	0.48145	0.19418
C130	C	0.02559	0.51371	0.18684
C131	C	0.01074	0.53002	0.18912
C132	C	0.05722	0.52903	0.17765
C133	C	0.07579	0.55823	0.25174
C134	C	0.10603	0.57264	0.24185
C135	C	0.12002	0.55895	0.1625
C136	C	0.10196	0.53063	0.08556
C137	C	0.0717	0.51595	0.09196
N138	N	0.15095	0.57235	0.1537
C139	C	0.4769	0.51071	0.38417
C140	C	0.46306	0.4784	0.39169
C141	C	0.50851	0.52821	0.3869
C142	C	0.45981	0.52493	0.37365
C143	C	0.46863	0.55285	0.45851
C144	C	0.4521	0.5663	0.44611
C145	C	0.42581	0.5532	0.35244
C146	C	0.41672	0.52557	0.2694
C147	C	0.43318	0.51198	0.27692
N148	N	0.41009	0.56838	0.3467
H149	H	0.29199	0.60095	0.25648
H150	H	0.38041	0.67803	0.19152
H151	H	0.30543	0.68916	0.13838
H152	H	0.33771	0.73118	0.32107
H153	H	0.37089	0.78578	0.30568
H154	H	0.43128	0.77361	-0.00677
H155	H	0.39821	0.719	0.00356
H156	H	0.25112	0.59191	0.04521
H157	H	0.19813	0.57145	0.06315
H158	H	0.21366	0.64662	0.38342
H159	H	0.26654	0.66726	0.37027
H160	H	0.31592	0.5808	0.07441
H161	H	0.33724	0.54906	0.09313
H162	H	0.40822	0.61667	0.40629
H163	H	0.38683	0.64842	0.3943
H164	H	0.44312	0.82547	0.05213
H165	H	0.16308	0.60736	0.32705
H166	H	0.37636	0.54003	0.18049
H167	H	0.52811	0.97061	0.22045
H168	H	0.50179	0.92678	0.07705
H169	H	0.47457	0.87204	0.08307

H170	H	0.40427	0.87141	0.3677
H171	H	0.43189	0.92663	0.37087
H172	H	0.01809	0.46773	0.19591
H173	H	0.02416	0.55493	0.18598
H174	H	0.06597	0.56947	0.31986
H175	H	0.11891	0.59497	0.30215
H176	H	0.11212	0.51975	0.01762
H177	H	0.0587	0.49381	0.02877
H178	H	0.43882	0.46414	0.39014
H179	H	0.51998	0.55311	0.38205
H180	H	0.4888	0.56372	0.53587
H181	H	0.45962	0.58775	0.51333
H182	H	0.39647	0.51429	0.19341
H183	H	0.42563	0.49075	0.20717
S184	S	0.42801	-0.0313	-0.33155
O185	O	0.41795	-0.01867	-0.20332
O186	O	0.41434	-0.0638	-0.34909
C187	C	0.38859	-0.00498	-0.6442
C188	C	0.39298	-0.03026	-0.57071
N189	N	0.39651	0.01962	-0.52173
N190	N	0.42391	-0.01833	-0.52396
C191	C	0.35698	-0.01825	-0.70439
C192	C	0.33284	-0.03577	-0.59899
C193	C	0.35124	-0.01608	-0.87425
C194	C	0.30396	-0.04874	-0.65709
C195	C	0.32251	-0.02893	-0.93474
C196	C	0.29862	-0.04564	-0.82633
C197	C	0.38277	-0.05716	-0.68902
C198	C	0.36731	-0.08665	-0.62471
C199	C	0.38763	-0.05387	-0.86262
C200	C	0.35825	-0.11188	-0.72756
C201	C	0.3785	-0.07881	-0.96788
C202	C	0.3639	-0.10807	-0.90068
H203	H	0.40375	0.00532	-0.75231
H204	H	0.37994	-0.03832	-0.45328
H205	H	0.41157	0.02075	-0.43951
H206	H	0.4347	-0.02138	-0.61797
H207	H	0.33664	-0.0384	-0.46534
H208	H	0.36984	-0.00306	-0.96126
H209	H	0.28536	-0.0619	-0.57039
H210	H	0.31866	-0.02636	-1.06843
H211	H	0.27587	-0.05602	-0.87331
H212	H	0.36313	-0.08995	-0.4881
H213	H	0.39955	-0.03105	-0.9183
H214	H	0.34667	-0.13476	-0.67227

H215	H	0.38307	-0.0752	-1.10422
H216	H	0.35687	-0.12783	-0.98306
S217	S	0.03318	-0.54135	-0.27174
O218	O	0.06482	-0.52417	-0.24665
O219	O	0.02037	-0.56206	-0.41266
C220	C	0.0331	-0.6004	-0.16221
C221	C	0.029	-0.58137	-0.02408
N222	N	0.00564	-0.62223	-0.25057
N223	N	0.01759	-0.56229	-0.08867
C224	C	0.04753	-0.61709	-0.09161
C225	C	0.0389	-0.6329	0.06102
C226	C	0.07155	-0.61531	-0.17593
C227	C	0.05309	-0.64715	0.12542
C228	C	0.0859	-0.62953	-0.11378
C229	C	0.07689	-0.64535	0.0387
C230	C	0.05704	-0.56238	0.08061
C231	C	0.05437	-0.56013	0.25454
C232	C	0.0856	-0.54843	0.01482
C233	C	0.07888	-0.5438	0.35838
C234	C	0.11044	-0.5322	0.11672
C235	C	0.10723	-0.52976	0.28982
H236	H	0.04803	-0.5841	-0.25826
H237	H	0.01217	-0.59793	0.064
H238	H	-0.00193	-0.61051	-0.31251
H239	H	-0.00447	-0.57508	-0.09352
H240	H	0.02016	-0.63466	0.13103
H241	H	0.07891	-0.60289	-0.29613
H242	H	0.04568	-0.65956	0.24556
H243	H	0.10455	-0.62798	-0.18386
H244	H	0.0882	-0.6565	0.08911
H245	H	0.03219	-0.5712	0.31096
H246	H	0.08868	-0.54954	-0.12154
H247	H	0.07567	-0.54228	0.49459
H248	H	0.13259	-0.52123	0.0603
H249	H	0.12675	-0.51691	0.37079
H250	H	0.44468	0.96664	0.18437
H251	H	0.37902	0.0158	-0.45304
H252	H	-0.01003	-0.63652	-0.17046

Supplementary Table 8. Atomic coordinates for the AA-stacking mode of CCOF2.

Atom	Element	x	y	z
C1	C	0.69634	0.36235	0.10539

C2	C	0.66574	0.35146	0.08031
C3	C	0.6473	0.32065	0.08816
C4	C	0.65816	0.30094	0.12029
C5	C	0.68902	0.31326	0.14283
C6	C	0.70877	0.34385	0.13356
C7	C	0.6384	0.26951	0.12973
C8	C	0.74044	0.35568	0.14795
C9	C	0.65366	0.37092	0.04708
C10	C	0.64823	0.2487	0.09151
C11	C	0.62926	0.21884	0.10478
C12	C	0.59928	0.20717	0.15656
C13	C	0.58936	0.22751	0.19319
C14	C	0.60789	0.25744	0.18015
C15	C	0.75989	0.38549	0.20357
C16	C	0.79004	0.39663	0.21252
C17	C	0.80337	0.37939	0.16803
C18	C	0.78439	0.34995	0.1138
C19	C	0.75418	0.33835	0.1039
C20	C	0.67142	0.40041	-0.02014
C21	C	0.65982	0.41898	-0.04428
C22	C	0.62993	0.40999	-0.00461
C23	C	0.61215	0.38082	0.0596
C24	C	0.62325	0.36187	0.08392
C25	C	0.57871	0.17577	0.17349
C26	C	0.83517	0.39064	0.17551
C27	C	0.61856	0.43036	-0.02927
C28	C	0.53871	0.01431	0.15943
C29	C	0.52346	0.03092	0.15528
C30	C	0.49186	0.01442	0.15826
C31	C	0.5393	0.06286	0.14793
C32	C	0.52565	0.07992	0.19979
C33	C	0.54104	0.11027	0.19619
C34	C	0.571	0.12591	0.14092
C35	C	0.58462	0.10937	0.08811
C36	C	0.56956	0.07904	0.09035
N37	N	0.58768	0.15697	0.13644
C38	C	0.9943	0.51467	0.27103
C39	C	0.97891	0.48318	0.27048
C40	C	0.99675	0.46923	0.27564
C41	C	0.94687	0.46626	0.26125
C42	C	0.9318	0.43624	0.20057
C43	C	0.90139	0.42043	0.18925
C44	C	0.88372	0.43313	0.23735
C45	C	0.89827	0.46265	0.29757
C46	C	0.92865	0.4788	0.30967

N47	N	0.85251	0.41773	0.22731
C48	C	0.52455	0.48255	0.01638
C49	C	0.53809	0.51385	0.02084
C50	C	0.49322	0.46689	0.02021
C51	C	0.54165	0.46734	0.01162
C52	C	0.52934	0.43739	0.07217
C53	C	0.54588	0.42326	0.07084
C54	C	0.57549	0.43746	0.01046
C55	C	0.58794	0.46693	-0.05048
C56	C	0.57173	0.48145	-0.05053
N57	N	0.59124	0.42178	0.01581
H58	H	0.7113	0.38629	0.09848
H59	H	0.62343	0.31141	0.06319
H60	H	0.698	0.29838	0.1713
H61	H	0.67128	0.25635	0.04861
H62	H	0.6381	0.2039	0.07328
H63	H	0.56622	0.21972	0.23484
H64	H	0.59885	0.2721	0.21472
H65	H	0.75095	0.40006	0.24276
H66	H	0.80384	0.41975	0.25665
H67	H	0.79354	0.33547	0.07651
H68	H	0.7407	0.31538	0.05636
H69	H	0.69491	0.40886	-0.05488
H70	H	0.67472	0.44143	-0.09608
H71	H	0.58866	0.37262	0.0936
H72	H	0.60822	0.33972	0.1394
H73	H	0.55589	0.16926	0.21658
H74	H	0.84328	0.37548	0.13343
H75	H	0.63392	0.45254	-0.08352
H76	H	0.47871	0.0258	0.15331
H77	H	0.50253	0.06904	0.24853
H78	H	0.5292	0.12195	0.23987
H79	H	0.60792	0.1208	0.04266
H80	H	0.58146	0.06747	0.0448
H81	H	0.94438	0.42525	0.15741
H82	H	0.89131	0.39749	0.14
H83	H	0.88517	0.47325	0.33722
H84	H	0.9386	0.50167	0.3604
H85	H	0.56241	0.5276	0.02045
H86	H	0.48187	0.44269	0.01328
H87	H	0.50653	0.42519	0.12628
H88	H	0.5356	0.40033	0.12104
H89	H	0.61096	0.47904	-0.10041
H90	H	0.58244	0.50433	-0.10236
C91	C	0.32346	0.63822	0.08728

C92	C	0.35425	0.64931	0.07112
C93	C	0.3725	0.68011	0.08526
C94	C	0.36131	0.69945	0.12176
C95	C	0.33035	0.68688	0.13831
C96	C	0.31081	0.65635	0.12219
C97	C	0.38067	0.73077	0.14429
C98	C	0.27922	0.64423	0.14305
C99	C	0.36662	0.62987	0.047
C100	C	0.37066	0.75155	0.10978
C101	C	0.38944	0.78138	0.12674
C102	C	0.41933	0.79304	0.18018
C103	C	0.4293	0.77273	0.21681
C104	C	0.41094	0.7428	0.20019
C105	C	0.26017	0.61425	0.19713
C106	C	0.23003	0.60287	0.21115
C107	C	0.21636	0.62002	0.17298
C108	C	0.23498	0.64965	0.12142
C109	C	0.26516	0.66155	0.10753
C110	C	0.3493	0.60035	-0.02046
C111	C	0.36109	0.58187	-0.03902
C112	C	0.39082	0.59098	0.00646
C113	C	0.40812	0.62006	0.07322
C114	C	0.39675	0.63889	0.09295
C115	C	0.43962	0.82449	0.19806
C116	C	0.18457	0.6086	0.18368
C117	C	0.40215	0.57054	-0.016
C118	C	0.47699	0.98564	0.16442
C119	C	0.49223	0.96903	0.16731
C120	C	0.52383	0.98553	0.16433
C121	C	0.47649	0.93707	0.17117
C122	C	0.49065	0.92064	0.22665
C123	C	0.47597	0.8903	0.22261
C124	C	0.44618	0.87404	0.16506
C125	C	0.43184	0.88993	0.1128
C126	C	0.44618	0.92026	0.11554
N127	N	0.43043	0.84298	0.15713
C128	C	0.02549	0.48553	0.27826
C129	C	0.04089	0.51703	0.27707
C130	C	0.02304	0.53098	0.27462
C131	C	0.07296	0.53394	0.27308
C132	C	0.08828	0.56388	0.21104
C133	C	0.11869	0.57934	0.19796
C134	C	0.13614	0.56638	0.24589
C135	C	0.12135	0.53705	0.30967
C136	C	0.09097	0.52129	0.32419

N137	N	0.16732	0.5813	0.23151
C138	C	0.48977	0.51167	0.03182
C139	C	0.47576	0.47991	0.02944
C140	C	0.52142	0.52758	0.02926
C141	C	0.47431	0.52831	0.0264
C142	C	0.48705	0.55734	0.10511
C143	C	0.47225	0.57324	0.1024
C144	C	0.44383	0.56178	0.02414
C145	C	0.43124	0.53359	-0.05817
C146	C	0.44613	0.51778	-0.06187
N147	N	0.42938	0.57874	0.03024
H148	H	0.30858	0.61431	0.07642
H149	H	0.39656	0.68957	0.06788
H150	H	0.32114	0.70138	0.17282
H151	H	0.34779	0.74398	0.06325
H152	H	0.38051	0.79627	0.09606
H153	H	0.45238	0.78056	0.26027
H154	H	0.42002	0.7281	0.23251
H155	H	0.26941	0.59971	0.22996
H156	H	0.21651	0.57963	0.25332
H157	H	0.22553	0.66406	0.08937
H158	H	0.27832	0.68466	0.06212
H159	H	0.32604	0.59184	-0.06181
H160	H	0.34651	0.55945	-0.09253
H161	H	0.43141	0.62822	0.11277
H162	H	0.4114	0.66103	0.14992
H163	H	0.46229	0.8313	0.24627
H164	H	0.17642	0.624	0.1487
H165	H	0.38661	0.5484	-0.06996
H166	H	0.53699	0.97414	0.16389
H167	H	0.51381	0.93204	0.27472
H168	H	0.4883	0.87911	0.26652
H169	H	0.4086	0.87795	0.0664
H170	H	0.43388	0.93132	0.06801
H171	H	0.07591	0.57499	0.16574
H172	H	0.12891	0.60213	0.14592
H173	H	0.13424	0.52625	0.34994
H174	H	0.08083	0.49848	0.37634
H175	H	0.53331	0.55187	0.02912
H176	H	0.50878	0.56723	0.17505
H177	H	0.48287	0.59527	0.16729
H178	H	0.40928	0.52357	-0.12444
H179	H	0.43541	0.49619	-0.13267
S180	S	-0.43684	-0.45523	-0.46311
O181	O	-0.42724	-0.44281	-0.64712

O182	O	-0.42106	-0.46644	-0.35474
C183	C	-0.40571	-0.37176	-0.27911
C184	C	-0.40404	-0.40033	-0.31832
N185	N	-0.42776	-0.37017	-0.40404
N186	N	-0.43367	-0.4265	-0.32866
C187	C	-0.37447	-0.34556	-0.28593
C188	C	-0.3592	-0.33538	-0.4554
C189	C	-0.35974	-0.33158	-0.12068
C190	C	-0.33029	-0.3119	-0.46103
C191	C	-0.33083	-0.30807	-0.12372
C192	C	-0.3159	-0.29807	-0.29453
C193	C	-0.38187	-0.40221	-0.1866
C194	C	-0.35371	-0.39546	-0.25046
C195	C	-0.3887	-0.41039	0.00101
C196	C	-0.33306	-0.39691	-0.13184
C197	C	-0.36844	-0.41226	0.12132
C198	C	-0.34036	-0.40543	0.05535
H199	H	-0.41448	-0.37358	-0.13508
H200	H	-0.39545	-0.3984	-0.46253
H201	H	-0.43757	-0.38816	-0.48625
H202	H	-0.44086	-0.43481	-0.2005
H203	H	-0.37027	-0.34615	-0.58741
H204	H	-0.37119	-0.33932	0.01484
H205	H	-0.31879	-0.30431	-0.59649
H206	H	-0.31976	-0.29742	0.00845
H207	H	-0.29308	-0.27953	-0.29785
H208	H	-0.34771	-0.38889	-0.3978
H209	H	-0.41072	-0.41575	0.0543
H210	H	-0.31106	-0.39148	-0.18608
H211	H	-0.37474	-0.41942	0.26785
H212	H	-0.32427	-0.40686	0.14944
H213	H	0.56307	0.02562	0.15975
H214	H	0.45263	0.97432	0.1684
H215	H	0.98193	0.52684	0.26486
H216	H	0.9863	0.44502	0.2776
H217	H	0.03786	0.47334	0.27774
H218	H	0.03344	0.55518	0.27536
H219	H	-0.41816	-0.35271	-0.49029

Supplementary Table 9. Metal content determined by ICP-OES.

Catalyst	Metal loading (wt%)
CCOF0-Ru	2.12 (Ru)
CCOF1-Ru	1.62 (Ru)
CCOF2-Ru	0.89 (Ru)
CCOF-DACH-Ru	2.68 (Ru)
CCOF2-Ru’	0.92 (Ru)
CCOF2-Rh	0.43 (Rh)
CCOF2-Ir	0.72 (Ir)

Supplementary Table10. EXAFS data fitting results of CCOF2-Ru.

Sample	Path	CN^a	R(Å)^b	σ^2 (Å^2)^c	ΔE_0(eV)^d	R factor
Ru foil	Ru-Ru	12.0*	2.676±0.003	0.0042	-5.3±0.9	0.0069
CCOF2-Ru	Ru-N/C	7.7±0.8	2.159±0.033	0.0083	9.1±3.1	0.0199
	Ru-Cl	0.9±0.3	2.436±0.021	0.0026		

^{a}*CN* is coordination number; ^{b}*R* is the distance between absorber and backscatter atoms; $^c\sigma^2$ is the Debye Waller factor value; $^d\Delta E_0$ is inner potential correction to account for the difference in the inner potential between the sample and the reference compound; *R* factor indicates the goodness of the fit. $S0^2$ was fixed to 0.879, according to the experimental EXAFS fit of Ru foil by fixing *CN* as the known crystallographic value. *This value was fixed during EXAFS fitting, based on the known structure of Ru. Fitting conditions: *k* range：2.0-10.5; *R* range: 1.0-2.6; fitting space: R space; *k*-weight = 3.

Supplementary Table 11. leaching experiments of CCOFX-Ru in asymmetric transfer hydrogenation of acetophenone.

Entry	Catalyst	t (h)	T (°C)	Addition	Solvent	Yield (%)
1	CCOF0-Ru	1	60	HCOONa	1mL ^{i}PrOH/ H_2O (1:1)	1.6
2	CCOF1-Ru	1	60	HCOONa	1mL ^{i}PrOH/ H_2O (1:1)	0.8
3	CCOF2-Ru	1	60	HCOONa	1mL ^{i}PrOH/ H_2O (1:1)	trace

Reaction conditions: The catalyst was stirred under standard conditions for 1 h in the absence of substrate, filtered, and the filtrate was subsequently reacted with acetophenone under N_2 atmosphere for 1 h.

Supplementary Table 12. Optimization of the reaction conditions and control experiments in asymmetric transfer hydrogenation of acetophenone.

Entry	Catalyst	t (h)	T (°C)	Addition	Solvent	Yield (%)	ee [a] (%)
1	CCOF2-Ru	1	60	HCOONa	1mL ^{i}PrOH/ H_2O (1:1)	>99	95
2	CCOF2-Ru	1	60	HCOONa	1mL H_2O	98	90
3	CCOF2-Ru	1	60	$HCOONH_4$	1mL ^{i}PrOH/ H_2O (1:1)	21	90
4	CCOF2-Ru	1	60	KOH	1mL ^{i}PrOH	65	50
5	CCOF2-amp-Ru	1	60	HCOONa	1mL ^{i}PrOH/ H_2O (1:1)	85	87
6	CCOF2	1	60	HCOONa	1mL ^{i}PrOH/ H_2O (1:1)	n.d. [b]	-
7	TFPB-DATP-Ru	1	60	HCOONa	1mL ^{i}PrOH/ H_2O (1:1)	n.d.	-
8	CCOF2-Ru (0.25%) [c]	1	60	HCOONa	1mL ^{i}PrOH/ H_2O (1:1)	75	95
9	CCOF2-Ru	1	60	HCOONa (2 equiv.)	1mL ^{i}PrOH/ H_2O (1:1)	90	95
10	CCOF2-Ru	1	60	HCOONa (1 equiv.)	1mL ^{i}PrOH/ H_2O (1:1)	67	94
11	CCOF2-Ru	1	60	HCOONa	1mL ^{i}PrOH	41	86
12	CCOF2-Ru	1	60	HCOONa	1mL ACN	18	95

Reaction conditions: ketones (0.2 mmol), addition (1 mmol), catalyst (0.5 mol % of Ru active site based on ICP analysis) under N_2 atmosphere in 1 mL solvent. [a] Determined by chiral HPLC. [b] n.d. = No detection. [c] 0.25 mol % of Ru active site based on ICP analysis.

Supplementary Table 13. Current research status of MCCOFs in asymmetric catalysis

Entry	Catalyst	t (h)	T (℃)	Synthetic strategies	Reaction	Yield (%)	ee (%)	Cycle (ee retention)	Leaching (wt%)	Ref.
1	CCOF19-Ir	72	25	PSM	Asymmetric hydrogenation	96	98	10 (>94%)	1.06 (total over 10)	7
2	(S)-Au-ADC−CCOF	12	r.t.	Direct synthesis	Asymmetric addition reactions of phenylacetylene to aldehyde	95	93	8 (>86%)	Not reported	8
3	CuII@(Λ)-TpPa-1	24	25	PSM	A^3-coupling reaction	99	98	10 (>94%)	Not reported	9
4	CCOF 23-Rh	8	50	PSM	Asymmetric Michael Addition Reaction	86	96	4 (>90%)	Not reported	10
5	Sc(III)@(R)-PBOTF	24	25	PSM	Asymmetric acetalization of 2-aminobenzamides with aldehydes	97	99	5 (>95%)	0.12 (total over 5)	11
6	CCOF 4-Cr-Mn	24	0	Subcomponent self-assembly	Alkene epoxidation/epoxide aminolysis	84	91	5 (>85%)	Not reported	12
7	(Δ)-TpTab-Cu	48	-10	PSM	Henry reaction	10	35	Not reported	Not reported	13
	(R)-Rh@TPB-DMTP-COF	24	40	PSM	Asymmetric transfer hydrogenation	99	96	5 (>93%)	Not reported	14
8	CCOF2-Ru	1	60	PSM	Asymmetric transfer hydrogenation	>99	97	5 (>95%)	0.1 (total over 5)	This work

Supplementary Table 14. The comparison of different heterogeneous catalysts for asymmetric transfer hydrogenation/ asymmetric hydrogenation.

Entry	Catalyst	Metal Loading (mol%)	t (h)	T (°C)	Conditions	Yield (%)	ee (%)	TOF (h^{-1})	Cycle (ee retention)	Leaching (wt%)	Extending t/per run (%)[a]	Ref.
1[b]	CCOF19-Ir	0.1	72	25	1 bar H_2, I_2, toluene	96	98	Not reported	10 (>94%)	1.06 (total over 10)	No extending	7
2[c]	CCOF19-Ru	0.4	120	80	2 bar H_2, EtOH	95	99.9	Not reported	10 (>99%)	Not reported	No extending	7
3[d]	CFLPF1	Metal-Free	48	RT	60 bar H_2, toluene	95	86	Not reported	5 (>80%)	Not reported	No extending	15
4[e]	(R)-39	Metal-Free	20	80	Benzothiazo-line, toluene	87	99	Not reported	5 (>96%)	Not reported	No extending	16
5[f]	(R)-1-PNMe$_2$/Rh	1	4	RT	60 bar H_2, toluene	99	99.2	Not reported	5 (>97%)	Not reported	No extending	17
6[g]	Ir-PDA-6d	0.05	4	60	HCOOH, H_2O/MeOH	95	94	1247	6 (>88%)	< 0.03 (per cycle)	125	18
7[h]	P-C-A1.0-RuOTs	1	12	40	50 bar H_2, EtOH	99	90	90	5 (>85%)	0.3 (total over 5)	No extending	19
8[i]	Ru-TsDPEN @SBA-100	1	2.5	40	HCOONa, H_2O	99	92	126	5 (>90%)	Not reported	220	20
9[i]	MMP@HMSN-0.10 (Rh)	0.5	3	40	HCOONa, H_2O	98	94	991	4 (>90%)	4.8%intotal Rh loading	233	21
10[i]	hybrid microreactor catalyst (Rh)	0.5	1.5	40	HCOONa, H_2O	98	95	625	5 (>90%)	6.8% of Rh loading	550	22
11[j]	Mesitylene RuArDPEN-MSNs	2	16	40	HCOONa, H_2O	99	96	Not reported	8 (>91%)	Not reported	No extending	23
12[i]	(R)-Rh@TPB-DMTP-COF	2.36	24	40	HCOOH/ HCOONa/ H_2O	99	96	Not reported	5 (>93%)	Not reported	No extending	14
**13[i]**	**CCOF2-Ru**	**0.5**	**1**	**60**	**HCOONa, ^{i}PrOH/ H_2O**	**>99**	**95**	**1104**	**5 (>95%)[k]**	**0.1 (total over 5)**	**12.5**	**This work**

$$^{a}\ \text{Extending t/per run} = \frac{(\text{Total cycle time} - \text{Reaction time of first run} \times \text{Number of cycles})}{\text{Reaction time of first run} \times (\text{Number of cycles} - 1)} \times 100\%$$

Substrate: [b] 6-chloro-2-methylquinoline; [c] methyl 3-oxo-3-phenylpropanoate; [d] methyl 2-phenylacrylate; [e] N-(4-Methoxyphenyl)-1-phenylethan-1-imine; [f] N-(1-phenylvinyl)acetamide; [g] Dihydro-4,4-dimethyl-2,3-furandione; [h] 2-Methylquinoline; [i] Phenylacetone; [j] 4-methoxy-N-(2,2,2-trifluoro-1-(4 fluorophenyl)ethylidene)aniline; [k] 5,7-Difluorochroman-4-one.

- Entries 1-2: From Zheng et al., *J. Am. Chem. Soc.*; MCCOF for asymmetric hydrogenation. High ee but long reaction time.[7]
- Entry 3: From Zhang et al., *J. Am. Chem. Soc.*; CCOF for asymmetric hydrogenation. Good ee but long reaction time, using hazardous high-pressure hydrogen.[15]
- Entry 4: From Hou et al., *J. Am. Chem. Soc.*; CCOF for asymmetric hydrogenation. High ee but long reaction time.[16]
- Entry 5: From Gong et al., *J. Am. Chem. Soc.*; CMOF for asymmetric hydrogenation. High ee but using hazardous high-pressure hydrogen.[17]
- Entry 6: From Lin et al., *J. Am. Chem. Soc.*; Polymer Catalyst for asymmetric transfer hydrogenation. High ee, high TOF, low metal loading, minimal leaching but 125% extension in reaction time per cycle during catalytic recycling.[18]
- Entry 7: From Tao et al., *ACS Catalysis*; Polymer Catalyst for asymmetric hydrogenation. High ee, but low TOF and using hazardous high-pressure hydrogen.[19]
- Entry 8-: From Bai et al., *Chem Commun*; Polymer Catalyst for asymmetric transfer hydrogenation. High ee but low TOF and 220% extension in reaction time per cycle during catalytic recycling.[20]
- Entry 9: From Jing et al., *Catalysis Science & Technology*; Polymer Catalyst for asymmetric transfer hydrogenation. High ee, high TOF but 233% extension in reaction time per cycle during catalytic recycling.[21]
- Entry 10: From Zhang et al., *ACS Catalysis*; Polymer Catalyst for asymmetric transfer hydrogenation. High ee but low TOF and 550% extension in reaction time per cycle during catalytic recycling.[22]
- Entry 11: From Zhang et al., *ACS Catalysis*; Polymer Catalyst for asymmetric transfer hydrogenation. High ee but high metal loading and gradual decrease yield in cyclic reactions.[23]
- Entry 12: From Wang et al., *Small*; MCCOF for asymmetric transfer hydrogenation. High ee but high metal loading and long reaction time.[14]
- Advantages: CCOF2-Ru achieves a TOF of 1104 h^{-1}, surpasses most reported polymer-based catalyst (Entries 7-10), with superior cycle stability (5 cycles,>95ee) and low activity loss in cyclic reactions (12.5% extension in reaction time per cycle vs >100% extension in reaction time per cycle, Entries 6,8-10).

Supplementary Table 15. Ru content determined by ICP-OES.

Cycles	Ru content (wt%)		
	CCOF2-Ru (0.89)	CCOF1-Ru (1.62)	CCOF0-Ru (2.12)
1	0.87	1.54	1.64
2	0.84	1.4	1.22
3	0.83	1.27	1.10
4	0.80	-	-
5	0.79	-	-

Section 5. HPLC Chromatograms and NMR Spectra of Catalytic Products

All racemic standards used for chiral HPLC analysis were prepared by reduction of the corresponding ketone precursors with $NaBH_4$ in methanol, and their structures were confirmed by NMR spectroscopy.

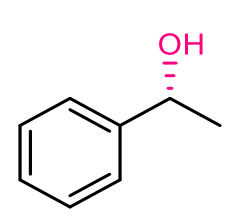

(*R*)-(+)-1-Phenylethanol (1a). (Known compound, see: Chem. 2024, 10, 250–264):

The product **1a** was purified by flash chromatography on silica gel (300-400 mesh). ^{1}H NMR (600 MHz, DMSO-D_6) δ 7.36 – 7.28 (dtd, *J* = 15.4, 7.4, 1.8 Hz, 4H), 7.23 – 7.18 (m, 1H), 5.16 – 5.12 (dd, *J* = 4.2, 1.6 Hz, 1H), 4.73 – 4.67 (qd, *J* = 6.5, 4.6 Hz, 1H), 1.33 – 1.30 (dd, *J* = 6.5, 1.6 Hz, 3H).

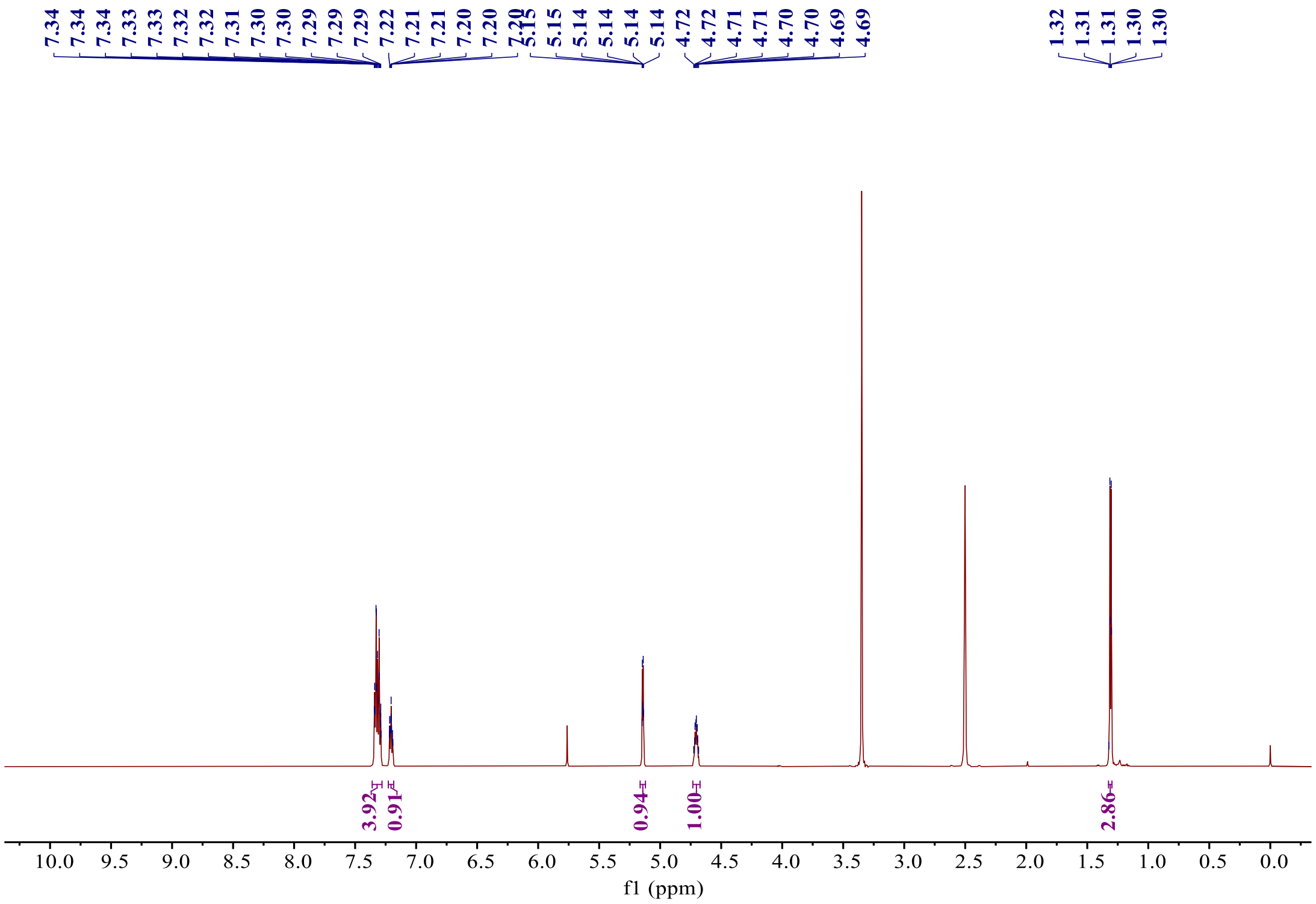

The ee-value of **1a** was determined by HPLC analysis with a 25 cm × 4.6 mm Daicel Chiralcel ID column (hexane/i-PrOH = 99/1, 1.0 mL/min). t_{minor} = 15.6 min, t_{major} = 14.6 min; ee = 95%.

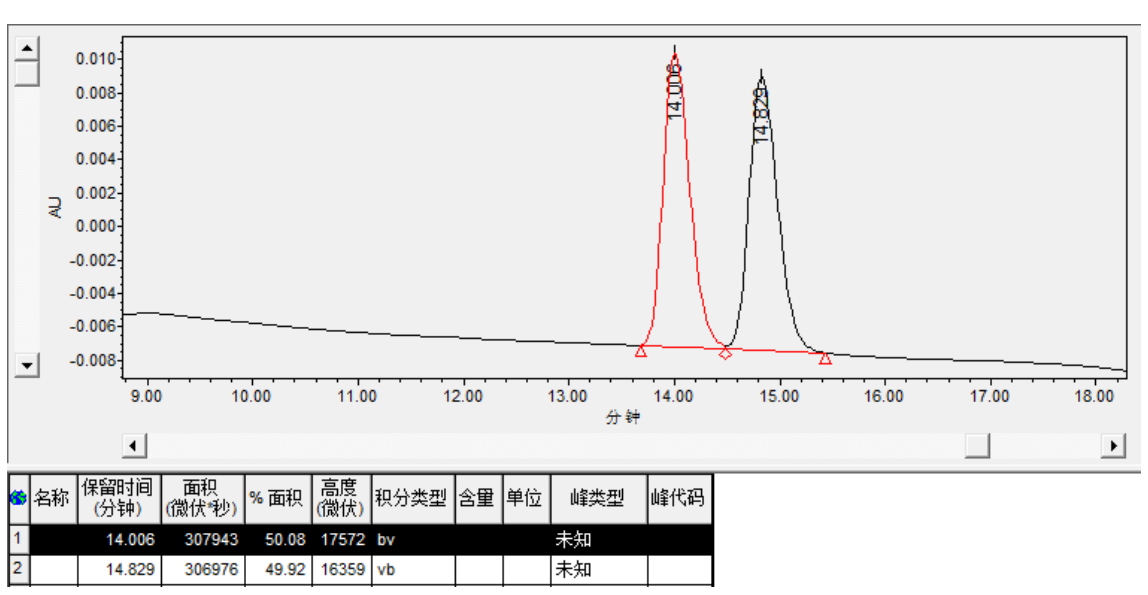

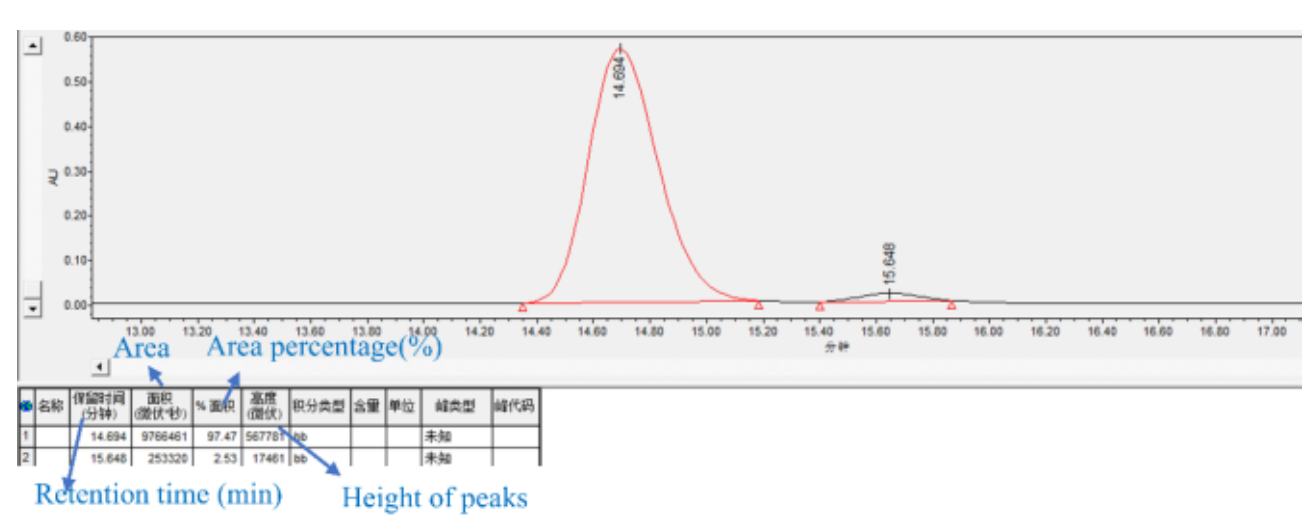

Peaks number	Retention time (min)	Area	Area percentage (%)	Height of peaks
1	14.694	9766461	97.47	567781
2	15.648	253320	2.53	17461

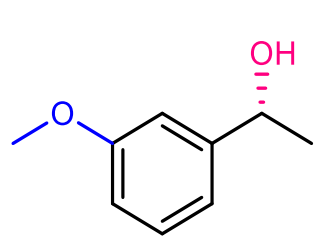

(*R*)-1-(3-methoxyphenyl)ethan-1-ol (1b). (Known compound, see: Journal of Organometallic Chemistry (1988), 346(3), 413-24):

The product **1b** was purified by flash chromatography on silica gel (300-400 mesh). ^{1}H NMR (600 MHz, CHLOROFORM-*D*) δ 7.29 – 7.23 (t, *J* = 8.1 Hz, 1H), 6.97 – 6.91 (m, 2H), 6.84 – 6.78 (ddd, *J* = 8.3, 2.5, 1.1 Hz, 1H), 4.89 – 4.83 (q, *J* = 6.5 Hz, 1H), 3.82 – 3.79 (s, 3H), 2.01 – 1.99 (s, 1H), 1.49 – 1.46 (d, *J* = 6.5 Hz, 3H).

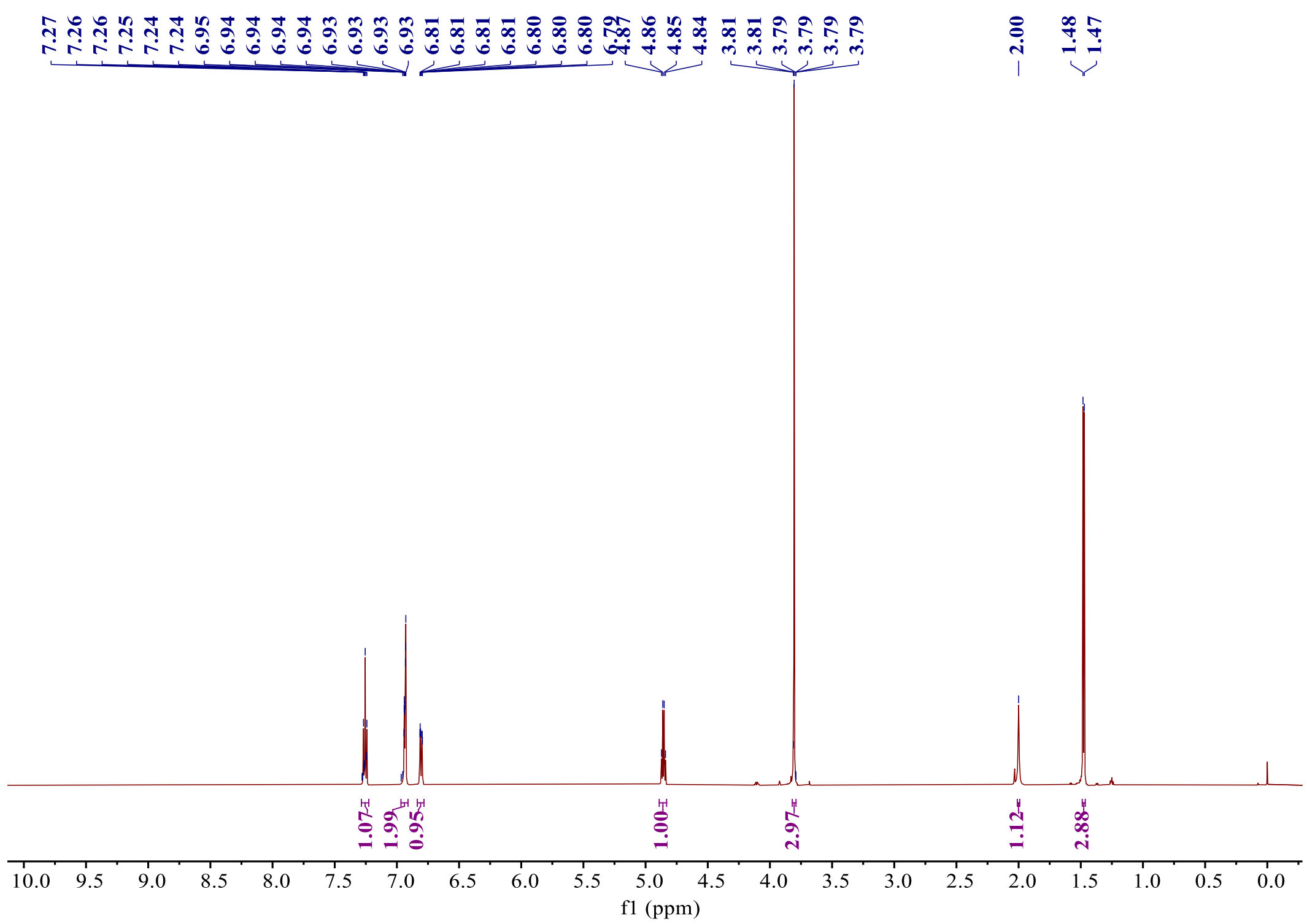

The ee value of **1b** was determined by HPLC analysis with a 25 cm × 4.6 mm Daicel Chiralcel ID column (hexane/i-PrOH = 99/1, 1.0 mL/min). t_{minor} = 38.2 min, t_{major} = 36.0 min; ee = 92%.

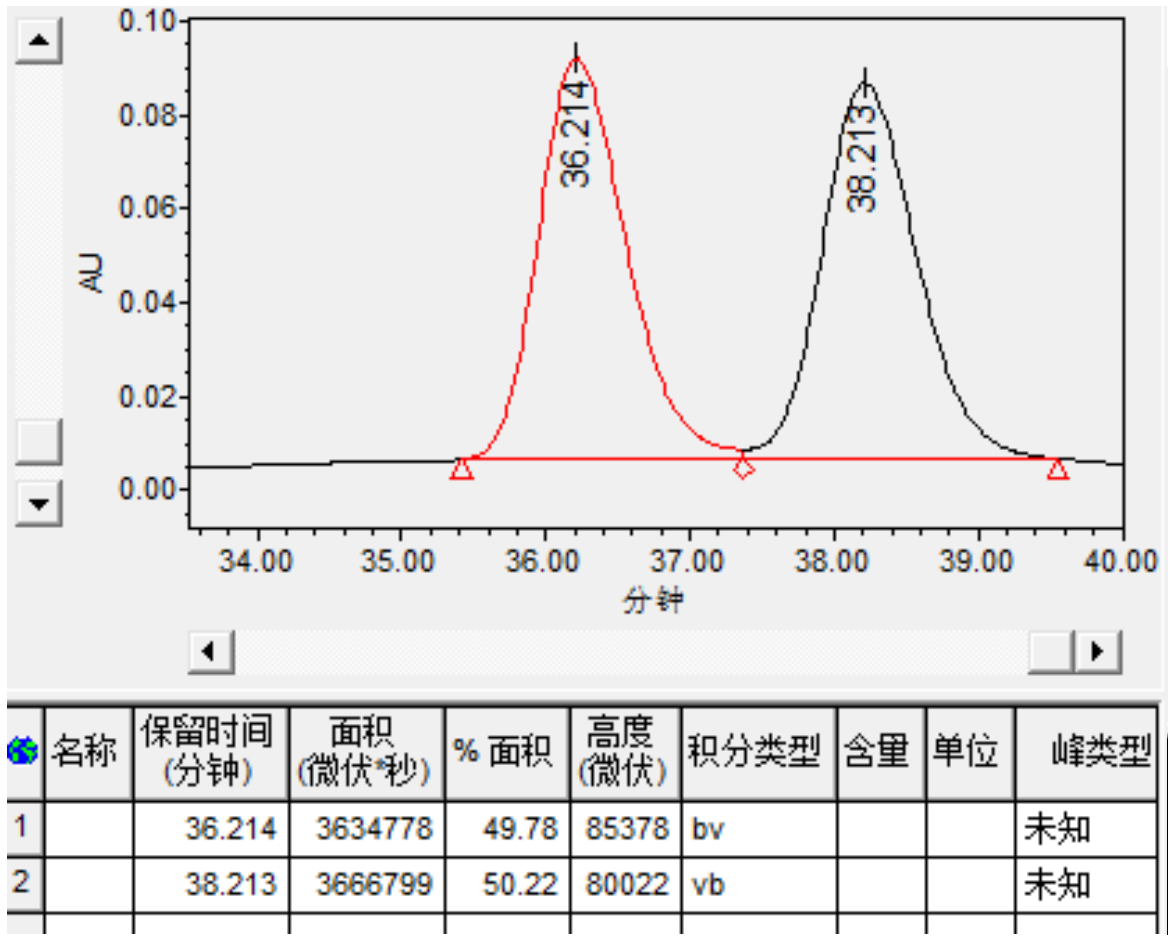

	名称	保留时间 (分钟)	面积 (微伏*秒)	% 面积	高度 (微伏)	积分类型	含量	单位	峰类型
1		36.214	3634778	49.78	85378	bv			未知
2		38.213	3666799	50.22	80022	vb			未知

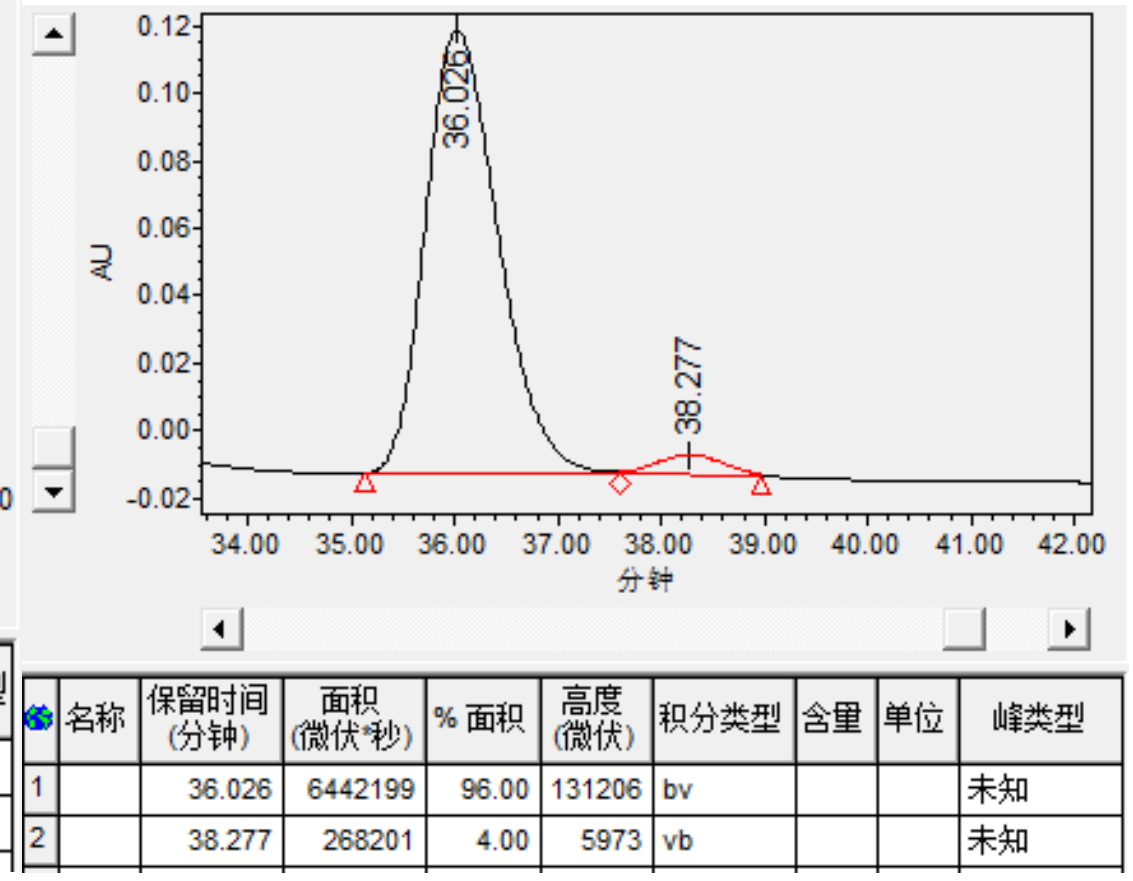

	名称	保留时间 (分钟)	面积 (微伏*秒)	% 面积	高度 (微伏)	积分类型	含量	单位	峰类型
1		36.026	6442199	96.00	131206	bv			未知
2		38.277	268201	4.00	5973	vb			未知

Peaks number	Retention time (min)	Area	Area percentage (%)	Height of peaks
1	36.026	6442199	96.00	131206
2	38.277	268201	4.00	5973

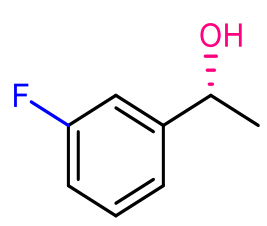

(*R*)-1-(3-fluorophenyl)ethan-1-ol (1c). (Known compound, see: Bioorganic Chemistry (1988), 16(4), 388-93):

The product **1c** was purified by flash chromatography on silica gel (300-400 mesh). ^{1}H NMR (600 MHz, CHLOROFORM-*D*) δ 7.32 – 7.28 (td, *J* = 7.9, 5.8 Hz, 1H), 7.14 – 7.11 (ddt, *J* = 7.7, 1.6, 0.8 Hz, 1H), 7.11 – 7.08 (dt, *J* = 9.9, 2.2 Hz, 1H), 6.97 – 6.93 (tdd, *J* = 8.4, 2.6, 1.0 Hz, 1H), 4.91 – 4.87 (q, *J* = 6.5 Hz, 1H), 1.98 – 1.95 (s, 1H), 1.49 – 1.47 (d, *J* = 6.5 Hz, 3H).

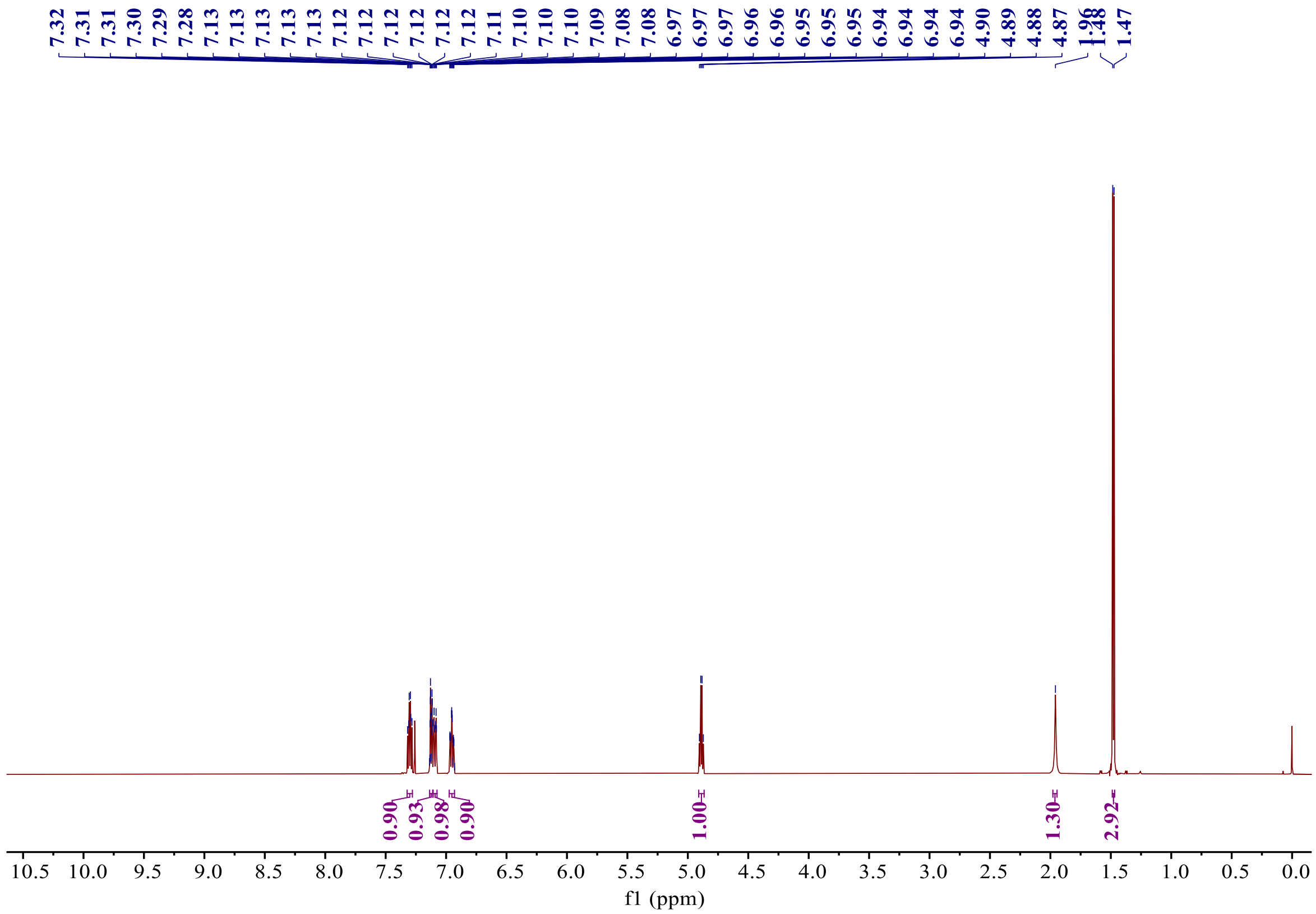

The ee-value of **1c** was determined by HPLC analysis with a 25 cm × 4.6 mm Daicel Chiralcel ID column (hexane/i-PrOH = 98/2, 1.0 mL/min). t_{minor} = 10.9 min, t_{major} = 9.8 min; ee = 90%.

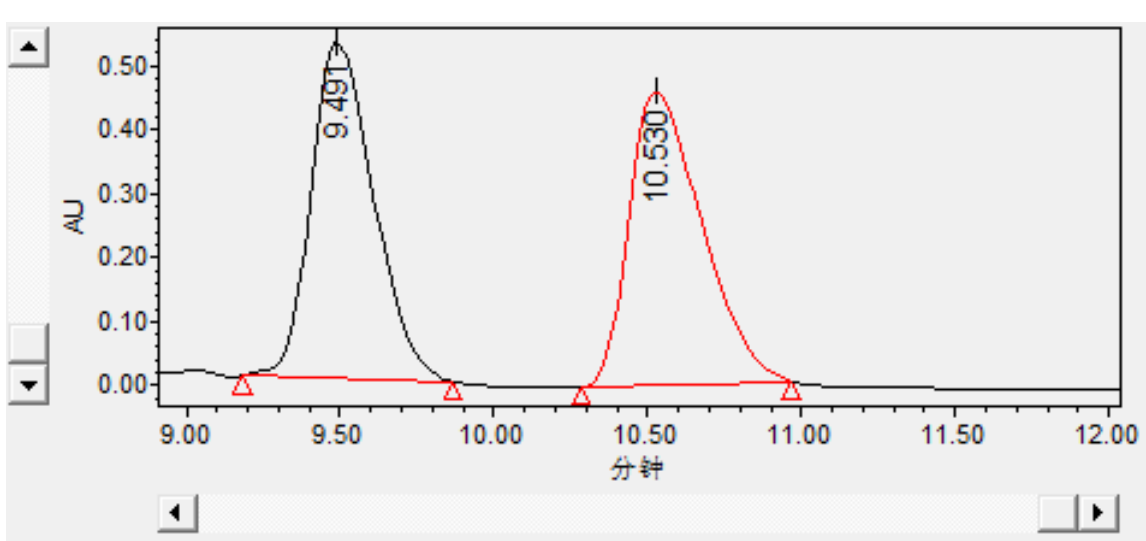

	名称	保留时间 (分钟)	面积 (微伏*秒)	% 面积	高度 (微伏)	积分类型	含量	单位	峰类型	峰代码
1		9.491	7458609	49.75	526128	bb			未知	
2		10.530	7534520	50.25	457428	bb			未知	

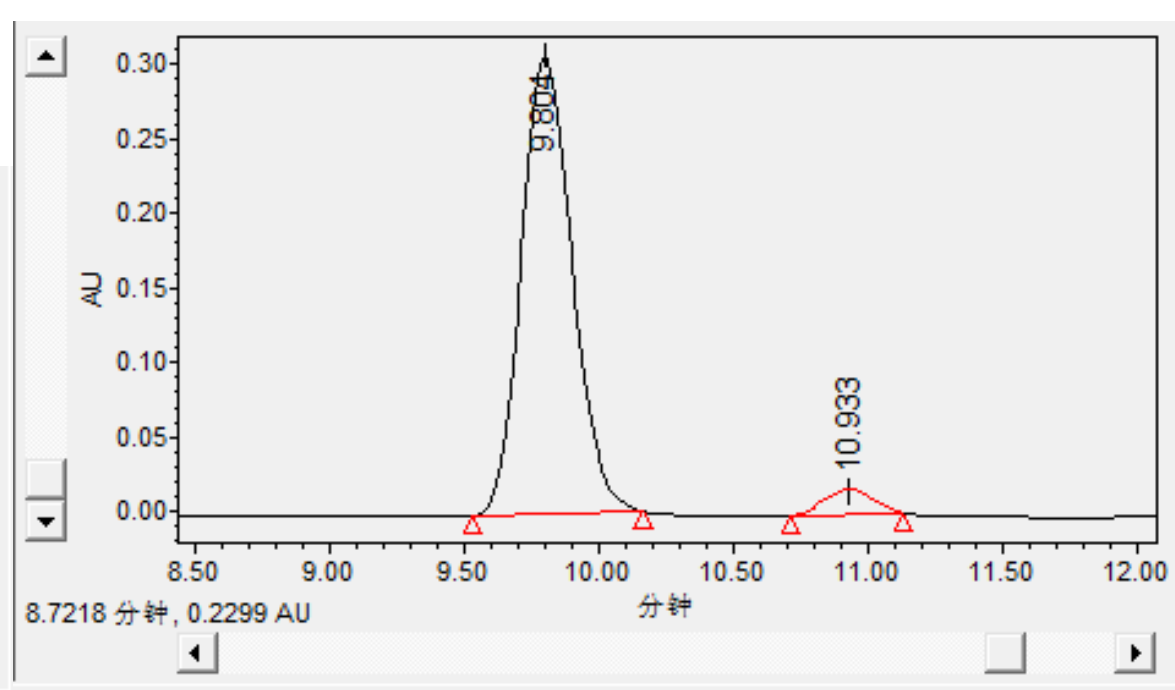

	名称	保留时间 (分钟)	面积 (微伏*秒)	% 面积	高度 (微伏)	积分类型	含量	单位	峰类型	峰代码
1		9.804	4022771	95.07	308196	bb			未知	
2		10.933	208820	4.93	16609	bb			未知	

Peaks number	Retention time (min)	Area	Area percentage (%)	Height of peaks
1	9.804	4022771	95.07	308196
2	10.933	208820	4.93	16609

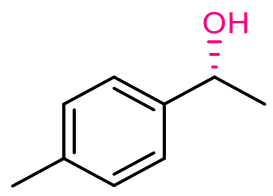

(*R*)-1-(p-tolyl)ethan-1-ol (1d): (Known compound, see: Journal of Organic Chemistry (2003), 68(2), 402-406):

The product **1d** was purified by flash chromatography on silica gel (300-400 mesh). ^{1}H NMR (600 MHz, CHLOROFORM-*D*) δ 7.27 – 7.24 (m, 2H), 7.17 – 7.14 (d, *J* = 7.8 Hz, 2H), 4.88 – 4.83 (q, *J* = 6.5 Hz, 1H), 2.35 – 2.33 (s, 3H), 1.87 – 1.83 (s, 1H), 1.49 – 1.46 (d, *J* = 6.5 Hz, 3H).

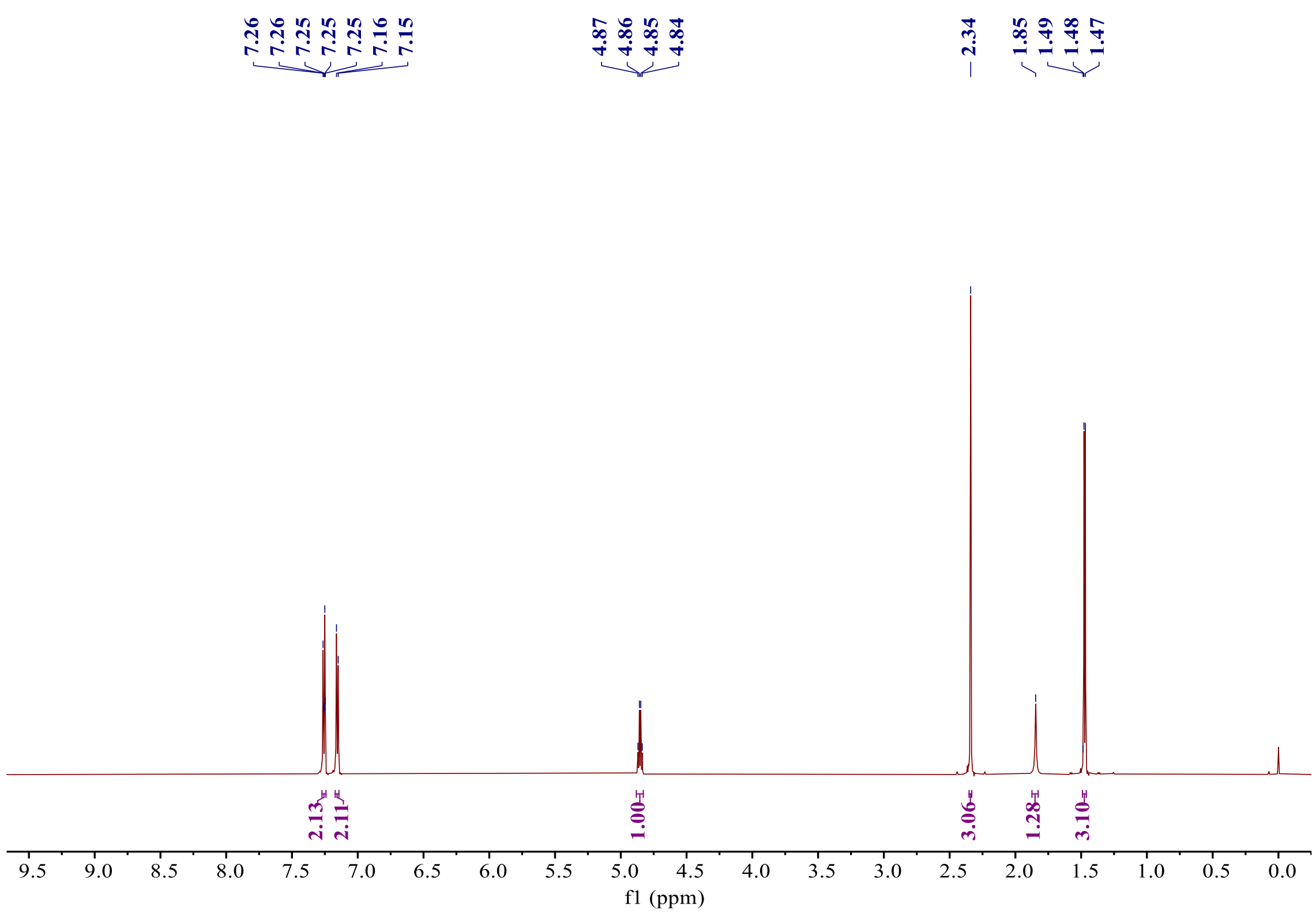

The ee-value of **1d** was determined by HPLC analysis with a 25 cm × 4.6 mm Daicel Chiralcel ID column (hexane/i-PrOH = 99/1, 1.0 mL/min). t_{minor} = 16.3 min, t_{major} = 15.4 min; ee = 91%.

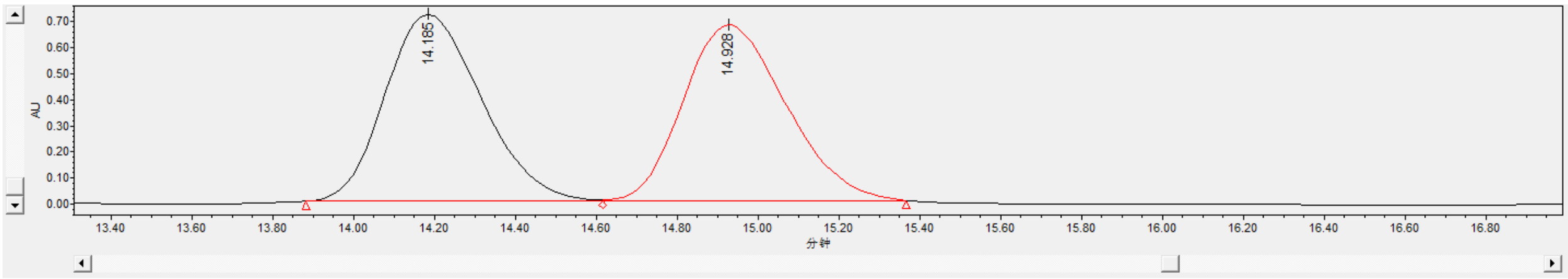

	名称	保留时间 (分钟)	面积 (微伏*秒)	% 面积	高度 (微伏)	积分类型	含量	单位	峰类型	峰代码
1		14.185	11849760	49.95	716268	bv			未知	
2		14.928	11874361	50.05	674711	vb			未知	

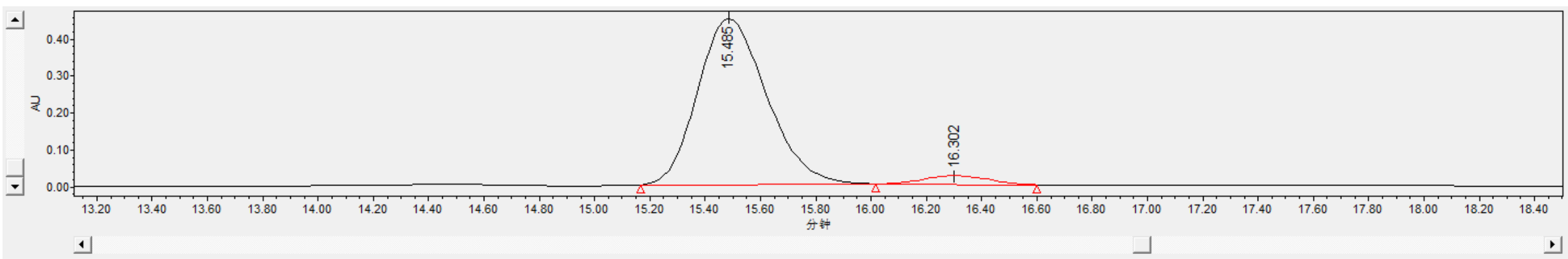

	名称	保留时间 (分钟)	面积 (微伏*秒)	% 面积	高度 (微伏)	积分类型	含量	单位	峰类型	峰代码
1		15.485	7766118	95.47	449958	bb			未知	
2		16.302	368706	4.53	23247	bb			未知	

Peaks number	Retention time (min)	Area	Area percentage (%)	Height of peaks
1	15.485	7766118	95.47	449958
2	16.302	368706	4.53	23247

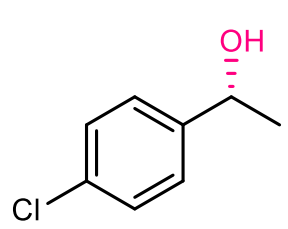

(*R*)-1-(4-chlorophenyl)ethan-1-ol (1e). (Known compound, see: Chem. 2024, 10, 250–264):

The product **1a** was purified by flash chromatography on silica gel (300-400 mesh). ^{1}H NMR (600 MHz, DMSO-D_6) δ 7.38 – 7.35 (s, 4H), 5.28 – 5.25 (d, *J* = 4.3 Hz, 1H), 4.75 – 4.70 (qd, *J* = 6.5, 4.2 Hz, 1H), 1.32 – 1.30 (d, *J* = 6.5 Hz, 3H).

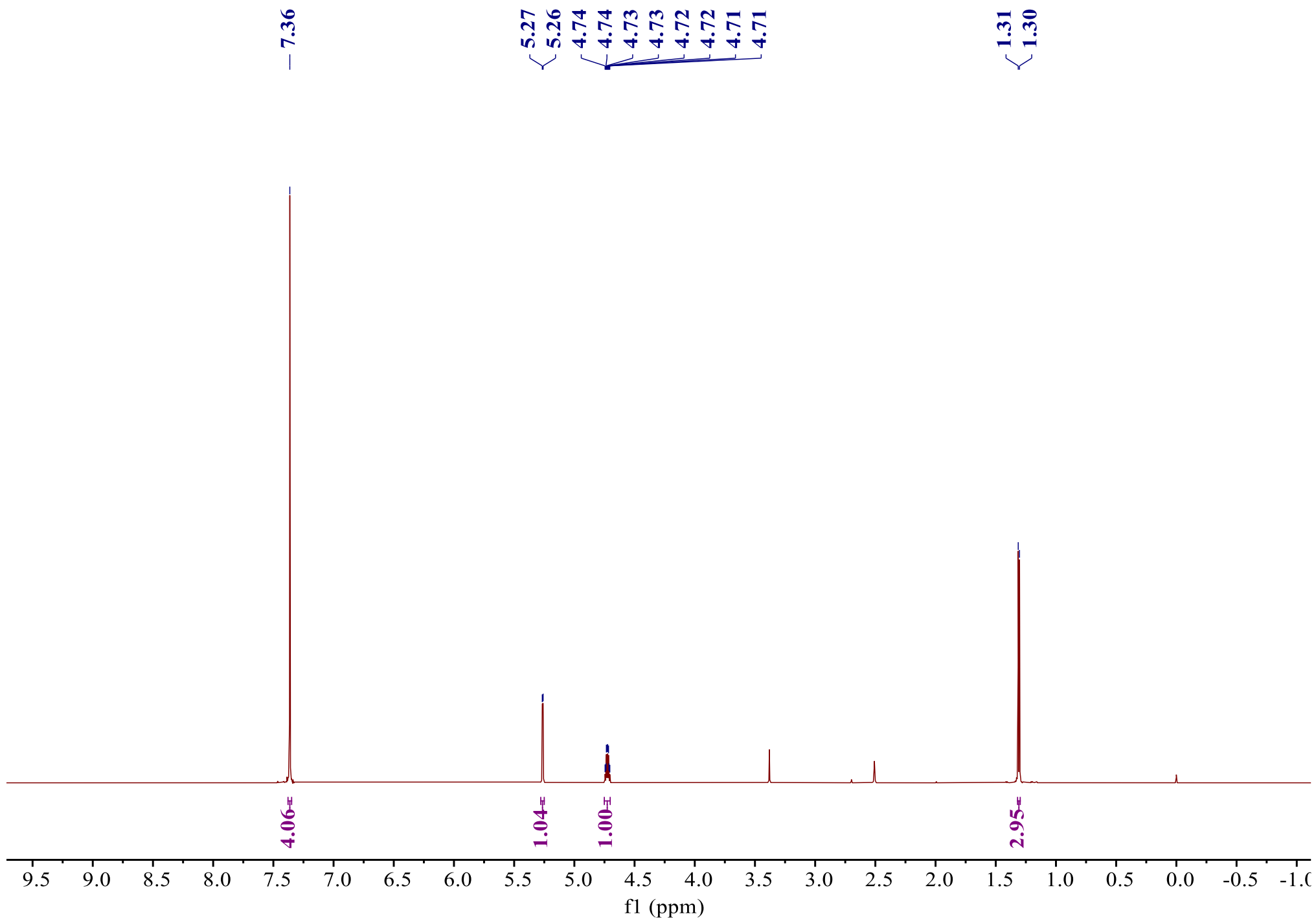

The ee-value of **1e** was determined by HPLC analysis with a 25 cm × 4.6 mm Daicel Chiralcel IC column (hexane/i-PrOH = 99/1, 1.0 mL/min). t_{minor} = 15.8 min, t_{major} = 14.9 min; ee = 88%.

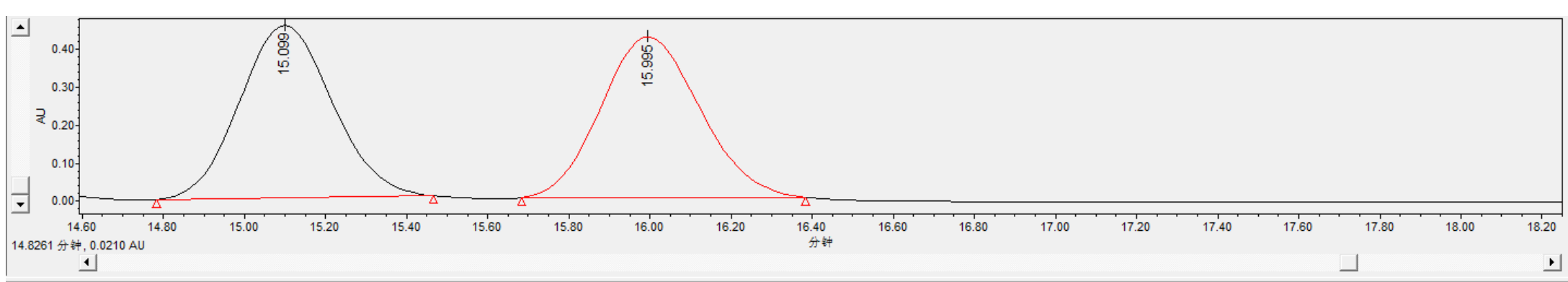

	名称	保留时间 (分钟)	面积 (微伏*秒)	% 面积	高度 (微伏)	积分类型	含量	单位	峰类型	峰代码
1		15.099	7234056	50.10	454662	bb			未知	
2		15.995	7204137	49.90	424727	bb			未知	

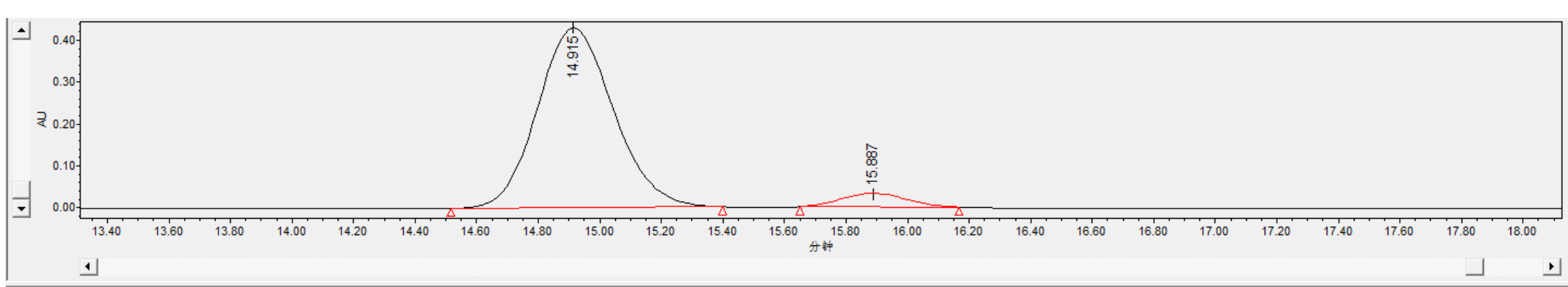

	名称	保留时间 (分钟)	面积 (微伏*秒)	% 面积	高度 (微伏)	积分类型	含量	单位	峰类型	峰代码
1		14.915	7397958	93.96	431031	bb			未知	
2		15.887	475799	6.04	32247	bb			未知	

Peaks number	Retention time (min)	Area	Area percentage (%)	Height of peaks
1	14.915	7397958	93.96	431031
2	15.887	475799	6.04	32247

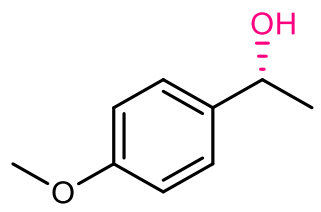

(*R*)-1-(4-methoxyphenyl)ethan-1-ol (1f): (Known compound, see: Journal of the American Chemical Society (2004), 126(4), 986-987).

The product **1f** was purified by flash chromatography on silica gel (300-400 mesh). ^{1}H NMR (600 MHz, CHLOROFORM-*D*) δ 7.32 – 7.28 (m, 2H), 6.90 – 6.87 (m, 2H), 4.88 – 4.82 (q, *J* = 6.4 Hz, 1H), 3.82 – 3.79 (s, 3H), 1.83 – 1.80 (s, 1H), 1.49 – 1.46 (d, *J* = 6.5 Hz, 3H).

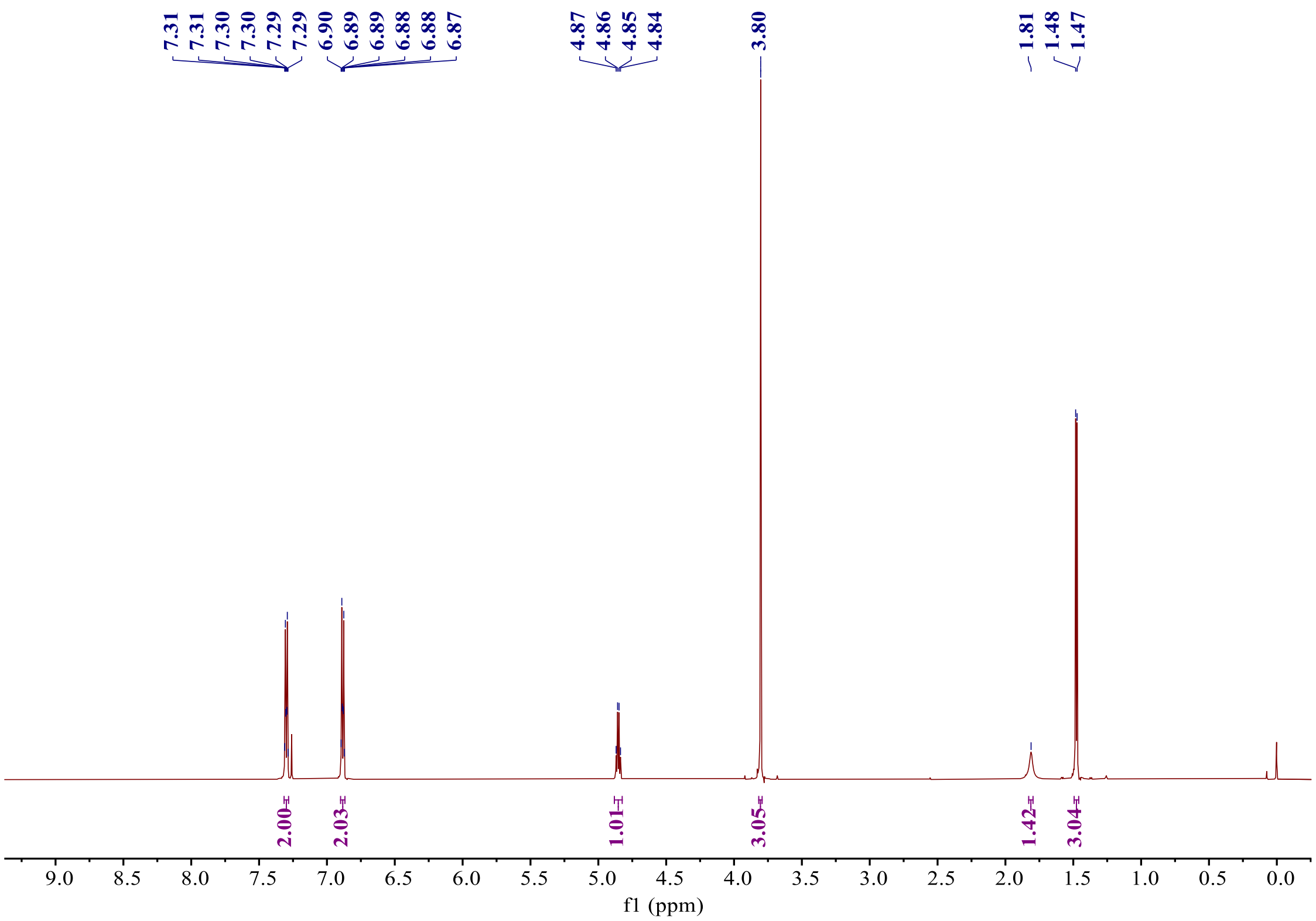

The ee. value of **1f** was determined by HPLC analysis with a 25 cm × 4.6 mm Daicel Chiralcel ID column (hexane/i-PrOH = 99/1, 1.0 mL/min). t_{minor} = 32.4 min, t_{major} = 30.7 min; ee = 91%.

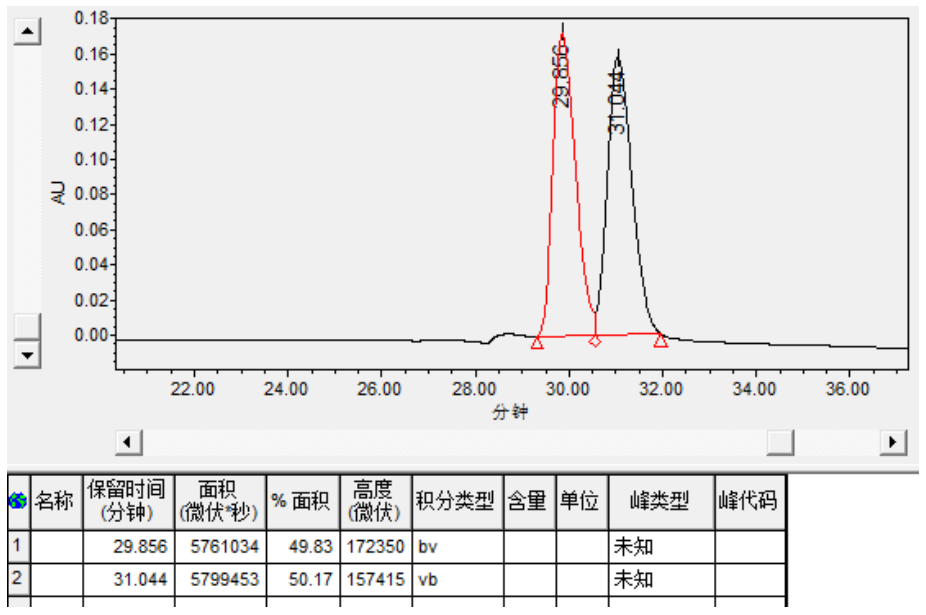

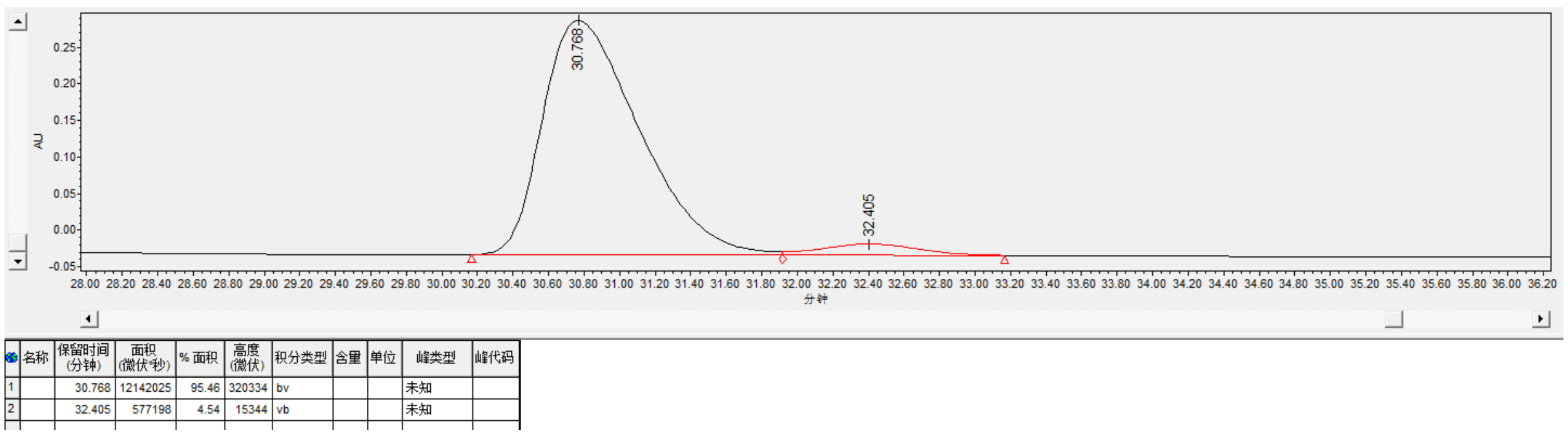

Peaks number	Retention time (min)	Area	Area percentage (%)	Height of peaks
1	30.768	12142025	95.46	320334
2	32.405	577198	4.54	15344

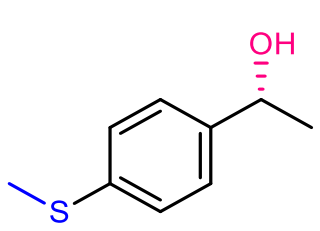

(*R*)-1-(4-(methylthio)phenyl)ethan-1-ol (1g): (Known compound, see: Journal of Organic Chemistry (2004), 69(20), 6860-6866).

The product **1g** was purified by flash chromatography on silica gel (300-400 mesh). ^{1}H NMR (600 MHz, CHLOROFORM-*D*) δ 7.30 – 7.27 (m, 2H), 7.25 – 7.22 (m, 2H), 4.87 – 4.82 (q, *J* = 6.5 Hz, 1H), 2.49 – 2.46 (s, 3H), 1.96 – 1.90 (s, 1H), 1.49 – 1.46 (d, *J* = 6.5 Hz, 3H).

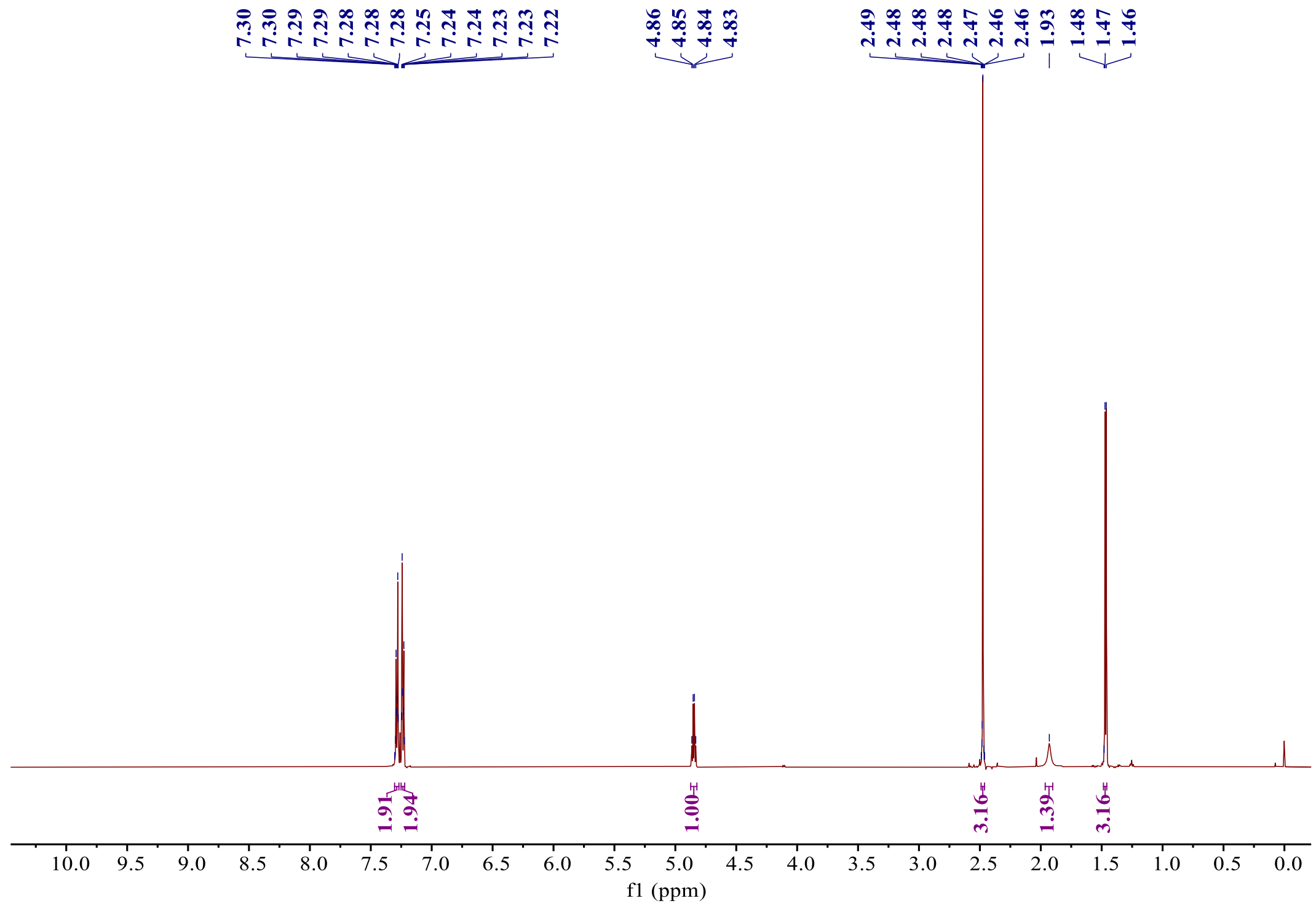

The ee. value of **1g** was determined by HPLC analysis with a 25 cm × 4.6 mm Daicel Chiralcel IC column (hexane/i-PrOH = 97/3, 1.0 mL/min). t_{minor} = 32.4 min, t_{major} = 30.7 min; ee = 91%.

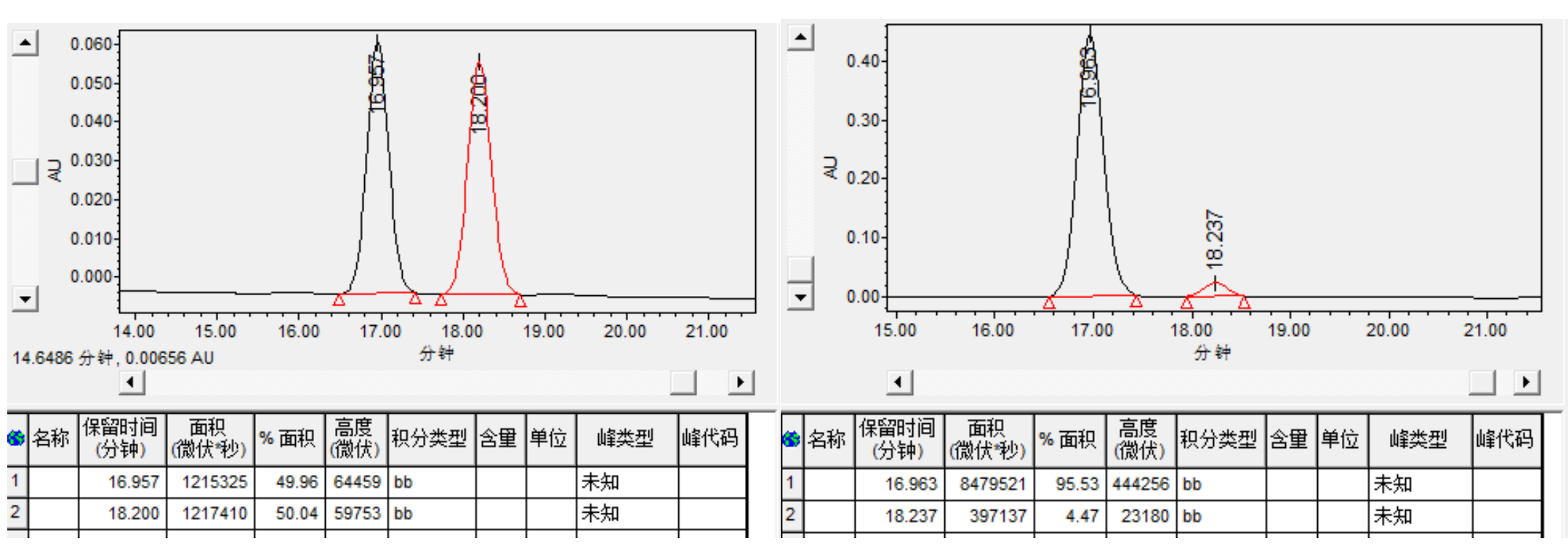

Peaks number	Retention time (min)	Area	Area percentage (%)	Height of peaks
1	16.963	8479521	95.53	444256
2	18.237	397137	4.47	23180

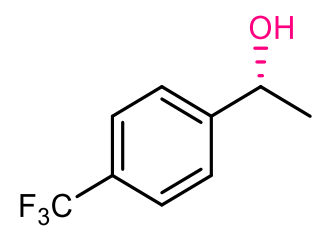

(*R*)-1-(4-(trifluoromethyl)phenyl)ethan-1-ol (1h). (Known compound, see: Organic Letters (2000), 2(26), 4173-4176):

The product **1h** was purified by flash chromatography on silica gel (300-400 mesh). ^{1}H NMR (600 MHz, CHLOROFORM-*D*) δ 7.63 – 7.59 (m, 2H), 7.51 – 7.47 (m, 2H), 4.99 – 4.95 (q, *J* = 6.5 Hz, 1H), 1.86 – 1.81 (s, 1H), 1.52 – 1.50 (d, *J* = 6.5 Hz, 3H).

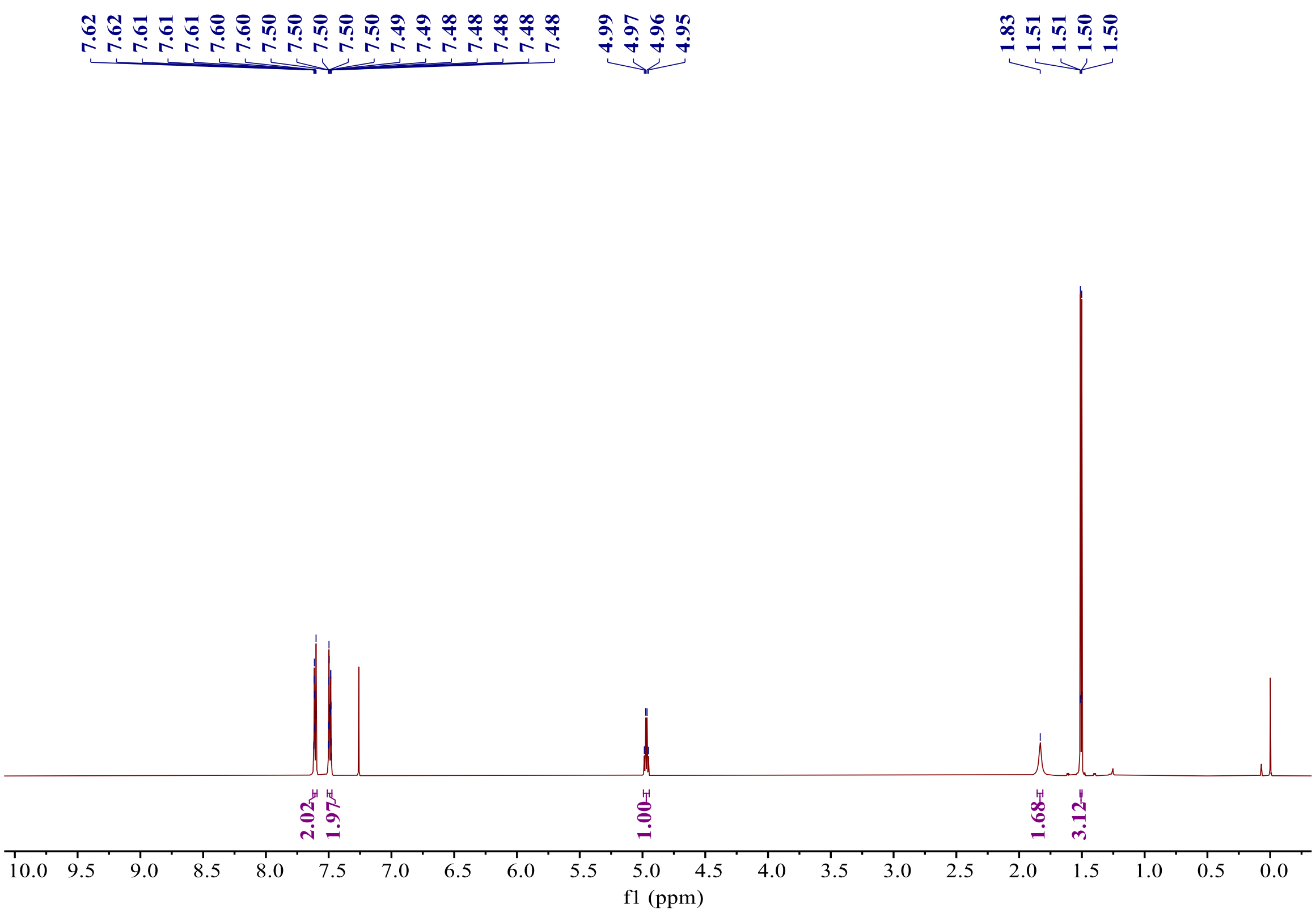

The ee. value of **1h** was determined by HPLC analysis with a 25 cm × 4.6 mm Daicel Chiralcel IC column (hexane/i-PrOH = 99/1, 1.0 mL/min). t_{minor} = 10.2 min, t_{major} = 9.3 min; ee = 86%.

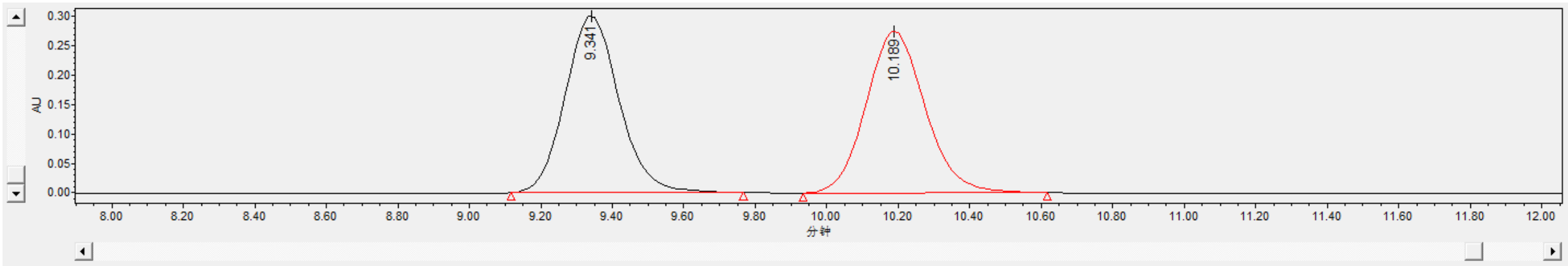

	名称	保留时间 (分钟)	面积 (微伏*秒)	% 面积	高度 (微伏)	积分类型	含量	单位	峰类型	峰代码
1		9.341	3146768	49.93	301391	bb			未知	
2		10.189	3155298	50.07	275372	bb			未知	

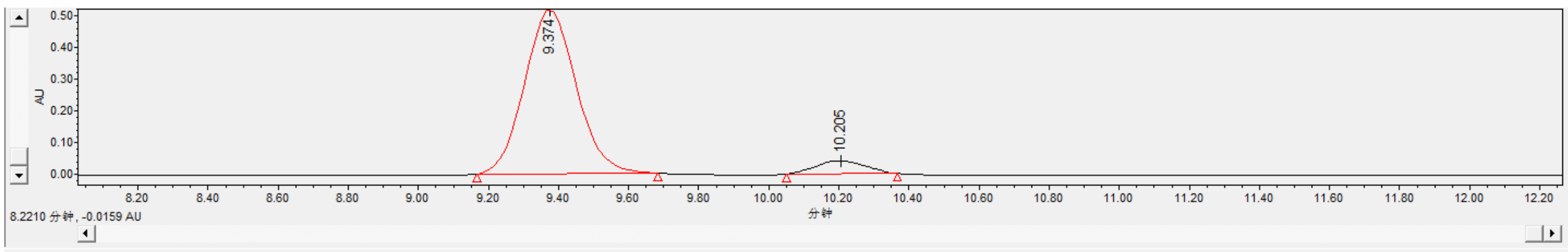

	名称	保留时间 (分钟)	面积 (微伏*秒)	% 面积	高度 (微伏)	积分类型	含量	单位	峰类型	峰代码
1		9.374	5290388	93.04	516376	bb			未知	
2		10.205	395654	6.96	40990	bb			未知	

Peaks number	Retention time (min)	Area	Area percentage (%)	Height of peaks
1	9.374	5290388	93.04	516376
2	10.205	395654	6.96	40990

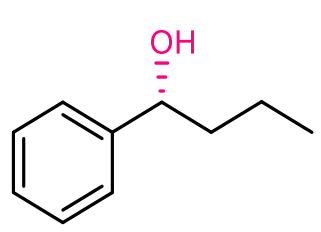

(*R*)-1-phenylbutan-1-ol (1i). (Known compound, see: Journal of Organic Chemistry (1988), 53(6), 1231-8):

The product **1i** was purified by flash chromatography on silica gel (300-400 mesh). ^{1}H NMR (600 MHz, CHLOROFORM-*D*) δ 7.36 – 7.34 (d, *J* = 4.9 Hz, 4H), 7.30 – 7.26 (m, 1H), 4.69 – 4.66 (dd, *J* = 7.6, 5.8 Hz, 1H), 1.84 – 1.82 (s, 1H), 1.82 – 1.64 (m, 2H), 1.49 – 1.27 (m, 2H), 0.96 – 0.92 (t, *J* = 7.4 Hz, 3H).

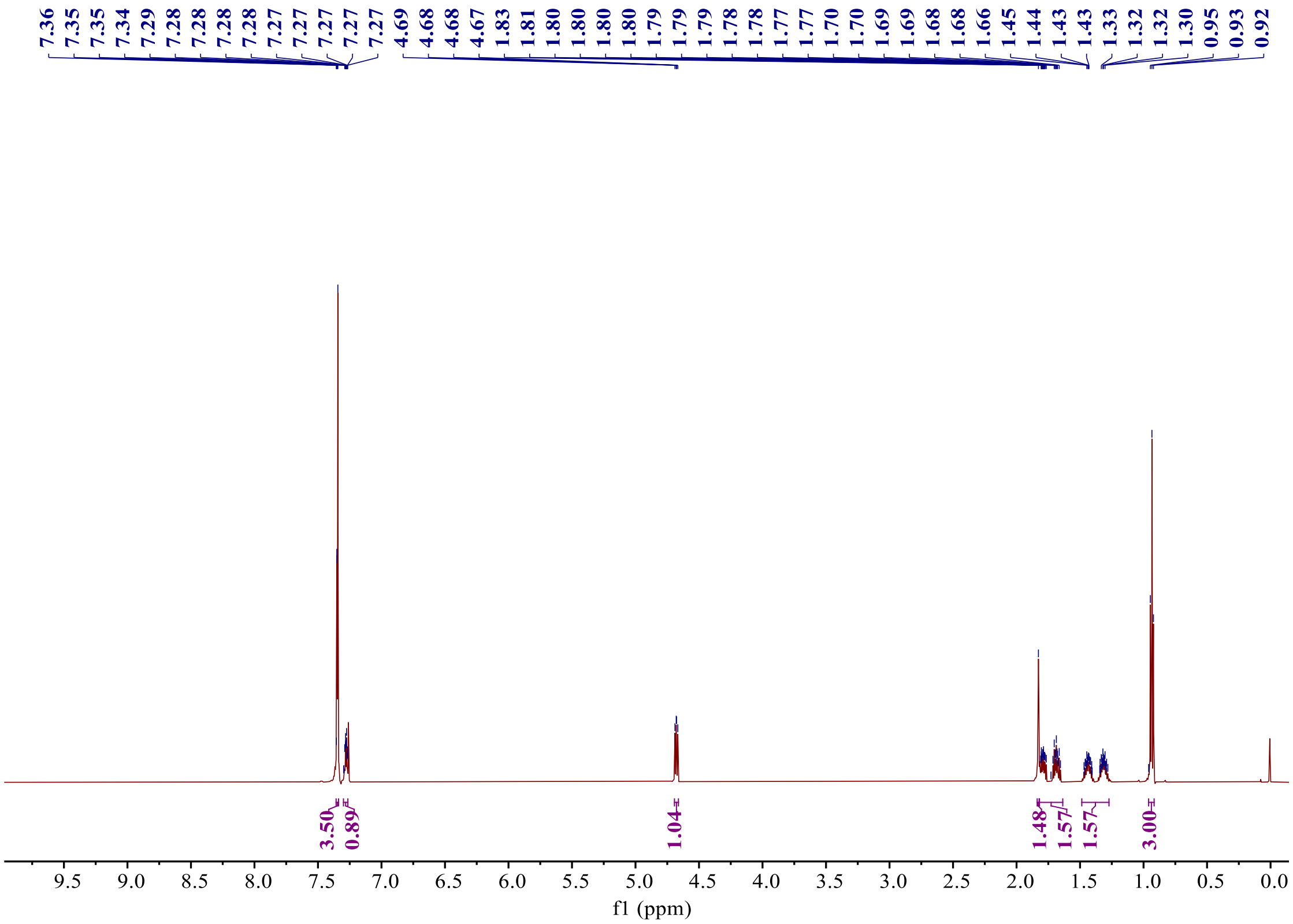

The ee-value of **1i** was determined by HPLC analysis with a 25 cm × 4.6 mm Daicel Chiralcel ID column (hexane/i-PrOH = 99/1, 1.0 mL/min). t_{minor} = 13.2 min, t_{major} = 13.6 min; ee = 87%.

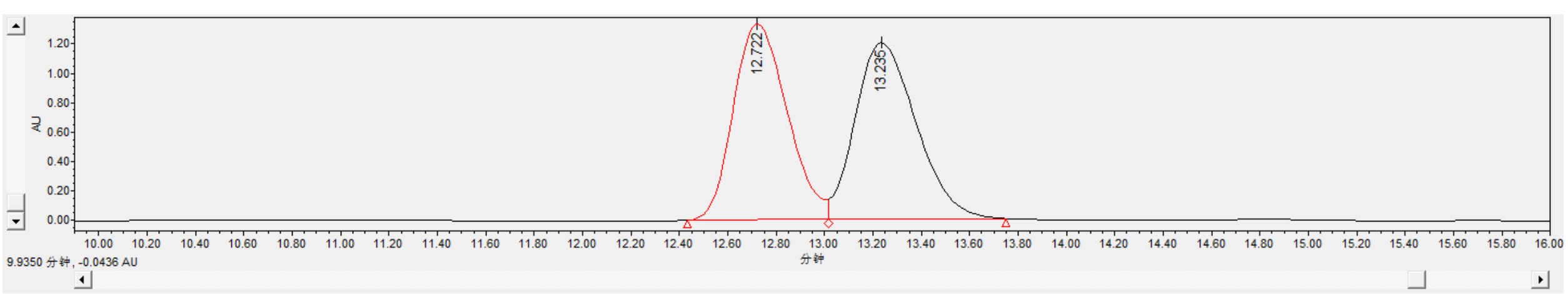

	名称	保留时间 (分钟)	面积 (微伏*秒)	% 面积	高度 (微伏)	积分类型	含量	单位	峰类型	峰代码
1		12.722	20602047	49.52	1335386	bv			未知	
2		13.235	20998746	50.48	1202976	vb			未知	

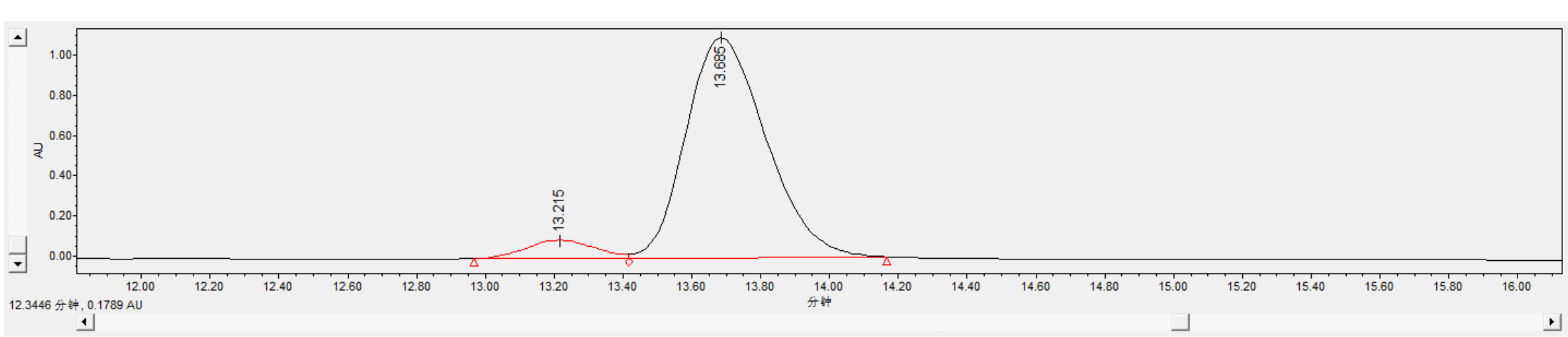

	名称	保留时间 (分钟)	面积 (微伏*秒)	% 面积	高度 (微伏)	积分类型	含量	单位	峰类型	峰代码
1		13.215	1238391	6.53	90353	bv			未知	
2		13.685	17712257	93.47	1095446	vb			未知	

Peaks number	Retention time (min)	Area	Area percentage (%)	Height of peaks
1	13.215	1238391	6.53	90353
2	13.685	17712257	93.47	1095446

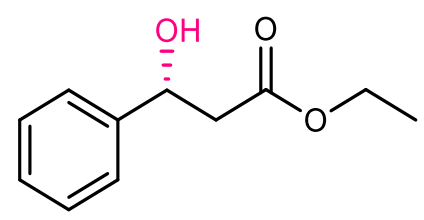

ethyl (*R*)-3-hydroxy-3-phenylpropanoate (1j). (Known compound, see: J. Am. Chem. Soc. 2023, 145, 11, 6100–6111):

The product **1j** was purified by flash chromatography on silica gel (300-400 mesh). ^{1}H NMR (600 MHz, CHLOROFORM-*D*) δ 7.40 – 7.34 (m, 4H), 7.31 – 7.27 (m, 1H), 5.17 – 5.11 (m, 1H), 4.22 – 4.16 (qd, *J* = 7.2, 0.9 Hz, 2H), 3.31 – 3.26 (m, 1H), 2.80 – 2.68 (m, 2H), 1.30 – 1.24 (td, *J* = 7.1, 0.9 Hz, 3H).

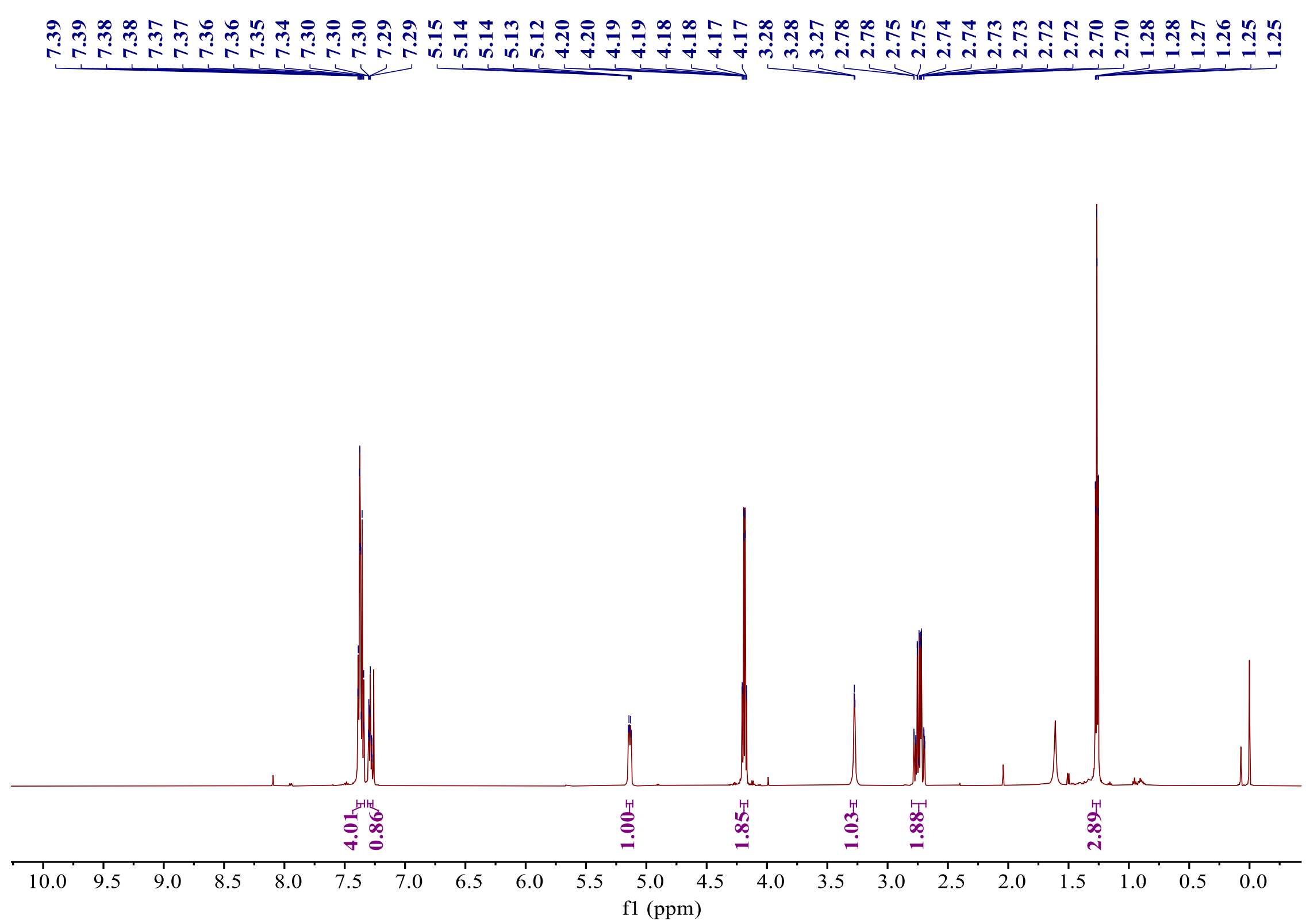

The ee-value of **1j** was determined by HPLC analysis with a 25 cm × 4.6 mm Daicel Chiralcel ID column (hexane/i-PrOH = 95/5, 1.0 mL/min). t_{minor} = 16.9 min, t_{major} = 13.1 min; ee = 93%.

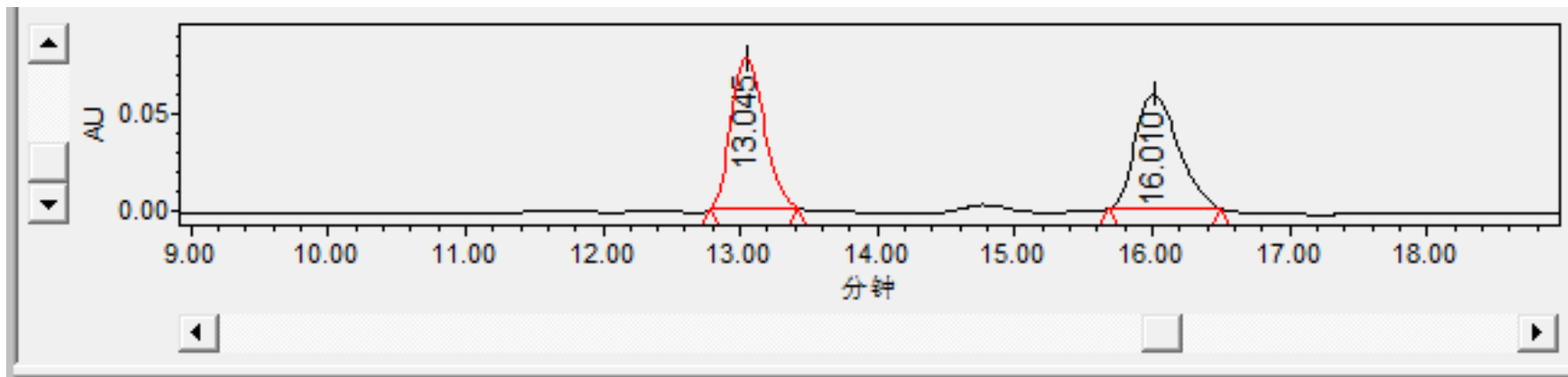

	名称	保留时间 (分钟)	面积 (微伏*秒)	% 面积	高度 (微伏)	积分类型	含量	单位	峰类型	峰代码
1		13.045	1295096	49.92	77778	bb			未知	
2		16.010	1299161	50.08	59305	bb			未知	

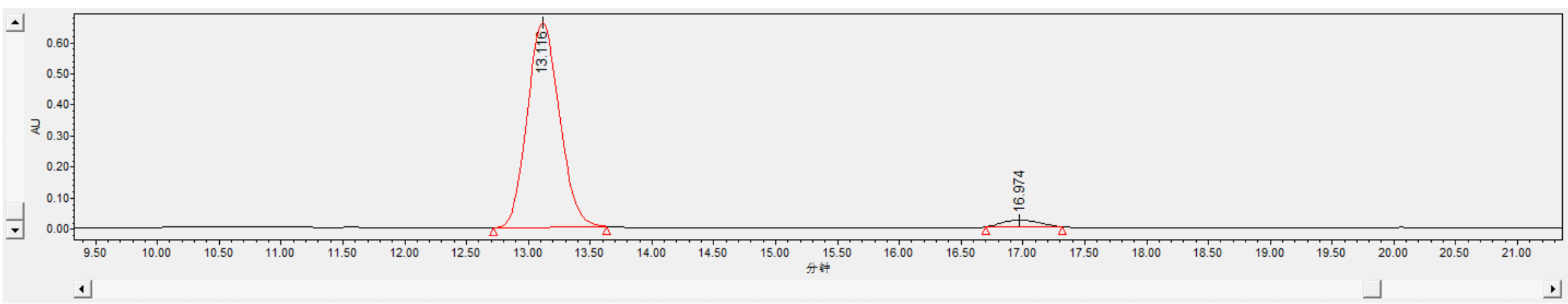

	名称	保留时间 (分钟)	面积 (微伏*秒)	% 面积	高度 (微伏)	积分类型	含量	单位	峰类型	峰代码
1		13.116	11892713	96.46	656596	bb			未知	
2		16.974	435993	3.54	21738	bb			未知	

Peaks number	Retention time (min)	Area	Area percentage (%)	Height of peaks
1	13.116	11892713	96.46	656596
2	16.974	435993	3.54	21738

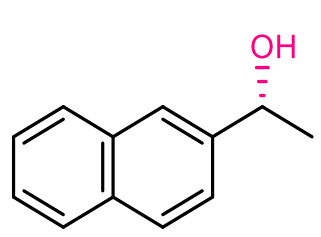

(*R*)-1-(naphthalen-2-yl)ethan-1-ol (1k). (Known compound, see: Chem. 2024, 10, 250–264):

The product **1a** was purified by flash chromatography on silica gel (300-400 mesh). ^{1}H NMR (600 MHz, CHLOROFORM-*D*) δ 7.86 – 7.82 (m, 3H), 7.81 – 7.80 (d, *J* = 1.8 Hz, 1H), 7.52 – 7.46 (m, 3H), 5.08 – 5.04 (q, *J* = 6.5 Hz, 1H), 2.06 – 2.00 (s, 1H), 1.60 – 1.57 (d, *J* = 6.5 Hz, 3H).

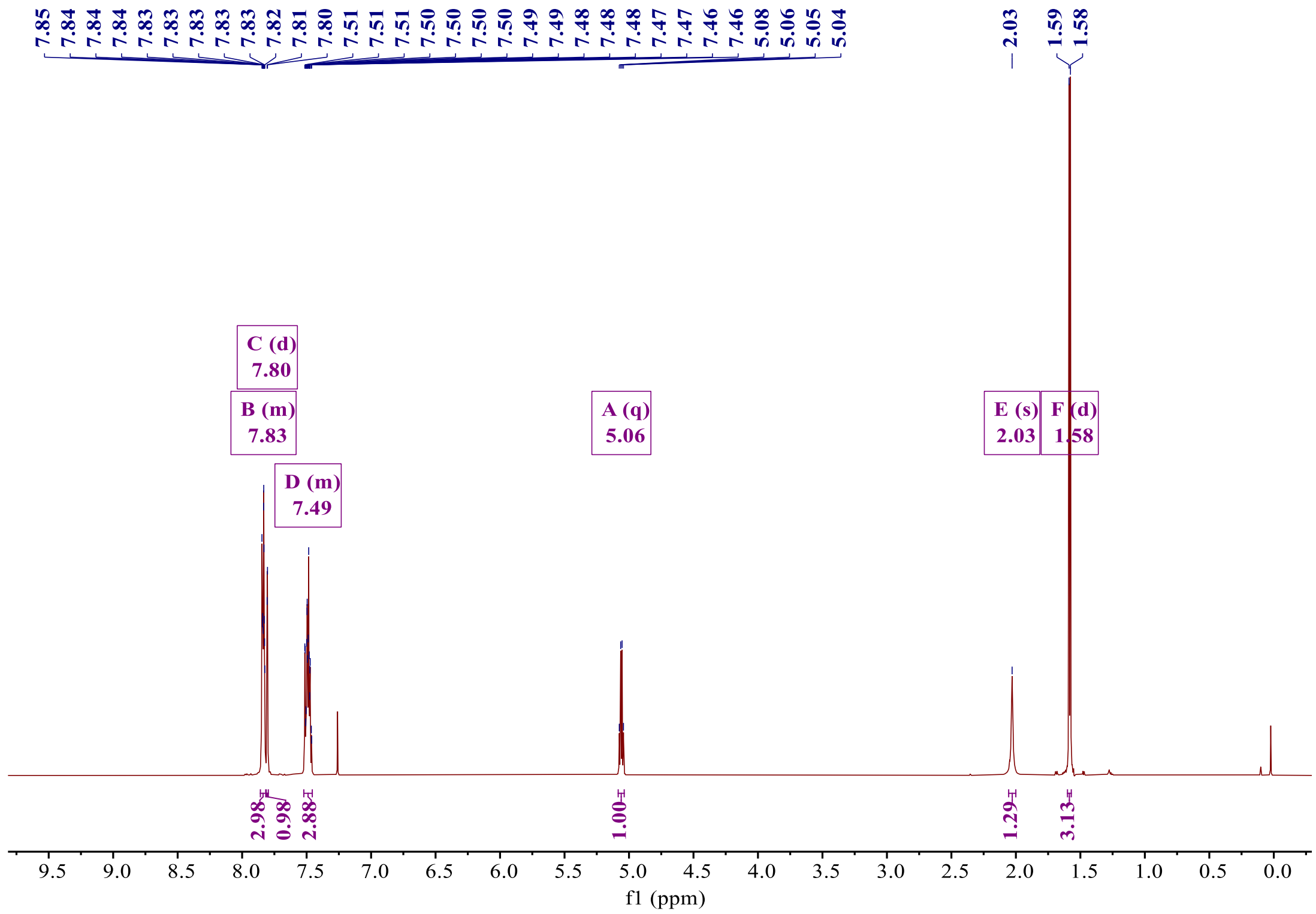

The ee-value of **1k** was determined by HPLC analysis with a 25 cm × 4.6 mm Daicel Chiralcel ID column (hexane/i-PrOH = 99/1, 1.0 mL/min). t_{minor} = 24.5 min, t_{major} = 22.5 min; ee = 93%.

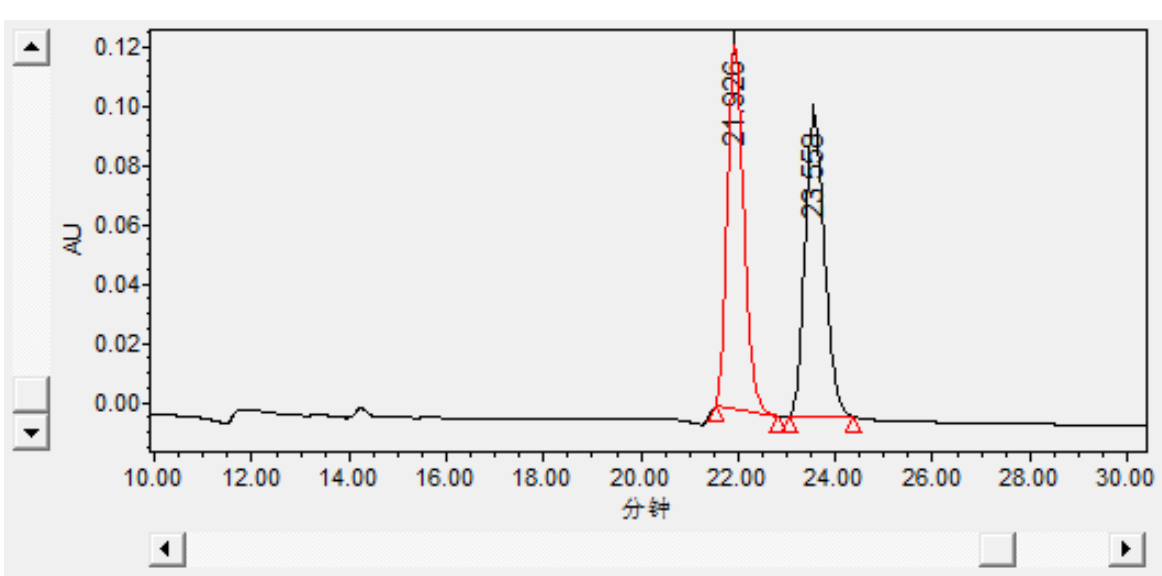

	名称	保留时间 (分钟)	面积 (微伏*秒)	% 面积	高度 (微伏)	积分类型	含量	单位	峰类型	峰代码
1		21.926	2908626	50.50	122773	bb			未知	
2		23.558	2850460	49.50	101072	bb			未知	

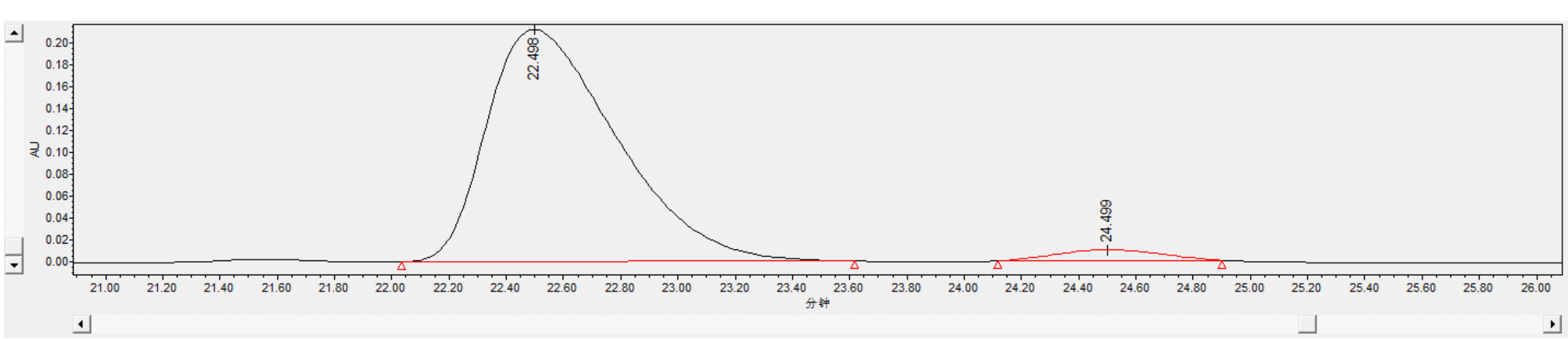

	名称	保留时间 (分钟)	面积 (微伏*秒)	% 面积	高度 (微伏)	积分类型	含量	单位	峰类型	峰代码
1		22.498	6702125	96.44	213145	bb			未知	
2		24.499	247192	3.56	9915	bb			未知	

Peaks number	Retention time (min)	Area	Area percentage (%)	Height of peaks
1	22.498	6702125	96.44	213145
2	24.499	247192	3.56	9915

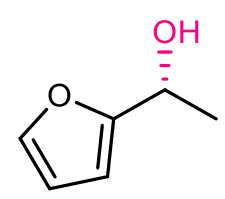

(*R*)-1-(furan-2-yl)ethan-1-ol (1l). (Known compound, see: Organic Letters (2000), 2(26), 4173-4176):

The product **1l** was purified by flash chromatography on silica gel (300-400 mesh). ^{1}H NMR (600 MHz, CHLOROFORM-*D*) δ 7.39 – 7.36 (m, 1H), 6.35 – 6.32 (dd, *J* = 3.2, 1.8 Hz, 1H), 6.25 – 6.22 (d, *J* = 3.3 Hz, 1H), 4.92 – 4.86 (q, *J* = 6.6 Hz, 1H), 2.00 – 1.89 (s, 1H), 1.56 – 1.54 (d, *J* = 6.6 Hz, 3H).

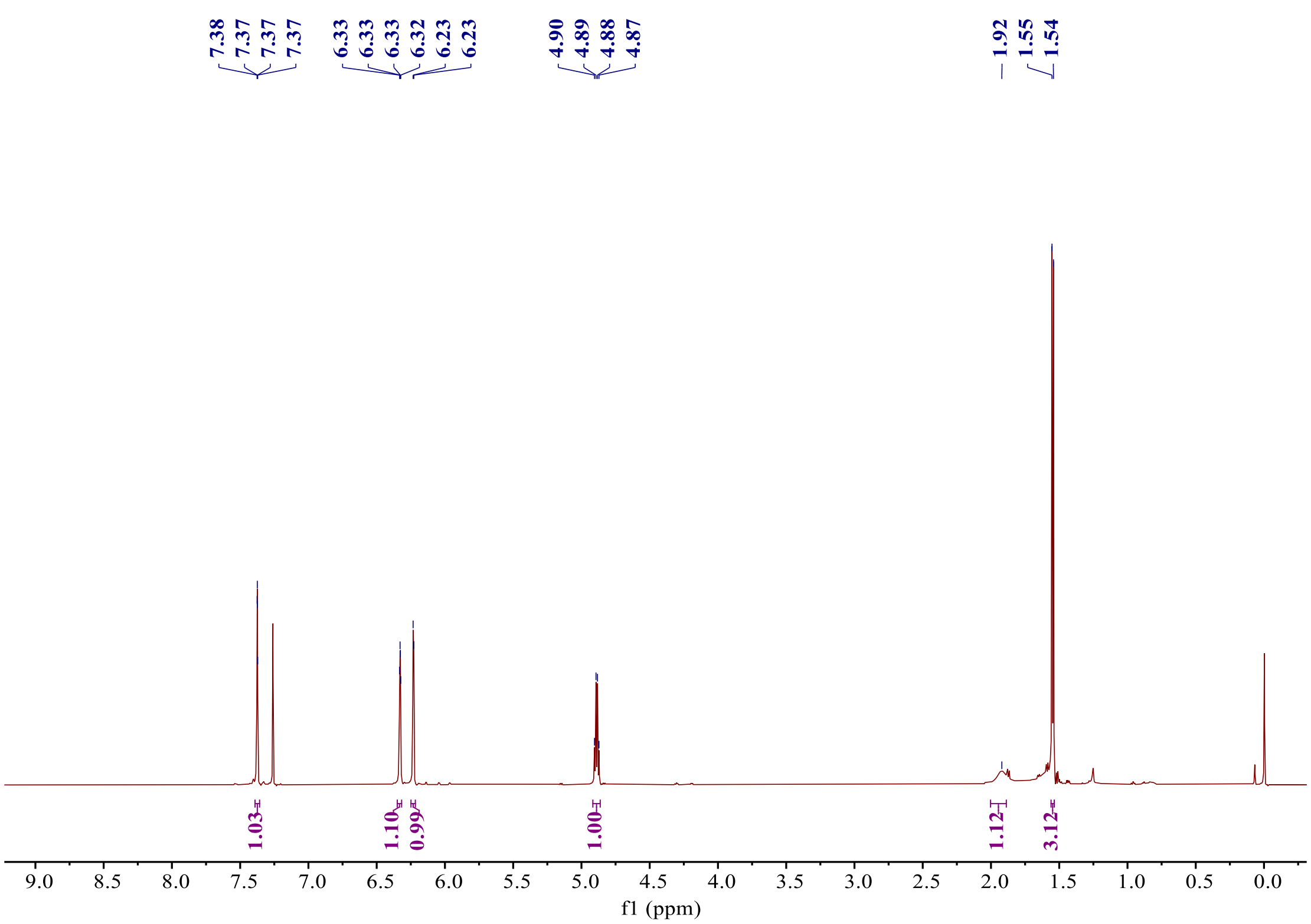

The ee-value of **1l** was determined by HPLC analysis with a 25 cm × 4.6 mm Daicel Chiralcel ID column (hexane/i-PrOH = 99/1, 1.0 mL/min). t_{minor} = 18.1 min, t_{major} = 19.6 min; ee = 97%.

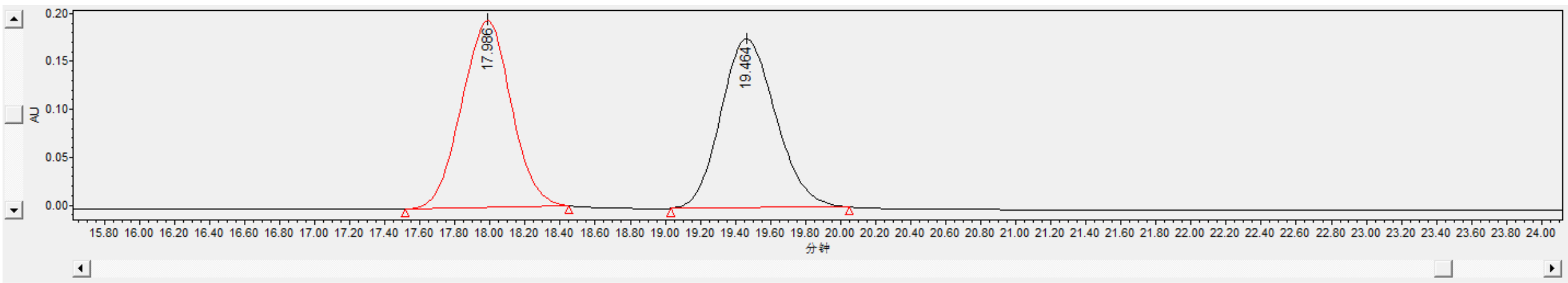

	名称	保留时间(分钟)	面积(微伏*秒)	% 面积	高度(微伏)	积分类型	含量	单位	峰类型	峰代码
1		17.986	3834491	49.97	195421	bb			未知	
2		19.464	3839085	50.03	176271	bb			未知	

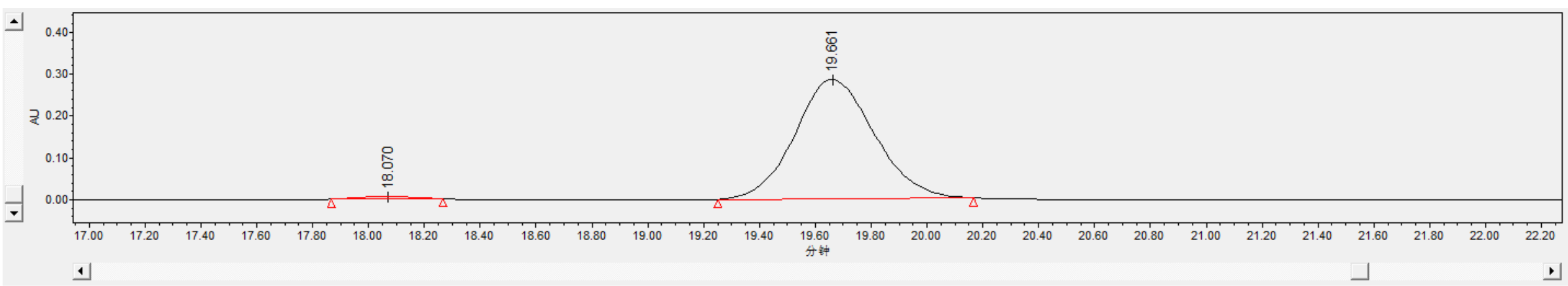

	名称	保留时间(分钟)	面积(微伏*秒)	% 面积	高度(微伏)	积分类型	含量	单位	峰类型	峰代码
1		18.070	81862	1.44	5846	bb			未知	
2		19.661	5601011	98.56	284478	bb			未知	

Peaks number	Retention time (min)	Area	Area percentage (%)	Height of peaks
1	18.070	81862	1.44	5846
2	19.661	5601011	98.56	28447

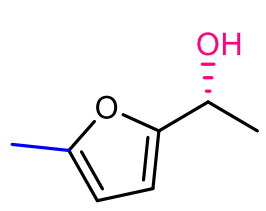

(*R*)-1-(5-methylfuran-2-yl)ethan-1-ol (1m). (Known compound, see: Chem. 2024, 10, 250–264):

The product **1a** was purified by flash chromatography on silica gel (300-400 mesh). ^{1}H NMR (600 MHz, CHLOROFORM-*D*) δ 6.11 – 6.09 (d, *J* = 3.1 Hz, 1H), 5.91 – 5.87 (dq, *J* = 3.3, 1.1 Hz, 1H), 4.86 – 4.80 (q, *J* = 6.9 Hz, 1H), 2.31 – 2.27 (d, *J* = 1.1 Hz, 3H), 1.91 – 1.87 (d, *J* = 3.7 Hz, 1H), 1.53 – 1.51 (d, *J* = 6.6 Hz, 3H).

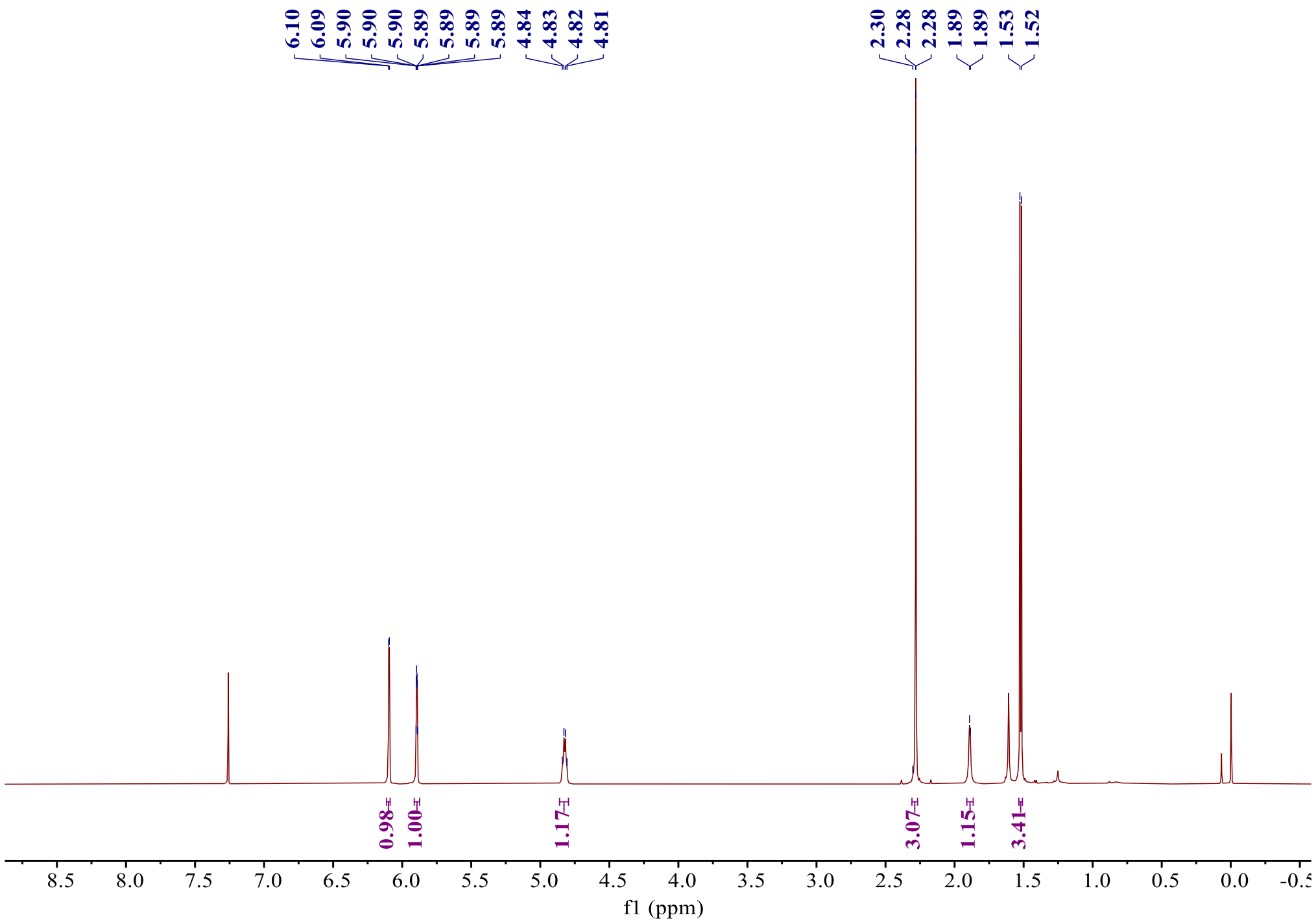

The ee-value of **1m** was determined by HPLC analysis with a 25 cm × 4.6 mm Daicel Chiralcel IC column (hexane/i-PrOH = 99/1, 1.0 mL/min). t_{minor} = 20.1 min, t_{major} = 20.6 min; ee = 95%.

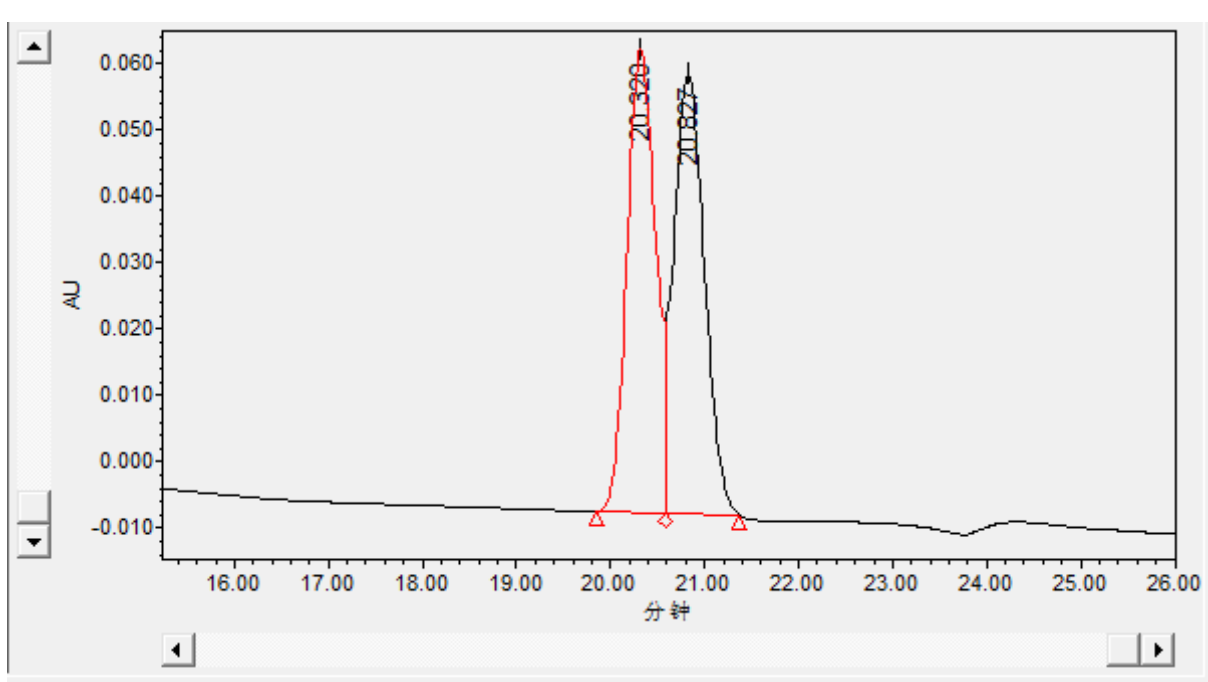

	名称	保留时间 (分钟)	面积 (微伏*秒)	% 面积	高度 (微伏)	积分类型	含量	单位	峰类型	峰代码
1		20.320	1463204	49.24	69903	bv			未知	
2		20.827	1508131	50.76	66499	vb			未知	

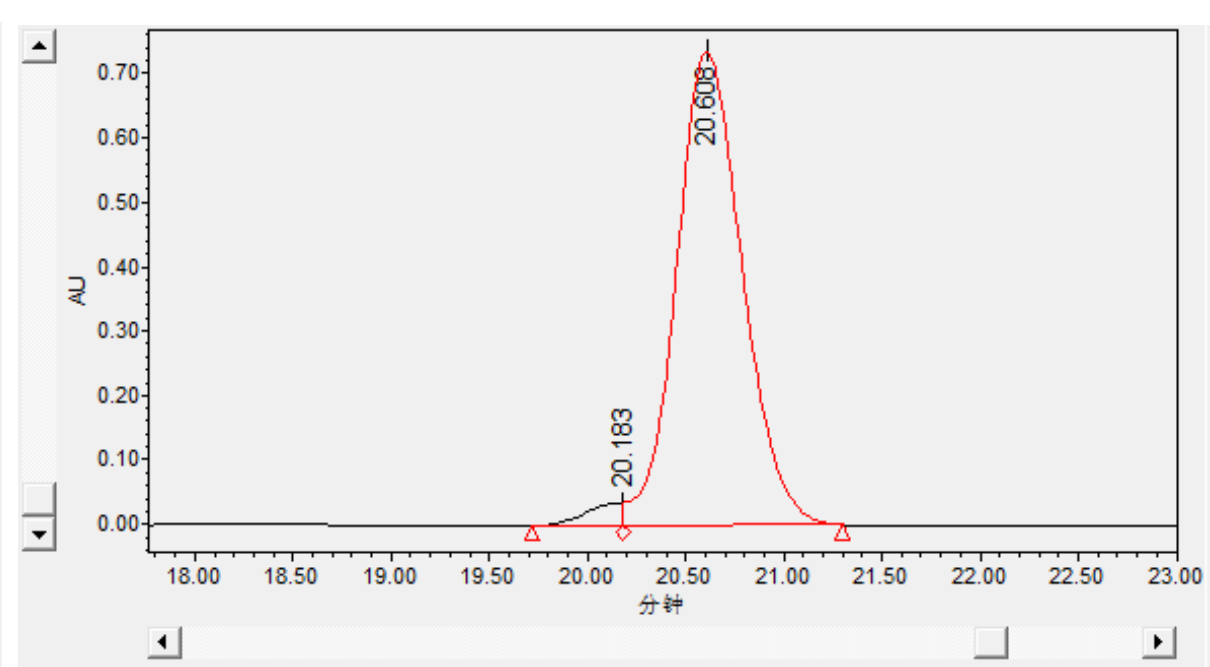

	名称	保留时间 (分钟)	面积 (微伏*秒)	% 面积	高度 (微伏)	积分类型	含量	单位	峰类型	峰代码
1		20.183	445603	2.52	35636	bv			未知	I06
2		20.608	17204415	97.48	736630	vb			未知	

Peaks number	Retention time (min)	Area	Area percentage (%)	Height of peaks
1	20.183	445603	2.52	35636
2	20.605	17204415	97.48	736630

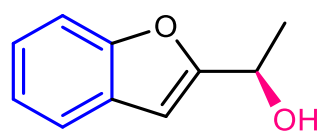

(*R*)-1-(benzofuran-2-yl)ethan-1-ol (1n). (Known compound, see: Journal of Organic Chemistry (1989), 54(9), 2085-91):

The product **1n** was purified by flash chromatography on silica gel (300-400 mesh). ^{1}H NMR (600 MHz, CHLOROFORM-*D*) δ 7.56 – 7.51 (dt, *J* = 7.6, 1.8 Hz, 1H), 7.47 – 7.43 (m, 1H), 7.28 – 7.23 (m, 1H), 7.22 – 7.19 (m, 1H), 6.66 – 6.46 (m, 1H), 5.02 – 4.97 (m, 1H), 2.40 – 2.23 (s, 1H), 1.64 – 1.59 (dt, *J* = 6.6, 2.1 Hz, 3H).

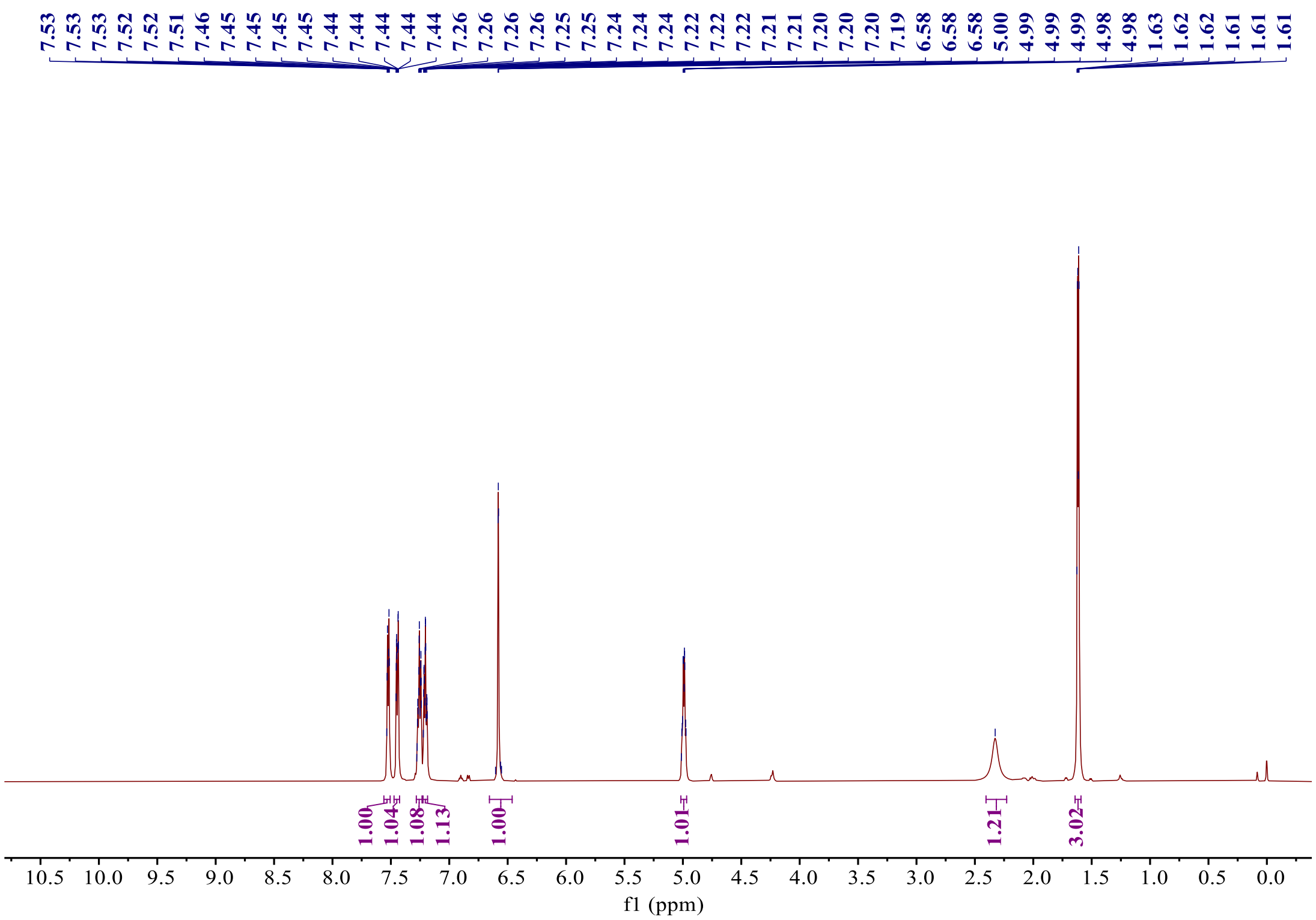

The ee-value of **1n** was determined by HPLC analysis with a 25 cm × 4.6 mm Daicel Chiralcel ID column (hexane/i-PrOH = 99/1, 1.0 mL/min). t_{minor} = 23.8 min, t_{major} = 22.7 min; ee = 90%.

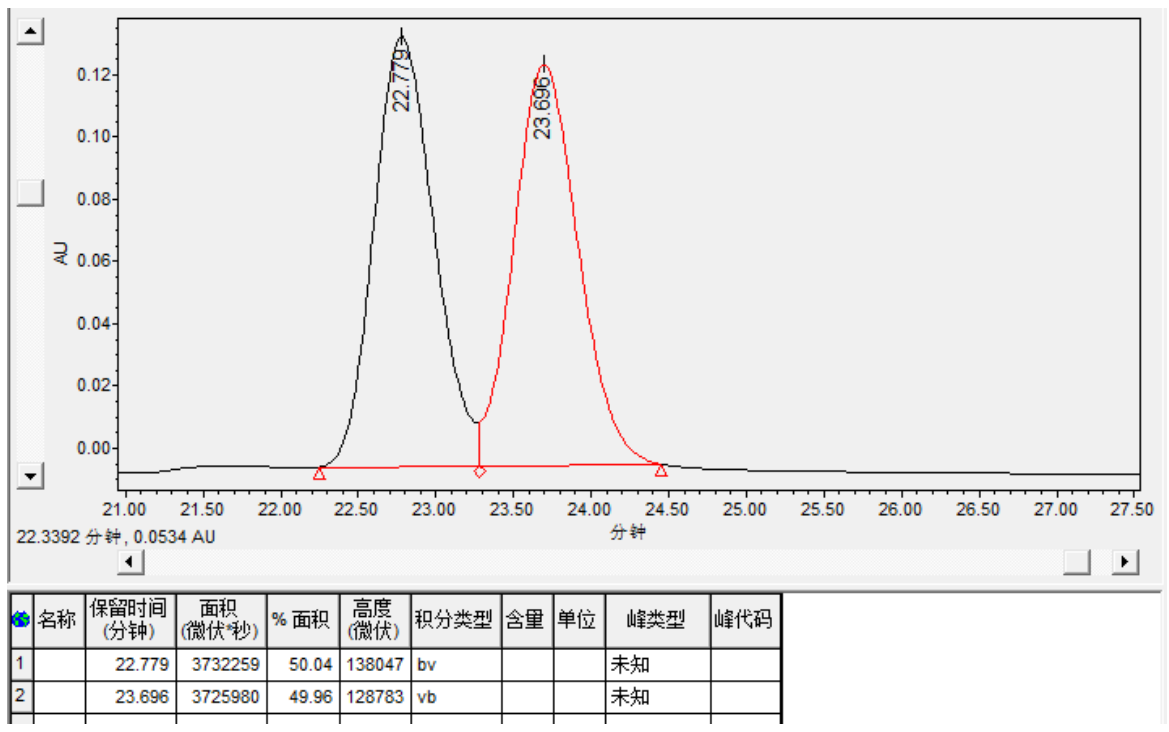

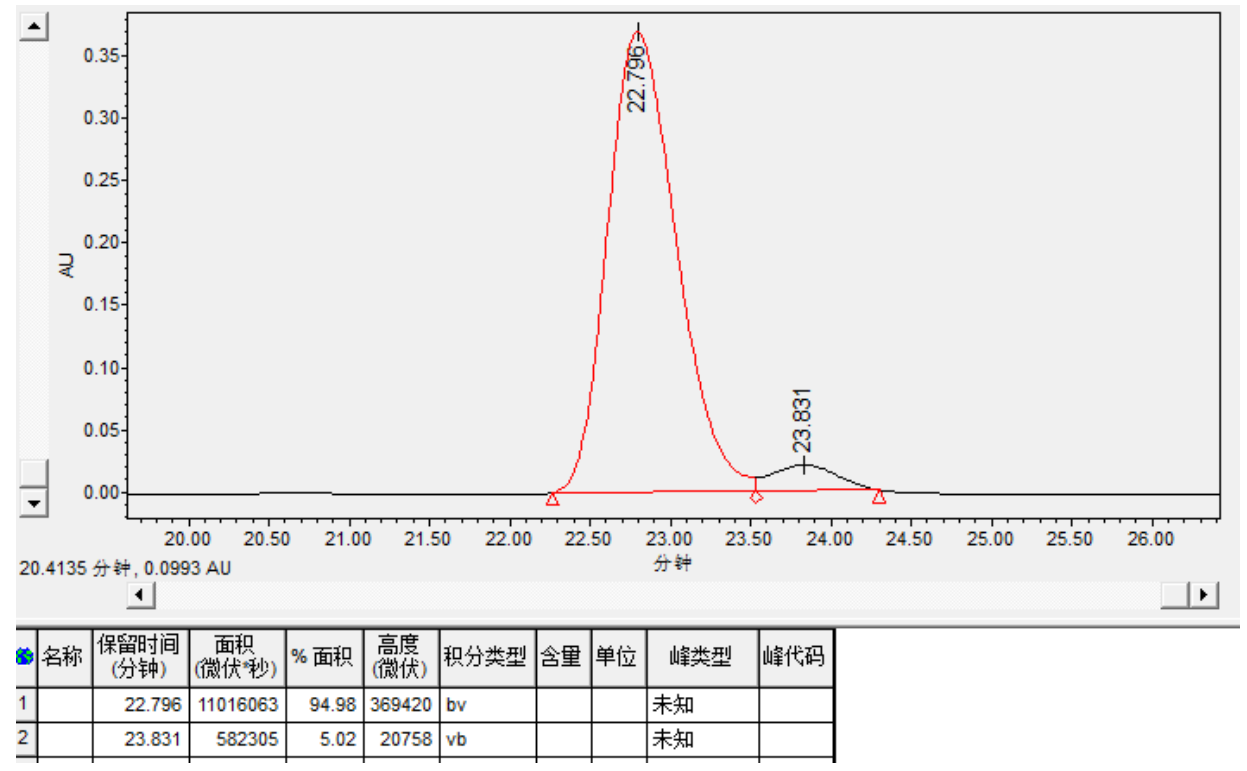

Peaks number	Retention time (min)	Area	Area percentage (%)	Height of peaks
1	22.796	11016063	94.96	369420
2	23.831	582305	5.02	20758

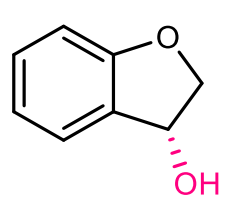

(*R*)-2,3-dihydrobenzofuran-3-ol (1o). (Known compound, see: Heterocycles (2010), 80(1), 141-147):

The product **1o** was purified by flash chromatography on silica gel (300-400 mesh). ^{1}H NMR (600 MHz, CHLOROFORM-*D*) δ 7.41 – 7.37 (m, 1H), 7.28 – 7.23 (m, 1H), 6.96 – 6.91 (tt, *J* = 7.4, 0.9 Hz, 1H), 6.89 – 6.85 (d, *J* = 8.1 Hz, 1H), 5.29 – 5.27 (dd, *J* = 6.6, 2.5 Hz, 1H), 4.50 – 4.46 (ddd, *J* = 10.6, 6.5, 0.8 Hz, 1H), 4.40 – 4.36 (ddd, *J* = 10.7, 2.5, 0.7 Hz, 1H), 2.34 – 2.20 (s, 1H).

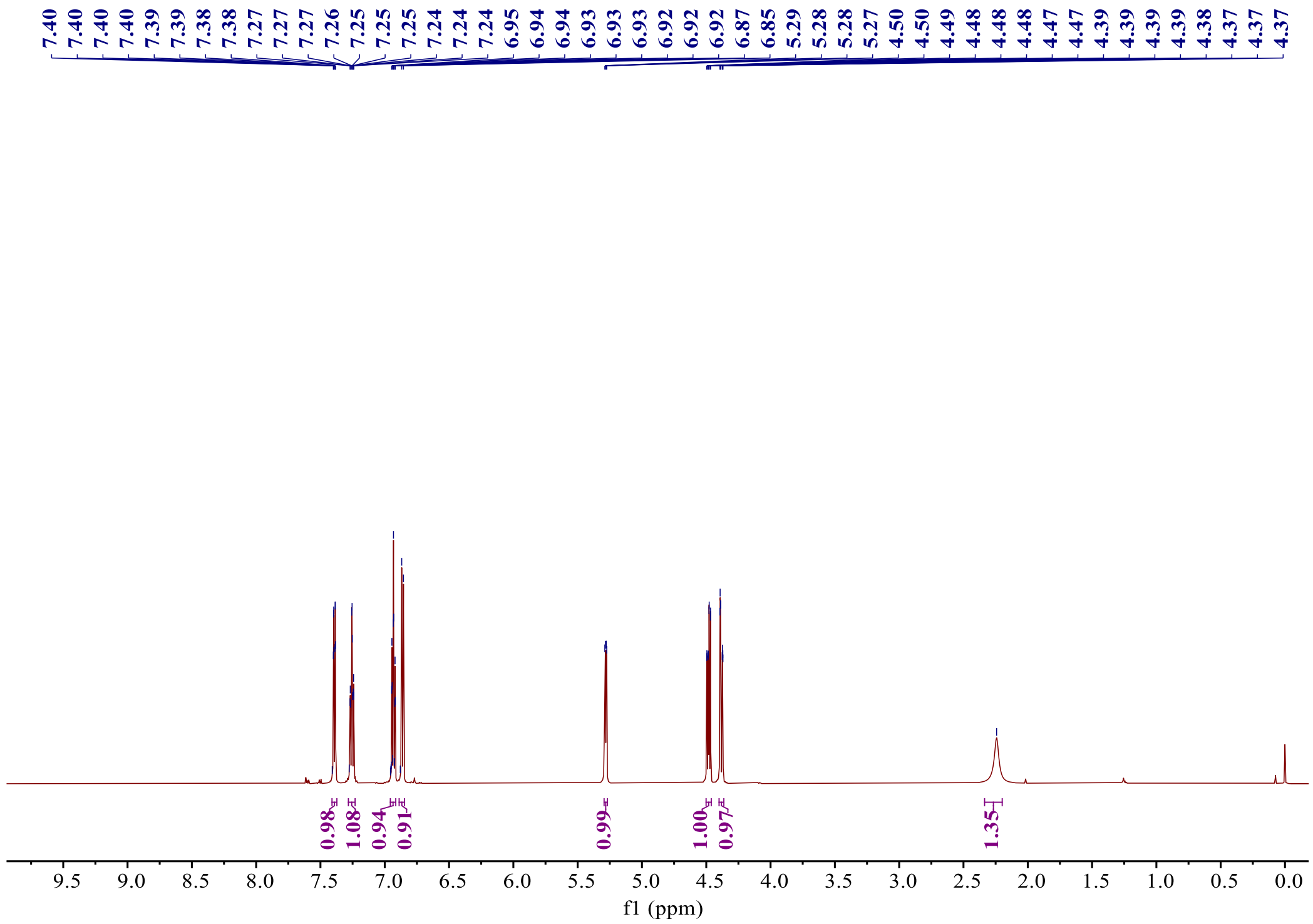

The ee-value of **1o** was determined by HPLC analysis with a 25 cm × 4.6 mm Daicel Chiralcel ID column (hexane/i-PrOH = 97/3, 1.0 mL/min). t_{minor} = 14.8 min, t_{major} = 14.1 min; ee = 90%.

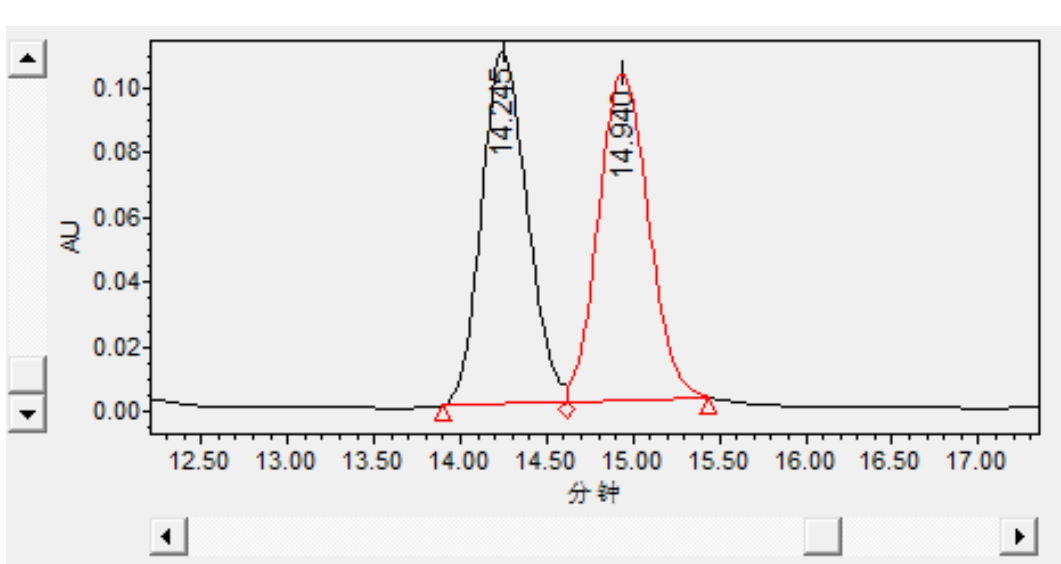

	名称	保留时间 (分钟)	面积 (微伏*秒)	% 面积	高度 (微伏)	积分类型	含量	单位	峰类型	峰代码
1		14.245	1995101	49.61	108726	bv			未知	
2		14.940	2026199	50.39	100829	vb			未知	

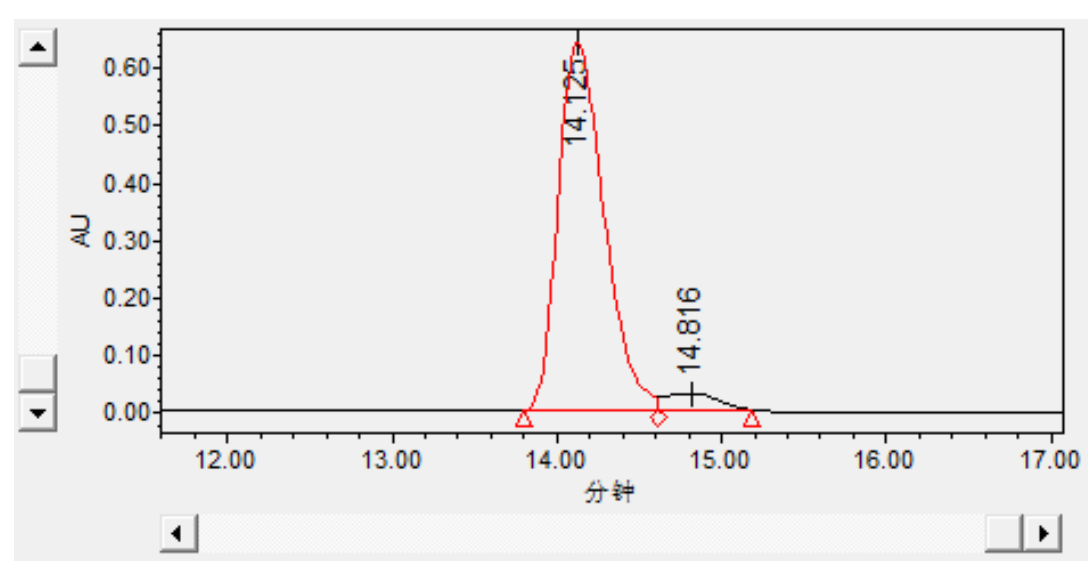

	名称	保留时间 (分钟)	面积 (微伏*秒)	% 面积	高度 (微伏)	积分类型	含量	单位	峰类型	峰代码
1		14.125	12320351	95.05	638734	bv			未知	
2		14.816	641818	4.95	28882	vb			未知	

Peaks number	Retention time (min)	Area	Area percentage (%)	Height of peaks
1	14.125	12320351	95.05	638734
2	14.816	641818	4.95	28882

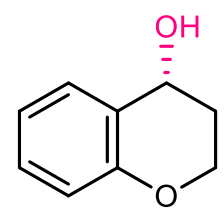

(*R*)-chroman-4-ol (1p). (Known compound, see: Journal of Organometallic Chemistry (1988), 346(3), 413-24):

The product **1p** was purified by flash chromatography on silica gel (300-400 mesh). ^{1}H NMR (600 MHz, CHLOROFORM-*D*) δ 7.33 – 7.28 (dt, *J* = 7.7, 2.0 Hz, 1H), 7.24 – 7.17 (m, 1H), 6.96 – 6.90 (tdd, *J* = 7.4, 2.4, 1.2 Hz, 1H), 6.87 – 6.82 (d, *J* = 8.2 Hz, 1H), 4.80 – 4.75 (q, *J* = 3.5 Hz, 1H), 4.31 – 4.19 (hd, *J* = 5.6, 2.6 Hz, 2H), 2.14 – 2.05 (m, 2H), 2.04 – 1.96 (m, 1H).

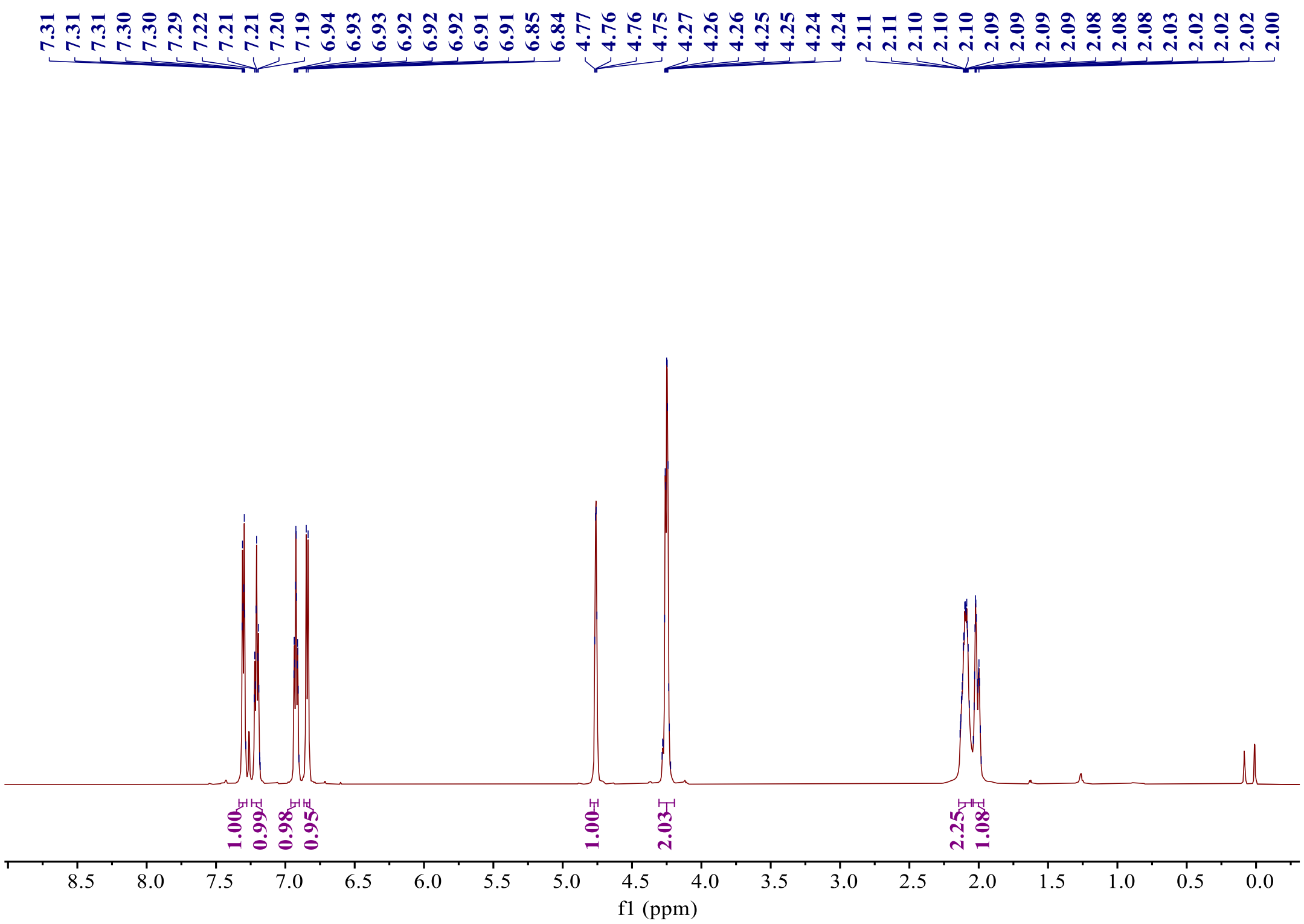

The ee-value of **1p** was determined by HPLC analysis with a 25 cm × 4.6 mm Daicel Chiralcel ID column (hexane/i-PrOH = 97/3, 1.0 mL/min). t_{minor} = 16.7 min, t_{major} = 13.9 min; ee = 97%.

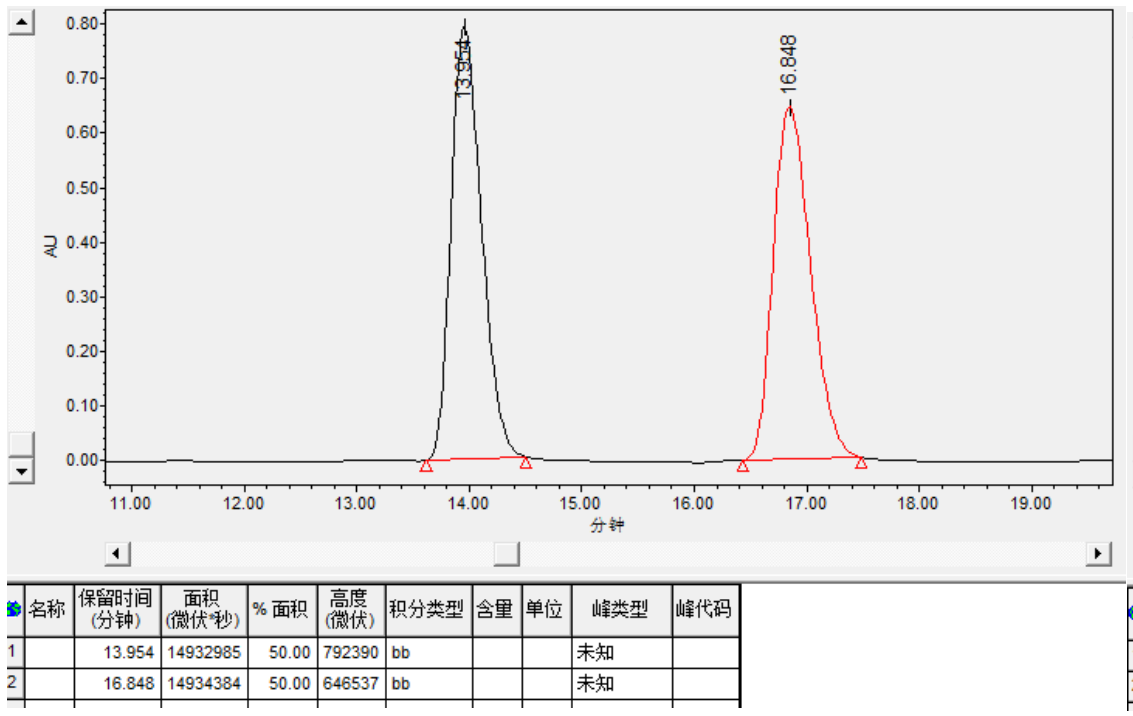

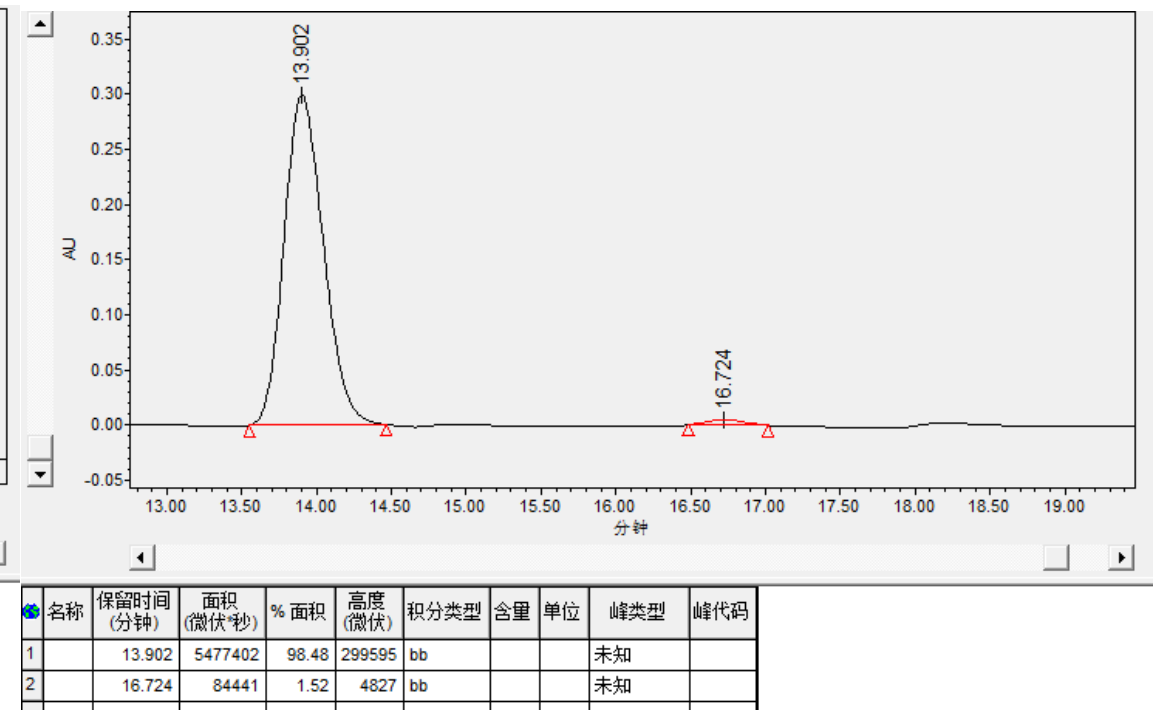

Peaks number	Retention time (min)	Area	Area percentage (%)	Height of peaks
1	13.902	5477402	98.48	299595
2	16.724	84441	1.52	4827

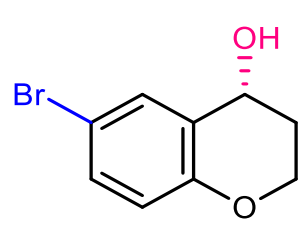

(*R*)-6-bromochroman-4-ol (1q). (Known compound, see: Applied Organometallic Chemistry (2024), 38(3), e7379):

The product **1q** was purified by flash chromatography on silica gel (300-400 mesh). ^{1}H NMR (600 MHz, CHLOROFORM-*D*) δ 7.44 – 7.41 (t, *J* = 2.5 Hz, 1H), 7.29 – 7.24 (dq, *J* = 7.5, 2.5 Hz, 1H), 6.76 – 6.68 (dd, *J* = 8.8, 2.2 Hz, 1H), 4.75 – 4.68 (m, 1H), 4.28 – 4.18 (ddd, *J* = 7.9, 3.6, 1.7 Hz, 2H), 2.32 – 2.19 (s, 1H), 2.12 – 2.04 (m, 1H), 2.01 – 1.94 (m, 1H).

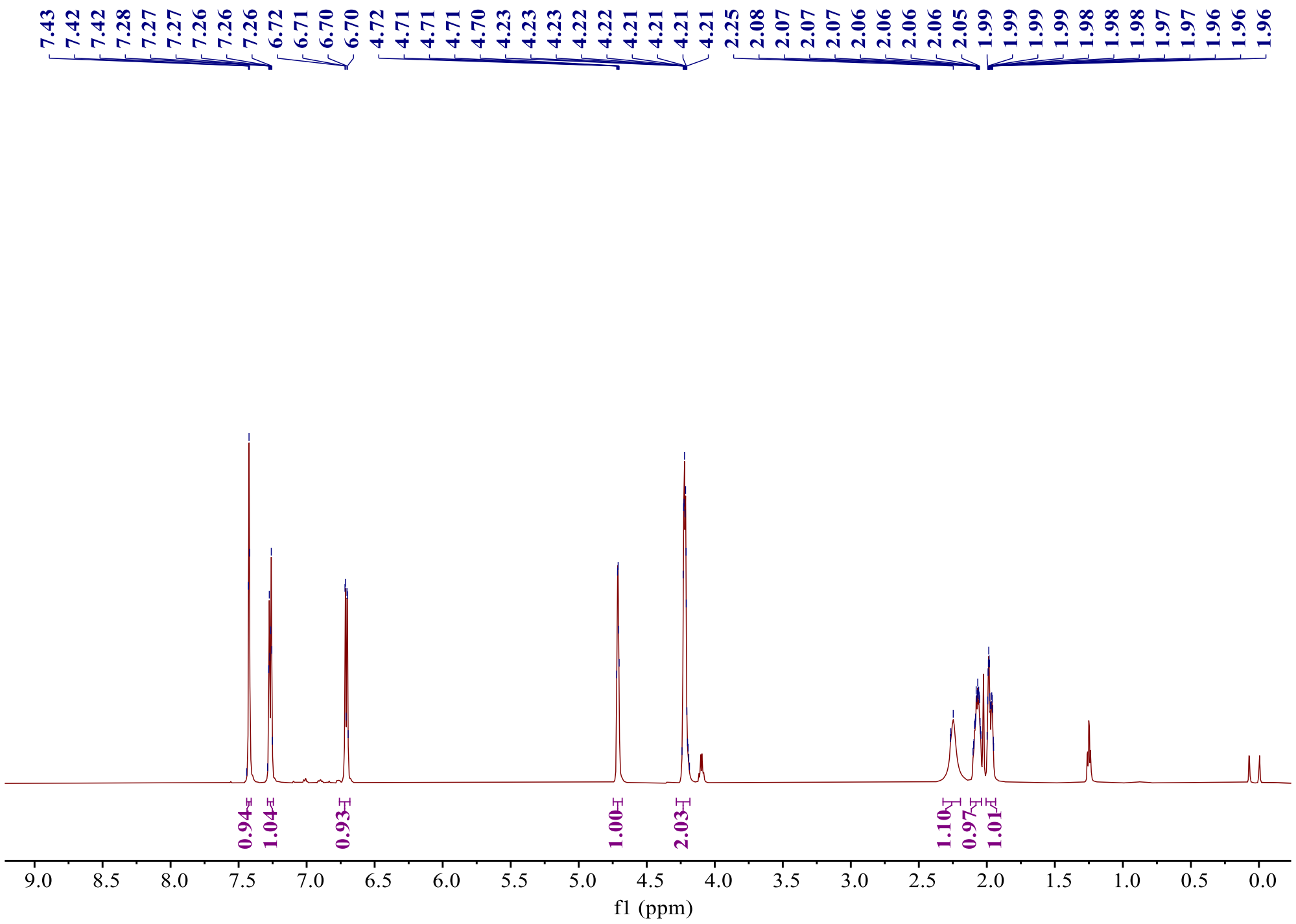

The ee-value of **1q** was determined by HPLC analysis with a 25 cm × 4.6 mm Daicel Chiralcel IC column (hexane/i-PrOH = 99/1, 1.0 mL/min). t_{minor} = 25.1 min, t_{major} = 29.8 min; ee = 90%.

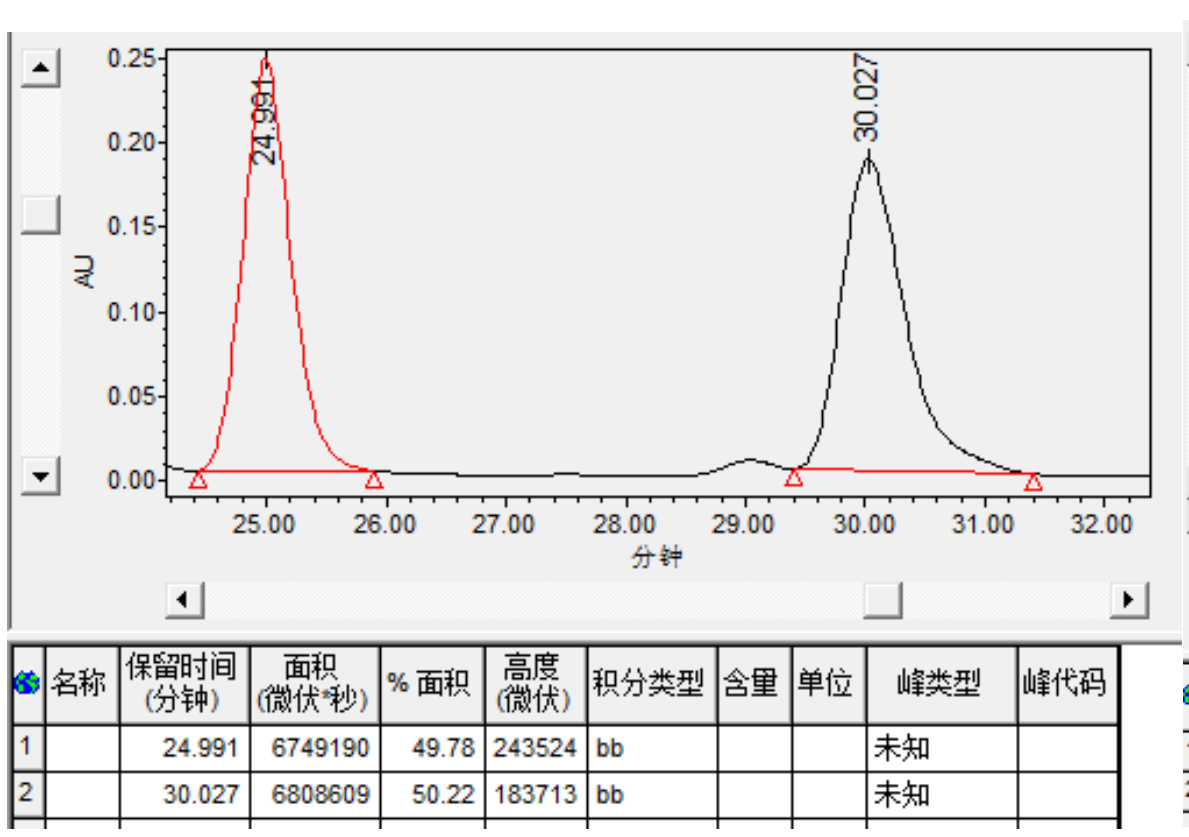

	名称	保留时间 (分钟)	面积 (微伏*秒)	% 面积	高度 (微伏)	积分类型	含量	单位	峰类型	峰代码
1		24.991	6749190	49.78	243524	bb			未知	
2		30.027	6808609	50.22	183713	bb			未知	

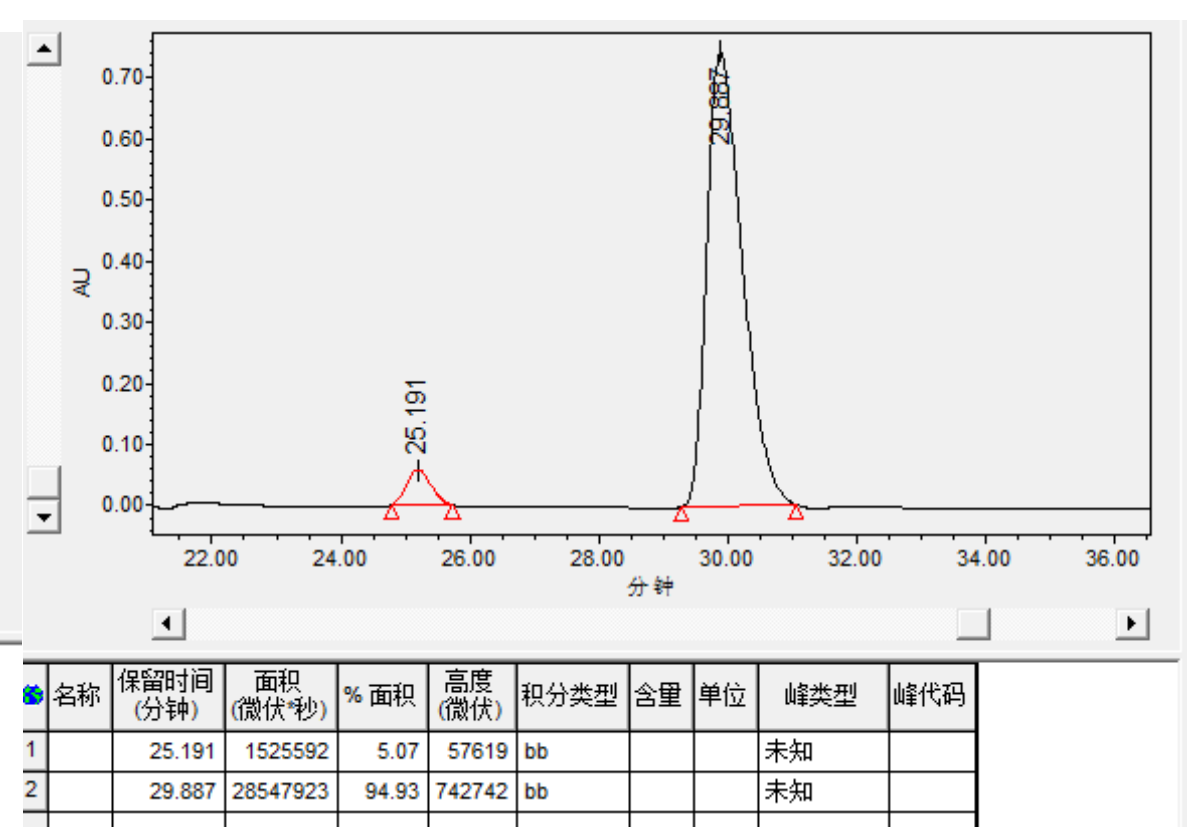

	名称	保留时间 (分钟)	面积 (微伏*秒)	% 面积	高度 (微伏)	积分类型	含量	单位	峰类型	峰代码
1		25.191	1525592	5.07	57619	bb			未知	
2		29.887	28547923	94.93	742742	bb			未知	

Peaks number	Retention time (min)	Area	Area percentage (%)	Height of peaks
1	25.191	1525592	5.07	57619
2	29.887	28547923	94.93	7427.42

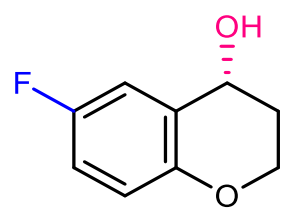

(*R*)-6-fluorochroman-4-ol (1r). (Known compound, see: Tetrahedron Letters (2012), 53(30), 3828-3830):

The product **1r** was purified by flash chromatography on silica gel (300-400 mesh). ^{1}H NMR (600 MHz, CHLOROFORM-*D*) δ 7.07 – 6.98 (dq, *J* = 9.2, 3.2 Hz, 1H), 6.95 – 6.84 (m, 1H), 6.81 – 6.74 (m, 1H), 4.78 – 4.70 (dt, *J* = 6.4, 3.1 Hz, 1H), 4.27 – 4.17 (ddd, *J* = 7.0, 4.4, 2.4 Hz, 2H), 2.20 – 2.14 (s, 1H), 2.14 – 2.06 (dddd, *J* = 14.4, 7.4, 3.6, 1.8 Hz, 1H), 2.02 – 1.95 (m, 1H).

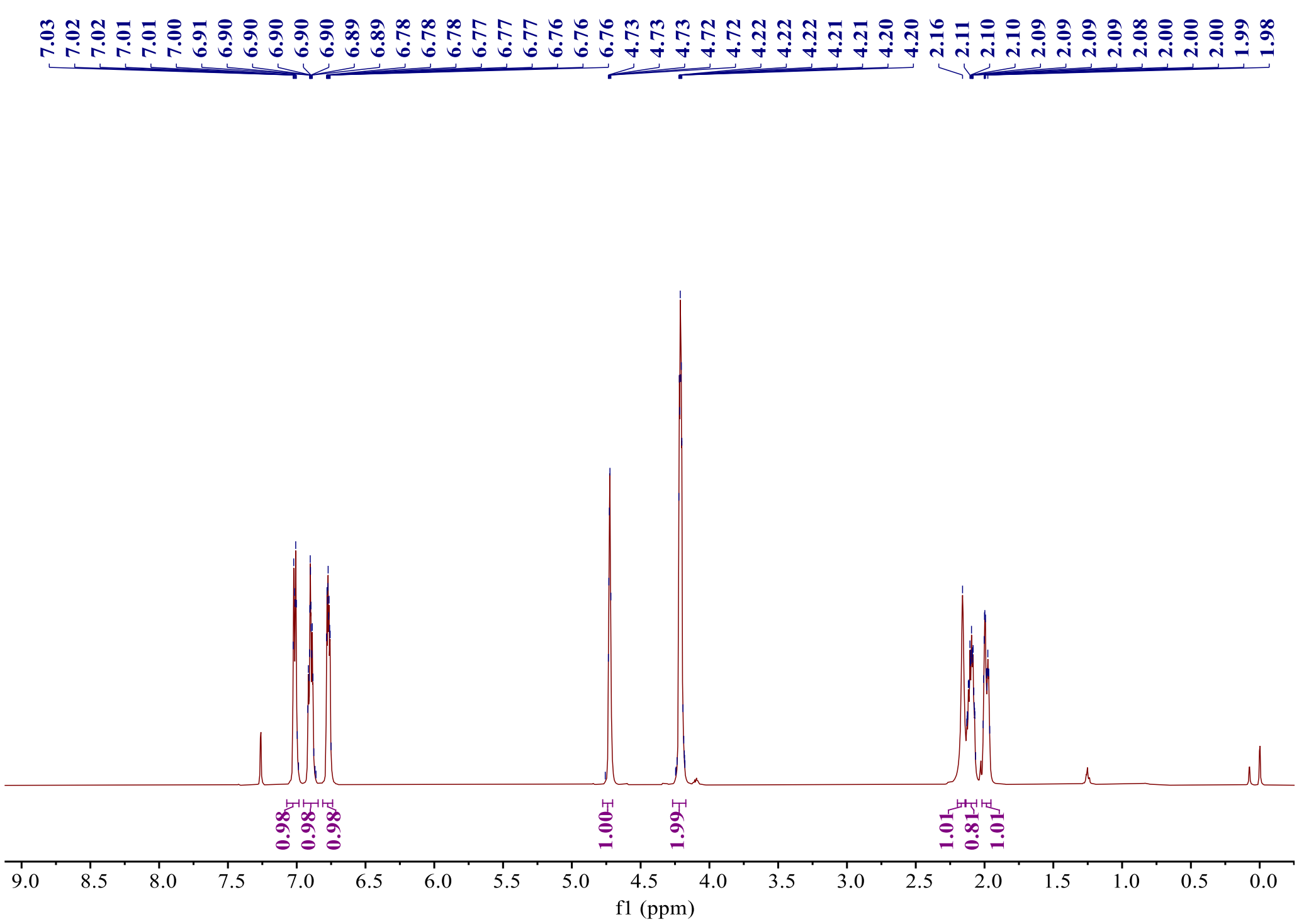

The ee-value of **1r** was determined by HPLC analysis with a 25 cm × 4.6 mm Daicel Chiralcel Ic column (hexane/i-PrOH = 99/1, 1.0 mL/min). t_{minor} = 28.2 min, t_{major} = 30.0 min; ee = 89%.

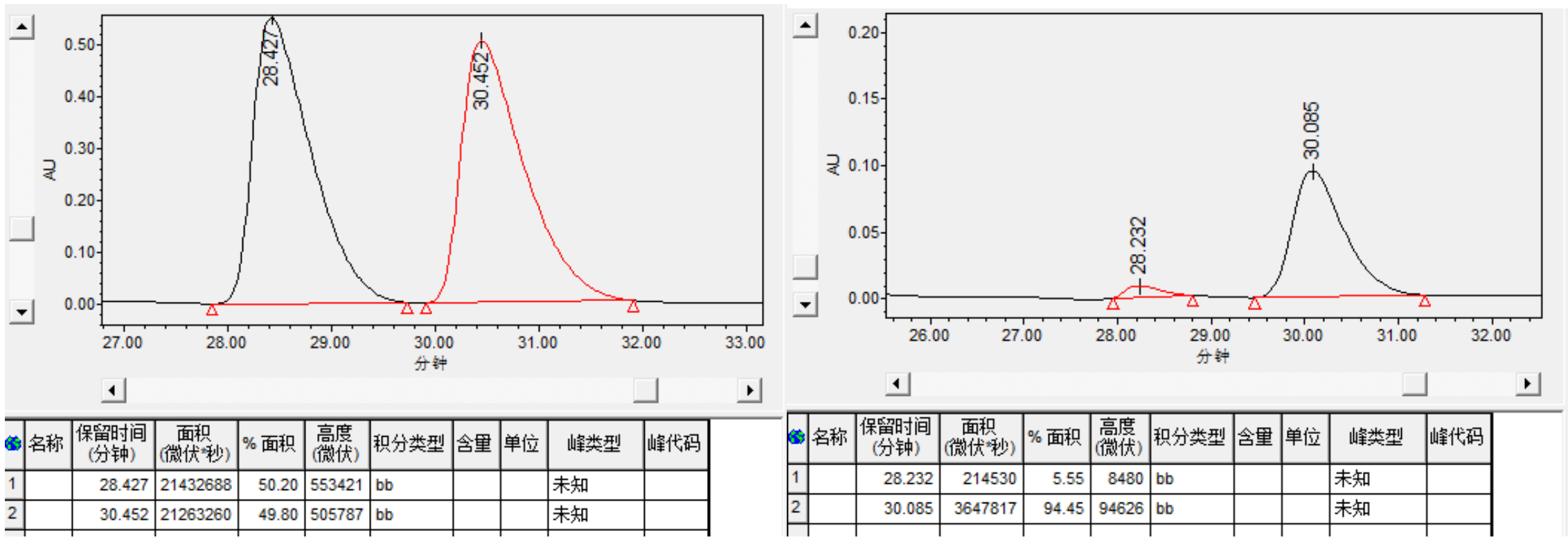

Peaks number	Retention time (min)	Area	Area percentage (%)	Height of peaks
1	28.232	214530	5.55	8480
2	30.085	3647817	94.45	94626

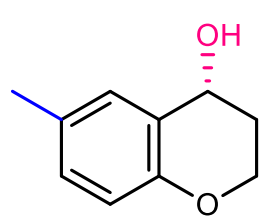

(*R*)-6-methylchroman-4-ol (1s). (Known compound, see: Journal of Medicinal Chemistry (2006), 49(5), 1818-1827):

The product **1s** was purified by flash chromatography on silica gel (300-400 mesh). ^{1}H NMR (600 MHz, CHLOROFORM-*D*) δ 7.15 – 7.09 (d, *J* = 2.3 Hz, 1H), 7.04 – 6.95 (dt, *J* = 8.4, 2.3 Hz, 1H), 6.79 – 6.72 (m, 1H), 4.74 – 4.70 (td, *J* = 4.2, 1.8 Hz, 1H), 4.24 – 4.18 (m, 2H), 2.29 – 2.26 (d, *J* = 2.0 Hz, 3H), 2.13 – 2.02 (m, 2H), 2.02 – 1.96 (m, 1H).

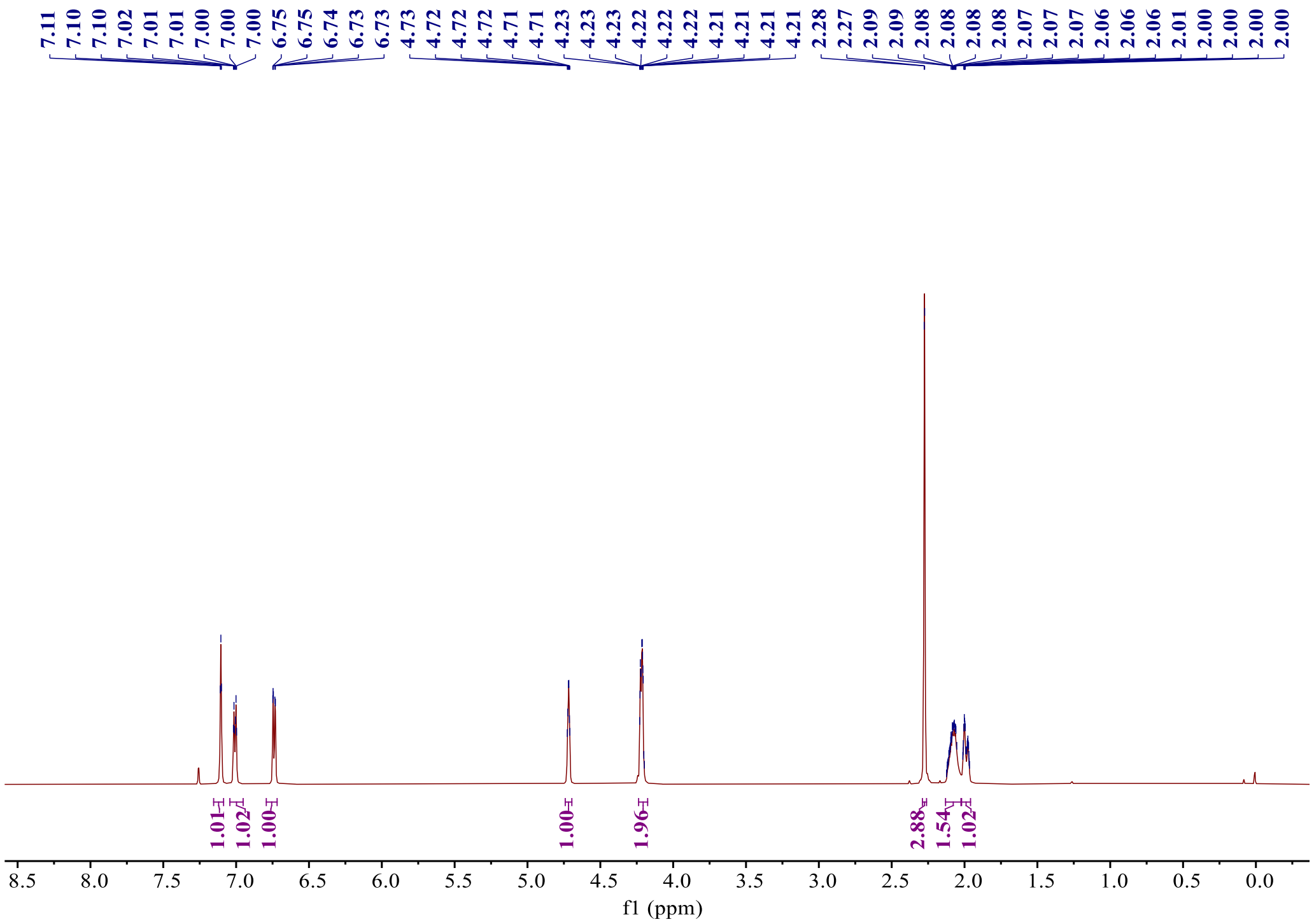

The ee-value of **1s** was determined by HPLC analysis with a 25 cm × 4.6 mm Daicel Chiralcel Ic column (hexane/i-PrOH = 98/2, 1.0 mL/min). t_{minor} = 13.9 min, t_{major} = 16.2 min; ee = 92%.

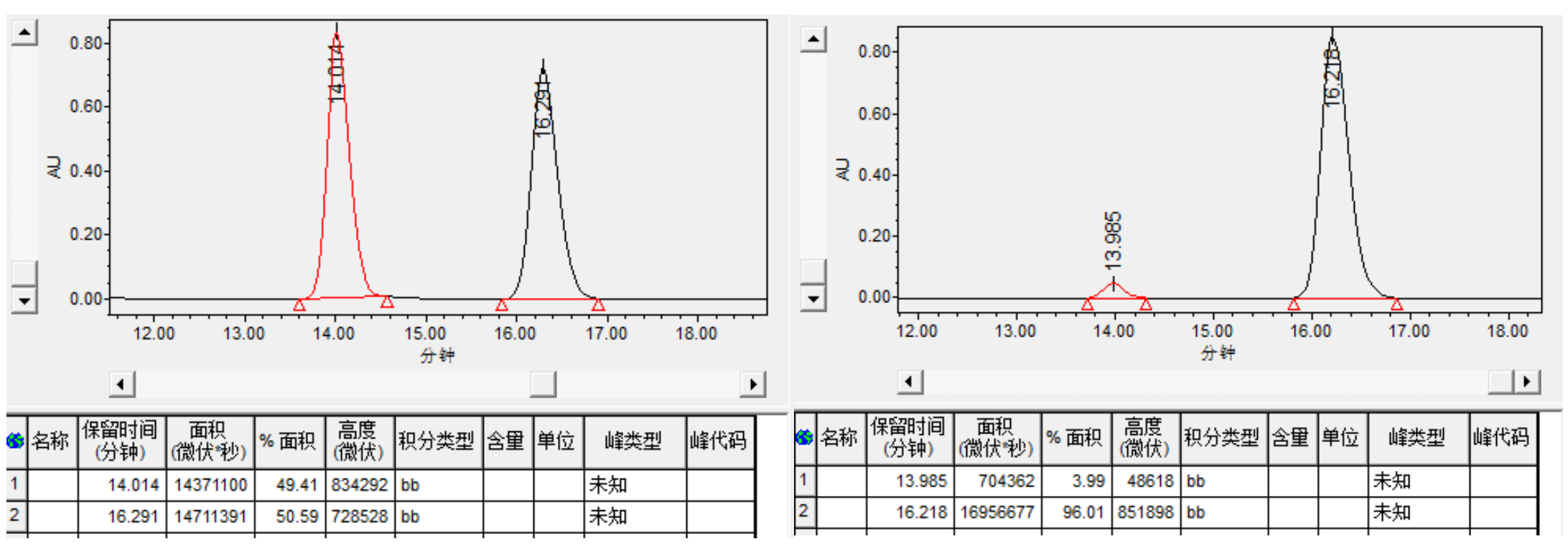

Peaks number	Retention time (min)	Area	Area percentage (%)	Height of peaks
1	13.985	704362	3.99	48618
2	16.218	16956677	96.01	851898

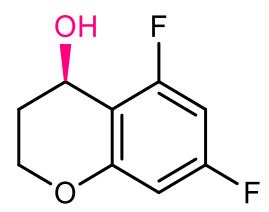

(*R*)-5,7-difluorochroman-4-ol (1t): (Known compound, see: Organic Preparations and Procedures International (2024), 56(3), 264-270).

The product **1t** was purified by flash chromatography on silica gel (300-400 mesh). ^{1}H NMR (600 MHz, CHLOROFORM-*D*) δ 6.45 – 6.37 (ddq, *J* = 10.4, 4.1, 2.4 Hz, 2H), 5.02 – 4.97 (t, *J* = 3.3 Hz, 1H), 4.33 – 4.20 (m, 2H), 2.27 – 2.15 (s, 1H), 2.07 – 1.95 (m, 2H). ^{19}F NMR (565 MHz, CHLOROFORM-*D*) δ -117.15, -109.09.

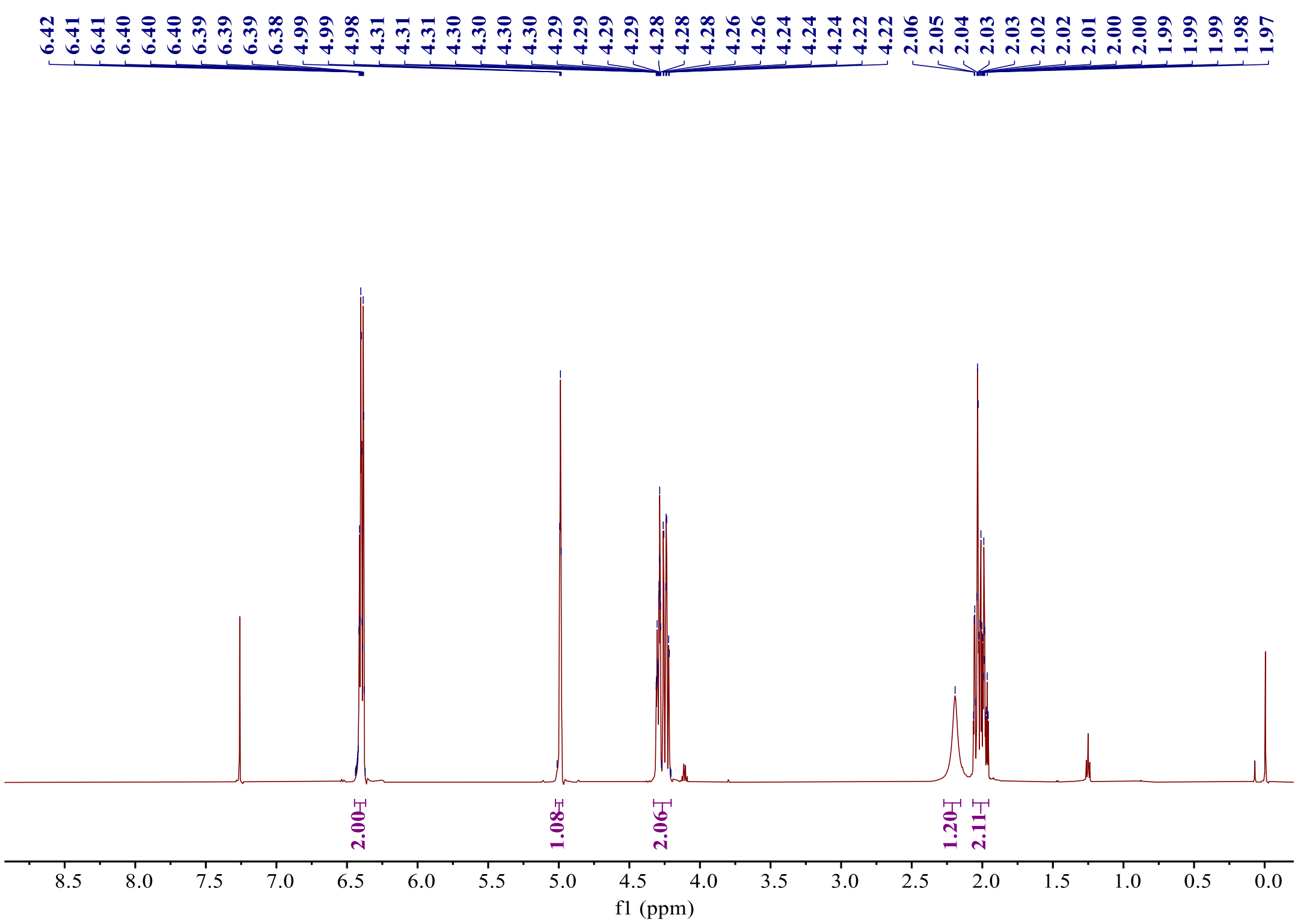

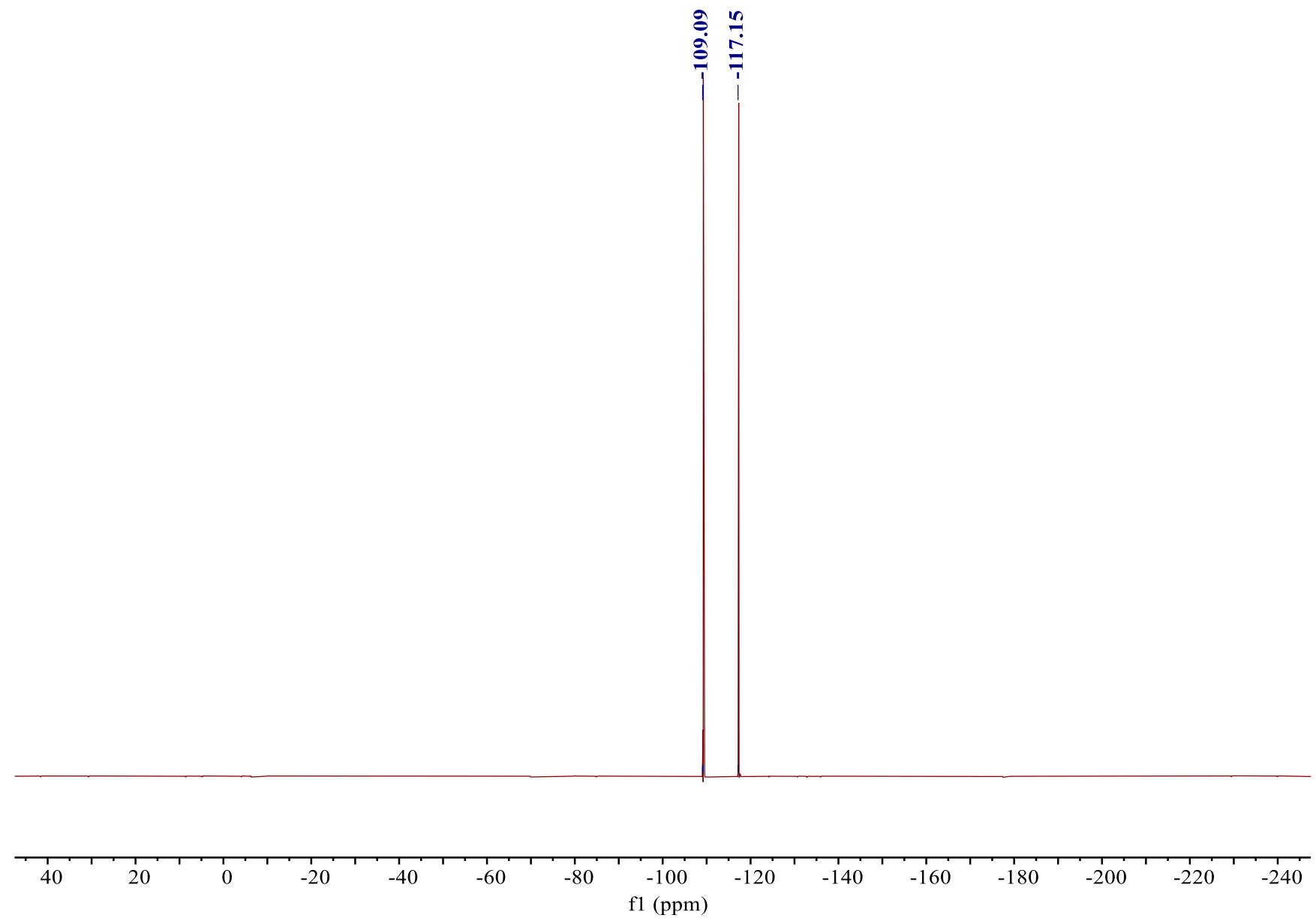

The ee-value of **1t** was determined by HPLC analysis with a 25 cm × 4.6 mm Daicel Chiralcel IC column (hexane/i-PrOH = 99/1, 1.0 mL/min). t_{minor} = 13.2 min, t_{major} = 15.3 min; ee = 97%.

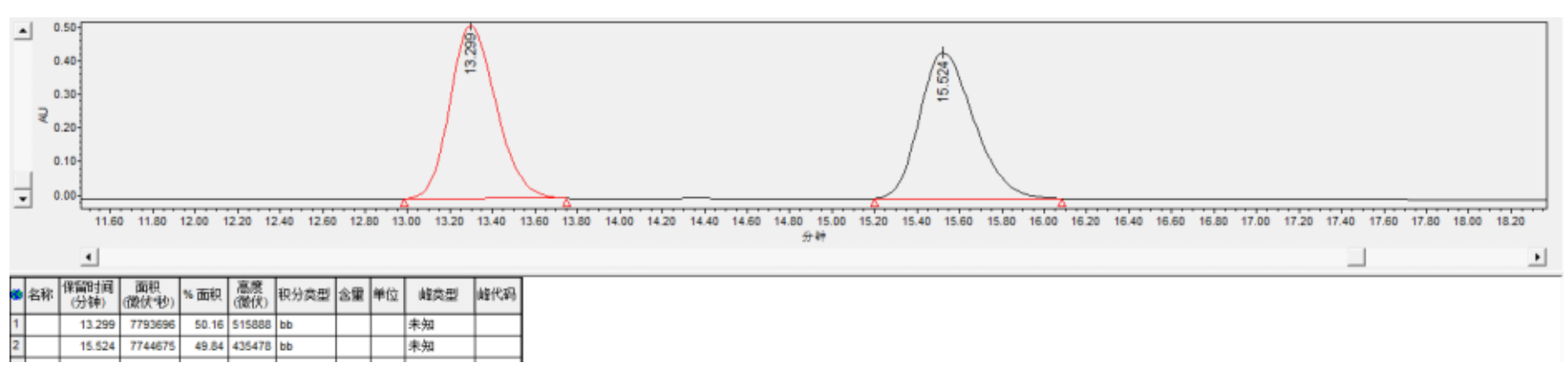

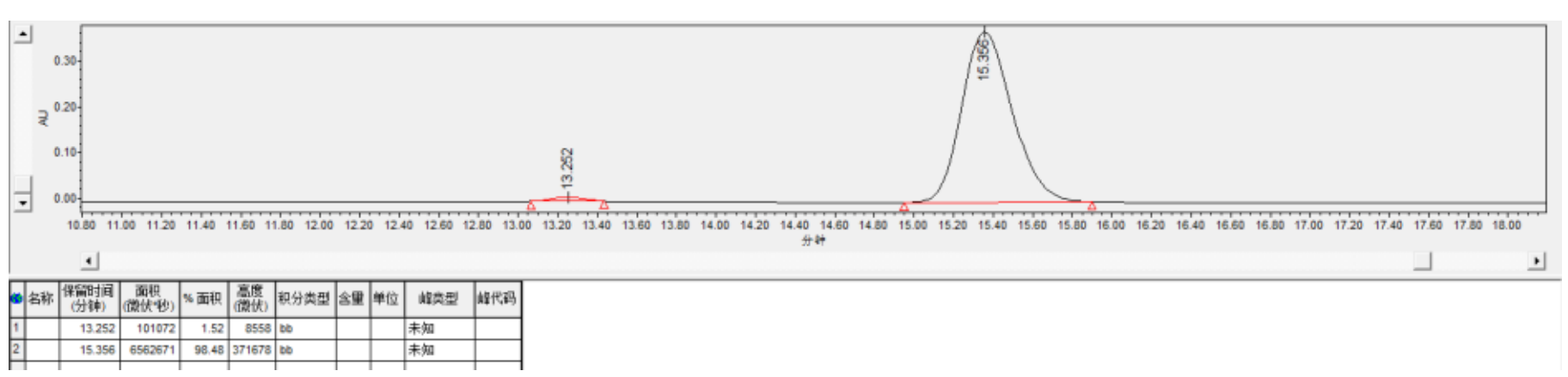

Peaks number	Retention time (min)	Area	Area percentage (%)	Height of peaks
1	13.252	101072	1.52	8558
2	15.356	6562671	98.48	37167

gram synthesis and cycle experiments of 1t:

cycle 1: yield =97.6% (0.9865 g), ee = 97%.

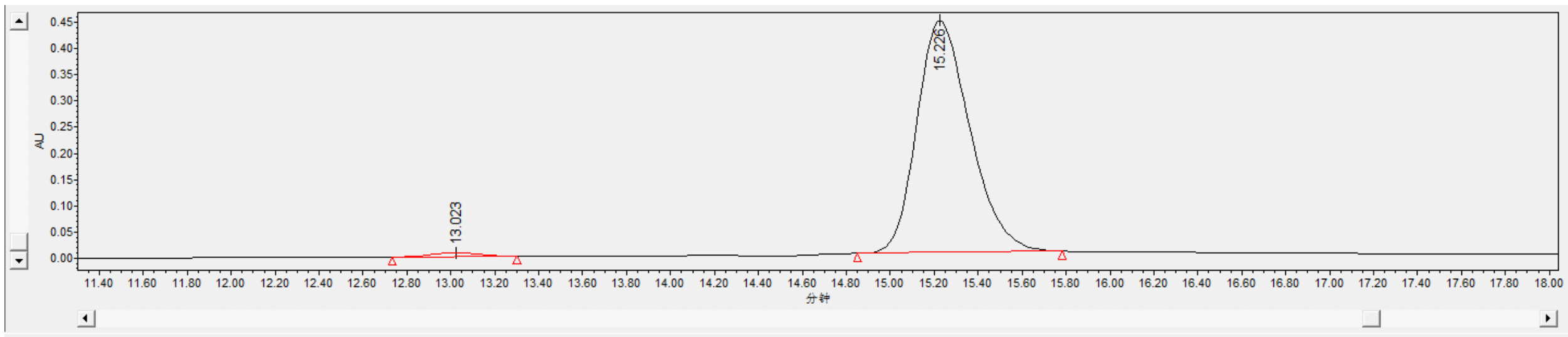

	名称	保留时间 (分钟)	面积 (微伏*秒)	% 面积	高度 (微伏)	积分类型	含量	单位	峰类型	峰代码
1		13.023	113521	1.51	7926	bb			未知	
2		15.226	7425094	98.49	442533	bb			未知	

Peaks number	Retention time (min)	Area	Area percentage (%)	Height of peaks
1	13.023	113521	1.51	7926
2	15.226	7425094	98.49	442533

cycle 2: yield =97.4% (0.9846 g), ee = 96%.

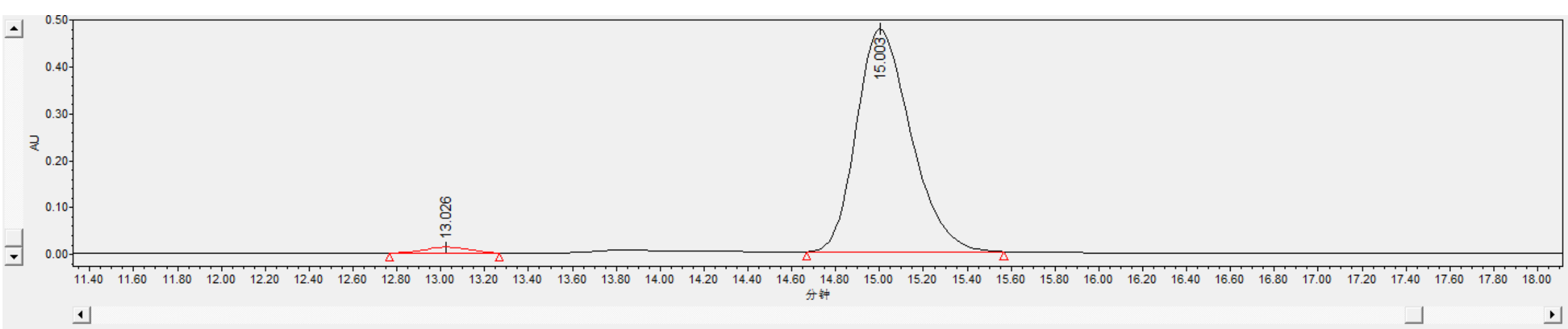

	名称	保留时间 (分钟)	面积 (微伏*秒)	% 面积	高度 (微伏)	积分类型	含量	单位	峰类型	峰代码
1		13.026	167884	2.01	12319	bb			未知	
2		15.003	8198638	97.99	475519	bb			未知	

Peaks number	Retention time (min)	Area	Area percentage (%)	Height of peaks
1	13.026	167884	2.01	12319
2	15.003	8198638	97.99	475519

cycle 3: yield =97.9% (0.9899 g), ee = 96%.

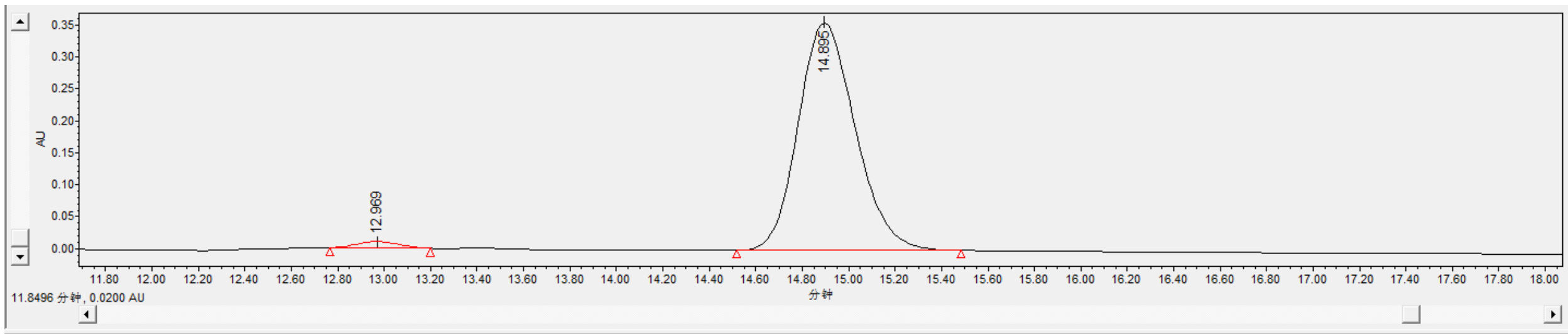

	名称	保留时间 (分钟)	面积 (微伏*秒)	% 面积	高度 (微伏)	积分类型	含量	单位	峰类型	峰代码
1		12.969	124991	2.02	10110	bb			未知	
2		14.895	6057401	97.98	356173	bb			未知	

Peaks number	Retention time (min)	Area	Area percentage (%)	Height of peaks
1	12.969	124991	2.02	10110
2	14.895	6057401	97.98	356173

cycle 4: yield =95.2% (0.9623 g), ee = 95.5%.

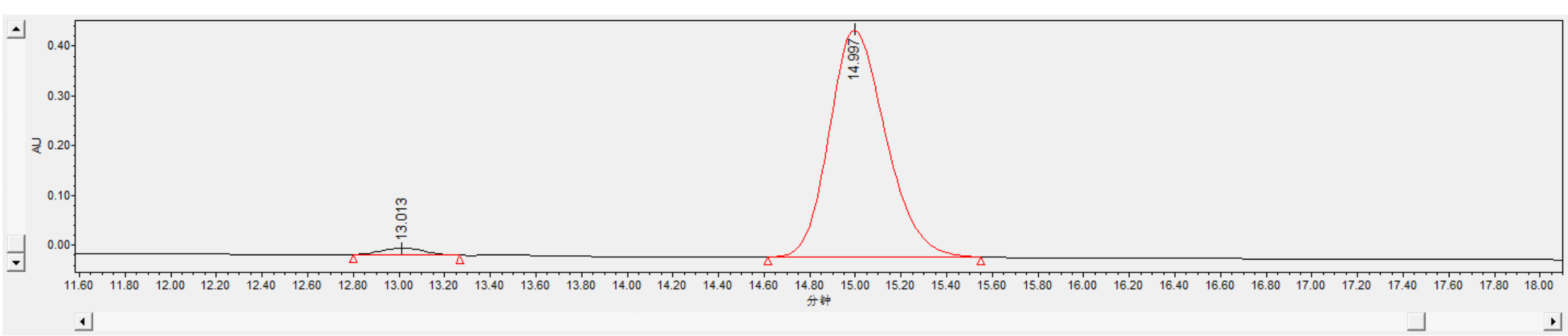

	名称	保留时间 (分钟)	面积 (微伏*秒)	% 面积	高度 (微伏)	积分类型	含量	单位	峰类型	峰代码
1		13.013	180299	2.25	13725	bb			未知	
2		14.997	7850460	97.75	456177	bb			未知	

Peaks number	Retention time (min)	Area	Area percentage (%)	Height of peaks
1	13.013	180299	2.25	13725
2	14.997	7850460	97.75	456177

cycle 5: yield =95.2% (0.9722 g), ee = 95%.

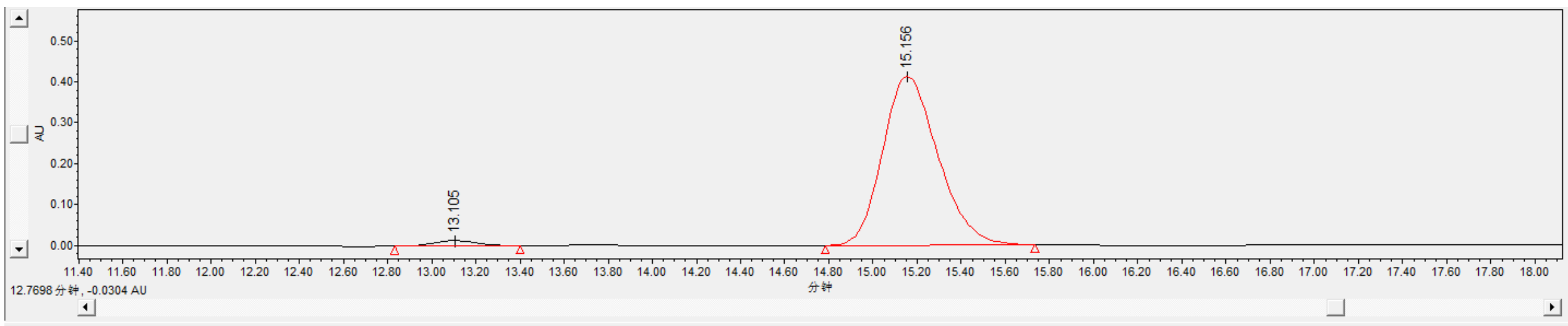

	名称	保留时间 (分钟)	面积 (微伏*秒)	% 面积	高度 (微伏)	积分类型	含量	单位	峰类型	峰代码
1		13.105	184264	2.50	13083	bb			未知	
2		15.156	7179895	97.50	413665	bb			未知	

Peaks number	Retention time (min)	Area	Area percentage (%)	Height of peaks
1	13.105	184264	2.50	13083
2	15.156	7179895	97.50	413665

REFERENCES

1. Willems TF, Rycroft CH, Kazi M, Meza JC, Haranczyk M. Algorithms and tools for high-throughput geometry-based analysis of crystalline porous materials. *Micropor Mesopor Mat.* **149**, 134-141 (2012).

2. Ravel B, Newville M. ATHENA, ARTEMIS, HEPHAESTUS: data analysis for X-ray absorption spectroscopy using IFEFFIT. *J. Synchrotron Radiat.* **12**, 537-541 (2005).

3. Zabinsky SI, Rehr JJ, Ankudinov A, Albers RC, Eller MJ. Multiple-scattering calculations of x-ray-absorption spectra. *Phys. Rev. B.* **52**, 2995-3009 (1995).

4. Funke H, Scheinost AC, Chukalina M. Wavelet analysis of extended x-ray absorption fine structure data. *Phys. Rev. B.* **71**, 094110 (2005).

5. Tan S, Wang K, Zeng Q, Liu Y. Insight into the Nanotribological Mechanism of Two-Dimensional Covalent Organic Frameworks. *ACS Appl. Mater. Interfaces.* **14**, 40173-40181 (2022).

6. Gong Z*, et al.* Structural Variation in Surface-Supported Synthesis by Adjusting the Stoichiometric Ratio of the Reactants. *ACS Nano.* **10**, 4228-4235 (2016).

7. Zheng Z, Yuan C, Sun M, Dong J, Liu Y, Cui Y. Construction of Monophosphine-Metal Complexes in Privileged Diphosphine-Based Covalent Organic Frameworks for Catalytic Asymmetric Hydrogenation. *J. Am. Chem. Soc.* **145**, 6100-6111 (2023).

8. Dong Y*, et al.* Au-Acyclic Diaminocarbene-Linked Homochiral Covalent Organic Frameworks: Synthesis and Asymmetric Catalytic Application. *Chem. Mater.* **37**, 473-479 (2024).

9. Li F, Kan J-L, Yao B-J, Dong Y-B. Synthesis of Chiral Covalent Organic Frameworks via Asymmetric Organocatalysis for Heterogeneous Asymmetric Catalysis. *Angew. Chem., Int. Ed.* **134,** e202115044 (2022).

10. Kang X, Cheng C, Chen X, Dong J, Liu Y, Cui Y. Three-Dimensional Homochiral Covalent Organic Frameworks with Intrinsic Chiral qzd Topology. *J. Am. Chem. Soc.* **146**, 8407-8416 (2024).

11. Li F, Zhang Y-L, Wang H-H, Kan J-L, Yao B-J, Dong Y-B. PyBOX-Integrated Chiral Covalent Organic Frameworks. *Angew. Chem., Int. Ed.* ***64****,* e202505329 (2025).

12. Han X, Xia Q, Huang J, Liu Y, Tan C, Cui Y. Chiral Covalent Organic Frameworks with High Chemical Stability for Heterogeneous Asymmetric Catalysis. *J. Am. Chem. Soc.* **139**, 8693-8697 (2017).

13. Han X*, et al.* Chiral induction in covalent organic frameworks. *Nat. Commun.* **9**, 1294 (2018).

14. Wang J-C*, et al.* Construction of Hollow Structured Covalent Organic Framework with Chiral Internal Catalytic Sites for Asymmetric Hydrogenation. *Small.* **21**, e07775 (2025).

15. Zhang Y*, et al.* Metal-Free Heterogeneous Asymmetric Hydrogenation of Olefins Promoted by Chiral Frustrated Lewis Pair Framework. *J. Am. Chem. Soc.* **146**, 979-987 (2023).

16. Hou B*, et al.* Single-Crystal X-ray Structures of Homochiral Brønsted Acidic Covalent Organic Frameworks. *J. Am. Chem. Soc.* **147**, 12127-12137 (2025).

17. Gong W*, et al.* Leveraging Chiral Zr(IV)-Based Metal-Organic Frameworks To Elucidate Catalytically Active Rh Species in Asymmetric Hydrogenation Reactions. *J. Am. Chem. Soc.* **144**, 3117-3126 (2022).

18. Lin Y, Xu G, Tang W. Chiral Polymeric Diamine Ligands for Iridium-Catalyzed Asymmetric Transfer Hydrogenation. *J. Am. Chem. Soc.* **146**, 27736-27744 (2024).

19. Tao L*, et al.* Efficient Asymmetric Hydrogenation of Quinolines over Chiral Porous

Polymers Integrated with Substrate Activation Sites.*ACS Catal.* **10**, 1783-1791 (2020).

20. Bai S*, et al.* Enhancement of catalytic performance in asymmetric transfer hydrogenation by microenvironment engineering of the nanocage. *Chem Commun.* **46**, 8145-8147 (2010).

21. Jing L, Zhang X, Guan R, Yang H. Construction of a chiral macromolecular catalyst in hollow silica nanoreactors for efficient and recyclable asymmetric catalysis. *Catal. Sci. Technol.* **8**, 2304-2311 (2018).

22. Zhang X, Jing L, Wei L, Zhang F, Yang H. Semipermeable Organic-Inorganic Hybrid Microreactors for Highly Efficient and Size-Selective Asymmetric Catalysis.*ACS Catal.* **7**, 6711-6718 (2017).

23. Zhang K*, et al.* Amphiphilic Hyperbranched Polyethoxysiloxane: A Self-Templating Assembled Platform to Fabricate Functionalized Mesostructured Silicas for Aqueous Enantioselective Reactions.*ACS Catal.* **6**, 6229-6235 (2016).
