# Supplementary material for: Multivariate approach of metal-chiral covalent organic frameworks for leaching-resistant asymmetric transfer hydrogenation
Source: Nat Commun. 2026 Sep 25;17:11147. doi: 10.1038/s41467-026-78066-y (PMC13710755; doi:10.1038/s41467-026-78066-y)
Supplement: Supplementary file 2 [file 41467_2026_78066_MOESM2_ESM.pdf]

nature portfolio

Peer Review File

Multivariate Approach of Metal-Chiral Covalent Organic Frameworks for Leaching-Resistant Asymmetric Transfer Hydrogenation

Corresponding Author: Professor Jiajia Cheng

This file contains all reviewer reports in order by version, followed by all author rebuttals in order by version.

Version 0:

Reviewer comments:

Reviewer #1

(Remarks to the Author)
The integration of chiral environments and catalytic metal sites within covalent organic frameworks (COFs) represents an attractive and timely strategy for asymmetric catalysis. The concept presented by Cheng et at. in this manuscript is interesting and potentially impactful, particularly for the development of heterogeneous, enantioselective catalysts. However, the current version of the manuscript presents substantial weaknesses in experimental description, structural characterization, and data presentation, which significantly limit the reliability of the conclusions.

A major concern is the overall quality and organization of the Supporting Information (SI). The SI is difficult to follow, contains numerous typographical errors, and lacks essential experimental details. The General Methods section should be significantly expanded to include full information on solvents, commercial sources of reagents, purification procedures, and detailed NMR acquisition parameters (field strength, multiplicity, solvent, temperature). At present, the experimental description does not meet the standards of reproducibility expected for this journal.

With respect to the synthesis of (R)-NH2-Boc, the reaction scheme presented on page 4 is unclear and contains several inaccuracies. In particular, the role of the R–Ph compound is not explained, and its relevance to the overall study is uncertain. All compounds shown in this scheme should be properly characterized (1H NMR, 13C NMR, DEPT, HRMS, and chiral HPLC where applicable). If these compounds have been previously reported, appropriate references should be clearly cited, and at minimum 1H NMR and chiral HPLC data should be provided. In general, the quality of the 13C NMR spectra throughout the SI is insufficient.

Regarding the chiral precursor, retention of configuration is a key point for this work. While chiral HPLC analysis is shown, it is unclear how the racemic reference material was prepared for peak assignment. This should be explicitly described. Ideally, the authors should attempt single-crystal X-ray diffraction of the chiral precursor (R)-NH2-Boc, which would provide unambiguous confirmation of absolute configuration.

It is also unclear why the synthesis of (R)-NH2′-Boc is included, as this compound does not appear to be used in the preparation of the COFs. As for (R)-NH2-Boc, a complete characterization of this compound is required.

For the synthesis of the CCOFX materials, an important unresolved issue concerns the stability of the Boc protecting group. The COF synthesis conditions (120 °C, acidic medium, three days) are typically incompatible with Boc protection. The manuscript does not provide convincing evidence that the Boc group remains intact after framework formation.

In addition, the proposed distributions of DATP and (R)-NH2-Boc units in CCOF1 and CCOF2 cannot be experimentally verified without single-crystal data. At a minimum, the authors should provide clear theoretical structural models for each material, including full atomic positions and not only AA stacking motifs. The computational methods used to generate these models should be described in detail. Pawley or Le Bail refinements should be performed to demonstrate that alternative structural models can be excluded. Theoretical pore volumes and BET surface areas should also be provided for comparison with experimental values.

Several aspects of the materials characterization require improvement:
• Complete elemental analysis (C, H, N, S) should be reported for all COFs, rather than only N:S ratios.
• The nitrogen adsorption isotherms show very low surface areas compared to those expected from the proposed structures, suggesting a significant fraction of amorphous material or impurities.
• This interpretation is supported by the TGA data, which show unusually high residual masses (50–60%) at 800 °C for COF materials, indicating the presence of substantial inorganic or metallic residues.
• SEM and TEM images in the SI (Figures S5, S9, S10, and S13) are of limited quality and should be improved.
Furthermore, TEM elemental mapping suggests an inhomogeneous distribution of elements, which contradicts the assumed uniformity of the materials.
The proposed catalytic mechanism is largely speculative and not supported by experimental or computational evidence. Given the current level of structural and compositional uncertainty of the materials, such mechanistic proposals appear premature.
The presentation of the Supporting Information also requires attention: figure sizes are highly inconsistent, with some non-essential figures being overly large, while key data are difficult to read due to small size or poor resolution.

Several specific points require clarification:
• The rationale for incorporating Rh and Ir into the same materials should be clearly explained.
• The nature of TFPB-DATP-Ru needs to be defined: how is Ru coordinated to the DATP ligand, and how has this been experimentally confirmed?
• The identity and characterization of (R)-Ph-Ru should be clarified.
For the substrate scope shown in Figure 5e, the origin of the racemic substrates must be specified (commercial vs synthesized). All catalytic products should be appropriately characterized: new compounds require full spectroscopic characterization, while known compounds should be supported by 1H NMR data and proper literature references.

Finally, the discussion of Ru leaching should be reconsidered. Although a leaching value of 0.27 wt% is reported, the initial Ru loading is 0.83 wt% (Table S8), corresponding to approximately 33% metal loss after catalysis. This level of leaching is significant and should be discussed more critically.

In summary, while the conceptual framework of this study is interesting, the current manuscript suffers from insufficient characterization, lack of clarity in experimental procedures, and overinterpretation of the catalytic results. This manuscript does not meet the standards and requirements for publication in Nature Communications.

Reviewer #2

(Remarks to the Author)
This study illustrates the synthesis of three CCOFs, including one two-component and two three-component systems. Ruthenium metallization afforded CCOF-2-Ru, which catalyzes asymmetric transfer hydrogenation of ketones. The authors claim that this work establishes a versatile paradigm for MCCOF design and addresses key challenges in heterogeneous asymmetric catalysis. However, this claim is not well supported. Similar mixed-linker strategies have been reported several years ago and are now well studied. Therefore, I cannot recommend its publication in Angewandte Chemie.
In addition, several issues need to be addressed before consideration in other journals:
1. Only two mixed-linker CCOFs are reported, which cannot be considered true multivariate assemblies. Moreover, the PXRD simulations are poor (Rwp and Rw values are quite high). High-resolution TEM images should be provided to substantiate the proposed structures, especially given the authors' claim of high crystallinity.
2. The catalytic study includes only ten typical substrates for asymmetric transfer hydrogenation, a well-established reaction in both homogeneous and heterogeneous systems. The observed activity and enantioselectivity are comparable to previously reported heterogeneous catalysts, offering limited advancement.

Reviewer #3

(Remarks to the Author)
This manuscript presents a multivariate assembly strategy combined with post-synthetic metallization to construct metal-chiral covalent organic frameworks (MCCOFs) for asymmetric transfer hydrogenation of ketones. The work addresses issues such as structural instability, metal leaching, and limited recyclability, and reports notable catalytic results including high yield and enantioselectivity. However, several key aspects require clarification and additional data to meet the standards of Nature Communications, as outlined below. Major revision is therefore recommended.

1.The crystallinity of CCOF-0-Boc is lower compared to CCOF-1-Boc and CCOF-2-Boc. Could this be due to partial Boc deprotection during the acidic solvothermal synthesis of CCOF-0-Boc, which alters the catalytic environment and pH, whereas in CCOF-2-Boc, the lower Boc content leads to less pH variation and thus better crystallinity and stability?
2.The authors claim that the dense chiral diamine ligands can “recapture dissociated Ru species.” Please provide evidence to support this hypothesis.
3.In the XRD pattern of CCOF0-Boc, the (100) reflection is strong while the (001) reflection is absent, yet the HRTEM image only shows lattice fringes corresponding to the (001) plane (0.48 nm spacing). This differs from the HRTEM results of CCOF1-Boc and CCOF2-Boc. Please clarify this discrepancy.
4.The statement “Thermogravimetric analysis (TGA) demonstrated thermal stability, with ~200 °C mass loss in Boc-protected CCOFs attributed to deprotection, absent in deprotected analogues (Supplementary Fig. 6), verifying complete Boc removal” is unclear. Please rephrase or explain.

5.After treatment with 1 M KOH, the XRD peak of CCOF1 appears sharper (narrower FWHM), suggesting improved crystallinity. Similarly, treatment with 1 M HCOONa seems to sharpen the XRD peaks of CCOF2. What is the reason for this?
6.Please clearly label the peak positions in the XRD patterns of CCOF1, CCOF2 and their corresponding Ru-loaded analogues (CCOF1-Ru and CCOF2-Ru).
7.In the N 1s XPS spectra, why is the N-H signal much stronger in (R)-Ph-Ru than in CCOF2-Ru? Why is there no clear metal-N signal? Please supplement with Raman spectroscopy to verify M-N bonding.
8.There appear to be significant errors in the synthetic schemes for compound 5 and (R)-Ph in Section 2 (Synthetic procedures).
9.CCOF0-Boc was synthesized at 100°C for 3 days, while CCOF1-Boc and CCOF2-Boc were synthesized at 120°C for 3 days. Could the lower crystallinity of CCOF0-Boc be related to the reaction temperature? Would synthesis at 120°C improve its crystallinity?
10.In Supplementary Fig. 11 (FTIR spectra of CCOFX-Ru), please label the characteristic peaks. is there C=O signal remain?
11.Please supplement with PXRD and FTIR data after several catalytic cycles to better demonstrate structural retention under reaction conditions.
12.In situ or operando spectroscopic studies (e.g., XAS) would help elucidate the coordination environment and evolution of Ru species during catalysis.

Version 1:

Reviewer comments:

Reviewer #1

(Remarks to the Author)
The authors have made a substantial effort to address the concerns raised during the previous round of review. In particular, the revised Supplementary Information has been significantly improved, and the characterization of both the synthesized precursors and the post-catalysis compounds is now considerably more complete. The authors have clarified many of the ambiguities present in the original submission, and the overall quality of the manuscript has clearly benefited from these revisions.

However, despite these significant improvements, my main concern regarding the structural characterization of the reported CCOFs remains unresolved.

The crystallinity of the described CCOFs, together with the limited quality and resolution of the PXRD data, is still insufficient to unambiguously support the structural assignments proposed by the authors. In particular, the structural evidence provided does not justify definitive claims regarding the framework arrangement.

Furthermore, the authors justify the low porosity of the materials by stating that “in 2D COFs bearing such bulky side chains, adjacent layers often undergo sliding or staggered stacking to minimize severe interlayer steric repulsion.” This explanation appears to contradict their proposed AA-eclipsed structural model. The optimized theoretical values reported in Supplementary Table 2 (which I assume correspond to the AA-eclipsed structure, including the complexed species) are significantly higher than the experimentally observed values. If the authors attribute the reduced porosity to layer sliding or staggered stacking, then they should provide and compare optimized AB-staggered structural models, as these would be more consistent with their own interpretation of the observed porosity.

At present, while the catalytic results convincingly demonstrate that the CCOF materials are catalytically active, and notably that CCOF2-Ru affords very high enantioselectivities, the actual structures of the catalytic materials cannot be conclusively established. This is due to several unresolved issues:
1- The low crystallinity of the materials;
2- The insufficient resolution of the PXRD patterns;
3- The absence of appropriate structural refinement (e.g., Rietveld refinement);
4- The significant discrepancy between the experimental porosity values and the optimized theoretical values reported by the authors.

Therefore, although the manuscript has been substantially improved and the catalytic performance is certainly interesting, I do not believe that the structural characterization currently reaches the level of rigor expected for publication in Nature Communications. In my opinion, the work would be more appropriate for publication in a specialized catalysis journal, where the catalytic findings could still be appreciated, even if the structural characterization remains less definitive than required for a high-impact general journal.

Reviewer #2

(Remarks to the Author)
I think this revise manuscript can be published now.

Reviewer #3

(Remarks to the Author)
I checked their manuscript and related files. Authors are revised their manuscript significantly. Now I'd like to recommend it for publication in current version.

Version 2:

Reviewer comments:

Reviewer #3

(Remarks to the Author)
I checked the whole manuscript again and agreed with the reviewer 1 that the structures of the COFs are unclear. The authors should check the related characterizations carefully. The authors should discuss with the structure experts about their results.
1. At present, the PXRD results do not clearly indicate that the synthesized COF is crystalline, particularly with regard to the diffraction peaks from the (100) crystal plane. It is recommended to further characterize the structure of the COF using small-angle X-ray diffraction (SAXRD) analysis.
2. It is recommended to plot the solid-state NMR spectra separately; the current plotting format makes it difficult to distinguish the characteristic peaks of the various functional groups. Additionally, it is necessary to perform SSNMR characterization of the monomers prior to synthesis.
3. The TGA results should be confirmed again.

Reviewer #4

(Remarks to the Author)
The authors have addressed all reveiwers' comments very well. I strongly support the publication of this manuscript.

Open Access This Peer Review File is licensed under a Creative Commons Attribution 4.0 International License, which permits use, sharing, adaptation, distribution and reproduction in any medium or format, as long as you give appropriate credit to the original author(s) and the source, provide a link to the Creative Commons license, and indicate if changes were made.
In cases where reviewers are anonymous, credit should be given to 'Anonymous Referee' and the source.
The images or other third party material in this Peer Review File are included in the article’s Creative Commons license, unless indicated otherwise in a credit line to the material. If material is not included in the article’s Creative Commons license and your intended use is not permitted by statutory regulation or exceeds the permitted use, you will need to obtain permission directly from the copyright holder.
To view a copy of this license, visit https://creativecommons.org/licenses/by/4.0/

Response to the comments

Reviewer 1:

The integration of chiral environments and catalytic metal sites within covalent organic frameworks (COFs) represents an attractive and timely strategy for asymmetric catalysis. The concept presented by Cheng et at. in this manuscript is interesting and potentially impactful, particularly for the development of heterogeneous, enantioselective catalysts. However, the current version of the manuscript presents substantial weaknesses in experimental description, structural characterization, and data presentation, which significantly limit the reliability of the conclusions. In summary, while the conceptual framework of this study is interesting, the current manuscript suffers from insufficient characterization, lack of clarity in experimental procedures, and overinterpretation of the catalytic results. This manuscript does not meet the standards and requirements for publication in Nature Communications.

Response: We sincerely appreciate the reviewer for their careful, critical, and constructive comments on our manuscript. We have supplemented the main text and Supporting Information with detailed experimental descriptions and extensive characterization procedures, including Raman spectroscopy and X-ray absorption spectroscopy (XAS). Additional control experiments and deuterium labeling experiments have been performed, and the experimental results have been discussed with deeper analysis. We have carefully revised the parts involving overinterpretation of the catalytic results. All ambiguous figures in the original manuscript and Supporting Information have been remeasured, reanalyzed, and carefully revised. Detailed experimental procedures, key NMR, and high-resolution mass spectrometry data have also been added to the Supporting Information. Furthermore, the language and formatting of the manuscript have been further polished and optimized. We have made every effort to thoroughly improve this work, and sincerely hope that it now meets the high standards and requirements for publication in *Nature Communications*.

Comment 1: A major concern is the overall quality and organization of the Supporting Information (SI). The SI is difficult to follow, contains numerous typographical errors, and lacks essential experimental details. The General Methods section should be significantly expanded to include full information on solvents, commercial sources of reagents, purification procedures, and detailed NMR acquisition parameters (field strength, multiplicity, solvent, temperature). At present, the experimental description does not meet the standards of reproducibility expected for this journal;

Response: We greatly appreciate the reviewer's kind and helpful suggestion. We have carefully proofread the entire SI to correct all typographical errors and supplement the missing experimental details throughout the document. Comprehensive information on solvents, commercial sources of reagents, and purification procedures has been substantially expanded. Furthermore, detailed NMR acquisition parameters have been fully updated and elaborated to ensure experimental reproducibility.

Manuscript Change:

Supporting Information (Section 1. General Methods and Section 2. Synthetic Procedures):

We have updated the SI to include full vendor details, solvent purification methods, and complete NMR parameters (e.g., 600 MHz, DMSO-D6, specific temperatures, and multiplicities).

Section 1. General methods

Chemicals

1,3,5-Tris(4-formylphenyl)benzene (TFPB), 4,4"-diamino-*p*-terphenyl (DATP), 2,5-dibromobenzenesulfonyl chloride, (1*R*,2*R*)-1,2-diphenylethane-1,2-diamine, (±)-1,2-diphenylethane-1,2-diamine, (1*R*,2*R*)-cyclohexane-1,2-diamine, (4,4,5,5-tetramethyl-1,3,2-dioxaborolan-2-yl)benzene, trifluoroacetic acid (TFA), 4-(4,4,5,5-tetramethyl-1,3,2-dioxaborolan-2-yl)aniline, di-tert-butyl dicarbonate (Boc_2O), trimethylsilyl trifluoromethanesulfonate (TMSOTf), $[Ru(p\text{-cymene})Cl_2]_2$, $[Ru(\eta^6\text{-}C_6H_6)Cl_2]_2$, $[CpRhCl_2]_2$, $[CpIrCl_2]_2$, tetrakis(triphenylphosphine)palladium(0) $[Pd(PPh_3)_4]$, *n*-butanol, ethyl acetate, petroleum ether, dichloromethane, sodium formate, ammonium formate, acetophenone, and other ketone derivatives were purchased from Energy Chemical and used as received. 1,4-Dioxane, potassium bromide, anhydrous ethanol, tetrahydrofuran (THF), acetic acid, potassium carbonate (K_2CO_3), triethylamine, isopropanol, sodium hydroxide, potassium hydroxide, and anhydrous sodium sulfate were obtained from Sinopharm Chemical Reagent Co., Ltd. All other commercial reagents were used as received unless otherwise noted.

Characterization

The IR (KBr pellet) spectra were recorded on a Nicolet Magna 750 FT-IR spectrometer. Thermogravimetric analyses (TGA) were carried out in an N_2 atmosphere with a heating rate of 10 °C/min on a STA449C integration thermal analyzer. Scanning electron microscopy (SEM) images were obtained on a Hitachi SU8000 and a Thermo Scientific Verios G4 scanning electron microscope. Powder X-ray diffraction (PXRD)

data were collected on a D8 Advance diffractometer using Cu Kα radiation. Transmission electron microscopy (TEM) images were obtained on a JEM-2100 instrument. Raman spectra were measured using an InVia Reflex 1900099S spectrometer. Elemental analysis of the COFs was performed on an Elementar Vario EL Cube analyzer. The molecular masses of the compounds were determined by LC-MS (AB Sciex ZenoTOF 7600). XPS spectra were obtained using an X-ray photoelectron spectrometer (Thermo Scientific ESCALAB QXi). Liquid ^{1}H, ^{13}C, DEPT, and ^{19}F NMR spectra were recorded on a Bruker AVANCE III 400 MHz NMR spectrometer. Solid-state ^{13}C NMR experiments were carried out on a Bruker AVANCE III HD 500 NMR spectrometer. Analytical high-performance liquid chromatography (HPLC) was performed on a Waters 1525 instrument with UV detection. N_2 adsorption isotherms of COFs were measured using a Micromeritics ASAP 2020 system at 77 K with a liquid N_2 bath. The structural models of the COF were constructed using the Materials Studio software package. The unit cell parameters were optimized using the Forcite module with molecular mechanics calculations employing the Universal force field. Pawley refinements were performed with the Reflex module implemented in Materials Studio. The theoretical BET surface areas and pore volumes were calculated directly from the simulated structures using Zeo++.[1] X-ray Absorption Spectroscopy (XAS) Measurements. Ru K-edge analysis was performed with a Si(311) crystal monochromator at the BL13ssw beamline at the Shanghai Synchrotron Radiation Facility (SSRF) (Shanghai, China). Before the analysis at the beamline, samples were pressed into thin sheets of 1 cm in diameter and sealed using Kapton tape film. The XAFS spectra were recorded at room temperature using a 4-channel Silicon Drift Detector (SDD) Bruker 5040. Ru K-edge extended X-ray absorption fine structure (EXAFS) spectra were recorded in transmission mode. Negligible changes in the line-shape and peak position of the Ru K-edge XANES spectra were observed between two scans taken for a specific sample. The XAFS spectra of the standard reference samples (Ru foil, $RuCl_3$, and RuO_2) were recorded in transmission mode. The XAFS data were processed according to standard procedures using the Athena module implemented in the IFEFFIT software package. The EXAFS spectra were obtained by subtracting the post-edge background from the overall absorption and then normalizing with respect to the edge-jump step. Subsequently, the $\chi(k)$ data were Fourier transformed to real (R) space using a Hanning window ($dk = 1.0$ Å^{-1}) to separate the EXAFS contributions from different coordination shells. To obtain the quantitative structural parameters around the central atoms, least-squares curve parameter fitting was performed using the ARTEMIS module of the IFEFFIT software package.[2, 3] For Wavelet Transform (WT) analysis, the $\chi(k)$ data exported from Athena were imported into the Hama Fortran

code.[4] The parameters were listed as follows: R-range, 0.0–4.0 Å; k-range, 0–16.0 $Å^{-1}$ for the sample and standards; k-weight, 2 (chosen to balance the visual resolution of light and heavy scatterers in the 2D contour plots); and the Morlet function with κ = 8, σ = 1 was used as the mother wavelet to provide the overall distribution.

Reference:

1. Willems TF, Rycroft CH, Kazi M, Meza JC, Haranczyk M. Algorithms and tools for high-throughput geometry-based analysis of crystalline porous materials. Micropor Mesopor Mat. 149, 134-141 (2012).
2. Ravel B, Newville M. ATHENA, ARTEMIS, HEPHAESTUS: data analysis for X-ray absorption spectroscopy using IFEFFIT. J. Synchrotron Radiat. 12, 537-541 (2005).
3. Zabinsky SI, Rehr JJ, Ankudinov A, Albers RC, Eller MJ. Multiple-scattering calculations of x-ray-absorption spectra. Phys. Rev. B. 52, 2995-3009 (1995).
4. Funke H, Scheinost AC, Chukalina M. Wavelet analysis of extended x-ray absorption fine structure data. Phys. Rev. B. 71, 094110 (2005).

2.5. Synthesis of (*R*,*R*)-TP-DPEN-Ru:

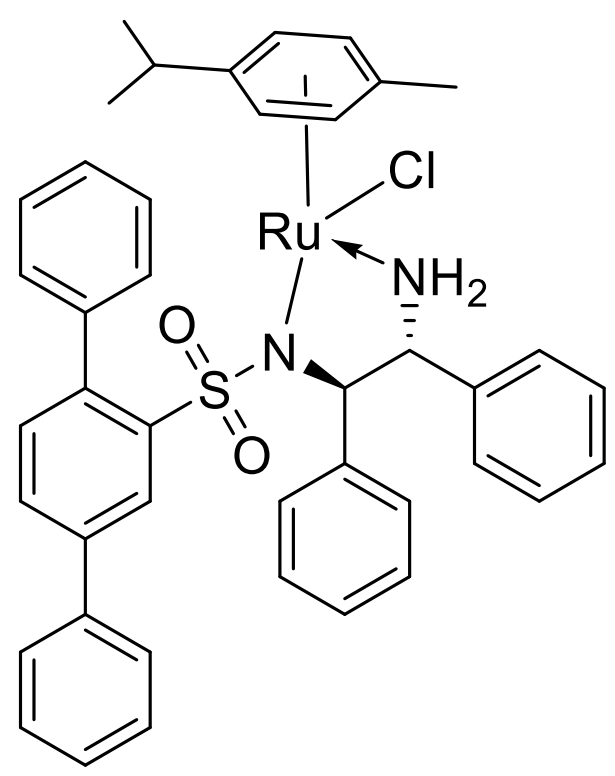

A mixture of $[Ru(p\text{-cymene})Cl_2]_2$ (153 mg, 0.25 mmol), (*R*,*R*)-TP-DPEN (504 mg, 1 mmol), and TEA (0.14 mL, 1 mmol) in CH_2Cl_2 (10 mL) was stirred at room temperature for 1 h under a N_2 atmosphere. The mixture was then washed with water and dried over anhydrous Na_2SO_4. Removal of the solvent under reduced pressure afforded the crude product. Purification by flash column chromatography on silica gel using dichloromethane/methanol (100:1, v/v) as the eluent gave the pure product as an orange-yellow powder (264 mg, 85% yield). ^1H NMR (600 MHz, ACETONITRILE-D_3) δ 8.04 – 7.94 (s, 1H), 7.66 – 7.45 (m, 6H), 7.44 – 7.31 (m, 6H), 7.10 – 7.04 (m, 3H), 6.90 – 6.84 (d, *J* = 7.3 Hz, 3H), 6.67 – 6.57 (s, 5H), 6.01 – 5.92 (s, 1H), 5.57 – 5.52 (s, 1H), 5.17 – 5.09 (s, 1H), 5.02 – 4.95 (s, 1H), 3.90 – 3.81 (d, *J* = 11.2 Hz, 1H), 3.75 – 3.68 (t, *J* = 12.6 Hz, 1H), 3.45 – 3.34 (t, *J* = 11.8 Hz, 1H), 3.01 – 2.93 (s, 1H), 2.27 –

2.23 (s, 3H), 1.34 – 1.27 (dd, J = 20.0, 7.0 Hz, 6H); ESI-HRMS: Found: m/z 739.1912. Calcd for $C_{42}H_{41}N_2O_2RuS$: $(M-Cl)^+$ 739.1927.

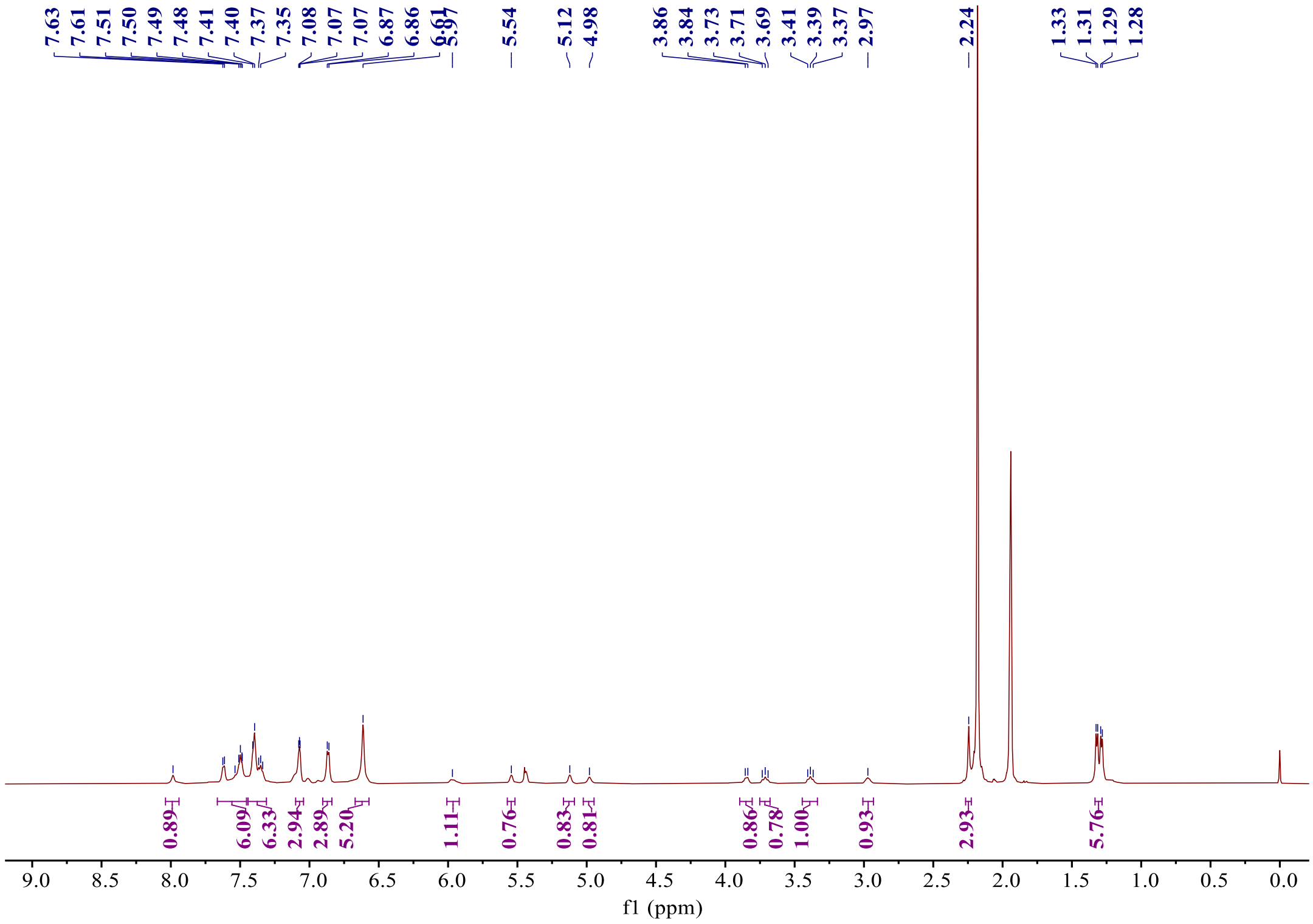

2.6. Synthesis of (*R*,*R*)-DACH-NH-Boc (9) (*tert*-butyl ((1*R*,2*R*)-2-(4,4''-diamino-[1,1':4',1''-terphenyl]-2'-sulfonamido)cyclohexyl)carbamate):

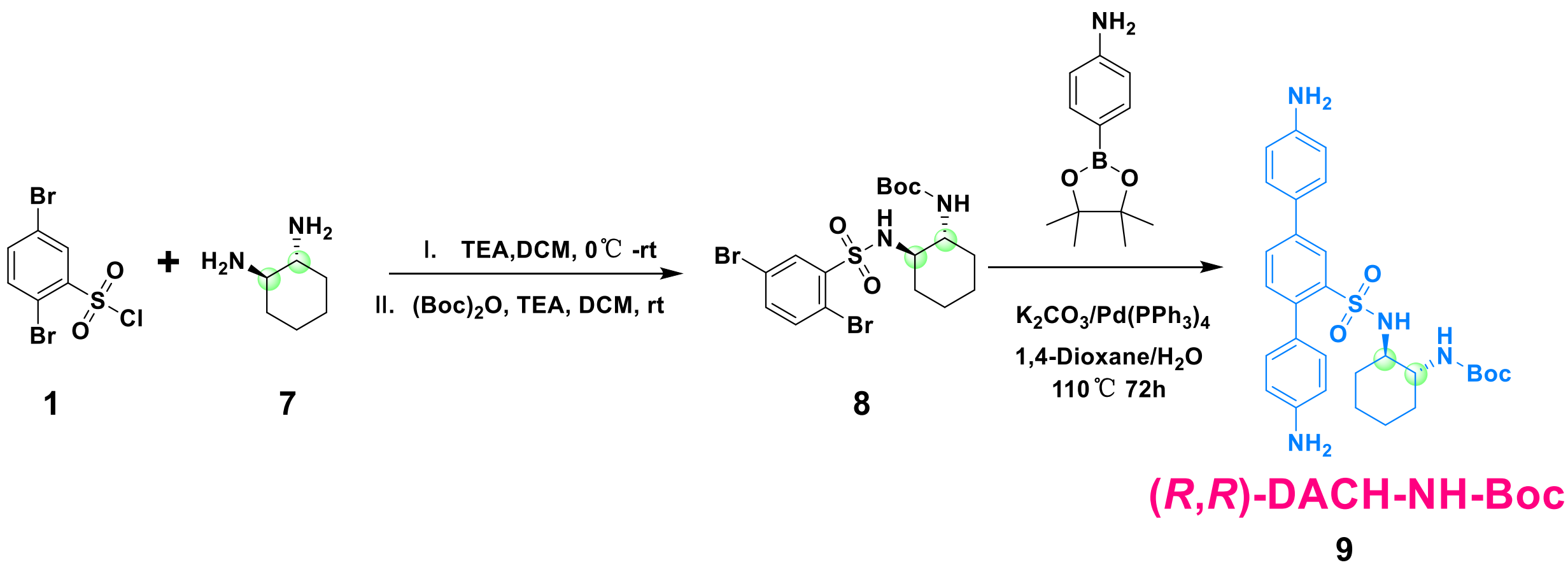

The synthetic procedure for (*R*,*R*)-DACH-NH-Boc (**9**) is analogous to that of (*R*,*R*)-DPEN-NH-Boc, requiring only the substitution of (1*R*,2*R*)-1,2-diphenylethane-1,2-diamine (**2**) with (1R,2R)-cyclohexane-1,2-diamine (**7**).

(1*R*,2*R*)-cyclohexane-1,2-diamine (**7**, 1.7 g, 14.9 mmol) was dissolved in 60 mL of DCM, and TEA (4.0 mL, 28.7 mmol) was added dropwise with stirring at 0 °C. A solution of 2,5-dibromobenzenesulfonyl chloride (**1**, 5.0 g, 14.9 mmol) in 20 mL of DCM was added slowly to the mixture at 0 °C. After stirring at 0 °C for 30 min, the

reaction mixture was warmed to room temperature and stirred for 4 h. Then, the mixture was cooled back to 0 °C, and an additional 4.0 mL of triethylamine was added, followed by slow addition of Boc_2O (4.0 mL, 17.3 mmol). The reaction was then warmed to room temperature and stirred overnight. The organic solvent was removed under reduced pressure, and the residue was acidified to pH = 2 with 1 M aqueous $KHSO_4$ and extracted with DCM. The combined organic layers were washed with saturated aqueous brine, dried over anhydrous Na_2SO_4, and concentrated under vacuum. Purification was performed via column chromatography on silica gel (200–300 mesh) using PE/EA (4:1, v/v) as the eluent. The product was dried under vacuum at 60 °C for 12 h to afford 6.3 g of white solid (**8**) in 83% yield. ^{1}H NMR (600 MHz, DMSO-D_6) δ 8.05 – 8.03 (d, J = 2.4 Hz, 1H), 7.86 – 7.83 (d, J = 8.8 Hz, 1H), 7.71 – 7.65 (m, 2H), 6.10 – 6.03 (d, J = 8.9 Hz, 1H), 3.21 – 3.13 (qd, J = 10.3, 4.0 Hz, 1H), 2.99 – 2.90 (dtd, J = 14.5, 9.6, 4.2 Hz, 1H), 1.70 – 1.65 (m, 1H), 1.61 – 1.56 (m, 1H), 1.52 – 1.46 (m, 2H), 1.33 – 1.30 (s, 9H), 1.30 – 1.24 (dd, J = 12.5, 2.9 Hz, 1H), 1.17 – 0.94 (m, 2H); ^{13}C NMR (151 MHz, DMSO-D_6) δ 24.6, 24.8, 28.8, 32.6, 33.4, 53.4, 56.8, 78.0, 119.1, 121.1, 133.2, 136.6, 137.5, 143.1, 155.8; DEPT 135° NMR (151 MHz, DMSO-D_6) δ 24.6, 24.8, 28.8, 32.6, 33.4, 53.4, 56.8, 133.2, 136.5, 137.5; ESI-HRMS: Found: m/z 532.9726. Calcd for $C_{17}H_{24}Br_2N_2O_4S$: $(M+Na)^+$ 532.9713.

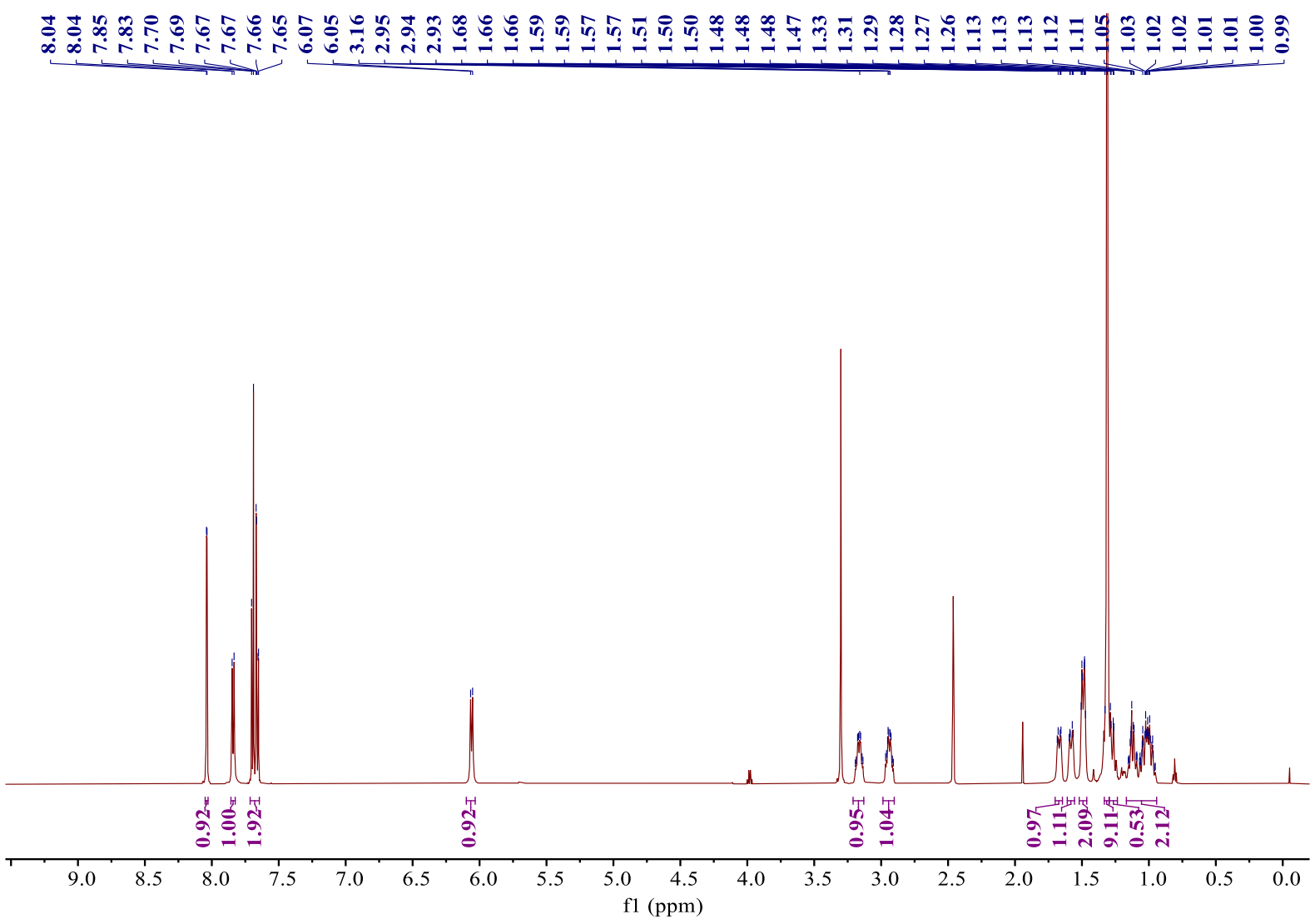

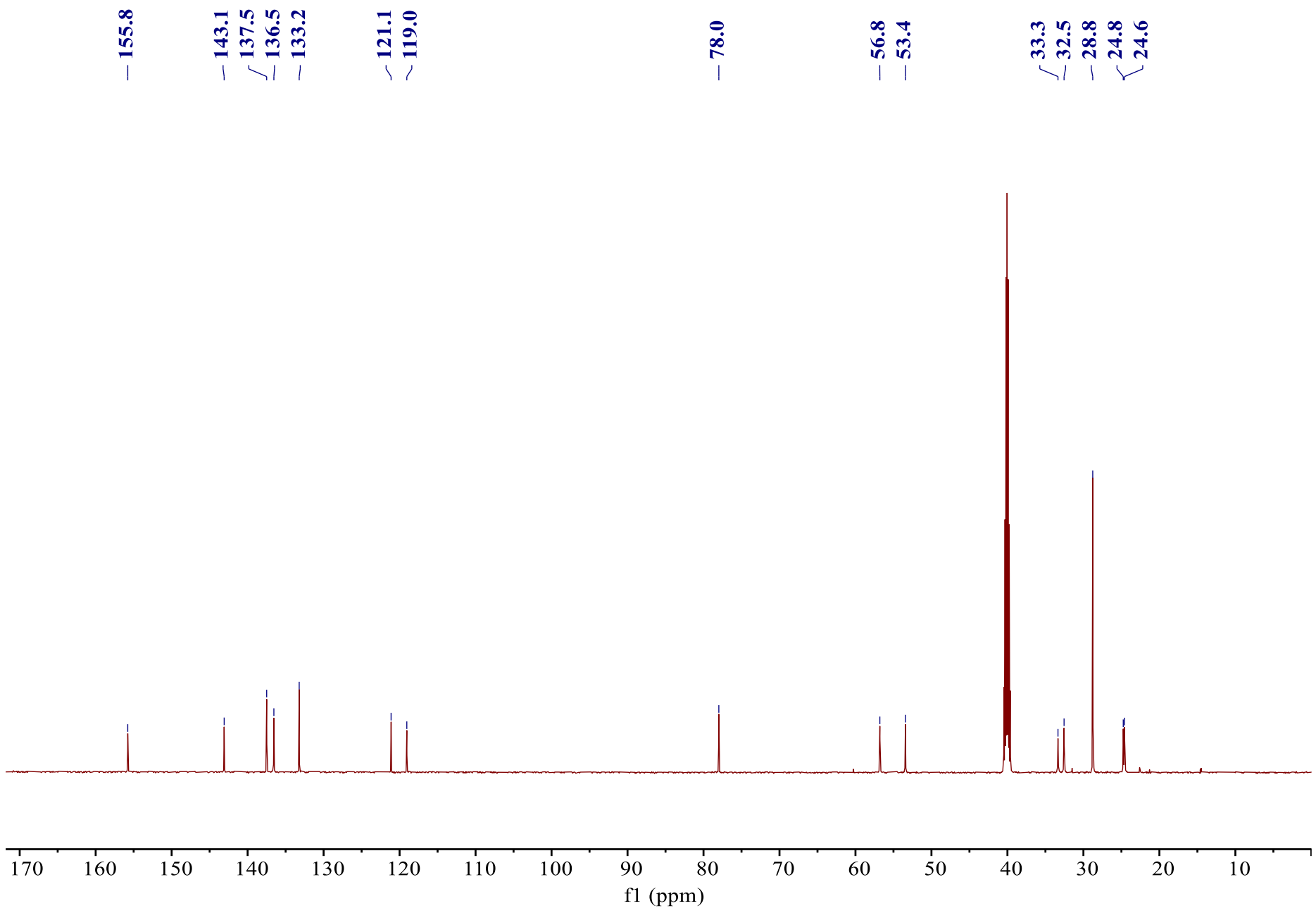

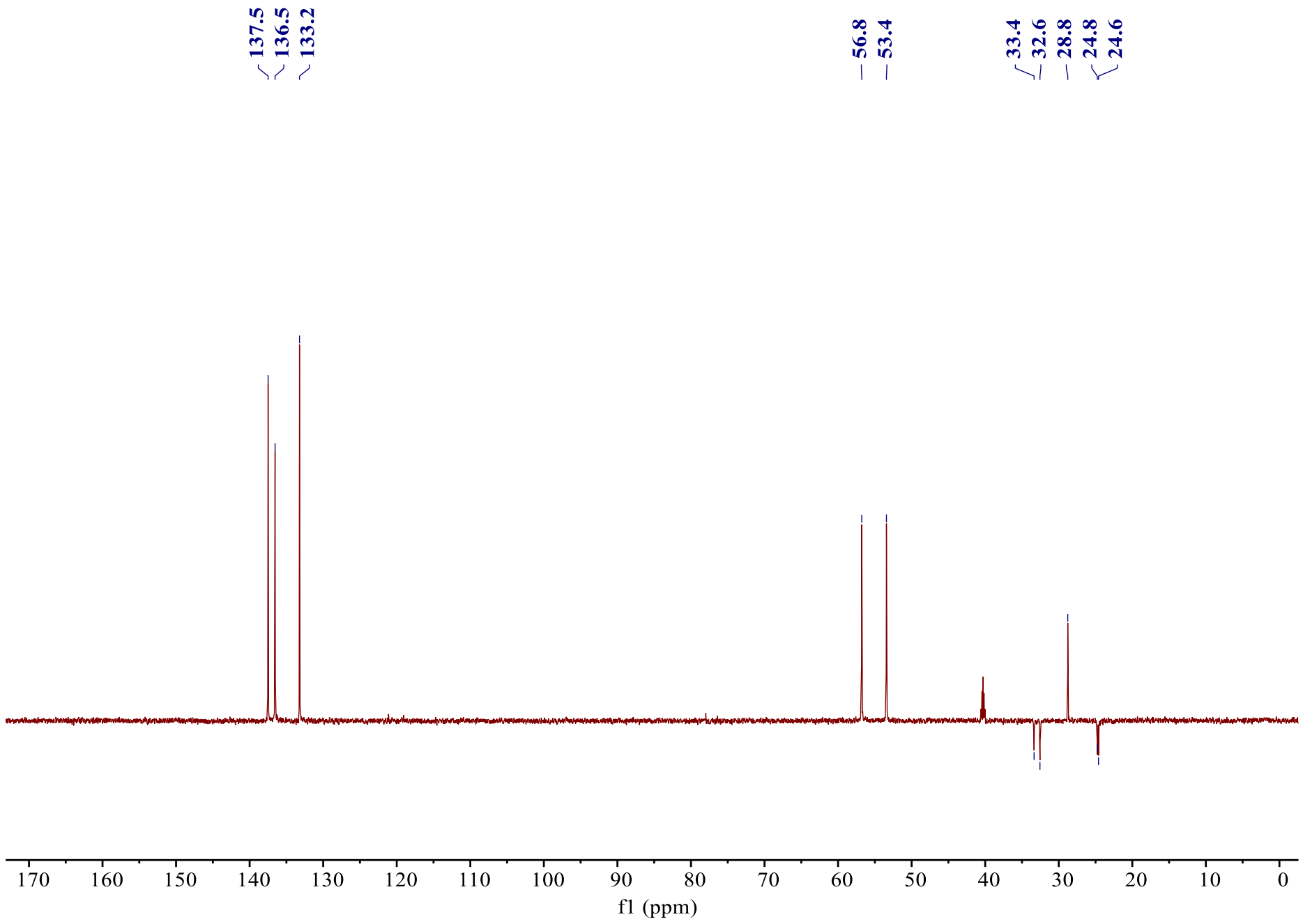

The ee-value of compound **8** was determined by HPLC analysis with a 25 cm × 4.6 mm Daicel Chiralcel IC column (hexane/i-PrOH = 90/10, 1.0 mL/min). t_{minor} = 11.2 min, t_{major} = 9.7 min; ee = 99.8 %. The racemic sample used in the HPLC analysis was synthesized by replacing (1*R*,2*R*)-cyclohexane-1,2-diamine with (±)-*trans*-1,2-diaminocyclohexane under the identical synthetic procedure.

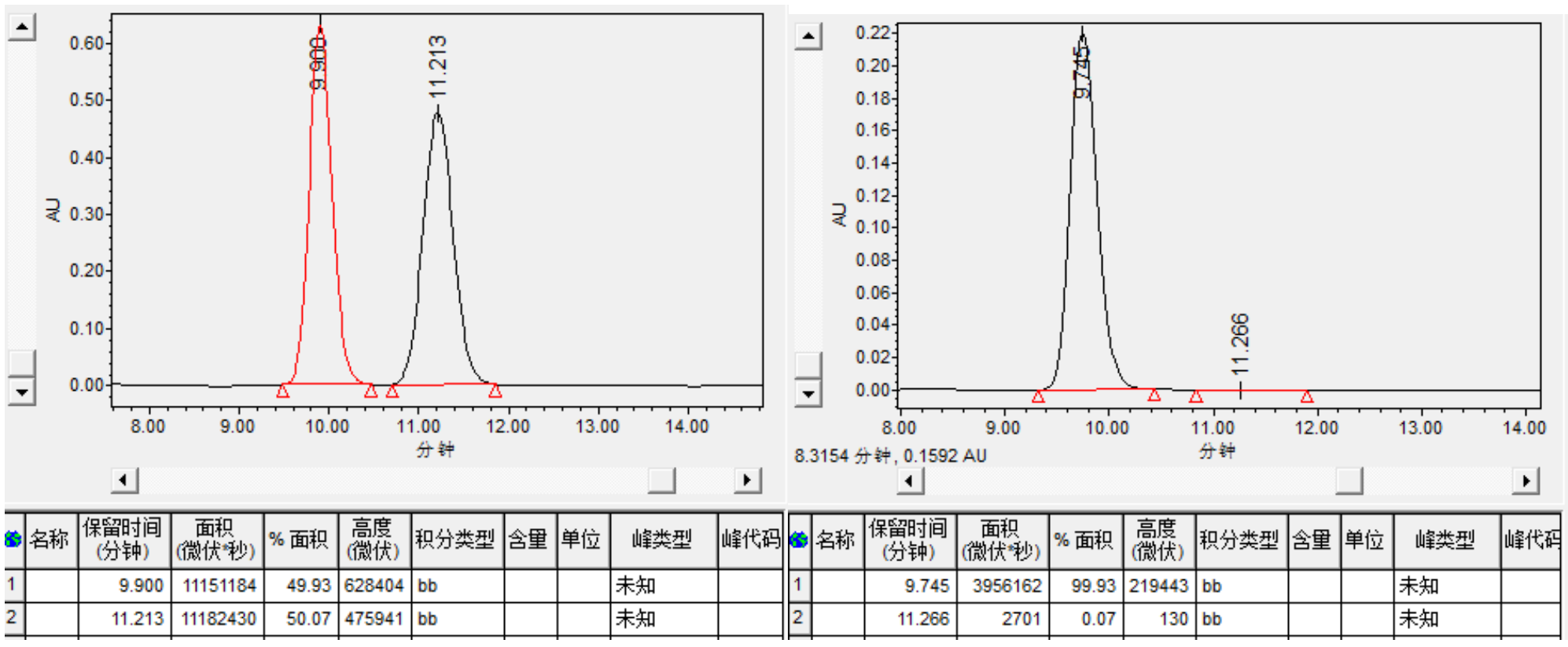

Peaks number	Retention time (min)	Area	Area percentage (%)	Height of peaks
1	9.745	3956162	99.93	219443
2	11.266	2701	0.07	130

Compound **8** (3.0 g, 5.86 mmol), 4-(4,4,5,5-tetramethyl-1,3,2-dioxaborolan-2-yl)aniline (3.86 g, mmol), K_2CO_3 (3.23 g, mmol), and $Pd(PPh_3)_4$ (0.41 mmol) were charged into a 250 mL three-necked flask. The reaction system was degassed and backfilled with nitrogen three times to maintain an inert atmosphere, followed by addition of a pre-degassed mixed solvent of 1,4-dioxane/water (4:1, v/v, 170 mL). The mixture was heated to 110 °C and stirred at this temperature for 72 h. After cooling to room temperature, insoluble residues were removed by filtration, and the organic solvent was evaporated under reduced pressure.

The crude residue was extracted with DCM, and the combined organic phase was washed with saturated aqueous brine, dried over anhydrous Na_2SO_4, and concentrated under vacuum. Purification was carried out by column chromatography on silica gel with CH_2Cl_2/methanol (25:1, v/v) as the eluent. The resulting product was dried under vacuum at 60 °C for 24 h to afford 4.63 g of pale yellow powder, identified as (*R*,*R*)-DACH-NH-Boc (**9**), in 89% yield with >99% ee. ^{1}H NMR (600 MHz, DMSO-D_6) δ 8.14 – 8.13 (d, *J* = 2.0 Hz, 1H), 7.75 – 7.70 (dd, *J* = 8.0, 2.0 Hz, 1H), 7.65 – 7.54 (m, 2H), 7.46 – 7.43 (m, 2H), 7.27 – 7.20 (d, *J* = 8.0 Hz, 1H), 7.12 – 7.06 (d, *J* = 8.3 Hz, 2H), 6.91 – 6.84 (d, *J* = 8.3 Hz, 1H), 6.72 – 6.65 (m, 2H), 6.61 – 6.54 (m, 2H), 6.27 – 6.20 (d, *J* = 8.1 Hz, 1H), 5.41 – 5.33 (m, 2H), 5.25 – 5.15 (s, 2H), 3.15 – 3.09 (d, *J* = 9.9 Hz, 1H), 2.89 – 2.80 (dd, *J* = 8.4, 4.9 Hz, 1H), 1.83 – 1.67 (d, *J* = 11.9 Hz, 1H), 1.52 – 1.43 (q, *J* = 13.4 Hz, 3H), 1.35 – 1.31 (s, 9H), 1.18 – 1.03 (m, 2H); ^{13}C NMR (151 MHz, DMSO-*D*6) δ 24.4, 24.6, 28.8, 32.2, 53.7, 56.9, 78.2, 113.6, 114.8, 125.1, 126.1, 127.4, 127.7, 128.5, 129.3, 129.3, 130.6, 132.0, 132.1, 132.6, 132.9, 133.6, 134.0,

138.6, 139.4, 141.6, 148.5, 149.5, 156.0; DEPT 135° NMR (151 MHz, DMSO-D_6) δ 24.4, 24.6, 28.8, 32.2, 53.7, 56.9, 113.6, 114.8, 125.1, 127.7, 128.5, 129.3, 129.3, 130.6, 132.0, 132.1, 132.6, 132.6, 134.0; ESI-HRMS: Found: m/z 559.2374. Calcd for $C_{29}H_{36}N_4O_4S$: $(M+Na)^+$ 559.2349.

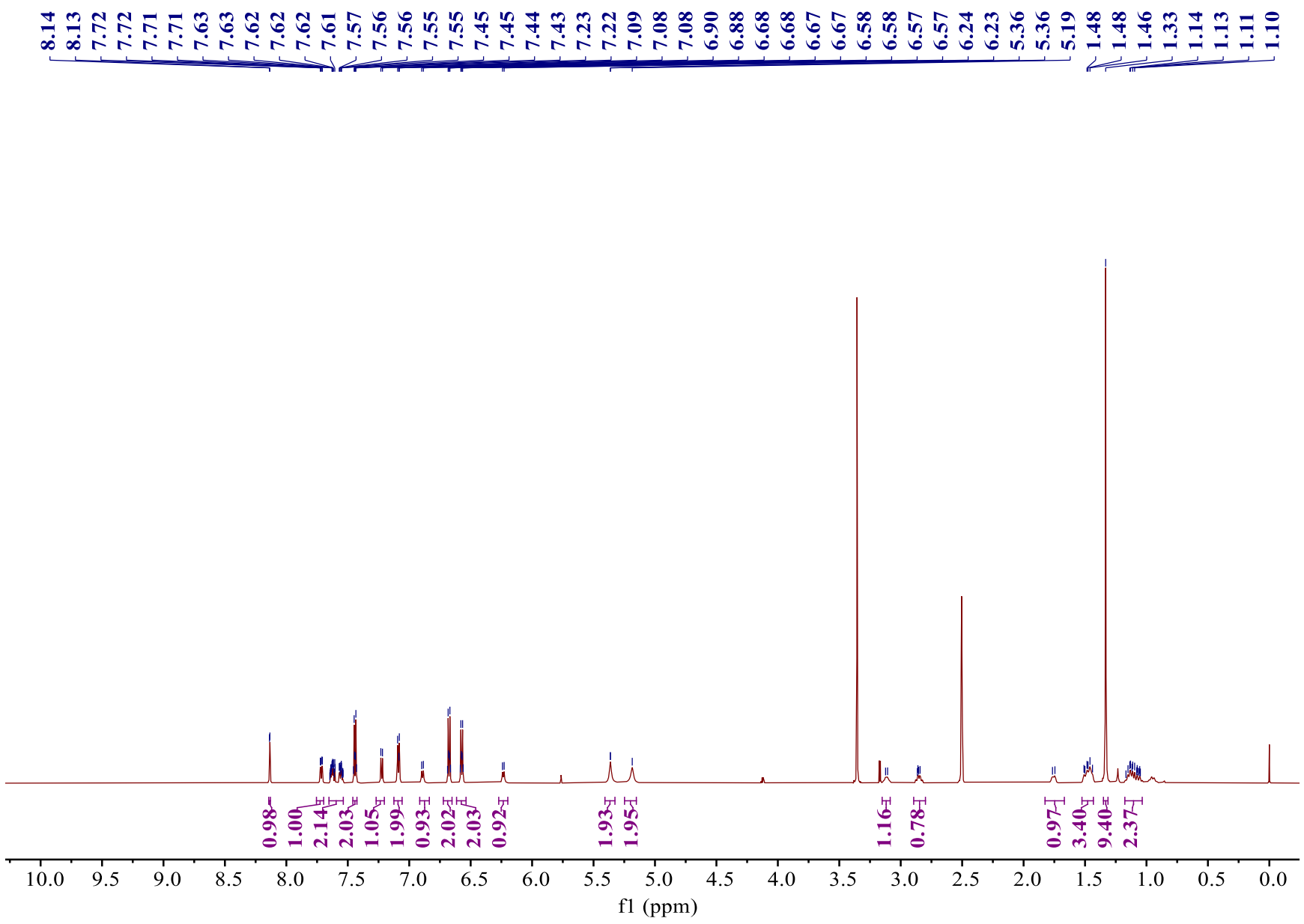

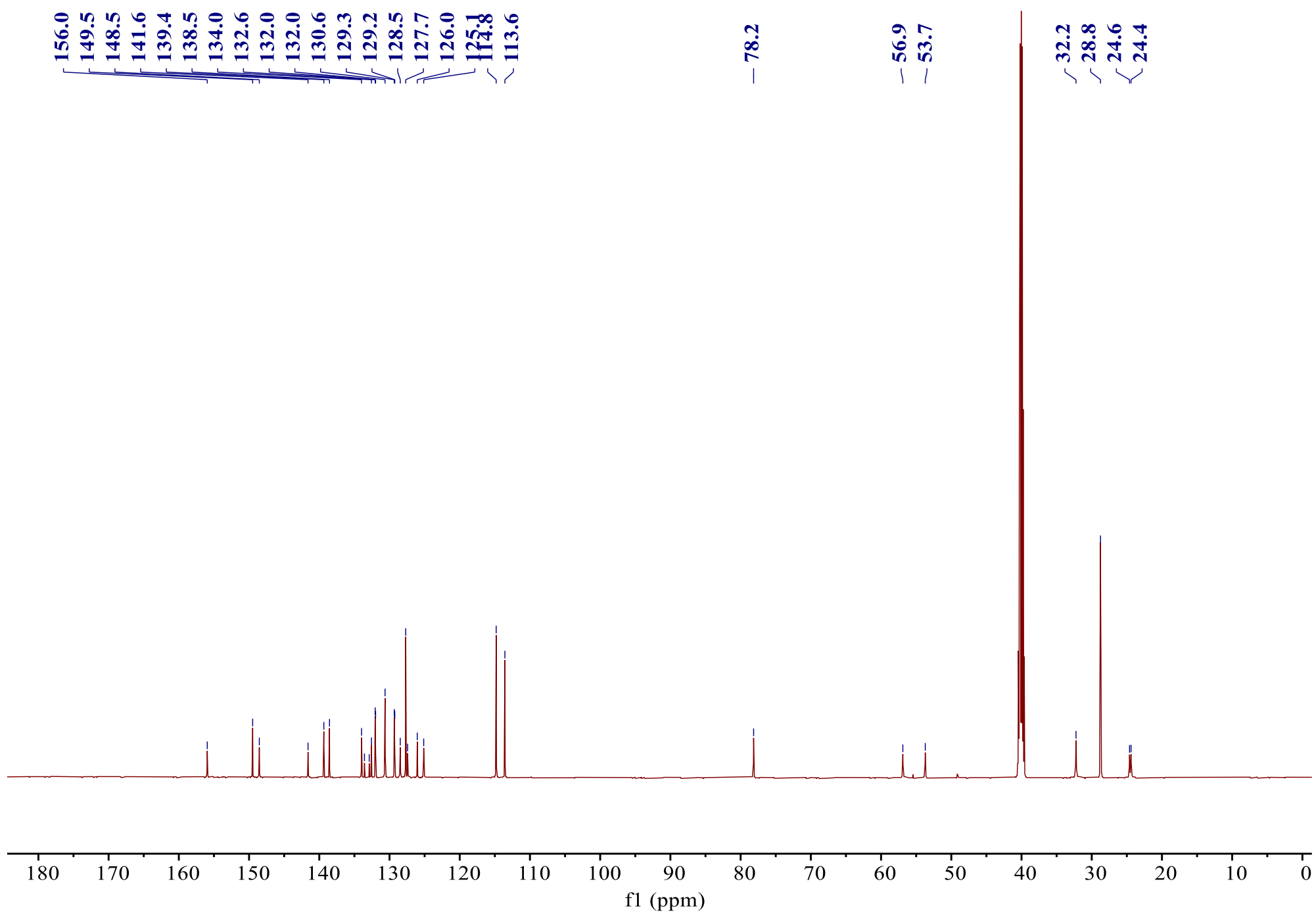

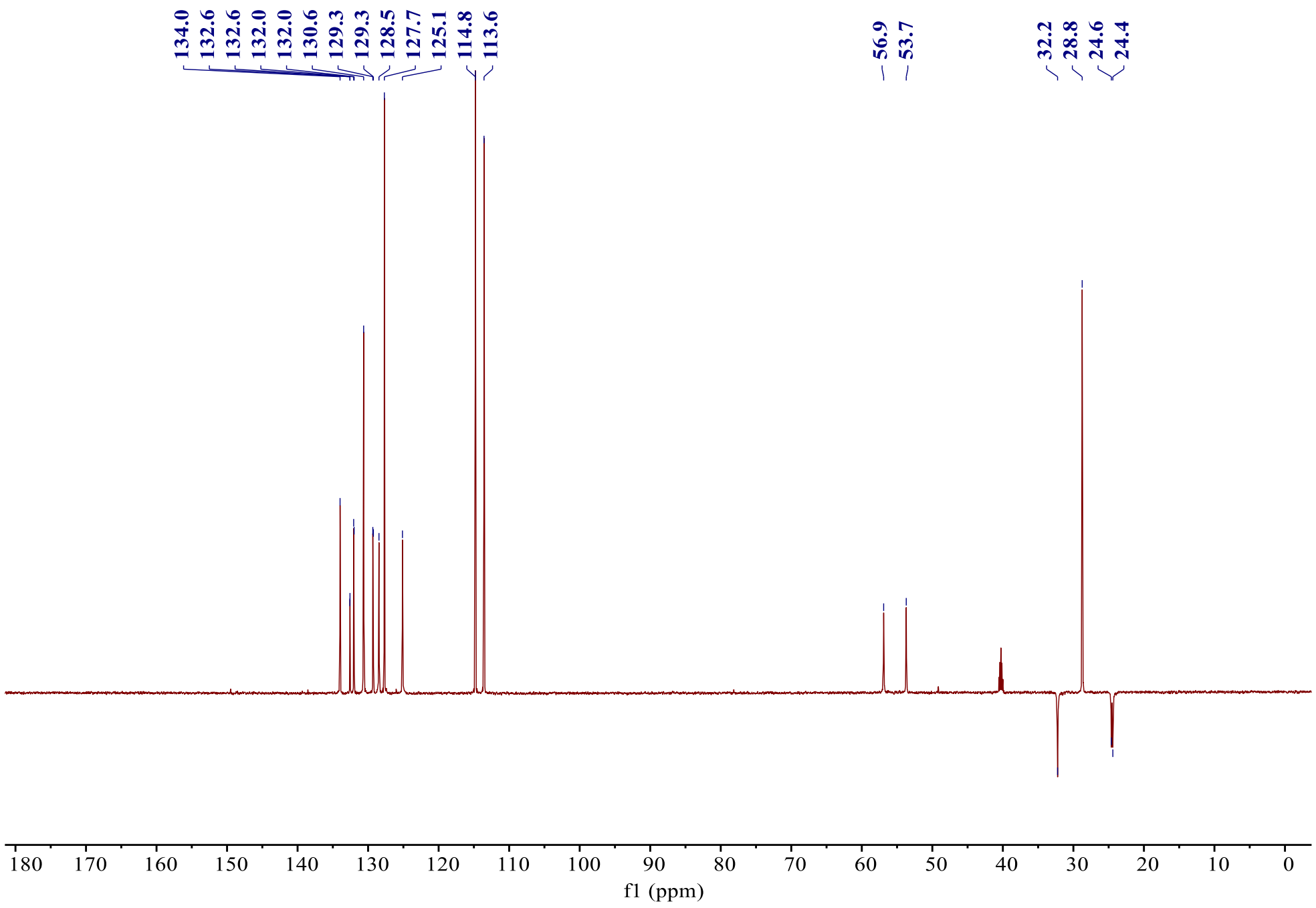

The ee-value of compound **9** was determined by HPLC analysis with a 25 cm × 4.6 mm Daicel Chiralcel IE column (hexane/i-PrOH = 60/40, 1.0 mL/min). t_{minor} = 46.0 min, t_{major} = 117.2 min; ee = 99.4 %. The racemic reference was prepared following the same synthetic procedure, starting from racemic compound **8**, which was derived from (±)-*trans*-1,2-diaminocyclohexane instead of the (1*R*,2*R*) enantiomer.

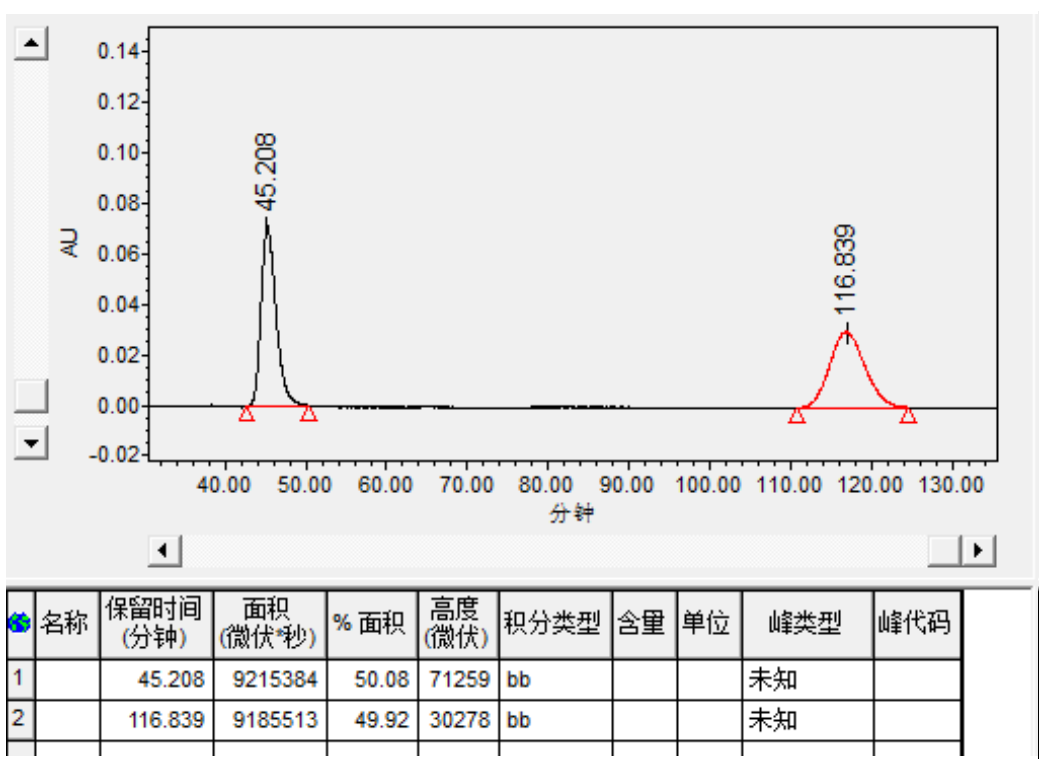

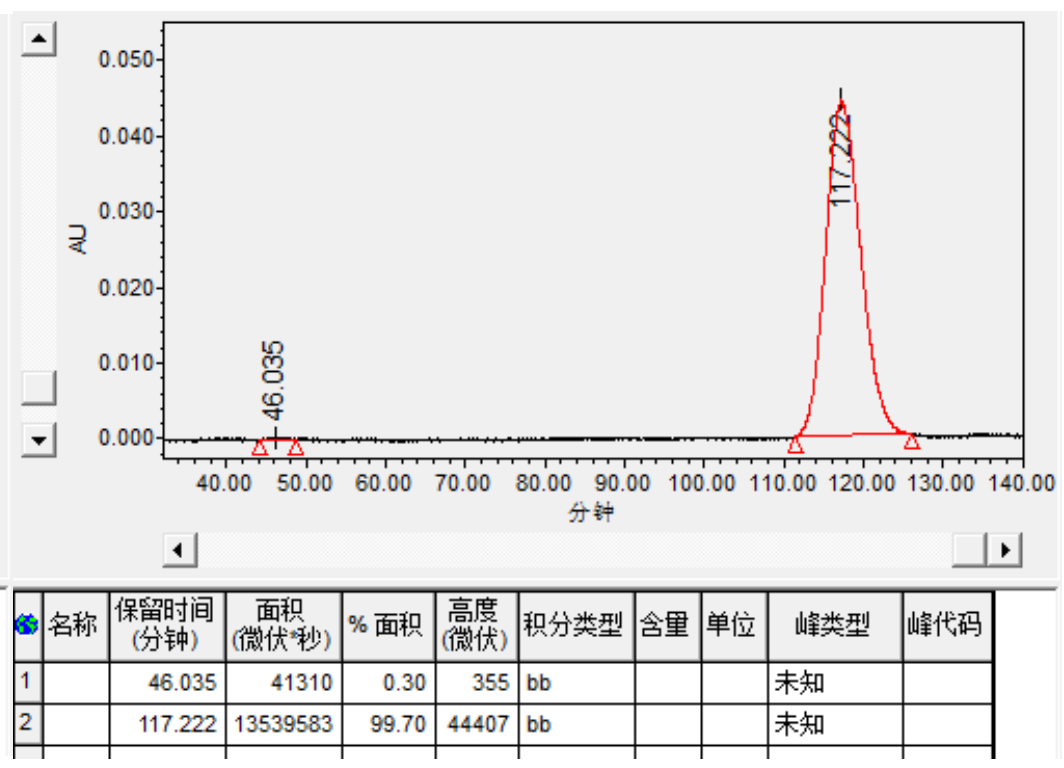

Peaks number	Retention time (min)	Area	Area percentage (%)	Height of peaks
1	46.035	41310	0.3	355
2	117.222	13539583	99.7	44407

Comment 2: With respect to the synthesis of (R)-NH2-Boc, the reaction scheme presented on page 4 is unclear and contains several inaccuracies. In particular, the role

of the R–Ph compound is not explained, and its relevance to the overall study is uncertain. All compounds shown in this scheme should be properly characterized (1H NMR, 13C NMR, DEPT, HRMS, and chiral HPLC where applicable). If these compounds have been previously reported, appropriate references should be clearly cited, and at minimum 1H NMR and chiral HPLC data should be provided. In general, the quality of the 13C NMR spectra throughout the SI is insufficient;

Response: Thank you for your kind reminder. We have corrected the errors in the reaction scheme. The (*R*)–Ph compound was used as a homogeneous model for CCOF2. The (*R*)–Ph–Ru species obtained upon coordination with Ru was employed as a homogeneous model catalyst to facilitate the understanding of the structure of the CCOF2–Ru catalyst. We have remeasured the ^{1}H NMR and ^{13}C NMR spectra to improve their quality and clarity. In addition, chiral HPLC data for all compounds, as well as the previously missing DEPT spectra and HRMS data, have been supplemented.

Manuscript Change:

Supporting Information (revised Reaction scheme and Synthetic Procedures section):

We have revised the synthetic schemes for (R)-NH2-Boc (renamed as (*R*,*R*)-DPEN-NH-Boc) and (*R*)-Ph (renamed as (*R*,*R*)-TP-DPEN) in the SI (Section 2) and replaced the previous NMR spectra with high-resolution ^{1}H, ^{13}C, and DEPT NMR spectra, alongside HRMS and chiral HPLC chromatograms.

Synthetic schemes for (*R*,*R*)-DPEN-NH-Boc and (*R*,*R*)-TP-DPEN (Page 5)

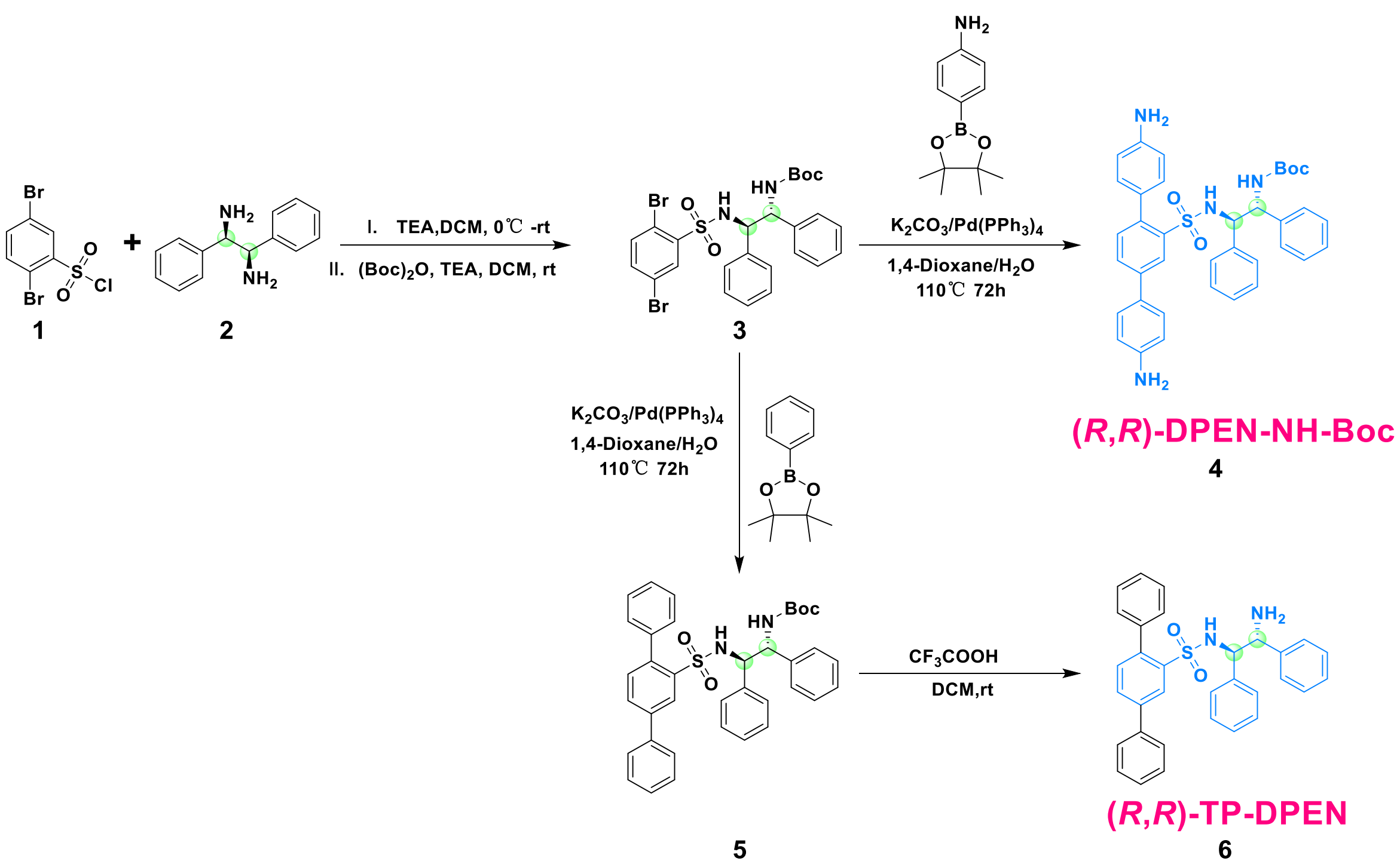

Synthetic schemes for (*R*,*R*)-DACH-NH-Boc (Page 22)

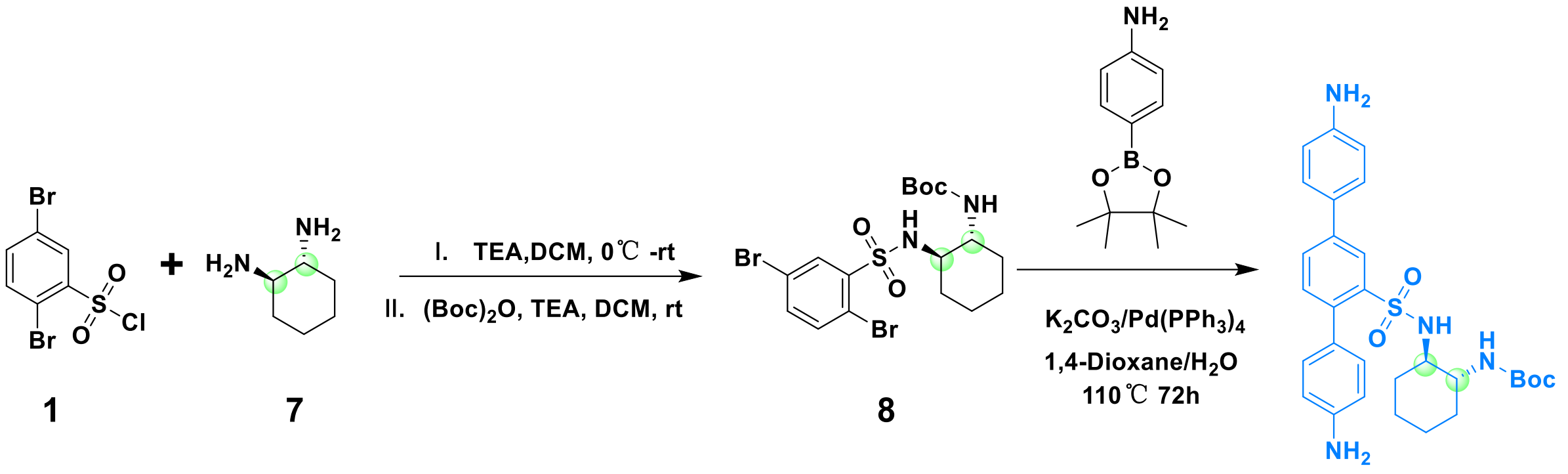

(*R*,*R*)-DACH-NH-Boc

9

Compound 3:

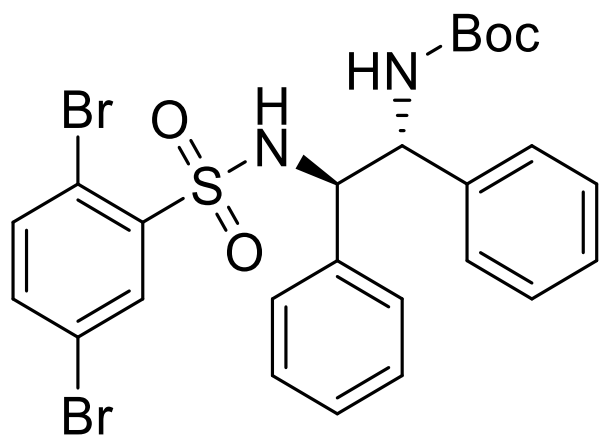

^{1}H NMR (600 MHz, DMSO-*D*$_6$) δ 8.58 – 8.46 (d, *J* = 8.7 Hz, 1H), 7.67 – 7.64 (d, *J* = 2.4 Hz, 1H), 7.49 – 7.39 (m, 3H), 7.25 – 7.19 (dd, *J* = 7.4, 5.0 Hz, 4H), 7.14 – 7.08 (t, *J* = 7.5 Hz, 4H), 7.07 – 7.02 (q, *J* = 7.0 Hz, 2H), 5.02 – 4.85 (dd, *J* = 9.7, 6.3 Hz, 1H), 4.82 – 4.67 (t, *J* = 7.3 Hz, 1H), 1.33 – 1.24 (s, 9H); ^{13}C NMR (151 MHz, DMSO-*D*$_6$) δ 28.7, 59.0, 63.0, 78.6, 118.6, 120.9, 127.3, 127.4, 127.5, 128.1, 128.2, 132.9, 136.3, 137.3, 139.4, 140.5, 141.8, 155.6; DEPT 135° NMR (151 MHz, DMSO-*D*$_6$) δ 28.7, 59.0, 63.0, 127.3, 127.4, 127.5, 128.1, 128.2, 132.9, 136.3, 137.3; ESI-HRMS: Found: m/z 630.9889. Calcd for $C_{25}H_{26}Br_2N_2O_4S$: $(M+Na)^+$ 630.9872.

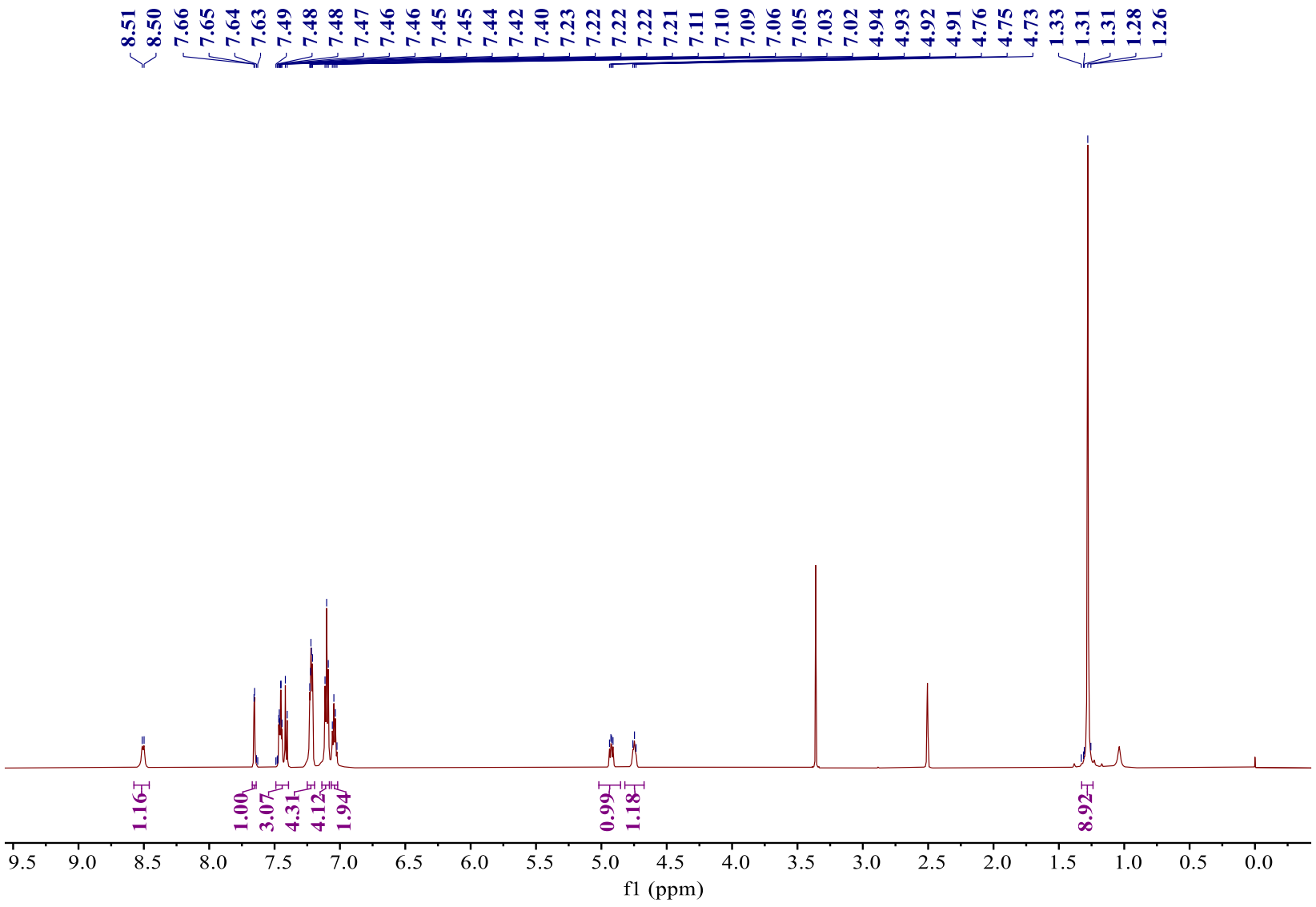
8.51
8.50
7.66
7.65
7.64
7.63
7.49
7.48
7.48
7.47
7.46
7.46
7.45
7.45
7.44
7.42
7.40
7.23
7.22
7.22
7.22
7.21
7.11
7.10
7.09
7.06
7.05
7.03
7.02
4.94
4.93
4.92
4.91
4.76
4.75
4.73
1.33
1.31
1.31
1.28
1.26
1.16
1.00
3.07
4.31
4.12
1.94
0.99
1.18
8.92
f1 (ppm)

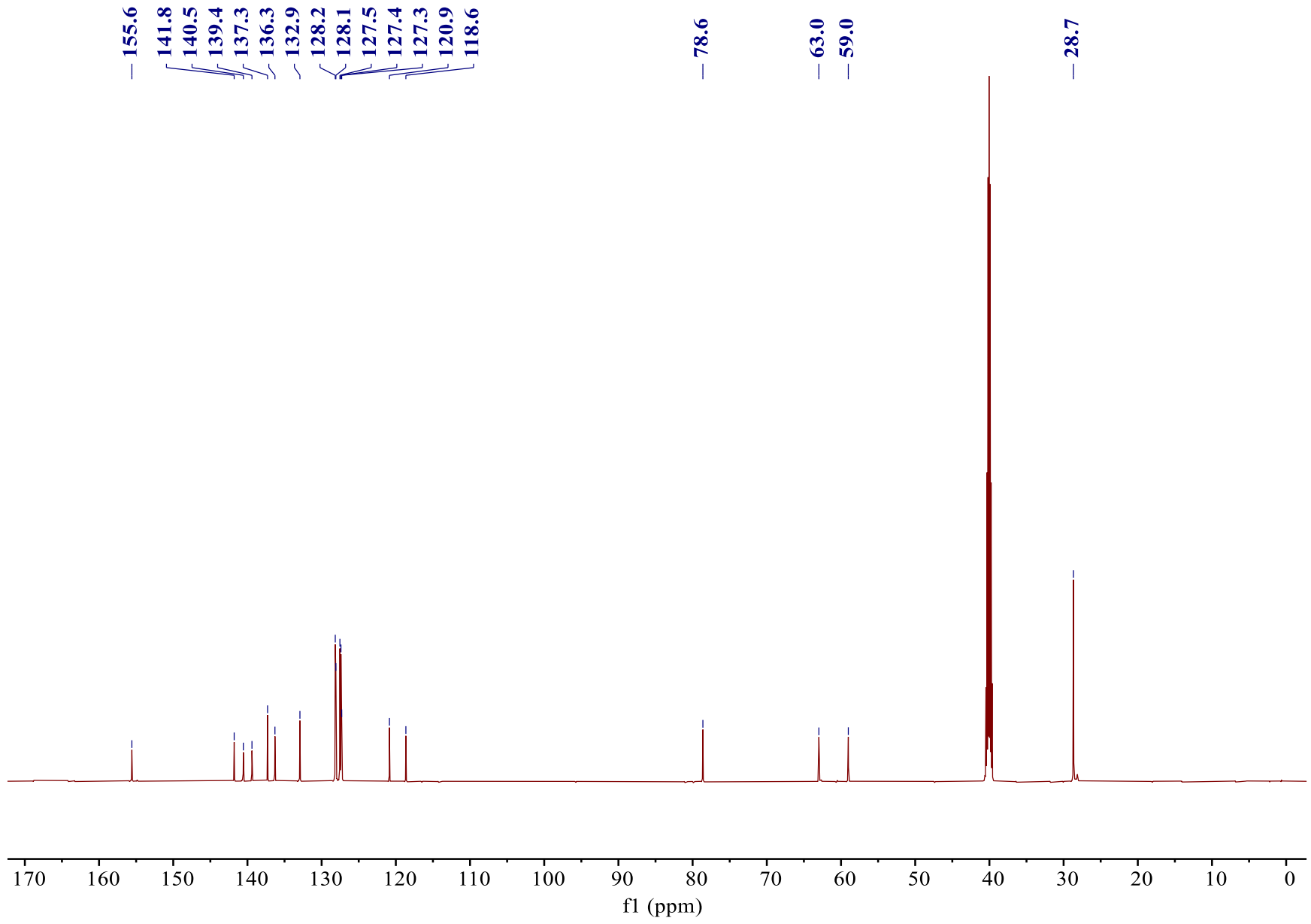
155.6
141.8
140.5
139.4
137.3
136.3
132.9
128.2
128.1
127.5
127.4
127.3
120.9
118.6
78.6
63.0
59.0
28.7
f1 (ppm)

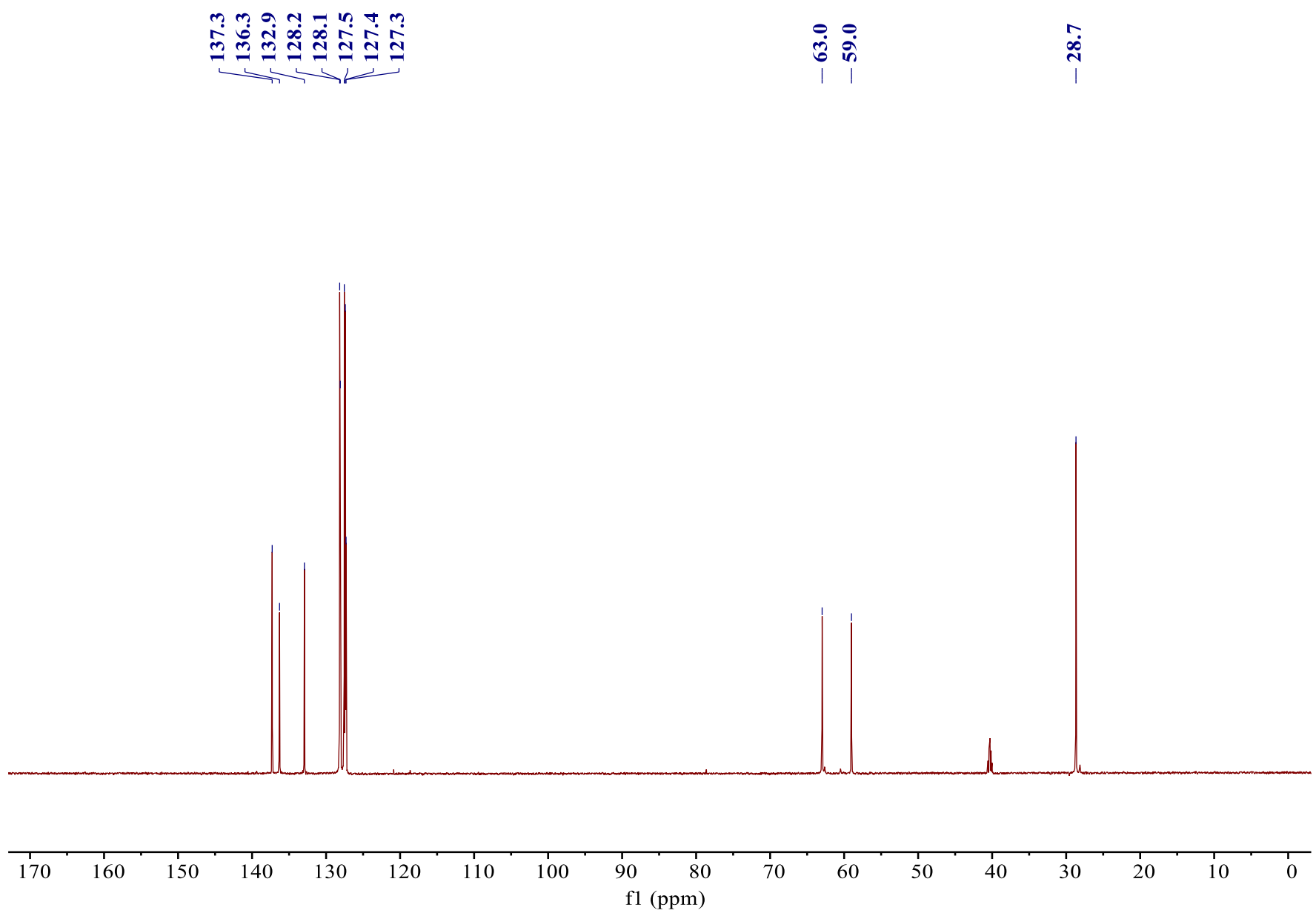
137.3
136.3
132.9
128.2
128.1
127.5
127.4
127.3
63.0
59.0
28.7
f1 (ppm)

The ee-value of compound **3** was determined by HPLC analysis with a 25 cm × 4.6 mm Daicel Chiralcel IC column (hexane/i-PrOH = 90/10, 1.0 mL/min). t_{minor} = 11.1 min, t_{major} = 7.5 min; ee = 99.2 %. The racemic sample used in the HPLC analysis was synthesized by replacing (1*R*,2*R*)-1,2-diphenylethane-1,2-diamine with (±)-1,2-diphenylethane-1,2-diamine under the identical synthetic procedure.

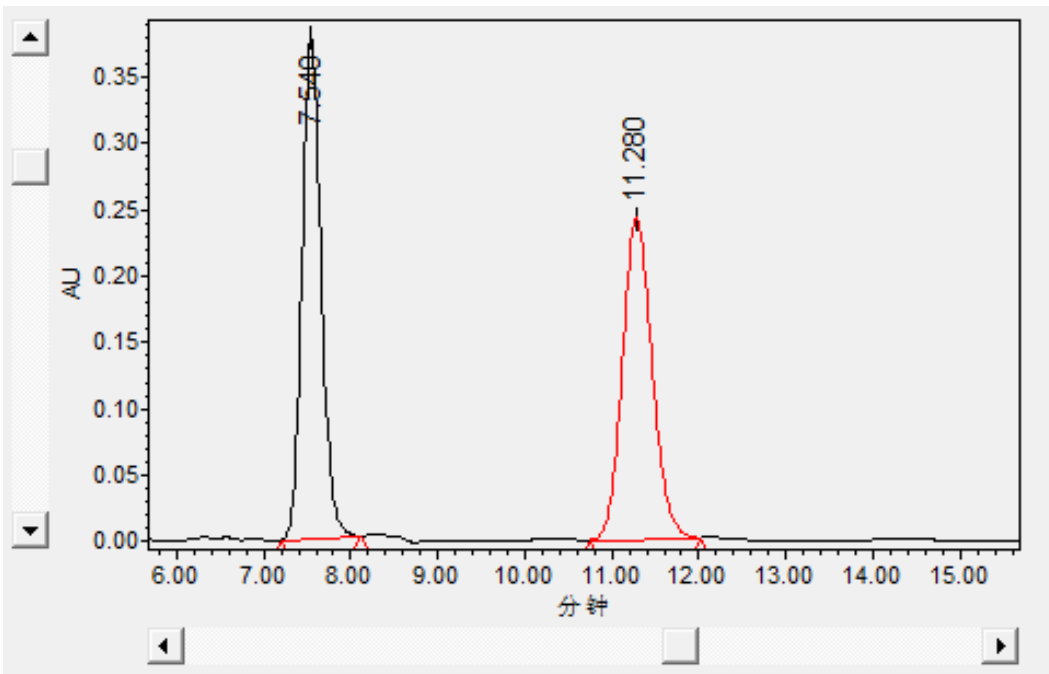

	名称	保留时间 (分钟)	面积 (微伏*秒)	% 面积	高度 (微伏)	积分类型	含量	单位	峰类型	峰代码
1		7.540	5788048	50.34	377355	bb			未知	
2		11.280	5709955	49.66	241982	bb			未知	

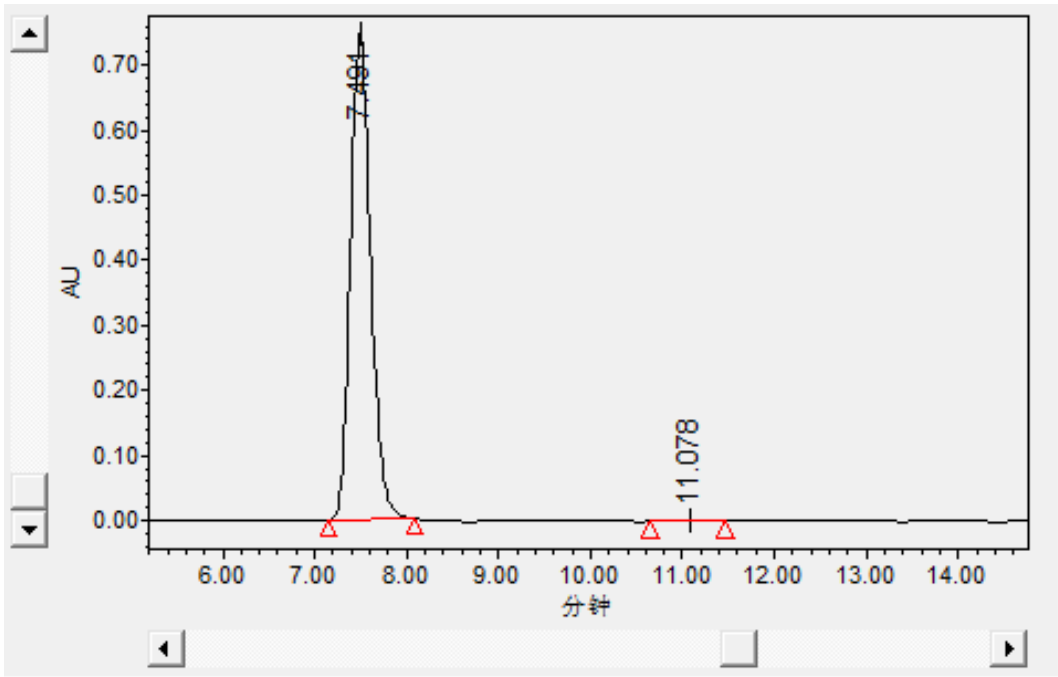

	名称	保留时间 (分钟)	面积 (微伏*秒)	% 面积	高度 (微伏)	积分类型	含量	单位	峰类型	峰代码
1		7.491	11528807	99.63	749239	bb			未知	
2		11.078	42485	0.37	1941	bb			未知	

Peaks number	Retention time (min)	Area	Area percentage (%)	Height of peaks
1	7.491	11528807	99.63	749239
2	11.078	42485	0.37	1941

(*R*,*R*)-DPEN-NH-Boc (Compound **4**):

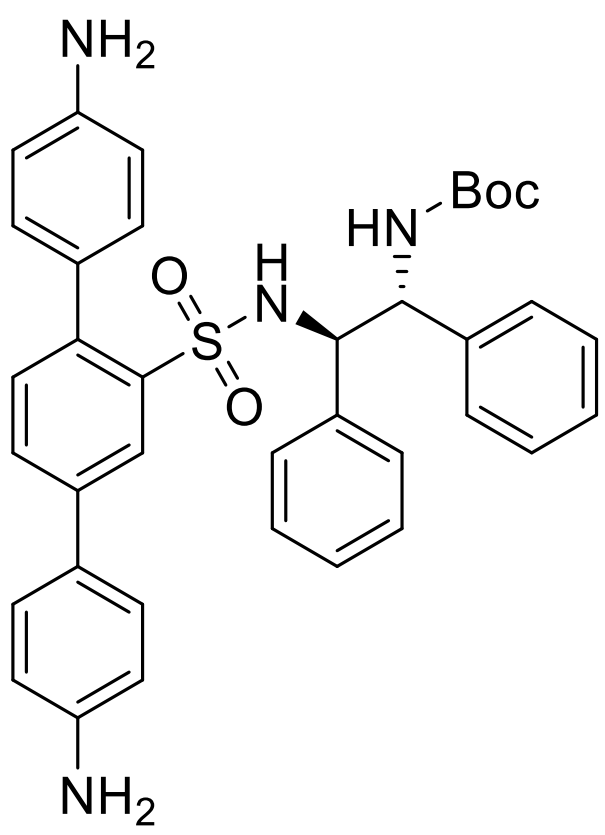

^{1}H NMR (600 MHz, DMSO-D_6) δ 8.02 – 7.91 (d, *J* = 9.6 Hz, 1H), 7.72 – 7.65 (d, *J* = 2.0 Hz, 1H), 7.55 – 7.42 (m, 1H), 7.20 – 7.14 (d, *J* = 9.1 Hz, 1H), 7.13 – 7.10 (dd, *J* = 7.2, 4.9 Hz, 4H), 7.10 – 7.03 (dt, *J* = 14.6, 6.8 Hz, 7H), 7.03 – 7.01 (d, *J* = 7.0 Hz, 1H), 7.01 – 6.99 (d, *J* = 7.8 Hz, 1H), 6.93 – 6.87 (d, *J* = 8.1 Hz, 2H), 6.63 – 6.59 (d, *J* = 8.0 Hz, 2H), 6.57 – 6.53 (d, *J* = 8.3 Hz, 2H), 5.39 – 5.10 (d, 4H), 4.85 – 4.79 (t, *J* = 8.3 Hz, 1H), 4.71 – 4.53 (t, *J* = 8.5 Hz, 1H), 1.34 – 1.26 (s, 9H); ^{13}C NMR (151 MHz,

DMSO-D_6) δ 28.7, 60.0, 62.5, 78.7, 113.4, 114.7, 125.1, 126.1, 127.2, 127.4, 127.5, 127.6, 127.7, 127.9, 128.2, 128.2, 128.3, 130.6, 133.7, 138.4, 139.2, 139.6, 140.4, 140.8, 148.3, 149.3, 155.5; DEPT 135° NMR (151 MHz, DMSO-D_6) δ 28.7, 60.0, 62.5, 113.4, 114.7, 125.1, 127.2, 127.4, 127.6, 127.7, 127.9, 128.2, 128.2, 128.3, 130.6, 133.7; ESI-HRMS: Found: m/z 657.2537. Calcd for $C_{25}H_{26}Br_2N_2O_4S$: $(M+Na)^+$ 657.2506.

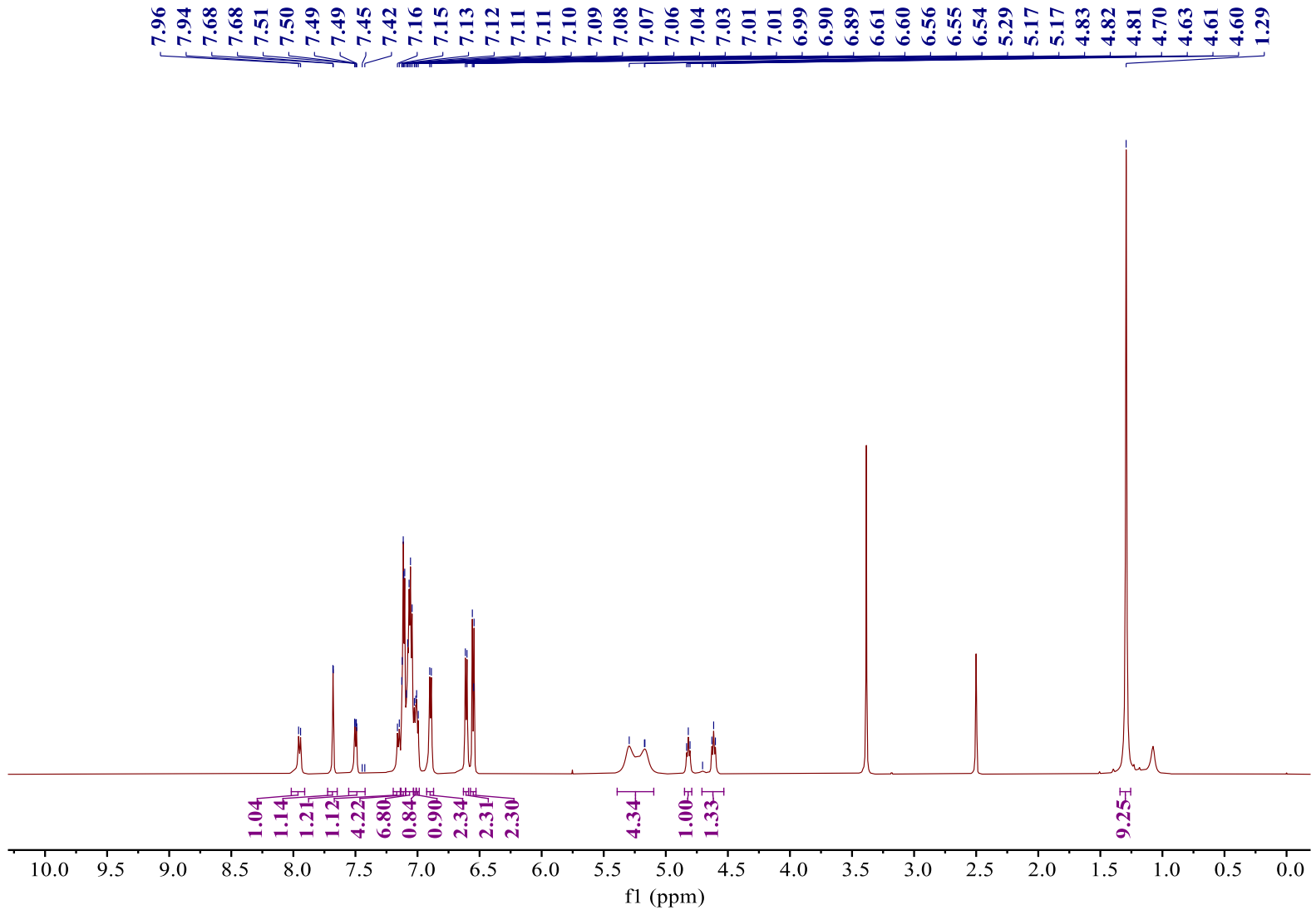

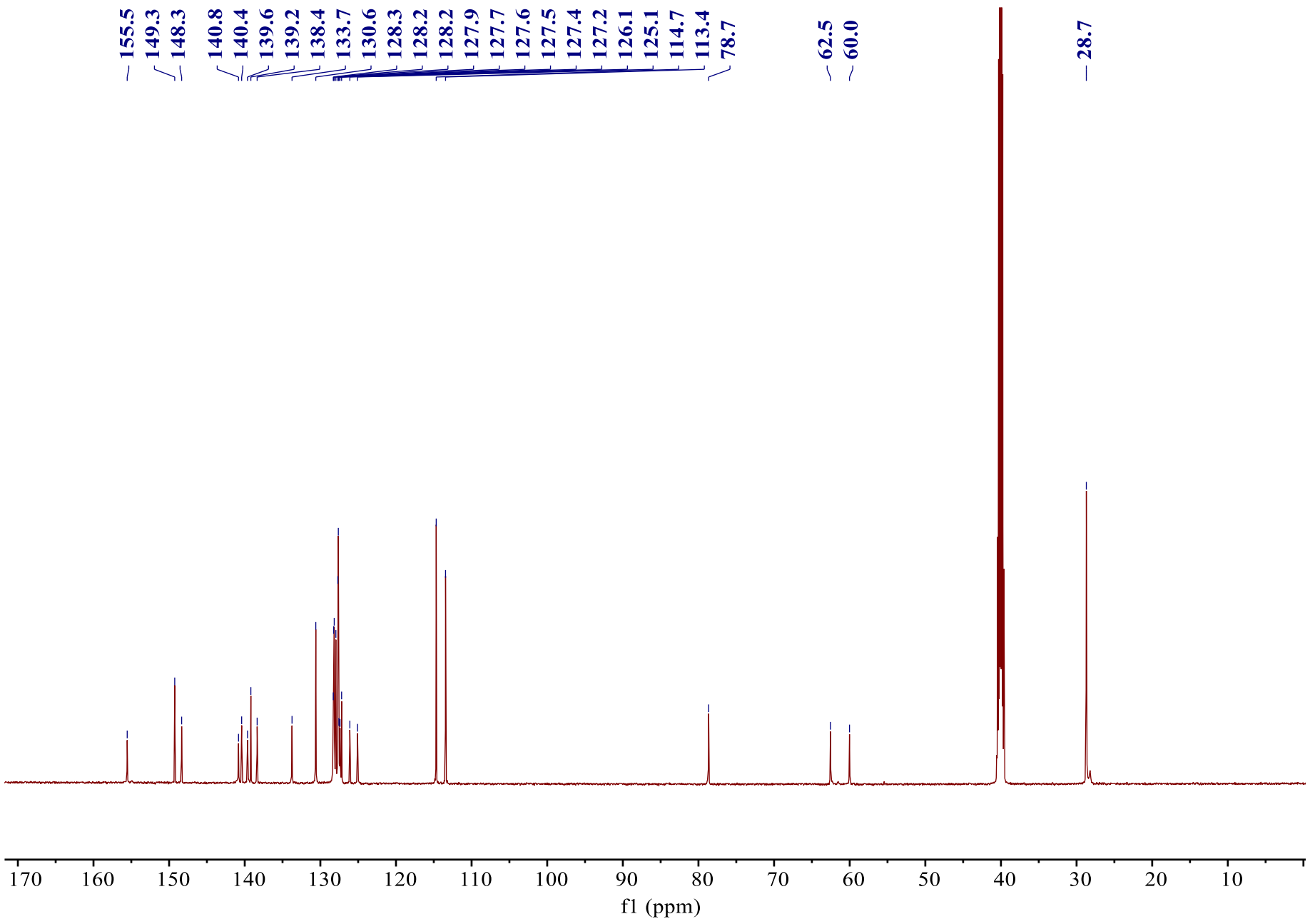

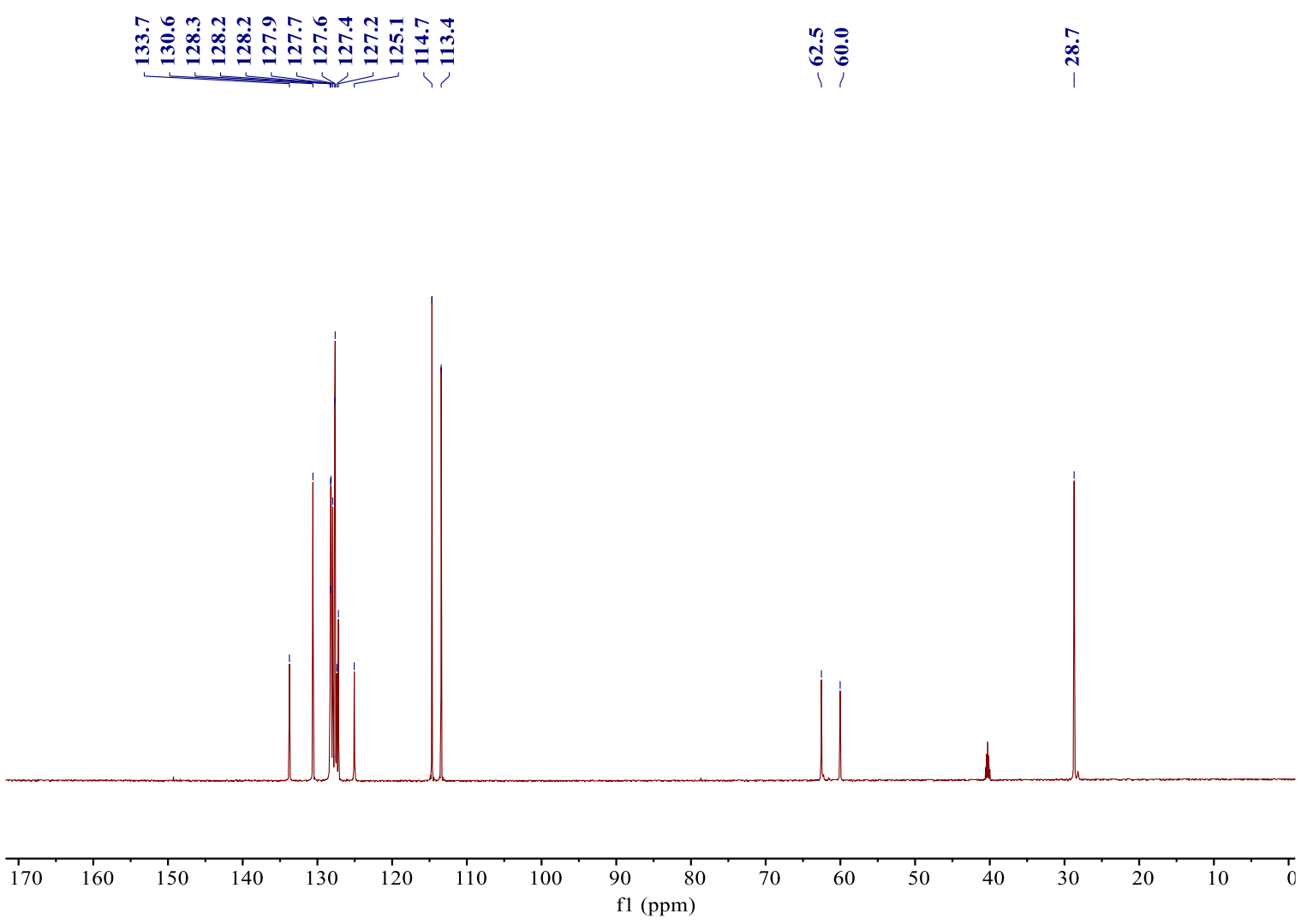

The ee-value of ***(R,R)*-DPEN-NH-Boc** was determined by HPLC analysis with a 25 cm × 4.6 mm Daicel Chiralcel IE column (hexane/i-PrOH = 60/40, 1.0 mL/min). t_{minor} = 33.7 min, t_{major} = 25.3 min; ee = 99.1%. The racemic reference was prepared following the same synthetic procedure, starting from racemic compound **3**, which was derived from (±)-1,2-diphenylethane-1,2-diamine instead of the (1*R*,2*R*) enantiomer.

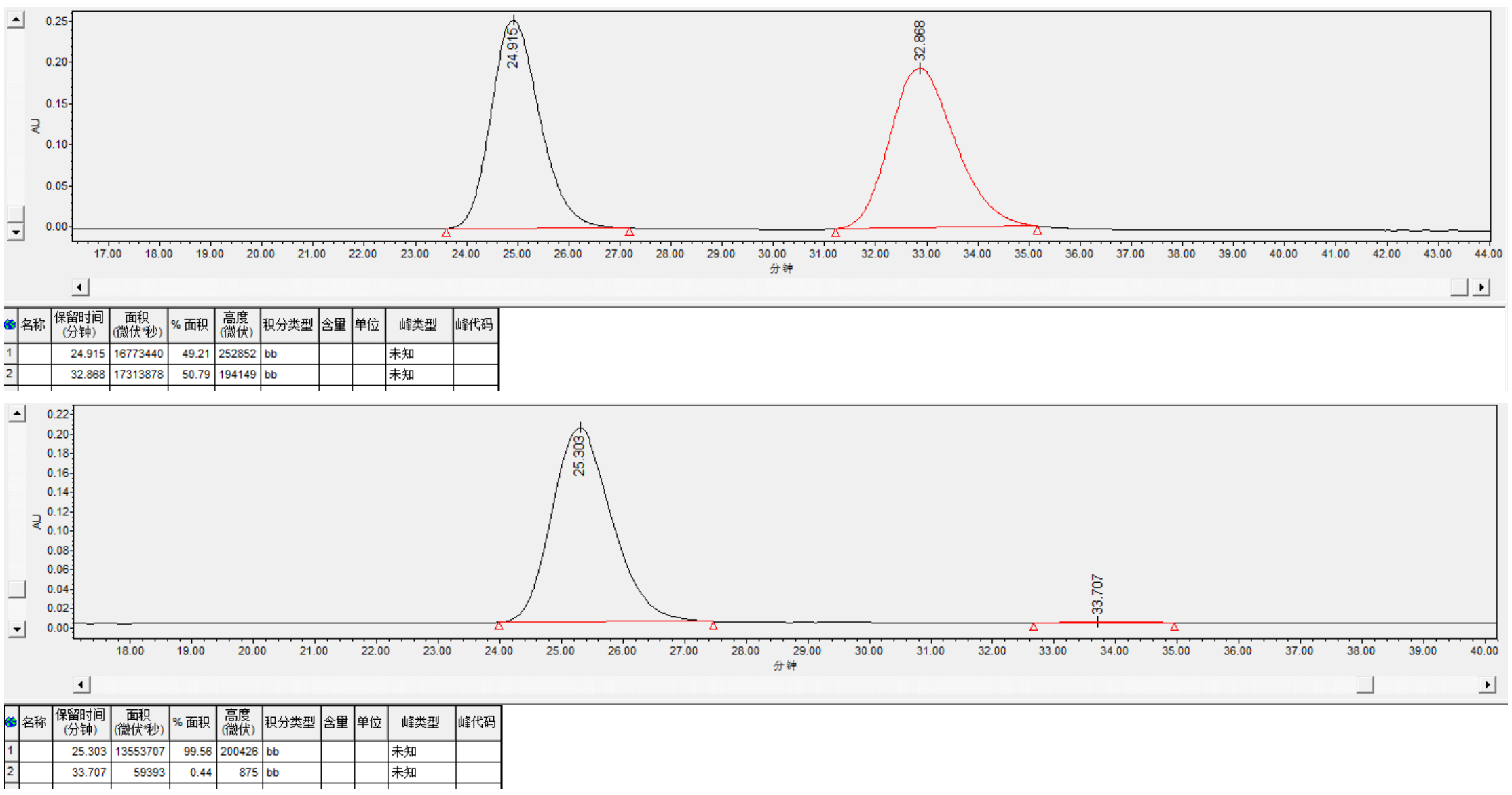

Peaks number	Retention time (min)	Area	Area percentage (%)	Height of peaks
1	25.303	13553707	99.56	200426
2	33.707	59393	0.44	875

Compound 5:

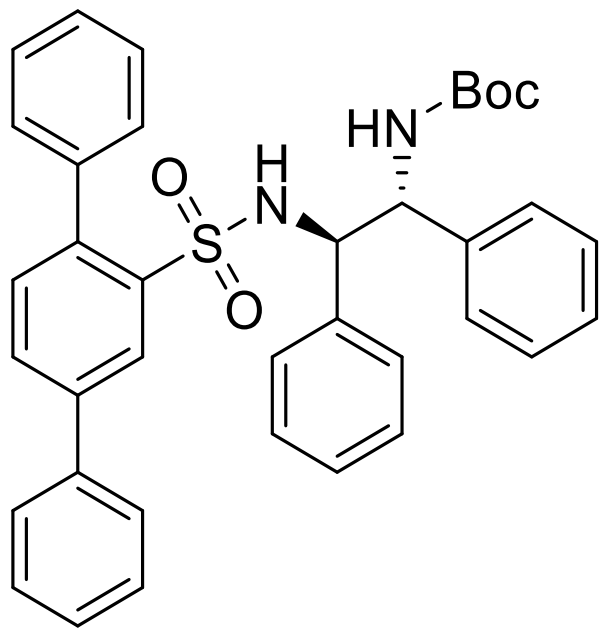

^{1}H NMR (600 MHz, CHLOROFORM-*D*) δ 8.17 – 8.10 (s, 1H), 7.72 – 7.63 (m, 1H), 7.55 – 7.50 (d, *J* = 7.6 Hz, 2H), 7.49 – 7.44 (t, *J* = 7.5 Hz, 2H), 7.43 – 7.37 (m, 2H), 7.36 – 7.29 (t, *J* = 7.7 Hz, 4H), 7.21 – 7.17 (d, *J* = 7.8 Hz, 1H), 7.13 – 7.05 (m, 4H), 7.04 – 6.98 (t, *J* = 7.5 Hz, 2H), 6.95 – 6.89 (m, 2H), 5.50 – 5.42 (d, *J* = 8.1 Hz, 1H), 5.01 – 4.94 (d, *J* = 7.6 Hz, 1H), 4.85 – 4.77 (t, *J* = 9.1 Hz, 1H), 4.43 – 4.35 (t, *J* = 8.9 Hz, 1H), 1.46 – 1.35 (s, 9H); ^{13}C NMR (151 MHz, CHLOROFORM-*D*) δ 28.4, 59.4, 63.7, 80.3, 127.2, 127.3, 127.5, 127.7, 127.9, 128.1, 128.1, 128.2, 128.4, 128.5, 129.0, 129.8, 130.1, 133.1, 137.4, 138.4, 138.7, 139.0, 139.4, 139.7, 140.3, 156.4; DEPT 135° NMR (151 MHz, CHLOROFORM-D) δ 28.4, 59.4, 63.7, 127.2, 127.3, 127.5, 127.7, 127.9, 128.1, 128.2, 128.2, 128.4, 128.5, 129.1, 129.8, 130.2, 133.1; ESI-HRMS: Found: m/z 627.2310. Calcd for $C_{37}H_{36}N_2O_4S$: $(M+Na)^+$ 627.2288.

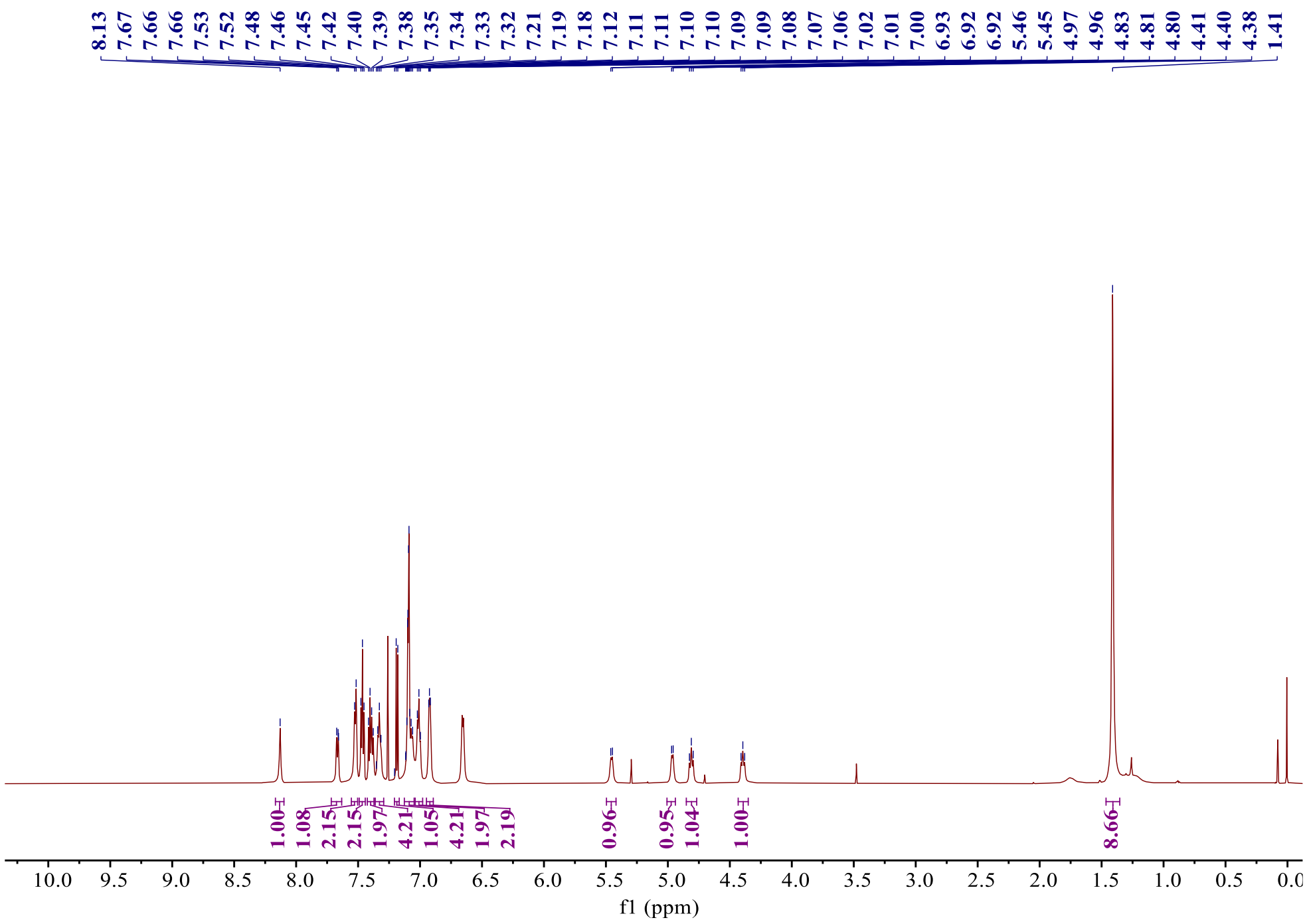

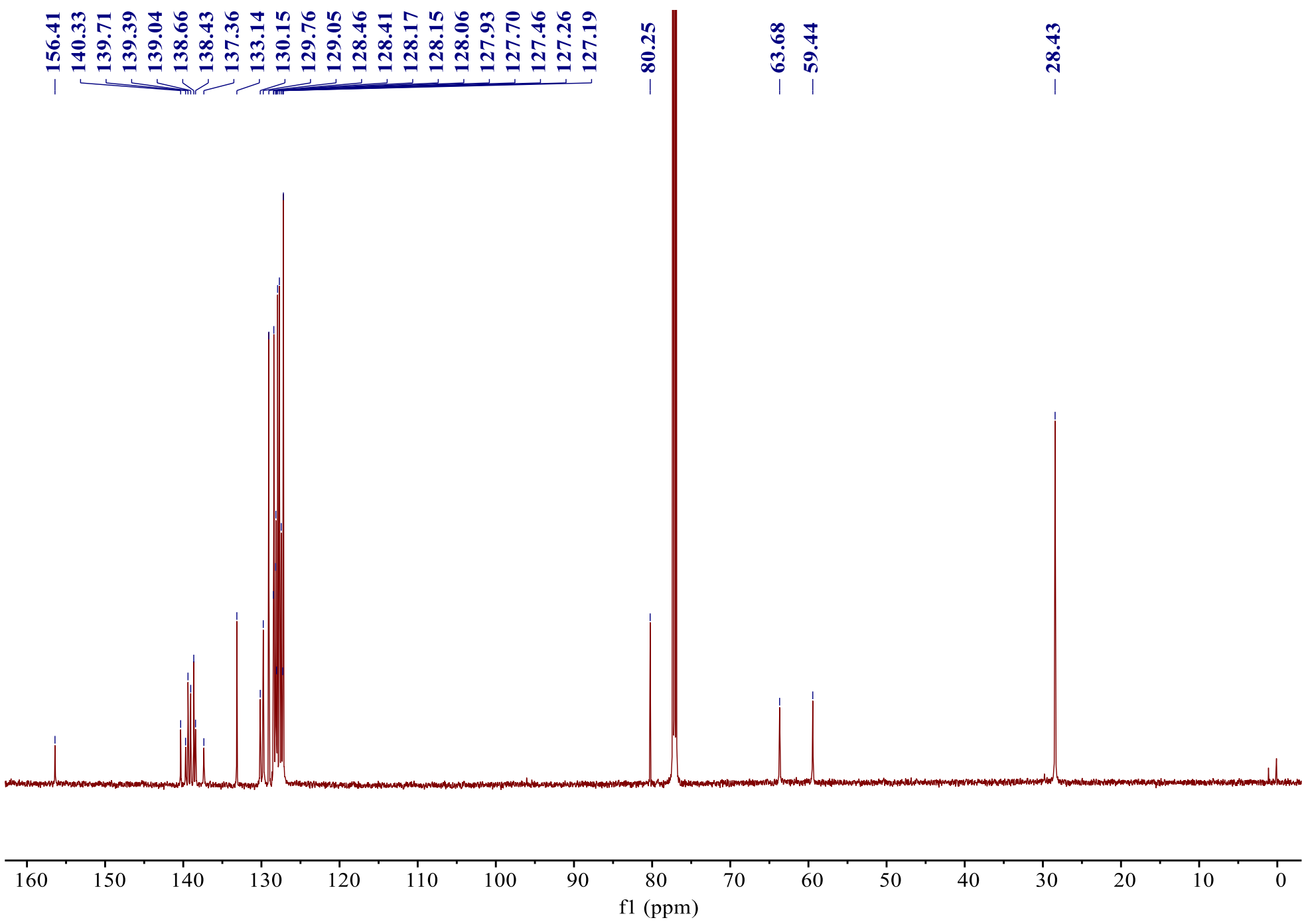

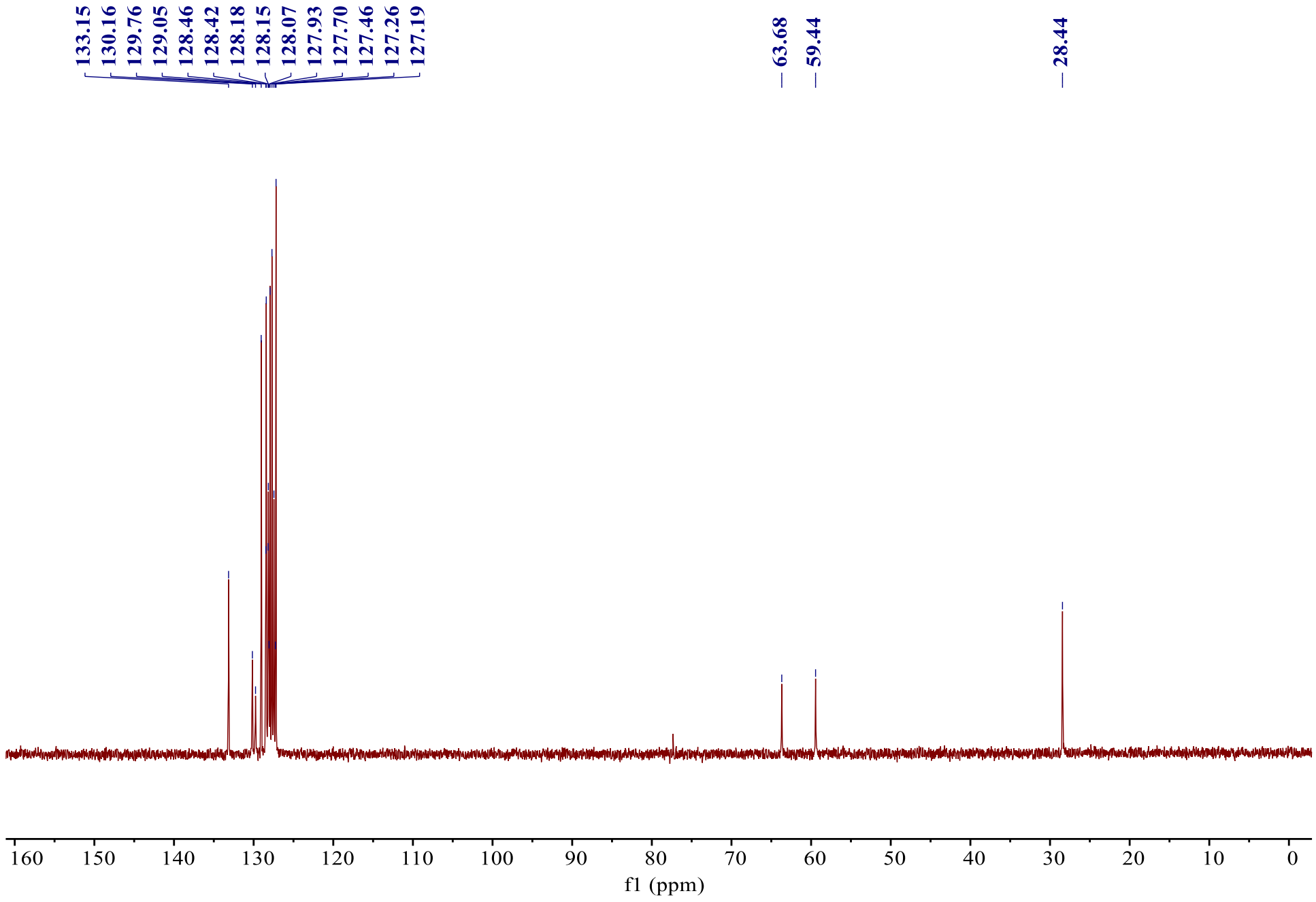

The ee-value of **compound 5** was determined by HPLC analysis with a 25 cm × 4.6 mm Daicel Chiralcel IA column (hexane/i-PrOH = 90/10, 1.0 mL/min). t_{minor} = 15.3 min, t_{major} = 8.2 min; ee = 99.4%. The racemic reference was prepared following the same synthetic procedure, starting from racemic compound **3**, which was derived from (±)-1,2-diphenylethane-1,2-diamine instead of the (1*R*,2*R*) enantiomer.

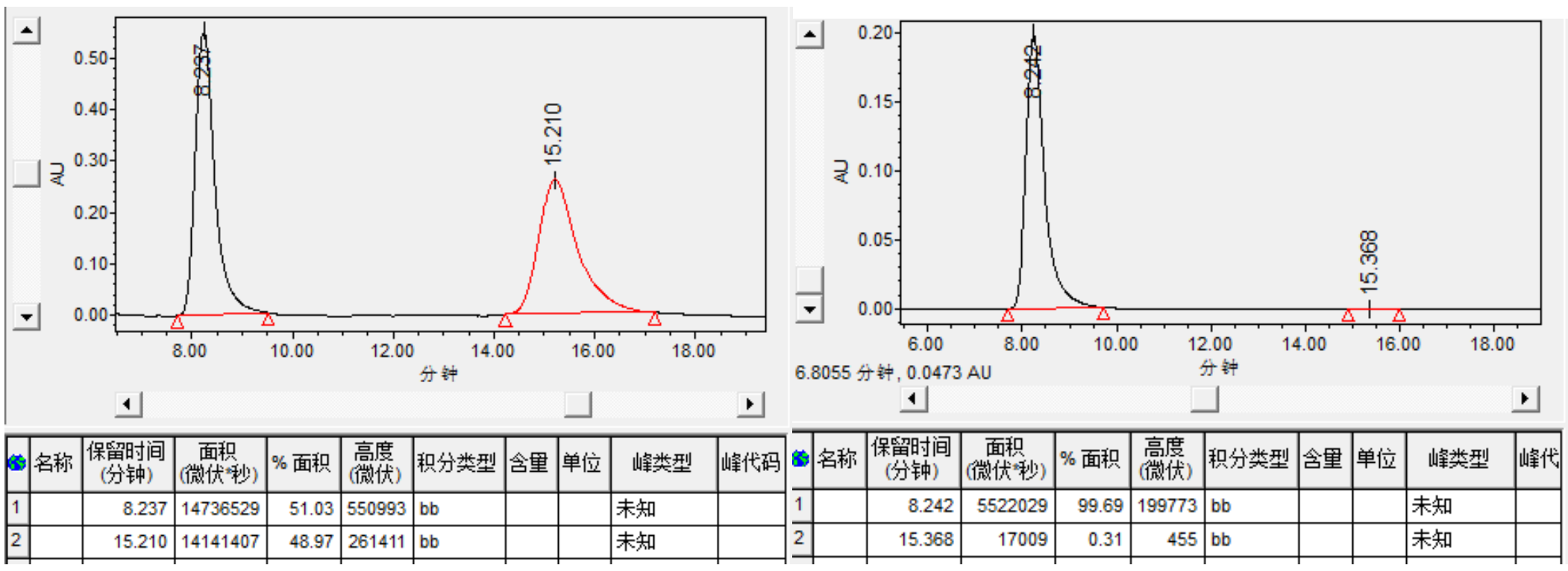

Peaks number	Retention time (min)	Area	Area percentage (%)	Height of peaks
1	8.242	5522029	99.69	199773
2	15.368	17009	0.31	455

(*R*,*R*)-TP-DPEN (Compound 6):

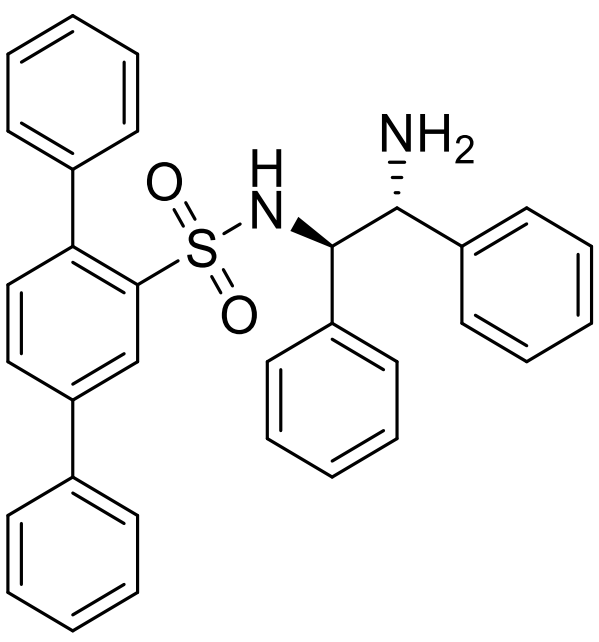

^{1}H NMR (600 MHz, DMSO-D_6) δ 7.91 – 7.88 (d, J = 2.0 Hz, 1H), 7.74 – 7.71 (dd, J = 7.9, 2.0 Hz, 1H), 7.47 – 7.43 (ddd, J = 8.8, 6.4, 0.9 Hz, 2H), 7.42 – 7.36 (m, 6H), 7.34 – 7.30 (m, 2H), 7.22 – 7.20 (d, J = 7.9 Hz, 1H), 7.08 – 6.96 (m, 10H), 4.39 – 4.31 (d, J = 8.2 Hz, 1H), 4.03 – 3.96 (d, J = 8.2 Hz, 1H); ^{13}C NMR (151 MHz, DMSO-D_6) δ 61.3, 65.4, 126.5, 127.2, 127.3, 127.8, 128.0, 128.1, 128.5, 129.5, 129.8, 129.8, 133.7, 138.7, 139.5, 139.7, 140.0, 140.1, 141.0, 143.1; DEPT 135° NMR (151 MHz, DMSO-D_6) δ 61.3, 65.4, 126.5, 127.2, 127.3, 127.8, 128.0, 128.1, 128.5, 129.5, 129.8, 129.8, 133.7; ESI-HRMS: Found: m/z 527.1783. Calcd for $C_{32}H_{28}N_2O_2S$: $(M+Na)^+$ 527.1764.

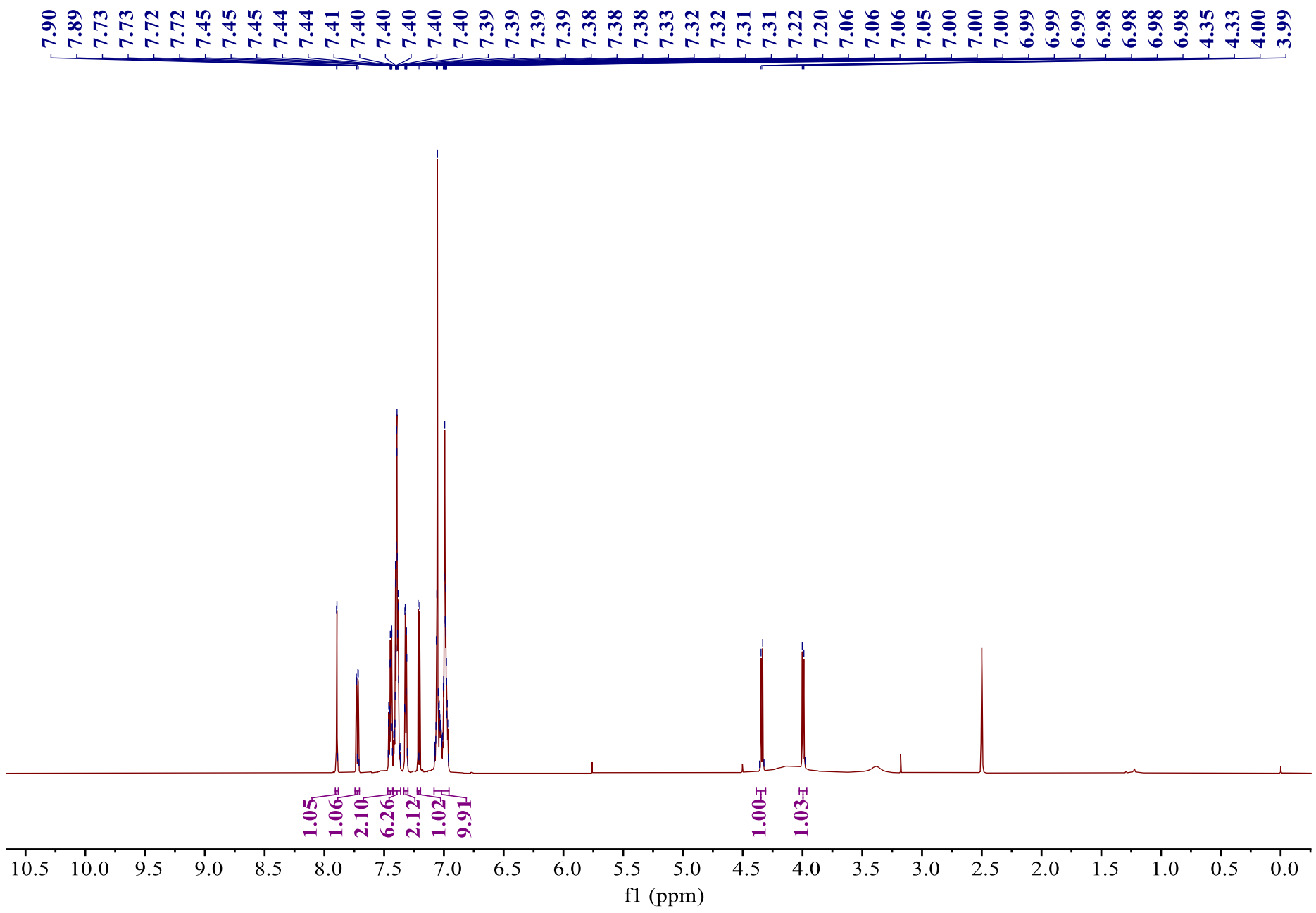
7.90
7.89
7.73
7.73
7.72
7.72
7.45
7.45
7.45
7.44
7.44
7.41
7.40
7.40
7.40
7.40
7.40
7.39
7.39
7.39
7.39
7.38
7.38
7.38
7.33
7.32
7.32
7.31
7.31
7.22
7.20
7.06
7.06
7.06
7.05
7.00
7.00
7.00
6.99
6.99
6.99
6.98
6.98
6.98
6.98
4.35
4.33
4.00
3.99
1.05
1.06
2.10
6.26
2.12
1.02
9.91
1.00
1.03
10.5
10.0
9.5
9.0
8.5
8.0
7.5
7.0
6.5
6.0
5.5
5.0
4.5
4.0
3.5
3.0
2.5
2.0
1.5
1.0
0.5
0.0
f1 (ppm)

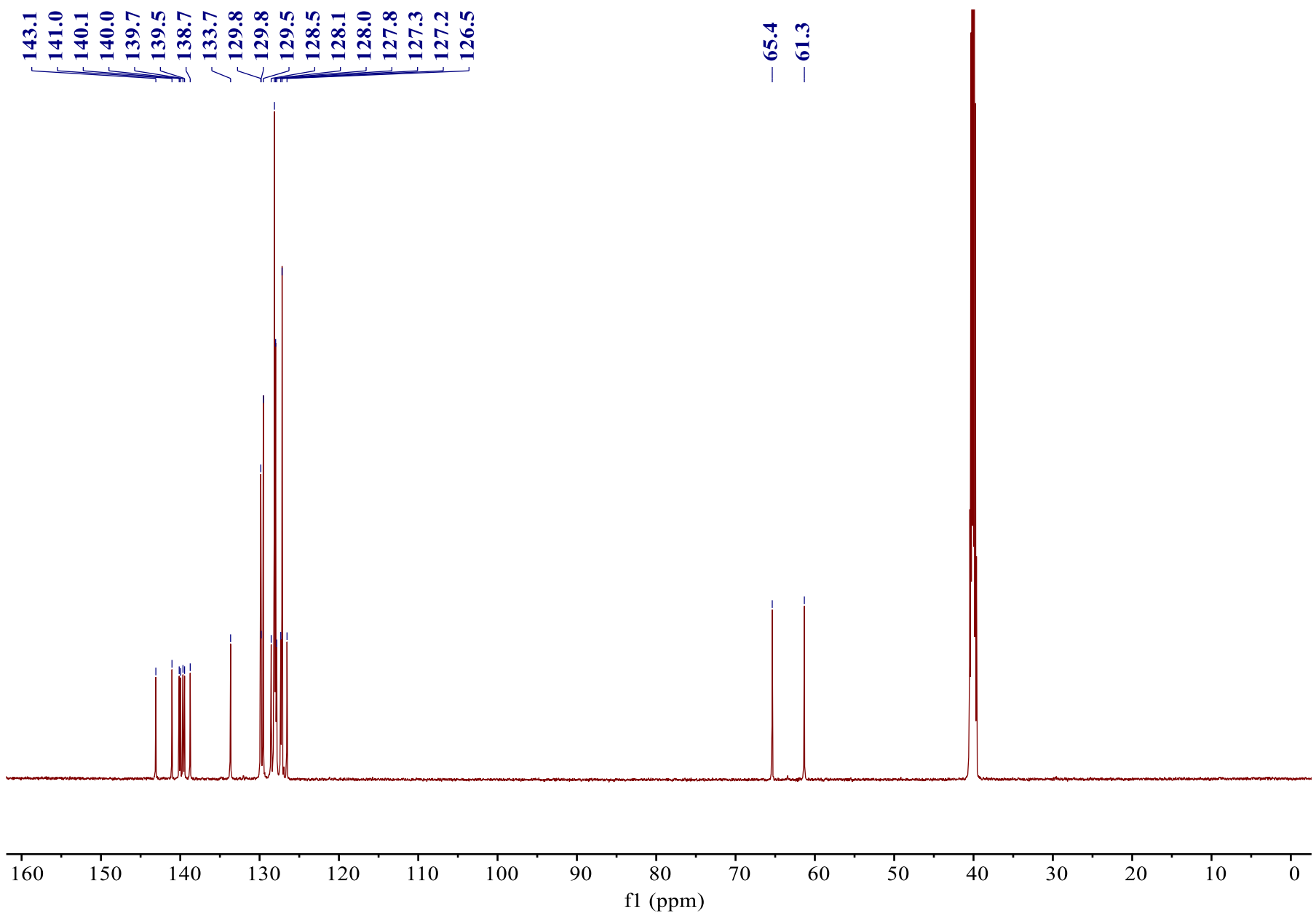
143.1
141.0
140.1
140.0
139.7
139.5
138.7
133.7
129.8
129.8
129.5
128.5
128.1
128.0
127.8
127.3
127.2
126.5
65.4
61.3
160
150
140
130
120
110
100
90
80
70
60
50
40
30
20
10
0
f1 (ppm)

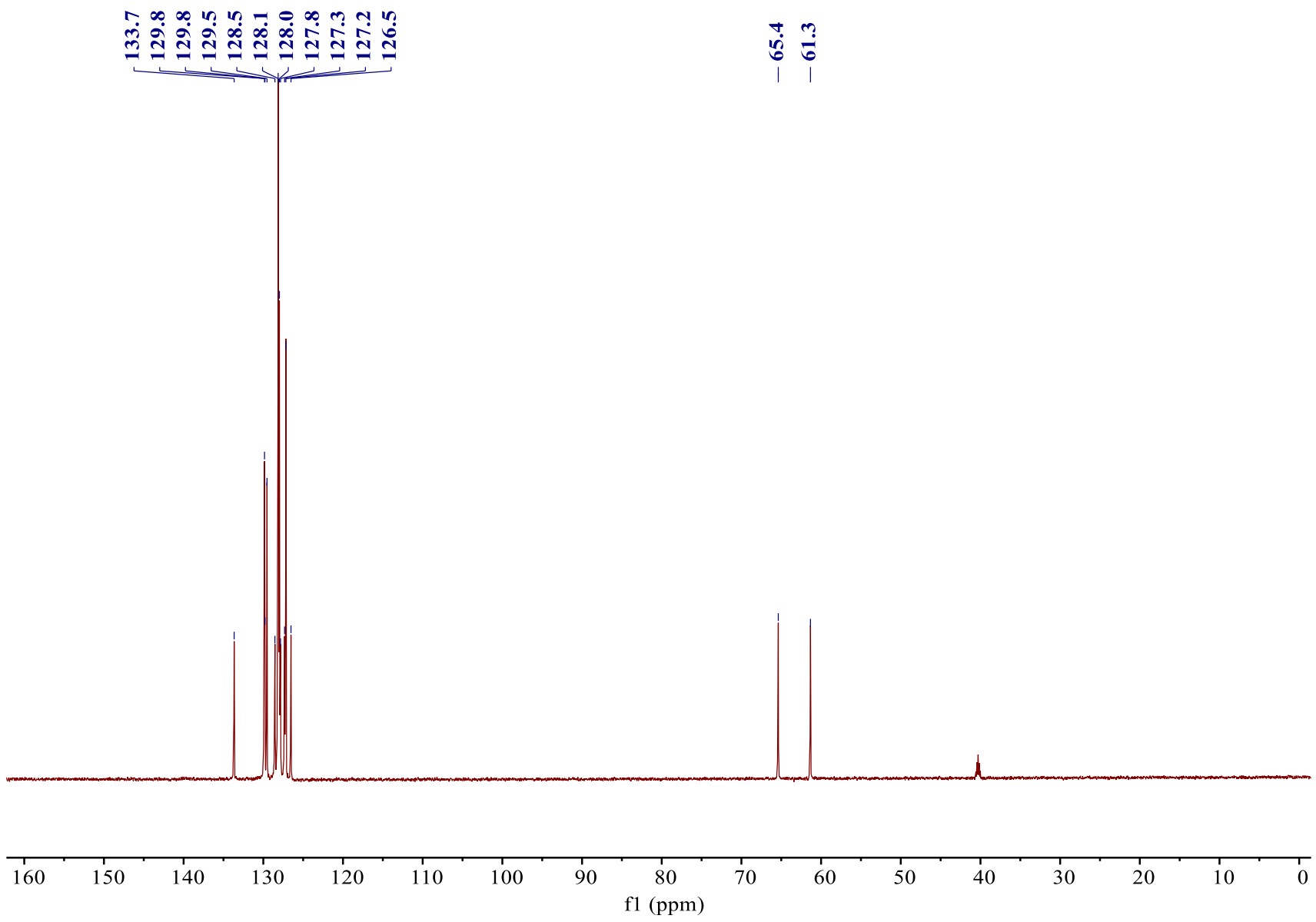

The ee-value of **(*R*,*R*)-TP-DPEN (6)** was determined by HPLC analysis with a 25 cm × 4.6 mm Daicel Chiralcel IE column (hexane/i-PrOH = 70/30, 1.0 mL/min). t_{minor} = 15.3 min, t_{major} = 8.2 min; ee = 99.9%. The racemic reference was prepared following the same synthetic procedure, starting from racemic compound **5**, which was derived from (±)-1,2-diphenylethane-1,2-diamine instead of the (1*R*,2*R*) enantiomer.

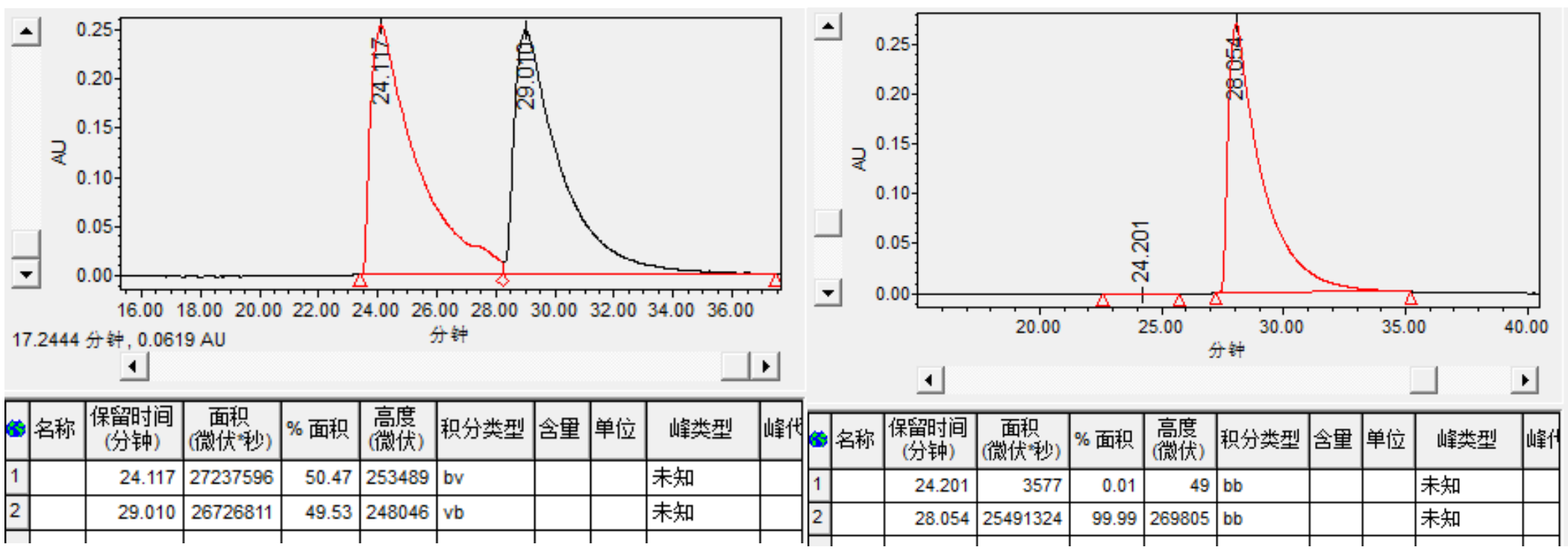

Peaks number	Retention time (min)	Area	Area percentage (%)	Height of peaks
1	24.201	3577	0.01	49
2	28.054	25491327	99.99	259805

Compound 8:

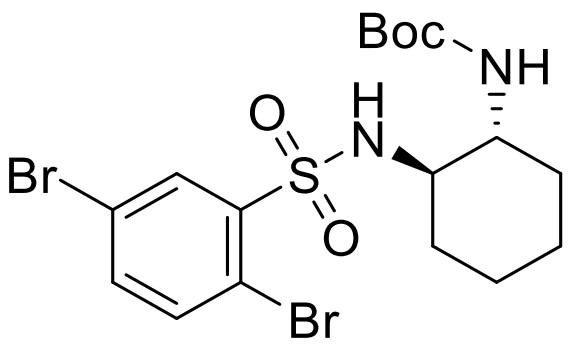

^{1}H NMR (600 MHz, DMSO-D_6) δ 8.05 – 8.03 (d, J = 2.4 Hz, 1H), 7.86 – 7.83 (d, J = 8.8 Hz, 1H), 7.71 – 7.65 (m, 2H), 6.10 – 6.03 (d, J = 8.9 Hz, 1H), 3.21 – 3.13 (qd, J = 10.3, 4.0 Hz, 1H), 2.99 – 2.90 (dtd, J = 14.5, 9.6, 4.2 Hz, 1H), 1.70 – 1.65 (m, 1H), 1.61 – 1.56 (m, 1H), 1.52 – 1.46 (m, 2H), 1.33 – 1.30 (s, 9H), 1.30 – 1.24 (dd, J = 12.5, 2.9 Hz, 1H), 1.17 – 0.94 (m, 2H); ^{13}C NMR (151 MHz, DMSO-D_6) δ 24.6, 24.8, 28.8, 32.6, 33.4, 53.4, 56.8, 78.0, 119.1, 121.1, 133.2, 136.6, 137.5, 143.1, 155.8; DEPT 135° NMR (151 MHz, DMSO-D_6) δ 24.6, 24.8, 28.8, 32.6, 33.4, 53.4, 56.8, 133.2, 136.5, 137.5; ESI-HRMS: Found: m/z 532.9726. Calcd for $C_{17}H_{24}Br_2N_2O_4S$: $(M+Na)^+$ 532.9713.

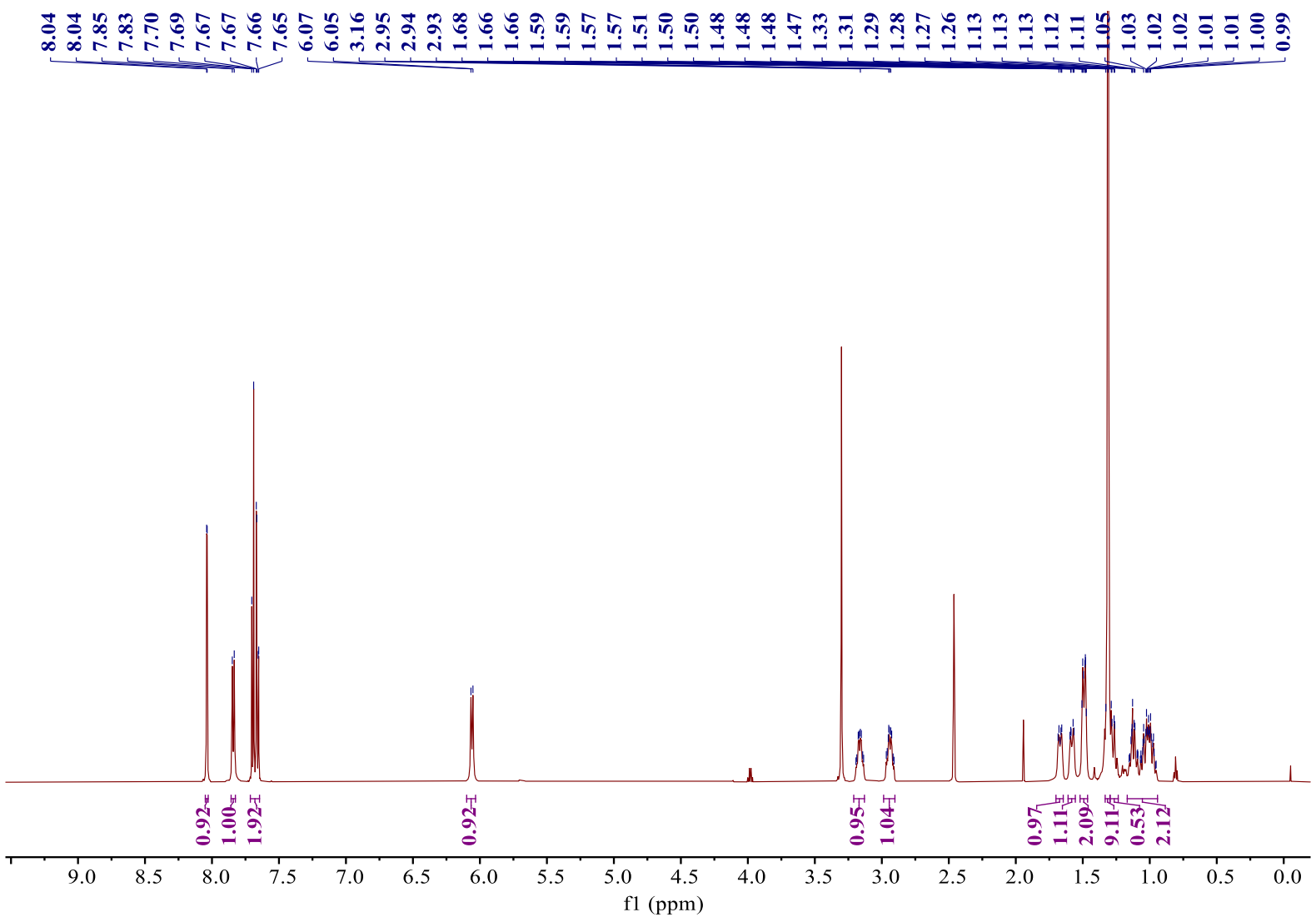

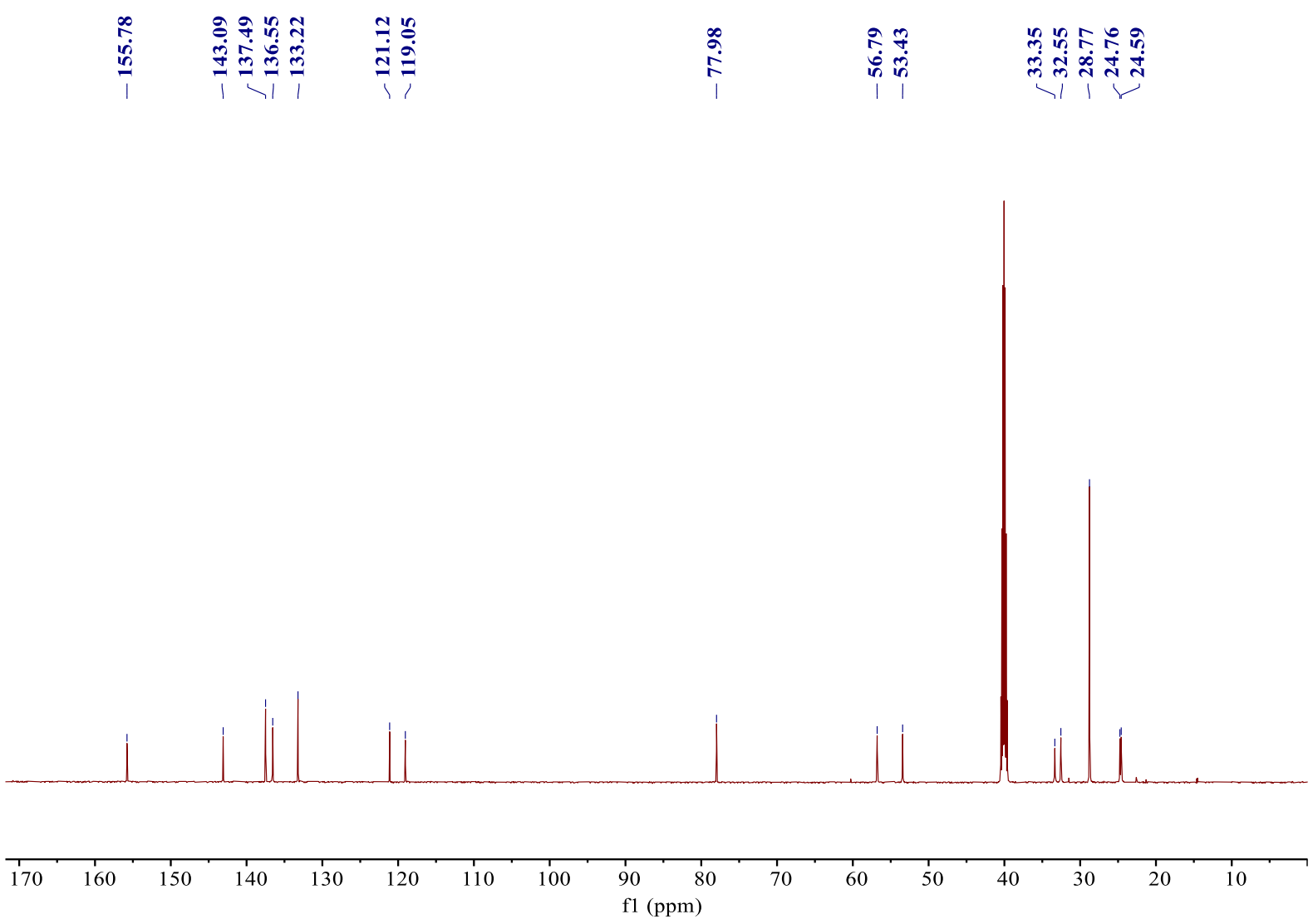

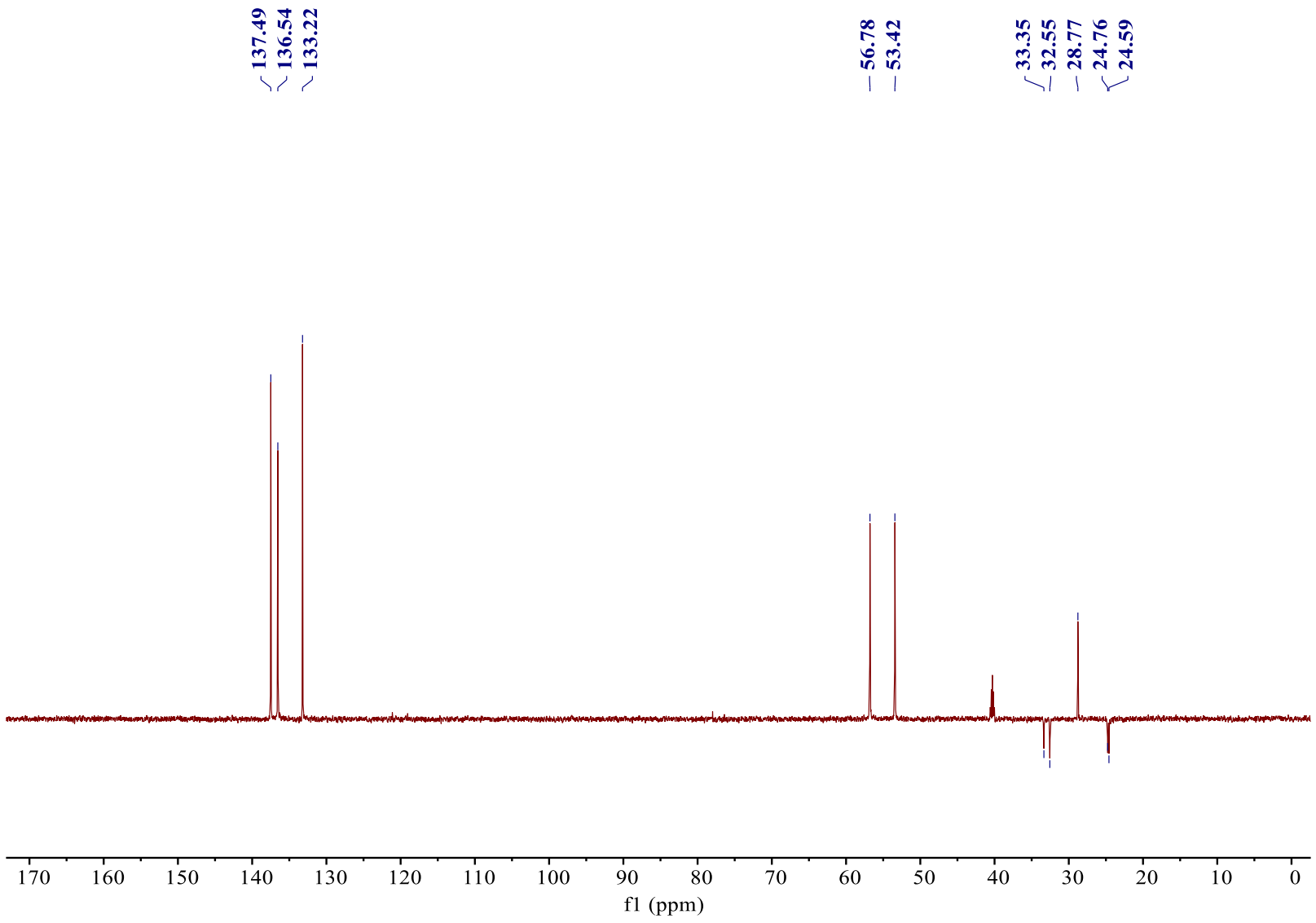

The ee-value of compound **8** was determined by HPLC analysis with a 25 cm × 4.6 mm Daicel Chiralcel IC column (hexane/i-PrOH = 90/10, 1.0 mL/min). t_{minor} = 11.2 min, t_{major} = 9.7 min; ee = 99.8 %. The racemic sample used in the HPLC analysis was synthesized by replacing (1*R*,2*R*)-cyclohexane-1,2-diamine with (±)-*trans*-1,2-diaminocyclohexane under the identical synthetic procedure.

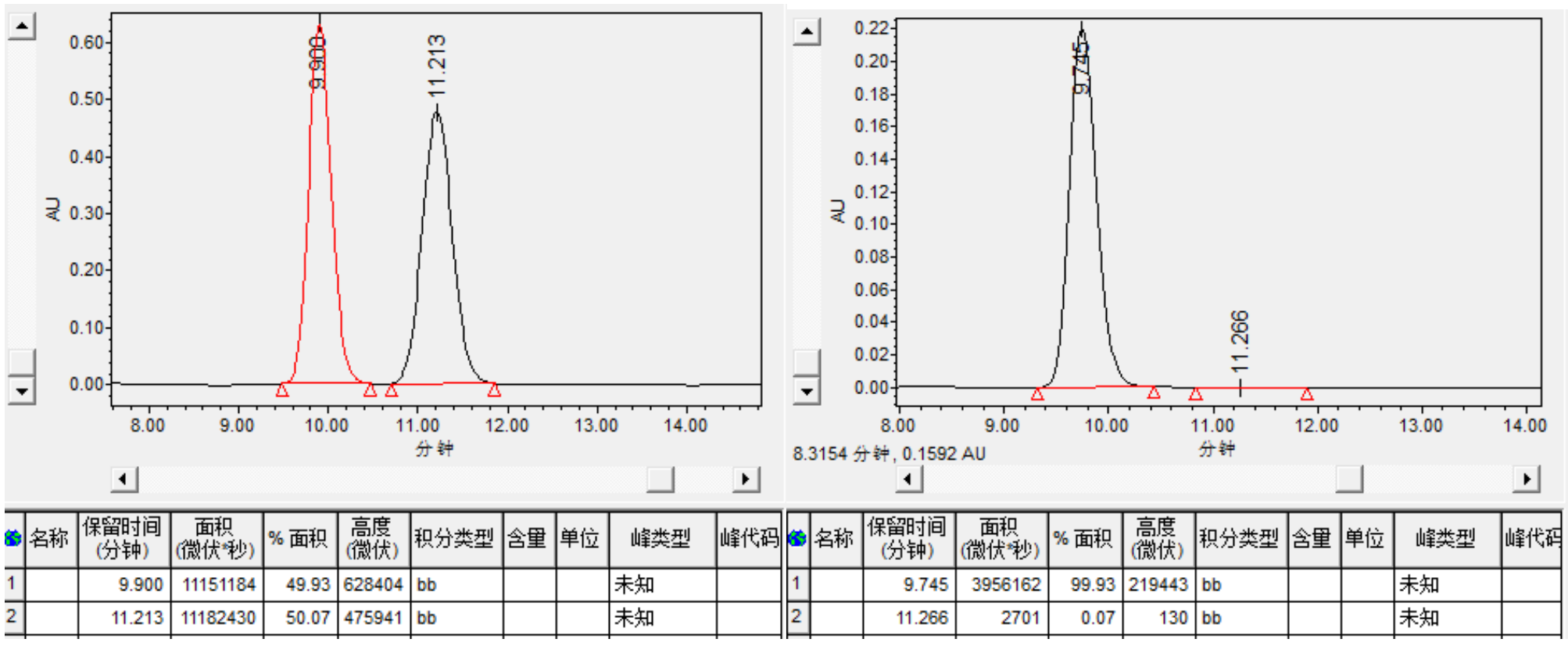

Peaks number	Retention time (min)	Area	Area percentage (%)	Height of peaks
1	9.745	3956162	99.93	219443
2	11.266	2701	0.07	130

(*R*,*R*)-DACH-NH-Boc (Compound 9):

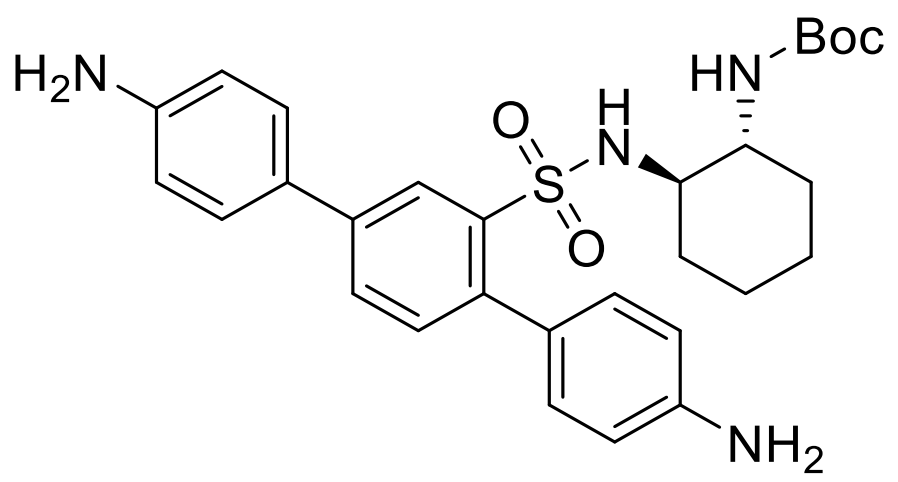

^{1}H NMR (600 MHz, DMSO-D_6) δ 8.14 – 8.13 (d, J = 2.0 Hz, 1H), 7.75 – 7.70 (dd, J = 8.0, 2.0 Hz, 1H), 7.65 – 7.54 (m, 2H), 7.46 – 7.43 (m, 2H), 7.27 – 7.20 (d, J = 8.0 Hz, 1H), 7.12 – 7.06 (d, J = 8.3 Hz, 2H), 6.91 – 6.84 (d, J = 8.3 Hz, 1H), 6.72 – 6.65 (m, 2H), 6.61 – 6.54 (m, 2H), 6.27 – 6.20 (d, J = 8.1 Hz, 1H), 5.41 – 5.33 (m, 2H), 5.25 – 5.15 (s, 2H), 3.15 – 3.09 (d, J = 9.9 Hz, 1H), 2.89 – 2.80 (dd, J = 8.4, 4.9 Hz, 1H), 1.83 – 1.67 (d, J = 11.9 Hz, 1H), 1.52 – 1.43 (q, J = 13.4 Hz, 3H), 1.35 – 1.31 (s, 9H), 1.18 – 1.03 (m, 2H); ^{13}C NMR (151 MHz, DMSO-$D6$) δ 24.4, 24.6, 28.8, 32.2, 53.7, 56.9, 78.2, 113.6, 114.8, 125.1, 126.1, 127.4, 127.7, 128.5, 129.3, 129.3, 130.6, 132.0, 132.1, 132.6, 132.9, 133.6, 134.0, 138.6, 139.4, 141.6, 148.5, 149.5, 156.0; DEPT 135° NMR (151 MHz, DMSO-D_6) δ 24.4, 24.6, 28.8, 32.2, 53.7, 56.9, 113.6, 114.8, 125.1, 127.7, 128.5, 129.3, 129.3, 130.6, 132.0, 132.1, 132.6, 132.6, 134.0; ESI-HRMS: Found: m/z 559.2374. Calcd for $C_{29}H_{36}N_4O_4S$: $(M+Na)^+$ 559.2349.

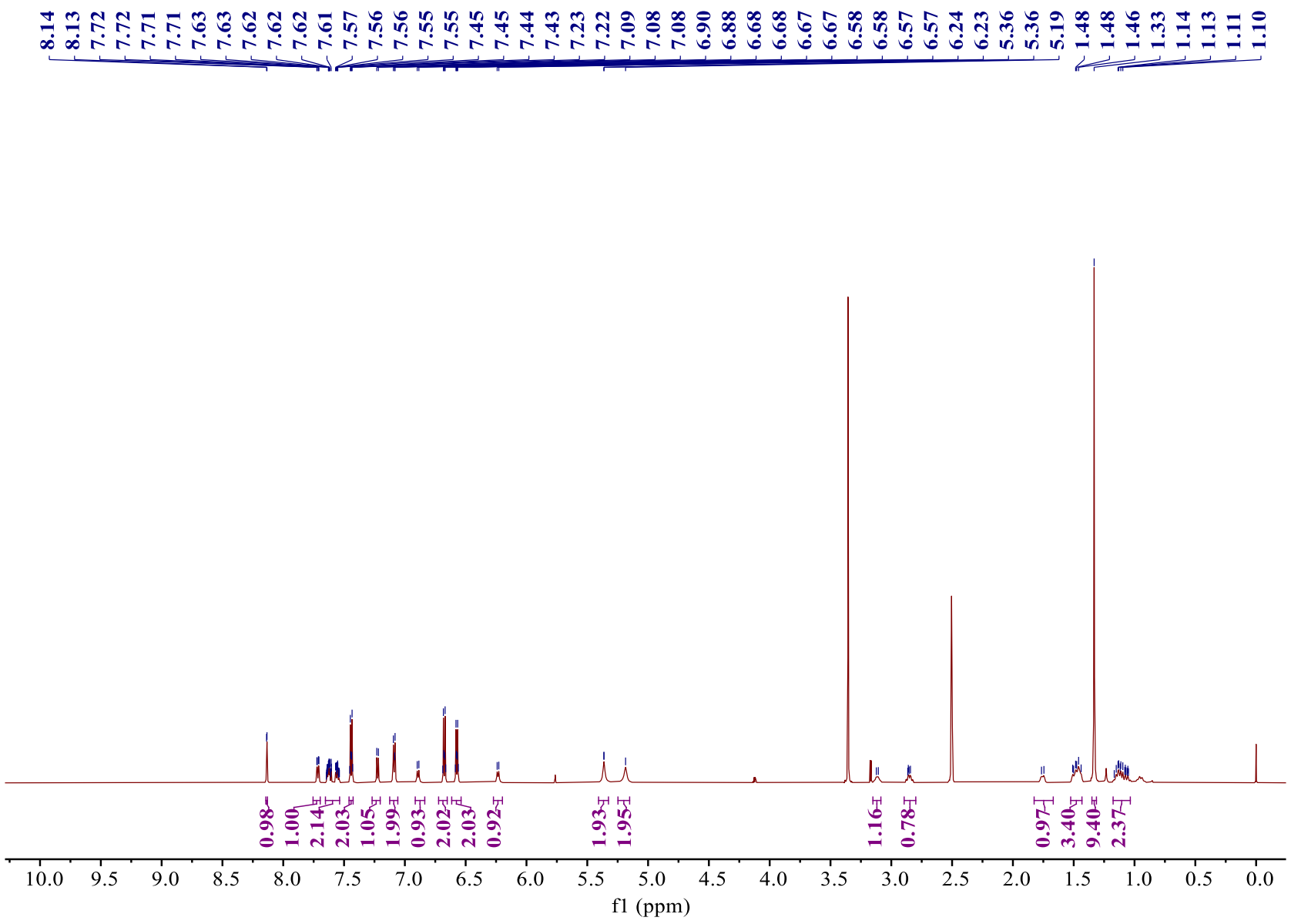

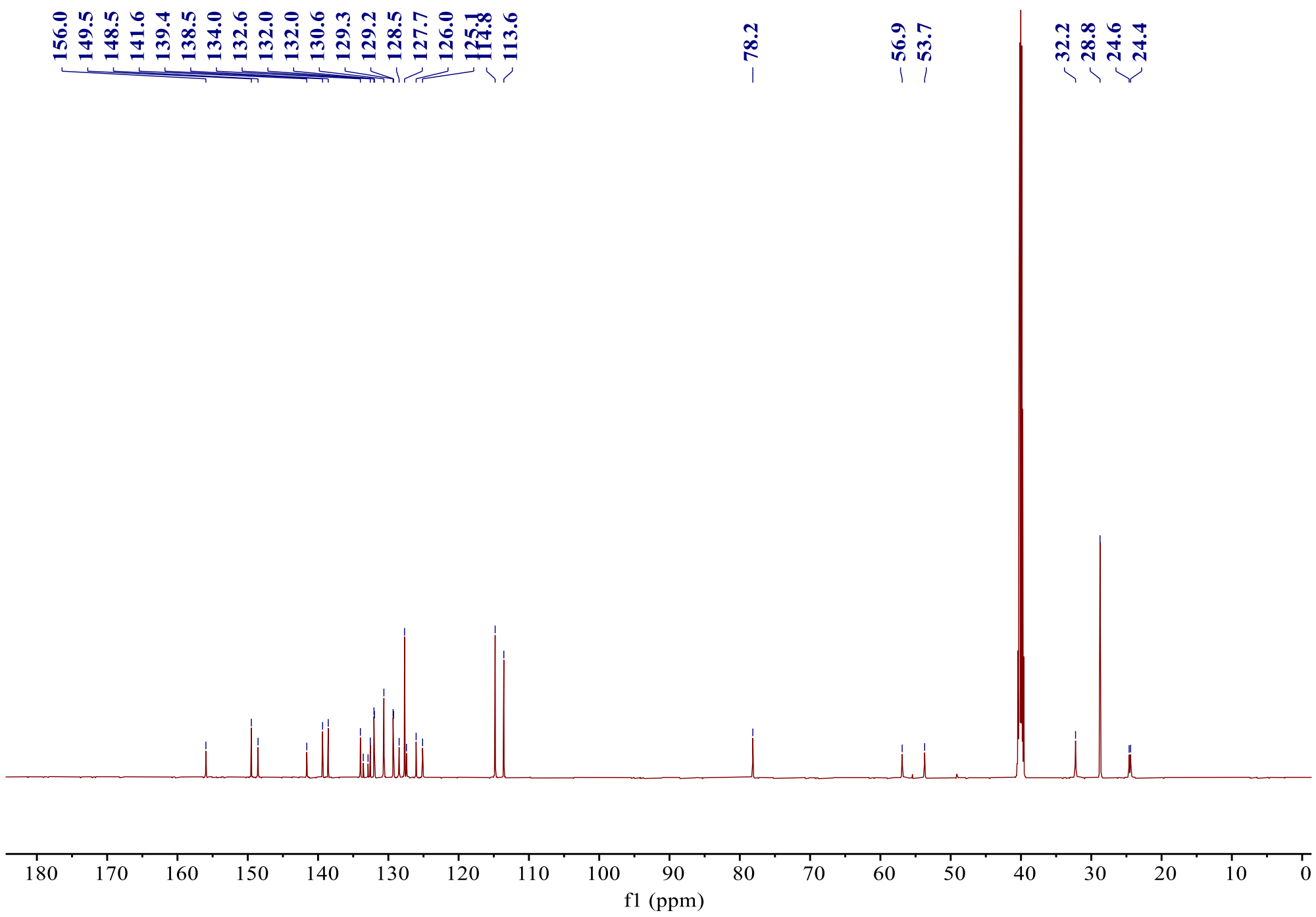

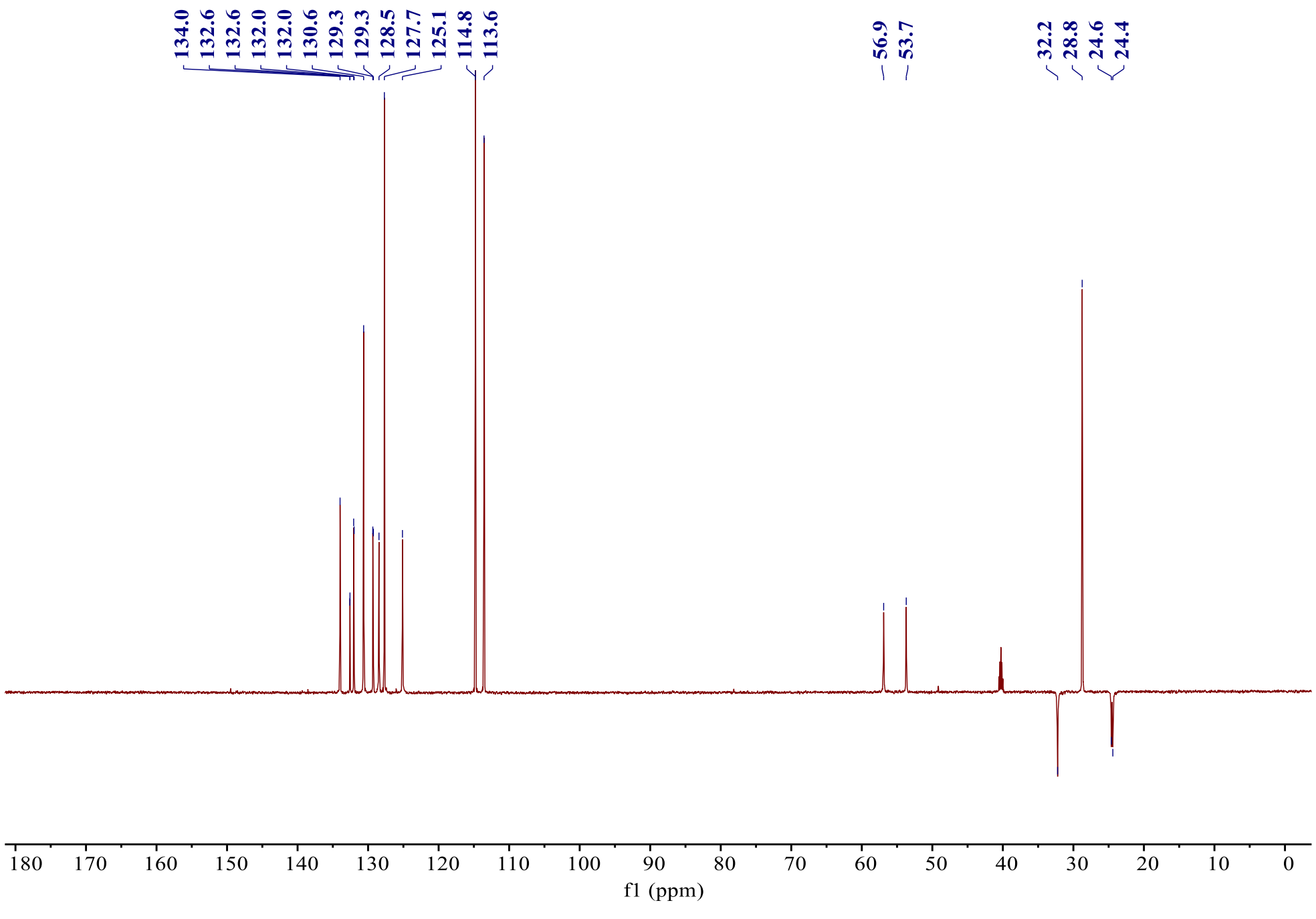

The ee-value of compound **9** was determined by HPLC analysis with a 25 cm × 4.6 mm Daicel Chiralcel IE column (hexane/i-PrOH = 60/40, 1.0 mL/min). t_{minor} = 46.0 min, t_{major} = 117.2 min; ee = 99.4 %. The racemic reference was prepared following the same synthetic procedure, starting from racemic compound **8**, which was derived from (±)-*trans*-1,2-diaminocyclohexane instead of the (1*R*,2*R*) enantiomer.

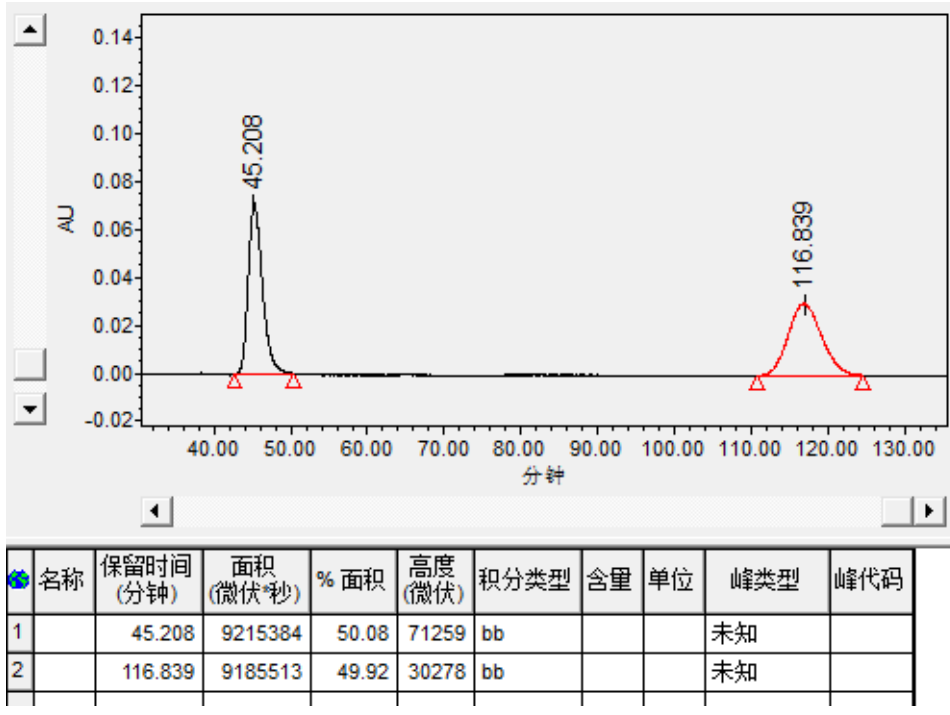

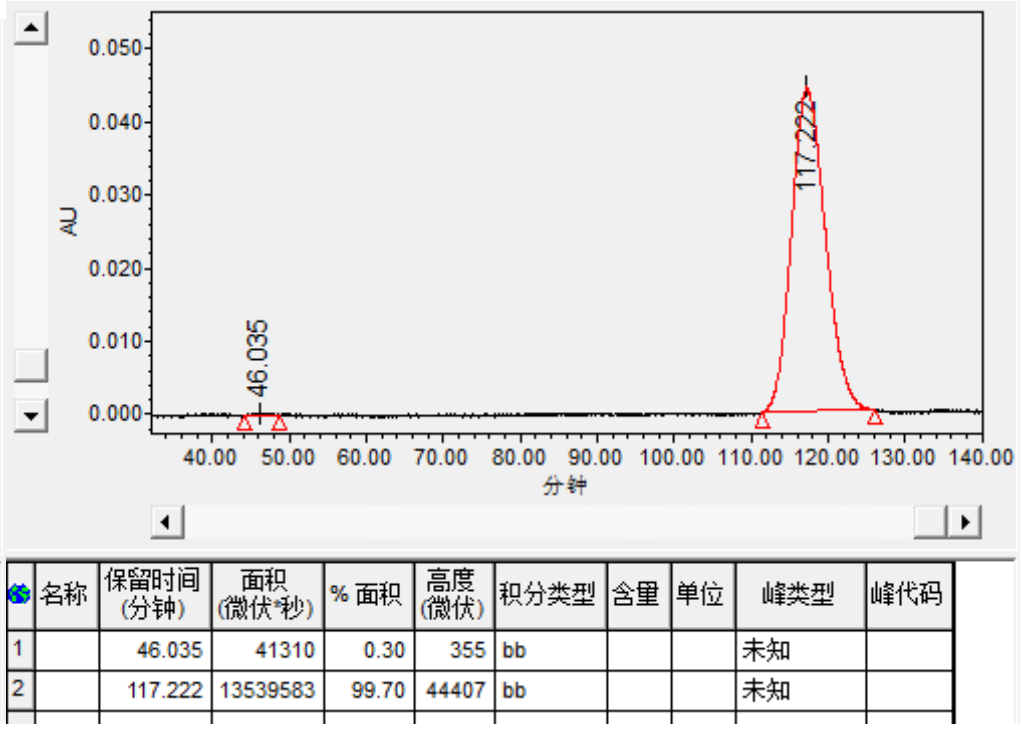

Peaks number	Retention time (min)	Area	Area percentage (%)	Height of peaks
1	46.035	41310	0.3	355
2	117.222	13539583	99.7	44407

Comment 3: Regarding the chiral precursor, retention of configuration is a key point for this work. While chiral HPLC analysis is shown, it is unclear how the racemic reference material was prepared for peak assignment. This should be explicitly described. Ideally, the authors should attempt single-crystal X-ray diffraction of the chiral precursor (R)-NH2-Boc, which would provide unambiguous confirmation of absolute configuration;

Response: We sincerely appreciate the reviewer's insightful comment and careful evaluation of our manuscript. We agree that the retention of configuration is a critical aspect of this study. The racemic reference compounds were synthesized using the corresponding racemic amine precursors under identical synthetic procedures. To clarify this, we have added a detailed description of the synthesis of the racemic samples for HPLC analysis in the Supporting Information.

Regarding the absolute configuration, we made extensive efforts to grow single crystals of the chiral precursor (*R*)-NH_2-Boc suitable for X-ray crystallographic analysis. We explored a wide variety of crystallization techniques, including vapor diffusion and liquid diffusion. Unfortunately, despite these comprehensive trials, none of the attempts yielded crystals of sufficient quality for single-crystal X-ray diffraction. Nevertheless, the absolute configurations of all compounds in the synthetic scheme have been unambiguously confirmed by chiral HPLC analysis against the newly detailed racemic standards.

Manuscript Change:

Supporting Information (Section 2. Synthetic Procedures):

We have added explicit descriptions to the SI detailing the preparation of the racemic references for all relevant compounds. For example, for compound **3** (and similarly for compounds **4**, **5**, **6**, **8**, and **9**):

"The racemic sample used in the HPLC analysis was synthesized by replacing (1*R*,2*R*)-1,2-diphenylethane-1,2-diamine with (±)-1,2-diphenylethane-1,2-diamine under the identical synthetic procedure."

Comment 4: It is also unclear why the synthesis of (R)-NH2′-Boc is included, as this compound does not appear to be used in the preparation of the COFs. As for (R)-NH2-Boc, a complete characterization of this compound is required;

Response: We appreciate the reviewer's kind reminder and valuable feedback. The compound previously denoted as (*R*)-NH2′-Boc was indeed used in the preparation of the control material CCOF0′-Ru (detailed in the original Supporting Information, Section 2.7). To eliminate any potential confusion in nomenclature and to better reflect their structural origins, we have renamed (*R*)-NH2-Boc and (*R*)-NH2′-Boc to (*R,R*)-DPEN-NH-Boc (derived from 1,2-diphenylethylenediamine, DPEN) and (*R,R*)-DACH-NH-Boc (derived from *trans*-1,2-diaminocyclohexane, DACH), respectively, throughout the revised manuscript and Supporting Information.

The synthesis of (*R,R*)-DACH-NH-Boc and its corresponding CCOF-DACH-Ru was conducted specifically to verify the necessity of our multicomponent strategy for MCCOFs. Inspired by classic Noyori-type asymmetric hydrogenation systems, we designed these two types of terphenyl-based chiral linkers. Our control experiments revealed that the DACH-based CCOF-DACH-Ru exhibits a well-ordered structure but low enantioselectivity. Conversely, the DPEN-based CCOF-DPEN-Ru provides higher ee values but suffers from poor crystallinity and stability (likely because the larger steric hindrance of the DPEN moiety disrupts interlayer stacking).

Gratifyingly, our target multivariate DPEN-DATP-based CCOF2/CCOF2-Ru successfully balances both aspects, achieving high crystallinity, excellent stability, and superior ee values. We acknowledge that this crucial comparison was insufficiently discussed in the original manuscript. We have now added a detailed explanation to justify the inclusion of (*R,R*)-DACH-NH-Boc. Furthermore, as requested, we have fully characterized (*R,R*)-DPEN-NH-Boc in the revised Supporting Information.

Manuscript Change:

Manuscript (added text on page 13, first paragraph):

"Notably, the distinct catalytic performances of CCOF-DACH-Ru (ordered structure, low ee), CCOF-DPEN-Ru (disordered structure, moderate ee), and CCOF2-Ru

(ordered structure, high ee) fully demonstrate the necessity of the multivariate approach in the design and synthesis of MCCOFs, as it elegantly balances the requirement for high enantioselectivity in asymmetric catalysis and the adverse impact of sterically hindered chiral functionalities on the structural ordering during MCCOF fabrication."

Supporting Information (revised Supplementary Fig. 23, page 50):

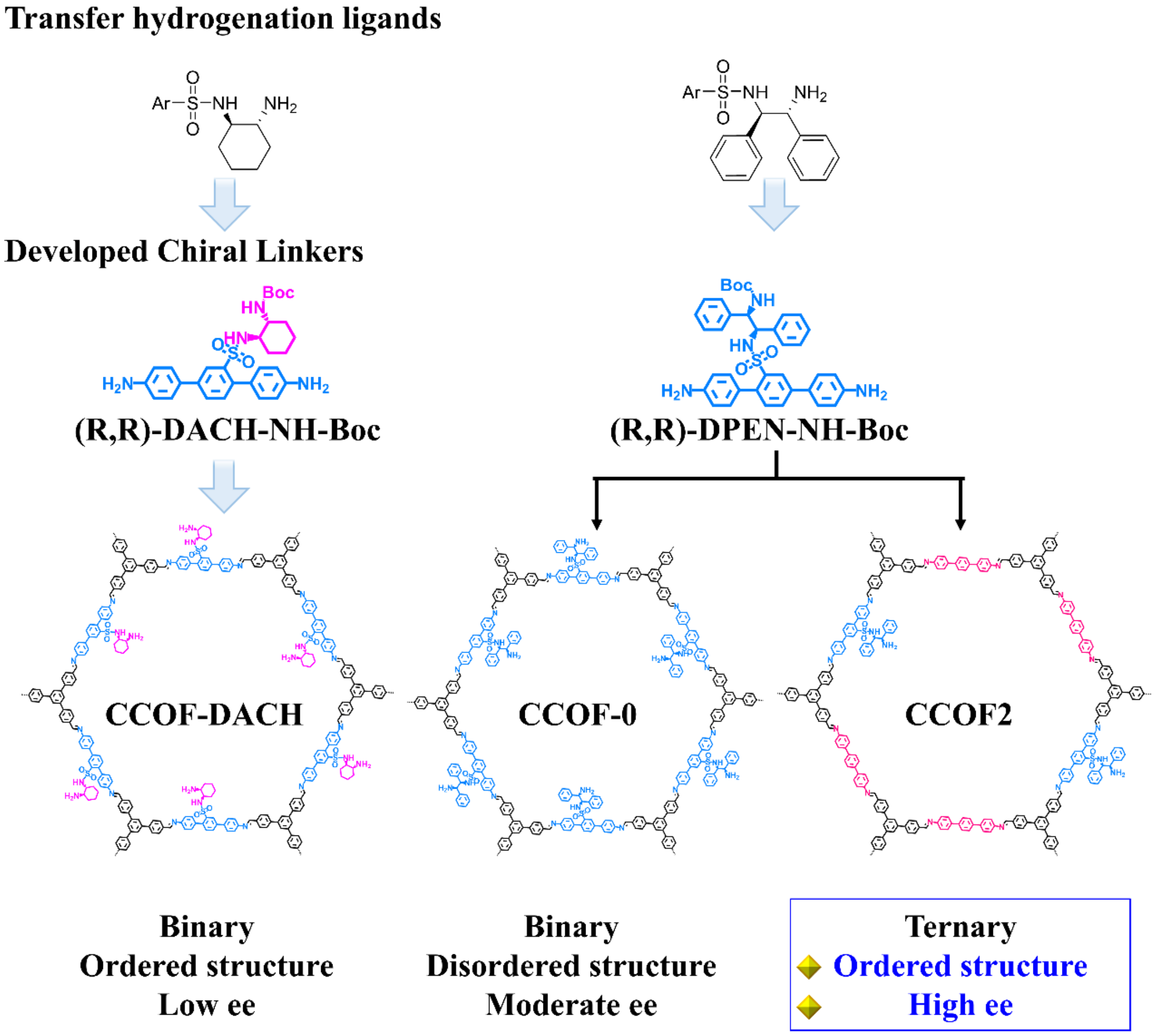

Supplementary Fig. 23 Design of Noyori-inspired CCOFs.

Supporting Information (Section 2.2, page 9):

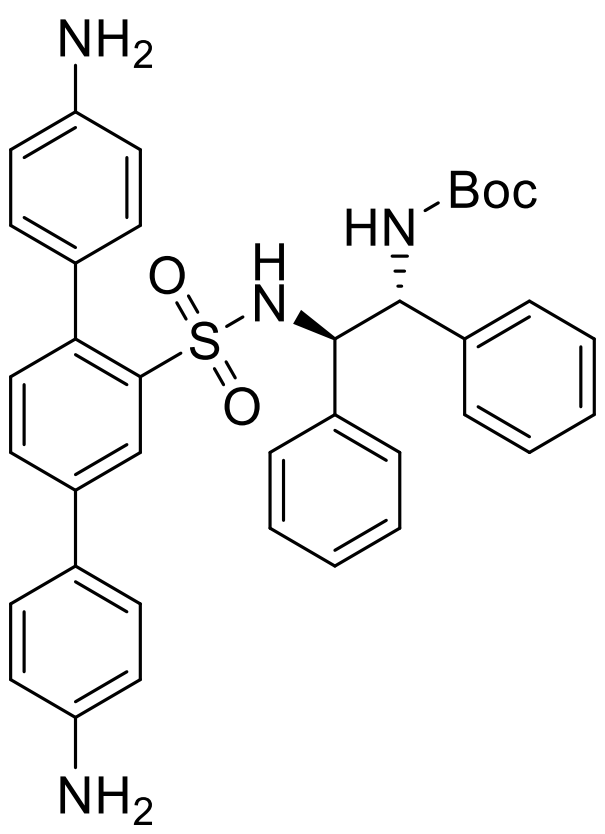

Compound **3** (5.0 g, 8.2 mmol), 4-(4,4,5,5-tetramethyl-1,3,2-dioxaborolan-2-yl)aniline (5.4 g, 24.6 mmol), potassium carbonate (K_2CO_3, 4.53g, 32.8 mmol), and tetrakis(triphenylphosphine)palladium(0) ($Pd(PPh_3)_4$, 0.47 g, 0.41 mmol) were charged into a 250 mL three-necked flask. The reaction system was degassed and backfilled with nitrogen three times to maintain an inert atmosphere, followed by addition of a pre-degassed mixed solvent of 1,4-dioxane/water (4:1, v/v, 170 mL). The mixture was heated to 110 °C and stirred at this temperature for 72 h. After cooling to room temperature, insoluble residues were removed by filtration, and the organic solvent was evaporated under reduced pressure.

The crude residue was extracted with dichloromethane (CH_2Cl_2), and the combined organic phase was washed with saturated aqueous brine, dried over anhydrous sodium sulfate (Na_2SO_4), and concentrated under vacuum. Purification was carried out by column chromatography on silica gel with CH_2Cl_2/methanol (25:1, v/v) as the eluent. The resulting product was dried under vacuum at 60 °C for 24 h to afford 4.63 g of pale yellow powder, identified as (*R,R*)-DPEN-NH-Boc (**4**), in 89% yield with 99.1% ee. ^{1}H NMR (600 MHz, DMSO-*D*$_6$) δ 8.02 – 7.91 (d, *J* = 9.6 Hz, 1H), 7.72 – 7.65 (d, *J* = 2.0 Hz, 1H), 7.55 – 7.42 (m, 1H), 7.20 – 7.14 (d, *J* = 9.1 Hz, 1H), 7.13 – 7.10 (dd, *J* = 7.2, 4.9 Hz, 4H), 7.10 – 7.03 (dt, *J* = 14.6, 6.8 Hz, 7H), 7.03 – 7.01 (d, *J* = 7.0 Hz, 1H), 7.01 – 6.99 (d, *J* = 7.8 Hz, 1H), 6.93 – 6.87 (d, *J* = 8.1 Hz, 2H), 6.63 – 6.59 (d, *J* = 8.0 Hz, 2H), 6.57 – 6.53 (d, *J* = 8.3 Hz, 2H), 5.39 – 5.10 (d, 4H), 4.85 – 4.79 (t, *J* = 8.3 Hz, 1H), 4.71 – 4.53 (t, *J* = 8.5 Hz, 1H), 1.34 – 1.26 (s, 9H); ^{13}C NMR (151 MHz, DMSO-*D*$_6$) δ 28.7, 60.0, 62.5, 78.7, 113.4, 114.7, 125.1, 126.1, 127.2, 127.4, 127.5, 127.6, 127.7, 127.9, 128.2, 128.2, 128.3, 130.6, 133.7, 138.4, 139.2, 139.6, 140.4, 140.8, 148.3, 149.3, 155.5; DEPT 135° NMR (151 MHz, DMSO-*D*$_6$) δ 28.7, 60.0, 62.5, 113.4, 114.7, 125.1, 127.2, 127.4, 127.6, 127.7, 127.9, 128.2, 128.2, 128.3, 130.6, 133.7. ESI-HRMS: Found: m/z 657.2537. Calcd for $C_{25}H_{26}Br_2N_2O_4S$: $(M+Na)^+$ 657.2506.

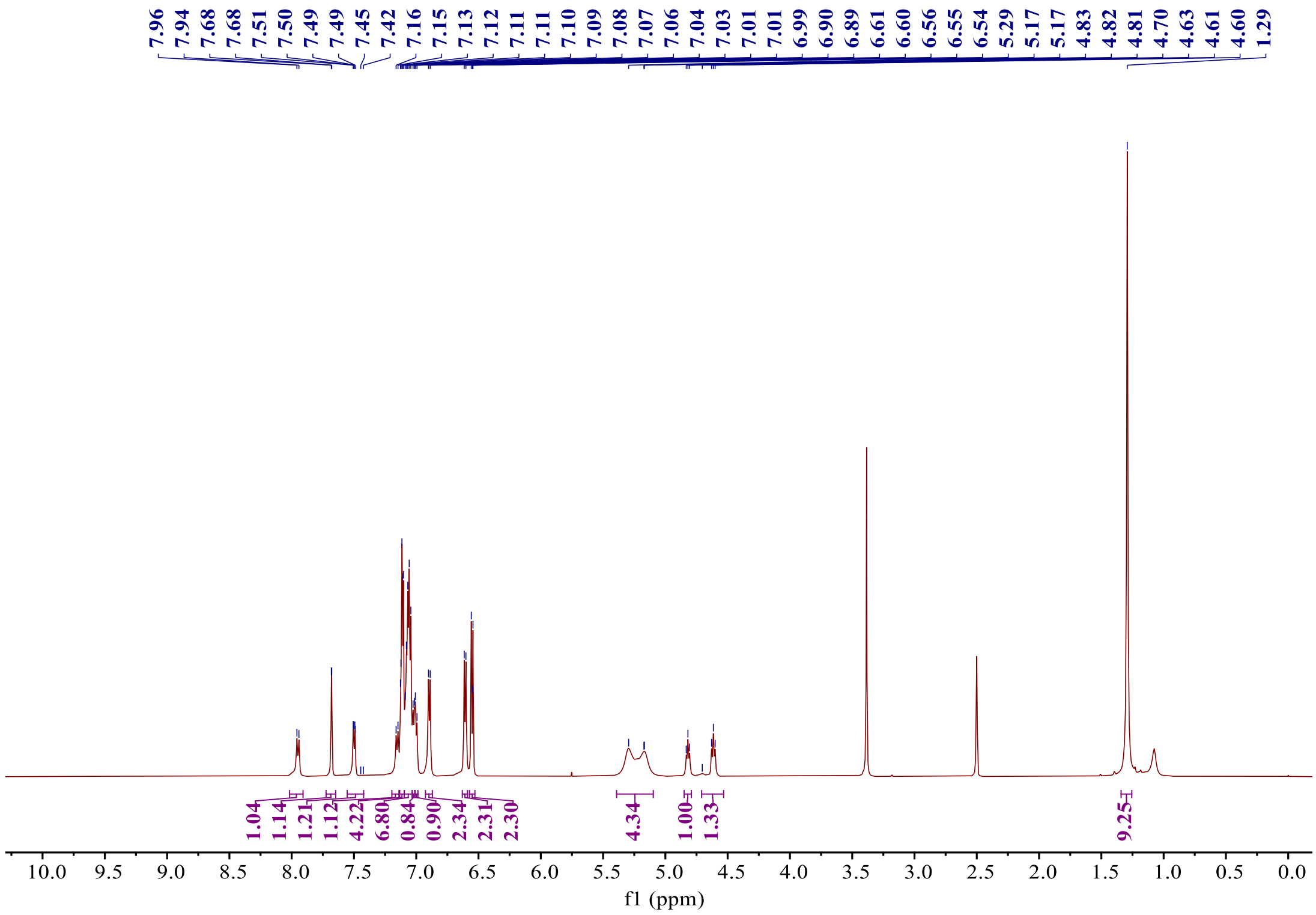

7.96
7.94
7.68
7.68
7.51
7.50
7.49
7.45
7.42
7.16
7.15
7.13
7.12
7.11
7.11
7.10
7.09
7.08
7.07
7.06
7.04
7.03
7.01
7.01
6.99
6.90
6.89
6.61
6.60
6.56
6.55
6.54
5.29
5.17
5.17
4.83
4.82
4.81
4.70
4.63
4.61
4.60
1.29
1.04
1.14
1.21
1.12
4.22
6.80
0.84
0.90
2.34
2.31
2.30
4.34
1.00
1.33
9.25
10.0 9.5 9.0 8.5 8.0 7.5 7.0 6.5 6.0 5.5 5.0 4.5 4.0 3.5 3.0 2.5 2.0 1.5 1.0 0.5 0.0
f1 (ppm)

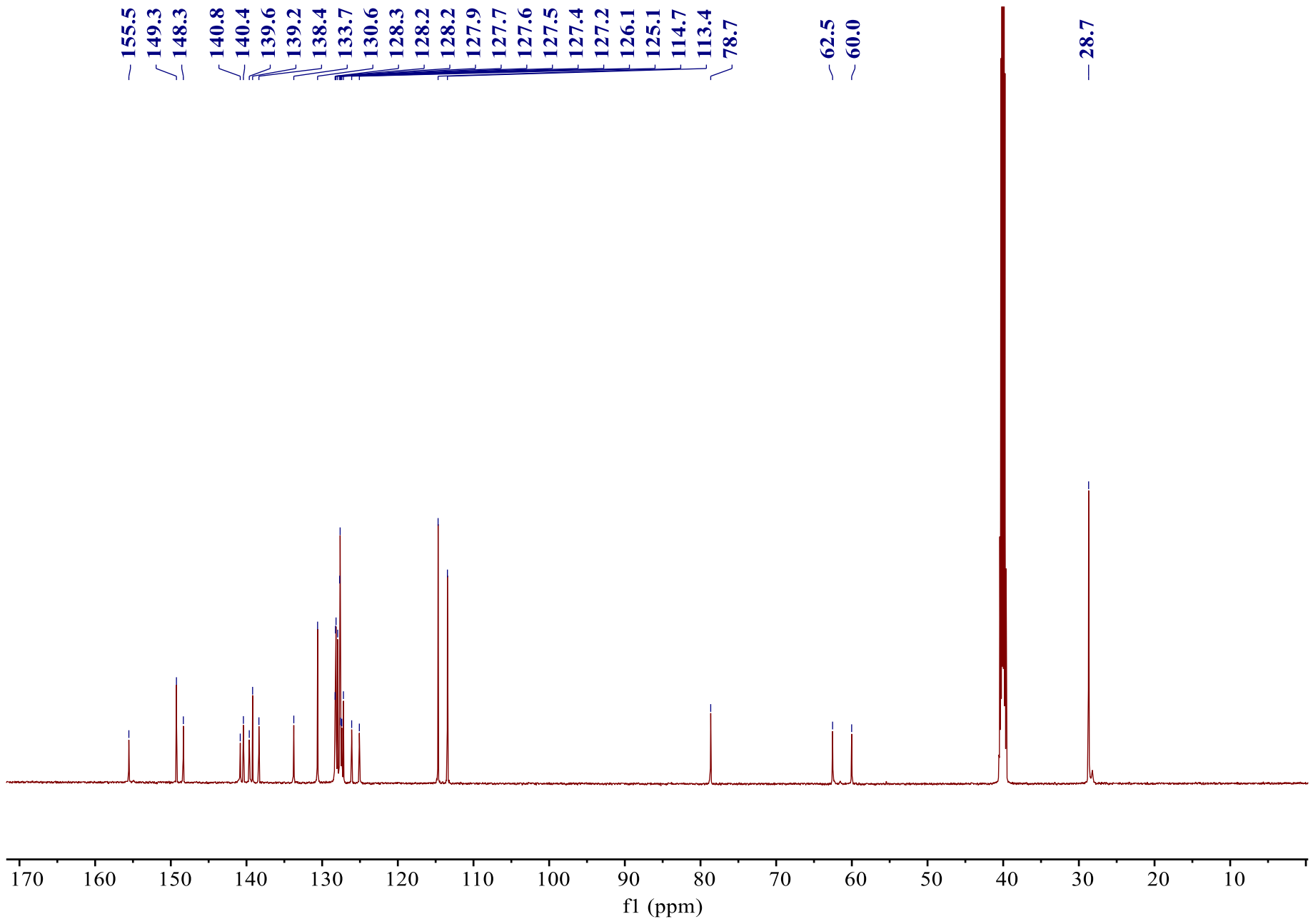

155.5
149.3
148.3
140.8
140.4
139.6
139.2
138.4
133.7
130.6
128.3
128.2
128.2
127.9
127.7
127.6
127.5
127.4
127.2
126.1
125.1
114.7
113.4
78.7
62.5
60.0
28.7
170 160 150 140 130 120 110 100 90 80 70 60 50 40 30 20 10
f1 (ppm)

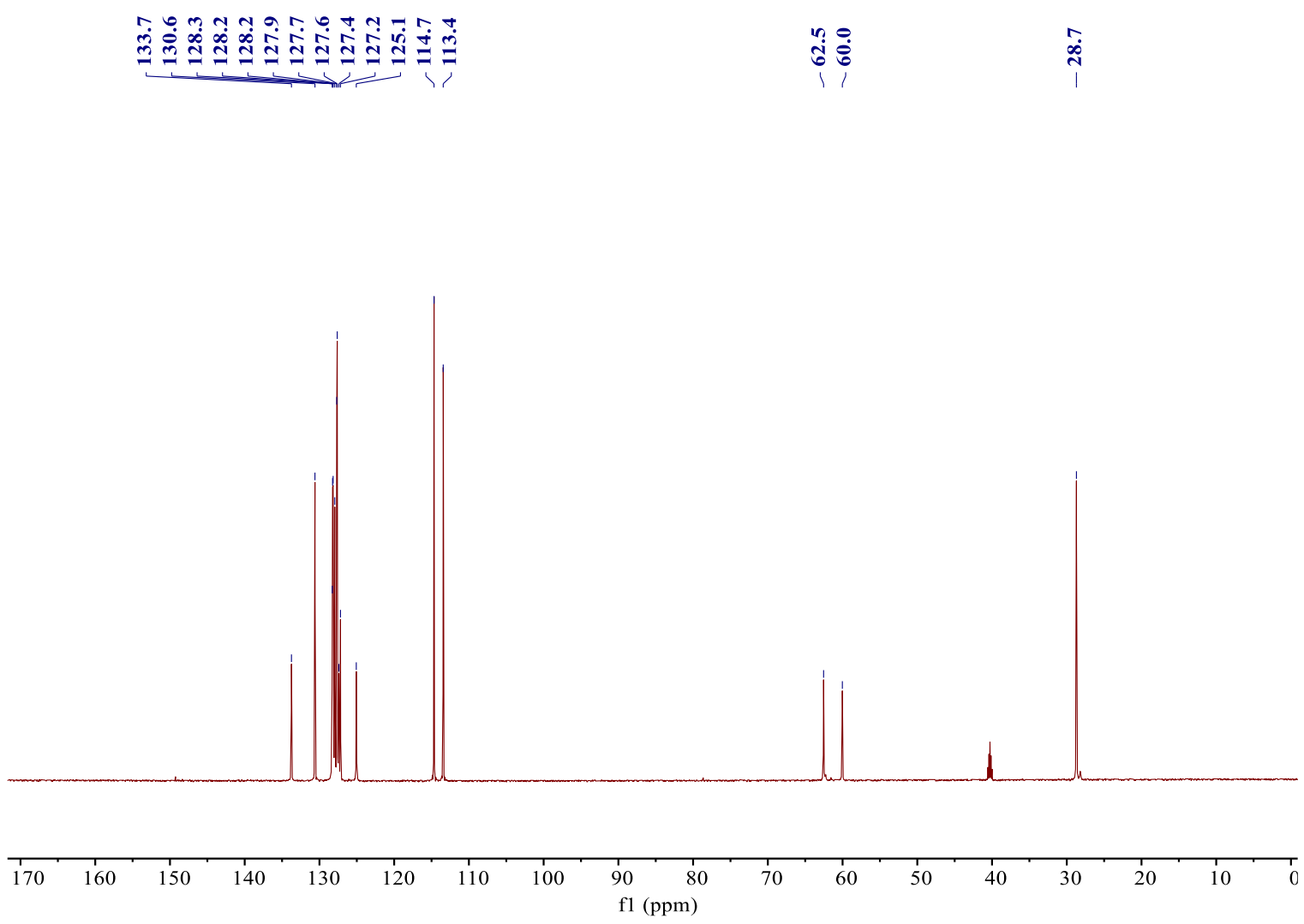

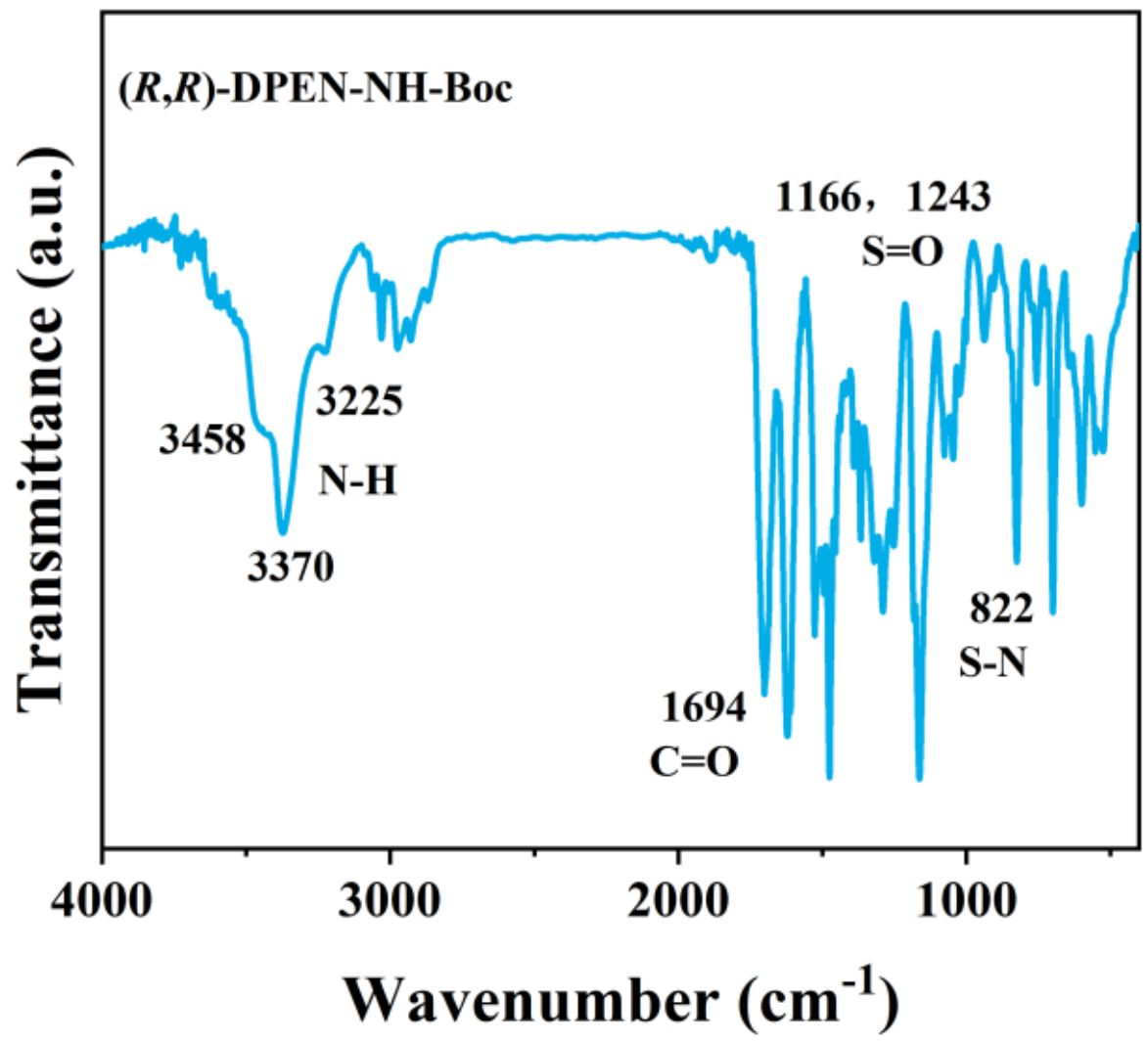

The ee-value of (*R,R*)-DPEN-NH-Boc was determined by HPLC analysis with a 25 cm × 4.6 mm Daicel Chiralcel IE column (hexane/i-PrOH = 60/40, 1.0 mL/min). t_{minor} = 33.7 min, t_{major} = 25.3 min; ee = 99.1%. The racemic reference was prepared following the same synthetic procedure, starting from racemic compound **3**, which was derived from (±)-1,2-diphenylethane-1,2-diamine instead of the (1*R*,2*R*) enantiomer.

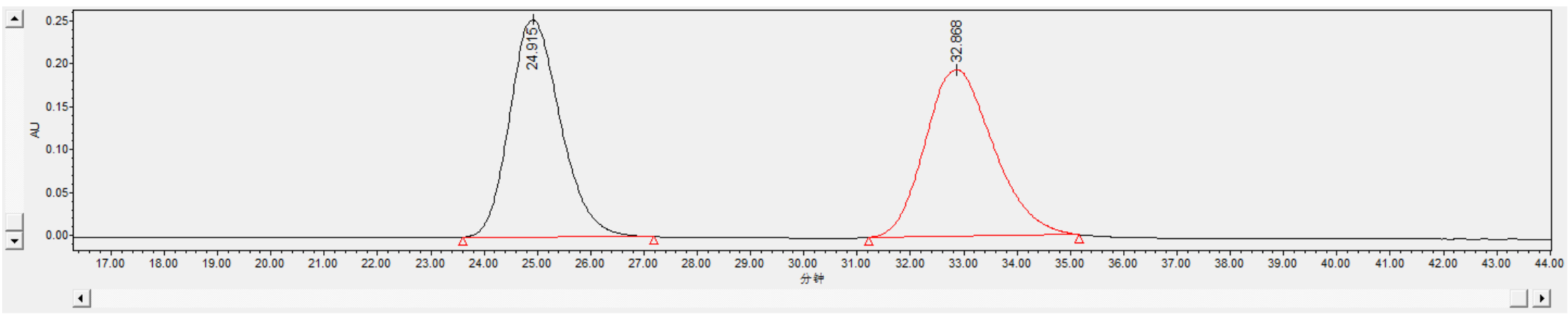

	名称	保留时间 (分钟)	面积 (微伏*秒)	% 面积	高度 (微伏)	积分类型	含量	单位	峰类型	峰代码
1		24.915	16773440	49.21	252852	bb			未知	
2		32.868	17313878	50.79	194149	bb			未知	

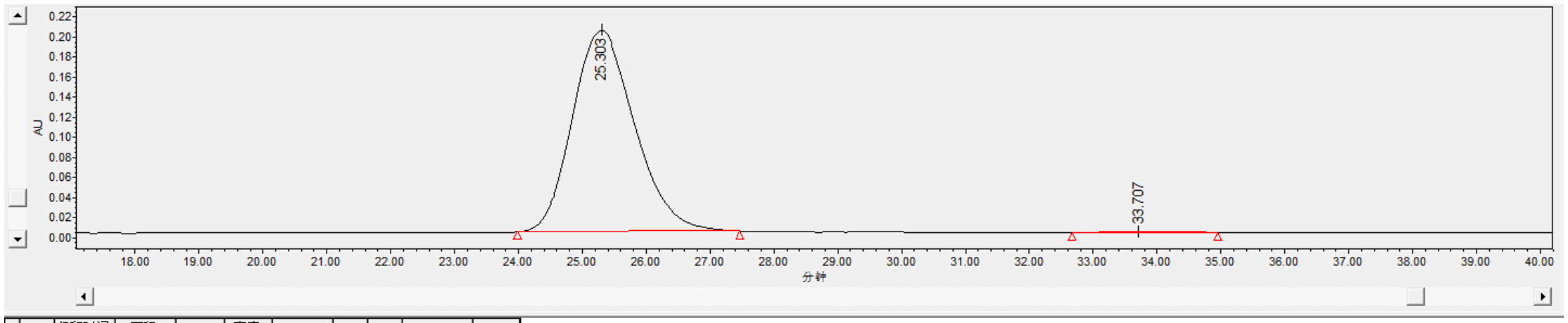

	名称	保留时间 (分钟)	面积 (微伏*秒)	% 面积	高度 (微伏)	积分类型	含量	单位	峰类型	峰代码
1		25.303	13553707	99.56	200426	bb			未知	
2		33.707	59393	0.44	875	bb			未知	

Peaks number	Retention time (min)	Area	Area percentage (%)	Height of peaks
1	25.303	13553707	99.56	200426
2	33.707	59393	0.44	875

Comment 5: For the synthesis of the CCOFX materials, an important unresolved issue concerns the stability of the Boc protecting group. The COF synthesis conditions (120 °C, acidic medium, three days) are typically incompatible with Boc protection.

Response: We thank the reviewer for raising this highly insightful and rigorous question. The stability of the Boc protecting group during solvothermal synthesis is indeed a critical factor to consider.

First, we would like to clarify from a chemical perspective that while Boc is acid-sensitive, its quantitative cleavage typically requires strong acids (such as trifluoroacetic acid or HCl). In our COF synthesis, we employ aqueous acetic acid (a weak acid, pKa ≈ 4.76) in relatively low concentrations merely as a catalyst for imine condensation. Under these specific mildly acidic conditions, the Boc group exhibits sufficient kinetic stability, even at 120 °C. This is also consistent with several successful precedents in the literature where Boc-protected COFs were synthesized under similar solvothermal conditions (e.g., *J. Am. Chem. Soc.* **2023**, *145*, 18015; *Angew. Chem. Int. Ed.* **2019**, *58*, 9443). More importantly, we have solid experimental evidence to confirm the retention of the Boc groups in our synthesized materials. The FTIR, solid-state NMR, and TGA profiles of the as-synthesized CCOF-Boc materials clearly demonstrate that the Boc groups remain largely intact after the 3-day reaction at 120 °C.

To further justify the chosen temperature, we performed control experiments by synthesizing the CCOFs at a lower temperature (100 °C). The comparative PXRD results reveal that the materials prepared at 120 °C exhibit significantly stronger and sharper diffraction peaks. This indicates that 120 °C is thermodynamically necessary to ensure sufficient error-correction (reversibility) for the formation of highly crystalline frameworks. Therefore, 120 °C represents the optimal balance between achieving high crystallinity and maintaining Boc stability.

Manuscript Change:

Supporting Information (revised Supplementary Fig. 1, page 35):

We have added the comparative PXRD data (100 °C vs 120 °C) to the Supporting Information (Supplementary Fig. 1; Page 35) to justify the synthetic temperature.

We have added a brief discussion in the Supporting Information regarding the stability of the Boc group under our mildly acidic solvothermal conditions, supported by the FTIR, solid-state NMR, and TGA characterizations.

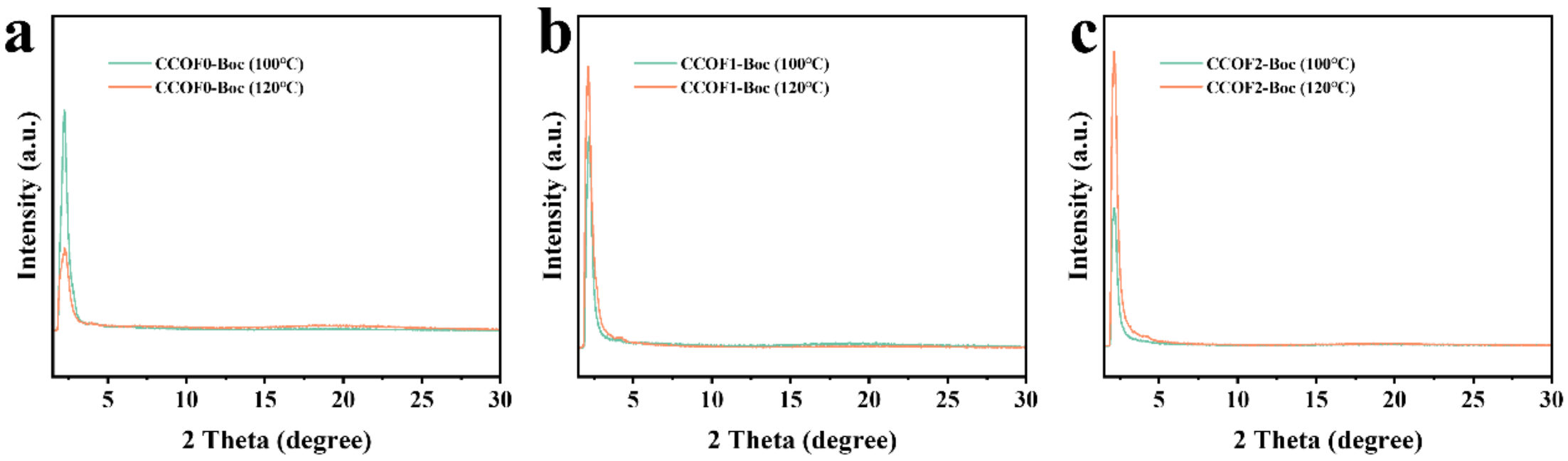

Supplementary Fig. 1 PXRD patterns of Boc-protected CCOFX-Boc synthesized at 100 °C and 120 °C. CCOF0-Boc: higher crystallinity at 100 °C; CCOF1-Boc and CCOF2-Boc: higher crystallinity at 120 °C. The retention of a substantial amount of Boc groups in CCOF1-BOC and CCOF2-BOC was confirmed by FTIR (Supplementary Fig. 2, the C=O peak of Boc at 1694 cm^{-1}), solid-state NMR (Supplementary Fig. 3, δ = 80, 27 ppm) and TGA curves (Supplementary Fig. 9, an obvious mass loss starting at approximately 210 °C). These results verify that the employed solvothermal conditions do not significantly affect the synthesis of the CCOFs.

Comment 6: In addition, the proposed distributions of DATP and (R)-NH2-Boc units in CCOF1 and CCOF2 cannot be experimentally verified without single-crystal data. At a minimum, the authors should provide clear theoretical structural models for each material, including full atomic positions and not only AA stacking motifs. The computational methods used to generate these models should be described in detail. Pawley or Le Bail refinements should be performed to demonstrate that alternative structural models can be excluded. Theoretical pore volumes and BET surface areas should also be provided for comparison with experimental values;

Response: We sincerely appreciate the reviewer pointing out this omission. We had provided the complete atomic coordinates of the theoretical structural models in the original SI, but we did not include clear and intuitive visualizations. We have now added

the optimized structural models with AA-stacking and AB-stacking arrangements in Supplementary Fig. 6. In addition, alternative structural models (AB-staggered) were excluded via Pawley refinements, with the corresponding results presented in the revised figures (Fig. 2c and Figs. 3a–d).

The theoretical BET surface areas and pore volumes were directly calculated from the simulated structures using Zeo++, and the results are summarized in Supplementary Table 2. Obvious differences are observed between the theoretical values and the experimental data, which may be related to the ordering degree of the as-synthesized CCOFs. We have added a detailed discussion regarding this discrepancy.

Manuscript Change:

Manuscript (revised text on page 7, second paragraph; revised Fig 2c, page 6; revised Fig 3a-d, Page 8):

"As shown in Supplementary Table 2, obvious differences exist between the theoretical pore volumes and BET surface areas calculated via Zeo++ from the simulated structures and the experimental values, which may be related to the ordering degree of the as-synthesized CCOFs. The low surface areas of CCOF0-Boc (CCOF-DPEN-Boc) and CCOF1-Boc can be rationalized by their relatively low crystallinity and the possible presence of minor disordered impurities within the pore structures. In contrast, the significantly improved BET surface area of CCOF2-Boc is consistent with its enhanced crystallinity."

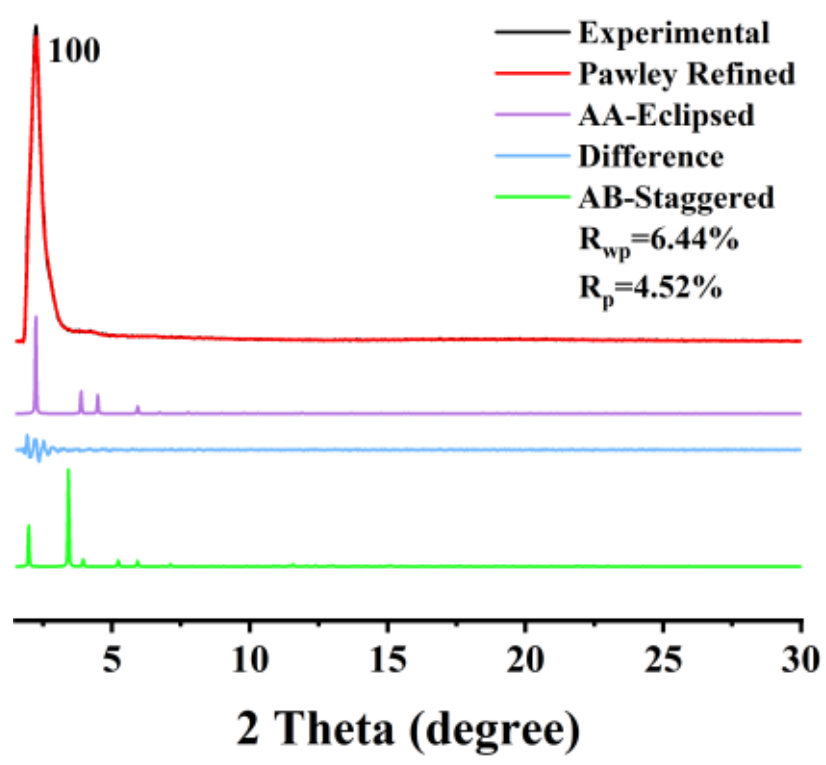

Fig. 2 c Experimental PXRD patterns of CCOF0-Boc.

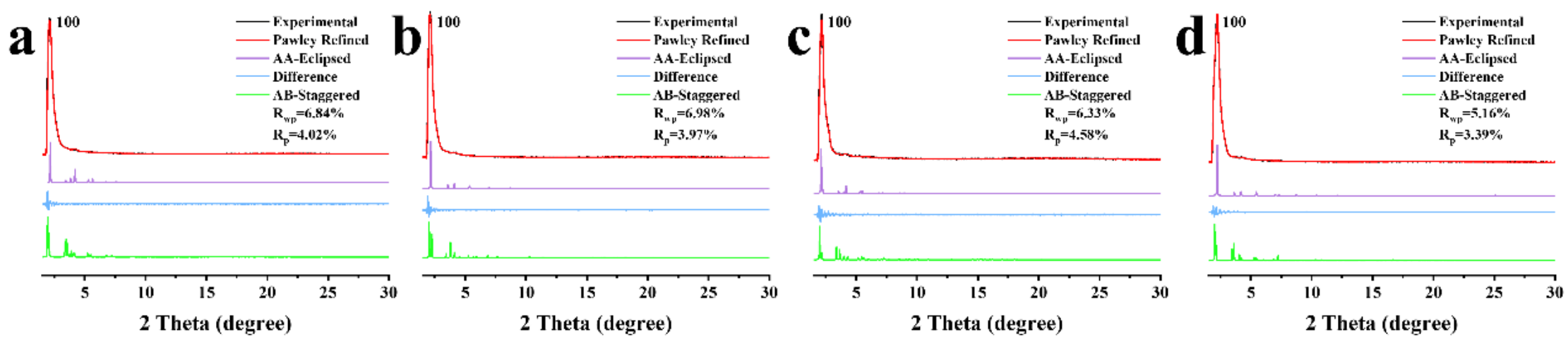

Fig. 3 Experimental PXRD patterns of **a** CCOF1-Boc, **b** CCOF2-Boc, **c** CCOF1, and

d CCOF2.

Supporting Information (added Figure on Page 39, Supplementary Fig. 6; added table on Page 55, Supplementary Table 2):

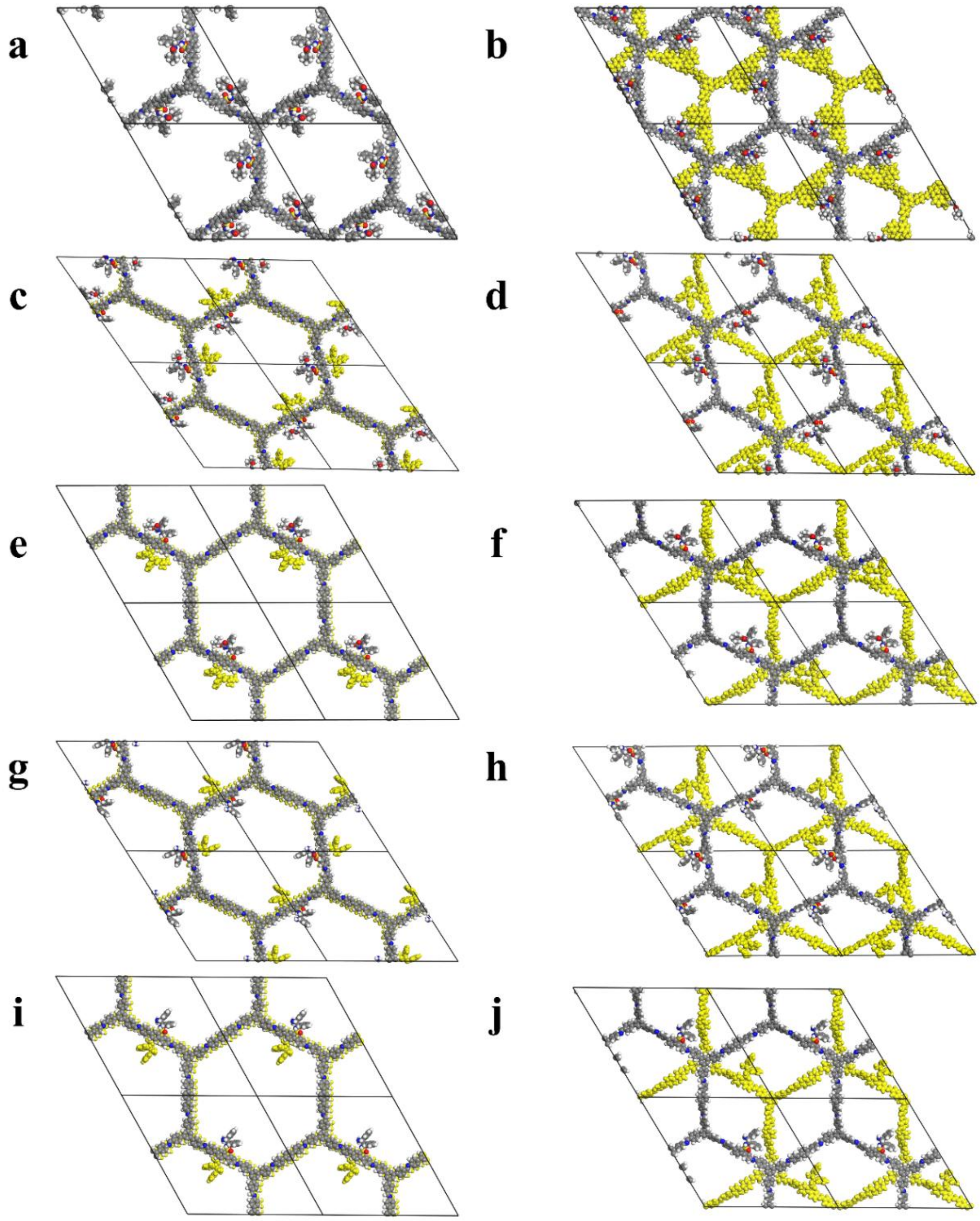

Supplementary Fig. 6 Optimized AA-eclipsed structural models of (a) CCOF0-Boc, (c) CCOF1-Boc, (e) CCOF2-Boc, (g) CCOF1, and (i) CCOF2; AB-staggered structural models of (b) CCOF0-Boc, (d) CCOF1-Boc, (f) CCOF2-Boc, (h) CCOF1, and (j) CCOF2.

Supplementary Table 2. Theoretical BET surface area and pore volumes of CCOFs.

COFs	BET surface areas (m^2/g)	Theoretical BET surface area (m^2/g)	Pore volumes (cm^3/g)	Theoretical pore volumes (cm^3/g)
CCOF0-Boc	21	1983	0.02	1.48
CCOF1-Boc	70	2187	0.03	1.71
CCOF2-Boc	518	2304	0.33	2.24
CCOF1	40	2422	0.02	2.01
CCOF2	473	2468	0.36	2.39

Comment 7: Several aspects of the materials characterization require improvement: Complete elemental analysis (C, H, N, S) should be reported for all COFs, rather than only N:S ratios;

Response: We sincerely appreciate the reviewer's valuable comment. We have supplemented the complete elemental analysis (C, H, N, S) data together with the corresponding theoretical values in the Supporting Information.

Manuscript Change:

Supporting Information (revised Supplementary Table 1, Page 54):

We have updated Supplementary Table 1 to include the complete C, H, N, and S weight percentages for CCOF1-Boc and CCOF2-Boc, alongside their theoretical values.

Supplementary Table 1. Element analysis data.[a]

COFs	C (wt%)	H (wt%)	N (wt%)	S (wt%)	$n_{(N)}/n_{(S)}$
CCOF1-Boc	76.33	5.22	6.05	2.49	5.55/1
Theoretical Value	79.61	5.31	6.36	2.91	5/1
CCOF2-Boc	79.75	5.18	5.67	1.68	7.71/1
Theoretical Value	83.42	5.18	6.13	1.75	8/1

[a]The elemental analysis data are reported as the average of two independent measurements.

Comment 8: The nitrogen adsorption isotherms show very low surface areas compared to those expected from the proposed structures, suggesting a significant fraction of

amorphous material or impurities;

Response: We highly appreciate the reviewer's insightful comment. We agree that a thorough discussion regarding the BET surface areas is necessary. The relatively low surface areas of the homomeric COFs (CCOF0-Boc, and CCOF1-Boc) compared to theoretically idealized bare frameworks are not due to impurities, but rather stem from their intrinsic structural features.

The chiral linkers bear sterically demanding Boc-protected diamine moieties (DPEN). These bulky pendent groups protrude into the 1D hexagonal channels of the COFs, intrinsically occupying a significant portion of the free void volume. Furthermore, in 2D COFs bearing such bulky side chains, adjacent layers often undergo sliding or staggered stacking to minimize severe interlayer steric repulsion. This structural deformation and layer offset severely block the accessible pores, which naturally leads to the low crystallinity and reduced BET surface areas observed for the homomeric COFs. This phenomenon of porosity reduction due to steric congestion is well-documented in the literature (e.g., *J. Am. Chem. Soc.* **2023**, *145*, 21798−21806; *J. Am. Chem. Soc*. **2017**, *139*, 10506−10513).

Gratifyingly, this is exactly where our multivariate strategy demonstrates its superiority. In the multivariate CCOF2-Boc, the random distribution of two structurally distinct chiral linkers effectively disrupts the uniform steric clash, providing significant steric relief. This allows the framework to adopt a more ordered, eclipsed-like stacking with higher crystallinity, thereby keeping the 1D channels more open and accessible. Consequently, CCOF2-Boc exhibits a significantly improved BET surface area. We have incorporated this structural rationale into the revised manuscript to better explain the porosity trends.

Manuscript Change:

Manuscript (rvised text on pge 7, scond pragraph):

"The relatively low BET surface areas of the CCOF0-Boc and CCOF1-Boc can be attributed to the steric hindrance induced by the bulky Boc-protected chiral moieties. These sterically demanding groups not only occupy the internal pore volume but also likely induce interlayer sliding or staggered stacking to minimize steric repulsion, leading to reduced crystallinity and partially blocked channels.[61, 62] In stark contrast, the multivariate strategy in MCCOF-Boc effectively alleviates this interlayer steric tension, promoting a more ordered framework stacking. This structural optimization is directly reflected in its significantly enhanced crystallinity and substantially higher BET surface area."

Reference:

61. Pelkowski CE, *et al.* Tuning Crystallinity and Stacking of Two-Dimensional Covalent Organic Frameworks through Side-Chain Interactions. *J. Am. Chem. Soc.* **145**, 21798-21806 (2023).
62. Thompson CM, *et al.* Computational and Experimental Studies on the Effects of Monomer Planarity on Covalent Organic Framework Formation. *J. Am. Chem. Soc.* **139**, 10506-10513 (2017).

Comment 9: This interpretation is supported by the TGA data, which show unusually high residual masses (50–60%) at 800 °C for COF materials, indicating the presence of substantial inorganic or metallic residues;

Response: We are extremely grateful to the reviewer for pointing out this anomaly, which led us to discover a critical typographical error in our Supporting Information. We sincerely apologize for this oversight and the resulting confusion. The TGA measurements were actually performed under a nitrogen atmosphere, not in air as mistakenly described in the original SI.

We completely agree with the reviewer that if the TGA were conducted in air, a 50–60% residual mass would strongly indicate the presence of substantial inorganic or metallic impurities. However, under an inert N_2 atmosphere, the highly aromatic and carbon-rich (>70%) backbones of purely organic COFs undergo thermal carbonization rather than complete combustion. This process naturally yields a stable carbonaceous residue.

A residual mass of 50–60% at 800 °C under N_2 is a well-documented and standard characteristic of purely organic COF materials, as supported by numerous authoritative studies (e.g., *J. Am. Chem. Soc.* **2016**, *138*, 11489; *J. Am. Chem. Soc.* **2017**, *139*, 8277; *Nat. Commun.* **2022**, *13*, 2615; *Angew. Chem. Int. Ed.* **2024**, *63*, e202405769). Therefore, the observed residue consists entirely of carbonized organic framework, which firmly rules out the presence of inorganic or metallic impurities in our pristine COFs.

Manuscript Change:

Supporting Information (revised Supplementary Fig. 9, page 42):

We have corrected the TGA atmosphere description in Section 1 of the SI and added a brief note in Supplementary Fig. 9 clarifying that the residual mass corresponds to carbonization under the N_2 atmosphere.

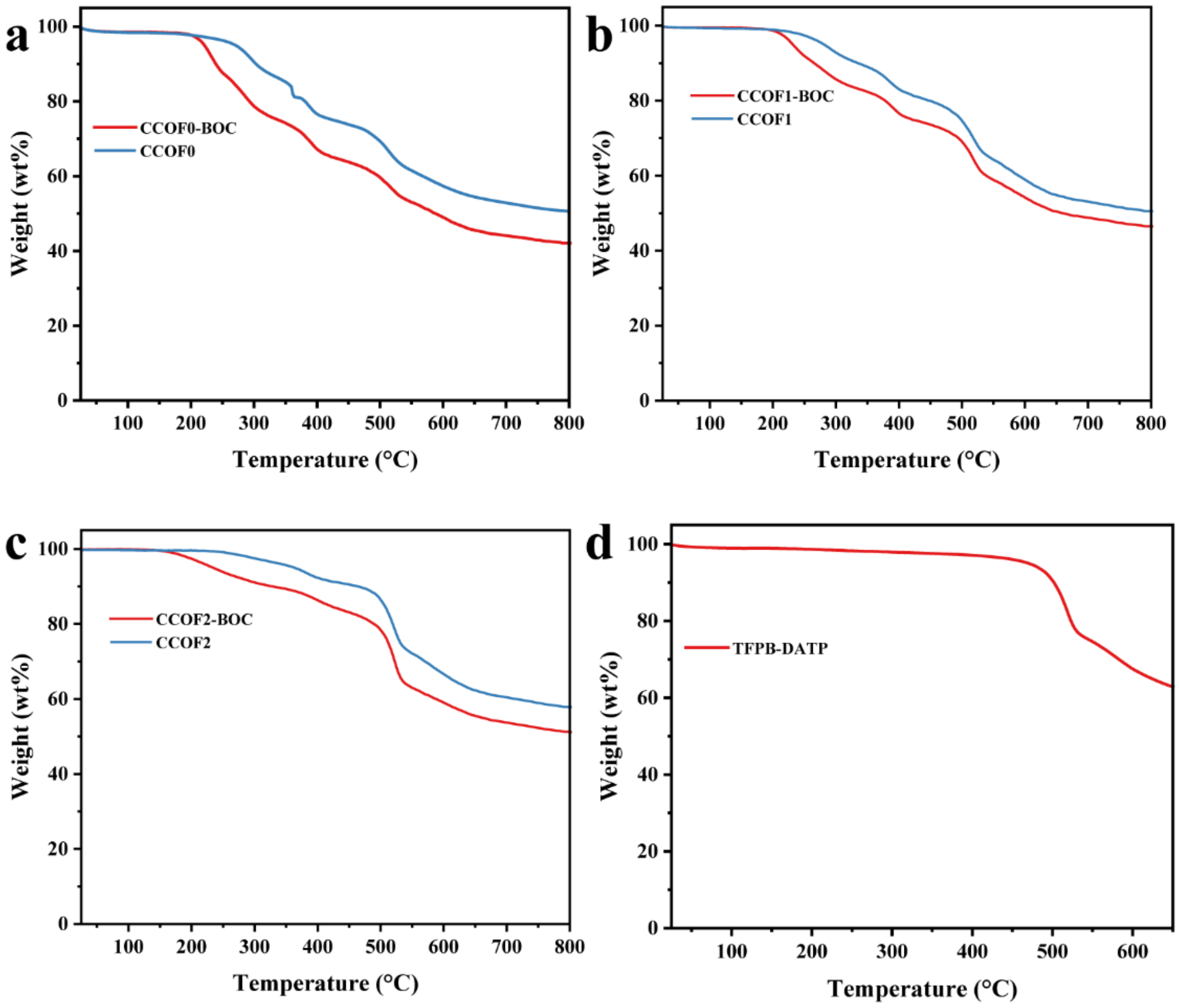

Supplementary Fig. 9 TGA curves of **a** CCOF0-Boc and CCOF0, **b** CCOF1-Boc and CCOF1, **c** CCOF2-Boc and CCOF2, and **d** TFPB-DATP. After thermal analysis, the residual samples in the crucibles transformed into black powders, likely due to the carbonization of the COFs at high temperature under nitrogen flow.

Comment 10: SEM and TEM images in the SI (Figures S5, S9, S10, and S13) are of limited quality and should be improved. Furthermore, TEM elemental mapping suggests an inhomogeneous distribution of elements, which contradicts the assumed uniformity of the materials;

Response: We sincerely appreciate the reviewer's rigorous evaluation of our electron microscopy data. We fully agree that the quality of the previous SEM and TEM images, as well as the elemental mapping, was sub-optimal and did not accurately reflect the true nature of our materials.

The apparent "inhomogeneous" distribution of Ru in the original TEM-EDS mapping was, in fact, an artifact caused by instrumental limitations and sample characteristics during the initial measurement. Because organic COF materials are highly sensitive to electron beam irradiation, we initially employed a very short acquisition time to prevent structural damage to the framework. Combined with the relatively low loading amount of Ru, this resulted in a very low signal-to-noise ratio (SNR). Consequently, the background noise dominated the signal, creating a false

visual impression of uneven elemental distribution. Furthermore, the absence of a corresponding HAADF-STEM (High-Angle Annular Dark-Field) image made it difficult to accurately correlate the elemental signals with the actual morphology.

To rigorously address this issue, we prepared fresh samples and re-conducted the electron microscopy characterizations. We first acquired new, high-resolution SEM and HRTEM images (now updated as Figures S8, S15, S16, and S25 in the revised SI), which clearly display the ordered morphology and lattice fringes of the COFs. More importantly, to accurately evaluate the metal dispersion, we performed new HAADF-STEM coupled with optimized EDS mapping. By carefully balancing the electron dose and extending the acquisition time, we obtained high-quality maps with a strong signal-to-noise ratio. The new results unambiguously demonstrate that Ru is homogeneously distributed throughout the entire COF matrix. Importantly, no aggregated Ru nanoparticles or clusters were observed, confirming the uniform coordination of Ru sites within the framework. We have updated all relevant figures in the Supporting Information and revised the main text to reflect these high-quality results.

Manuscript Change:

Manuscript (revised text on page 10, first paragraph):

"HRTEM demonstrated that the ordered pore architectures of CCOF1-Ru and CCOF2-Ru were well-maintained after Ru coordination. Furthermore, high-angle annular dark-field scanning transmission electron microscopy (HAADF-STEM) coupled with corresponding energy-dispersive X-ray spectroscopy (EDS) mapping unambiguously revealed a highly homogeneous distribution of Ru, C, N, and O elements throughout the COF matrix, with no observable metal nanoparticle aggregation (Supplementary Fig. 15). This confirms the uniform integration of the catalytic Ru sites within the framework."

Supporting Information (revised Supplementary Fig. 8, Supplementary Fig. 15, Supplementary Fig. 16 and Supplementary Fig. 25):

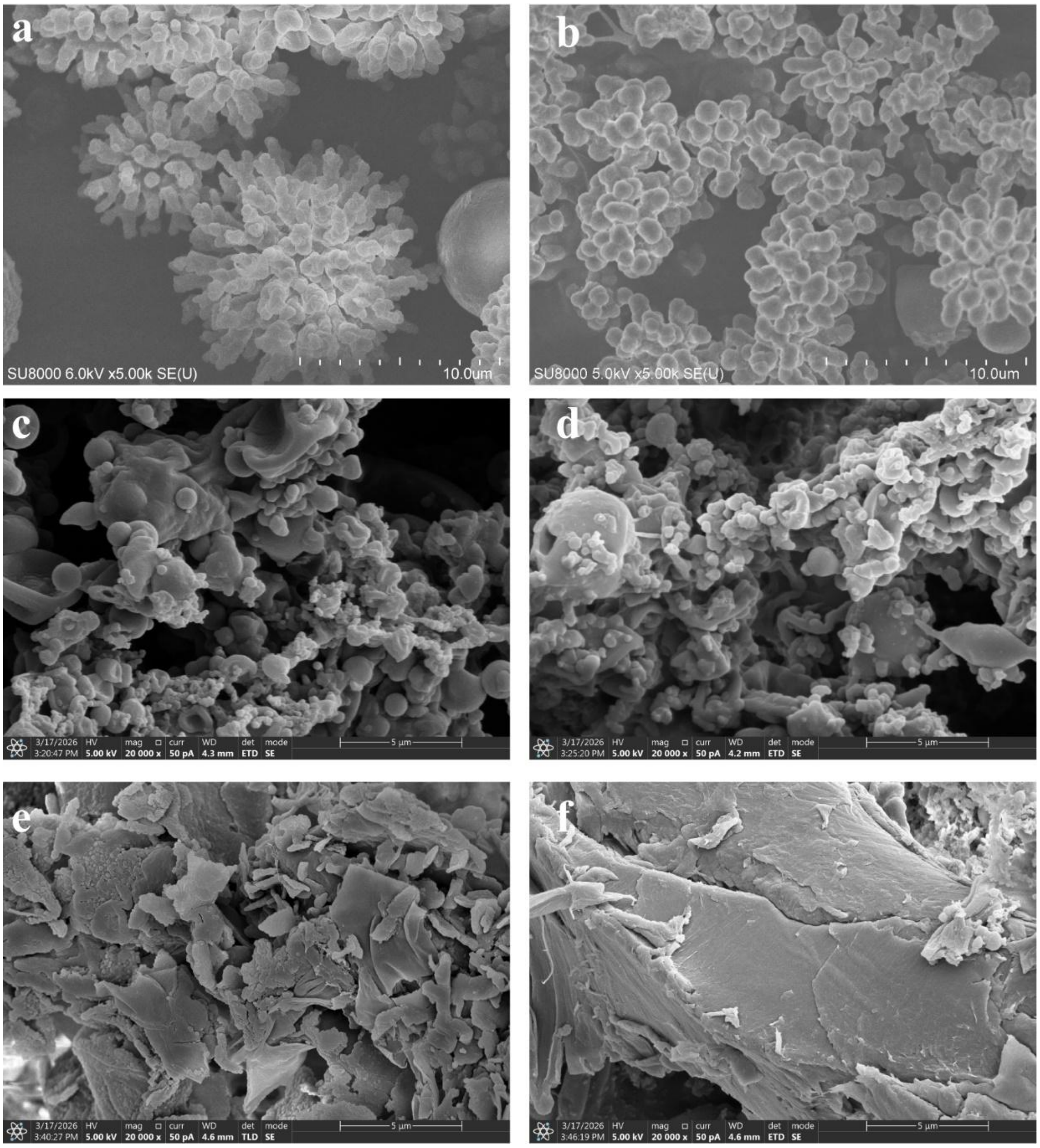

Supplementary Fig. 8 SEM images of **a** CCOF0-Boc, **b** CCOF0, **c** CCOF1-Boc, **d** CCOF1, **e** CCOF2-Boc and **f** CCOF2.

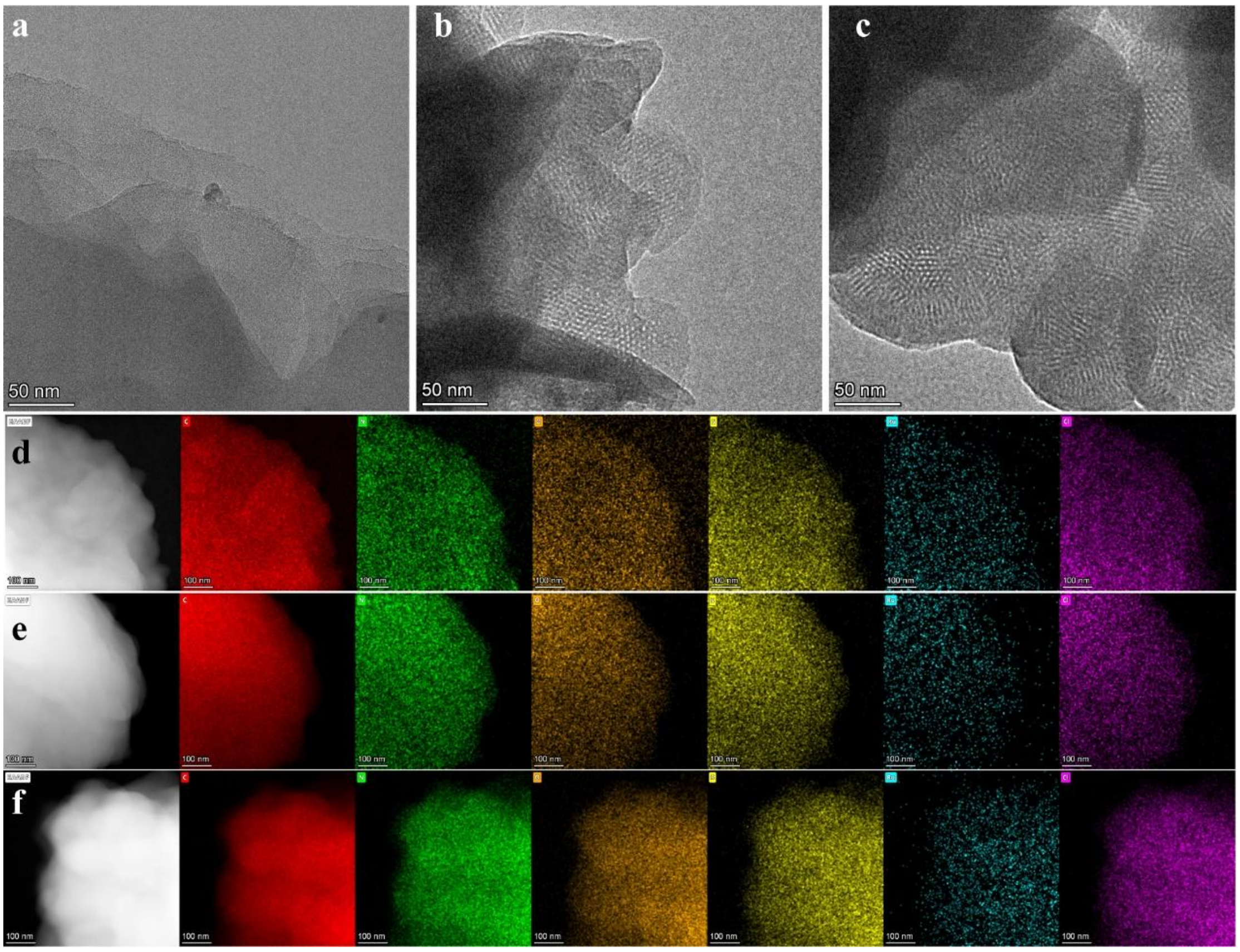

Supplementary Fig. 15 TEM image of a CCOF0-Ru, b CCOF1-Ru and c CCOF2-Ru. HAADF image and TEM-EDS mapping of d CCOF0-Ru, e CCOF1-Ru and f CCOF2-Ru.

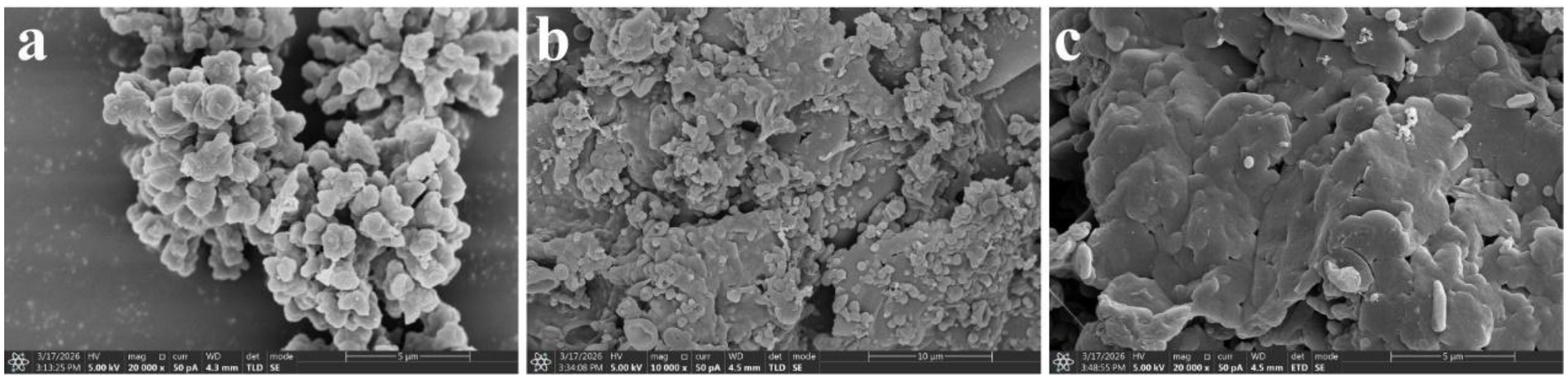

Supplementary Fig. 16 SEM image of a CCOF0-Ru, b CCOF1-Ru and c CCOF2-Ru.

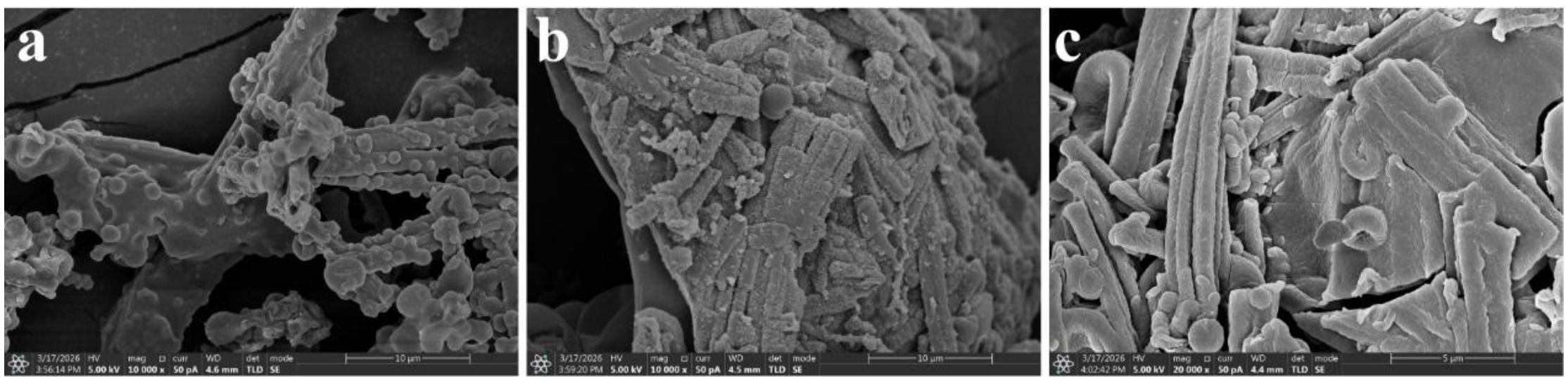

Supplementary Fig. 25 SEM image of **a** CCOF-DACH-Boc, **b** CCOF-DACH and **c** CCOF-DACH-Ru.

Comment 11: The proposed catalytic mechanism is largely speculative and not supported by experimental or computational evidence. Given the current level of structural and compositional uncertainty of the materials, such mechanistic proposals appear premature;

Response: We sincerely thank the reviewer for this constructive critique. We agree that a proposed mechanism must be grounded in solid experimental evidence. Building upon the structural foundation of the CCOF catalysts, we have performed a series of new mechanistic experiments—including control reactions, deuterium-labeling, and GC analysis—to provide robust experimental support for our proposed catalytic cycle.

The control experiments show that reducing the catalyst loading or sodium formate amounts leads to a progressive decrease in yield, suggesting sodium formate serves as the hydrogen source. Using isopropanol or acetonitrile as solvents resulted in significant yield reductions, indicating water may participate in the catalytic cycle. Furthermore, deuterium-labeling experiments confirmed a kinetic isotope effect of k_H/k_D = 3.0, which is in excellent agreement with the value reported for the homogeneous Noyori-type catalyst (k_H/k_D = 3.2, *Org. Biomol. Chem.* **2004**, *2*, 1818–1821). GC analysis confirmed the presence of H_2 and CO_2 post-reaction. Based on these new experimental results and literature precedents, we have revised the proposed reaction mechanism and added a robust discussion to the main text.

Manuscript Change:

Revised Manuscript (revised text on page 14, first paragraph):

"To further elucidate the mechanism of the asymmetric transfer hydrogenation of ketones catalyzed by CCOF2-Ru, we performed additional control and deuterium-labeling experiments. Reducing the catalyst loading leads to a decrease in yield (Supplementary Table 12, entry 8), and the yield progressively declines with decreasing amounts of sodium formate (Supplementary Table 12, entries 9–10), suggesting that sodium formate serves as the hydrogen source. When isopropanol was used as the solvent, both the yield and ee decreased significantly (Supplementary Table 12, entry 11, 41% yield, 86% ee). Using the polar aprotic solvent acetonitrile resulted in a marked reduction in yield (Supplementary Table 12, entry 12, 18% yield, 95% ee). These results indicate that water actively participates in the catalytic cycle.

The deuterium-labeling experiments further confirm the role of sodium formate. As shown in Supplementary Fig. 27, these experiments demonstrate that the hydrogen atoms originate predominantly from sodium formate, rather than from water or isopropanol. Furthermore, a kinetic isotope effect of k_H/k_D = 3.0 was determined using

a 1:1 molar mixture of DCO_2Na and HCO_2Na (conversion <10%, product deuteration level of 25%; Supplementary Fig. 27-28), which is in excellent agreement with the value reported for the homogeneous Noyori-type catalyst (k_H/k_D = 3.2).[85] These findings suggest that C–H bond cleavage of the formate complex may be the rate-determining step. Furthermore, GC analysis of the reaction tube confirmed the presence of H_2 and CO_2. The formation of CO_2 can be attributed to the β-hydride elimination of the CCOF2-Ru formate complex, which simultaneously generates the active Ru–H intermediate. Meanwhile, the evolution of H_2 is likely associated with the Ru–H intermediate releasing H_2 upon interaction with H^+ from water.

Based on our experimental results and the reported mechanisms of Noyori-type catalysts, we propose a plausible reaction mechanism (Supplementary Fig. 29).[85–89] Upon treatment with HCOONa, CCOF2-Ru undergoes ion exchange to generate the Ru–formate complex (CCOF2-Ru **I**). Subsequent β-hydride elimination affords the catalytically active Ru–H intermediate (CCOF2-Ru **II**). Stereoselective hydride transfer to the prochiral ketone substrate takes place within the confined chiral microenvironment of CCOF2-Ru, producing the Ru–diamine species (CCOF2-Ru **III**). In the presence of sodium formate and H_2O, CCOF2-Ru **III** is converted back to CCOF2-Ru **I**, thereby entering the next catalytic cycle."

Manuscript Change:

Supporting Information (added figures on page 52, Supplementary Fig. 27-28; revised figure on page 53, Supplementary Fig. 29; revised table on page 82, Supplementary Table 12):

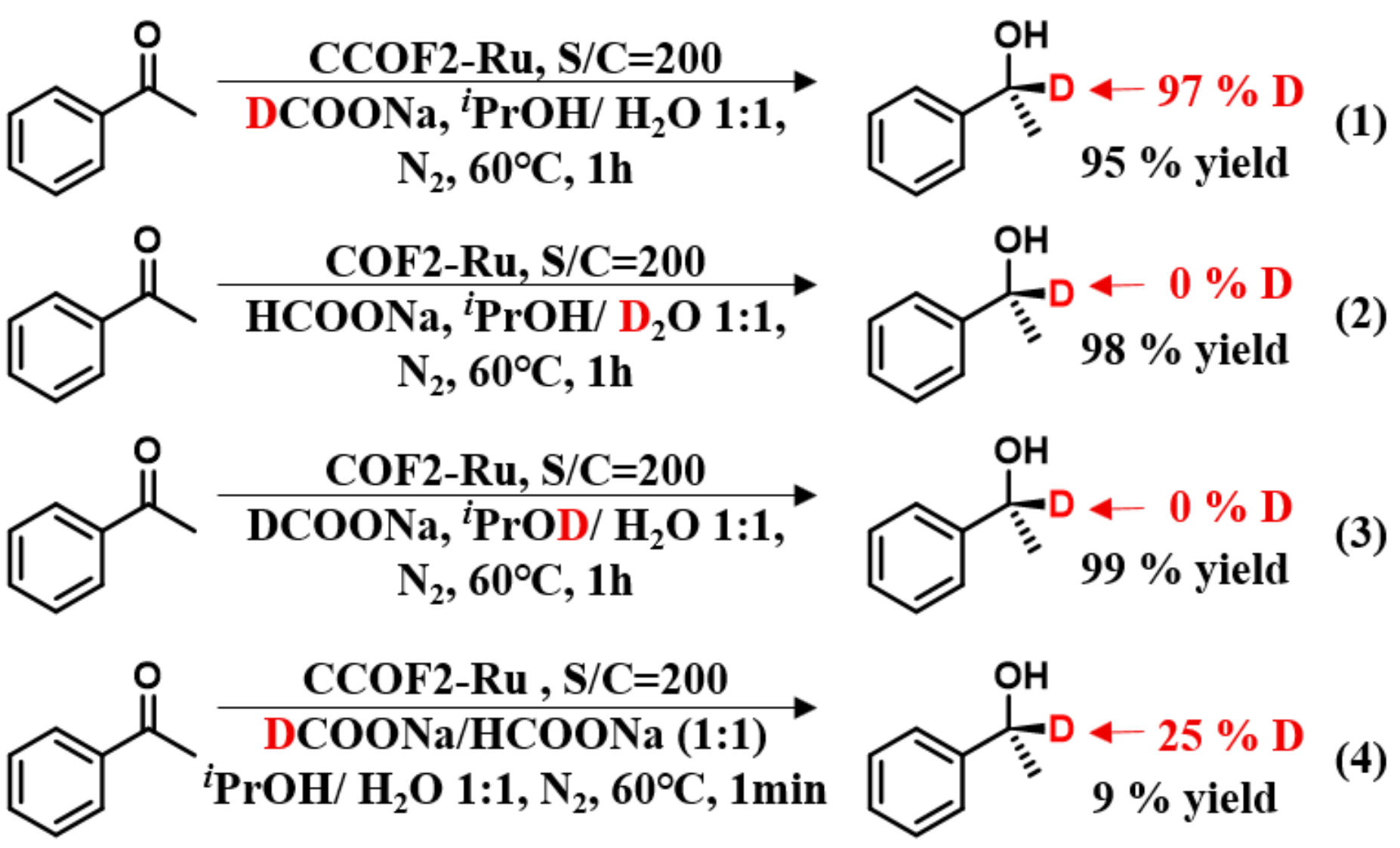

Supplementary Fig. 27 Deuterium-labeling studies.

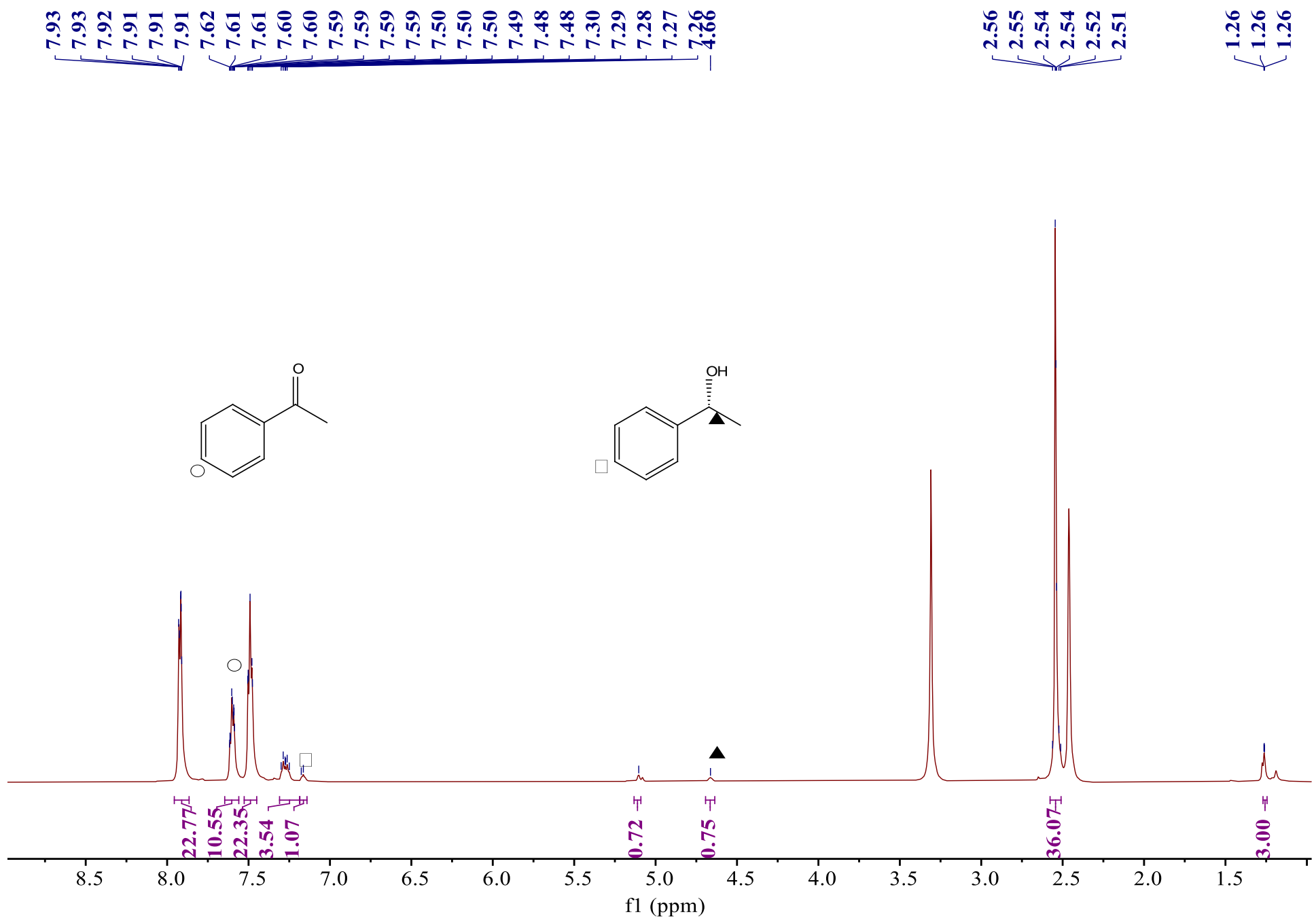

Supplementary Fig. 28 ^{1}H NMR (600 MHz, DMSO-D6) spectra of the deuterium-labeling KIE studies. At a conversion of <10%, the deuterium incorporation in the product was determined to be 25%, corresponding to a kinetic isotope effect of $k_H/k_D \approx 3.0$.

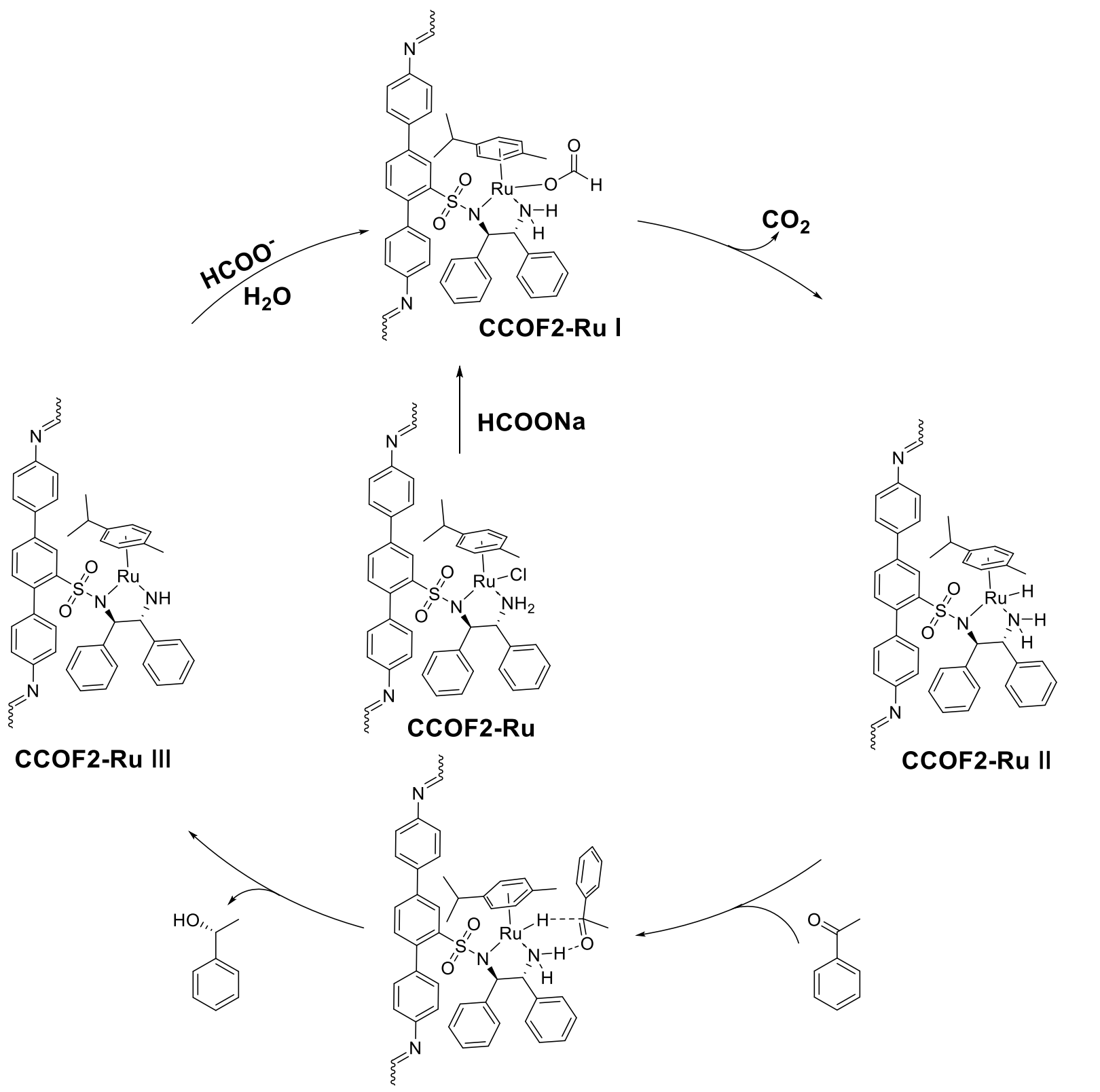

Supplementary Fig. 29 Plausible reaction mechanism for COF2-Ru catalyzed asymmetric transfer hydrogenation of acetophenone.

Supplementary Table 12. Optimization of the reaction conditions and control experiments in asymmetric transfer hydrogenation of acetophenone.

Entry	Catalyst	t (h)	T (°C)	Addition	Solvent	Yield (%)	ee [a] (%)
1	CCOF2-Ru	1	60	HCOONa	1mL ^{i}PrOH/ H_2O (1:1)	>99	95
2	CCOF2-Ru	1	60	HCOONa	1mL H_2O	98	90
3	CCOF2-Ru	1	60	$HCOONH_4$	1mL ^{i}PrOH/ H_2O (1:1)	21	90
4	CCOF2-Ru	1	60	KOH	1mL ^{i}PrOH	65	50
5	CCOF2-amp-Ru	1	60	HCOONa	1mL ^{i}PrOH/ H_2O (1:1)	85	87
6	CCOF2	1	60	HCOONa	1mL ^{i}PrOH/ H_2O (1:1)	n.d. [b]	-
7	TFPB-DATP-Ru	1	60	HCOONa	1mL ^{i}PrOH/ H_2O (1:1)	n.d.	-
8	CCOF2-Ru (0.25%) [c]	1	60	HCOONa	1mL ^{i}PrOH/ H_2O (1:1)	75	95
9	CCOF2-Ru	1	60	HCOONa (2 equiv.)	1mL ^{i}PrOH/ H_2O (1:1)	90	95
10	CCOF2-Ru	1	60	HCOONa (1 equiv.)	1mL ^{i}PrOH/ H_2O (1:1)	67	94
11	CCOF2-Ru	1	60	HCOONa	1mL ^{i}PrOH	41	86
12	CCOF2-Ru	1	60	HCOONa	1mL ACN	18	95

Reaction conditions: ketones (0.2 mmol), addition (1 mmol), catalyst (0.5 mol % of Ru active site based on ICP analysis) under N_2 atmosphere in 1 mL solvent. [a] Determined by chiral HPLC. [b] n.d. = No detection. [c] 0.25 mol % of Ru active site based on ICP analysis.

Reference:

85. Wu X, Li X, Hems W, King F, Xiao J. Accelerated asymmetric transfer hydrogenation of aromatic ketones in water. *Org. Biomol. Chem.* **2**, 1818-1821 (2004).

Comment 12: The presentation of the Supporting Information also requires attention:

figure sizes are highly inconsistent, with some non-essential figures being overly large, while key data are difficult to read due to small size or poor resolution;

Response: We appreciate the reviewer's valuable suggestion. We have adjusted the figure sizes throughout the Supporting Information, revised the overall layout for consistency, and replaced all unclear images, including the SEM and TEM micrographs, to ensure high resolution and readability.

Comment 13: Several specific points require clarification: The rationale for incorporating Rh and Ir into the same materials should be clearly explained.

Response: We sincerely thank the reviewer for highlighting the need to clarify this important point. The incorporation of Rh and Ir alongside Ru was not a random selection, but a deliberate design strategy rooted in both catalytic optimization and materials science innovation. In the field of Noyori-type asymmetric transfer hydrogenation (ATH), complexes based on Ru, Rh, and Ir represent the three most classic and effective catalytic centers. Because the nature of the metal center dictates the hydride transfer kinetics, the metal-ligand bond lengths, and the resulting chiral microenvironment, screening this specific triad is essential to systematically evaluate the structure-activity relationships and identify the optimal metal center for our specific CCOF system. Furthermore, from a materials design perspective, we aimed to demonstrate that CCOF2 is not merely a single-use support for Ru, but a highly robust and modular "macromolecular chiral ligand" platform. Successfully coordinating different metal precursors—such as $[Ru(\eta^6\text{-}C_6H_6)Cl_2]_2$, $[CpRhCl_2]_2$, and $[CpIrCl_2]_2$—while retaining the framework's crystallinity proves the broad compatibility and structural versatility of our synthetic strategy. We have expanded the discussion in the revised manuscript to explicitly highlight this rationale.

Manuscript Change:

Manuscript (revised text on page 12, first paragraph):

"To evaluate the generality of CCOF2 as a versatile chiral platform and to systematically investigate the influence of different metal centers on the catalytic performance, we extended our post-synthetic metalation strategy to include other classic Noyori-type metal precursors. Specifically, we attempted the coordination of $[Ru(\eta^6\text{-}C_6H_6)Cl_2]_2$, $[CpRhCl_2]_2$, and $[CpIrCl_2]_2$ onto the CCOF2 framework. The results reveal that this multivariate assembly exhibits excellent modularity: CCOF2 can uniformly stabilize Ru, Rh, and Ir metal centers via an identical protocol while fully retaining its crystalline architecture (PXRD, Supplementary Fig. 20). Among these isostructural metallo-COFs, CCOF2-Ru displays the highest catalytic activity and

enantioselectivity for asymmetric transfer hydrogenation (Fig. 5b), highlighting the critical role of metal selection in optimizing the confined chiral microenvironment."

Comment 14: The nature of TFPB-DATP-Ru needs to be defined: how is Ru coordinated to the DATP ligand, and how has this been experimentally confirmed;

Response: We sincerely appreciate the reviewer's insightful comment. Defining the exact nature and coordination environment of the control material, TFPB-DATP-Ru, is crucial for understanding the structure-activity relationship and highlighting the unique design advantages of our primary CCOF catalysts. To address this, we have systematically investigated TFPB-DATP-Ru using a combination of analytical techniques.

First, ICP-OES analysis reveals that the Ru loading in TFPB-DATP-Ru is extremely low (0.17 wt%), standing in stark contrast to the high Ru loading achieved in CCOF2. This significant difference strongly demonstrates that without the specifically designed chiral diamine docking sites, the regular imine-linked COF backbone (TFPB-DATP) possesses very poor affinity for Ru coordination, thereby indirectly validating the necessity and innovation of our targeted pocket design in CCOF2. Regarding the physical state of these trace metals, the PXRD pattern of TFPB-DATP-Ru (Supplementary Fig. 21a) retains the original framework crystallinity without any characteristic peaks of Ru clusters or oxides. Combined with FTIR spectra (Supplementary Fig. 21b) showing no structural degradation, this indicates that the trace amount of Ru is highly dispersed rather than aggregated into nanoparticles. Furthermore, high-resolution XPS provided critical insights into the exact coordination mode. Despite the low Ru content, we observed a distinct shift in the binding energy of the imine C=N peak in the N 1s XPS spectrum (Supplementary Fig. 22). This provides direct spectroscopic evidence that the highly dispersed trace Ru species coordinate with the imine nitrogen atoms on the COF backbone.

In summary, this non-specific, low-efficiency coordination perfectly contrasts with the robust, high-loading bidentate coordination within the chiral pockets of CCOF2, further underscoring the success of our structural design.

Manuscript Change:

Manuscript (revised text on page 11, second paragraph):

"To understand the specific role of the chiral diamine pockets, we investigated the coordination behavior of the control framework, TFPB-DATP (Supplementary information Section 2.9; Supplementary Fig. 21-22). ICP-OES revealed a remarkably low Ru loading of only 0.17 wt% in TFPB-DATP-Ru, indicating that the pristine imine

backbone lacks strong coordination sites for the metal precursor. High-resolution N 1s XPS analysis of TFPB-DATP-Ru (Supplementary Fig. 22) displayed a binding energy shift for the imine C=N moiety, suggesting that the trace Ru species merely coordinate to the backbone imine nitrogens. This weak and non-specific coordination sharply contrasts with the robust metalation achieved in CCOF2, thereby confirming the necessity of the engineered chiral microenvironment for efficient catalyst loading."

Supporting Information (page 48-49):

We have added Supplementary Fig. 21 (PXRD and FTIR) and Supplementary Fig. 22 (XPS spectra) to the SI, along with a discussion noting the shift in the imine C=N peak indicative of Ru coordination.

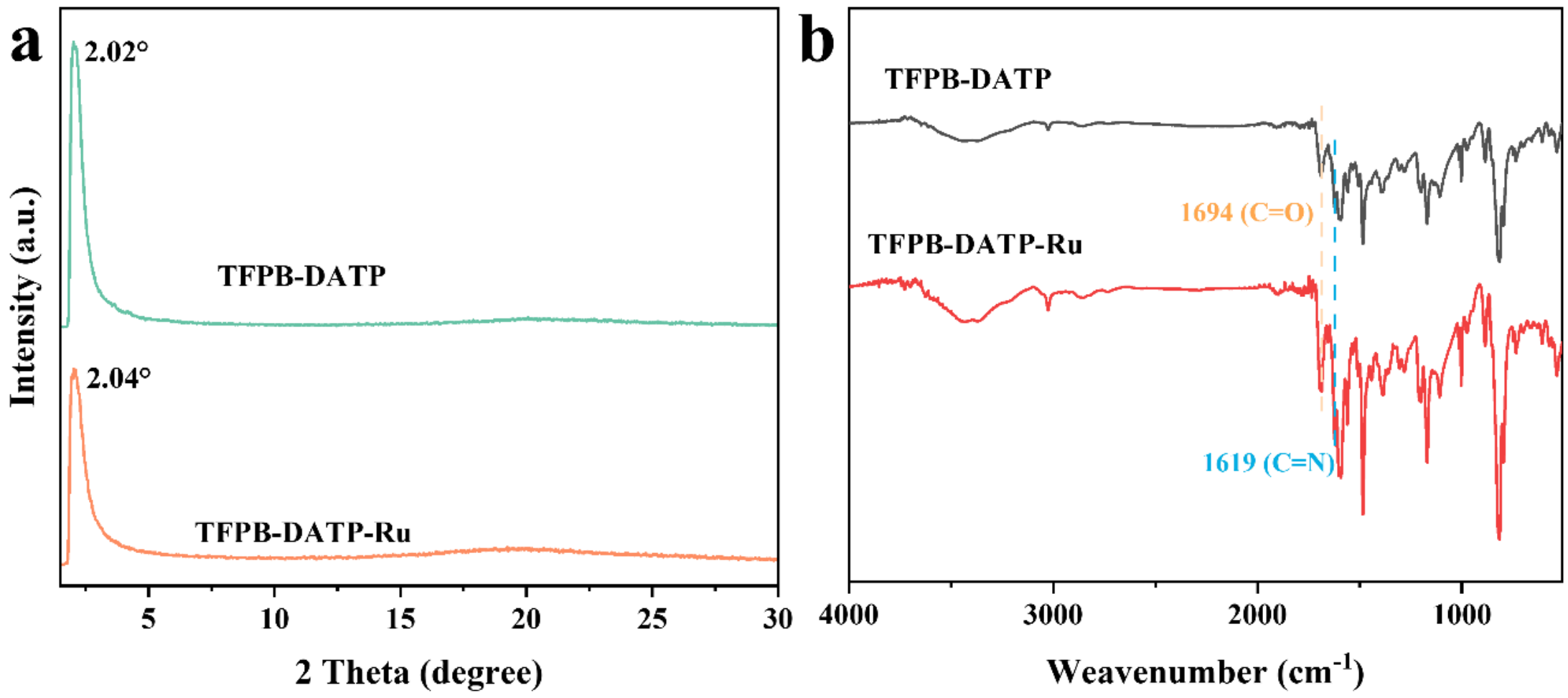

Supplementary Fig. 21 **a** PXRD patterns and **b** FTIR spectra of TFPB-DATP and TFPB-DATP-Ru. Supplementary Fig. 21 shows the PXRD patterns and FTIR spectra of TFPB-DATP before and after Ru coordination. The absence of Ru cluster or oxide peaks in PXRD confirms the high dispersion of the trace Ru species and the preservation of framework crystallinity.

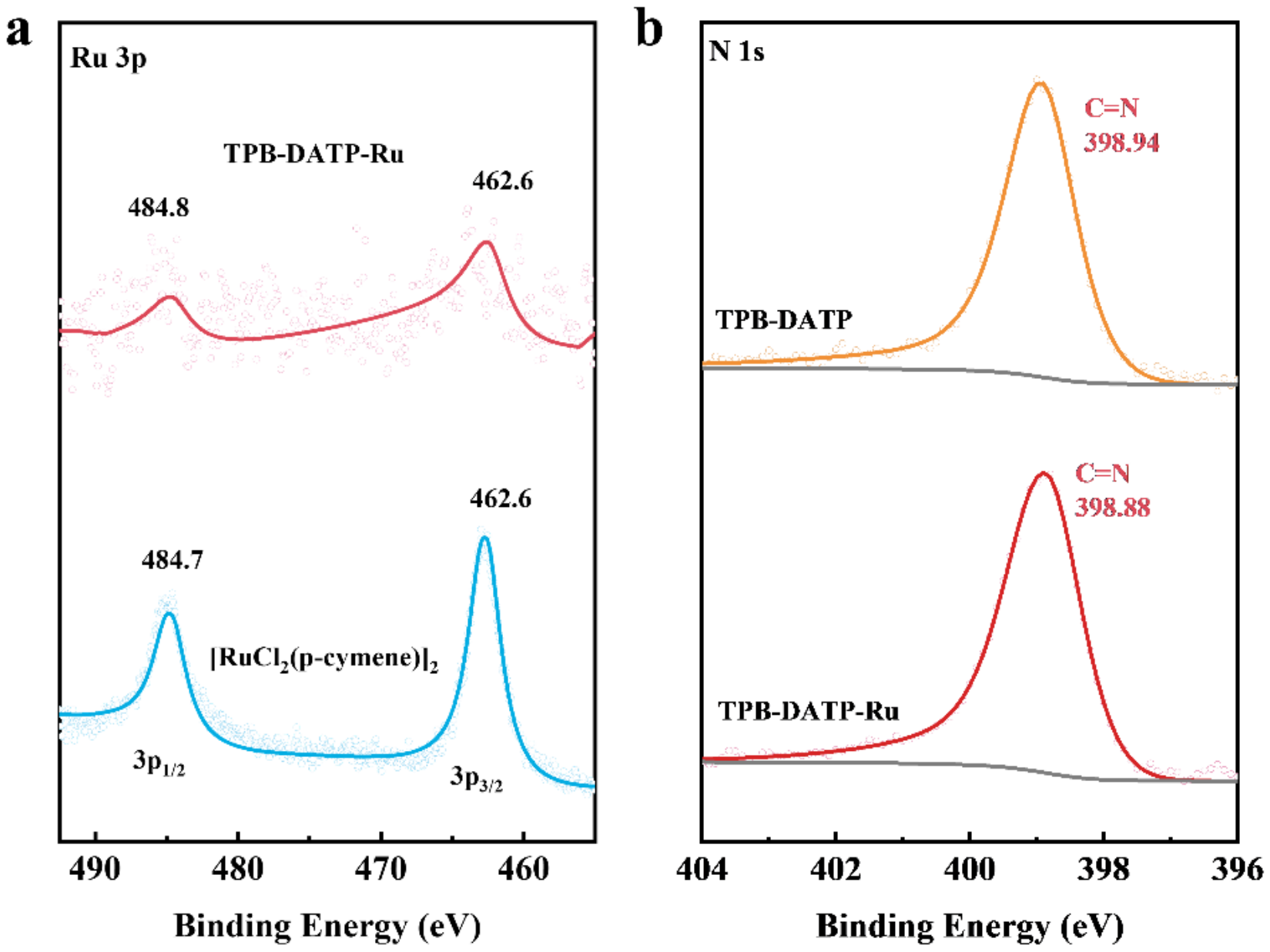

Supplementary Fig. 22 XPS spectra: a Ru 3p, b N 1s for TPB-DATP-Ru. A slight shift of the imine C=N peak is observed in the N 1s spectrum of TPB-DATP-Ru, indicating the possible coordination of Ru to C=N.

Comment 15: The identity and characterization of (R)-Ph-Ru should be clarified;

Response: We thank the reviewer for your careful review. We have supplemented the detailed synthesis procedure and full characterization (including ^{1}H NMR and HRMS) of the (*R*)-Ph-Ru (renamed as (*R*,*R*)-TP-DPEN-Ru) homogeneous model complex in the Supporting Information.

Manuscript Change:

Supporting Information (Page 20, Section 2.5):

2.5. Synthesis of (*R*,*R*)-TP-DPEN-Ru:

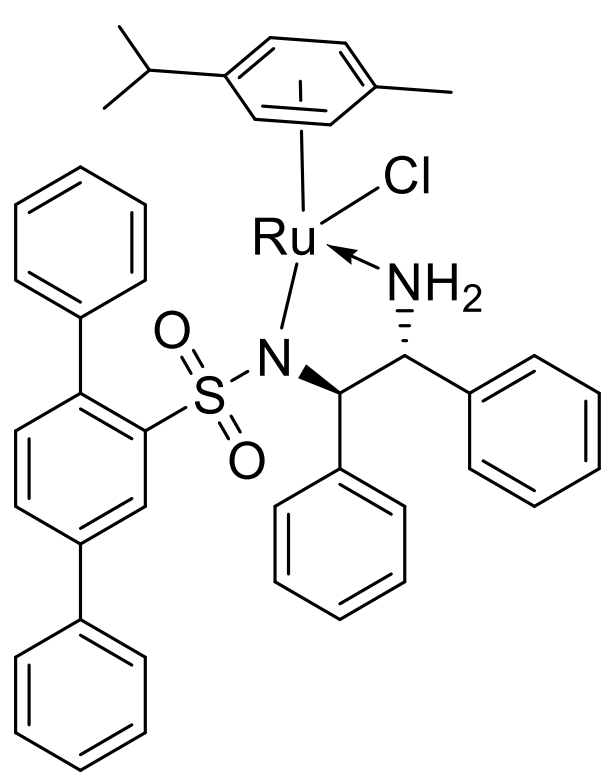

A mixture of [Ru(p-cymene)Cl_2]$_2$ (153 mg, 0.25 mmol), (*R*,*R*)-TP-DPEN (504 mg, 1

mmol), and TEA (0.14 mL, 1 mmol) in CH_2Cl_2 (10 mL) was stirred at room temperature for 1 h under a N_2 atmosphere. The mixture was then washed with water and dried over anhydrous Na_2SO_4. Removal of the solvent under reduced pressure afforded the crude product. Purification by flash column chromatography on silica gel using dichloromethane/methanol (100:1, v/v) as the eluent gave the pure product as an orange-yellow powder (264 mg, 85% yield). ^{1}H NMR (600 MHz, ACETONITRILE-*D*$_3$) δ 8.04 – 7.94 (s, 1H), 7.66 – 7.45 (m, 6H), 7.44 – 7.31 (m, 6H), 7.10 – 7.04 (m, 3H), 6.90 – 6.84 (d, *J* = 7.3 Hz, 3H), 6.67 – 6.57 (s, 5H), 6.01 – 5.92 (s, 1H), 5.57 – 5.52 (s, 1H), 5.17 – 5.09 (s, 1H), 5.02 – 4.95 (s, 1H), 3.90 – 3.81 (d, *J* = 11.2 Hz, 1H), 3.75 – 3.68 (t, *J* = 12.6 Hz, 1H), 3.45 – 3.34 (t, *J* = 11.8 Hz, 1H), 3.01 – 2.93 (s, 1H), 2.27 – 2.23 (s, 3H), 1.34 – 1.27 (dd, *J* = 20.0, 7.0 Hz, 6H). ESI-HRMS: Found: m/z 739.1912. Calcd for $C_{42}H_{41}N_2O_2RuS$: $(M-Cl)^+$ 739.1927.

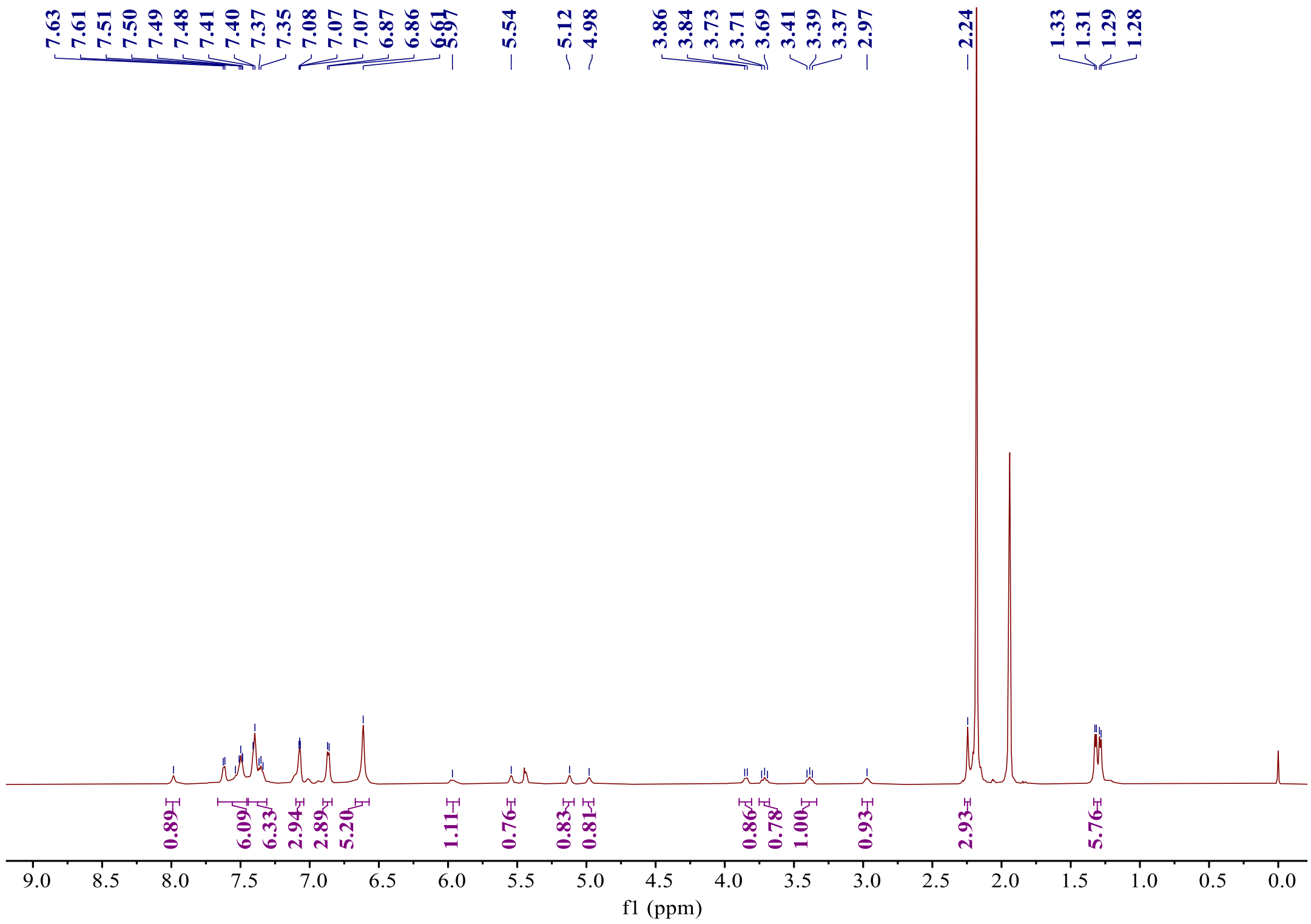

Comment 16: For the substrate scope shown in Figure 5e, the origin of the racemic substrates must be specified (commercial vs synthesized). All catalytic products should be appropriately characterized: new compounds require full spectroscopic characterization, while known compounds should be supported by 1H NMR data and proper literature references;

Response: We thank the reviewer for your careful review. The racemic substrates used as standards for chiral HPLC analysis were prepared by the reduction of the

corresponding commercial ketones with $NaBH_4$ in methanol. Their structures were verified by NMR spectroscopy. We have supplemented the synthetic procedures for these racemic samples, as well as the ^{1}H NMR data and relevant literature references for all known catalytic products, in the Supporting Information.

Manuscript Change:

Supporting Information (revised text and added NMR spectra on pages 86-116, Section 5):

Section 5. HPLC Chromatograms and NMR Spectra of the Catalytic Products

All racemic standards used for chiral HPLC analysis were prepared by reduction of the corresponding ketone precursors with $NaBH_4$ in methanol, and their structures were confirmed by NMR spectroscopy.

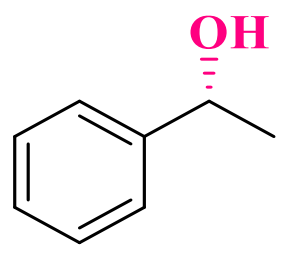

(*R*)-(+)-1-Phenylethanol (1a). Known compound, see: *Chem.* 10, 250-264. (2024).

The product **1a** was purified by flash chromatography on silica gel (300-400 mesh). ^{1}H NMR (600 MHz, DMSO-D_6) δ 7.36 – 7.28 (dtd, J = 15.4, 7.4, 1.8 Hz, 4H), 7.23 – 7.18 (m, 1H), 5.16 – 5.12 (dd, J = 4.2, 1.6 Hz, 1H), 4.73 – 4.67 (qd, J = 6.5, 4.6 Hz, 1H), 1.33 – 1.30 (dd, J = 6.5, 1.6 Hz, 3H).

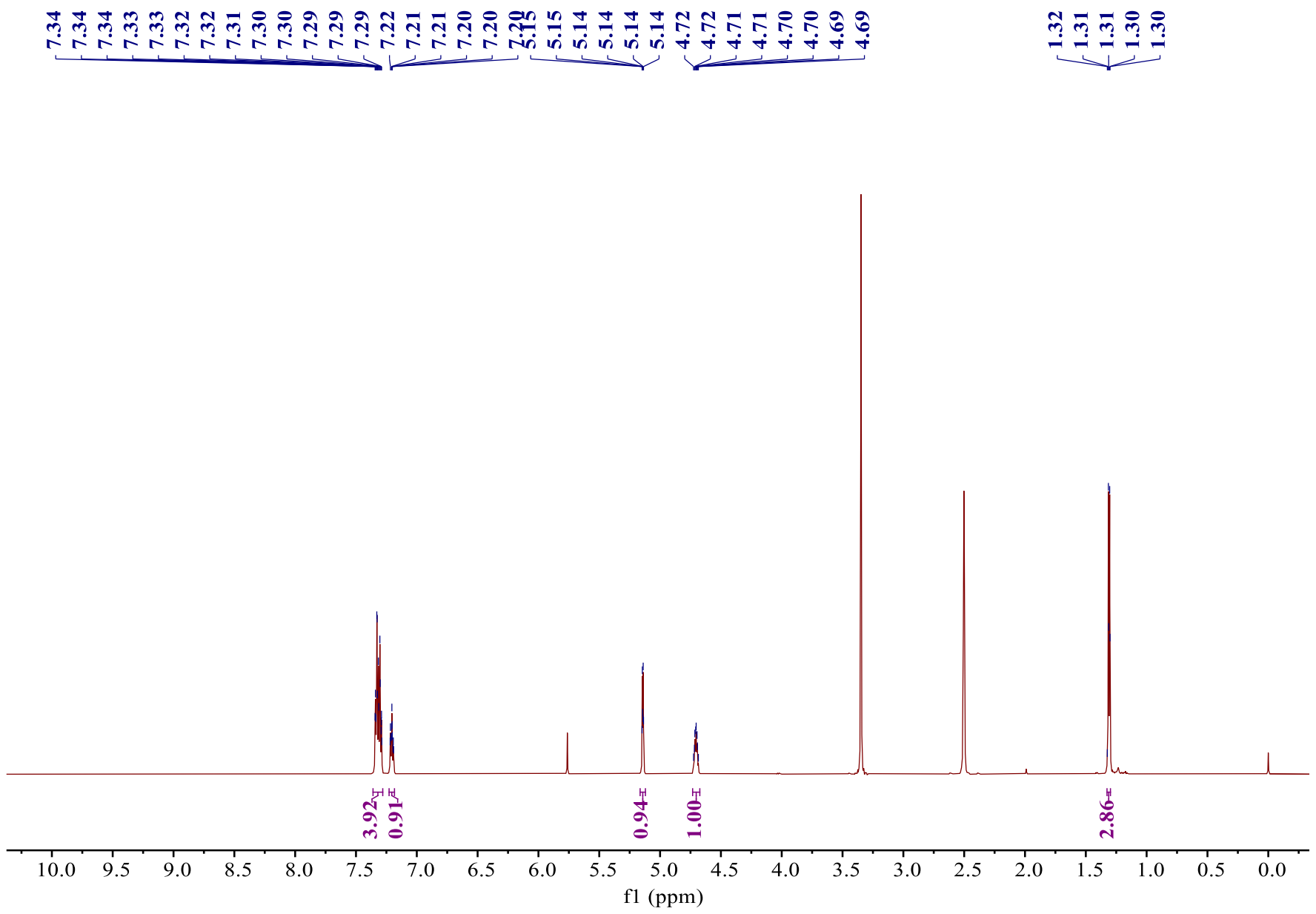

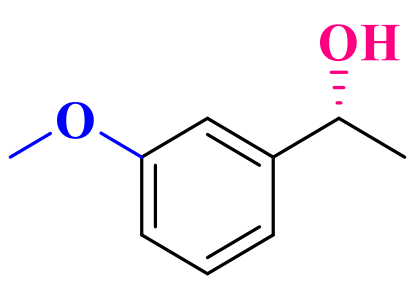

(*R*)-1-(3-methoxyphenyl)ethan-1-ol (1b). Known compound, see: *J. Organomet. Chem.* 346, 413-424, (1988).

The product **1b** was purified by flash chromatography on silica gel (300-400 mesh). ^{1}H NMR (600 MHz, CHLOROFORM-*D*) δ 7.29 – 7.23 (t, *J* = 8.1 Hz, 1H), 6.97 – 6.91 (m, 2H), 6.84 – 6.78 (ddd, *J* = 8.3, 2.5, 1.1 Hz, 1H), 4.89 – 4.83 (q, *J* = 6.5 Hz, 1H), 3.82 – 3.79 (s, 3H), 2.01 – 1.99 (s, 1H), 1.49 – 1.46 (d, *J* = 6.5 Hz, 3H).

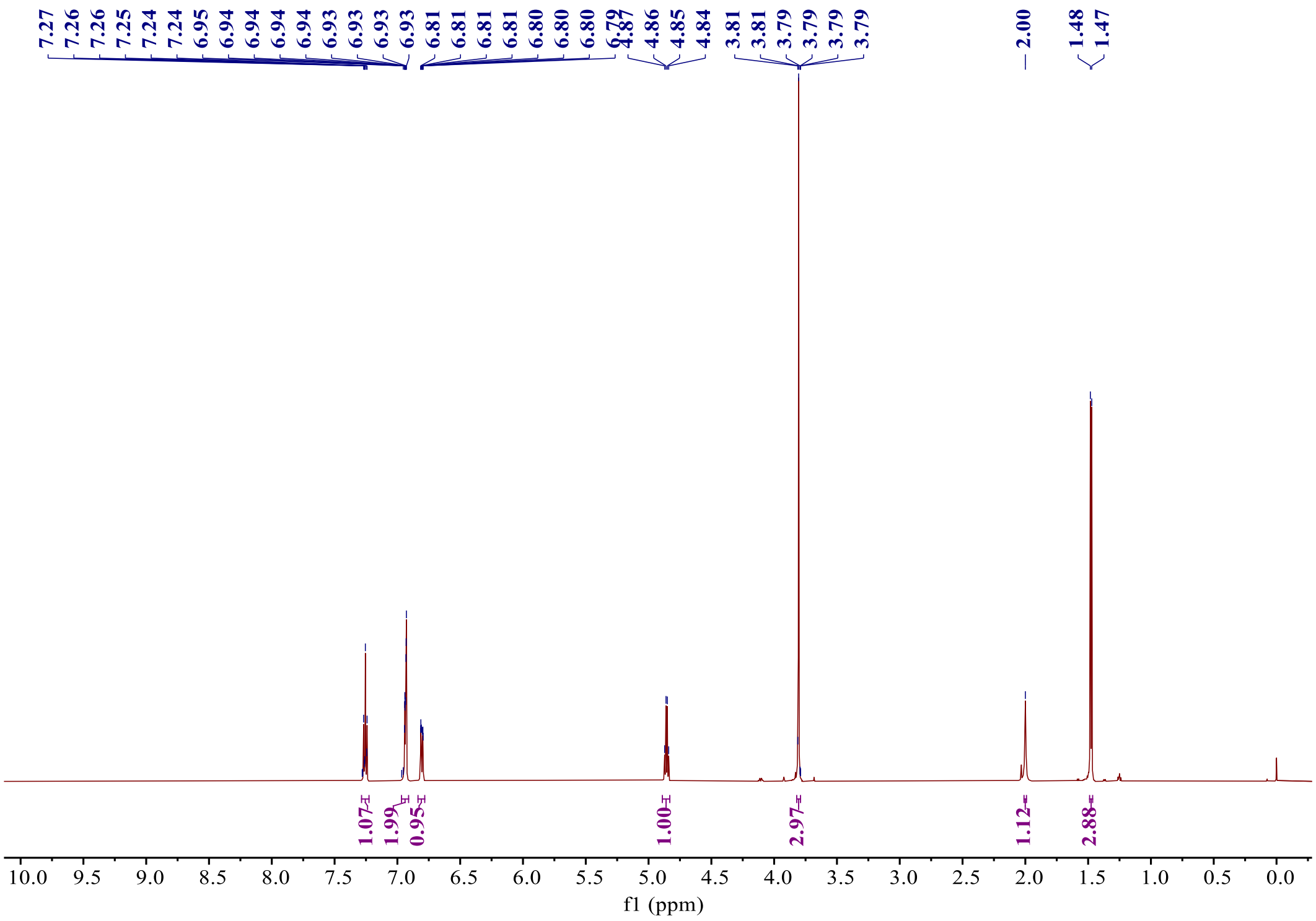

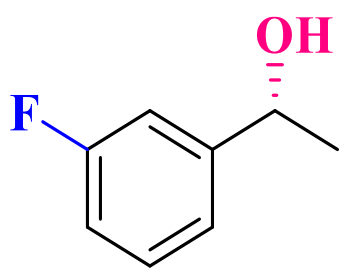

(*R*)-1-(3-fluorophenyl)ethan-1-ol (1c). Known compound, see: *Bioorg. Chem.* 16, 388-393, (1988).

The product **1c** was purified by flash chromatography on silica gel (300-400 mesh). ^{1}H NMR (600 MHz, CHLOROFORM-*D*) δ 7.32 – 7.28 (td, *J* = 7.9, 5.8 Hz, 1H), 7.14 – 7.11 (ddt, *J* = 7.7, 1.6, 0.8 Hz, 1H), 7.11 – 7.08 (dt, *J* = 9.9, 2.2 Hz, 1H), 6.97 – 6.93 (tdd, *J* = 8.4, 2.6, 1.0 Hz, 1H), 4.91 – 4.87 (q, *J* = 6.5 Hz, 1H), 1.98 – 1.95 (s, 1H), 1.49 – 1.47 (d, *J* = 6.5 Hz, 3H).

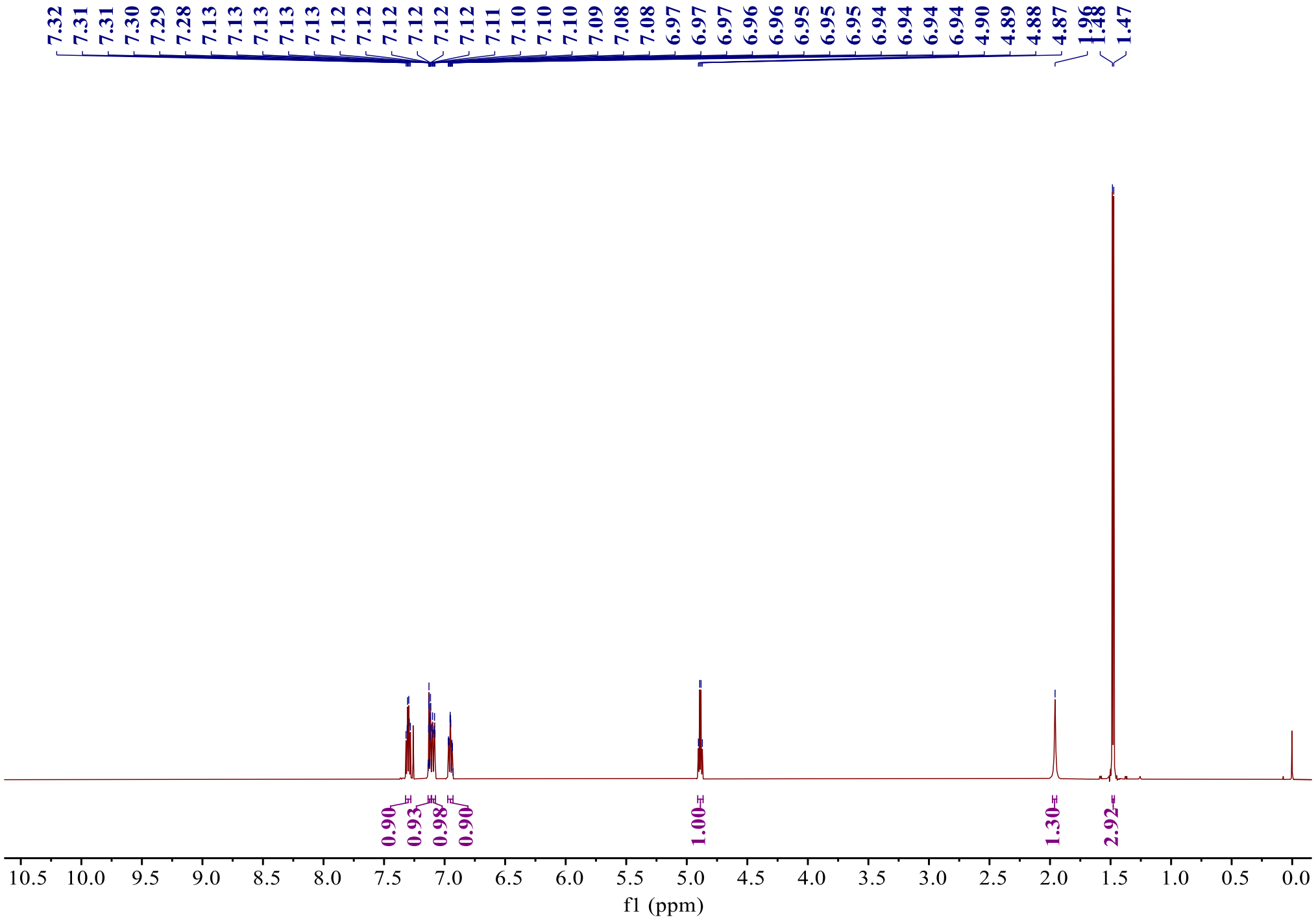

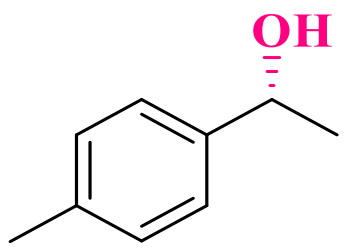

(*R*)-1-(*p*-tolyl)ethan-1-ol (1d). Known compound, see: *J. Org. Chem.* 68, 402-406, (2003).

The product **1d** was purified by flash chromatography on silica gel (300-400 mesh). ^{1}H NMR (600 MHz, CHLOROFORM-*D*) δ 7.27 – 7.24 (m, 2H), 7.17 – 7.14 (d, *J* = 7.8 Hz, 2H), 4.88 – 4.83 (q, *J* = 6.5 Hz, 1H), 2.35 – 2.33 (s, 3H), 1.87 – 1.83 (s, 1H), 1.49 – 1.46 (d, *J* = 6.5 Hz, 3H).

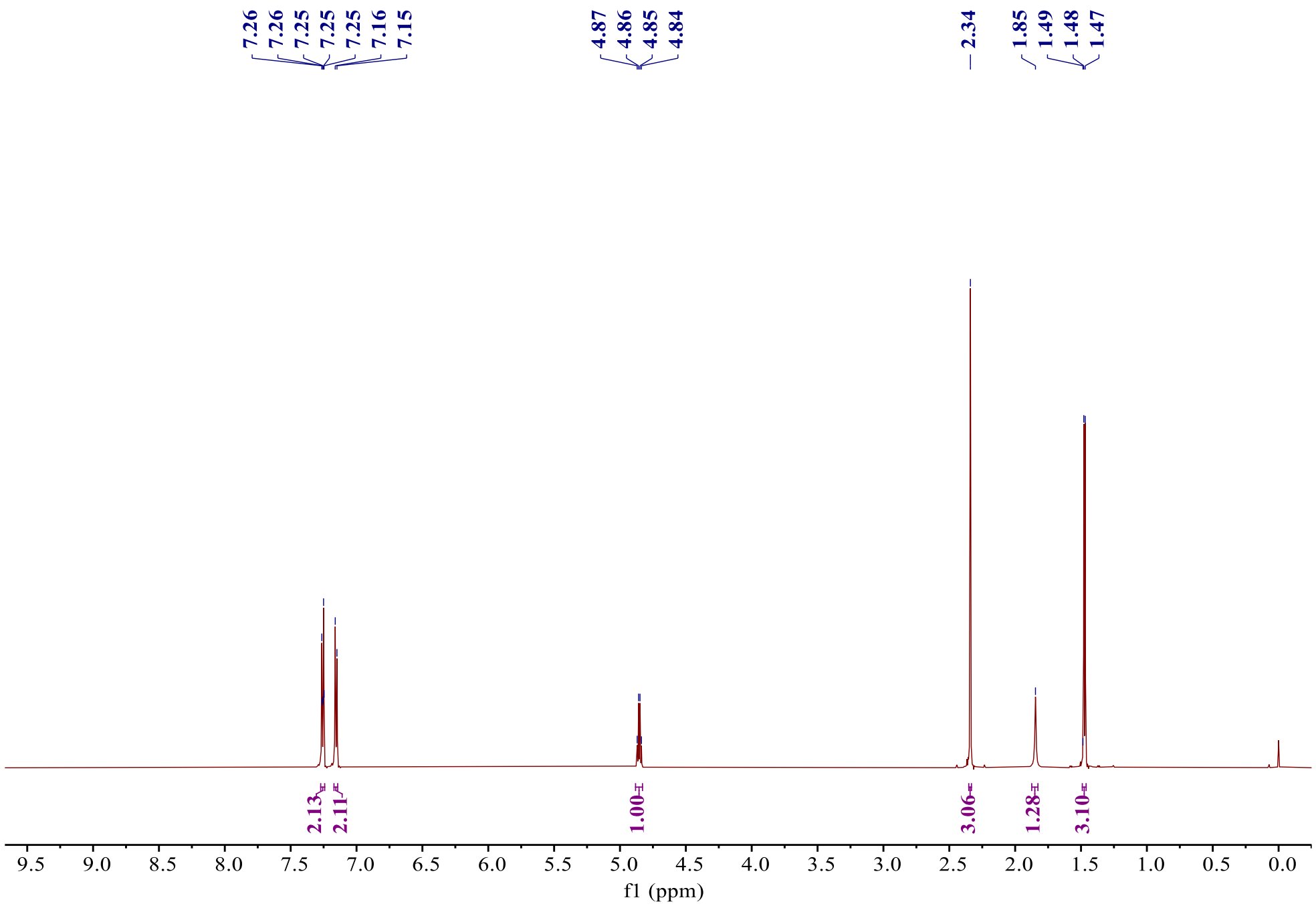

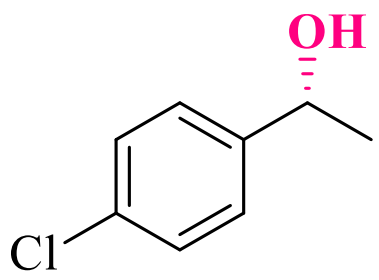

(*R*)-1-(4-chlorophenyl)ethan-1-ol (1e). Known compound, see: *Chem.* 10, 250-264. (2024).

The product **1a** was purified by flash chromatography on silica gel (300-400 mesh). ^{1}H NMR (600 MHz, DMSO-D_6) δ 7.38 – 7.35 (s, 4H), 5.28 – 5.25 (d, *J* = 4.3 Hz, 1H), 4.75 – 4.70 (qd, *J* = 6.5, 4.2 Hz, 1H), 1.32 – 1.30 (d, *J* = 6.5 Hz, 3H).

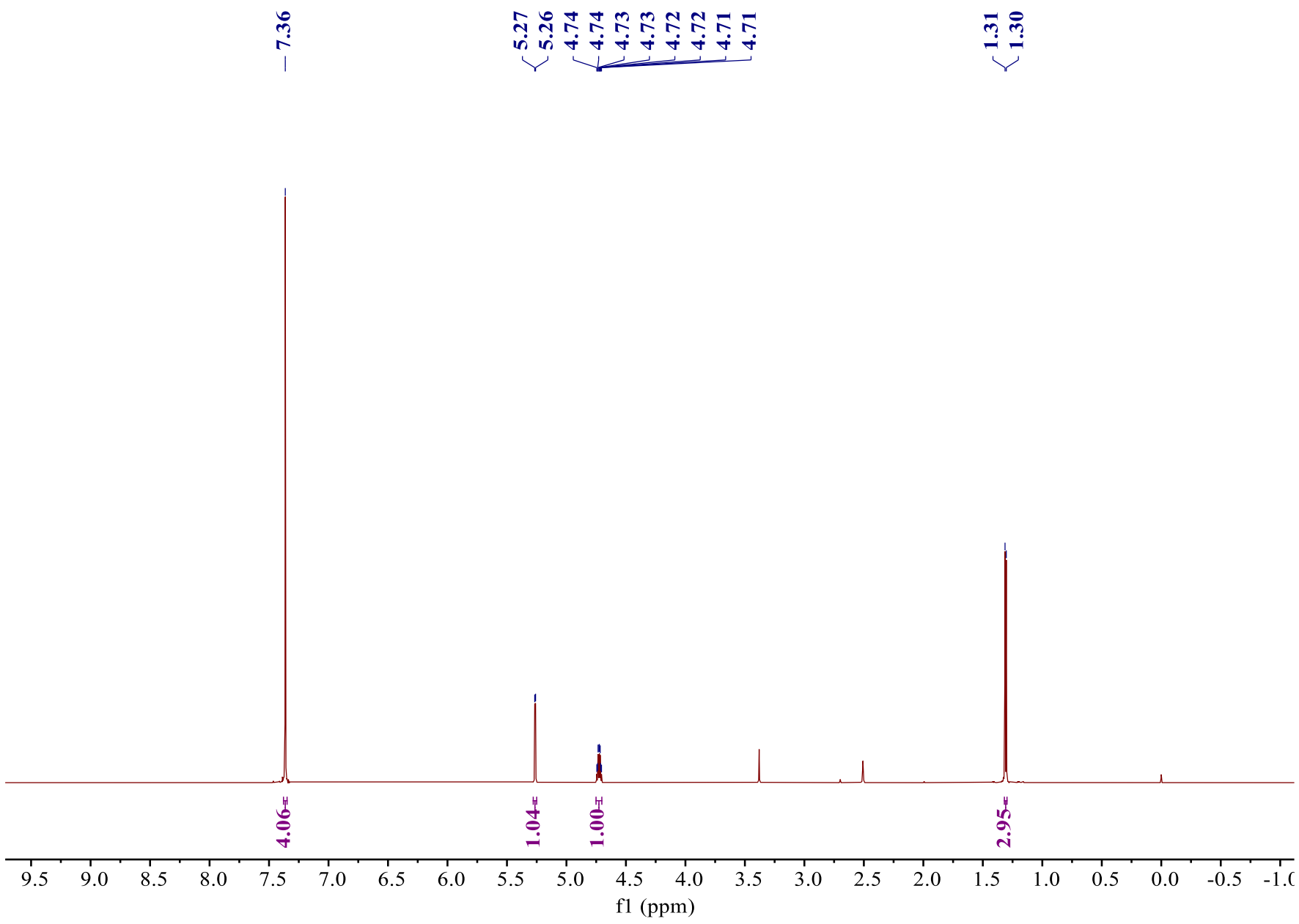

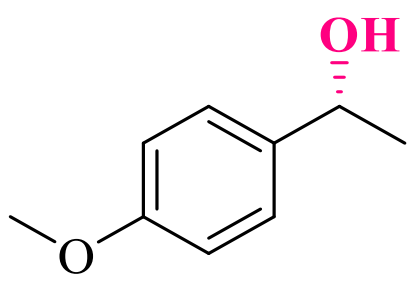

(*R*)-1-(4-methoxyphenyl)ethan-1-ol (1f). Known compound, see: *J. Am. Chem. Soc.* 126, 986-987, (2004).

The product **1f** was purified by flash chromatography on silica gel (300-400 mesh). ^{1}H NMR (600 MHz, CHLOROFORM-*D*) δ 7.30 – 7.27 (m, 2H), 7.25 – 7.22 (m, 2H), 4.87 – 4.82 (q, *J* = 6.5 Hz, 1H), 2.49 – 2.46 (s, 3H), 1.96 – 1.90 (s, 1H), 1.49 – 1.46 (d, *J* = 6.5 Hz, 3H).

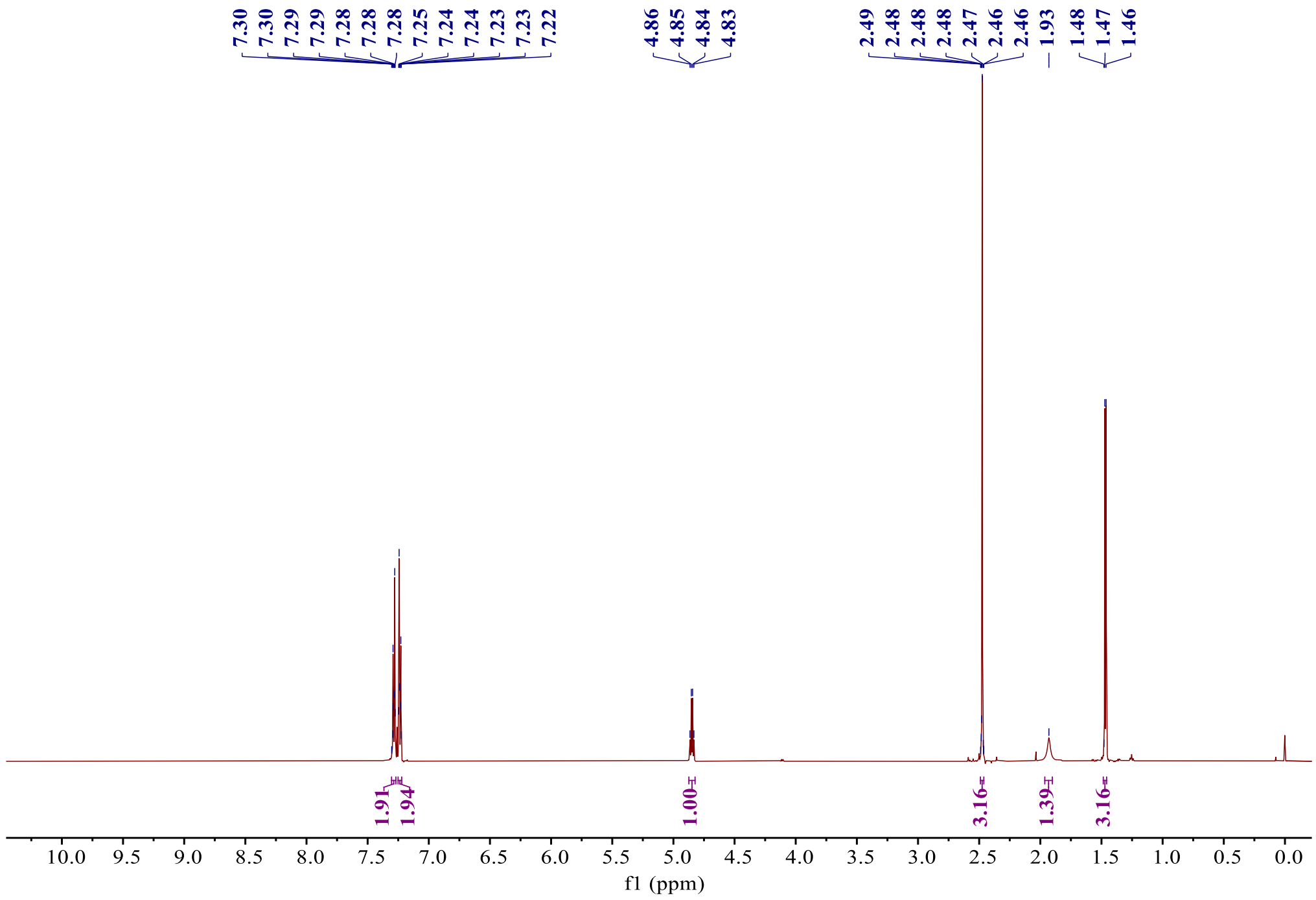

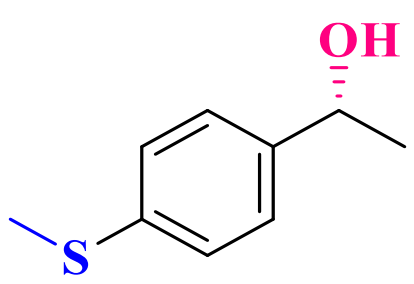

(*R*)-1-(4-(Methylthio)phenyl)ethan-1-ol (1g). Known compound, see: *J. Org. Chem.* 69, 6860-6866, (2004).

The product **1g** was purified by flash chromatography on silica gel (300-400 mesh). ^{1}H NMR (600 MHz, CHLOROFORM-*D*) δ 7.32 – 7.28 (m, 2H), 6.90 – 6.87 (m, 2H), 4.88 – 4.82 (q, *J* = 6.4 Hz, 1H), 3.82 – 3.79 (s, 3H), 1.83 – 1.80 (s, 1H), 1.49 – 1.46 (d, *J* = 6.5 Hz, 3H).

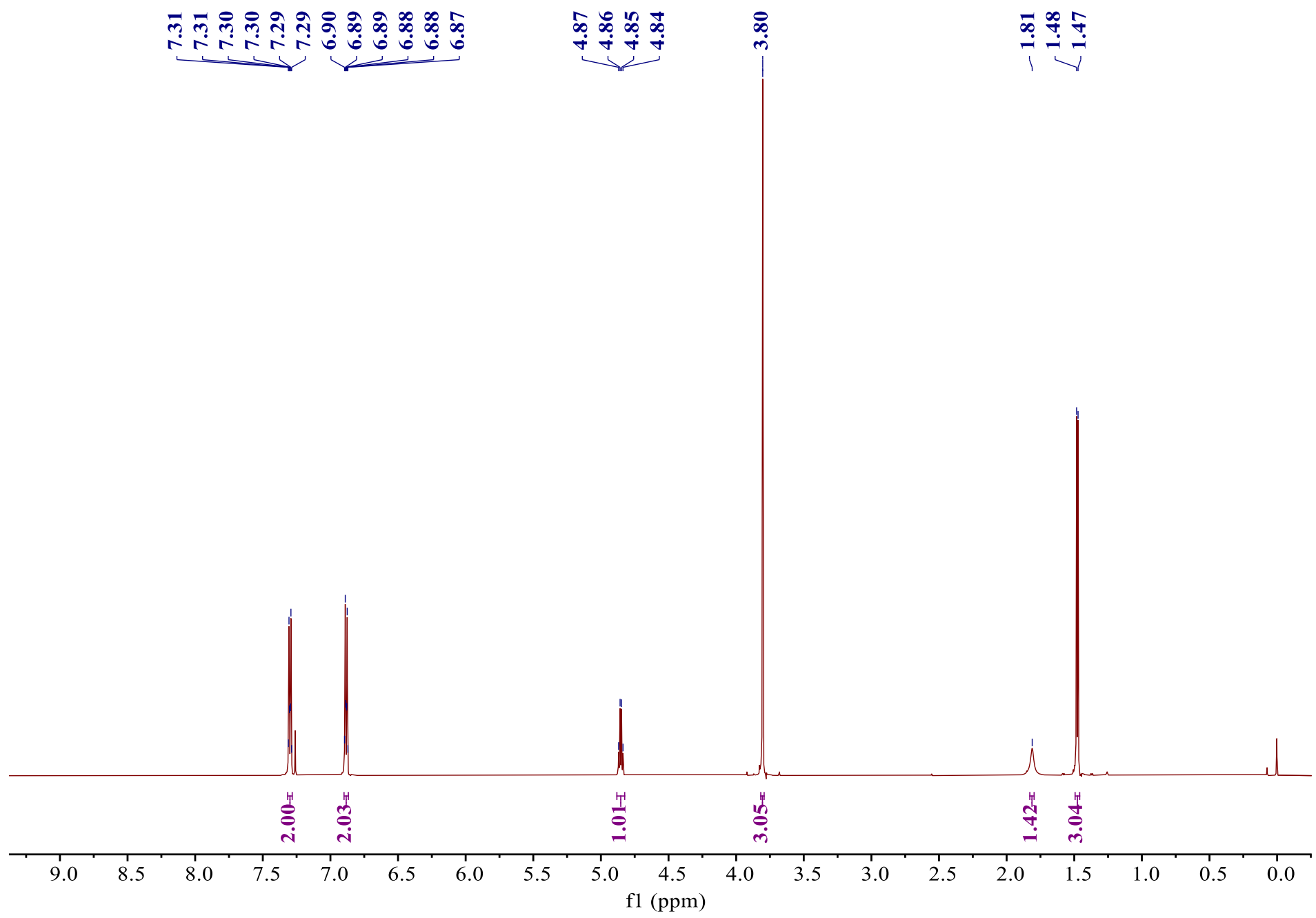

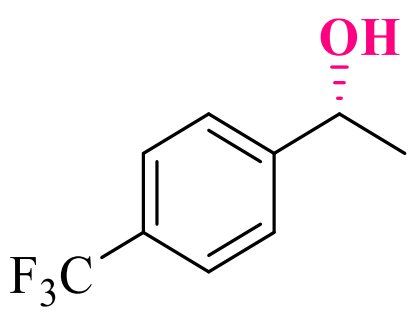

(*R*)-1-(4-(Trifluoromethyl)phenyl)ethan-1-ol (1h). Known compound, see: Org. Lett. 2, 4173-4176, (2000).

The product **1h** was purified by flash chromatography on silica gel (300-400 mesh). ^{1}H NMR (600 MHz, CHLOROFORM-*D*) δ 7.63 – 7.59 (m, 2H), 7.51 – 7.47 (m, 2H), 4.99 – 4.95 (q, *J* = 6.5 Hz, 1H), 1.86 – 1.81 (s, 1H), 1.52 – 1.50 (d, *J* = 6.5 Hz, 3H).

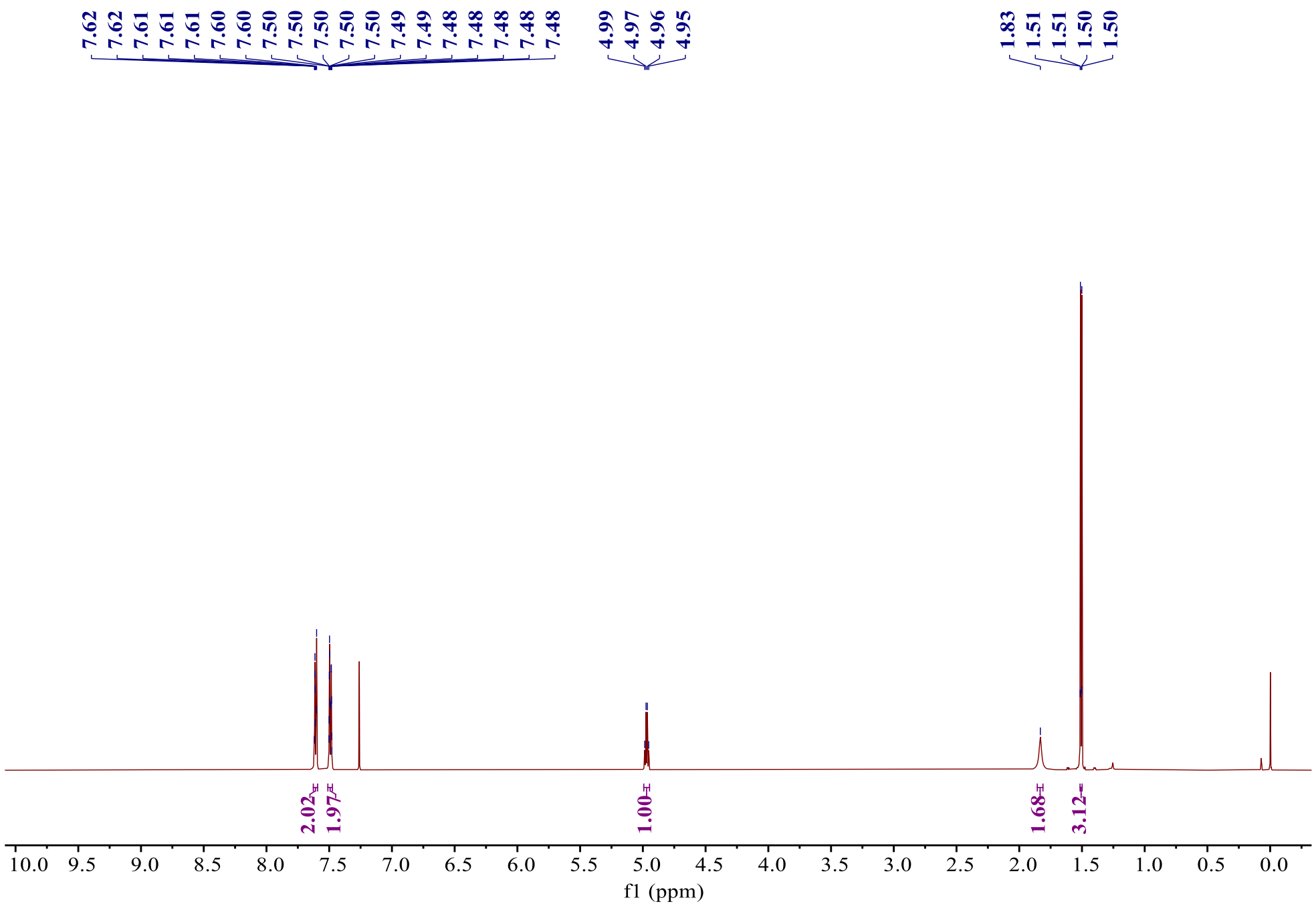

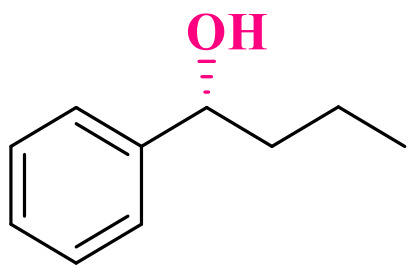

(*R*)-1-Phenylbutan-1-ol (1i). Known compound, see: *J. Org. Chem.* 53, 1231-8, (1988).

The product **1i** was purified by flash chromatography on silica gel (300-400 mesh). ^{1}H NMR (600 MHz, CHLOROFORM-*D*) δ 7.36 – 7.34 (d, *J* = 4.9 Hz, 4H), 7.30 – 7.26 (m, 1H), 4.69 – 4.66 (dd, *J* = 7.6, 5.8 Hz, 1H), 1.84 – 1.82 (s, 1H), 1.82 – 1.64 (m, 2H), 1.49 – 1.27 (m, 2H), 0.96 – 0.92 (t, *J* = 7.4 Hz, 3H).

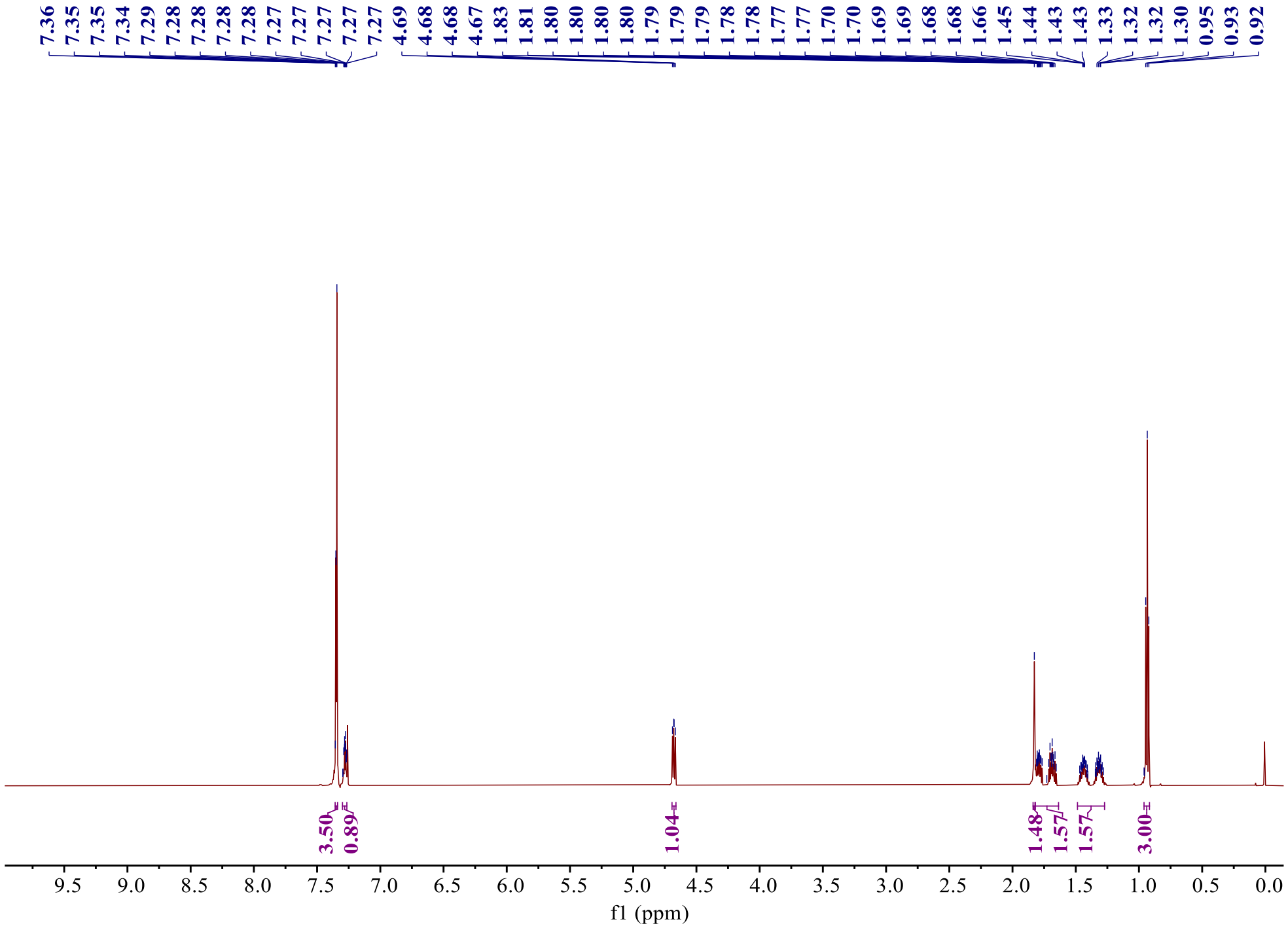

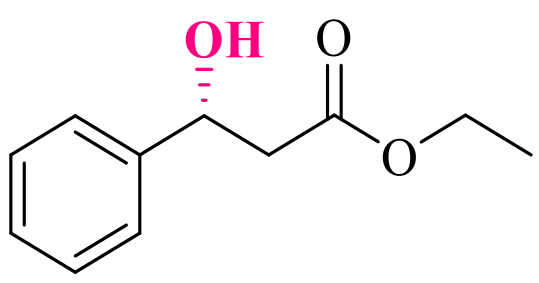

Ethyl (*R*)-3-hydroxy-3-phenylpropanoate (1j). Known compound, see: *J. Am. Chem. Soc.* 145, 6100-6111, (2023).

The product **1j** was purified by flash chromatography on silica gel (300-400 mesh). ^{1}H NMR (600 MHz, CHLOROFORM-*D*) δ 7.37 – 7.33 (m, 4H), 7.30 – 7.26 (td, *J* = 5.4, 2.6 Hz, 1H), 4.97 – 4.91 (dd, *J* = 8.9, 3.6 Hz, 1H), 3.88 – 3.79 (dd, *J* = 7.6, 4.0 Hz, 2H), 2.88 – 2.75 (s, 3H), 2.03 – 1.89 (m, 2H).

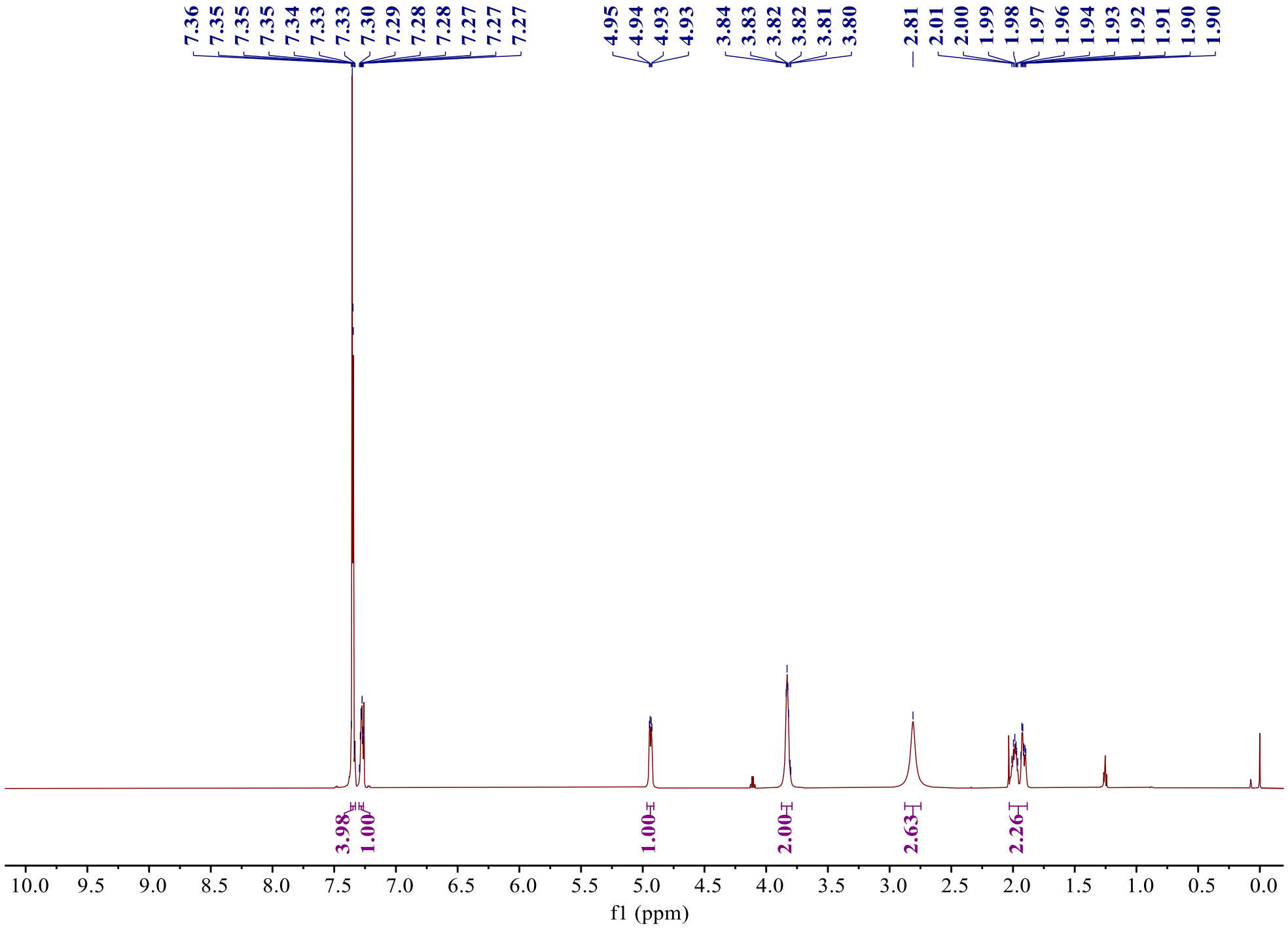

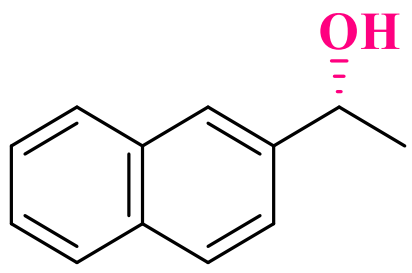

(*R*)-1-(Naphthalen-2-yl)ethan-1-ol (1k). Known compound, see: *Chem.* 10, 250-264, (2024).

The product **1a** was purified by flash chromatography on silica gel (300-400 mesh). ^{1}H NMR (600 MHz, CHLOROFORM-*D*) δ 7.86 – 7.82 (m, 3H), 7.81 – 7.80 (d, *J* = 1.8 Hz, 1H), 7.52 – 7.46 (m, 3H), 5.08 – 5.04 (q, *J* = 6.5 Hz, 1H), 2.06 – 2.00 (s, 1H), 1.60 – 1.57 (d, *J* = 6.5 Hz, 3H).

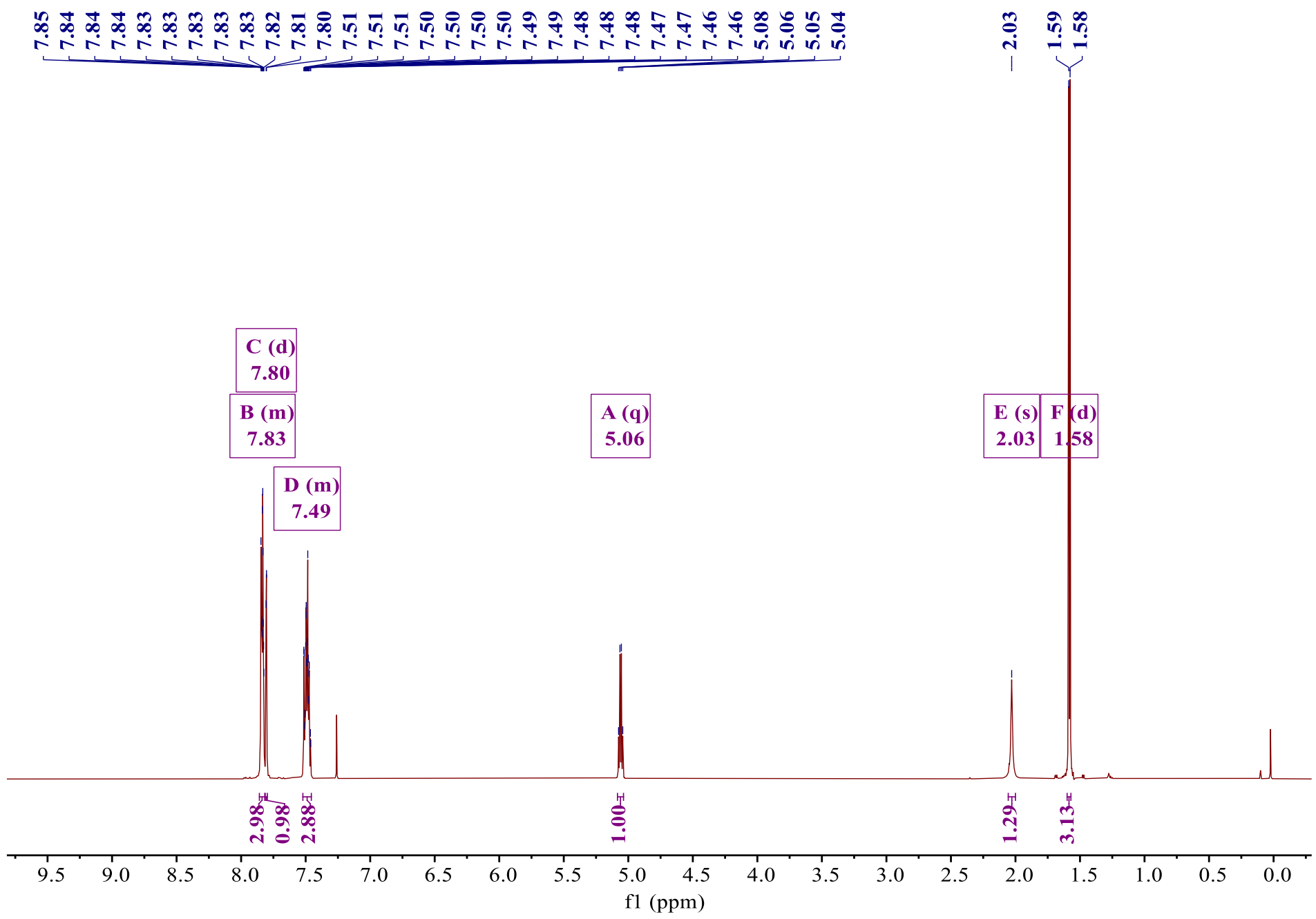

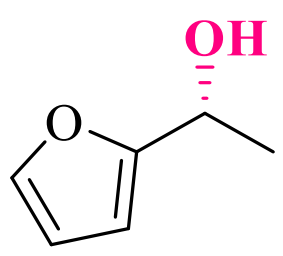

(*R*)-1-(Furan-2-yl)ethan-1-ol (1l). Known compound, see: *Org. Lett*. 2, 4173-4176, (2000).

The product **1l** was purified by flash chromatography on silica gel (300-400 mesh). ^{1}H NMR (600 MHz, CHLOROFORM-*D*) δ 7.39 – 7.36 (m, 1H), 6.35 – 6.32 (dd, *J* = 3.2, 1.8 Hz, 1H), 6.25 – 6.22 (d, *J* = 3.3 Hz, 1H), 4.92 – 4.86 (q, *J* = 6.6 Hz, 1H), 2.00 – 1.89 (s, 1H), 1.56 – 1.54 (d, *J* = 6.6 Hz, 3H).

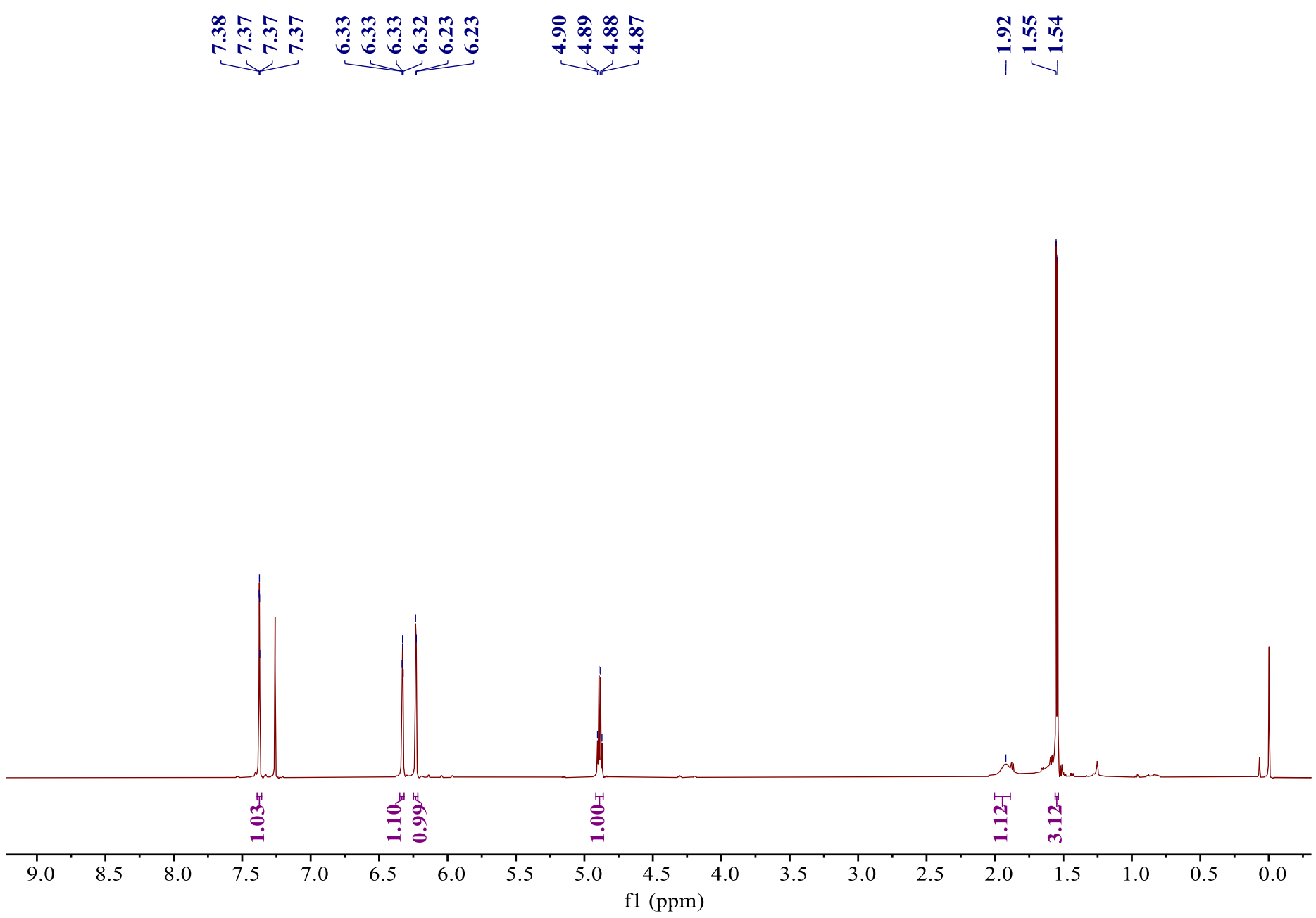

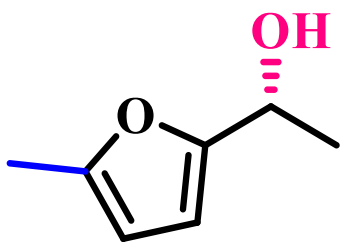

(*R*)-1-(5-Methylfuran-2-yl)ethan-1-ol (1m). Known compound, see: *Chem.* 10, 250-264. (2024).

The product **1a** was purified by flash chromatography on silica gel (300-400 mesh). ^{1}H NMR (600 MHz, CHLOROFORM-*D*) δ 6.11 – 6.09 (d, *J* = 3.1 Hz, 1H), 5.91 – 5.87 (dq, *J* = 3.3, 1.1 Hz, 1H), 4.86 – 4.80 (q, *J* = 6.9 Hz, 1H), 2.31 – 2.27 (d, *J* = 1.1 Hz, 3H), 1.91 – 1.87 (d, *J* = 3.7 Hz, 1H), 1.53 – 1.51 (d, *J* = 6.6 Hz, 3H).

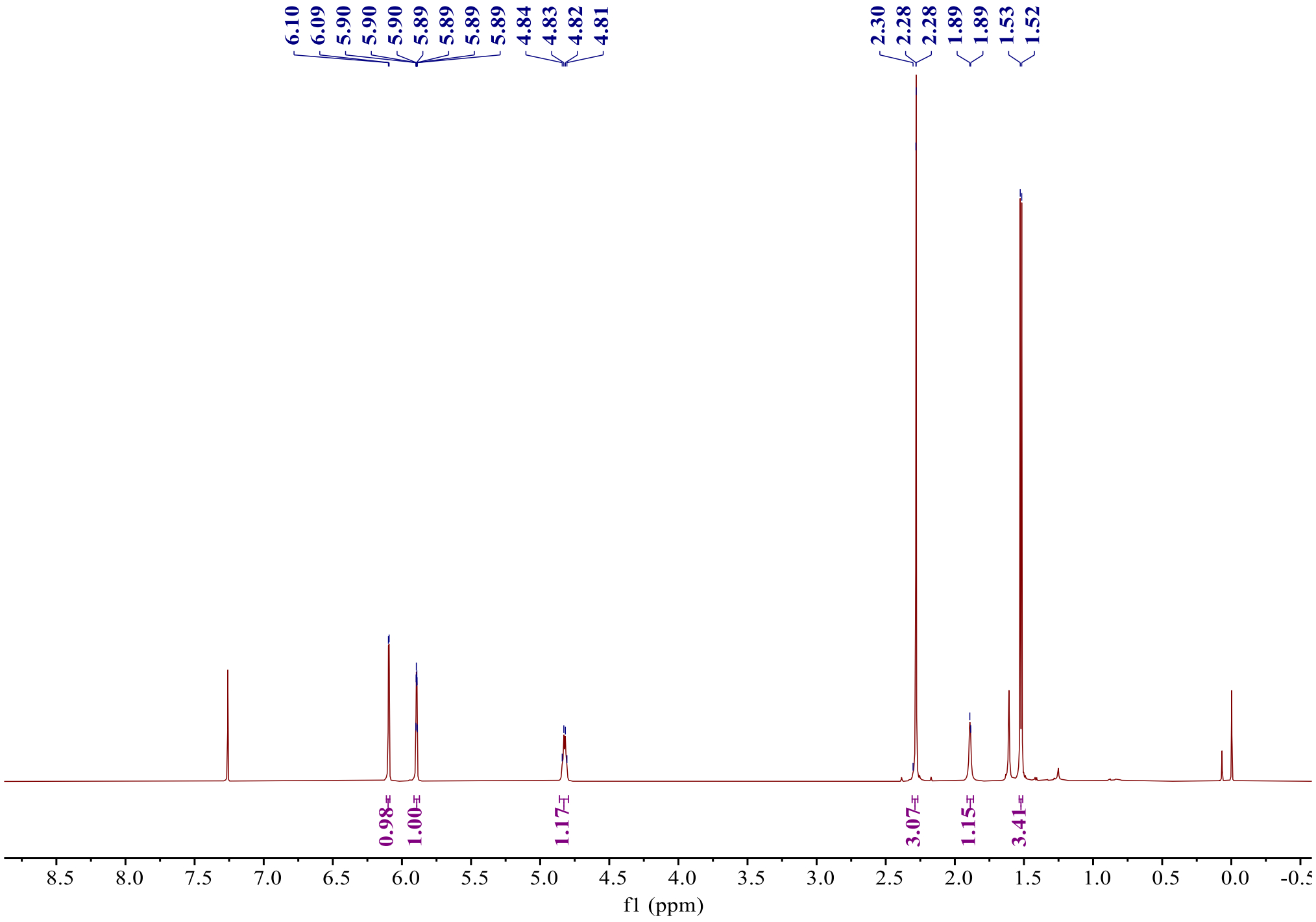

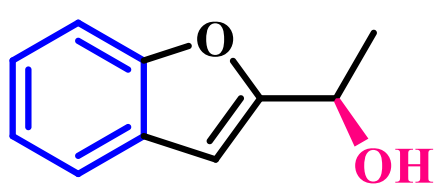

(*R*)-1-(Benzofuran-2-yl)ethan-1-ol (1n). Known compound, see: *J. Org. Chem.* 54, 2085-91, (1989).

The product **1n** was purified by flash chromatography on silica gel (300-400 mesh). ^{1}H NMR (600 MHz, CHLOROFORM-*D*) δ 7.56 – 7.51 (dt, *J* = 7.6, 1.8 Hz, 1H), 7.47 – 7.43 (m, 1H), 7.28 – 7.23 (m, 1H), 7.22 – 7.19 (m, 1H), 6.66 – 6.46 (m, 1H), 5.02 – 4.97 (m, 1H), 2.40 – 2.23 (s, 1H), 1.64 – 1.59 (dt, *J* = 6.6, 2.1 Hz, 3H).

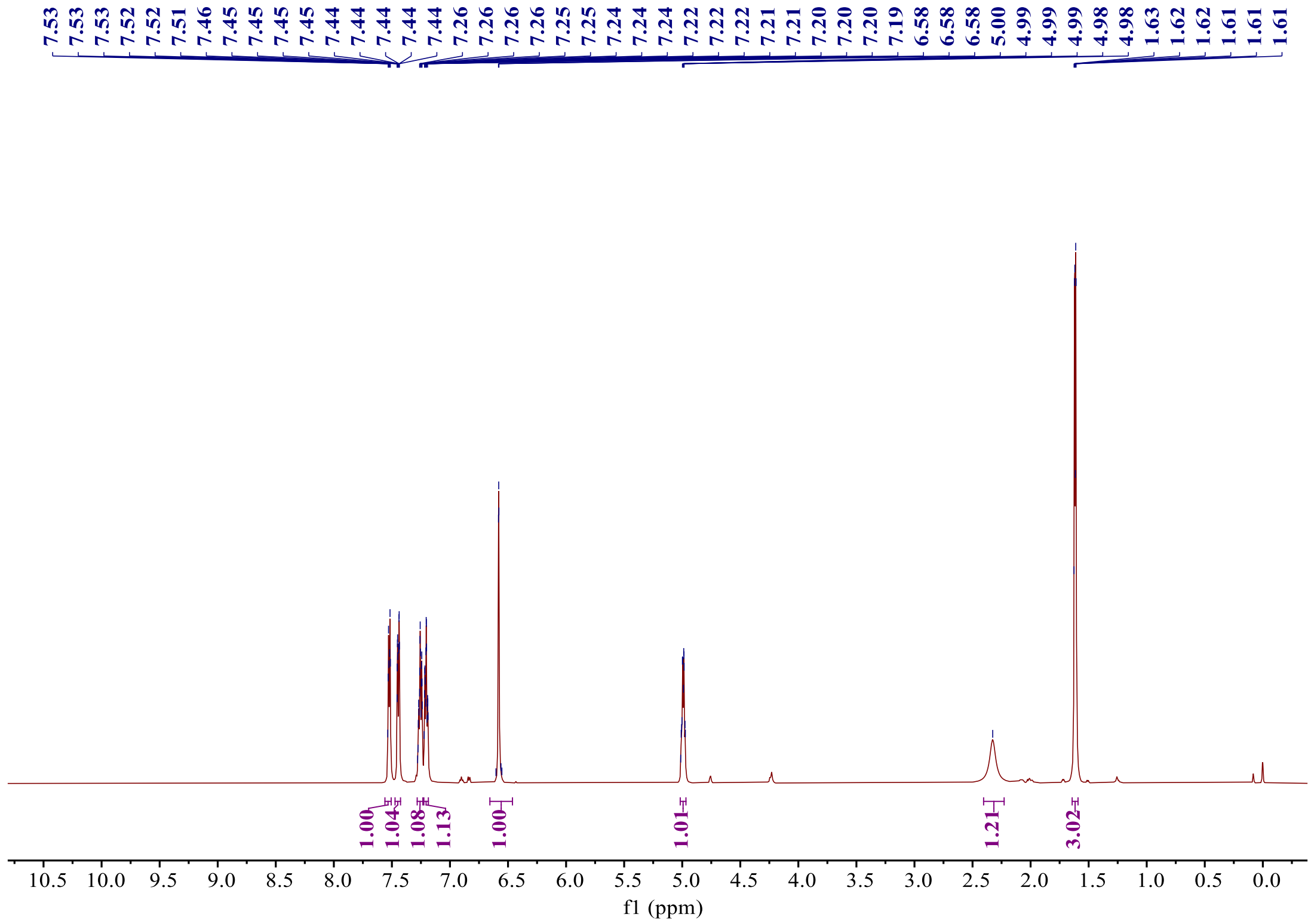

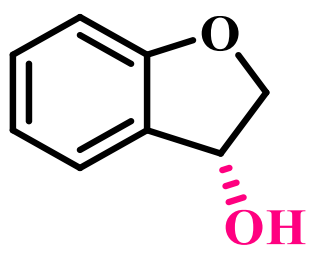

(*R*)-2,3-Dihydrobenzofuran-3-ol (1o). Known compound, see: Heterocycl. 80, 141-147, (2010).

The product **1o** was purified by flash chromatography on silica gel (300-400 mesh). ^{1}H NMR (600 MHz, CHLOROFORM-*D*) δ 7.41 – 7.37 (m, 1H), 7.28 – 7.23 (m, 1H), 6.96 – 6.91 (tt, *J* = 7.4, 0.9 Hz, 1H), 6.89 – 6.85 (d, *J* = 8.1 Hz, 1H), 5.29 – 5.27 (dd, *J* = 6.6, 2.5 Hz, 1H), 4.50 – 4.46 (ddd, *J* = 10.6, 6.5, 0.8 Hz, 1H), 4.40 – 4.36 (ddd, *J* = 10.7, 2.5, 0.7 Hz, 1H), 2.34 – 2.20 (s, 1H).

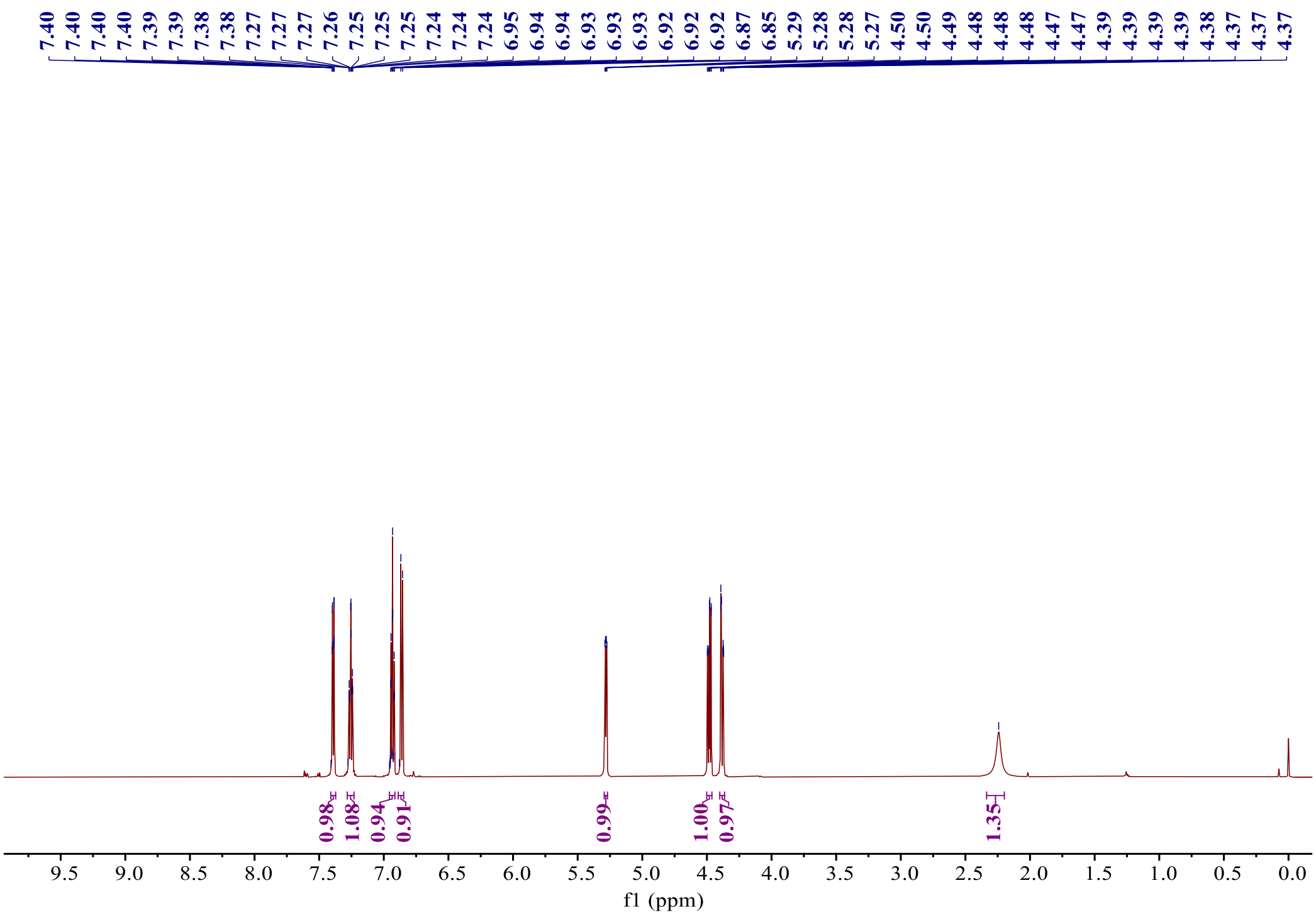

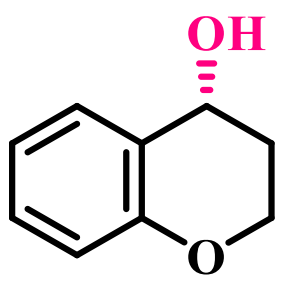

(*R*)-Chroman-4-ol (1p). ***J. Organomet. Chem*. 346, 413-24, (1988).**

The product **1p** was purified by flash chromatography on silica gel (300-400 mesh). ^{1}H NMR (600 MHz, CHLOROFORM-*D*) δ 7.33 – 7.28 (dt, *J* = 7.7, 2.0 Hz, 1H), 7.24 – 7.17 (m, 1H), 6.96 – 6.90 (tdd, *J* = 7.4, 2.4, 1.2 Hz, 1H), 6.87 – 6.82 (d, *J* = 8.2 Hz, 1H), 4.80 – 4.75 (q, *J* = 3.5 Hz, 1H), 4.31 – 4.19 (hd, *J* = 5.6, 2.6 Hz, 2H), 2.14 – 2.05 (m, 2H), 2.04 – 1.96 (m, 1H).

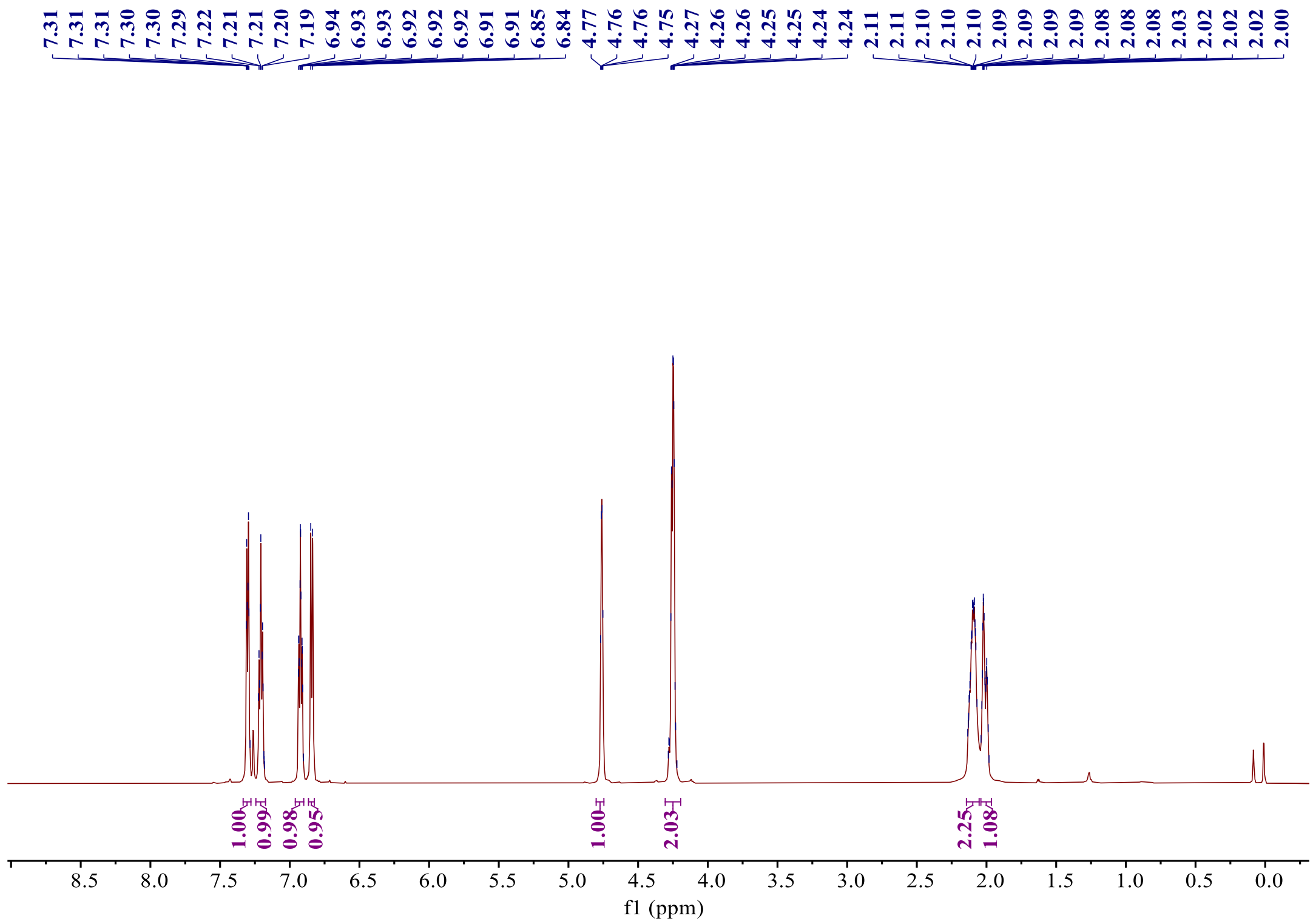

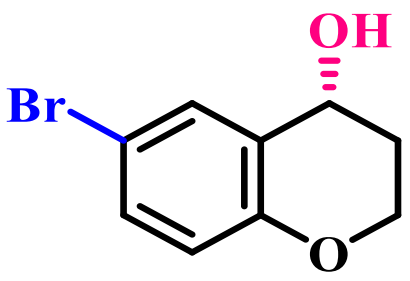

(*R*)-6-Bromochroman-4-ol (1q). Known compound, see: *Appl. Organomet. Chem*. 38, e7379, (2024).

The product **1q** was purified by flash chromatography on silica gel (300-400 mesh). ^{1}H NMR (600 MHz, CHLOROFORM-*D*) δ 7.44 – 7.41 (t, *J* = 2.5 Hz, 1H), 7.29 – 7.24 (dq, *J* = 7.5, 2.5 Hz, 1H), 6.76 – 6.68 (dd, *J* = 8.8, 2.2 Hz, 1H), 4.75 – 4.68 (m, 1H), 4.28 – 4.18 (ddd, *J* = 7.9, 3.6, 1.7 Hz, 2H), 2.32 – 2.19 (s, 1H), 2.12 – 2.04 (m, 1H), 2.01 – 1.94 (m, 1H).

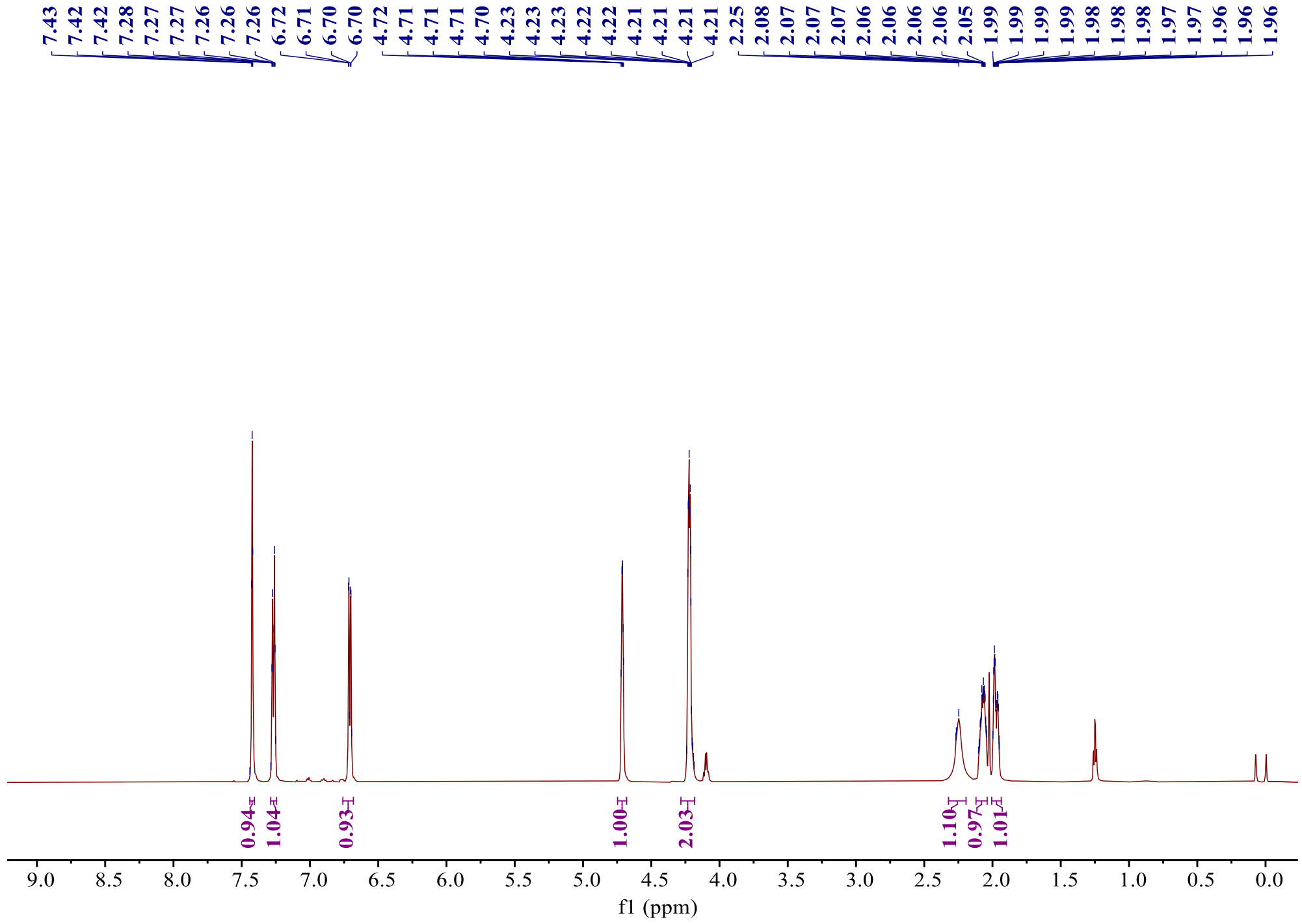

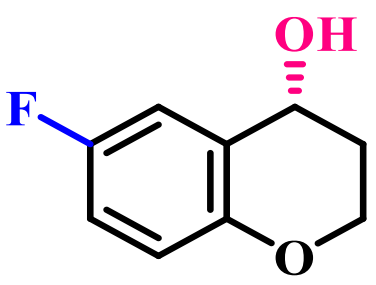

(*R*)-6-Fluorochroman-4-ol (1r). Known compound, see: *Tetrahedron Lett*. 53, 3828-3830, (2012).

The product **1r** was purified by flash chromatography on silica gel (300-400 mesh). ^{1}H NMR (600 MHz, CHLOROFORM-*D*) δ 7.07 – 6.98 (dq, *J* = 9.2, 3.2 Hz, 1H), 6.95 – 6.84 (m, 1H), 6.81 – 6.74 (m, 1H), 4.78 – 4.70 (dt, *J* = 6.4, 3.1 Hz, 1H), 4.27 – 4.17 (ddd, *J* = 7.0, 4.4, 2.4 Hz, 2H), 2.20 – 2.14 (s, 1H), 2.14 – 2.06 (dddd, *J* = 14.4, 7.4, 3.6, 1.8 Hz, 1H), 2.02 – 1.95 (m, 1H).

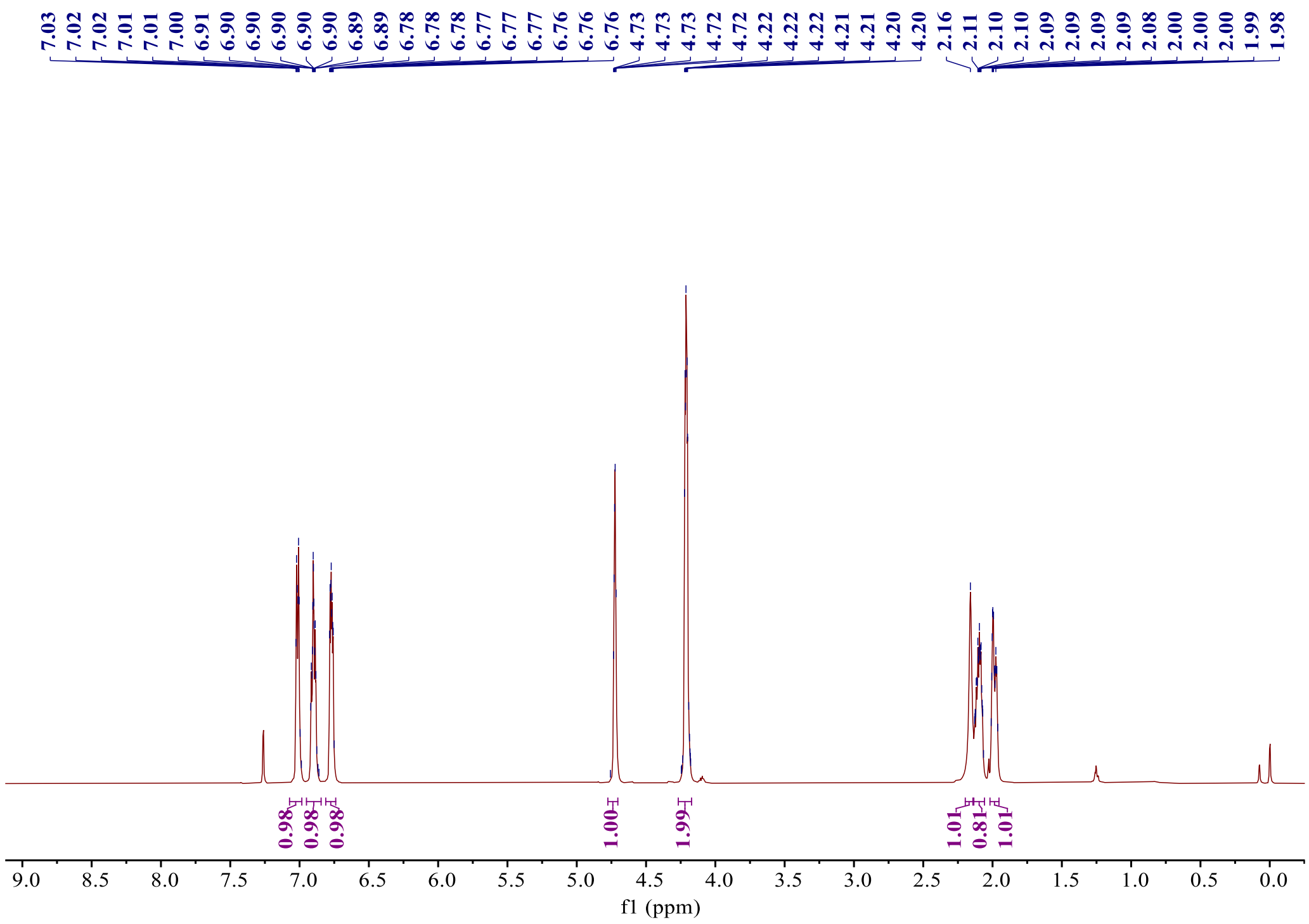

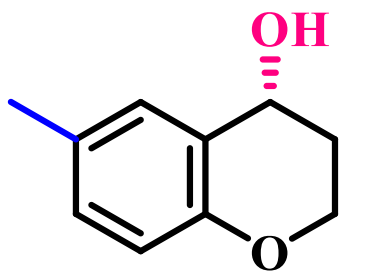

(*R*)-6-Methylchroman-4-ol (1s). Known compound, see: *J. Med. Chem*. 49, 1818-1827, (2006).

The product **1s** was purified by flash chromatography on silica gel (300-400 mesh). ^{1}H NMR (600 MHz, CHLOROFORM-*D*) δ 7.15 – 7.09 (d, *J* = 2.3 Hz, 1H), 7.04 – 6.95 (dt, *J* = 8.4, 2.3 Hz, 1H), 6.79 – 6.72 (m, 1H), 4.74 – 4.70 (td, *J* = 4.2, 1.8 Hz, 1H), 4.24 – 4.18 (m, 2H), 2.29 – 2.26 (d, *J* = 2.0 Hz, 3H), 2.13 – 2.02 (m, 2H), 2.02 – 1.96 (m, 1H).

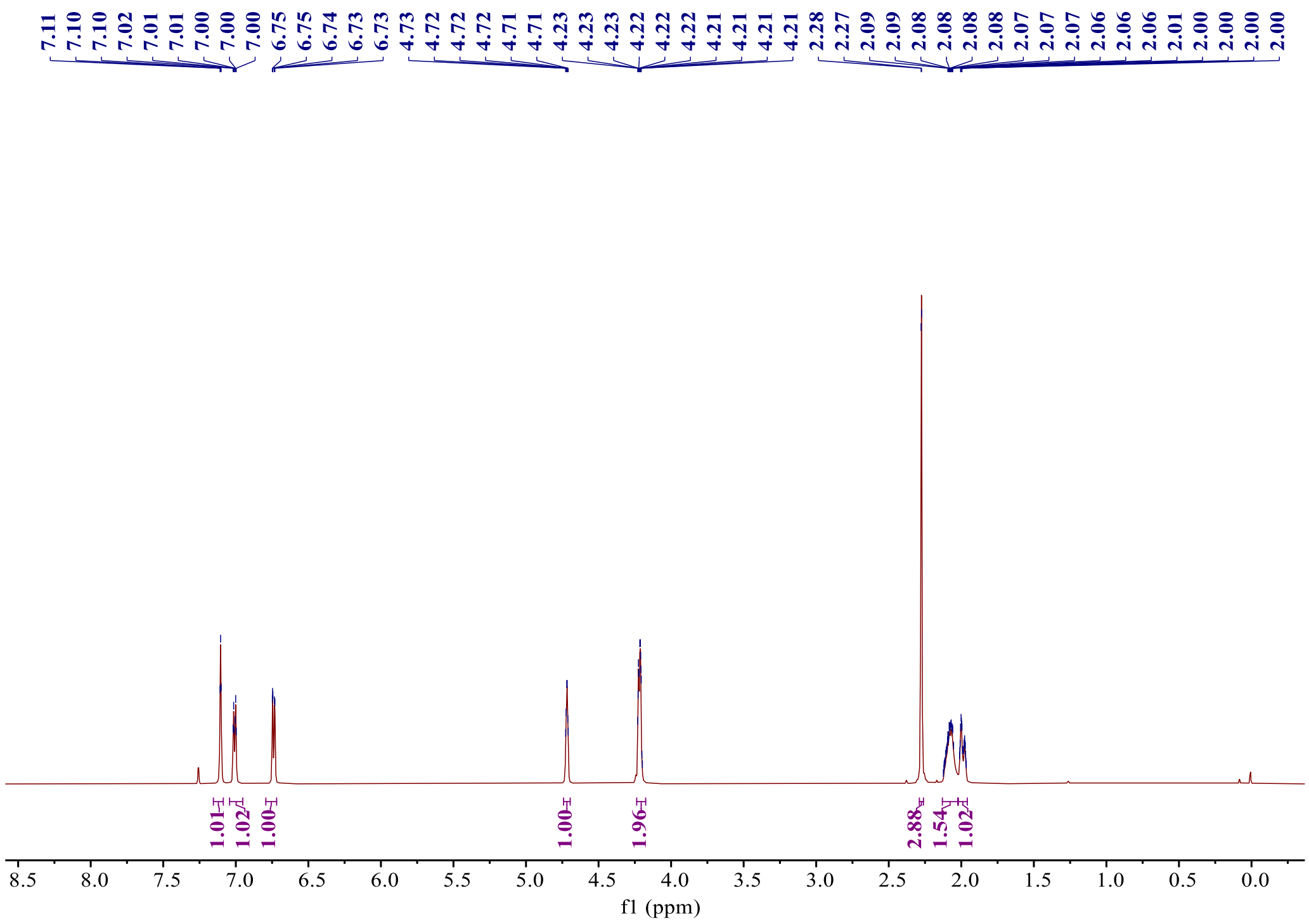

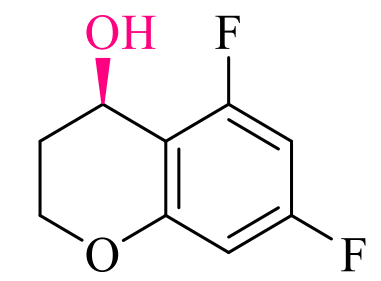

(*R*)-5,7-Difluorochroman-4-ol (1t): Known compound, see: *Org. Prep. Proced. Int.* 56, 264-270, (2024).

The product **1t** was purified by flash chromatography on silica gel (300-400 mesh). ^{1}H NMR (600 MHz, CHLOROFORM-*D*) δ 6.45 – 6.37 (ddq, *J* = 10.4, 4.1, 2.4 Hz, 2H), 5.02 – 4.97 (t, *J* = 3.3 Hz, 1H), 4.33 – 4.20 (m, 2H), 2.27 – 2.15 (s, 1H), 2.07 – 1.95 (m, 2H). ^{19}F NMR (565 MHz, CHLOROFORM-*D*) δ -117.15, -109.09.

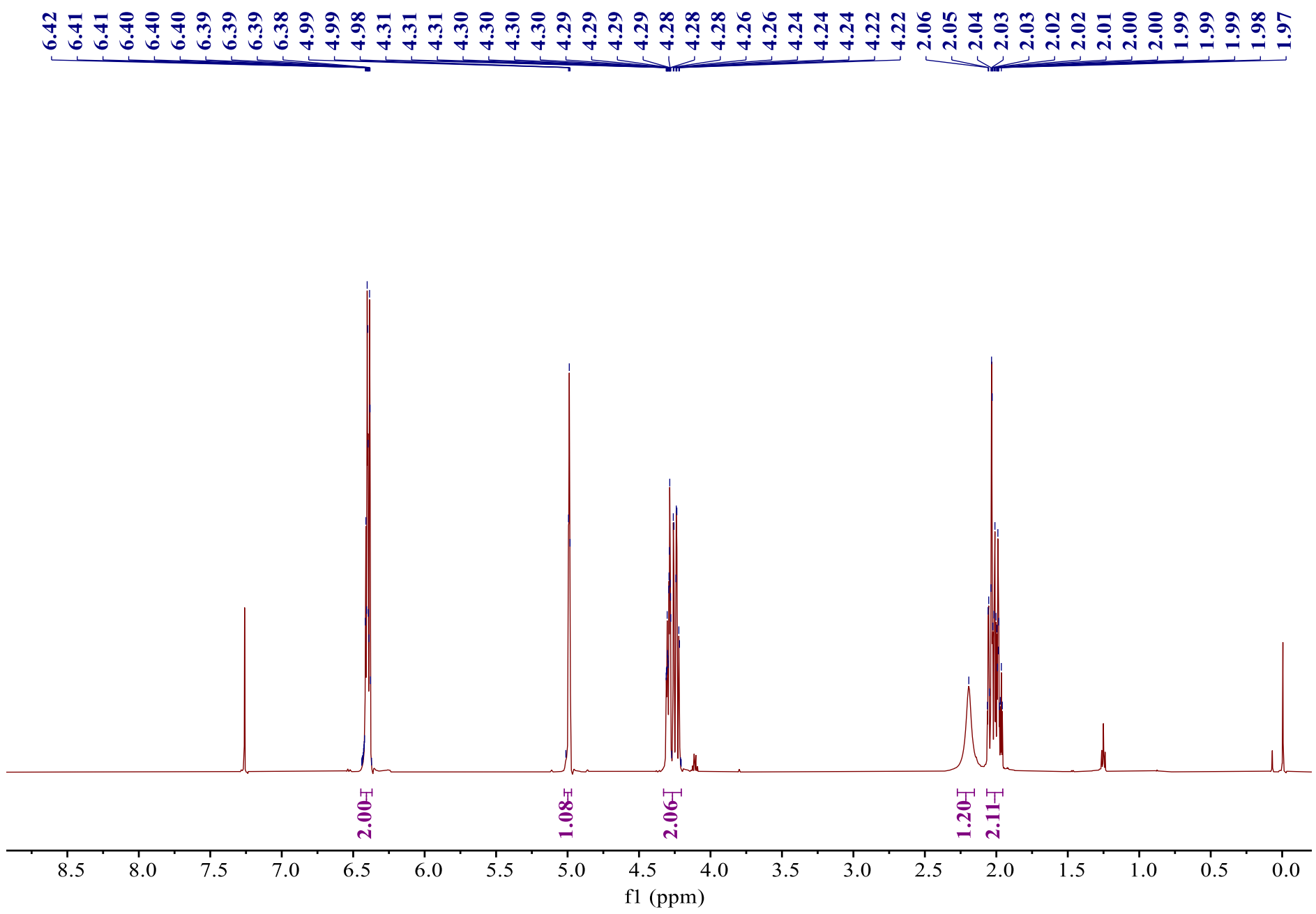

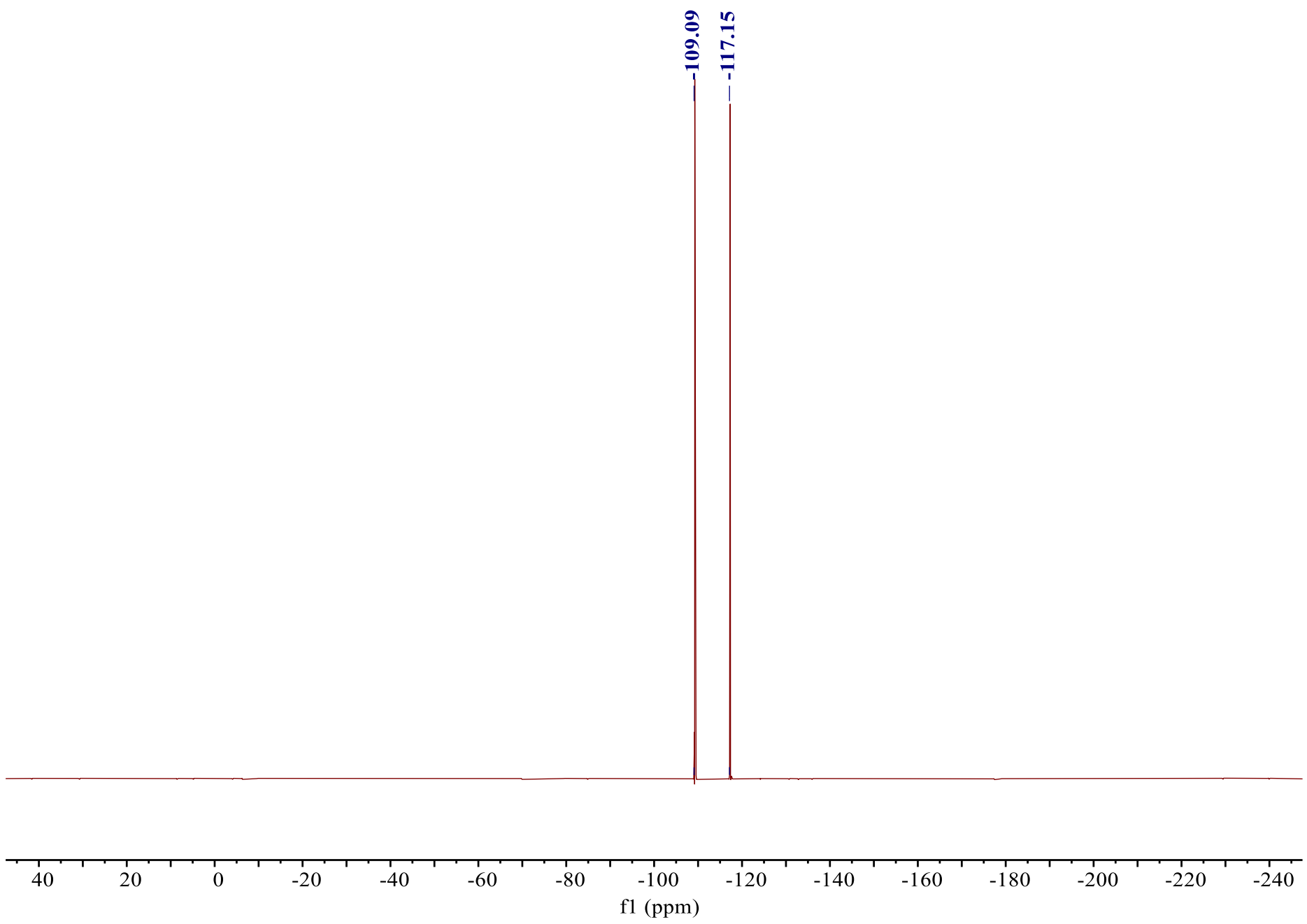

Comment 17: Finally, the discussion of Ru leaching should be reconsidered. Although a leaching value of 0.27 wt% is reported, the initial Ru loading is 0.83 wt% (Table S8), corresponding to approximately 33% metal loss after catalysis. This level of leaching is significant and should be discussed more critically.

Response: We sincerely thank the reviewer for this critical and highly constructive comment. We completely agree that the initially observed 33% metal loss was significant and warranted a much deeper investigation. Prompted by your insightful feedback, we re-evaluated the leaching behavior, which led to a deeper understanding of the coordination microenvironment.

When analyzing the initial data, we observed an intriguing phenomenon: the loss of catalytic activity did not correlate proportionally with the extent of metal leaching. Specifically, while significant metal loss occurred in early cycles, the catalytic activity and enantioselectivity remained remarkably stable in subsequent runs. We hypothesized that this discrepancy stems from the presence of two distinct types of Ru species in the initial scaled-up batch. On one hand, the true catalytic centers consist of active Ru strongly coordinated with the chiral sulfonamide groups within the confined pockets, which are highly stable. On the other hand, weakly bound "spectator Ru" species are likely attached to the imine bonds on the COF backbone—as evidenced by our control experiment with TFPB-DATP-Ru (which lacks chiral pockets but still showed 0.17 wt% Ru loading)—or physically trapped within the pores. Because of the large scale of the initial gram-scale synthesis, the standard Soxhlet extraction might have been insufficient to remove these loosely bound species, which readily leached out during the initial cycles without affecting the overall catalytic performance.

To rigorously test this hypothesis and eliminate the weakly bound spectator Ru, we synthesized a new batch of CCOF2-Ru for the gram-scale recycling experiments. In this optimized protocol, we appropriately extended both the metal coordination time to ensure pocket saturation and the Soxhlet extraction time to thoroughly remove any physically trapped or weakly imine-bound Ru. The new results perfectly validate our hypothesis. For the optimized CCOF2-Ru, the initial Ru loading was 0.89 wt%. After five consecutive gram-scale recycling runs, the Ru content remained at 0.79 wt%, corresponding to a significantly reduced metal loss of only ~11% (compared to the previous 33%). Meanwhile, the exceptional catalytic activity and enantioselectivity were fully maintained throughout the cycles.

This critical re-evaluation not only significantly improved the absolute stability metrics of our material but also provided compelling indirect evidence for the robustness of our targeted chiral pocket design, demonstrating that the Ru species anchored within the engineered chiral microenvironment are highly stable and resistant to leaching. Accordingly, we have comprehensively revised the discussion on catalyst recycling in the main text to critically address the initial leaching and present the optimized leaching data. Furthermore, we have updated the corresponding recycling data in the SI to reflect the updated Ru content determined by ICP-OES across the five cycles.

Manuscript Change:

Supporting Information (revised Supplementary Table 15, page 86):

Supplementary Table 15. Ru content determined by ICP-OES.

Cycles	Ru content (wt%)		
	CCOF2-Ru (0.89)	CCOF1-Ru (1.62)	CCOF0-Ru (2.12)
1	0.87	1.54	1.64
2	0.84	1.40	1.22
3	0.83	1.27	1.10
4	0.80	-	-
5	0.79	-	-

Reviewer 2:

This study illustrates the synthesis of three CCOFs, including one two-component and two three-component systems. Ruthenium metallization afforded CCOF-2-Ru, which catalyzes asymmetric transfer hydrogenation of ketones. The authors claim that this work establishes a versatile paradigm for MCCOF design and addresses key challenges in heterogeneous asymmetric catalysis. However, this claim is not well supported. Similar mixed-linker strategies have been reported several years ago and are now well studied. Therefore, I cannot recommend its publication in Angewandte Chemie. In addition, several issues need to be addressed before consideration in other journals.

Response: We sincerely thank the reviewer for their critical evaluation of our manuscript. We agree that multivariate (or mixed-linker) strategies have been extensively studied in metal-free COFs to tune crystallinity and stability. However, to the best of our knowledge, successfully translating this strategy to metallated chiral covalent organic frameworks (MCCOFs) remains unprecedented and highly challenging.

The design of MCCOFs is not a simple extension of metal-free systems. It requires overcoming severe trade-offs between (i) incorporating bulky chiral metal complexes, (ii) maintaining framework crystallinity and porosity, and (iii) preventing metal leaching during catalysis. As demonstrated in our study (Fig. 1 and Supplementary Fig. 23), binary MCCOFs (CCOF0-Ru and CCOF-DACH-Ru) suffer from either poor structural order or inferior enantioselectivity. By introducing the multivariate approach, our ternary system (CCOF2-Ru) successfully decouples these competing factors, utilizing an achiral linker (DATP) to relieve steric strain and enhance backbone stability, while the chiral linker creates a confined, highly active catalytic pocket. This synergistic effect leads to substantially higher structural order, superior catalytic performance, and suppressed metal leaching.

We have revised the manuscript to better articulate the unique challenges of MCCOF design and to clarify how our multivariate strategy specifically addresses the bottlenecks in heterogeneous asymmetric catalysis. We hope the reviewer agrees that this conceptual translation from metal-free COFs to functional MCCOFs represents a substantial advance in the field.

Manuscript Change:

The introduction and discussion sections have been revised to explicitly contrast the well-known metal-free multivariate COFs with the unique steric and electronic challenges of designing MCCOFs, highlighting the specific structural solutions

provided by our ternary design.

Comment 1: Only two mixed-linker CCOFs are reported, which cannot be considered true multivariate assemblies. Moreover, the PXRD simulations are poor (Rwp and Rw values are quite high). High-resolution TEM images should be provided to substantiate the proposed structures, especially given the authors' claim of high crystallinity;

Response: We sincerely appreciate the reviewer's rigorous evaluation of our structural data and terminology. We agree with the precise distinction regarding the term "multivariate," as "multivariate assembly" may indeed imply a higher degree of compositional complexity than our ternary systems currently exhibit. To ensure scientific rigor and align with established conventions in the field (e.g., J. Am. Chem. Soc. 2017, 139, 8277; Angew. Chem. Int. Ed. 2024, 63, e202405476), we have revised the terminology to the "multivariate approach" throughout the manuscript. This term accurately reflects our methodology of using mixed linkers to synergistically tune the framework's microenvironment without overstating the system's complexity.

Regarding the PXRD refinement residuals, we respectfully wish to provide additional context specific to metallated chiral COFs (MCCOFs). Achieving low R_{wp} and R_p values in COFs featuring bulky chiral groups and heavy transition metals (like Ru) is notoriously difficult, as the introduction of such complex functionalities typically induces significant local disorder and dampens X-ray scattering intensity. Despite these inherent challenges, our optimized structural models—refined from the low-symmetry P1 to the higher-symmetry $P2_1$ space group (Supplementary Table 3)—yielded R_{wp} <7% and R_p <5% for all COFs. In the specific context of highly functionalized and post-synthetically metallated COFs, these values are highly competitive and indicative of excellent long-range order. For comparison, recent landmark publications on complex COFs in top-tier journals report similar or higher residual values (e.g., *Nat. Commun.* 2022, 13, 1770: R_{wp} = 6.96%; *Adv. Funct. Mater.* 2024, 34, 2413943: R_{wp} = 8.89%).

Furthermore, we completely agree with the reviewer that given the complexity of our MCCOFs, PXRD alone is insufficient, and direct visual evidence is the most compelling way to substantiate our claims of high crystallinity. Prompted by this excellent suggestion, we have acquired new HRTEM images for the metallated frameworks, CCOF1-Ru and CCOF2-Ru (Supplementary Fig. 15b, c). As shown in the revised SI, these new HRTEM images clearly display long-range, well-defined honeycomb mesoporous channels. This direct visual evidence perfectly corroborates our PXRD models and unequivocally proves that the frameworks maintain robust structural integrity and high crystallinity even after the Ru-coordination process. In fact, this structural preservation highlights the core innovation of our work: the multivariate

approach successfully mitigates the steric strain of bulky metal complexes, preserving the crystalline order that is usually lost in binary MCCOFs.

We believe that the combination of our rigorous PXRD refinements and the new HRTEM evidence fully addresses the reviewer's concerns regarding the structural validity of our catalysts.

Manuscript Change:

Manuscript:

1) The term "multivariate assembly" has been replaced with "multivariate approach" throughout the text and Supporting Information.
2) New HRTEM images of CCOF1-Ru and CCOF2-Ru have been added to the Supporting Information (Supplementary Fig. 15).
3) The main text has been updated to integrate the HRTEM findings, highlighting how the visual evidence of ordered honeycomb channels supports the PXRD data and validates the structural stability of the MCCOFs post-metallation.

Supporting Information (revised Supplementary Fig. 15, page 45):

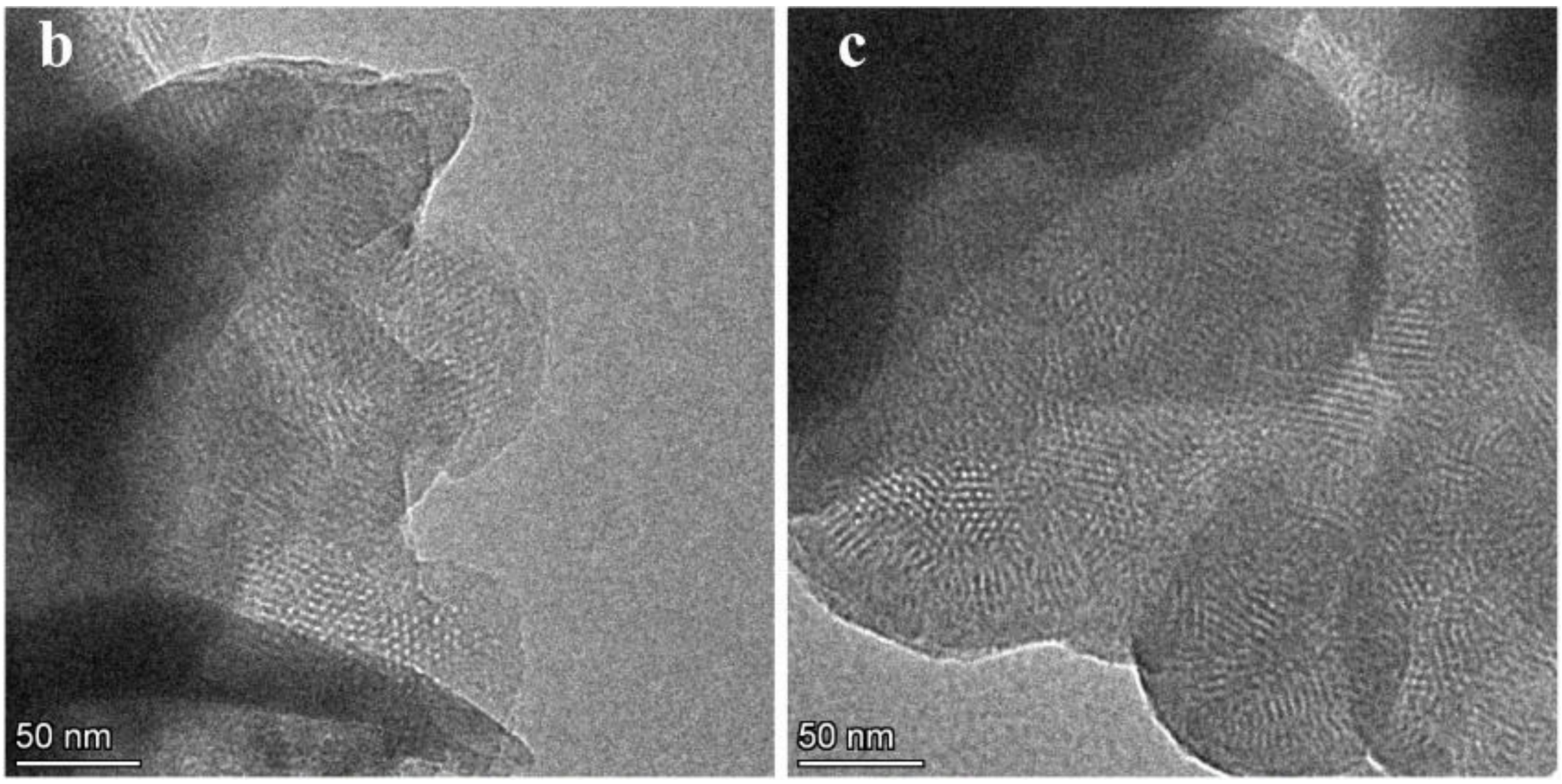

Supplementary Fig. 15 TEM image of **b** CCOF1-Ru and **c** CCOF2-Ru.

Comment 2: The catalytic study includes only ten typical substrates for asymmetric transfer hydrogenation, a well-established reaction in both homogeneous and heterogeneous systems. The observed activity and enantioselectivity are comparable to previously reported heterogeneous catalysts, offering limited advancement.

Response: We sincerely thank the reviewer for this critical and constructive evaluation.

We agree that demonstrating a broader, more challenging substrate scope is essential to fully establish the utility and superiority of a newly designed catalyst. Prompted by this comment, we have rigorously expanded our catalytic studies, doubling the substrate library from 10 to 20 ketone derivatives (now presented in the revised Fig. 5e). Crucially, this expanded scope goes well beyond typical substrates to include highly challenging and biologically significant skeletons, such as heterocyclic ketones (furanones and benzofuranones) and chroman-4-one derivatives. CCOF2-Ru exhibited excellent asymmetric catalytic performance across this diverse and sterically demanding library, unequivocally demonstrating the broad applicability and practical value of our catalyst in synthesizing high-value chiral alcohols.

Furthermore, we have provided a deeper discussion on the fundamental advancements our work brings to the field. While we acknowledge that the ATH of ketones is a classic reaction, the development of robust heterogeneous systems that can match the microenvironmental precision of homogeneous catalysts—while offering superior recyclability and site-isolation—remains a formidable and highly active frontier. This is evidenced by a continuous stream of recent breakthroughs in top-tier journals (e.g., *J. Am. Chem. Soc.* 2024, 146, 4942; *J. Am. Chem. Soc.* 2024, 146, 27736; *Chem* 2024, 10, 250). The primary advancement of our work lies not merely in incrementally improving absolute ee values, but in the fundamental structural innovation of the catalyst design. By employing a multivariate approach, we have successfully engineered a confined and precisely tunable "chiral pocket" within a robust COF matrix. This design achieves two critical breakthroughs: first, it effectively isolates the Ru catalytic centers, preventing the aggregation or deactivation commonly observed in traditional heterogeneous catalysts; second, it utilizes the synergistic effect of mixed linkers to create a tailored steric and electronic microenvironment. This engineered microenvironment mimics the precision of enzymatic or homogeneous systems, which proved to be the key to the excellent performance observed for the newly added bulky heterocyclic substrates. Therefore, while the absolute catalytic metrics are highly competitive with state-of-the-art heterogeneous catalysts, the true novelty of this work is the multivariate platform itself, which bridges the gap between homogeneous precision and heterogeneous stability to offer a powerful new paradigm for the future design of chiral porous materials.

Manuscript Change:

Manuscript:

1) The substrate scope in the main text (Fig. 5e) has been updated to include 20 substrates, featuring the newly tested challenging heterocyclic ketones and

chroman-4-one derivatives.

2) The accompanying discussion in the main text has been substantially expanded to highlight the biological relevance of these products.
3) We have incorporated recent literature (*J. Am. Chem. Soc.* 2024, 146, 4942; *Chem* 2024, 10, 250, etc.) into the introduction and discussion sections to contextualize the ongoing importance of heterogeneous ATH and to better highlight the structural design advancements of our multivariate MCCOF platform.

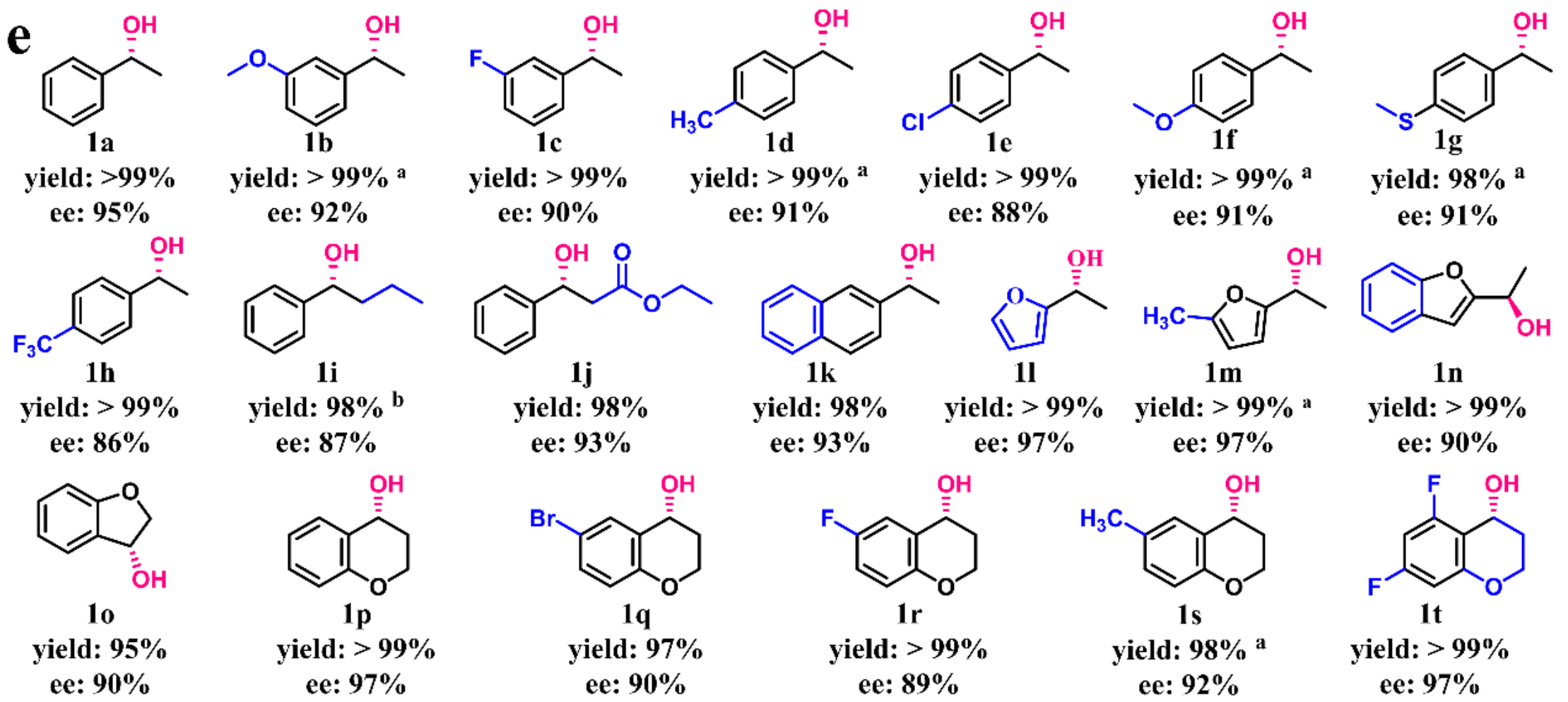

Fig. 5e Substrate scope for CCOF2-Ru-catalyzed ATH reaction of ketones (Reaction conditions: ketone (0.2 mmol), HCOONa (1 mmol), CCOF2-Ru (0.5 mol % of Ru based on ICP analysis), N_2 atmosphere, 1 mL ^{i}PrOH/ H_2O (1:1 v/v), 60 °C, 1 h unless noted. [a] 7 h, [b] 3 h).

Reviewer 3:

This manuscript presents a multivariate assembly strategy combined with post-synthetic metallization to construct metal-chiral covalent organic frameworks (MCCOFs) for asymmetric transfer hydrogenation of ketones. The work addresses issues such as structural instability, metal leaching, and limited recyclability, and reports notable catalytic results including high yield and enantioselectivity. However, several key aspects require clarification and additional data to meet the standards of Nature Communications, as outlined below. Major revision is therefore recommended.

Response: We sincerely thank the reviewer for their careful evaluation and highly constructive feedback. The insightful comments regarding structural characterization, mechanistic elucidation, and catalytic stability have been instrumental in improving the rigor and clarity of our manuscript. We have carefully addressed each point, performed the requested additional analyses, and revised the manuscript accordingly.

Comment 1: The crystallinity of CCOF-0-Boc is lower compared to CCOF-1-Boc and CCOF-2-Boc. Could this be due to partial Boc deprotection during the acidic solvothermal synthesis of CCOF-0-Boc, which alters the catalytic environment and pH, whereas in CCOF-2-Boc, the lower Boc content leads to less pH variation and thus better crystallinity and stability?

Response: We sincerely appreciate the reviewer's highly insightful hypothesis regarding the potential impact of partial Boc deprotection and pH variations. While this is a very valid chemical consideration, our systematic structural comparisons have determined that the primary factor governing the crystallinity in these systems is the severe steric hindrance imposed by the dense chiral functionalities.

To decouple the effects of Boc concentration and steric bulk, we synthesized a control framework, CCOF-DACH-Boc (SI, Sections 2.7 and 2.8). Both CCOF0-Boc and CCOF-DACH-Boc share the same framework topology and were synthesized under identical Boc group concentrations and reaction conditions (100 °C). However, they differ in their chiral sulfonamide functionalities: CCOF0-Boc contains the bulky (1*R*,2*R*)-1,2-diphenylethane-1,2-diamine derivative, whereas CCOF-DACH-Boc contains the less sterically demanding (1*R*,2*R*)-cyclohexane-1,2-diamine derivative. As established in the literature (*J. Am. Chem. Soc.* **2023**, *145*, 21798-21806; *J. Am. Chem. Soc*. **2017**, *139*, 10506-10513; *Adv. Mater.* **2020**, *32*, 2002366), increasing the steric hindrance of substituents generally lowers the crystallinity of the resulting COF. Our PXRD patterns clearly demonstrate that CCOF-DACH-Boc exhibits significantly higher crystallinity than CCOF0-Boc. This control experiment strongly suggests that

acidic deprotection is not the dominant factor; rather, from a crystallization thermodynamics and kinetics perspective, the 100% incorporation of highly sterically demanding diphenyl-based chiral groups in CCOF0-Boc creates severe intrapore steric repulsion. This restricts the conformational freedom of the building blocks and hinders the reversible "error-correction" process essential for dynamic covalent framework crystallization, leading to lower long-range order.

This inherent structural limitation of CCOF0-Boc precisely motivated our design of the multivariate approach. By reducing the density of these bulky chiral functionalities and introducing co-monomers in MCCOF-Boc, we effectively alleviate the internal steric strain. This multivariate approach not only restores the dynamic reversibility required for high crystallinity and structural stability but also creates an optimized, uncrowded microenvironment for the subsequent catalytic transformations.

Manuscript Change:

We have significantly expanded the discussion in the main text (Section: Synthesis and Characterization) to explicitly address the thermodynamic/steric challenges of crystallizing fully functionalized CCOF0-Boc. We have also updated the text to highlight how the structural comparison with CCOF-DACH-Boc (SI, Sections 2.7 and 2.8) validates the multivariate approach as a necessary and effective strategy to overcome steric penalties and achieve high crystallinity.

Manuscript (revised text on page 7, Second paragraph):

The relatively low BET surface areas of CCOF0-Boc and CCOF1-Boc can be attributed to the steric hindrance induced by the bulky Boc-protected chiral moieties. These sterically demanding groups not only occupy the internal pore volume but also likely induce interlayer sliding or staggered stacking to minimize steric repulsion, leading to reduced crystallinity and partially blocked channels.[61, 62] This steric-driven limitation is further corroborated by our control experiment with CCOF-DACH-Boc (Supplementary Fig. 24), which demonstrates that reducing the steric bulk of the chiral groups significantly restores crystallinity under identical synthetic conditions. In stark contrast, the multivariate approach in MCCOF-Boc effectively alleviates this interlayer steric tension, promoting a more ordered framework stacking. This structural optimization is directly reflected in its significantly enhanced crystallinity and substantially higher BET surface area.

Reference:

61. Pelkowski CE, *et al.* Tuning Crystallinity and Stacking of Two-Dimensional

Covalent Organic Frameworks through Side-Chain Interactions. *J. Am. Chem. Soc.* **145**, 21798-21806 (2023).

62. Thompson CM, *et al.* Computational and Experimental Studies on the Effects of Monomer Planarity on Covalent Organic Framework Formation. *J. Am. Chem. Soc.* **139**, 10506-10513 (2017).

Supporting Information (revised synthetic scheme for CCOF-DACH-Boc, page 32; revised Supplementary Fig. 24, page 50):

Synthesis of CCOF-DACH-Boc

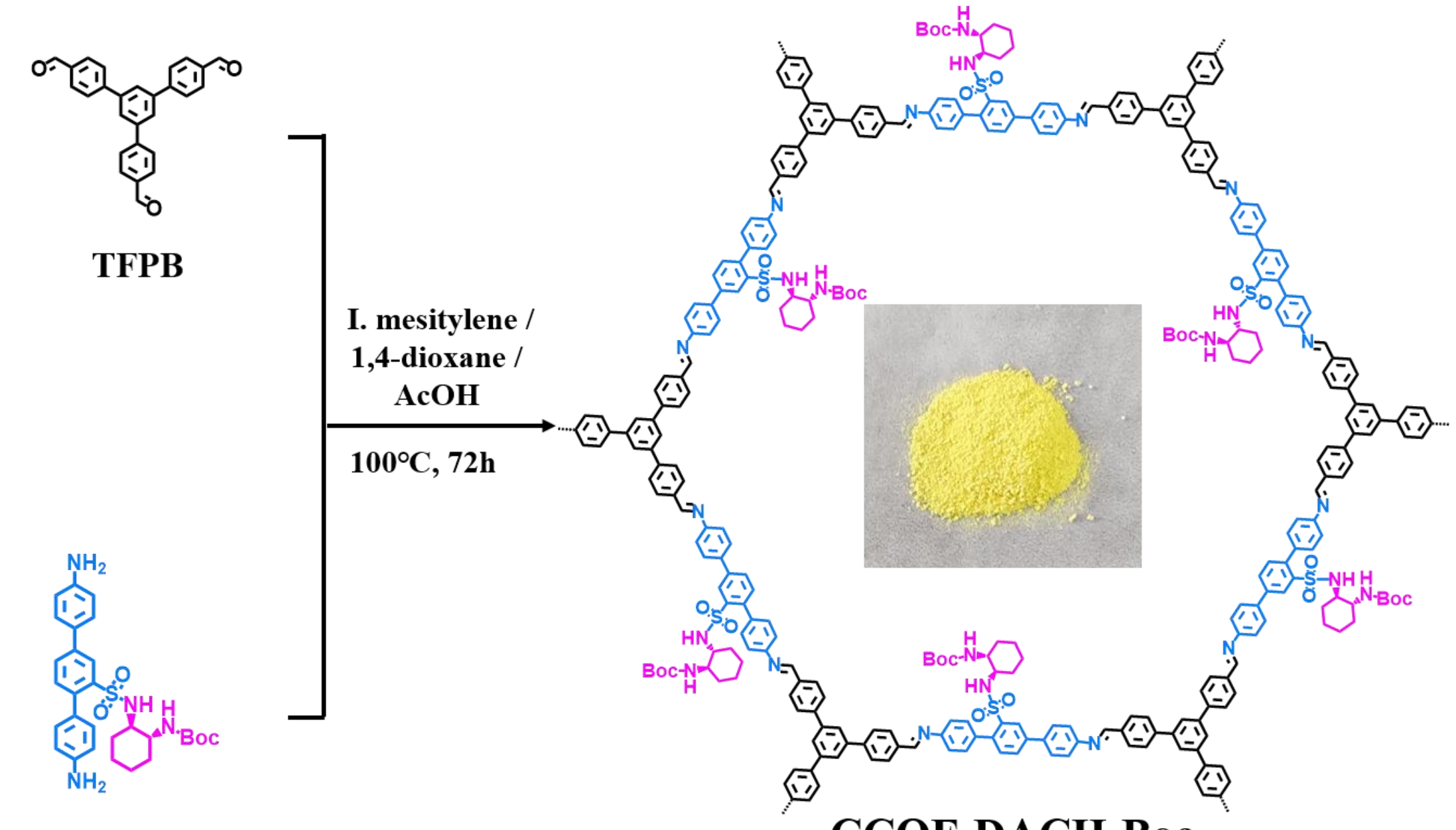

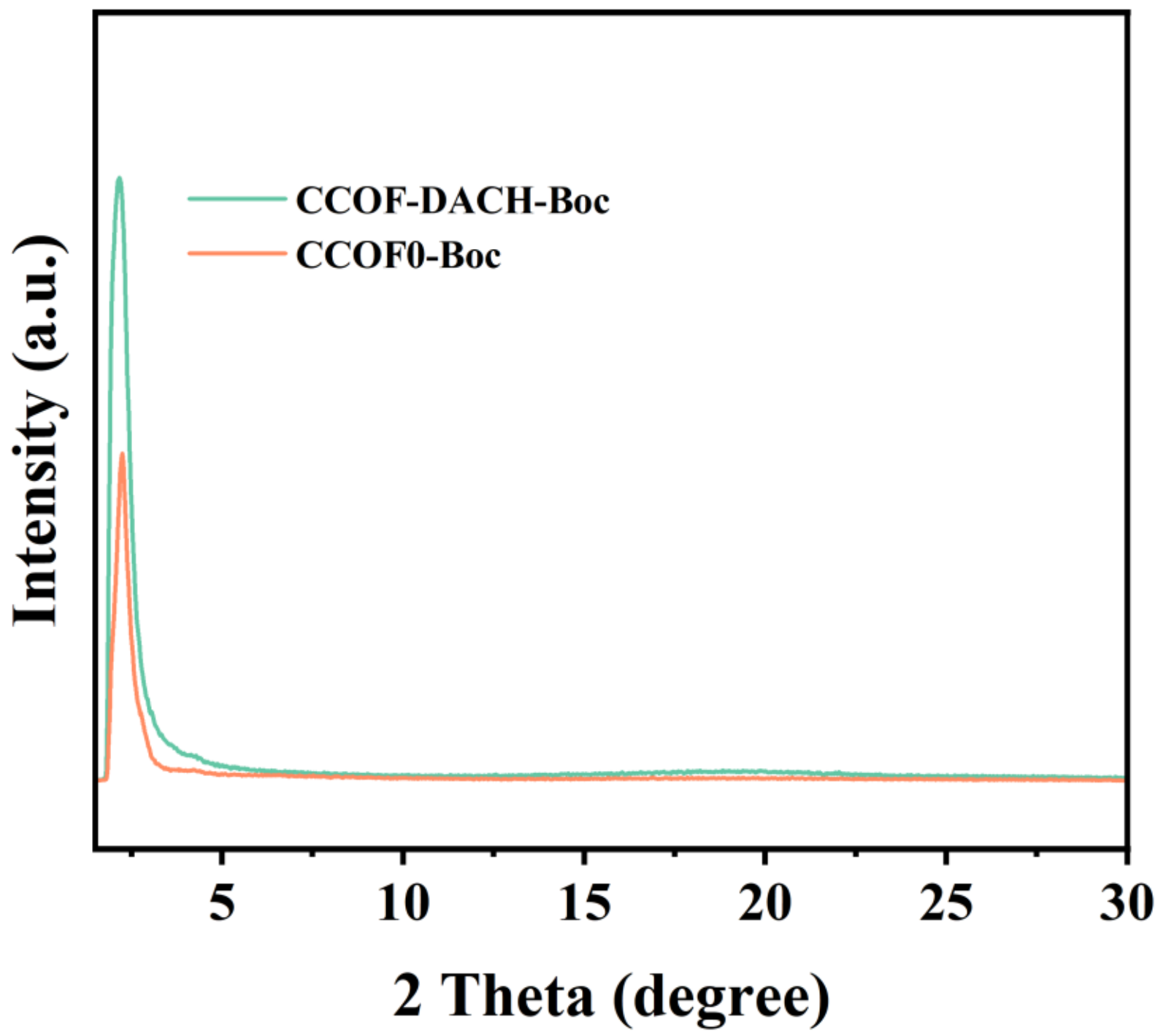

Supplementary Fig. 24 PXRD patterns of Boc-protected CCOF0-Boc and CCOF-DACH-Boc synthesized at 100 °C. The patterns reveal that CCOF-DACH-Boc exhibits significantly higher crystallinity than CCOF0-Boc. Both frameworks share the same topology and were prepared under identical conditions, including the same Boc group concentration and a reaction temperature of 100 °C. Hence, the lower crystallinity of CCOF0-Boc is more likely attributable to greater steric hindrance from its substituents, which impairs COF crystallinity.

Comment 2: The authors claim that the dense chiral diamine ligands can "recapture dissociated Ru species." Please provide evidence to support this hypothesis;

Response: We sincerely thank the reviewer for highlighting this important mechanistic point. While directly observing the dynamic "dissociation-and-recapture" process in situ is technically challenging, we have provided a convergence of stoichiometric, spectroscopic, and catalytic evidence to strongly substantiate this hypothesis in our revised manuscript.

Based on our ICP-OES analysis (0.83 wt% Ru in CCOF2-Ru), we calculated the exact stoichiometric ratio of Ru to the chiral diamine ligands, revealing that only approximately 2 out of every 13 chiral diamine sites are coordinated with Ru. This leaves a massive excess (~85%) of uncoordinated, structurally identical chelating sites densely distributed along the 1D channels of the COF. Furthermore, the straightforward synthesis of CCOF2-Ru (stirring at 40 °C without additional base) and subsequent characterizations via XPS (Fig. 4a,b), EXAFS (Fig. 4d,e), and TEM-EDS mapping (Supplementary Fig. 10) confirm the facile and uniform coordination of Ru to these sites. Because this coordination is thermodynamically favorable and kinetically rapid, any Ru species that transiently dissociates during the catalytic cycle will immediately encounter a highly concentrated local environment of free bidentate chelating sites within the confined nanopores, driving rapid re-coordination.

The most compelling macroscopic evidence for this recapture mechanism lies in our leaching and recyclability data. As shown in Fig. 5f, the catalyst maintains its high activity and enantioselectivity over five consecutive cycles. Additionally, hot filtration tests afforded only trace amounts of the product in the filtrate, indicating negligible Ru leaching (Supplementary Table 11). If the dissociated Ru species were not effectively recaptured by the abundant adjacent free ligands, severe macroscopic metal leaching and a rapid decline in catalytic performance would inevitably occur. Maintaining such a high ratio of uncoordinated anchoring sites to metal-coordinated sites is a well-established and highly effective strategy in heterogeneous catalysis to create a "sponge effect" that suppresses metal leaching (*J. Am. Chem. Soc.* **2024**, *146*, 27736; *ACS Catal.*

2017, *7*, 6711). Our multi-faceted data perfectly aligns with this mechanism.

Manuscript Change:

We have revised the manuscript (Section: Metallization of CCOFs and catalytic performance) to explicitly present this multi-tiered evidence. We detailed the stoichiometric ratio of coordinated vs. uncoordinated sites based on ICP data, correlated this with the negligible Ru leaching observed in our catalytic recycling tests, and added the relevant literature citations to firmly support the recapture mechanism.

Manuscript (revised text on page 17, first paragraph):

Based on our ICP-OES analysis, a massive excess (~85%) of uncoordinated, structurally identical chelating sites densely distributed along the 1D channels of the COF. The synthesis of CCOF2-Ru is straightforward (stirring at 40°C without additional base), XPS (Fig. 4a,b) and EXAFS (Fig. 4d,e) alongside TEM-EDS mapping (Supplementary Fig. 15f) confirms the facile and uniform coordination of Ru to these sites. Because the initial coordination is thermodynamically favorable and kinetically rapid, any Ru species that transiently dissociates during the catalytic cycle will immediately encounter a highly concentrated local environment of these free bidentate chelating sites within the confined nanopores, driving rapid re-coordination.

Comment 3: In the XRD pattern of CCOF0-Boc, the (100) reflection is strong while the (001) reflection is absent, yet the HRTEM image only shows lattice fringes corresponding to the (001) plane (0.48 nm spacing). This differs from the HRTEM results of CCOF1-Boc and CCOF2-Boc. Please clarify this discrepancy;

Response: We sincerely thank the reviewer for this highly observant and professional comment. The apparent discrepancy between the XRD and HRTEM results for CCOF0-Boc is not a contradiction, but rather a classic manifestation of the fundamental geometric differences between X-ray diffraction (reflection mode) and HRTEM (transmission mode), compounded by the anisotropic morphology and electron-beam sensitivity of the sample.

In standard powder XRD (Bragg-Brentano geometry), the detected signal originates from crystallographic planes that are parallel to the sample substrate. Conversely, in HRTEM, the electron beam transmits vertically through the sample grid, meaning it visualizes lattice fringes of planes that are parallel to the electron beam (i.e., perpendicular to the substrate). As shown in the SEM images (Fig. 2f), CCOF0-Boc forms urchin-like aggregates composed of smaller anisotropic structures. When these particles are deposited onto the XRD holder or TEM grid, they tend to lie flat.

Consequently, the (100) planes are oriented parallel to the substrate, resulting in a strongly enhanced (100) reflection in XRD due to preferred orientation (texturing). However, because these same (100) planes are perpendicular to the vertical electron beam in TEM, their lattice fringes are geometrically difficult to capture unless the particle is specifically tilted. Instead, the (001) planes, which are orthogonal to the substrate, align perfectly with the electron beam, making the (001) fringes (0.48 nm) readily visible in HRTEM.

Beyond geometric orientation, differential electron beam sensitivity further contributes to this observation. CCOF0-Boc has relatively lower crystallinity compared to CCOF1/2-Boc. In COF structures, the (100) planes represent the porous and fragile in-plane channel structures, whereas the (001) planes represent the dense, strongly interacting π–π stacking layers. Under high-energy electron beam irradiation, the porous (100) channels of the less crystalline CCOF0-Boc are susceptible to rapid structural degradation (knock-on damage). While we did sporadically observe faint, larger-spacing (100) fringes locally, they amorphized too quickly to capture high-quality images. The dense (001) stacking, however, is significantly more robust against beam damage, allowing for clear observation; therefore, to ensure absolute data reliability, we only annotated the unambiguous (001) fringes. Similar phenomena have been observed in previous studies, such as the spherical COF reported by Li et al., which exhibited only a clear (100) peak in PXRD but showed distinct (001) lattice fringes in HRTEM (*ACS Appl. Mater. Interfaces* 2024, *16*, 59379–59387).

In contrast, CCOF1-Boc and CCOF2-Boc possess significantly higher crystallinity and adopt robust block-like morphologies (Supplementary Fig. 8c,e). Their high structural order drastically improves their stability under the electron beam, and their blocky, less anisotropic shapes result in more random orientations on the TEM grid. This combination allows the (100) planes to survive the electron beam and be consistently captured, as shown in Fig. 3i,j.

Manuscript Change:

We deeply appreciate the reviewer for pointing this out. We have added a detailed crystallographic and morphological explanation in the SI (Supplementary Fig. 5) to clarify the geometric orientation effects in XRD vs. HRTEM, as well as the beam sensitivity considerations for CCOF0-Boc.

Supporting Information (revised Supplementary Fig. 5):

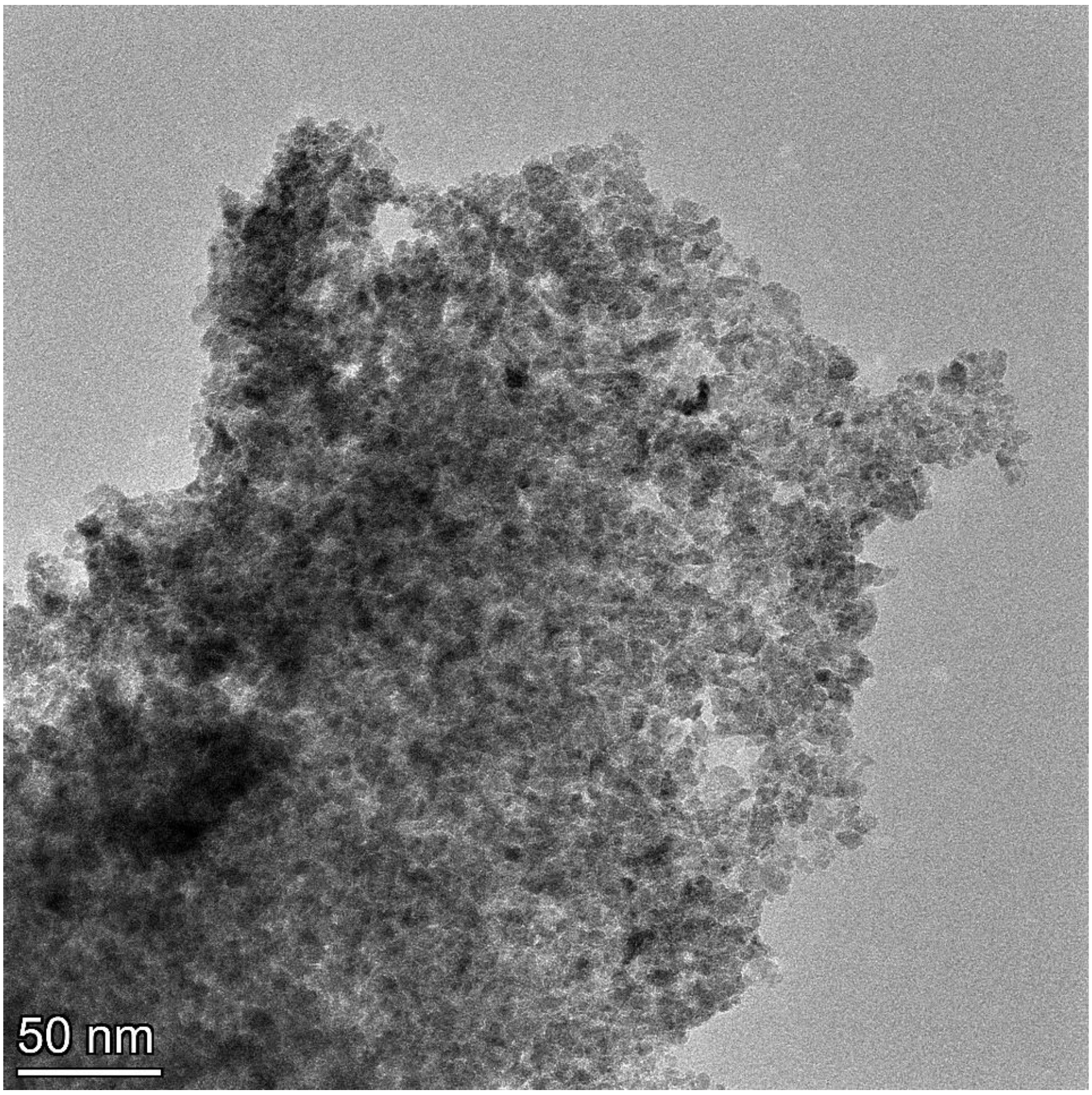

Supplementary Fig. 5 TEM images of CCOF0-Boc.

TEM images further revealed that CCOF0-Boc is composed of smaller anisotropic structures. When these particles are deposited onto the XRD holder or TEM grid, they tend to lie flat. Consequently, the (100) planes are oriented parallel to the substrate, resulting in a strongly enhanced (100) reflection in XRD (preferred orientation/texturing). However, because these same (100) planes are perpendicular to the vertical electron beam in TEM, their lattice fringes are geometrically difficult to capture unless the particle is specifically tilted. Instead, the (001) planes (which are orthogonal to the substrate) align perfectly with the electron beam, making the (001) fringes (0.48 nm) readily visible in HRTEM (Fig. 2e). Furthermore, CCOF0-Boc has relatively lower crystallinity compared to CCOF1/2-Boc. In COF structures, the (100) planes represent the porous and fragile in-plane channel structures, whereas the (001) planes represent the dense, strongly interacting π–π stacking layers. Under high-energy electron beam irradiation, the porous (100) channels of the less crystalline CCOF0-Boc are susceptible to rapid structural degradation (knock-on damage). While we did sporadically observe faint, larger-spacing (100) fringes locally, they amorphized too quickly to capture high-quality images. The dense (001) stacking, however, is

significantly more robust against beam damage, allowing for clear observation. To ensure data reliability, we only annotated the unambiguous (001) fringes.

Comment 4: The statement "Thermogravimetric analysis (TGA) demonstrated thermal stability, with ~200 °C mass loss in Boc-protected CCOFs attributed to deprotection, absent in deprotected analogues (Supplementary Fig. 6), verifying complete Boc removal" is unclear. Please rephrase or explain;

Response: We apologize for the lack of clarity. We have reanalyzed the TGA data and revised the text to clearly delineate the distinct thermal degradation stages. The initial mass loss around 210 °C in Boc-protected samples corresponds to the thermal cleavage of the Boc groups. A subsequent significant weight reduction occurs near 275 °C, which aligns with the known thermal decomposition temperature of sulfonamide moieties (*Sep. Purif. Technol.* **2001**, *22*, 209). Finally, pronounced mass loss begins at 480 °C, indicative of total framework collapse, which is consistent with the TGA profile of the non-functionalized TFPB-DATP framework.

Manuscript Change

Manuscript (revised text on page 8, first paragraph):

The TGA discussion (Page 8) has been rewritten for clarity: "Thermogravimetric analysis (TGA) was also performed to evaluate the thermal stability of the CCOFs. As shown in Supplementary Fig. 9, the Boc-protected CCOF samples exhibit an obvious mass loss starting at approximately 210 °C, attributed to thermal deprotection of the Boc groups. All samples display a significant weight reduction at around 275 °C, likely indicating the decomposition of the sulfonated clusters.[65,66] Furthermore, a pronounced mass loss begins at 480 °C, attributable to framework collapse." Relevant references have been added.

Supporting Information (revised Supplementary Fig. 9, page 42):

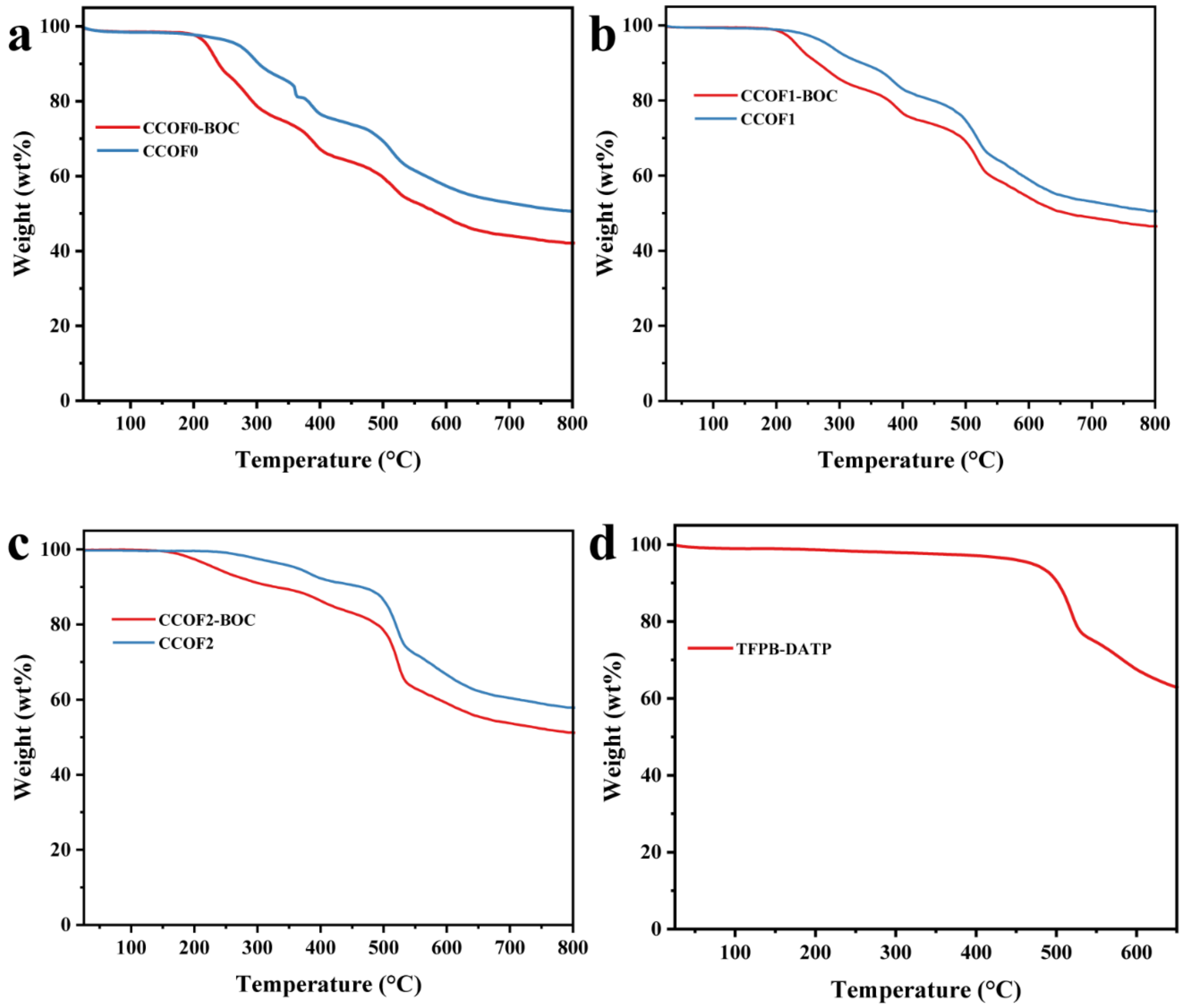

Supplementary Fig. 9 TGA curves of **a** CCOF0-Boc and CCOF0, **b** CCOF1-Boc and CCOF1, **c** CCOF2-Boc and CCOF2, and **d** TFPB-DATP. After thermal analysis, the residual samples in the crucibles transformed into black powders, likely due to the carbonization of the COFs at high temperature under nitrogen flow.

Reference:

65. Qi Y, Jin X, Yu C, Wang Y, Yang L, Li Y. A novel chelating resin containing high levels of sulfamine group: Preparation and its adsorption characteristics towards p-toluenesulfonic acid and Hg(II). *Chem. Eng. J.* **233**, 315-322 (2013).
66. Charradi k, *et al.* Incorporation of multilayered double hydroxides/sepiolite augments proton conductivity performance in low sulfonated polyether sulfone octyl sulfonamide. *Mater Renew Sustain Energy.* **13**, 97-107 (2024).

Comment 5: After treatment with 1 M KOH, the XRD peak of CCOF1 appears sharper (narrower FWHM), suggesting improved crystallinity. Similarly, treatment with 1 M HCOONa seems to sharpen the XRD peaks of CCOF2. What is the reason for this?

Response: We appreciate the reviewer's meticulous attention to the data. We have

carefully re-examined the XRD profiles before and after alkaline treatment. The peak positions (2θ ≈ 2.18°) and integrated intensities remain essentially unchanged, indicating that the bulk crystallinity is preserved rather than fundamentally altered. The slight perceived sharpening (narrower FWHM) is an artifact of minor variations in measurement conditions (e.g., sample surface flatness during preparation, instrument calibration status on different days), rather than a genuine structural enhancement. We consider these minor differences to fall within the typical experimental variance of powder XRD for soft porous materials.

Comment 6: Please clearly label the peak positions in the XRD patterns of CCOF1, CCOF2 and their corresponding Ru-loaded analogues (CCOF1-Ru and CCOF2-Ru);

Response: We agree with this helpful suggestion. We have updated the figures to clearly label the corresponding peak positions (2θ values).

Manuscript Change:

The peak positions have been labeled in the revised XRD patterns. To improve the flow of the main text, the original Fig. 4d has been moved to Supplementary Fig. 13.

Supporting Information (revised Supplementary Fig. 13, page 44):

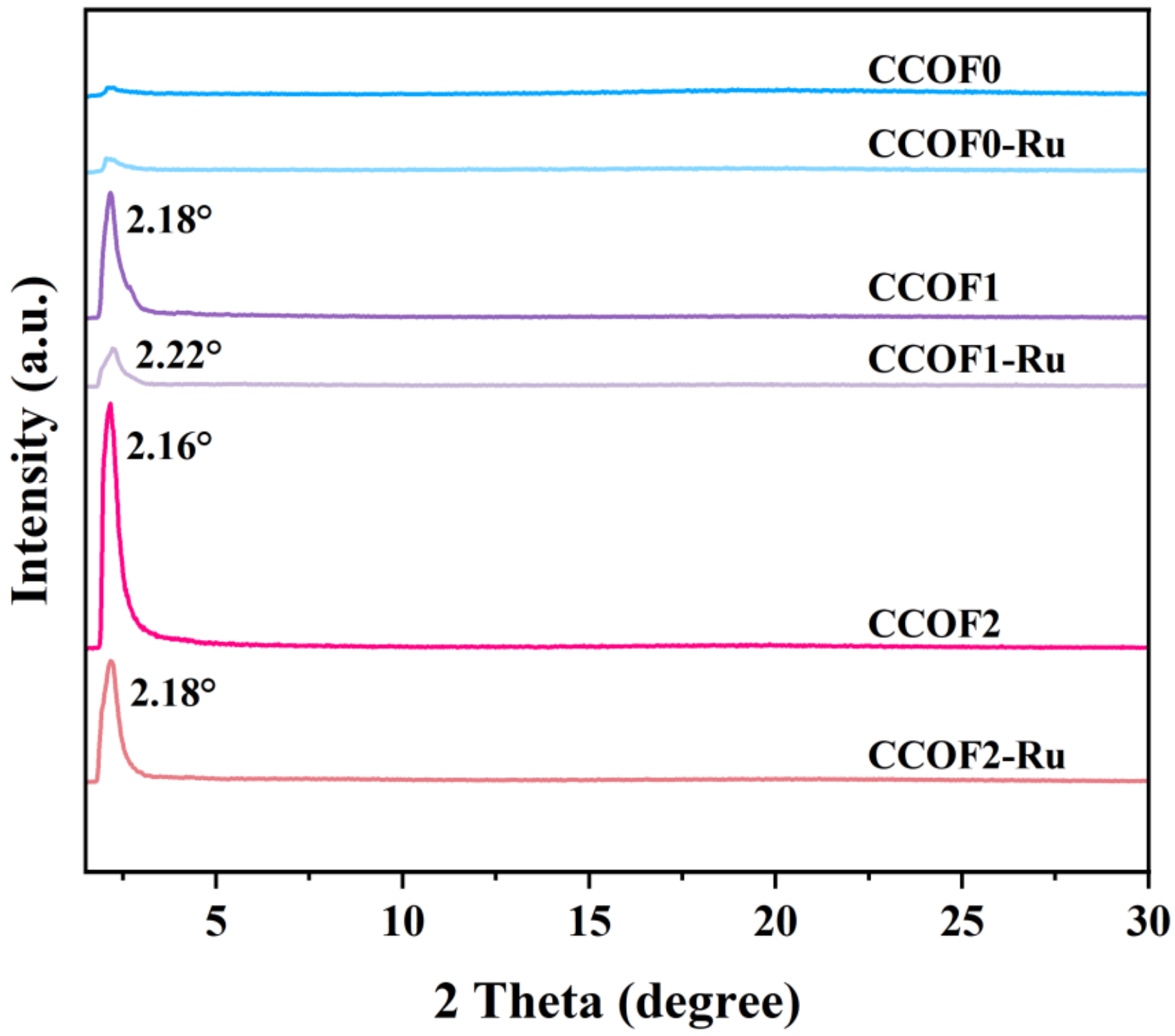

Supplementary Fig. 13 PXRD patterns of the CCOFX and CCOFX-Ru.

Comment 7: In the N 1s XPS spectra, why is the N-H signal much stronger in (R)-Ph-

Ru than in CCOF2-Ru? Why is there no clear metal-N signal? Please supplement with Raman spectroscopy to verify M-N bonding;

Response: We sincerely thank the reviewer for this highly insightful and constructive comment, which prompted us to re-evaluate our XPS analysis and perform the requested Raman characterization. Regarding the apparent intensity difference of the N-H signal between (*R*)-Ph-Ru and CCOF2-Ru, we would like to clarify that this is an artifact of data presentation rather than a quantitative difference in chemical composition. In the original Fig. 4f, the N 1s XPS spectra were presented as stacked plots with independent intensity scales (arbitrary units) to optimize visual clarity, making the apparent peak heights not directly comparable. Furthermore, we deeply appreciate the reviewer pointing out the ambiguity of the metal-N signal. To ensure accuracy and eliminate any subjective fitting artifacts, we acquired the XPS spectrum of a well-defined, commercially available complex, RuCl(*p*-cymene)[(*R*,*R*)-Ts-DPEN], as a standard reference. Using this rigorous standard, we identified two distinct peaks at 399.9 eV and 398.1 eV, corresponding unambiguously to the N-H and Ru-N species, respectively. Based on this reliable reference, we have accurately re-fitted our CCOF2-Ru N 1s spectra. The revised deconvolution (now updated in Fig. 4b) clearly reveals the Ru-N signal at 398.1 eV, providing solid spectroscopic evidence for the successful coordination.

Following your valuable suggestion, we also performed Raman spectroscopy on CCOF2-Ru to further verify the M-N bonding (Supplementary Fig. 17b). Unfortunately, no distinct Ru-N characteristic peak was observed. We attribute this absence to two fundamental physical limitations that are well-supported by recent literature. First, the Ru content in CCOF2-Ru is extremely low (only 0.83 wt%), which is likely below the detection limit of conventional Raman spectroscopy. Second, Ru-N species inherently exhibit a very low Raman scattering cross-section. For instance, in a recent study on a Ru-coordinated COF (*Nat. Commun.* **2024**, *15*, 5419) with a much higher Ru loading (6.8 wt%), no Ru-N peaks could be detected in either conventional or in situ Raman spectra, even though the Ru-N bonds were definitively confirmed by synchrotron XANES. Therefore, the combination of our ultra-low Ru loading and the intrinsically weak Raman activity of Ru-N bonds accounts for this absence. Nevertheless, we believe our standard-calibrated XPS data now provides robust and definitive evidence for the Ru-N coordination.

Manuscript Change:

We have updated the high-resolution N 1s XPS spectra (Fig. 4b) based on the commercial reference complex. A detailed discussion regarding the rigorous XPS

assignments and the scientific rationale for the Raman results (Supplementary Fig. 17b) has been added to the main text.

Manuscript (revised text, page 10):

"The high-resolution N 1s spectrum of CCOF2-Ru reveals that the C=N signal at 398.8 eV remains unchanged compared to that of CCOF2 (Fig. 4b). Upon Ru coordination, a new signal emerges at 398.1 eV, corresponding to the Ru–N species, alongside a feature at approximately 399.9 eV attributed to N–H. To ensure the accuracy of this assignment, we measured a commercially available RuCl(*p*-cymene)[(*R*,*R*)-Ts-DPEN] complex as a standard reference, which exhibited identical binding energies for the Ru-N and N-H species."

"The Raman spectra (Supplementary Fig. 17b) confirm that CCOF2-Ru retains the same framework structure as CCOF2 after Ru coordination. Furthermore, the characteristic Ru-N peak was not observed. This is consistent with recent literature[71] and is attributed to the ultra-low Ru loading (0.83 wt%) combined with the inherently low Raman scattering cross-section of Ru-N bonds."

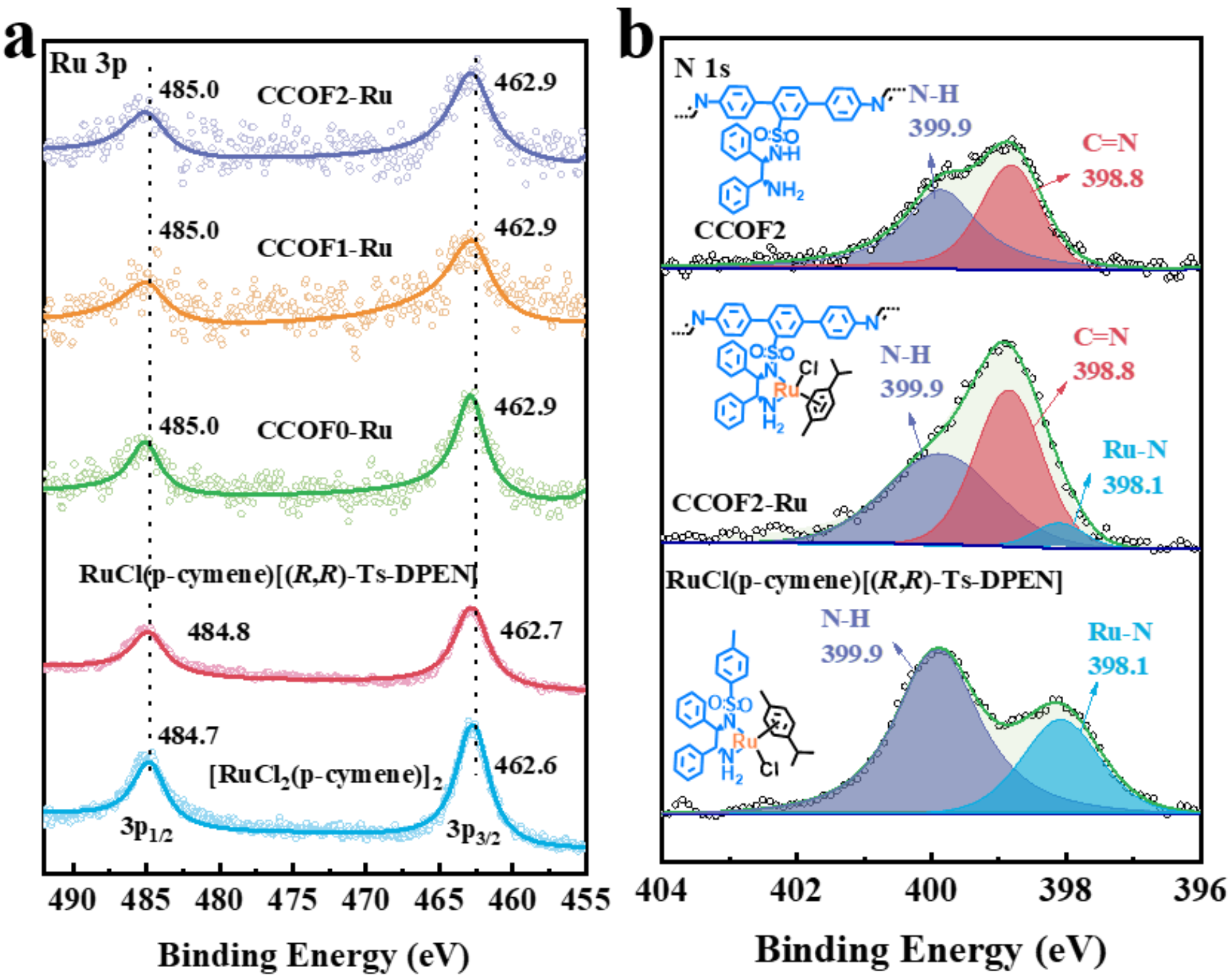

Fig. 4 High-resolution **a** Ru 3p and **b** N 1s XPS spectra of CCOF2-Ru.

Supporting Information (revised Supplementary Fig. 17b, page 46):

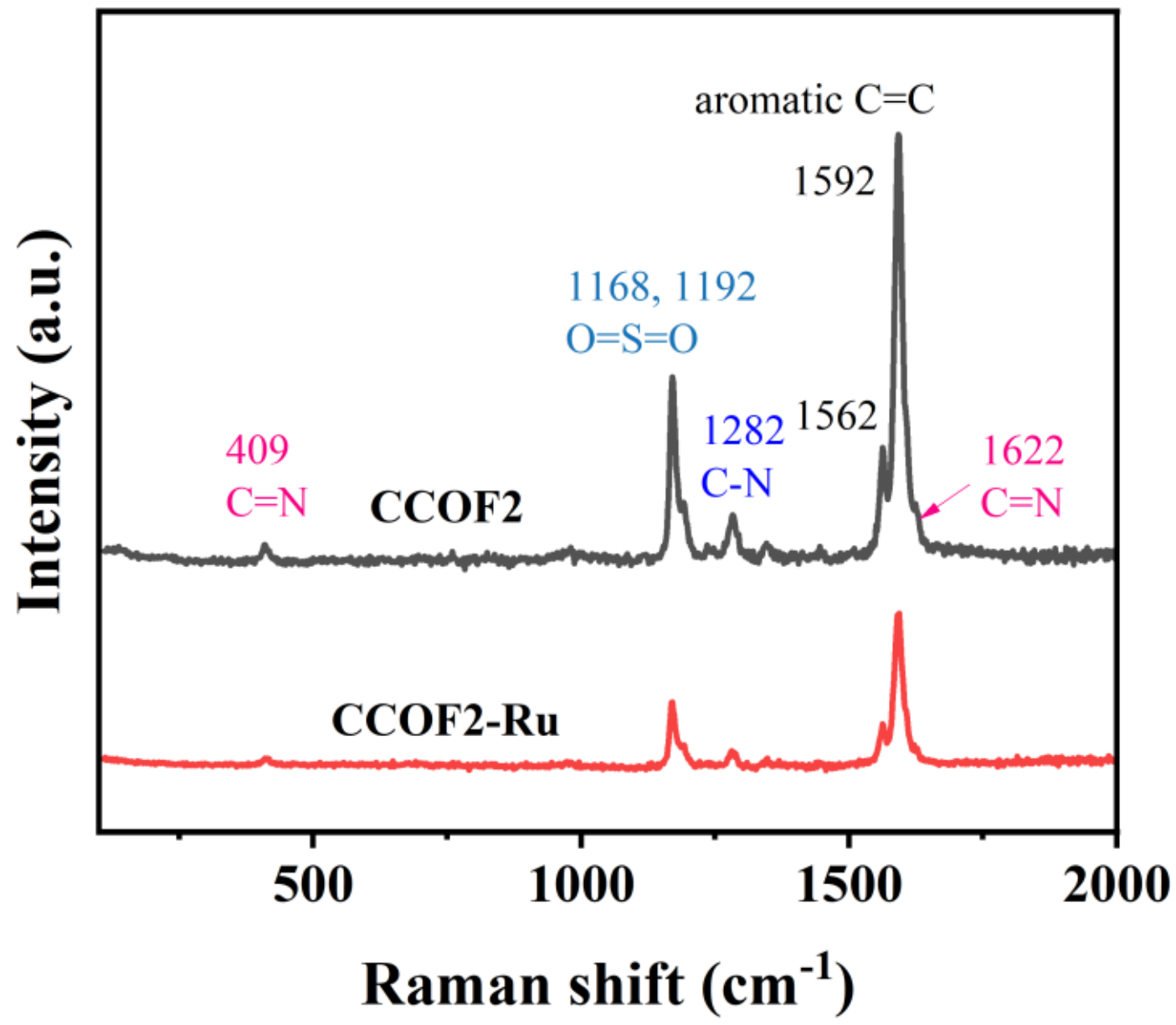

Supplementary Fig. 17b Raman spectra of CCOF2 and CCOF2-Ru.

Reference:

71. Jia H, Yao N, Jin Y, Wu L, Zhu J, Luo W. Stabilizing atomic Ru species in conjugated sp2 carbon-linked covalent organic framework for acidic water oxidation. *Nat. Commun*. **15**, 5419 (2024).

Comment 8: There appear to be significant errors in the synthetic schemes for compound 5 and (R)-Ph in Section 2 (Synthetic procedures);

Response: We sincerely thank the reviewer for catching this oversight. The molecular structures depicted for Compound 5 and (*R*)-Ph were indeed incorrect in the original draft. We have thoroughly corrected all synthetic schemes in Section 2 of the Supporting Information. Structures and labels have been fixed, reaction conditions added, and compound names clarified.

Manuscript Change:

Supporting Information (revised synthetic schemes for (*R*,*R*)-DACH-NH-Boc, (*R*,*R*)-TP-DPEN and (*R*,*R*)-DACH-NH-Boc):

Synthetic schemes for (*R*,*R*)-DACH-NH-Boc and (*R*,*R*)-TP-DPEN

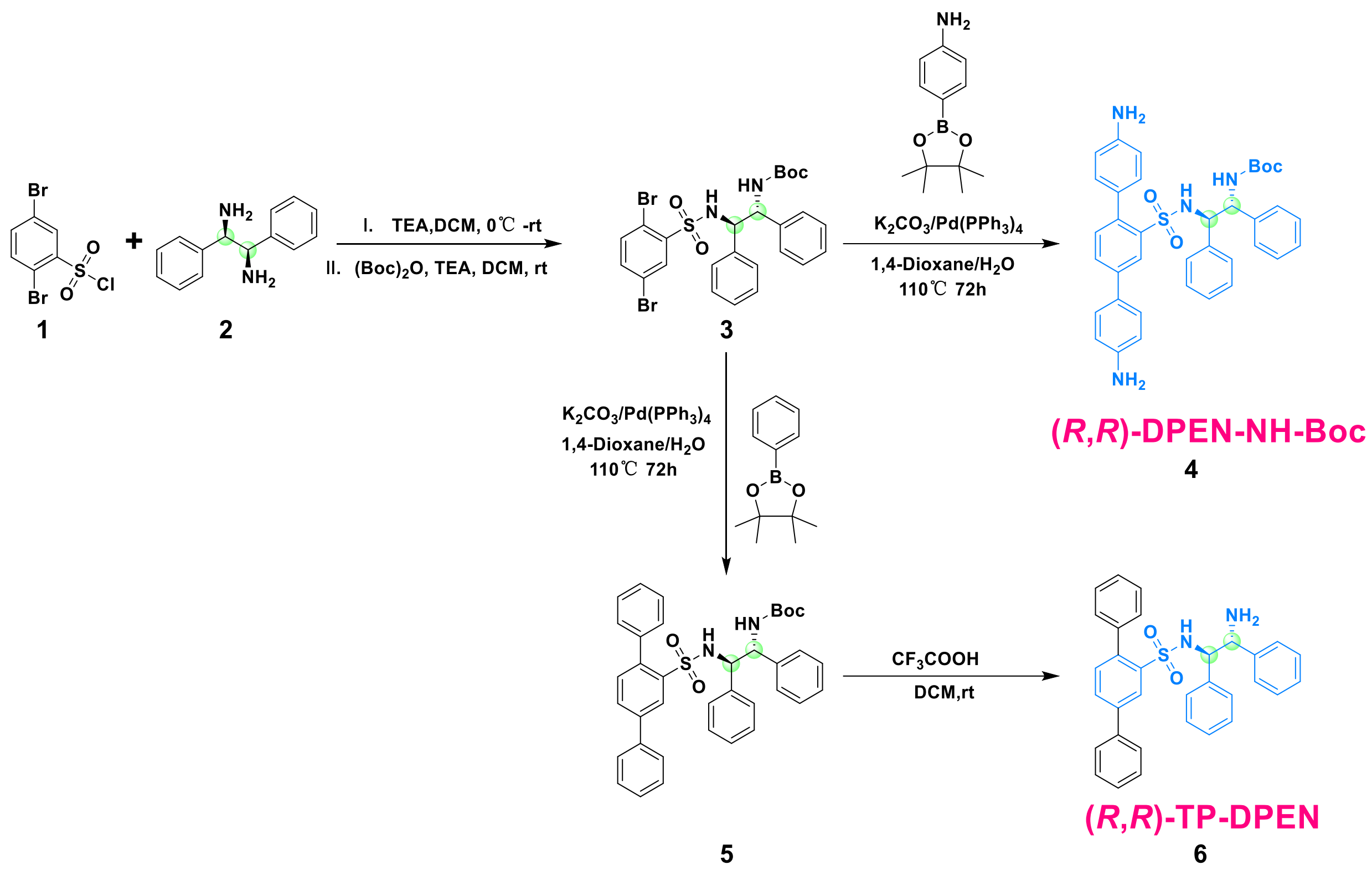

Synthetic schemes for (*R*,*R*)-DACH-NH-Boc

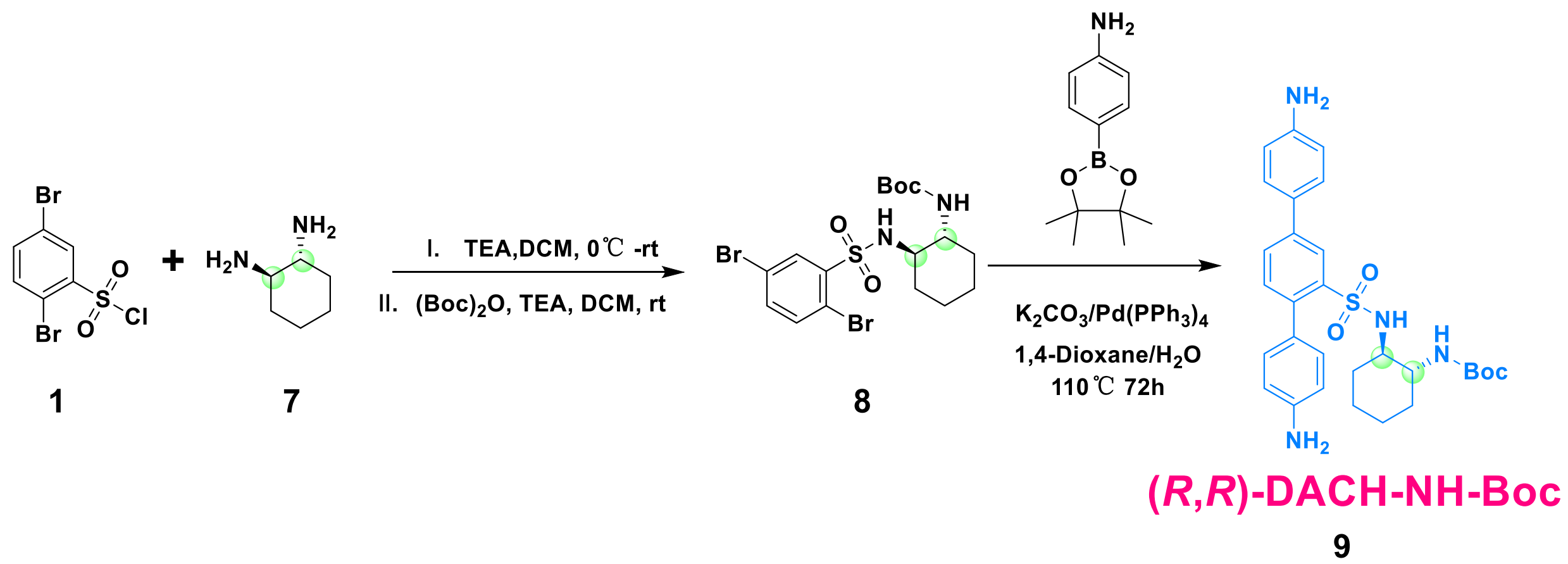

Comment 9: CCOF0-Boc was synthesized at 100°C for 3 days, while CCOF1-Boc and CCOF2-Boc were synthesized at 120°C for 3 days. Could the lower crystallinity of CCOF0-Boc be related to the reaction temperature? Would synthesis at 120°C improve its crystallinity?

Response: We appreciate the reviewer's logical query. During our initial synthetic optimization, we did attempt to synthesize CCOF0-Boc at 120 °C. Interestingly, the crystallinity of the 120 °C sample was significantly lower than that obtained at 100 °C. Conversely, CCOF1-Boc and CCOF2-Boc exhibited optimal crystallinity at 120 °C. Therefore, the lower crystallinity of CCOF0-Boc is an inherent property of its dense chiral functionalization (as discussed in Comment 1), and raising the temperature to

120 °C proved detrimental rather than beneficial.

Manuscript Change:

Supporting Information (revised Supplementary Fig. 1):

We have added a brief note in the SI detailing these temperature optimization trials to justify the chosen synthetic conditions.

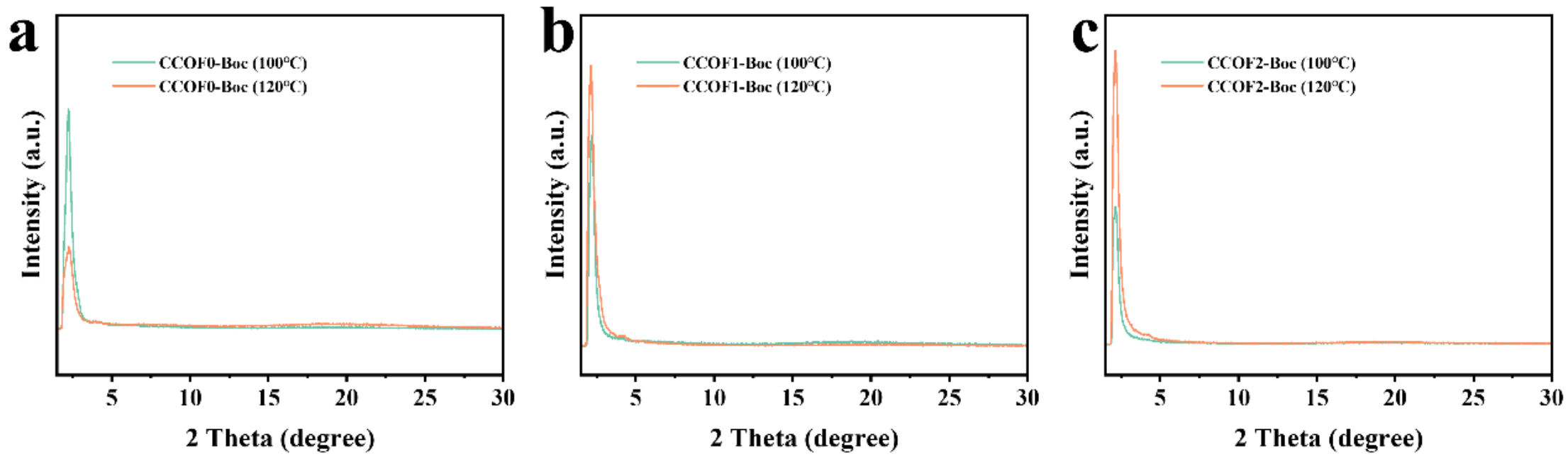

Supplementary Fig. 1 PXRD patterns of Boc-protected CCOFX-Boc synthesized at 100 °C and 120 °C. CCOF0-Boc: higher crystallinity at 100 °C; CCOF1-Boc and CCOF2-Boc: higher crystallinity at 120 °C. The retention of a substantial amount of Boc groups in CCOF1-BOC and CCOF2-BOC was confirmed by FTIR (Supplementary Fig. 2, the C=O peak of Boc at 1694 cm^{-1}), solid-state NMR (Supplementary Fig. 3, δ = 80, 27 ppm) and TGA curves (Supplementary Fig. 9, an obvious mass loss starting at approximately 210 °C). These results verify that the employed solvothermal conditions do not significantly affect the synthesis of the CCOFs.

Comment 10: In Supplementary Fig. 11 (FTIR spectra of CCOFX-Ru), please label the characteristic peaks. is there C=O signal remain?

Response: We thank the reviewer for this careful observation and valuable suggestion. We have updated the FTIR spectra (now Supplementary Fig. 17a) to provide a clearer visualization, with all characteristic peaks explicitly labeled. Regarding your specific query, a distinct C=O stretching peak at 1694 cm^{-1} is indeed present in the spectra. We understand your underlying concern might be whether this signal indicates the incomplete removal of the Boc protecting groups. To clarify this, we rely on our solid-state ^{13}C CP/MAS NMR spectra, which provide definitive structural proof. While FTIR is highly sensitive to C=O bonds, it cannot easily distinguish the exact chemical environment of the carbonyl. Our NMR data, however, clearly show the complete disappearance of the characteristic signals for the *tert*-butyl and carbonyl carbons of the Boc group. This orthogonal evidence confirms that the Boc deprotection was successful and that the C=O signal in the FTIR spectra does not originate from residual Boc groups.

Since the Boc groups are completely removed, we assign this 1694 cm^{-1} C=O stretching peak to terminal aldehyde groups. In the field of imine-linked COF chemistry, it is widely recognized and well-documented that macroscopic COF crystallites possess abundant external edge sites and internal grain boundaries, which inevitably terminate with unreacted monomer functional groups such as aldehydes (*Angew. Chem. Int. Ed.* **2024**, *63*, e202412420; *Nat. Commun.* **2022**, *13*, 1770; *Angew. Chem. Int. Ed.* **2024**, *63*, e202412643). Therefore, the presence of this C=O signal represents the natural edge and defect sites of the macroscopic COF particles rather than incomplete deprotection. It is a common feature in bulk COF materials and does not compromise the overall framework integrity or the subsequent catalytic performance.

Manuscript Change:

The updated FTIR spectra with labeled peaks are provided in Supplementary Fig. 17a, and the assignment of the 1694 cm^{-1} peak to terminal aldehydes has been explicitly stated in the text.

Supporting Information (revised Supplementary Fig. 17a, page 46):

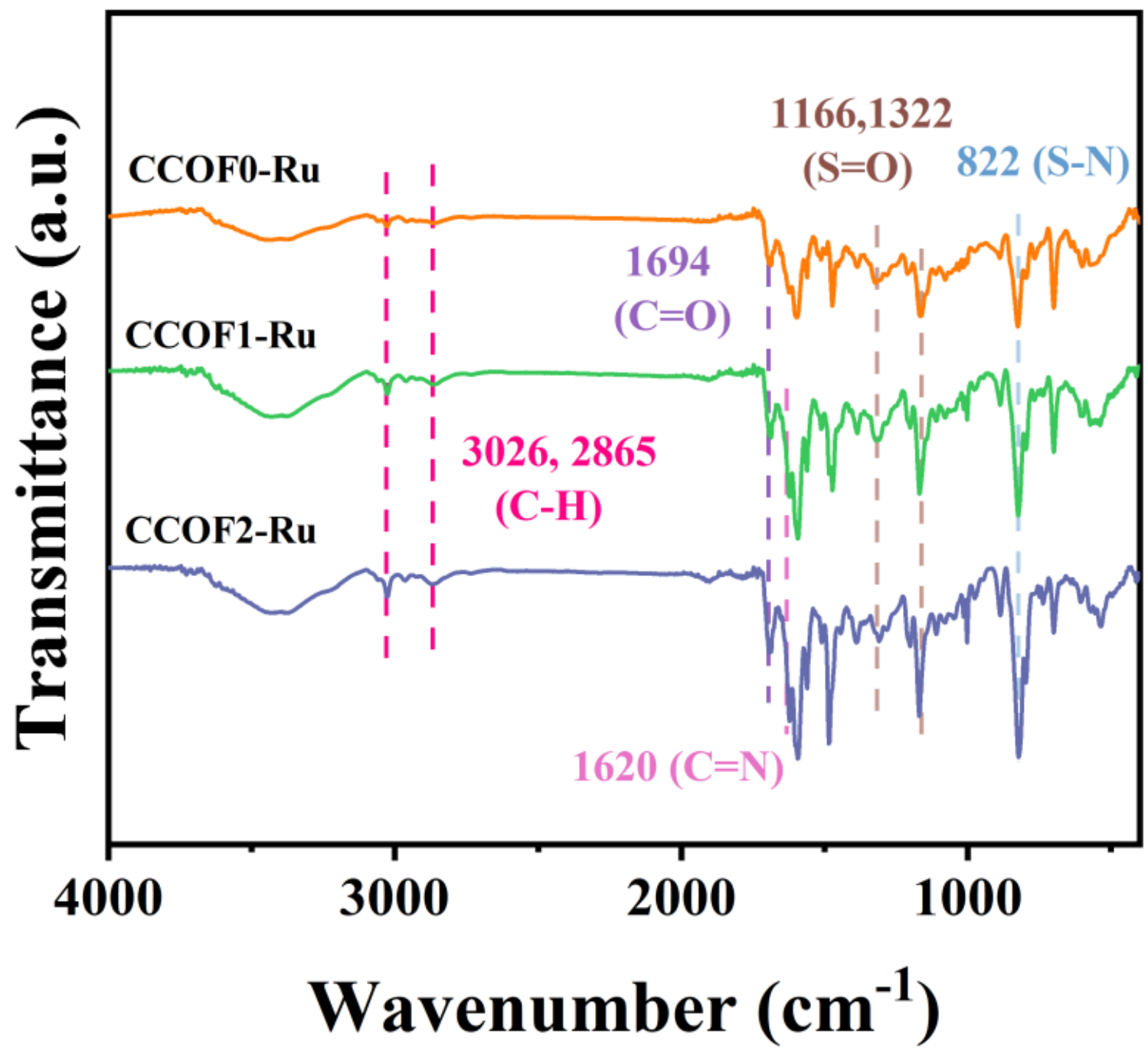

Supplementary Fig. 17 a FTIR spectra of CCOFX-Ru. The peak at 1694 cm^{-1} in the FTIR spectrum is attributed to terminal aldehydes.

Comment 11: Please supplement with PXRD and FTIR data after several catalytic cycles to better demonstrate structural retention under reaction conditions;

Response: We sincerely appreciate the reviewer's valuable suggestion. We completely

agree that demonstrating structural robustness under reaction conditions is crucial for evaluating heterogeneous catalysts. To rigorously address this, we have supplemented PXRD and FTIR spectroscopic measurements on the CCOF2-Ru catalyst after five consecutive asymmetric transfer hydrogenation cycles.

As shown in the newly added Figure 5g, the PXRD pattern of the recovered CCOF2-Ru catalyst is highly consistent with that of the pristine material, confirming that the long-range crystalline order of the framework is well-preserved. Furthermore, the FTIR spectrum of the recycled catalyst (Supplementary Fig. 30a) exhibits all the characteristic vibrational bands of the original framework, indicating that the chemical structure remains intact without any noticeable degradation. These results provide strong evidence for the excellent structural stability of CCOF2-Ru during the catalytic process.

Manuscript Change:

Manuscript (main text, Catalytic Stability Section):

"To further verify the structural robustness of the catalyst under reaction conditions, the recovered CCOF2-Ru catalyst was thoroughly characterized after five consecutive catalytic cycles. The PXRD pattern of the recycled catalyst (Fig. 5g) demonstrated that the long-range crystalline order was fully retained. Additionally, FTIR spectroscopy (Supplementary Fig. 30a) confirmed that the chemical structure of the framework remained intact, with no observable degradation of the functional groups, highlighting the exceptional stability of the multivariate assembled MCCOFs."

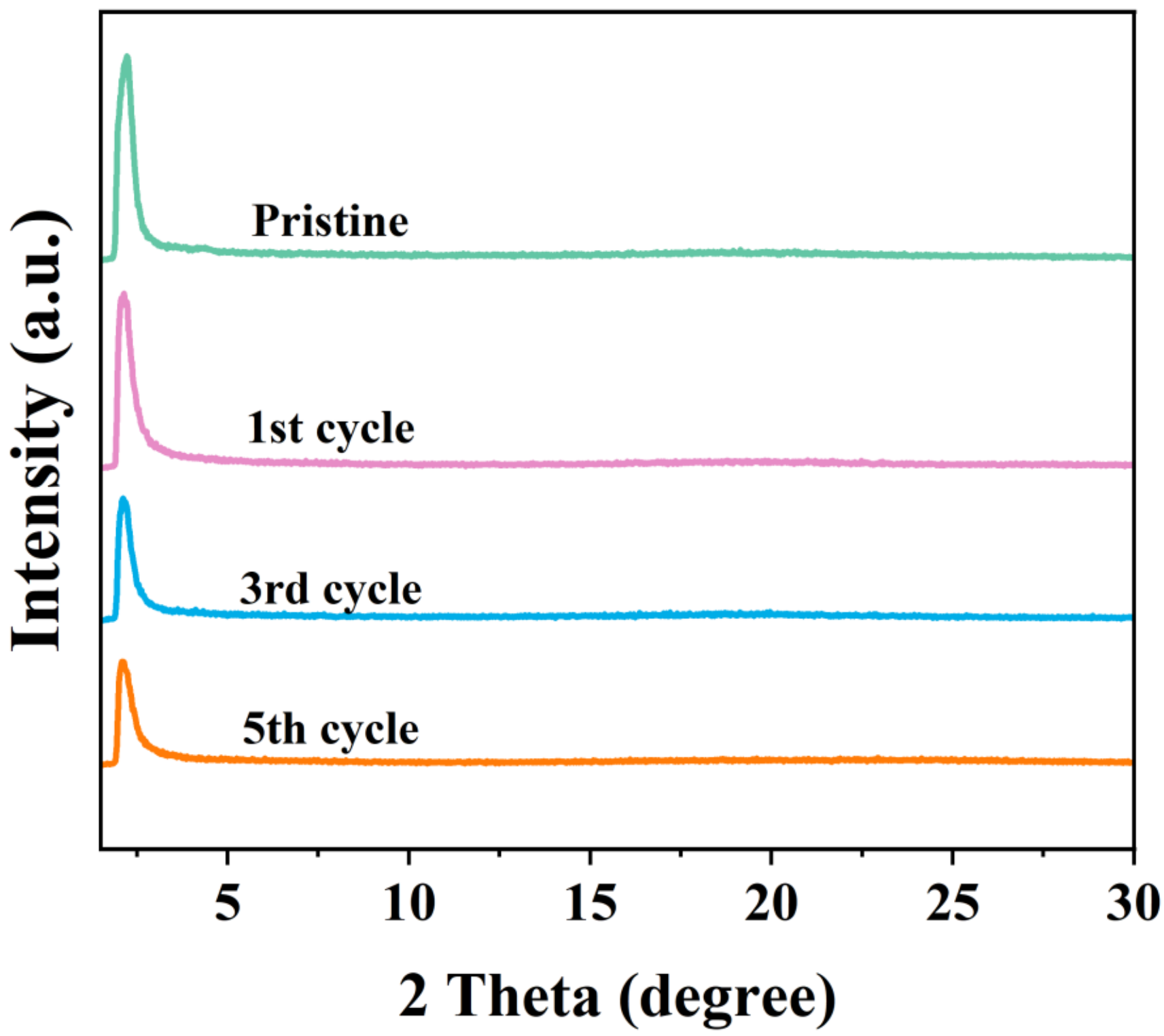

Fig. 5g PXRD patterns of pristine CCOF2-Ru and the recovered catalyst after 5 cycles.

Supporting Information (revised Supplementary Fig. 30a):

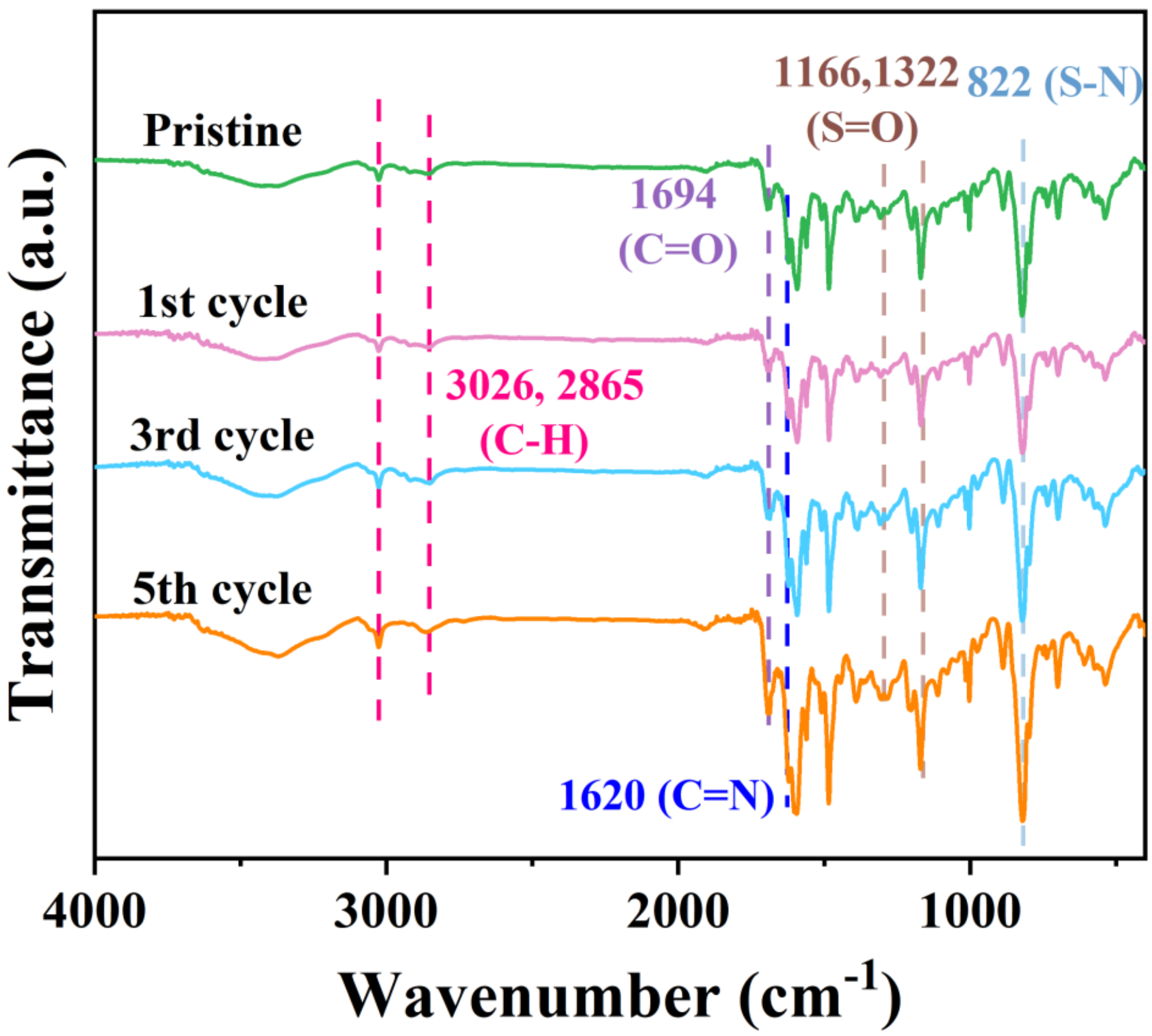

Supplementary Fig. 30a FTIR spectra of pristine CCOF2-Ru and the recovered catalyst after 5 cycles.

Comment 12: In situ or operando spectroscopic studies (e.g., XAS) would help elucidate the coordination environment and evolution of Ru species during catalysis.

Response: We express our sincere gratitude to the reviewer for this highly constructive and professional suggestion. We fully agree that probing the precise coordination environment of the Ru species is crucial for understanding the catalytic mechanism. Prompted by your insightful comment, we have successfully conducted Synchrotron XAS measurements to elucidate the coordination structure of the Ru species. This advanced characterization provides bulk-averaged, atomic-level precision that perfectly complements our surface-sensitive XPS data.

Specifically, the Ru K-edge XANES spectra (new Fig. 4c) reveal that the valence state of Ru in CCOF2-Ru lies between +3 and +4, which is in excellent agreement with our XPS findings. Furthermore, the EXAFS data provide unambiguous evidence for the atomic dispersion and precise coordination geometry of the Ru centers. The Fourier transform EXAFS spectrum (Fig. 4d) and wavelet transform analysis (Fig. 4e–g) show distinct peaks for Ru–N/C and Ru–Cl bonds, with no detectable Ru–Ru or Ru–O–Ru scattering paths, confirming the absence of any nanoparticle aggregation. By fitting the EXAFS data using the crystallographic model of the Ru complex (Supplementary Fig. 19 and Table 10), we determined a first coordination shell comprising Ru–N/C (bond length: 2.159 Å, coordination number (CN) ≈ 7.7) and Ru–Cl (bond length: 2.436 Å, CN ≈ 0.9). This total coordination number matches perfectly with our proposed molecular structure of CCOF2-Ru (Fig. 1b), which theoretically consists of CN = 1 for Ru–Cl, CN = 2 for Ru–N, and CN = 6 for Ru–C.

Regarding the reviewer's excellent suggestion to track the dynamic evolution of Ru species using in situ or operando XAS, we seriously evaluated the feasibility of this experiment at the synchrotron facility. However, conducting operando XAS for our specific system currently faces insurmountable physical limitations. The Ru loading in our CCOF2-Ru catalyst is exceptionally low (only 0.83 wt%), and our catalytic reaction is a heterogeneous solid-liquid multiphase process. In a standard liquid-cell setup, the solvent causes severe scattering and attenuation of the X-ray beam. The combination of ultra-low metal loading and strong solvent interference pushes the signal-to-noise ratio below the detection limit of current operando XAS beamlines, making it nearly impossible to extract reliable dynamic EXAFS fitting data during the reaction.

Nevertheless, the high-quality static XAS data we have now provided rigorously establishes the exact starting active site structure (the precatalyst state), which serves

as the critical foundation for understanding the subsequent catalytic steps. We hope the reviewer agrees that the newly added comprehensive XAS analysis significantly elevates the structural depth and scientific rigor of this work. We also plan to collaborate with specialized beamlines to develop custom high-sensitivity cells for operando studies in our future research. A detailed discussion of these XAS results, along with the corresponding figures and fitting tables, has been incorporated into the revised manuscript (Fig. 4c–g) and the Supporting Information (Supplementary Figs. 18-19, and Supplementary Table 10).

Manuscript Change (Revised text on Page 10, First Paragraph; Revised Figure on Page 11, Fig 4c-g):

Added to the main text: "To further investigate the chemical state and coordination environment of the CCOF2 Ru catalyst, X-ray absorption fine structure (XAFS) analysis was conducted. As shown in Fig. 4c, by comparing the Ru K-edge X-ray absorption near-edge structure (XANES) spectra of CCOF2 Ru with those of $RuCl_3$ and RuO_2, the valence state of Ru atoms in CCOF2 Ru is found to lie between +3 and +4, consistent with the XPS observations. As presented in Fig. 4d, analysis of the extended X-ray absorption fine structure (EXAFS) data reveals two distinct peaks in the Fourier transform (FT) EXAFS spectrum of CCOF2 Ru, attributed to Ru–N/C (1.53 Å) and Ru–Cl (2.02 Å) bonds. No peaks corresponding to Ru–Ru or Ru–O–Ru bonds are observed, indicating that the ruthenium species are atomically dispersed. Furthermore, wavelet transform analysis of the EXAFS spectra further confirms the presence of Ru–N/C and Ru–Cl coordination structures (Fig. 4e–g). Using a coordination shell composed of chloride, p-cymene, and a DPEN ligand around Ru as the initial structural model for EXAFS curve fitting of the CCOF2 Ru samples, with atomic coordinates taken from the crystal structure previously reported by Dub et al.[72] (COD ID: 4128180), the FT *R*-space EXAFS spectra (Supplementary Fig. 19) together with the fitting results (Supplementary Table 10) indicate the presence of a first coordination shell comprising Ru–N/C (bond length: 2.159 Å, coordination number (CN) ≈ 7.7) and Ru–Cl (bond length: 2.436 Å, CN ≈ 0.9) in CCOF2-Ru. The total coordination number is consistent with the expected structure of CCOF2 Ru (Fig. 1), namely CN = 1 for Ru–Cl, CN = 2 for Ru–N, and CN = 6 for Ru–C."

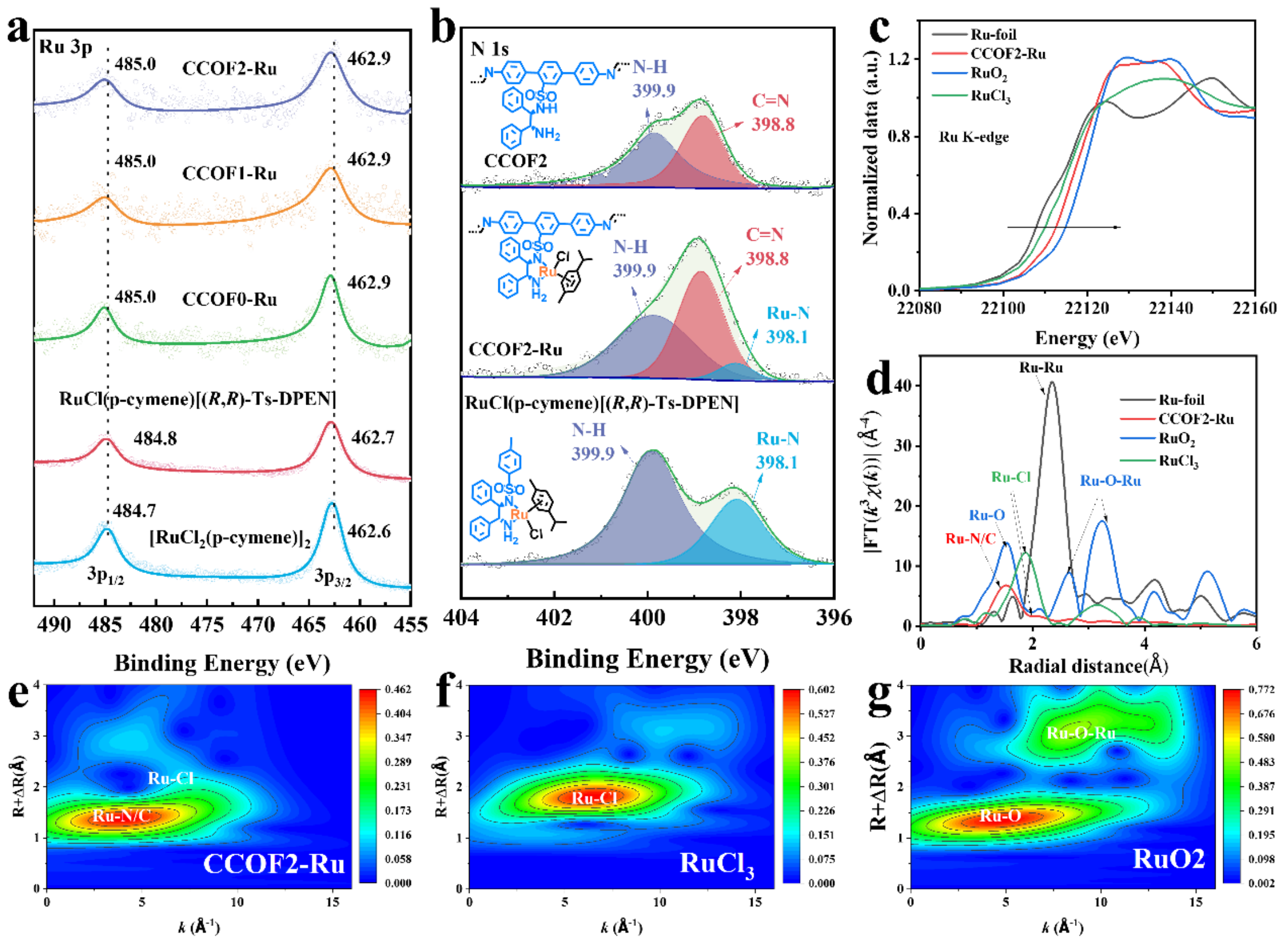

Fig. 4 Structural investigation of CCOF2-Ru. XPS spectra of **a** Ru 3p and **b** N 1s. **c** XANES spectra, **d** EXAFS of CCOF2-Ru. Wavelet transform of **e** CCOF2-Ru, **f** $RuCl_3$ and **g** RuO_2.

Supporting Information (revised Figures on page 46, Supplementary Fig. 18; revised Figures on page 47, Supplementary Fig. 19; revised tables on page 81, Supplementary Table10):

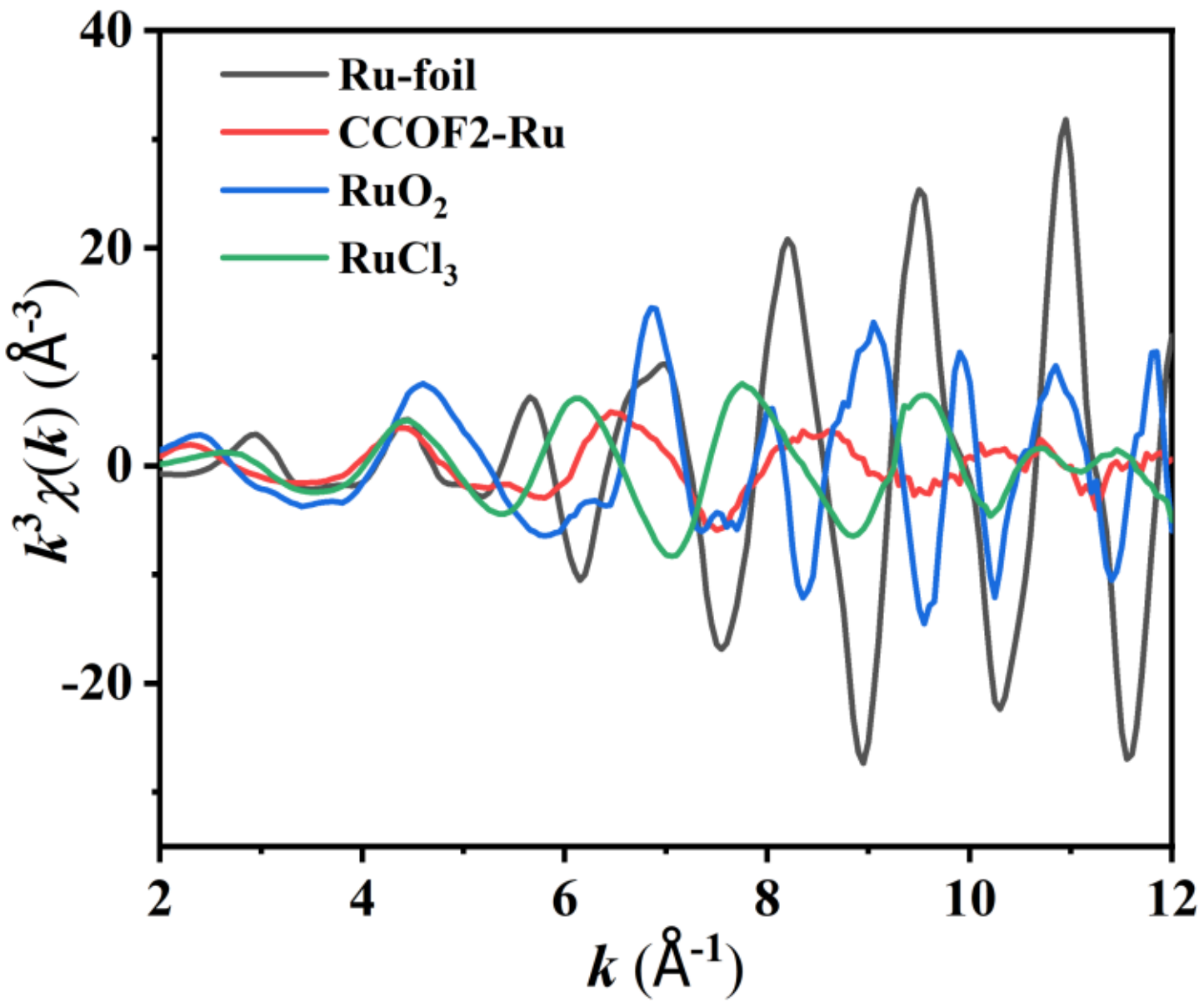

Supplementary Fig. 18 CCOF2-Ru and standard referenced k3χ(k) oscillation curve.

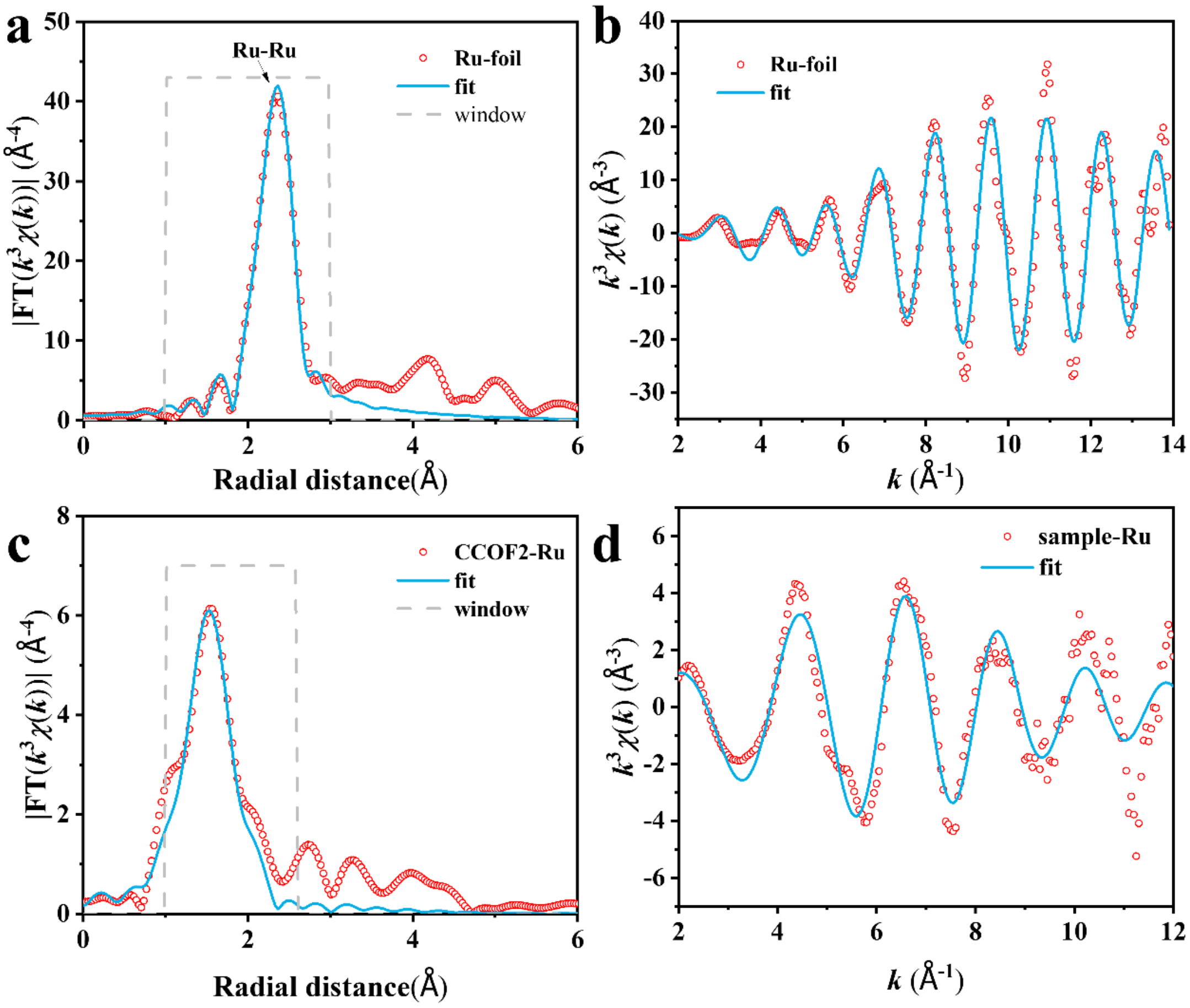

Supplementary Fig. 19 The fitted Fourier transform R-spaced extended X-ray absorption fine spectra (FT-EXAFS) of a Ru foil and c CCOF2-Ru. The fitted k3χ(k) oscillation curve of b Ru foil and d CCOF2-Ru.

Supplementary Table10. EXAFS data fitting results of CCOF2-Ru.

Sample	Path	CN^a	R(Å)^b	σ^2 (Å^2)^c	ΔE_0(eV)^d	R factor
Ru foil	Ru-Ru	12.0*	2.676±0.003	0.0042	-5.3±0.9	0.0069
CCOF2-Ru	Ru-N/C	7.7±0.8	2.159±0.033	0.0083	9.1±3.1	0.0199
	Ru-Cl	0.9±0.3	2.436±0.021	0.0026		

^{a}*CN* is coordination number; ^{b}*R* is the distance between absorber and backscatter atoms; $^c\sigma^2$ is the Debye Waller factor value; $^d\Delta E_0$ is inner potential correction to account for the difference in the inner potential between the sample and the reference compound; *R* factor indicates the goodness of the fit. $S0^2$ was fixed to 0.879, according to the experimental EXAFS fit of Ru foil by fixing *CN* as the known crystallographic value. *This value was fixed during EXAFS fitting, based on the known structure of Ru.

Fitting conditions: *k* range: 2.0 - 10.5; *R* range: 1.0-2.6; fitting space: R space; *k*-weight = 3.

Reference:

72. Dub PA, Matsunami A, Kuwata S, Kayaki Y. Cleavage of N–H Bond of Ammonia via Metal–Ligand Cooperation Enables Rational Design of a Conceptually New Noyori–Ikariya Catalyst. J. Am. Chem. Soc. 141, 2661-2677 (2019).

Response to the comments

Reviewer 1:

The authors have made a substantial effort to address the concerns raised during the previous round of review. In particular, the revised Supplementary Information has been significantly improved, and the characterization of both the synthesized precursors and the post-catalysis compounds is now considerably more complete. The authors have clarified many of the ambiguities present in the original submission, and the overall quality of the manuscript has clearly benefited from these revisions. However, despite these significant improvements, my main concern regarding the structural characterization of the reported CCOFs remains unresolved.

Response: We sincerely thank the reviewer for the positive and encouraging assessment of the revisions made in the previous round. We also appreciate the reviewer's continued concern regarding the structural characterization of the CCOFs. We agree that this point is central to the manuscript and have therefore further strengthened the structural analysis and revised the manuscript wording to avoid overinterpretation.

Specifically, we have added Rietveld refinements based on updated PXRD profiles, compared AA-eclipsed and AB-staggered structural models, calculated pore limiting diameters (PLD) and largest cavity diameters (LCD) for both stacking models, and revised the discussion of the porosity discrepancy. We now describe the AA-eclipsed framework as the structural model best supported by the combined evidence, rather than as a definitive crystallographic solution. These changes are intended to make the structural assignment more rigorous, cautious, and transparent.

Comment 1: The crystallinity of the described CCOFs, together with the limited quality and resolution of the PXRD data, is still insufficient to unambiguously support the structural assignments proposed by the authors. In particular, the structural evidence provided does not justify definitive claims regarding the framework arrangement;

Response: We thank the reviewer for this important and constructive criticism. We agree that, for polycrystalline 2D COFs with weak higher-order PXRD reflections, the framework arrangement should not be described with the same level of certainty as a single-crystal structure. We have therefore revised the manuscript to present the AA-eclipsed arrangement as the structural model most consistent with the combined experimental and computational evidence, rather than as an unambiguous assignment based solely on PXRD.

To address this point, we re-examined the PXRD data and performed Rietveld refinements using the updated experimental profiles. The CCOF series shows intense low-angle reflections assigned to the (100) planes. In addition, weak but discernible higher-order reflections, such as (110) or (200), are observed in the updated patterns for selected samples. Although these higher-order reflections are weak, their positions are consistent with the simulated AA-eclipsed models. The refined unit-cell parameters and residual factors are now provided in Supplementary Table 3. These refinements support the proposed AA-eclipsed models, but we have revised the manuscript to state explicitly that the refinements are supportive and should be interpreted together with the other characterization results.

We also compared AA-eclipsed and AB-staggered structural models by calculating the pore limiting diameter (PLD) and largest cavity diameter (LCD) using ZEO++ for each CCOF. For CCOF0-Boc and CCOF1-Boc, the AB-staggered models give substantially smaller pore apertures than the experimentally observed pore-size populations. For CCOF2-Boc and CCOF2, which exhibit higher crystallinity and BET surface areas, the experimentally observed pore sizes are closer to the values predicted by the AA-eclipsed models. Thus, the porosity data do not prove a unique structure on their own, but they are more consistent with the AA-eclipsed models than with the AB-staggered alternatives.

TEM and HRTEM images provide additional local structural information. The observed honeycomb-like channels in CCOF1 and CCOF2 are consistent with an eclipsed hexagonal arrangement and are less compatible with a fully staggered AB model, which would be expected to reduce or distort the open hexagonal channels in projection.

Overall, the revised structural assignment is based on the convergence of several observations: imine formation and linker incorporation from FTIR and solid-state ^{13}C CP/MAS NMR, compositional consistency from elemental analysis, dominant low-angle PXRD reflections and Rietveld refinements consistent with AA stacking, pore-size comparisons favoring AA over AB models, and local HRTEM visualization of honeycomb-like channels. We have modified the manuscript wording accordingly to ensure that the structural conclusion is scientifically cautious and not overstated.

Manuscript Change:

Manuscript (Revised text on page 6, first paragraph; Revised figures on page 6, Fig. 2c,d and page 8, Fig. 3a-h):

The following text has been revised in the manuscript:

“The Boc-protected variants (CCOF0-Boc, CCOF1-Boc, and CCOF2-Boc)

displayed intense low-angle diffraction peaks at 2.26°, 2.20°, and 2.12°, respectively, corresponding to the (100) reflection, together with weak higher-order reflections for selected samples (Supplementary Fig. 4). The long-range order of these frameworks is evidenced by the narrow full width at half maximum (FWHM) values of the (100) plane (0.44°–0.51°). Rietveld refinements using AA-eclipsed structural models provide satisfactory agreement with the experimental patterns, as reflected by the residual factors summarized in Supplementary Table 3. For the binary CCOF0-Boc, comparison of experimental profiles with simulated patterns (Fig. 2c) supports its assignment to a hexagonal 2D network with an eclipsed AA stacking arrangement (space group P3, Fig. 2d). A weak diffraction peak at 3.82° in the inset of Fig. 2c can be assigned to the (110) reflection. The refinement yielded unit-cell parameters of $a = b = 45.4766$ Å, $c = 4.8584$ Å, $\alpha = \beta = 90°$, and $\gamma = 120°$, in good agreement with the theoretical model, with low residual factors ($R_p = 3.19\%$ and $R_{wp} = 4.66\%$)."

"Similarly, the ternary analogues (CCOF1-Boc through CCOF2) were found to adopt eclipsed AA stacking within hexagonal 2D networks (space group P21), as supported by Rietveld refinements (Fig. 3a–h; Supplementary Fig. 6; Supplementary Table 3-8). The weak diffraction peaks at 4.20°, 4.18°, 4.18°, and 4.16° in the insets of Fig. 3a–d can be assigned to the (200) reflections of CCOF1-Boc, CCOF2-Boc, CCOF1, and CCOF2, respectively."

The HRTEM-related statement has been revised as follows:

"The honeycomb-like channels observed in the HRTEM images of CCOF1 and CCOF2 (Fig. 3k,l) are consistent with the proposed AA-eclipsed hexagonal arrangement and are less compatible with a fully AB-staggered model, which would be expected to substantially reduce or distort the open hexagonal channels in projection."

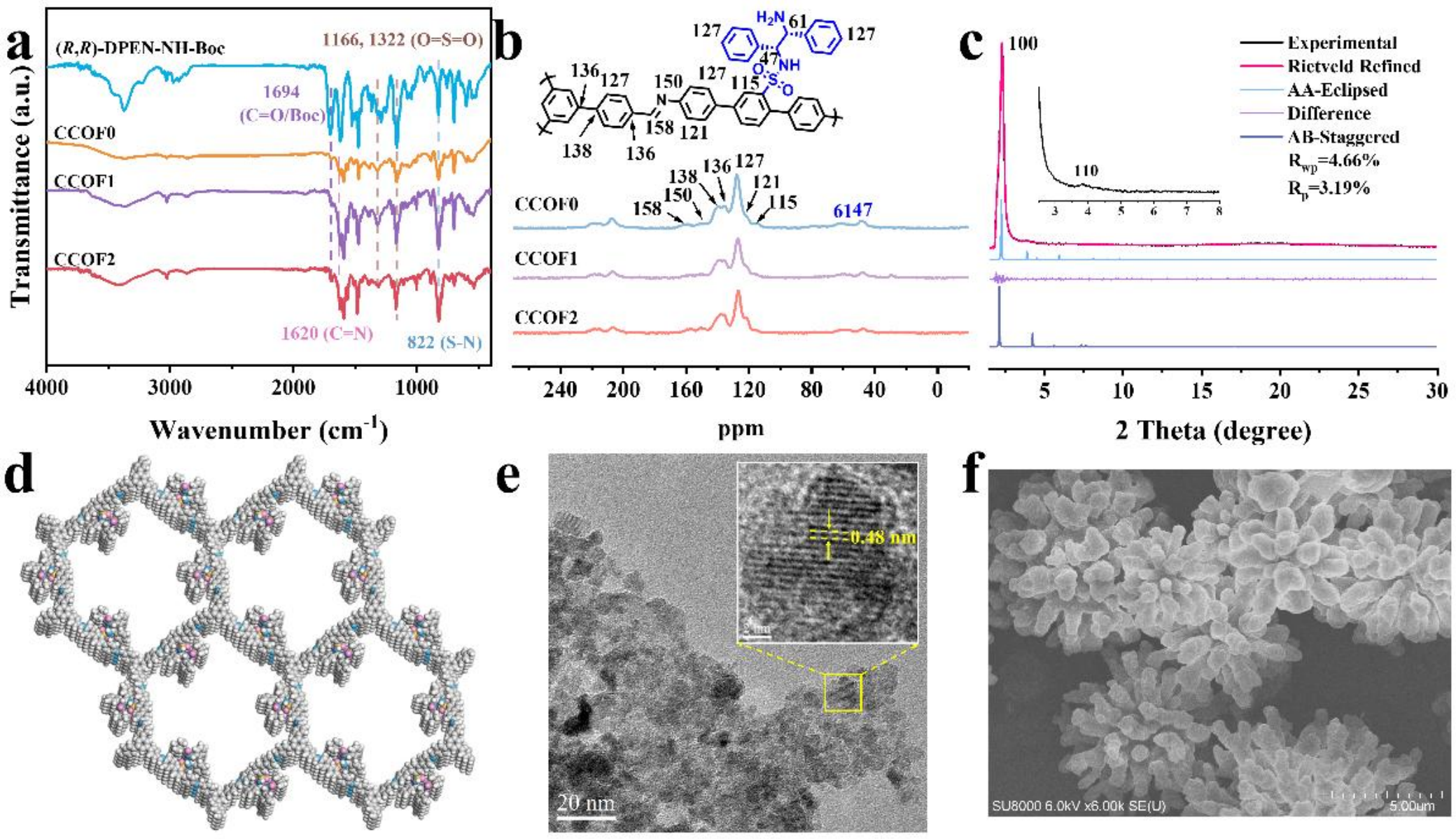

Fig. 2 Structural characterization of CCOFs. a FTIR spectra and **b** solid-state ^{13}C CP/MAS NMR spectra of CCOFX. **c** Experimental PXRD patterns of CCOF0-Boc, **d** simulated AA stacking crystal structure of top view (gray, C; blue, N; red, O; yellow, S; white, H), **e** TEM image and SEM image **f** of CCOF0-Boc.

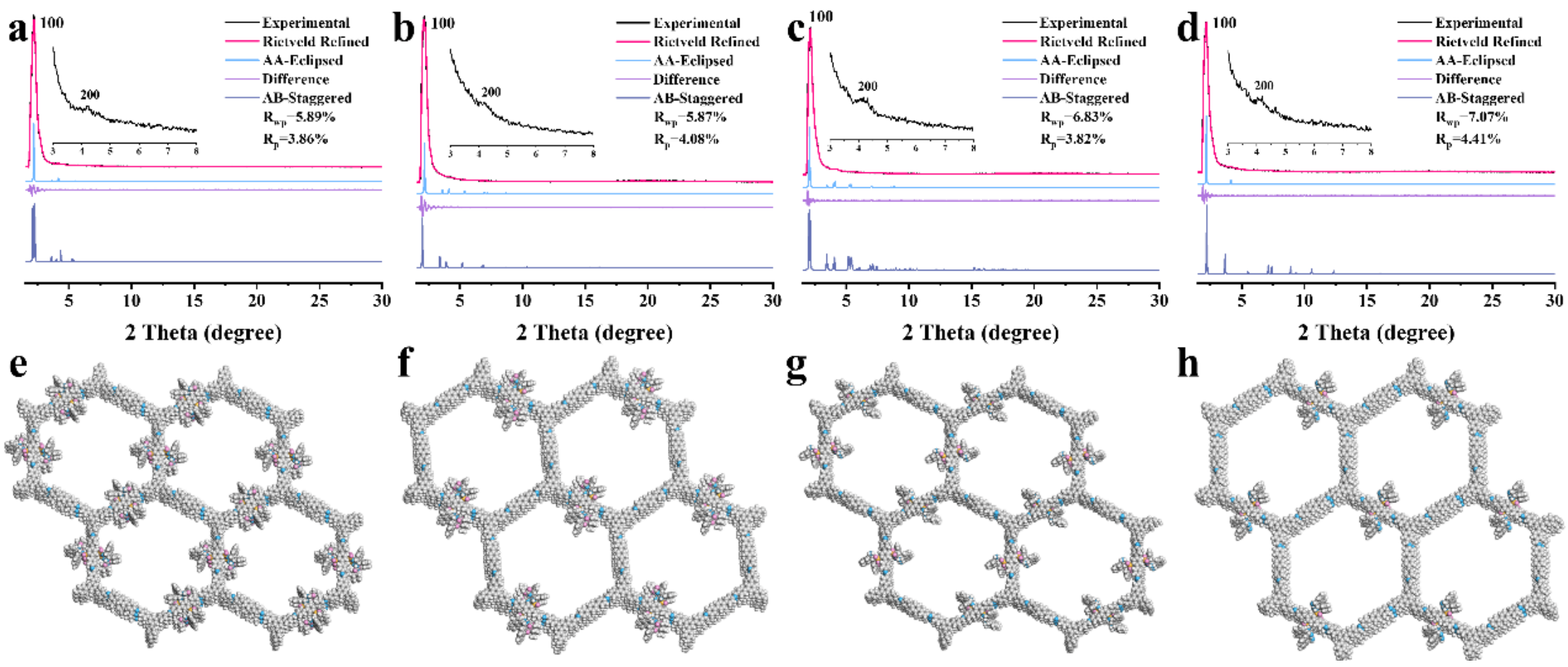

Fig. 3 PXRD analysis, structural models and HR-TEM images of ternary CCOFs. Experimental PXRD patterns of **a** CCOF1-Boc, **b** CCOF2-Boc, **c** CCOF1, and **d** CCOF2. Simulated AA stacking crystal structure of top views of **e** CCOF1-Boc, **f** CCOF2-Boc, **g** CCOF1, and **h** CCOF2 (gray, C; blue, N; red, O; yellow, S; white, H).

Supporting Information (revised Supplementary Fig. 6, page 39):

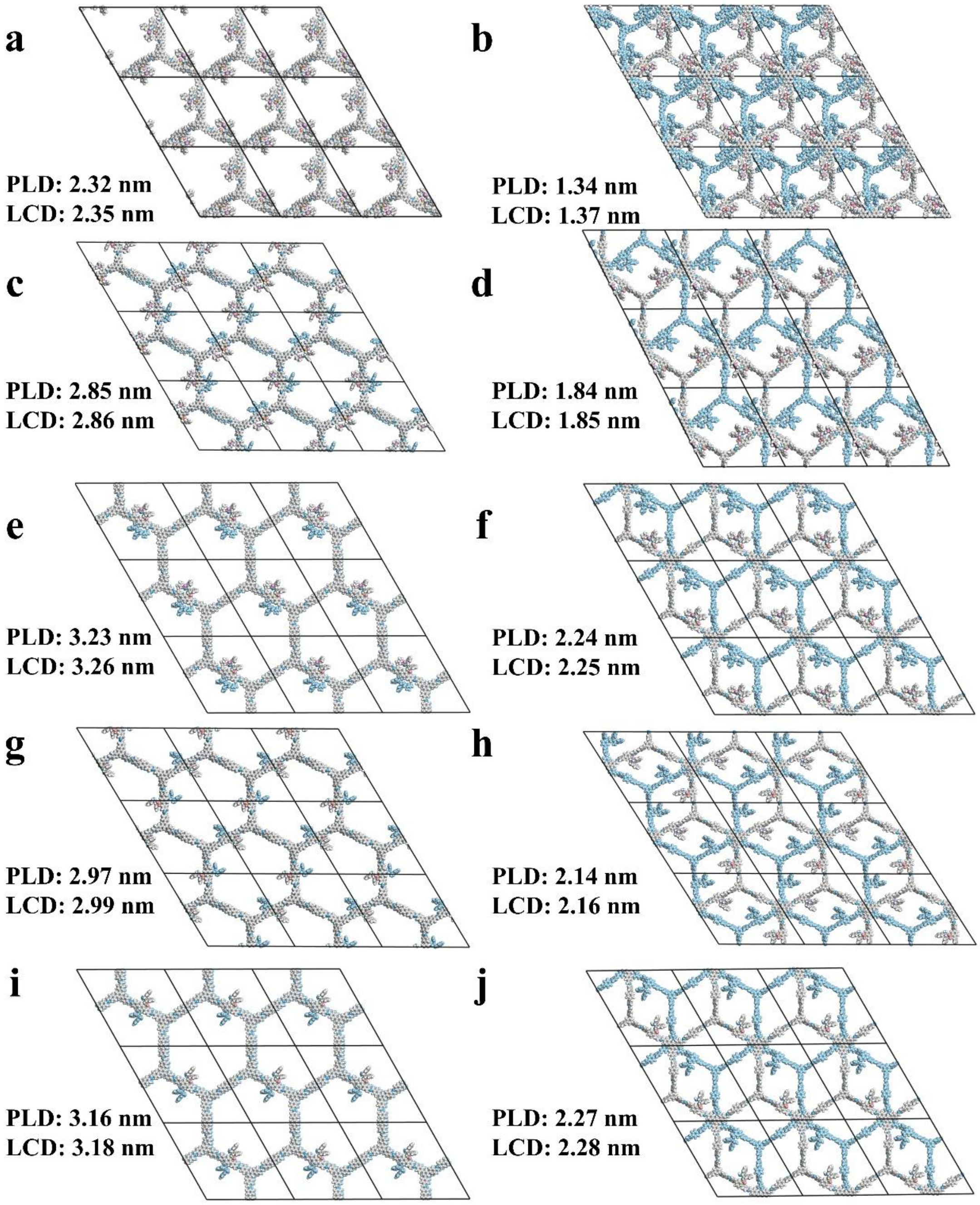

Supplementary Fig. 6 Optimized AA-eclipsed structural models of (a) CCOF0-Boc, (c) CCOF1-Boc, (e) CCOF2-Boc, (g) CCOF1, and (i) CCOF2, and AB-staggered structural models of (b) CCOF0-Boc, (d) CCOF1-Boc, (f) CCOF2-Boc, (h) CCOF1, and (j) CCOF2. The PLD and LCD were calculated using ZEO++. For all CCOFs, the PLD and LCD values of the AA-eclipsed models match the BET-derived pore sizes (Supplementary Fig. 7) better than those of the AB-staggered models, suggesting that these CCOFs are more likely to adopt AA-eclipsed stacking.

Comment 2: Furthermore, the authors justify the low porosity of the materials by

stating that "in 2D COFs bearing such bulky side chains, adjacent layers often undergo sliding or staggered stacking to minimize severe interlayer steric repulsion." This explanation appears to contradict their proposed AA-eclipsed structural model. The optimized theoretical values reported in Supplementary Table 2 (which I assume correspond to the AA-eclipsed structure, including the complexed species) are significantly higher than the experimentally observed values. If the authors attribute the reduced porosity to layer sliding or staggered stacking, then they should provide and compare optimized AB-staggered structural models, as these would be more consistent with their own interpretation of the observed porosity;

Response: We thank the reviewer for identifying this inconsistency. We agree that attributing the low BET surface areas primarily to layer sliding or AB-staggered stacking could conflict with the proposed AA-eclipsed model. We have therefore removed that explanation and replaced it with a more cautious interpretation.

In the revised manuscript, the low experimental BET surface areas are attributed mainly to three factors that do not require a global AA-to-AB stacking transformation: (i) occupation of internal pore volume by bulky Boc-protected chiral side chains; (ii) reduced long-range order and local disordered domains, particularly in CCOF0-Boc and CCOF1-Boc; and (iii) partial pore blockage or incomplete channel accessibility in real powder samples.

The discrepancy between theoretical and experimental porosity is expected to be especially large for sterically crowded 2D COFs because the theoretical values are calculated from idealized, infinite, defect-free, fully accessible structural models. In contrast, experimental nitrogen sorption reflects the accessible porosity of real materials, which can be strongly affected by finite crystallite size, stacking faults, local disorder, trapped side chains, pore blockage, and imperfect activation. Therefore, the lower experimental BET areas do not necessarily require a globally AB-staggered structure.

Following the reviewer's suggestion, we constructed optimized AB-staggered models and compared their PLD/LCD values with those of the AA-eclipsed models using ZEO++ (Supplementary Fig. 6). The AB-staggered models of CCOF0-Boc and CCOF1-Boc give PLD/LCD values below 2 nm, whereas the experimental pore-size distributions show appreciable pore populations extending into the 2–4 nm range. For CCOF2-Boc and CCOF2, the observed pore sizes are also closer to the AA-eclipsed models. Thus, while local stacking disorder or layer offsets cannot be completely excluded, the available data are more consistent with predominantly AA-eclipsed frameworks containing defects or partially inaccessible channels than with fully AB-staggered structures.

The trend across the series supports this interpretation: CCOF2-Boc and CCOF2 show higher crystallinity and substantially higher BET surface areas than CCOF0-Boc, CCOF1-Boc, and CCOF1, indicating that DATP incorporation improves framework ordering and pore accessibility. We have revised the relevant manuscript text accordingly.

Manuscript Change:

The low-porosity discussion on page 7 has been revised. The earlier statement invoking layer sliding or staggered stacking has been removed. Supplementary Fig. 6 now includes optimized AA-eclipsed and AB-staggered models with PLD/LCD values, and Supplementary Table 2 reports theoretical and experimental BET surface areas and pore volumes.

The revised manuscript text reads:

"As shown in Supplementary Table 2, the experimentally measured BET surface areas and pore volumes are lower than those calculated using Zeo++ from the idealized structural models. This discrepancy is most pronounced for CCOF0-Boc and CCOF1-Boc and is mainly attributed to the difference between ideal, defect-free theoretical models and real powder samples containing bulky Boc-protected chiral side chains, reduced long-range order, local defects, and partially inaccessible channels. The bulky chiral units occupy part of the internal pore volume and may weaken interlayer π–π interactions, thereby reducing structural order and channel accessibility.[61,62] Importantly, the reduced accessible porosity does not necessarily imply a global AB-staggered stacking arrangement. Comparison of optimized AA-eclipsed and AB-staggered models shows that the experimentally observed pore-size distributions are more consistent with the AA-eclipsed models, particularly for the more crystalline CCOF2-Boc and CCOF2. The significantly higher BET surface area of CCOF2-Boc is therefore consistent with its enhanced crystallinity and supports the role of the multivariate strategy in improving framework order and pore accessibility."

Reference:

61. Pelkowski CE, et al. Tuning Crystallinity and Stacking of Two-Dimensional Covalent Organic Frameworks through Side-Chain Interactions. *J. Am. Chem. Soc*. **145**, 21798-21806 (2023).

62. Thompson CM, et al. Computational and Experimental Studies on the Effects of Monomer Planarity on Covalent Organic Framework Formation. *J. Am. Chem. Soc*. **139**, 10506-10513 (2017).

Comment 3: At present, while the catalytic results convincingly demonstrate that the CCOF materials are catalytically active, and notably that CCOF2-Ru affords very high enantioselectivities, the actual structures of the catalytic materials cannot be conclusively established. This is due to several unresolved issues:

1- The low crystallinity of the materials;

2- The insufficient resolution of the PXRD patterns;

3- The absence of appropriate structural refinement (e.g., Rietveld refinement);

4- The significant discrepancy between the experimental porosity values and the optimized theoretical values reported by the authors;

Response: We thank the reviewer for summarizing the remaining structural issues so clearly. We agree that the catalytic activity and enantioselectivity of the materials are strongly supported by the catalytic data, whereas the crystallographic description of the CCOF powders should be presented with appropriate caution. In the further revision, we therefore revised the structural language and added new analyses to address each point.

1. Low crystallinity.

We acknowledge that CCOF0-Boc, CCOF1-Boc, and especially the deprotected CCOF0 display limited crystallinity compared with highly ordered benchmark COFs. We have revised the manuscript to avoid overgeneralized statements such as 'high crystallinity of the CCOF series.' Instead, we now describe a clear trend in structural ordering across the series: incorporation of DATP improves the crystallinity and porosity, with CCOF2-Boc and CCOF2 showing the most ordered structures among the materials studied. This trend is supported by sharper low-angle PXRD reflections, lower FWHM values, improved BET surface areas, and better retention of structure after deprotection.

2. PXRD resolution.

We agree that the higher-order PXRD reflections are weak, which limits the degree of crystallographic certainty. We have therefore revised the manuscript to state that the PXRD data support, but do not alone unambiguously prove, the proposed framework arrangement. The weak higher-order reflections can be rationalized by the bulky, non-planar chiral sulfonyldiamine substituents and terphenyl-based linkers, which may introduce local torsional disorder and stacking faults. Such effects broaden and attenuate higher-index reflections while preserving the dominant low-angle reflection characteristic of the in-plane periodicity. This explanation is consistent with literature precedents for 2D COFs containing non-planar or sterically demanding linkers (J. Am. Chem. Soc. 2016, 138, 11489–11492; Angew. Chem., Int. Ed. 2024, 63, e202405769;

J. Am. Chem. Soc. 2025, 147, 12460–12468).

3. Structural refinement.

Following the reviewer's suggestion, we performed Rietveld refinements using the updated PXRD profiles. The AA-eclipsed models provide satisfactory agreement with the experimental patterns, and the refined unit-cell parameters and residual factors are now summarized in Supplementary Table 3. We also show the experimental, refined, difference, AA-eclipsed simulated, and AB-staggered simulated profiles in Fig. 2c and Fig. 3a–d. These refinements provide important additional support for the proposed structural models. However, in response to the reviewer's concern, we have revised the manuscript to make clear that these refinements are not presented as absolute proof of the framework arrangement, but as one component of an integrated structural assignment.

4. Porosity discrepancy.

We agree that the discrepancy between theoretical and experimental porosity requires careful explanation. We have revised the manuscript accordingly. The theoretical BET areas and pore volumes were calculated from idealized, periodic, fully accessible structural models, whereas the experimental BET surface areas reflect the accessible porosity of real powder materials. In sterically crowded chiral COFs, defects, finite crystallite size, local stacking disorder, residual pore blockage, partial amorphization, and bulky side-chain occupation can all substantially reduce the experimentally accessible surface area. Therefore, the large discrepancy does not necessarily indicate that the assigned framework topology is incorrect. To address the reviewer's specific suggestion, we constructed AB-staggered models and compared their calculated PLD/LCD values with those of the AA-eclipsed models. This comparison shows that the observed pore-size distributions are more consistent with the AA-eclipsed models, especially for CCOF2-Boc and CCOF2.

Finally, we note that the central catalytic conclusion does not rely on assuming a perfect ideal structure throughout every crystallite. The key experimentally supported point is that the multivariate CCOF2-derived catalyst has higher structural order, greater accessible porosity, more stable Ru anchoring, lower Ru leaching, and superior catalytic performance than the binary and less ordered analogues. This structure–function relationship is supported by PXRD, nitrogen sorption, HRTEM, XPS, EXAFS, ICP-OES, leaching tests, recycling experiments, and post-catalysis PXRD/FTIR analyses. We have revised the manuscript to present this conclusion in a more balanced and evidence-based manner.

Manuscript Change:

Pages 6–8 and 10–18 have been revised to distinguish direct experimental observations from structural interpretation. Fig. 2c,d Fig. 3a–h, Supplementary Fig. 6, Supplementary Table 2, and Supplementary Table 3 have been updated.

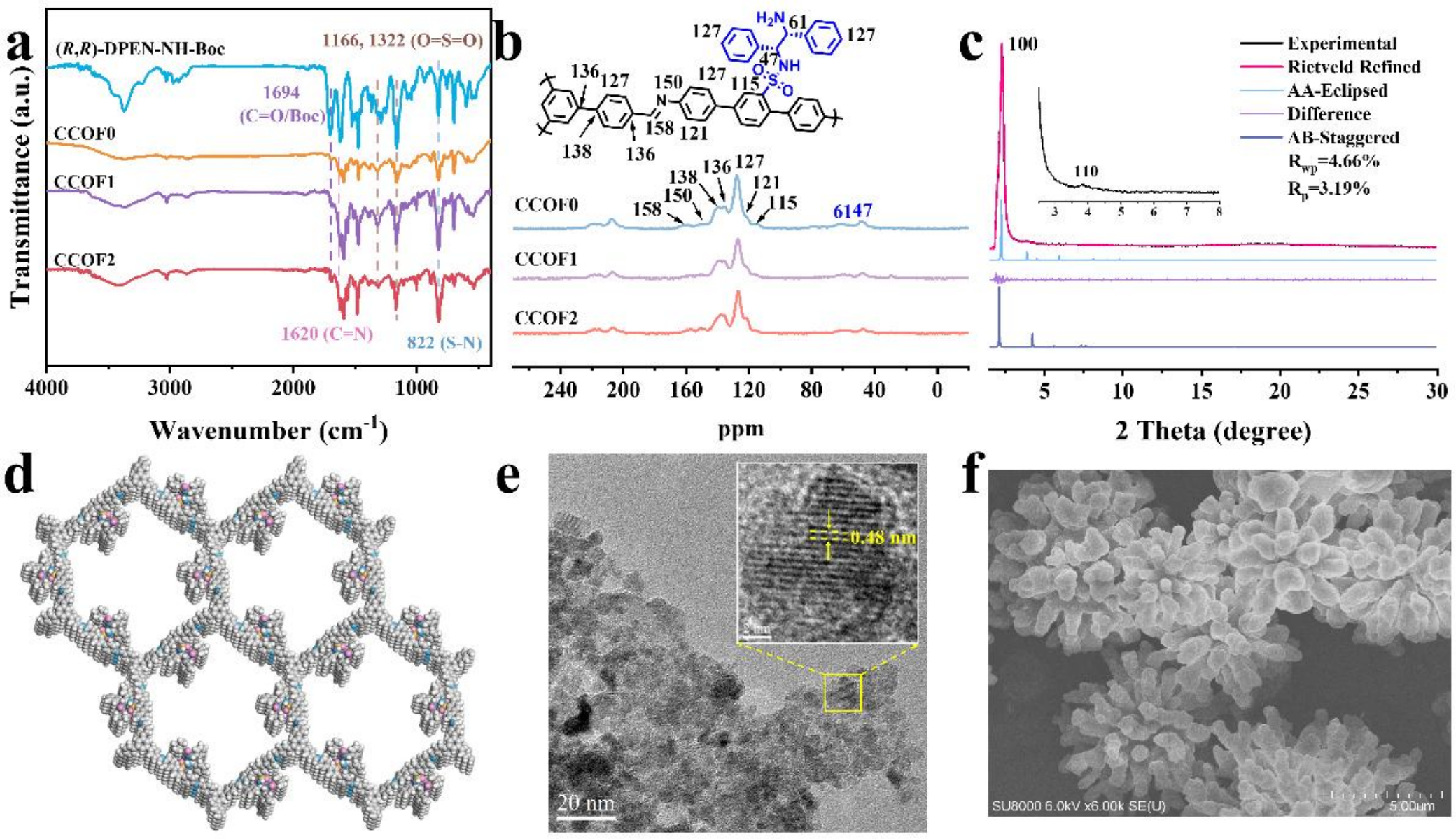

Fig. 2 Structural characterization of CCOFs. a FTIR spectra and **b** solid-state ^{13}C CP/MAS NMR spectra of CCOFX. **c** Experimental PXRD patterns of CCOF0-Boc, **d** simulated AA stacking crystal structure of top view (gray, C; blue, N; red, O; yellow, S; white, H), **e** TEM image and SEM image **f** of CCOF0-Boc.

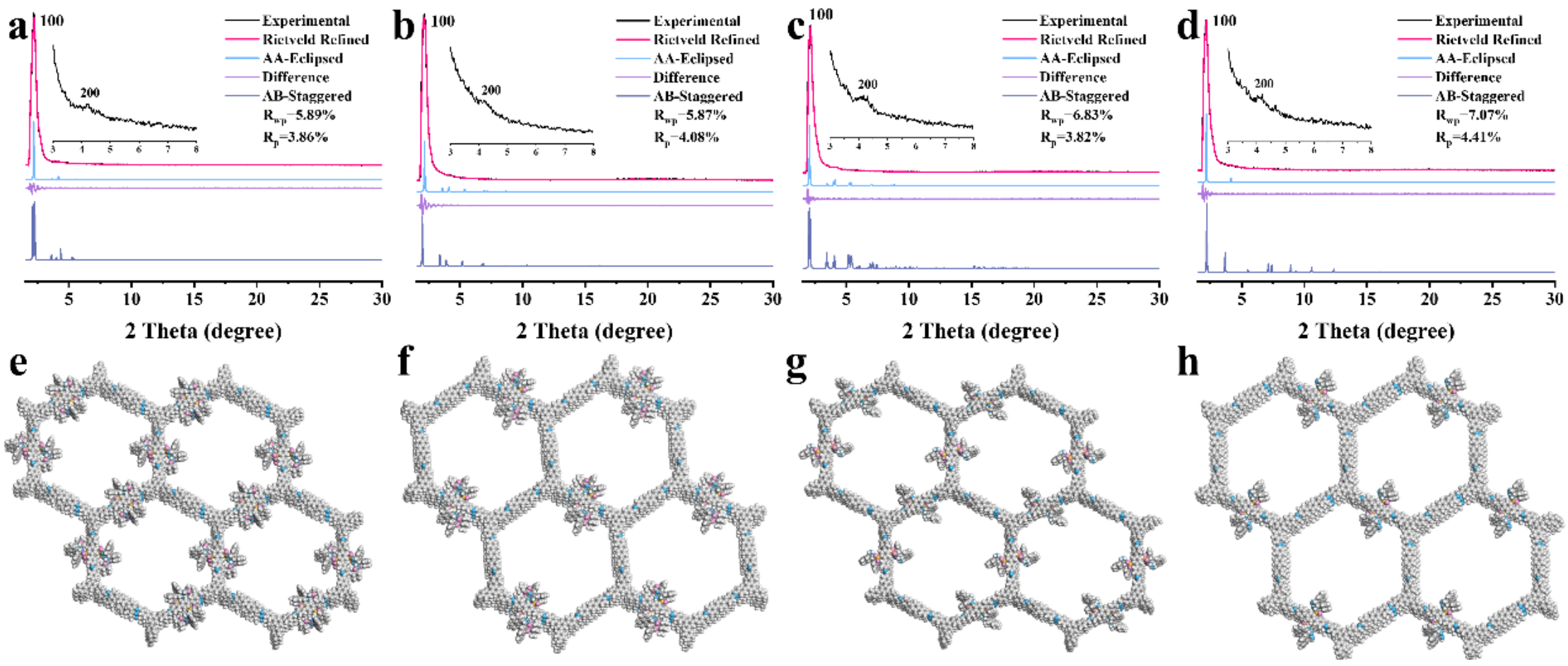

Fig. 3 PXRD analysis, structural models and HR-TEM images of ternary CCOFs. Experimental PXRD patterns of **a** CCOF1-Boc, **b** CCOF2-Boc, **c** CCOF1, and **d** CCOF2. Simulated AA stacking crystal structure of top views of **e** CCOF1-Boc, **f** CCOF2-Boc, **g** CCOF1, and **h** CCOF2 (gray, C; blue, N; red, O; yellow, S; white, H).

Supplementary Table 2. Theoretical BET surface area and pore volumes of CCOFs.

COFs	BET surface areas (m^2/g)	Theoretical BET surface area (m^2/g)	Pore volumes (cm^3/g)	Theoretical pore volumes (cm^3/g)
CCOF0-Boc	21	2009	0.02	1.30
CCOF1-Boc	70	2446	0.03	1.69
CCOF2-Boc	518	2304	0.33	2.03
CCOF1	40	2663	0.02	2.05
CCOF2	473	2451	0.36	2.19

Supplementary Table 3. Rietveld refined crystal unit cell parameters of CCOFs.

COFs	stacking mode	unit-cell parameters	Rwp, Rp
CCOF0-Boc	AA	P3 a = b = 45.4766 Å, c = 4.8584 Å α =β = 90.00° γ = 120.00°	Rwp = 4.66% Rp = 3.19%
CCOF1-Boc	AA	$P2_1$ a = 52.8905 Å, b = 51.8086 Å, c = 7.6248 Å α = β = 90°, γ = 120.7785°	Rwp = 5.89% Rp = 3.86%
CCOF2-Boc	AA	$P2_1$ a = 51.8041 Å, b = 52.2932 Å, c = 7.1590Å α =β = 90°, γ = 119.8850°	Rwp = 5.87% Rp = 4.08%
CCOF1	AA	$P2_1$ a = 52.9766 Å, b = 51.9749 Å, c = 7.9221 Å α =β = 90°, γ = 121.2603°	Rwp = 6.93% Rp = 3.82%
CCOF2	AA	$P2_1$ a = 51.7202 Å, b = 51.9502 Å, c = 7.1166 Å α =β = 90°, γ = 119.3502°	Rwp = 7.07% Rp = 4.41%

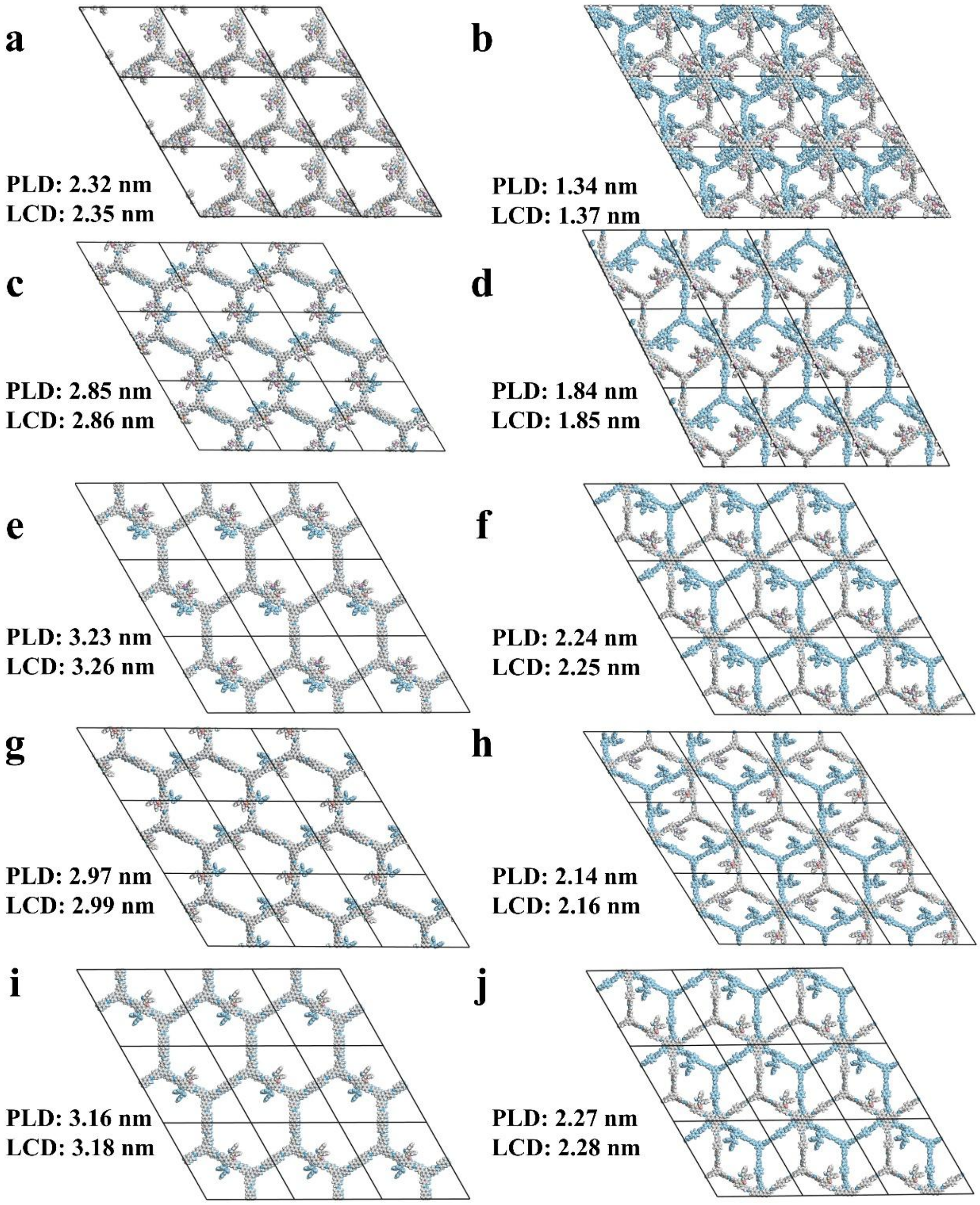

Supplementary Fig. 6 Optimized AA-eclipsed structural models of (a) CCOF0-Boc, (c) CCOF1-Boc, (e) CCOF2-Boc, (g) CCOF1, and (i) CCOF2, and AB-staggered structural models of (b) CCOF0-Boc, (d) CCOF1-Boc, (f) CCOF2-Boc, (h) CCOF1, and (j) CCOF2. The PLD and LCD were calculated using ZEO++. For all CCOFs, the PLD and LCD values of the AA-eclipsed models match the BET-derived pore sizes (Supplementary Fig. 7) better than those of the AB-staggered models, suggesting that these CCOFs are more likely to adopt AA-eclipsed stacking.

Comment 4: Therefore, although the manuscript has been substantially improved and

the catalytic performance is certainly interesting, I do not believe that the structural characterization currently reaches the level of rigor expected for publication in *Nature Communications*. In my opinion, the work would be more appropriate for publication in a specialized catalysis journal, where the catalytic findings could still be appreciated, even if the structural characterization remains less definitive than required for a high-impact general journal;

Response: We respectfully thank the reviewer for this candid assessment and for recognizing the significance of the catalytic performance. We fully appreciate that *Nature Communications* requires both a compelling conceptual advance and rigorous support for the structural claims. In response, we have substantially revised the manuscript to ensure that the structural conclusions are presented at a level of certainty justified by the data.

Specifically, we have added Rietveld refinements, compared AA-eclipsed and AB-staggered models, calculated PLD/LCD values for both stacking models, revised the explanation of low BET surface areas, and distinguished more clearly between experimental observations and structural interpretation. We agree that the CCOFs are not single-crystalline materials and that some samples contain appreciable disorder. The revised manuscript therefore does not present the structures as definitively solved in an absolute crystallographic sense. Instead, it presents the AA-eclipsed model as the most consistent structural description based on converging evidence from PXRD refinement, pore-size analysis, HRTEM, FTIR, solid-state NMR, elemental analysis, nitrogen sorption, and post-synthetic stability tests.

We also wish to emphasize that the main advance of the manuscript is not solely the crystallographic assignment of a new COF structure, but the development of a multivariate design strategy that converts unstable binary chiral COFs into more ordered, porous, metal-binding, and leaching-resistant catalytic frameworks. The structure–function relationship is directly supported by comparative experiments: CCOF2-Ru shows higher crystallinity and porosity, more stable Ru coordination, lower Ru leaching, and superior catalytic activity/recyclability compared with the binary and less ordered analogues. In addition, XPS and EXAFS support atomically dispersed Ru coordination within the chiral diamine environment, while recycling and leaching experiments demonstrate practical catalyst robustness.

We hope that the further revised manuscript, with strengthened analysis and appropriately qualified structural claims, now provides a sufficiently rigorous and balanced basis for publication in *Nature Communications*.

Response to the comments

Response to the Editor

We thank the Editor for the opportunity to submit a final revision of our manuscript. In response to the remaining concerns of Reviewer #3, we have carefully re-examined the PXRD, solid-state NMR, HRTEM, and TGA data and revised the manuscript and Supplementary Information accordingly.

The solid-state NMR spectra are now presented separately and include the spectrum of the chiral monomer. The spectral assignments and the interpretation of the TGA mass-loss regions have also been clarified. We have also revised the structural discussion throughout the manuscript to present the AA-eclipsed arrangement as the model most consistent with the combined experimental and computational evidence, while acknowledging the resolution limits of the current PXRD data and the value of higher-resolution structural studies in the future.

During the final cross-check, we also corrected several minor inconsistencies in sample labels, figure and table cross-references, and the reporting of experimental details. These corrections do not affect the results or conclusions of the manuscript.

Reviewer #3:

I checked the whole manuscript again and agreed with the reviewer 1 that the structures of the COFs are unclear. The authors should check the related characterizations carefully. The authors should discuss with the structure experts about their results.

Response: We thank the Reviewer for the careful re-evaluation of our manuscript. Following the Reviewer's suggestion, we re-examined the PXRD data, structural models, solid-state NMR spectra, TGA profiles and related characterization results. We have revised the manuscript to distinguish more clearly between direct experimental observations and model-dependent structural interpretation. In particular, the AA-eclipsed arrangement is now described as the structural model most consistent with the combined experimental and computational evidence, rather than as a uniquely determined crystal structure. The detailed revisions are provided below.

Comment 1: At present, the PXRD results do not clearly indicate that the synthesized COF is crystalline, particularly with regard to the diffraction peaks from the (100) crystal plane. It is recommended to further characterize the structure of the COF using small-angle X-ray diffraction (SAXRD) analysis;

Response: We appreciate the Reviewer's concern regarding the evidence for periodic order and the assignment of the dominant low-angle reflection. We clarify that the reported powder X-ray diffraction data were collected using Cu Kα radiation in a low-angle laboratory PXRD configuration over 2θ = 1.5-30°, with a slit width of 0.1 mm and a scan rate of 10° min^{-1}. These acquisition parameters have now been added to Section S1 of the Supplementary Information. Although a dedicated SAXRD measurement could not be performed for this revision, we have revised the structural interpretation to reflect the resolution limits of the current data.

The Boc-protected and ternary CCOF samples exhibit reproducible low-angle diffraction features centered at approximately 2.1-2.3°, together with weaker higher-order features for selected samples. These observations support periodic in-plane order on the length scale expected for the proposed frameworks. Comparison with simulated diffraction profiles indicates that the positions of these reflections are consistent with the evaluated AA-eclipsed models. HRTEM provides complementary local evidence of periodic ordering, including lattice fringes and channel-like projections in selected regions.

We agree that the weak higher-order reflections limit the certainty of the stacking assignment. The manuscript has therefore been revised to present the AA-eclipsed arrangement as the model most consistent with the combined PXRD, porosity, and microscopy data. Local stacking disorder cannot be excluded. We have also added an explicit statement that future synchrotron powder diffraction, total-scattering analysis, or three-dimensional electron diffraction would be valuable for resolving the local stacking arrangement more definitively.

Manuscript Change:

Manuscript (revised text, page 9):

"Taken together, the PXRD, HRTEM, and gas-sorption results support the formation of porous 2D frameworks with periodic in-plane order. The combined experimental data are most consistent with the evaluated AA-eclipsed stacking model, although small layer offsets, stacking faults, and local disorder cannot be excluded. A more definitive determination of the local stacking arrangement will require future higher-resolution structural characterization, such as synchrotron powder diffraction, total-scattering/pair-distribution-function analysis, or three-dimensional electron diffraction."

Supporting Information (revised PXRD measurement details, page S3):

"Powder X-ray diffraction (PXRD) patterns were collected on a Bruker D8 Advance diffractometer using Cu Kα radiation in a low-angle configuration over a 2θ range of

1.5–30°, at a scan rate of 10° min^{-1}, with the slit width set to 0.1 mm."

Comment 2: It is recommended to plot the solid-state NMR spectra separately; the current plotting format makes it difficult to distinguish the characteristic peaks of the various functional groups. Additionally, it is necessary to perform SSNMR characterization of the monomers prior to synthesis;

Response: We thank the Reviewer for this constructive suggestion. The solid-state ^{13}C CP/MAS NMR spectra are now presented separately in Supplementary Fig. 3 to allow clearer identification and comparison of the characteristic resonances. We have also added the spectrum of the isolated chiral monomer, (*R,R*)-DPEN-NH-Boc, which provides the most directly relevant reference for assigning the Boc- and DPEN-derived resonances in the CCOFs.

In revising the figure, we re-examined and revised the signal assignments. The resonances at approximately 156, 80, and 29 ppm are assigned to the Boc carbamate carbonyl, quaternary carbon, and tert-butyl methyl carbons, respectively, whereas the resonances at approximately 62 and 49 ppm arise primarily from the DPEN-derived stereogenic carbons. Following deprotection, the Boc-associated resonances are substantially reduced or no longer clearly resolved, whereas the DPEN-derived signals remain. The corresponding discussion and figure caption have been revised accordingly.

Manuscript Change:

Manuscript (revised text, page 5):

"Solid-state ^{13}C cross-polarization magic-angle spinning (CP/MAS) NMR corroborated imine carbons (~158 ppm) and signals associated with the incorporated DPEN-derived unit near approximately 62 and 49 ppm (Supplementary Fig. 3). The Boc-protected samples additionally exhibit resonances near approximately 156, 80, and 29 ppm, assigned to the carbamate carbonyl, quaternary carbon, and tert-butyl methyl carbons, respectively. These Boc-associated signals decrease markedly or disappear following deprotection, whereas the DPEN-derived carbon signals remain, supporting Boc removal without loss of the chiral linker (Fig. 2b)."

Supporting Information (revised Supplementary Fig. 3, page S36):

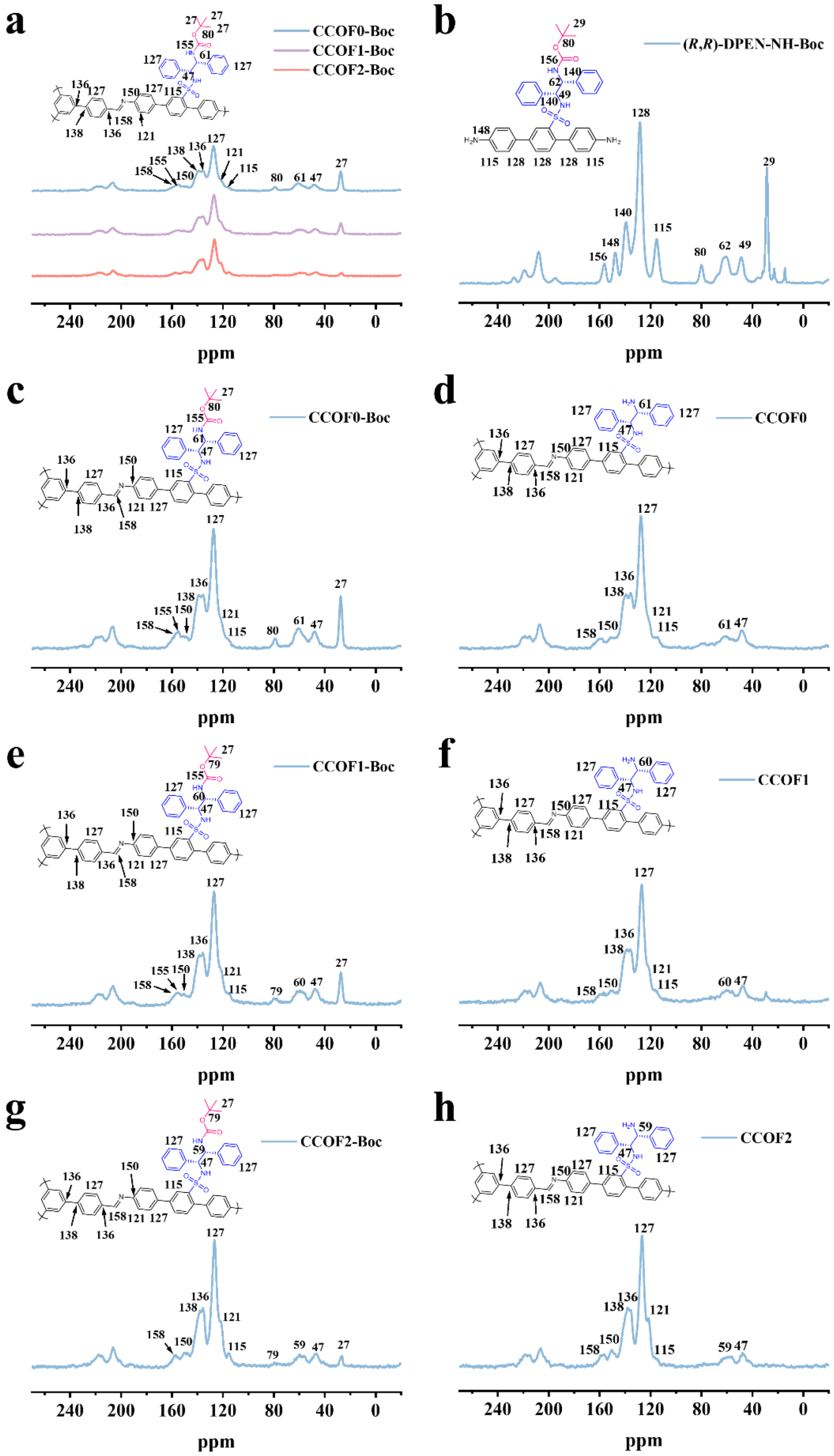

Supplementary Fig. 3 solid-state ^{13}C CP/MAS NMR spectra of the chiral monomer and COF samples. **a** Stacked comparison of the Boc-protected CCOFs, **b** (*R*,*R*)-DPEN-NH-Boc, **c** CCOF0-Boc, **d** CCOF0, **e** CCOF1-Boc, **f** CCOF1, **g** CCOF2-Boc and **h** CCOF2. The resonances at approximately 156, 80, and 29 ppm are assigned to the Boc

carbamate carbonyl, quaternary carbon, and tert-butyl methyl carbons, respectively, whereas those at approximately 62 and 49 ppm are associated primarily with the stereogenic carbons of the DPEN-derived unit. Following deprotection, the Boc-associated resonances are substantially attenuated or no longer clearly resolved, whereas the DPEN-derived signals remain, with some broadening and small chemical-shift changes. In CCOF2-Boc, the Boc carbamate-carbonyl resonance is not resolved as a separate signal, likely because of overlap with nearby framework resonances.

Comment 3: The TGA results should be confirmed again;

Response: We thank the Reviewer for prompting us to re-examine the TGA data and their interpretation. Upon re-examining the TGA profiles, we refined the terminology and adopted more cautious assignments of the mass-loss regions.

First, we have removed the term "sulfonated clusters," because the CCOFs contain sulfonamide-bearing chiral groups rather than discrete sulfonated clusters. The Boc-protected samples exhibit an additional mass-loss feature beginning at approximately 210 °C that is markedly reduced in the corresponding deprotected samples. On the basis of this comparison, this feature is assigned to decomposition of the Boc-containing groups.

A further mass-loss region near approximately 275 °C is observed for the chiral CCOF samples but is less evident for the TFPB-DATP control, which lacks the chiral sulfonamide-containing component. We therefore describe this feature as tentatively associated with decomposition of the pendant chiral sulfonamide-containing groups. At temperatures above approximately 480 °C, all frameworks undergo substantial mass loss consistent with major framework decomposition and carbonization under nitrogen. Because evolved-gas analysis was not performed, the assignments of the individual mass-loss regions are now explicitly described as tentative. The corresponding text and the caption of Supplementary Fig. 9 have been revised.

Manuscript Change (revised text, page 8):

"As shown in Supplementary Fig. 9, the Boc-protected CCOF samples exhibit an obvious mass loss starting at approximately 210 °C that is markedly reduced in the corresponding deprotected samples, attributed to thermal deprotection of the Boc groups. A further mass-loss region near approximately 275 °C is observed for the chiral CCOF samples but is less evident for the TFPB-DATP control, which lacks the chiral sulfonamide-containing component. This feature is therefore tentatively associated with decomposition of the pendant chiral sulfonamide-containing groups.[65, 66] At temperatures above approximately 480 °C, all frameworks, including the CCOFs and

the TFPB-DATP control, undergo substantial mass loss, which is consistent with major framework decomposition and carbonization under nitrogen. "

Reviewer #4

The authors have addressed all reviewers' comments very well. I strongly support the publication of this manuscript.

Response: We thank the Reviewer for the positive assessment and support for publication.

We thank the Editor and Reviewers again for their careful and constructive evaluation. We believe that the revisions have fully addressed their comments and substantially improved the clarity and rigor of the manuscript. We hope that the revised manuscript is now suitable for publication in *Nature Communications*.
